
JOURNAL INFORMATION
==============================
NLM Title Abbreviation: Trials
Journal ID: Trials
NLM Journal ID: 101263253

Trials

EISSN: 1745-6215
Publisher: BMC

ARTICLE INFORMATION
==============================
PMCID: PMC6805286
PMID: 31639031
DOI: 10.1186/s13063-019-3688-6
Article ID: 3688
Article version: 1
Subjects: Meeting Abstracts

Meeting abstracts from the 5th International Clinical Trials Methodology Conference (ICTMC 2019)

Brighton, UK. 06-09 October 2019

Electronic publication date: 2019 Oct 22
Publication date: 2019
Volume: 20
Issue: Suppl 1
Issue sponsor: The publication charge for this supplement was not funded by any sponsorship.
Electronic Location ID: 579
Copyright: © The Author(s). 2019
License: Open AccessThis article is distributed under the terms of the Creative Commons Attribution 4.0 International License (http://creativecommons.org/licenses/by/4.0/), which permits unrestricted use, distribution, and reproduction in any medium, provided you give appropriate credit to the original author(s) and the source, provide a link to the Creative Commons license, and indicate if changes were made. The Creative Commons Public Domain Dedication waiver (http://creativecommons.org/publicdomain/zero/1.0/) applies to the data made available in this article, unless otherwise stated.
License URL: https://creativecommons.org/licenses/by/4.0/

Conference name: 5th International Clinical Trials Methodology Conference
Conference Acronym: ICTMC 2019
Conference location: Brighton, UK
Conference date: 06-09 October 2019
issue-copyright-statement: © The Author(s) 2019

==============================

## P-1
**Abstract omitted**

## P-2
**Some practical considerations in the design of multi-arm multi-stage designs**

Jerome Wulff, Nikolaos Demiris
Cambridge Clinical Trial Unit, Cambridge, United Kingdom
*Trials* 2019, **20(Suppl 1):**P-2

**Introduction:** In the design of cancer clinical trials, one is often concerned with a number of options in the event that several treatments are of interest.
**Methods:** We explore in this work the distinct possibilities when four treatments are available, one acting as control and three as potentially efficacious alternatives. This design may be embedded within the context of multi-arm multi-stage (MAMS) trials where one may select a two- or three-stage design.
**Potential Results:** We explore the application of such designs, including trade-offs between potential gains in the number of patients with additional stages contrasted with patients "lost" due to practical considerations such as patients randomised in dropped arms while waiting for interim analyses and inspection by an Independent Data and Safety Committee. In addition, in cancer studies one may focus on the primary end-point using a time-to-event analysis or a binary outcome by looking at the probability of (potentially progression-free) survival at a specific, clinically meaningful, time point. The effect of such choices is extensively investigated.
**Potential Relevance & Impact:** We conclude with a discussion of the available software for MAMS designs and their advantages and disadvantages in terms of accuracy.

## P-3
**The UK plasma based Molecular profiling of Advanced breast cancer to inform Therapeutic CHoices (plasmaMATCH) Trial: A multiple parallel-cohort, phase IIa platform trial aiming to provide proof of principle efficacy for designated targeted therapies in patient subgroups identified through ctDNA screening (CRUK/15/010)**

Sarah Kernaghan[1], Laura Moretti[1], Lucy Kilburn[1], Katie Wilkinson[1], Claire Snowdon[1], James Morden[1], Iain Macpherson[2], Andrew Wardley[3], Rebecca Roylance[4], Richard Baird[5], Alistair Ring[6], Nicholas Turner[7], Judith M Bliss[1], on behalf of the plasmaMATCH Trial Management Group
[1]Clinical Trials and Statistics Unit at The Institute of Cancer Research (ICR-CTSU), United Kingdom; [2]The Beatson West of Scotland Cancer Centre, Glasgow, United Kingdom; [3]The Christie NHS Foundation Trust, Manchester, United Kingdom; [4]University College London Hospitals NHS Foundation Trust, London, United Kingdom; [5]Cambridge University Hospitals NHS Foundation Trust, Cambridge, United Kingdom; [6]The Royal Marsden NHS Foundation Trust, Sutton, United Kingdom; [7]The Institute of Cancer Research and The Royal Marsden NHS Foundation Trust, London, United Kingdom
*Trials* 2019, **20(Suppl 1):**P-3

**Introduction:** plasmaMATCH is a novel platform trial which assesses the potential of circulating tumour DNA (ctDNA) screening to direct targeted therapies in advanced breast cancer (ABC) patients. The trial recruited ahead of target and will report initial results within 3years of first patient first visit demonstrating efficiency of this design.
**Methods:** plasmaMATCH is an open-label, multi-centre phase IIa platform trial, consisting of a ctDNA screening component and five parallel treatment cohorts. Patients with an actionable mutation identified at ctDNA screening are invited to enter Cohorts A-D to receive a targeted treatment matched to the mutation identified (A: ESR1–extended-dose fulvestrant; B: HER2–neratinib+/-fulvestrant; C&D: AKT1 (or PTEN for Cohort D) –AZD5363+/-fulvestrant). Cohort E was added later to recruit patients with triple negative BC with no actionable mutation identified by ctDNA screening to receive olaparib+AZD6738. ~1150 patients will be screened, with 195 evaluable patients entered into cohorts (A–78; B–16; C–16; D–16; E–maximum 69). Each cohort will be analysed independently. The primary endpoint for Cohorts A–E is confirmed objective response rate by RECIST v1.1. Secondary endpoints include clinical benefit rate, progression-free survival, safety and frequency of mutations identified in ctDNA screening.
**Timing of potential results:** Screening for Cohort A closed in March-2019 and for B-D in April-2019. ctDNA screening and Cohorts A-D results will be presented in Q4-2019.
**Potential relevance & impact:** plasmaMATCH is a successfully recruiting platform trial that seeks to determine the efficiency of the dynamic trial platform design in providing proof of principle efficacy for designated targeted therapies. plasmaMATCH also seeks to demonstrate utility of ctDNA as a screening tool for ABC patients, with the aim of future integration into routine clinical practice. Details of the novel trial design will be presented with illustrations of trial innovation and efficiencies. Clinical outcome data will not be presented.

## P-4
**Abstract omitted**

## P-5
### Optimising hypothesis tests of efficacy in external pilot trials using Bayesian statistical decision theory
Duncan T. Wilson, Rebecca E. A. Walwyn, Julia Brown, Amanda J. Farrin
Leeds Institute of Clinical Trials Research, University of Leeds, Leeds, UK
*Trials* 2019, **20(Suppl 1):**P-5

**Introduction:** External pilot trials of complex interventions are often conducted in advance of a definitive trial to assess feasibility and to inform its design. The efficacy of the intervention is rarely assessed using a formal hypothesis test since it would have low power, given the small sample size of a pilot and assuming a conventional type I error rate (e.g. 0.05). An external pilot not testing efficacy will effectively have a type I error rate of 1, suggesting an infinite preference for type I errors over type II errors. As such a preference will never occur in practice, we consider methods for finding the optimal balance of type I and II error rates in external pilots.
**Methods:** We consider the problem of determining the sample size and type I error rate which maximise the expected utility of an external pilot trial testing intervention efficacy. We introduce a utility function which accounts for improvement in primary outcome, the cost of sampling, treatment costs, and the decision-maker's attitude to risk. We apply the method to the re-design of a pilot trial with a continuous primary outcome with known standard deviation and where uncertainty in the treatment effect is quantified using a normal prior distribution.
**Timing of potential results:** A study of the proposed method's properties under a range of values for the utility function and prior distribution parameters is to be completed by August 2019.
**Potential relevance and impact:** By viewing external pilot trial design from a Bayesian decision-theoretic viewpoint, we will provide a method for finding the optimal balance of type I and II error rates in external pilots. In particular, we will identify in which (if any) settings the current approach of not assessing efficacy is the optimal course of action.

## P-6
### Rare Disease Clinical Trials: Using a continuous covariate to allocate patients in a response-adaptive clinical trial
Holly Jackson
Lancaster University, United Kingdom
*Trials* 2019, **20(Suppl 1):**P-6

**Introduction:** The randomised controlled trial (RCT) is the conventional method used in a clinical trial, as it produces large power. However, the RCT gives no opportunity to change the treatment allocation probability within the trial. In many rare disease clinical trials, a large proportion of the patient population is entered into the trial. Hence, a response-adaptive design can change the probability of each patient receiving a treatment, to prioritise the health of those patients within the trial. The aim of these trials is not just to determine if a new treatment is safe and effective, but also, to treat as many patients as successfully as possible. Response-adaptive designs are not often used as they produce a low power and many of them do not consider the patient's covariates.
**Method:** We present a response-adaptive method, using a continuous covariate and a non-parametric regression procedure to allocate patients to the best treatment for them with varying probability. This method starts with 0.5 allocation probability to their estimated best treatment, but increases to 0.9 for the last patient who enters the trial. We evaluated the method against an RCT using simulations.
**Results:** This method produces more patient successes than the RCT in all scenarios. A number of these scenarios involved the best treatment changing depending on the patient's covariate. In these scenarios, we split the trial depending on the patient's covariate and calculated the power. The power of this method is at least 82.7% of the power of the RCT for all scenarios.
**Discussion:** Future work will include testing this method for many more scenarios to see in which situations it works best. We would also like to extend this method to involving multiple covariates (including both continuous and binary) to make its use in clinical trials more realistic.

## P-7
### Introducing the Adaptive designs CONSORT Extension (ACE) Statement to improve reporting of randomised trials that use an adaptive design
Munya Dimairo[1], Philip Pallmann[2], James Wason[3,4], Susan Todd[5], Thomas Jaki[6], Steven A. Julious[1], Adrian P. Mander[3], Christopher J. Weir[7], Franz Koenig[8], Marc K. Walton[9], Jon P. Nicholl[1], Elizabeth Coates[1], Katie Biggs[1], Toshimitsu Hamasaki[10], Michael A. Proschan[11], John A. Scott[12], Yuki Ando[13], Daniel Hind[1], Douglas G. Altman[14]
[1]School of Health and Related Research, University of Sheffield, Sheffield, United Kingdom; [2]Centre for Trials Research, Cardiff University, Cardiff, United Kingdom; [3]MRC Biostatistics Unit, University of Cambridge, Cambridge, United Kingdom; [4]Institute of Health and Society, Newcastle University, Newcastle upon Tyne, United Kingdom; [5]Department of Mathematics and Statistics, University of Reading, Reading, United Kingdom; [6]Department of Mathematics and Statistics, Lancaster University , Lancaster, United Kingdom; [7]Edinburgh Clinical Trials Unit, Usher Institute of Population Health Sciences and Informatics, University of Edinburgh, Edinburgh, United Kingdom; [8]Centre for Medical Statistics, Informatics, and Intelligent Systems, Medical University of Vienna, Austria; [9]Janssen Pharmaceuticals, United States of America; [10]National Cerebral and Cardiovascular Center, Japan; [11]National Institute of Allergy and Infectious Diseases, National Institutes of Health, United States of America; [12]Division of Biostatistics in the Center for Biologics Evaluation and Research, Food and Drug Administration, United States of America; [13]Pharmaceuticals and Medical Devices Agency, Japan; [14]Centre for Statistics in Medicine, University of Oxford, United Kingdom
*Trials* 2019, **20(Suppl 1):**P-7

**Background:** The reporting of adaptive designs (ADs) in randomised trials is inconsistent and needs improving [1]. Incompletely reported AD randomised trials are difficult to reproduce and are hard to interpret and synthesise. This consequently hampers their ability to inform practice as well as future research and contributes to research waste. Better transparency and adequate reporting will enable the potential benefits of ADs to be realised.
**Methods:** We developed an Adaptive designs CONSORT Extension (ACE) guideline through a two-stage Delphi process with input from multidisciplinary key stakeholders in clinical trials research in the public and private sectors from 21 countries, followed by a consensus meeting [1]. Delphi survey response rates were 94/143 (66%), 114/156 (73%), and 79/143 (55%) in round one, two and across both rounds, respectively. Members of the CONSORT Group were involved during the development process.
**Results:** The resultant ACE checklist is comprised of seven new items, nine modified items, six unchanged items for which additional explanatory text clarifies further considerations for ADs, and 20 unchanged items not requiring further explanatory text. The ACE abstract checklist has one new item, one modified item, one unchanged item with additional explanatory text for ADs, and 15 unchanged items not requiring further explanatory text. The ACE guideline contains minimum essential reporting requirements and it applies to both frequentist and Bayesian ADs in randomised trials.
**Discussion:** The intention is to enhance transparency and improve reporting of AD randomised trials to improve the interpretability of their results and reproducibility of their methods, results and inference. We also hope indirectly to facilitate the much-needed knowledge transfer of innovative trial designs to maximise their potential benefits.

## P-8

### The PITHIA trial: a stepped wedge, cluster randomised, registry based national trial with economic evaluation

Laura A Pankhurst[1], Emma Laing[1], Helen L Thomas[1], Alison J Deary[1], Karla Hemming[2], Dominic M Summers[3], John OO Ayorinde[3], Edward CF Wilson[4], Victoria Bardsley[5], Desley A H Neil[6], Gavin J Pettigrew[3]
[1]Clinical Trials Unit, NHS Blood and Transplant , Cambridge and Bristol, United Kingdom; [2]Department of Public Health, University of Birmingham, Birmingham, United Kingdom; [3]Department of Surgery, University of Cambridge, Cambridge, United Kingdom; [4]Health Economics Group, Norwich Medical School, University of East Anglia, Norwich, United Kingdom; [5]Cambridge University Hospitals NHS Foundation Trust, United Kingdom; [6]Department of Histopathology, University Hospitals Birmingham NHS Foundation Trust, United Kingdom
*Trials* 2019, **20(Suppl 1):**P-8

**Introduction:** The Pre-Implantation Trial of Histopathology In renal Allografts (PITHIA) will assess whether a national, 24-hour, digital histopathology service increases the number, and improves outcomes, of kidneys transplanted in the UK from older deceased donors.
**Methods:** PITHIA is a stepped-wedge cluster randomised study, involving all UK adult kidney transplant centres. At 4-monthly intervals, a group of randomly selected centres will be given access to urgent histopathology: centres can request biopsies of kidneys from donors aged over 60, as required. Biopsies are reviewed by specialist renal histopathologists, who provide a Remuzzi score showing the extent of chronic damage. The score provided may be used by centres to decide whether and how the kidney may be used. The trial is open, and it is anticipated that over 2000 kidneys will be eligible during the 24-month trial duration.
**Results:** The trial has two primary end points: proportion of primary kidney offers transplanted and kidney function 12 months post-transplant. The trial will be analysed using mixed effects models allowing for clustering within centres and adjusting for secular trends. Results will inform a decision-model based economic evaluation to determine whether it is cost-effective.
The trial is registry based; hence the majority of the data can be drawn from the UK Transplant Registry (UKTR) held by NHS Blood and Transplant. The UKTR collects survival and covariate data on all patients undergoing transplantation. The design allows patients to be followed up using only data that is collected routinely. Only one additional data collection form is required, to record and report the histopathology information to the requesting centre.
**Conclusion:** The PITHIA trial is using a stepped-wedge design to include all centres in an evaluation of a new service. The registry based design is novel in transplantation, and is low cost to implement with high levels of data completeness.

## P-9

### Issues in the design, analysis, and reporting of factorial trials: a review

Diana Elbourne[1], Brennan C Kahan[2], Elaine M Beller[3], Michael Tsui[4], Vipul Jairath[5], Douglas Altman[6]
[1]LSHTM, London, United Kingdom; [2]Queen Mary University of London, London, United Kingdom; [3]Bond University, New South Wales, Australia; [4]Schulich School of Medicine and Dentistry, Ontario, Canada; [5]University of Western Ontario, Ontario, Canada; [6]Centre for Statistics in Medicine, Oxford, United Kingdom
*Trials* 2019, **20(Suppl 1):**P-9

**Introduction:** Factorial designs can allow efficient evaluation of multiple treatments within a single trial. We report the quality of the design, analysis, and reporting in a sample of factorial trials.
**Methods:** A 6-person team from the UK, Australia and Canada reviewed 2x2 factorial trials evaluating health-related interventions and outcomes in humans. Using MEDLINE, we identified articles published between January 2015 and March 2018. We randomly selected 100 articles for inclusion.
**Results:** Few trials (22%) provided a rationale for using a factorial design. Only 63 trials assessed interaction for the primary outcome, and only 39/63 (62%) made a further assessment for at least one secondary outcome. 12/63 trials (19%) identified a significant interaction for the primary outcome, and 16/39 trials (41%) identified a significant interaction for at least one secondary outcome. Inappropriate methods of analysis to protect against potential negative interaction effects were common, with 18 (18%) of trials choosing an analysis method based on a preliminary test for interaction, and 13% (n=10/75) of authors conducting a factorial analysis including an interaction term in the model.
**Conclusions:** Reporting of factorial trials was often suboptimal, and assessment of interactions was poor. Investigators often used inappropriate methods of analysis to try to protect against adverse effects of interactions. The CONSORT Group (Consolidated Standards of Reporting Trials) has developed guidelines to alleviate problems arising from inadequate reporting of RCTs. The results of this review suggest items of the CONSORT statement that can be extended for factorial trials.

## P-10

### Designing a multi-arm multi-stage trial in progressive multiple sclerosis

Baptiste Leurent[1], Frederik Barkhof[2], Olga Ciccarelli[2], Arman Eshaghi[2], Emma Gray[6], Vivien Li[5], Jennifer Nicholas[1], Nigel Stallard[3], James Wason[4], Fay Cafferty, Jeremy Chataway[2,5]
[1]London School of Hygiene and Tropical Medicine; [2]University College London; [3]University of Warwick; [4]Newcastle University; [5]University College London Hospitals; [6]MS Society
*Trials* 2019, **20(Suppl 1):**P-10

**Introduction:** Multiple sclerosis affects more than 100,000 people in the UK, with few effective treatments for the progressive stage of the disease (PMS). Multi-Arm Multi-Stage (MAMS) trials may accelerate treatment discovery in PMS, as done with success in other disease areas. MAMS are adaptive trials characterised by multiple experimental arms, and multiple interim analyses, where treatments with insufficient indication of efficacy are discontinued. MAMS designs can provide efficiencies, particularly in terms of duration and sample size, but their preparation is more complex.
The aim of this research was to explore designs for a feasible and efficient MAMS trial in PMS.

**Methods:** We simulated trials with a correlated interim (e.g. brain atrophy) and final outcome (e.g. time to disability progression). Data from earlier PMS trials were used to determine parameters such as the association between the outcomes. We explored different design options, including choices for interim and final outcomes, timing of interim analyses, and stopping rules. Under each scenario, trials were simulated and operating characteristics (sample size, duration, type-I and type-II error rates) graphically displayed.
**Timing of Potential Results:** Simulations are ongoing and will be completed by August 2019. Simulations have been informed by analysis of two-phase II trials but will be refined with results from ongoing analysis of several larger phase III trials. Preliminary results suggest that multiple interim analyses could be beneficial to better balance the trade-off between stopping ineffective treatments early and the risk of missing effective ones.
**Discussion:** Designing a MAMS trial presents several complexities. To date simulations are key to inform decisions such as the appropriate outcome and time-point for the interim analyses. The findings will inform the optimum trial design to maximise the chance of identifying effective treatments for PMS and should be instructive in trial design in other neurodegenerative diseases such as dementia.

## P-11
**Abstract omitted**

## P-12
### Protocol for a pragmatic cluster randomised controlled trial assessing the clinical effectiveness and cost effectiveness of electronic risk-assessment tools for cancer for patients in general practice (ERICA)

Raff Calitri[1,2], Luke Mounce[2], Gary Abel[2], John Campbell[2], Anne Spencer[2], Antonieta Medina-Lara[2], Martin Pitt[2], Elizabeth Shepard[2], Fiona Warren[2], Sarah Dean[1,2], Willie Hamilton[2]
[1]Exeter Clinical Trials Unit, University of Exeter, Exeter, United Kingdom; [2]College of Medicine & Health, University of Exeter, Exeter, United Kingdom
*Trials* 2019, **20(Suppl 1):**P-12

**Introduction:** Compared with other developed countries, the UK has poorer cancer outcomes. Early cancer diagnosis within general practice has the potential to facilitate improvements. Paper and mouse mat Risk Assessment Tools (RATs) for 18 cancers have been developed to support GPs in identifying cancer. The RATs give precise estimates of the risk of an underlying cancer based on a single symptom or combination of symptoms. Some of the RATs have been converted into electronic versions (eRATs) and embedded into GPs' clinical systems, delivering an automated prompt to consider the possibility of cancer when a patient has at least a 2% risk of cancer. Early pilot work suggests that the eRATs are acceptable to GPs. There is no evidence to date of their clinical- or cost-effectiveness.
**Methods:** A pragmatic, cluster RCT with 530 practices across England randomised 1:1 to receive either the intervention (access to the eRATs medical device including: lung, oesophago-gastric, kidney, bladder, ovarian, colorectal) or usual practice. There will also be embedded process and health economics evaluations along with a parallel study modelling the impact of eRATs on NHS service delivery. Clinical outcomes will be observed in routinely collected data exported from the cancer registry. The primary outcome will be the proportion of the combined six cancers diagnosed during a 2-year follow-up that were at Stage 1/2 (early – cure likely) versus Stage 3/4 (late – cure not likely) at the time of diagnosis. Ethics approval and trial registration will be sought in the early spring 2019. Practice recruitment is planned to launch in summer 2019 and close in winter 2019.
**Results:** Results will be available from winter 2023.
**Discussion:** The results of the RCT will provide a definitive assessment of the clinical- and cost-effectiveness of the six eRATs being studied and report their impact on patient care.

## P-13
### Testing the Feasibility of a Complex Intervention for Perinatal Mental Health in The Gambia

Katie Rose M Sanfilippo[1], Victoria Cornelius[2], Bonnie McConnell[3], Paul Ramchandani[4], Ian Cross[4], Hassoum Ceesay[5], Buba Darboe[6], Hajara B Huma[5,6], Malick Gaye[5,6], Vivette Glover[2], Lauren Stewart[1]
[1]Goldsmiths, University of London, London, United Kingdom; [2]Imperial College London, London, United Kingdom; [3]Australian National University, Canberra, Australia; [4]University of Cambridge, Cambridge, United Kingdom; [5]National Centre for Arts and Culture, Banjul, Gambia; [6]Ministry of Health and Social Welfare, Banjul, Gambia
*Trials* 2019, **20(Suppl 1):**P-13

**Introduction:** Perinatal mental health problems affect up to 1 in 5 women worldwide and affect not only the mother but can also have long-term adverse effects on her child. It is thus of high priority to develop new low-cost, low-resource, non-stigmatising and culturally appropriate approaches to reduce symptoms of anxiety and depression perinatally.
**Methods:** We have worked to test the feasibility of undertaking a stepped wedge trial to examine how group singing could be beneficial in alleviating perinatal mental distress in The Gambia. Women in the intervention participated in weekly singing sessions, led by local Kanyeleng singing groups, for six weeks while the control group received standard care. Symptoms of anxiety and depression were measured using self-report questionnaires. The feasibility of the design was assessed through recruitment, retention and attrition rates of participants, clinic's adherence to the schedule and completeness of data by site. Qualitative interviews and video and audio recordings were used to evaluate the acceptability of the intervention.
**Timing of Potential Results:** We will have the final results of this trial by the end of May 2019.
**Potential Relevance & Impact:** When running a trial in a low resource context different challenges are present, such as lack of infrastructure and technology, low literacy rates, and different cultural expectations, as well as affordances, such as high levels of willingness to help and the ability to quickly affect policy. In this presentation, we will discuss how the design of the trial was planned and how the implementation of this design was achieved. This trial's findings will allow us to investigate the use of music as a potential intervention for perinatal mental health in The Gambia as well as discuss different methodological techniques which can be applied to low and middle-income countries.

## P-14
**Abstract withdrawn**

## P-15
**Abstract omitted**

## P-16
### Move-It 動起來: Digital worksite exercise in China - outcome and process evaluation

Holly Blake[1,3], Betsy Lai[2], Jonathan Houdmont[2], Amanda Griffiths[2]
[1]School of Health Sciences, University of Nottingham, Nottingham, United Kingdom; [2]School of Medicine, University of Nottingham, Nottingham, United Kingdom; [3]NIHR Nottingham Biomedical Research Centre, Nottingham, United Kingdom
*Trials* 2019, **20(Suppl 1):**P-16

**Introduction:** Developing strategies to promote exercise is a major health priority in China. Integrating exercise within the working day may benefit employee health, although workplace interventions are less commonplace in China. We evaluate the outcomes and processes of a video and web-based worksite exercise intervention for sedentary office workers in China.
**Methods:** Participants were recruited from an insurance information technology service organisation with sites in two major cities in China. A theoretically informed digital workplace intervention (Move-It [illegible]) involving a 10-minute Qigong exercise session (video demonstration via website) was delivered twice a day at set break times during the working day for 12 consecutive weeks. The outcome study was a cluster-randomised wait-list control trial with outcomes assessed in two groups (intervention, n = 143; wait-list control, n = 73). Process evaluation was conducted using the RE-AIM framework: reach, effectiveness, adoption, implementation and maintenance. Data from employee exercise logs, six focus groups with employees and managers, and analysis of documents including employee profiles and promotional materials were examined.
**Results:** Employees' physical activity increased from baseline to post-intervention in both the intervention and control group, though the magnitude of change failed to reach statistical significance. There were no changes in job performance or weekday sitting hours. Process evaluation showed that the intervention had wide reach and was successfully marketed to all employees with good uptake. The participatory approach increased perceived organisational support and enhanced adoption. The intervention was implemented broadly as planned, with employee enthusiasm for long-term maintenance but no concrete plans in place at study sites.
**Discussion:** Qigong worksite exercise intervention can be successfully delivered to sedentary office workers in China using video and web-based platforms and may increase physical activity although further outcome trials are required. The study highlights the complexity of conducting health research in real-world organisational settings.

## P-17

### Improving engagement in a health app: considerations in designing a Micro-Randomised Trial

Lauren Bell[1], Henry Potts[2], Elizabeth Williamson[1]
[1]London School of Hygiene and Tropical Medicine, United Kingdom; [2]University College London, United Kingdom
*Trials* 2019, **20(Suppl 1):**P-17

**Introduction:** Health systems are undergoing a digital revolution, with recent developments seeing therapeutic apps emerging as prescribed treatments. However, a common barrier to the therapeutic app's effectiveness is sustaining user engagement. One feature to increase user engagement is with push notifications, which are messages sent to the user from the app.
This research focuses on Drink Less, a digital therapeutic app which is a complex intervention that aims to help users reduce harmful and hazardous alcohol drinking. The app includes five different therapeutic components and sends a daily push notification at 11am.
**Methods:** Observational data comprises of 25,083 users of 1,108,102 sessions between May 2017 and January 2019. We are exploring patterns of use and engagement with the app through descriptive statistics, graphical summaries and cluster analyses. Results from this exploratory analyses will inform the design of a Micro-Randomised Trial (MRT) which aims to understand the effect of new push notifications as time-varying treatments.
The MRT objective is to optimise the delivery of notifications to increase user engagement by tailoring the message content and timing of delivery to baseline characteristics. Following the randomisation of a notification, our outcome will be time spent on the app (seconds) during the next hour.
**Timing of potential results:** Exploratory analyses of current patterns of use will be finalised at the end of May 2019. The Micro-Randomised Trial protocol will be finalised by the end of August 2019.
Potential relevance and impact
The results will be generalizable to other behaviour change therapeutic apps. This reflects good practice of learning from real world use and brings transparency to the app-developing process which is often considered a 'black-box'. To date, this will be the largest Micro-Randomised Trial undertaken, providing insights to shared experiences, challenges and solutions of clinical trials for developing digital therapies.

## P-18

### Issues with missing data in trials of complex interventions: Using therapy non-compliance, we demonstrate a framework for assessing how to deal with the potential bias caused by missing data, a systematic way of determining which pre-/post-randomisation variables predict missingness in final outcomes and assess the need for multiple imputation

Rachel Holland[1], Sabine Landau[2]
[1]KCL, Greater London, United Kingdom; [2]KCL, Greater London, United Kingdom
*Trials* 2019, **20(Suppl 1):**P-18

**Introduction:** The ACTIB trial [1] followed 558 participants randomly assigned to either telephone delivered CBT (TCBT), web-based CBT (WCBT) in addition to treatment as usual (TAU) or TAU alone. Binary compliance was defined differently for TCBT and WCBT and not assessed for TAU. Therapy non-compliance (primary outcome completion) was for TCBT, WCBT and TAU respectively; 16% (73%) , 31% (67%) and undefined (70%).
**Methods:** We intended to analyse the primary outcomes using longitudinal linear mixed modelling, a method robust to missing data assuming that the mechanism driving missingness was missing at random (MAR), and valid if all potential predictors of missingness have been collected pre-randomisation and incorporated into the analysis model.
Non-compliance with therapy, determined by an independent statistician, was found to be predictive of missing data in both therapy arms (Fisher's exact tests $p<0.001$) invalidating the MAR assumption. We therefore employed MI to accommodate MAR process which includes a post-randomisation variable predicting missingness.
We used a three-step framework (described in detail in [1]);
1.Assess empirically what baseline variables predict missingness using a stepwise forward selection procedure to identify important predictors
2.Empirically assess whether post-randomisation variables predict primary outcome missingness, e.g. therapy compliance. If true then do step 3,
3.Use MI including all variables identified in steps 1 and 2 in the imputation step of the procedure.
**Results:** Imputation resulted in more conservative trial arm differences comparing therapy arms individually with TAU. The attenuation was more pronounced in the WCBT arm which imputed more missing values. This suggests that MI analyses allowing non-compliance to predict later drop can help remove missing data biases.
**Discussion:** MI can be used where post-randomisation variables such as compliance predict missing outcomes. Variables allowed to drive missingness under a MAR assumption should be assessed systematically.

## P-19

### Outcome selection and reporting for innovative surgical procedures and devices: a review of current practice in IDEAL/IDEAL-D studies to inform the development of a core outcome set

Rhiannon Claire Macefield[1], Nicholas Wilson[1], Kerry NL Avery[1], Shelley Potter[1,2]
[1]Bristol Centre for Surgical Research, Bristol Medical School: Population Health Sciences, University of Bristol, Bristol, United Kingdom; [2]Bristol Breast Care Centre, North Bristol NHS Trust
*Trials* 2019, **20(Suppl 1):**P-19

**Introduction:** Evaluation and reporting of innovative surgical procedures and devices has historically been poor. Development of a core outcome set (COS); generic domains to be measured and reported in all studies of surgical innovations, may help to improve safe and transparent evaluation for their introduction into clinical practice. Methods for identifying outcomes for COSs for effectiveness studies are well established, however, these are unlikely to encompass outcomes relevant to innovation.

The IDEAL (Idea, Development, Exploration, Assessment and Long-term monitoring) framework provides a pathway for evaluating surgical innovations. This targeted review examined outcome selection and reporting in self-identified IDEAL studies to identify current practice and inform conceptualisation of domains for a COS.
**Methods:** Electronic database searches for articles citing key IDEAL publications was undertaken. Records with 'IDEAL' in the title/abstract were selected and screened for eligibility. Excluded were systematic reviews, secondary studies, letters/editorials and studies not including humans or surgery. Data were extracted from full-text publications with outcomes extracted verbatim. Descriptive study characteristics including detail on outcome selection and reporting were summarised. Outcomes were reviewed by the study team and iteratively grouped into domains.
**Results:** of 786 records citing key IDEAL papers, 98 (12%) stated 'IDEAL' in the title/abstract. Some 29 (30%) eligible studies (24 reports, 5 protocols) were included. Studies self-identified as IDEAL stage 1 (n=10), 2a (n=8), 2b (n=5), 3(n=1), multiple stages (n=4) or not stated (n=1). Detail on outcome selection and reporting varied considerably across studies. Over 1000 verbatim outcomes were grouped into 30 domains with several unique to innovation (e.g. modifications to procedures/devices; surgeon's experiences).
**Conclusion:** This review further highlights the need to standardise outcome selection and reporting in studies of surgical innovations. Findings have informed a Delphi survey to reach consensus on a COS and reporting guidelines to promote standardised, transparent outcome reporting for safe evaluation.

## P-20

### Managing learning and clustering effects at the design stage in randomised surgical trials: a retrospective cohort of trial funding applications

Elizabeth Conroy[1], Anna Rosala-Hallas[1], Jane M Blazeby[2], Girvan Burnside[1], Jonathan A Cook[3], Carrol Gamble[1,4]
[1]Department of Biostatistics, University of Liverpool, a member of Liverpool Health Partners, Liverpool, United Kingdom; [2]Centre for Surgical Research, Population Health Sciences, Bristol, United Kingdom; [3]Centre for Statistics in Medicine, University of Oxford, Oxford, United Kingdom; [4]North West Hub for Trials Methodology Research, University of Liverpool, Liverpool, United Kingdom
*Trials* 2019, **20(Suppl 1):**P-20

**Background:** Complexities associated with delivering surgical interventions, such as clustering effects, by centre or surgeon, and surgical learning, should be considered at trial design. This work aims to provide insight into current practice in the management, and acknowledgement, of these considerations during the development of randomised surgical trials.
**Methods:** The cohort searched comprised funded applications, within a four year period, from the National Institute of Health Research Health Technology Assessment and Efficacy and Mechanism Evaluation funding streams. Data were extracted on considerations for learning and clustering effects and the driver, funder or applicant, behind these.
**Results:** Fifty applications were eligible. Managing learning through establishing pre-defined centre and surgeon credentials was common. Few applications also planned exploratory analysis of learning within centre (n=1), and surgeon (n=2) specifically. Clustering, by centre and surgeon, was commonly managed through stratifying randomisation, with the majority also planning to subsequently adjust the main analysis (81% for centre stratified and 60% surgeon stratified). One-third of responses to referees contained funder led changes in support of learning and/or clustering consideration.
**Discussion:** This review indicates that researchers are familiar with the need to do consider the impact of learning and clustering, by centres and surgeon, at trial outset. Furthermore, the funder is identified as a potential driver of considerations. Early consideration of these complexities at trial design will ensure that future trials do not have the same shortfalls as the past.

## P-21

### Successful collaboration between a Clinical Trial Unit (CTU) and the National Radiotherapy Trials Quality Assurance (RTTQA) Group to expedite opening a complex phase III trial (PIVOTALboost)

Clare Cruickshank[1], Helen Mayles[2], Olivia Naismith[3], Nicola Snelson[2], Alison Tree[4], John Staffurth[5], Ann Henry[6], Shama Hassan[1], Stephanie Brown[1], Isabel Syndikus[7], Emma Hall[1]
[1]The Institute Of Cancer Research, London, United Kingdom; [2]National Radiotherapy Trials Quality Assurance Group, The Clatterbridge Cancer Centre, Wirral, United Kingdom; [3]National Radiotherapy Trials Quality Assurance Group, The Royal Marsden NHS Foundation Trust, London, United Kingdom; [4]The Royal Marsden NHS Foundation Trust/The Institute of Cancer Research, London, UK; [5]Cardiff University/Velindre Cancer Centre, Cardiff, UK; [6]The Leeds Teaching Hospitals NHS Trust, Leeds, UK; [7]The Clatterbridge Cancer Centre, Wirral, UK
*Trials* 2019, **20(Suppl 1):**P-21

**Introduction:** PIVOTALboost is a multicentre phase III trial which tests pelvic nodal irradiation and focused dose-escalation (with high dose-rate brachytherapy (HDR) or intensity modulated radiotherapy (IMRT) to a boost volume) in the treatment of localised prostate cancer. The complex radiotherapy treatment requires close liaison between the clinical investigators, CTU and the National RTTQA group to minimise deviations from the trial protocol to ensure integrity of trial outcomes. The radiotherapy techniques used within PIVOTALboost were not routine standard of care for all centres and their implementation required a change in practice.
**Methods:** Radiotherapy treatment was accredited using outlining and planning benchmark cases for focal boost and pelvic node treatment and radiotherapy quality assurance (RTQA) guidelines were made available. The benchmarks were sent to participating sites for completion prior to a pre-launch, face-to-face training workshop where experience was shared and tailored feedback given. Agreement was sought between the RTTQA group and CTU on a priority list of centres to open, based on their trial approval progress. Teleconferences were held every 2 weeks between the CTU and RTTQA group and issues or problems were escalated between each team and the priority list amended as necessary.
**Results:** The workshop was well attended with 59 participants from 26 sites (21 sites had managed to submit their benchmark cases for prior review). Regular communication between the CTU and RTTQA group meant that resources were effectively managed with both teams working on an agreed priority centre list. Within the 1st year of PIVOTALboost opening to recruitment, 24 centres were open, and 237 patients had been recruited.
**Discussion:** It is proposed that to meet trial milestones close collaboration between the CTU, RTTQA team and clinical investigators is needed. This includes managing the pre-trial RTQA schedule and this should continue throughout patient recruitment, follow-up and analysis.

## P-22

### Updating recruitment forecasts via simulations to explore impact of availability of experimental arms in the phase III PIVOTALboost trial

Nuria Porta[1], Vicki Hinder[1], Clare Griffin[1], Alison Tree[1,2], John Staffurth[3], Ann Henry[4], Shama Hassan[1], Stephanie Brown[1], Clare Cruickshank[1], Isabel Syndikus[5], Emma Hall[1]
[1]The Institute of Cancer Research, London, United Kingdom; [2]The Royal Marsden NHS Foundation Trust, London, United Kingdom; [3]Cardiff University/Velindre Cancer Centre, Cardiff, United Kingdom; [4]The Leeds Teaching Hospitals NHS Trust, Leeds, United Kingdom; [5]The Clatterbridge Cancer Centre, Wirral, United Kingdom
*Trials* 2019, **20(Suppl 1):**P-22

**Background:** In PIVOTALboost (ISRCTN80146950) localised prostate cancer patients receive standard prostate intensity modulated radiotherapy (IMRT) (A); standard IMRT plus pelvic node IMRT (B); standard IMRT plus prostate boost (C); or standard IMRT plus pelvic node IMRT and prostate boost (D). Allocation to boost arms C and D depends on patient eligibility and having a suitable tumour volume, and boost technologies being available at site. Power was therefore reduced for the boost comparisons, so the overall population will be split 9:9:8:8 across arms. During design, recruitment simulations led to an initial allocation ratio of 2:2:3:3 to counteract early imbalances, with plans to change to 1:1:1:1 after 12-18 months. We assumed 80% patients would have suitable tumour volume, 5% would be ineligible for boost, and sites could open to 2-arm randomisation (AvB) but would change to 4-arm randomisation once boost technologies were implemented at site. Simulations indicated 30% patients would be allocated via 2-arm randomisation by 18 months.
**Methods:** Design simulations assumptions are compared to data available after 16 months of recruitment and updated to estimate end of recruitment.
**Results:** To 1st May 2019, 386 patients have been randomised from 31 sites: 132 (A), 131 (B), 61 (C) and 62 (D). Overall, 66% had suitable boost tumour volume, 31% were ineligible for boost, and 47% have entered via the 2-arm AvB randomisation. Technical challenges such as use of fiducials or new planning methods are reported to be deterring centres to allocate patients to the 4-arm randomisation.
**Discussion:** Changing allocation ratio to 1:1:1:1 is now not appropriate, as it would increase imbalance between arms. Simulation of recruitment is a useful tool both to design and monitor recruitment projections. Assumptions and simulations should be updated as the trial progress to optimise the likelihood of achieving target numbers in an efficient manner.

## P-23

### The HI-Light trial: facilitating at home self-delivery of narrowband-UVB (NB-UVB) light therapy for people with vitiligo

Garry Meakin[1], Rachel Haines[1], Jonathan Batchelor[2], Kim Thomas[2], Jennifer White[1], Joanne Chalmers[2], Paul Leighton[2]
[1]Nottingham Clinical Trials Unit, University of Nottingham, Nottingham, United Kingdom; [2]Centre of Evidence Based Dermatology, University of Nottingham, Nottingham, University of Nottingham
*Trials* 2019, **20(Suppl 1):**P-23

**Introduction:** Vitiligo is a dermatological condition causing loss of pigment on affected areas of the skin. Management includes narrowband-UVB (NB-UVB) light therapy, however this is reserved for widespread vitiligo and is only available in secondary care, requiring regular hospital visits.
The HI-Light trial evaluated handheld NB-UVB light therapy at home for early/limited vitiligo. Treatment carried potentially severe adverse events (AEs), including burns.
**Methods:** For safety, a training package was designed to ensure participants were able to (1) administer the intervention (2) identify AEs, (3) take appropriate action in response to AEs.
'Train the trainer' training took place at site initiation visits. Participant training at baseline lasted on average 1 hour, focused on treatment administration and AE detection and was further reinforced by a take-home training DVD and participant handbook. The handbook included (1) treatment instructions, (2) treatment schedule, (3) description of AEs, (4) AE management guidance, (4) contact information.
Participants received a telephone call two weeks post-randomisation and were encouraged to contact the research team at any time over the 9 month treatment period with questions or concerns. Participants were reviewed face to face at 3, 6 and 9 months. Participant interviews, part of a process evaluation, covered topics relating to training and intervention use.
**Results:** 517 participants were randomised into the trial. There were no treatment related serious AEs and all reported AEs were managed effectively. Nurses were confident in delivering training. Participants had a good understanding of how to use the light device, felt confident and adhered well. The support from nurses when dealing with side-effects was highly valued and no major issues with using the devices were uncovered.
**Discussion:** With appropriate training, an intervention that carries a high risk of side effects if not undertaken properly, can be administered at home by the participant, safely and effectively.

## P-24

### Defining clusters in primary care: managing blinding and contamination

Catriona Parker[1], Suzanne Hartley[1], Michael Holland[1], Bonnie Cundill[1], Sarah Alderson[2], Robbie Foy[2], Andrew Clegg[3], Amanda Farrin[1]
[1]Leeds Institute of Clinical Trials Research, University of Leeds, Leeds, United Kingdom; [2]Leeds Institute of Health Sciences, University of Leeds, Leeds, United Kingdom; [3]Academic Unit of Elderly Care and Rehabilitation, University of Leeds, Leeds/Bradford, United Kingdom
*Trials* 2019, **20(Suppl 1):**P-24

**Background:** In cluster randomised trials, clusters of participants such as practices are randomised rather than individuals, with the intervention aimed at the cluster level. Blinding can be difficult, increasing the risk of selection or performance bias or contamination between arms.
The UK primary care landscape is changing, with cross-networking at all levels from Clinical Commissioning Groups to practice federations. The level and complexity of the networks varies from linked to fully merged practices, sharing resources such as management, health professionals and specialised services.
This changing landscape has implications for cluster definition and strategies for managing blinding and contamination.
**Methods:** We will present the process of cluster selection in two primary care trials. ASPIRE is an audit and feedback trial. PROSPER is a feasibility study of an intervention aimed at supporting older people, findings will be used to inform definitive trial design and methods. General practice was defined as the unit of randomisation.
**Results:** ASPIRE defined a practice as a cluster, unless a full merger was planned. PROSPER undertook a more detailed assessment of shared resources as part of site selection. Only 5/13 practices were considered sufficiently independent to be a standalone cluster.
Reasons for grouping practices into clusters included a planned merger during trial period and sharing of practice managers. Sharing of staff and training sessions across linked practices was common.
**Discussion:** The level and complexity of networking in primary care has implications on the extent of blinding to allocation, but impact depends on the nature of the intervention. The feedback intervention in ASPIRE was less affected but PROSPER attempted to manage potential contamination by adjusting the cluster size. Methods of managing contamination in cluster trials may need to evolve with networking arrangements in primary care and be tailored to intervention characteristics.

## P-25

### A review of the use of theoretical and implementation frameworks in trials of complex interventions

Lorelei Jones, Paul Brocklehurst
University Of Bangor, Bangor, United Kingdom
*Trials* 2019, **20(Suppl 1):**P-25

**Introduction:** Understanding how and why a complex intervention works in a trial setting is crucial to determining if the results will be replicated in a specific context. A number of theoretical frameworks, such as Normalisation Process Theory, Theoretical Domains Framework, and Promoting Action on Research Implementation (PARiHS) have been developed that could potentially be used in intervention and trial design to understand the range of factors that influence adoption and impact. The aim of this study is to review the use of theoretical and implementation frameworks in trials of complex interventions.

**Methods:** 100 randomised controlled trials of complex interventions were sampled from guidelines produced by the UK National Institute for Health and Care Excellence (NICE). The full text of journal articles reporting the trial findings were retrieved and read to assess the extent to which the trial employed implementation frameworks or theory.
**Timing of potential results:** The importance of context is widely acknowledged, and both implicit and explicit theory is evident in intervention design (e.g. by incorporating education for clinicians), however so far, we have found no examples that use frameworks in the design of the trial itself. In discussing trial results authors would speculate about the influence of context, however evidence was restricted to informal feedback from participants, or reference to findings from a small number of qualitative studies in the broader literature.
**Potential relevance and impact:** Although largely absent from trial design, understanding barriers and facilitators of implementation, and using this evidence to make recommendations for practice, was a key concern of NICE guideline development groups (and they used theoretical frameworks to support this). Using theoretical frameworks in trial design could help provide important evidence on what is needed for interventions to be effective in real-world settings and support the work of translating findings into recommendations for practice.

## P-26
**Abstract withdrawn**

## P-27
### How do we optimise approach and recruitment strategies for inclusion of people with dementia in primary care clinical trials?
Rebecca Chapman, Sarah Griffiths, Lorna Manger, Ian Sherriff, Siobhan Creanor, Cath Quinn, Val Mann, Richard Byng
University of Plymouth, United Kingdom
*Trials* 2019, **20(Suppl 1):**P-27

**Introduction:** The DPACT-Dementia Support Study aims to evaluate the effectiveness of a primary care-based person-centred dementia support intervention. One of the challenges is recruiting people diagnosed with a dementia, who may also be living with frailty and emotional difficulties, and who live alone or lack an extended support network into clinical trials. Previous primary care-based trials have struggled to recruit such individuals, who may respond least well to written material, yet have the potential to benefit most from the intervention. Our aim is to test key uncertainties in relation to trial science including recruitment issues and inclusion/exclusion criteria in a Feasibility Study the learning form which will inform process for larger cluster randomised control trial.
**Methodology:** Our strategy involves testing the applicability of clinical research network staff in the identification of participants from GP registers. Our recruitment process designed to be person centred and flexible will then test the effectiveness of an Opt-In approach for potential participants in response to invitation form GP practice. A positive response initiates a pathway, with a series of exclusion points, of combined letters, phone calls and face-to-face meetings as appropriate. For those who do not respond to any opportunity along the chain, clinical follow-up will be arranged through the GP practice, to allow identification of hitherto unknown clinical or social needs. The approach follows MCA 2005 recommendations where opinion from personal or nominated consul tee would be sought should an individual with dementia lack capacity to consent.
**Timing of Potential Results:** The results from Feasibility Study will inform cluster RCT due to commence in August 2020.
**Potential Relevance & Impact:** Inclusion of clinical research network staff and optimisation of recruitment strategies has the potential to overcome practical implications of inclusion of under-served populations in primary care based clinical trials.

## P-28
### Developing quality assurance measures to use in surgical trials: an example from the ROMIO study
Natalie Blencowe[1,2], Anni Skilton[1], Rachel Brierley[2], C Paul Barham[3], Jane Blazeby[1,2]
[1]Centre for Surgical Research, Bristol, UK; [2]University Hospitals Bristol NHS Foundation Trust, Bristol, UK; [3]CTEU, Bristol, UK
*Trials* 2019, **20(Suppl 1):**P-28

**Introduction:** Randomised controlled trials (RCTs) in surgery are frequently criticised because surgeon expertise and standards of surgery are not considered or accounted for during study design. We developed and tested quality assurance (QA) measures for use within a predominantly pragmatic surgical RCT comparing minimally invasive and open techniques for oesophageal cancer (the NIHR ROMIO study).
**Methods:** Three types of QA measure were developed, and their feasibility established during the study: (i) entry criteria for surgeons, (ii) standardisation of operative techniques (by establishing key procedural phases) and (iii) monitoring of intervention fidelity during the trial, using intraoperative photography to document adherence to the key procedural phases.
**Results:** Surgeons each submitted two unedited video recordings of oesophagectomy (n=64), which were each assessed by two surgeons using the validated OSATS tool. Standardization of operative techniques was undertaken by deconstructing oesophagectomy and consideration of the components that should be delivered identically (n=4) and differently (n=2) between minimally invasive and open surgery. Monitoring of intervention fidelity was achieved by collecting digital photographs (n=1710) onto a bespoke online platform developing a tool to assess whether the six key operative phases had been delivered as intended.
**Conclusion:** The QA methods we have developed are practical to use in surgical RCTs, to investigate standards of surgery and assess fidelity to intervention protocols. Whilst the collection of large quantities of anonymised video and photo data is feasible, more work is needed to streamline the electronic assessment of videos.

## P-29
### How should we proceed from local to national evaluation of complex interventions: Overcoming challenges in scaling up a local primary care prescribing feedback intervention to a national trial – a consensus process
Sarah Alderson, Amanda Farrin, Robbie Foy
University Of Leeds, Leeds, United Kingdom
*Trials* 2019, **20(Suppl 1):**P-29

**Background:** Cumulative meta-analysis of 'Audit and Feedback' (A&F) trials have shown no improvement in effect sizes, suggesting a lack of learning on how to improve effectiveness. The Campaign to Reduce Opioid Prescribing (CROP) provided 316 practices in West Yorkshire with bimonthly evidence-based enhanced A&F reports on their opioid prescribing for one year. The CROP intervention reduced predicted opioid prescription spending by £900,000 in West Yorkshire.
We are exploring feasibility and ethical challenges of scaling up the CROP intervention for a national primary care randomised controlled trial to reduce harmful opioid prescribing whilst adding significantly to the wider evidence base on A&F.

**Methods:** A structured Delphi consensus panel of A&F experts, primary care medicines optimisation leads and members of our existing Patient and Public Involvement Panel will identify solutions to the ethical and feasibility issues of scaling up the CROP intervention, drawing upon a state-of-the-science summary of recommendations, on-going research and medicines optimisation expertise.
**Timing of potential results:** We will present the findings of the consensus process, due to take place in May and June 2019 with results due in July 2019. Results will be presented by panellist type and overall results.
Potential relevance and impact
A key factor to the successful planning and delivery of a national primary care A&F intervention trial will be how well ethical and feasibility issues relating to consent (waive consent, practice or Clinical Commissioning Group consent), unit of randomisation (practices, Clinical Commissioning Groups or regional), and the source of primary care data (nationally gathered databases or electronic health records) are resolved. We will present the consensus process recommendations on how acceptable and ideal different core ethical and feasibility options are for a national trial of different modifications to A&F for primary care opioid prescribing that will add significantly to the wider evidence base on A&F.

## P-30

### Defining follow-up of control practices in a cluster randomized trial of a complex intervention: where to start?

Sue Stirling[1], Allan Clark[1], Stanley Musgrave[1], Polly-Anna Ashford[1], Estelle Payerne[1], Jane Smith[2], Andrew Wilson[1]
[1]University Of East Anglia Medical School, Norwich, United Kingdom; [2]University of Exeter Medical School, Exeter
*Trials* 2019, **20(Suppl 1):**P-30

**Introduction:** In a randomised controlled trial consistent determination of the start of follow-up is critical. In many trials start of follow-up is often the day of randomisation. However, in a cluster randomized trial, with a complex intervention, determination of the start of follow-up may be problematic. Arrisa-UK is a two-arm multicentre cluster randomised controlled pragmatic trial of a complex intervention including practice staff training and creating an asthma at-risk register. Before randomisation, GP practices identified at-risk asthma patients, using trial-specific search criteria. Intervention practices' "at-risk" asthma patients are flagged electronically, with reminders triggered when their Electronic Health Record is opened by practice personnel. Follow-up commences on the day patient flags go "live". There is no equivalent date for control practices. Furthermore, time between randomisation and "live" flags for intervention practices ranged from 35 days to 619 days, due to delays with computerised training. Practices randomised to intervention early in the trial experienced longer delays than those randomized later.
**Methods:** We consider how to define start of follow-up for control practices. One approach is the date of randomisation. Another is to add the average number of days between randomisation and live flagging of intervention practices to the randomisation date of control practices; the resulting date used to commence follow-up. Alternatively, as time between randomisation and flagging of intervention practices decreased as the trial progressed, average delay between randomization and live flags for intervention practices for each 3-month period of randomization could be applied to control practices randomized in the same period. These approaches will be explored.
**Conclusion:** in a cluster randomized trial of a complex intervention, start of follow-up may be difficult to identify in control practices, and possible solutions should be explored.

## P-31

**Abstract omitted**

## P-32

### Central data monitoring in phase III RCTs in the UKCRC Registered Clinical Trials Units: results from a survey

Sharon B. Love, Victoria Yorke-Edwards, Sarah Lensen, Matthew R. Sydes
MRC Clinical Trials Unit at UCL, University College London, United Kingdom
*Trials* 2019, **20(Suppl 1):**P-32

**Introduction:** The FDA and EMA each published guidelines in 2013 supporting the use of risk-based monitoring approaches in clinical trials, including the wider use of central data monitoring. These ideas were enshrined in an ICH GCP Addendum in 2016. However, there is little explicit guidance or evidence for how best to achieve risk-based monitoring. We surveyed the 50 registered clinical trials units (CTUs) in the United Kingdom Clinical Research Collaboration (UKCRC), to find out about current approaches in the UK.
**Methods:** A questionnaire about monitoring in phase III randomised CTIMP trials was distributed to the UKCRC registered CTUs in November 2018. Questions focused on the relationship of central monitoring to on-site monitoring, and included why central data monitoring was used, how it was programmed, and how often run, what triggers were used, process automation, and how often triggers were assessed.
**Results:** 86% (43/50) of the CTUs responded. Five CTUs do not carry out phase III randomized CTIMPs, and another stated that the sponsor conducted the monitoring. Therefore, the monitoring experiences relate to 37 CTUs. 92% (34/37) used centrally available data to evaluate site performance, with 97% (36/37) using it to guide, target or supplement site visits. Only 6% (2/36) always used central monitoring to replace on-site visits, whilst 17% (6/36) never did. 53% (19/36) did not program the central monitoring explicitly and 56% (30/36) conduct central monitoring at least monthly. All 31 CTUs who used triggered monitoring had a process that included human assessment of whether a trigger should result in a site visit. Consent, protocol deviations, suspected fraud, number of unanswered queries, AE/SAE incidence and number of data queries were common triggers.
**Discussion:** These survey results demonstrate substantial variation in approaches to central monitoring across UKCRC registered CTUs. This finding encourages research for an evidence-based standard.

## P-33

### Risk-based trial monitoring: Site performance metrics across time

Victoria Emma Yorke-Edwards, Carlos Diaz-Montana, Konstantina Mavridou, Sarah Lensen, Matthew R. Sydes, Sharon B. Love
MRC Clinical Trials Unit at UCL, University College London, United Kingdom
*Trials* 2019, **20(Suppl 1):**P-33

**Introduction:** Trialists need to ensure data integrity and patient safety in each clinical trial. Often, the intensity of central and site monitoring strategy is chosen based on the level of risk inherent in the trial design or population. Many monitoring strategies involve selecting metrics (e.g. number of protocol violations) and assessing site performance based on these. If the metrics are poor (threshold breach for several or one significant metric) for a site, then an on-site monitoring visit is carried out. We will analyse three years of historical trial data to investigate changes in the metrics over time and how these relate to site visits that were undertaken.
**Methods:** We will look at metrics in each trial across time and investigate trends over time. In particular we will assess
a.whether there is an improvement in the metric score after a site visit
b.whether change in the metric score is sustained over the following year
c.if some metrics are more sensitive
d.interaction of the metrics
**Timing of Potential Results:** Data were received in April 2019. Results will be available in August 2019.

**Potential Relevance and Impact:** With the ICH E6 (R2) 2016 guidelines advocating risk-based monitoring, more trials are now using site performance metrics to assess if a site visit is required. Trialists need to find out more about how site performance metrics normally behave. These forthcoming data will add to our knowledge and contribute to discussions of which metrics to use.

## P-34

### Assessing the quality of data collection in clinic; lessons from the Wound Healing in Surgical Trauma (WHiST) RCT

Marta Campolier, Ruth Knight, Louise Spoors, Juul Achten, Matthew Costa
University of Oxford, Oxford, United Kingdom
*Trials* 2019, **20(Suppl 1):**P-34

**Introduction:** In 'closed' high-energy fractures associated with major trauma the surgical site infection (SSI) rate is an important outcome, as SSI involves prolonged antibiotic use, delayed rehabilitation and additional hospital admissions. To investigate the efficacy of interventions which might improve infection rates, robust methodology to assess the presence of deep SSI is required.
**Objective:** To compare wound healing complications that define a deep SSI on case report forms (CRFs) completed by patients and research nurses with information extracted from patients' medical notes.
**Methods:** This study was performed as part of the WHIST trial. Patients with a major trauma fracture requiring surgery were randomly assigned to standard or negative pressure wound dressings. The rate of deep SSI, defined by the Center for Disease Control criteria, was the primary outcome. CRFs were completed 30 days post-surgery by the participant and research nurse. An independent surgeon retrospectively reviewed a sample of participants' medical records (including all potentially infected and a random sample of non-infected cases), for pre-specified wound complications.
**Results:** 308 participants had their medical records checked. For the majority (83%), the CRFs and the routine medical record agreed on the presence or absence of a deep SSI. However, four participants with no deep SSI found on the CRFs, had a deep SSI according to their medical records. Conversely, 49 deep SSIs were indicated according to the CRFs, but not identified from the medical record. For nearly half of these latter participants (49%), at least one wound healing complication was identified in the audit. However, the remaining participants had no wound healing complications.
**Conclusions:** This study suggests that a review of the medical record alone is likely to lead to an underestimation of the rate of deep SSIs. This could have implications for the interpretation of reports of infection using routinely collected data.

## P-35

### Approaches to data cleaning in a pilot study: (EMmY) Effectiveness and acceptability of myo-inositol nutritional supplement in the prevention of gestational diabetes: a pilot placebo controlled double blind randomised trial

James Michael Heighway, Zoe Drymoussi, Doris Lanz, Shakila Thangaratinam
Barts Research Centre for Women's Health, Queen Mary University of London, Centre for Primary Care and Public Health Barts and The London School of Medicine & Dentistry Yvonne Carter Building 58 Turner Street London E1 2AB, United Kingdom
*Trials* 2019, **20(Suppl 1):**P-35

**Background:** Pilot studies provide research teams with restricted time to demonstrate that the trial would be feasible at full-scale, as well as limited time to demonstrate that high quality outcome data can be collected for this study. This demand for clean and accurate data in a short time frame requires an efficient data cleaning process. Our objective is to assess an automated data cleaning approach versus a manual method.
**Methods:** The main method used for data cleaning within the EMmY study comprised of a post-entry validation tool. Rules were created in the database to detect discrepancies of interest, including missing fields, missing forms, and incongruent fields. Queries were then raised directly through the database to study sites, for site users to address and resolve electronically.
We compared this method against the manual approach used in previous studies within our unit. In this case, sites would receive a list of data discrepancies to complete manually and return them to the research team to update in the database.
Prior to final analysis, the study statistician raises further queries. To compare the two data cleaning approaches, we will examine the proportion of queries raised by the study statistician at the end of the study, as an indicator of the level of data quality reached.
**Timing of potential results:** The EMmY study completed follow-up in March 2019, and final data analysis will take place in May 2019. Therefore, the above comparison in data cleaning approaches will be made in August 2019.
**Potential relevance and impact:** While automated discrepancy rules are more labour-intensive upfront, they yield potentially valuable time savings, whilst also maintaining the high accuracy and quality required in the short timeframe of a pilot study. Our analysis will provide insight as to whether this upfront effort is warranted over a short study duration.

## P-36

### The Validity of Setting Error Rates in Clinical Trials

Marcus Achison, Andrew N. McKenzie, Simon B. Adamson, Stephanie Haenicke, Vesselin Georgiev, Joana Rocha, Eva Lahnsteiner, Emma J. McKenzie
TCTU, Dundee University, Dundee, United Kingdom
*Trials* 2019, **20(Suppl 1):**P-36

**Introduction:** Data entry errors in clinical trial databases are inevitable due to the complexity of the information and the manual nature of the data collection process. The aim of this review was to evaluate the applicability of pre-setting acceptable error rates in clinical trials. As an indication of data quality, it is common practice to calculate error rates as part of the database audit process. Based on the error rate, decisions can be made if further data cleaning is required or if the database is ready to be locked.
**Methods:** An assessment of current literature was performed. Scientific publications, industry practices and standard operating procedures from clinical trials units were examined to determine the purpose and relevance of setting limits on error rates and how to apply them for specific clinical trials.
**Results and Discussion:** Error rates limits are influenced by such factors as industry standard, personal choice and historical precedent, with acceptable error rates ranging from 0.05% to 5%. They can also vary within a single trial, with one limit being set for critical primary outcome data and another less stringent limit for non-critical data. This review suggests that auditing and monitoring of data from early on in a trial leads to lower error rates and buys time for corrective and preventative procedures to be put in place for the remainder of the trial and for future trials. Also, in anticipation of more widespread data sharing being part of future clinical trials, publication of quality control measures such as error rates may become unavoidable.

## P-37

### Improving trial eligibility criteria: new methods to enhance fitness-for-purpose

William J Cragg[1], Kathryn McMahon[1], Jamie B Oughton[1], Rachael Selby[2,3], Rachel Sigsworth[1], Chris Taylor[1], Vicky Napp[1]
[1]Leeds Institute of Clinical Trials Research, University of Leeds, Leeds, United Kingdom; [2]University of Sheffield Medical School, Sheffield, United Kingdom; [3]Sheffield Teaching Hospitals NHS Foundation Trust, Sheffield, United Kingdom
*Trials* 2019, **20(Suppl 1):**P-37

**Introduction:** Eligibility criteria (EC) are key in defining patient populations included in a trial. There are no widely used, detailed standards for writing clear criteria in line with trial objectives and intended generalisability.
Inadequacies in criteria can lead to problems such as ineligible patients being recruited, patients being excluded from trials without strong rationale, or trial results being unintentionally limited in generalisability. Such problems are known to occur and can affect trial validity and jeopardise patient safety. Suboptimal criteria may need to be corrected in later protocol amendments, therefore contributing to research inefficiency.
In the context of trials at the Clinical Trials Research Unit (CTRU) at University of Leeds, our aims are:
1)Develop standards for writing clear EC;
2)Design quality control procedures to ensure EC are fit-for-purpose;
3)Evaluate these new methods.
**Methods:** Intervention development: using examples from CTRU trials of EC that have required amendment, we will develop a suggested formula for writing EC and a quality control tool for assessing them once drafted.
**Evaluation:** the new methods will be applied to at least two in-development trials. We will review the criteria before and after the process and qualitatively summarise the differences the new methods have made. We will also collect qualitative feedback on usefulness from those involved.
We will conduct brief surveys of Chief Investigators, investigators and other site staff to find out how they use EC and to help assess the feasibility of our new methods.
**Timing of potential results:** Late summer 2019.
**Potential relevance and impact:** Our outputs will aim to help standardise the format and raise the quality of EC used in all kinds of trials. This could reduce the number of errors relating to EC and randomisation, and ensure criteria are appropriate to the aims and intended generalisability of each trial.

## P-38

### Opportunities and experiences of accessing pharmaceutical individual patient data for statistical research

Rachel Phillips[1], Victoria Cornelius[1], Odile Sauzet[2]
[1]Imperial College London, London, United Kingdom; [2]Epidemiologie & International Public Health, Faculty of Health Sciences, Universität Bielefeld, Bielefeld, Germany
*Trials* 2019, **20(Suppl 1):**P-38

**Introduction:** We are living in the era of data sharing. In 2013, the EMA released its draft policy on publication and access to clinical-trial data including individual patient data (IPD). Although this policy is not yet mandated, in 2014 the pharmaceutical industry committed to share IPD from clinical trials in the US and EU upon request from a suitably qualified scientific/medical researcher. In addition, many funders (e.g. MRC and Wellcome) now mandate open access to IPD on completion of the primary research.
The ClinicalStudyDataRequest.com resource set-up in 2013 to enable such activity now has 19 collaborators including many major pharmaceutical companies (e.g. GSK and Roche) and funders (e.g. MRC and Wellcome). We present the key lessons learnt when applying for access to IPD via ClinicalStudyDataRequest.com and tips for a successful application.
**Methods:** In late 2018 we began the process of applying for access to IPD to conduct methodological research. In early 2019, our application was approved and access to IPD from several clinical trials was granted.
**Results:** What have we learnt?
i)How to prepare a successful application.
ii)How to identify relevant/appropriate studies (especially for methods research where the disease and/or drug are not the focus).
iii)How to prepare for receiving and storing the data.
iv)What is expected from you?
**Discussion:** IPD offers a valuable resource for statisticians and other investigators to undertake methods research and platforms such as the ClinicalStudyDataRequest.com provide access to such datasets. Access to IPD enables an opportunity to better understand novel methods when applied to real-world data in a timely manner. The process of acquiring IPD from ClinicalStudyDataRequest.com is simple and can be relatively quick but careful consideration of several key factors prior to submission can aid the process. We encourage wider use of this valuable resource from the research community.

## P-39

### Managing the paper mountain – systems and processes for tracing, managing and transferring high volume trial data from paper sources

Karolina Rusiak, Ian Woodrow
NWORTH CTU - Bangor University, Bangor, United Kingdom
*Trials* 2019, **20(Suppl 1):**P-39

Clinical data management systems and processes are an integral part of collecting, capturing, combining, cleaning, validating, and extracting high quality data for statistical analysis. This paper considers the systems and processes for the collection, capture and management of a high volume of paper source data from the IDEAL study. IDEAL was a 5-year longitudinal multicentre study, which explored the experiences of people living with dementia. Data collection was on validated TeleForm paper based case report forms (CRFs) at three time points from 38 centres. In total, 15,943 paper CRFs were received at the trials unit. The priority was to have effective and responsive processes and systems to trace, manage, and transfer high volume data from paper to electronic database. To minimise issues with large volume of data tracking and management, and to reduce the risk of human errors, unique scanable barcodes were created for each paper CRF, which were traceable on a web-based system. The returned completed CRF data were inputted into a MACRO system. Initially data were entered manually but processes and tools were then developed to allow for specific non-text data to be automatically scanned using TeleForm, converted into MACRO format using a bespoke mapping tool and then uploaded into MACRO minimising manual data entry and human errors. The bespoke mapping tool required detailed definitions and extensive validation. A separate query tracking system was used with MACRO to support data validation, cleaning and verification prior to the locking of data sets and delivery for analysis. The large volume of paper source data generated by IDEAL required closely integrated digital systems with use of established tools, bespoke conversion and tracking tools allowing the trials unit to efficiently collect and manage the large volume of data without the cost and risk of developing or procuring new combined system.

## P-40

### Data completeness and quality using an electronic versus paper Case Report Form for collecting surgical data

Hana Tabusa[1], Jessica Harris[1], Samir Bellani[1], Neil Smart[2], Natalie Blencowe[3], Charlotte Murkin[3], Barnaby Reeves[1]
[1]CTEU Bristol, BTC, University of Bristol, Bristol, United Kingdom; [2]Royal Devon and Exeter NHS Foundation Trust, Exeter, United Kingdom; [3]Population Health Sciences, Bristol Medical School, University of Bristol, Bristol, United Kingdom
*Trials* 2019, **20(Suppl 1):**P-40

**Introduction:** The CIPHER Study (investigating surgical factors for preventing parastomal hernia) developed an electronic CRF (eCRF) to collect surgical data in the operating theatre about stoma creation. An eCRF was chosen because: i) some data cannot be retrieved subsequently; ii) some data items depend on preceding answers; iii) real-time validation can be applied to minimise missing/invalid data. A paper CRF was subsequently implemented, and sites chose to use either method. We report changes in completeness, quality and timeliness of data before/after implementing the paper CRF.
**Methods:** Recruitment started in January 2018 using the eCRF, which takes 5-10 minutes to complete. Some sites reported difficulty completing the eCRF and developed informal paper CRFs. We issued a formal paper CRF in January 2019. We describe numbers/% of participants (a) for whom each CRF type was used, (b) missing <=1 data item and (c) missing <=1 data item and data entered <=1 day after operation, before/after implementing the formal paper CRF.
**Results:** BEFORE (12 months, 48 sites, 355 participants): 3 sites (6%) reported using informal paper CRFs for 31 participants (9%); for eCRFs/paper CRFs, 86% vs 97% of participants had <=1 missing data item and 69% vs 57% had <=1 missing data item with data entered <=1 day after operation. AFTER (4 months, 57 sites, 329 participants): 45 sites (79%) used paper CRFs for 223 participants (68%); 98% vs 96% of participants had <=1 missing data item and 73% vs 61% had <=1 missing data item with data entered <=1 day after operation.
**Conclusion:** About 20% of sites have successfully used the eCRF throughout and report data entry works well. However, many sites switched to paper, which is now used for most participants. Data capture is faster with the eCRF. We will compare data values to investigate the validity of each method.

## P-41

### AVATAR-AF: Getting to the heart of data management for analysis (5 simple rules to follow to ensure data integrity)

Nicholas Andrew Johnson, Thiagarajah Sasikaran
Imperial College London, United Kingdom
*Trials* 2019, **20(Suppl 1):**P-41

**Introduction:** AVATAR-AF - a three-arm, unblinded, randomised trial had results presented at the EHRA in March 2019. To ensure a high-quality dataset was available for analysis, a number of challenges had to be overcome. Upon reflection and review, 5 simple rules were subsequently developed to ensure the integrity of clinical trial data for analysis.
**Methods:** Analysis for AVATAR-AF consisted of two treatment comparisons; one between AVATAR cryo-balloon ablation and a non-invasive course of anti-arrhythmic medication, the other comparing AVATAR and conventional ablation approaches. Primary endpoint was time from initial intervention (index ablation/commencement of drug therapy) to first hospital episode related to the treatment for atrial arrhythmia. Two secondary endpoints alongside standard safety output were also defined. Statistical analysis between arms comprised of Kaplan-Meier statistics with log-rank testing and proportional hazards models. A structured data-cleaning process was formulated between the trial manager and study statistician to ensure the integrity of the analysis data whilst also allowing for a quick transition from last subject visit to database lock (8 weeks). The plan enabled primary analysis to be completed, quality-controlled and prepared for abstract submission just 17 days following database lock.
**Results:** After reviewing the processes which allowed the trial team to complete analysis within the 10-week time-frame, five key lessons were produced and presented internally within the CTU. These were based on the topics of Communication, Database Design, DMEC/DSMB Reports, Post-Hoc Analysis and Escalation.
**Discussion:** Data quality is one of the key components to a successful trial. However, with trials getting more ambitious and complex in design, considerations to ensure data quality can easily get lost during design, recruitment and follow-up. The five topics when combined and considered throughout the trial process form a solid framework that can be universally applied to ensure a high-quality analysis dataset.

## P-42

### Routine electronic health records used as participant data in UK randomised trials: The BOSS trial as a case study

Sharon Love[1], Sarah Lensen[1], Anna Kilanowski[1], Victoria Yorke-Edwards[1], Hugh Barr[2], Matthew R Sydes[1]
[1]MRC Clinical Trials Unit at UCL, London, United Kingdom;
[2]GLOUCESTERSHIRE HOSPITALS NHS FOUNDATION TRUST, Gloucester, United Kingdom
*Trials* 2019, **20(Suppl 1):**P-42

**Introduction:** There is much discussion about the current usefulness of electronic health records. In the UK, death data is nationally registered and for a fee is provided to support clinical trials. We will look at the data provided from the registry and that amassed as traditional trial data to look for patterns of data availability and to compare the correctness of data from the two sources.
**Methods:** The BOSS trial (Barrett's Oesophagus two yearly Surveillance versus no Surveillance; ISRCTN54190466) is a 3400-patient randomised controlled trial, in follow-up, designed to estimate effectiveness and cost-effectiveness1. The primary outcome of the trial is overall survival for which trial data is collected (CRF) and data has been provided from the registry annually since 2013. We will look at the death data for the whole trial across time collected by the 2 methods and describe any discrepancies e.g. maybe the registry data is available a year late or early.
**Timing of Potential Results:** Work is currently ongoing but will be completed by August 2019
**Potential Relevance and Impact:** There are many efforts and resources directed towards increasing the accessibility and quality of "big data" in healthcare. However, trialists, regulators and commissioners need to know more about the quality and timeliness of the routine data. This project will show how routine death data and trial death data compare over a 6-year period and can set a precedent for other trials presenting this, and similar, information.

## P-43

### Blinding in surgical trials: using bespoke online systems to improve blinding

Elke Gemperle Mannion, Andrew Metcalfe, Jaclyn Brown, Chockalingam Muthiah
University Of Warwick, United Kingdom
*Trials* 2019, **20(Suppl 1):**P-43

**Introduction:** START: REACTS is a participant and assessor blinded, adaptive, multi-centre randomised trial of up to 221 patients in 21 centres. The study aims to assess the clinical effectiveness of arthroscopic debridement with the insertion of an InSpace balloon to arthroscopic debridement alone. In standard care, the medical records and particularly the operation note contains all the information about the surgery, available to any healthcare professional involved in the treatment of the patient. To ensure that the participant, the assessor, and the nursing and physiotherapy teams who provide post-operative care and rehabilitation remain blinded for the duration of the study, two bespoke electronic online systems were developed to ensure the allocation the participant received remains hidden.
**Methods:** Randomisation is done online at the end of the arthroscopic debridement.
Blinded information is inserted into a secure online Operation Note system which we have created, which contains the allocation. The medical records will contain the information about the arthroscopic debridement only and contain web links to the operation note system and secure unblinding module.
Access to the participant's trial allocation through the unblinding module is granted only in case of emergency and available to any NHS personnel at any time of the day.

We will ask patients if they are aware of their allocation, after collection of the primary outcome, to see if the blinding procedure was successful in this trial.
**Findings:** Initial findings indicate that both systems are robust, user friendly and have had good acceptance by the Trusts.
**Conclusion:** Many challenges were encountered setting up a blinded surgical trial. We believe that this new approach could make blinding in surgical trials much more robust. Results from the success of the blinding will be disseminated with the main trial results.

## P-44

### Challenges in applying clinical trial standards to routine data. A case study from a randomised controlled trial embedded in a National Clinical Audit

Rebecca Walwyn[1], Suzanne Hartley[1], Robbie Foy[1], Simon Stanworth[2], Amanda Farrin[1]
[1]University Of Leeds, Leeds, United Kingdom; [2]NHS Blood & Transplant, Oxford, United Kingdom
*Trials* 2019, **20(Suppl 1):**P-44

**Introduction:** The use of routine data can enhance the efficiency of a clinical trial but presents methodological challenges. The AFFINITIE programme (Enhanced audit and feedback interventions to increase the uptake of evidence-based transfusion practice) embedded two cluster-randomised, factorial trials evaluating enhanced feedback interventions within the UK National Comparative Audit of Blood Transfusion. This audit programme aims to promote the uptake of evidence-based guidance and reduce the unnecessary use of blood components within a rolling programme of audits with bespoke data collection.
**Methods:** We used audit data, supplemented by other routinely collected data, in trial design, intervention content, outcome assessment and analysis.
**Results:** We encountered five main challenges. First, we had to link data, collected by multiple data providers for different purposes, with clusters randomised to trial interventions, where the definition of the clusters varied by data source over time. Second, as we embedded the trials in real time within ongoing audits, challenges arose in dataset version control and from the desire to include sites falling behind with data entry to maximise sample size. Third, as each audit addressed a new topic, organisational learning across audits was limited. A new database was constructed for each audit, not to clinical trials standards, compromising data quality and demanding greater data management. Fourth, the actual nature and complexity of the data available for analysis was only apparent once the data were available, after defining the primary outcome and agreeing the statistical analysis plan. Fifth, staff changes posed a particular challenge, as knowledge of the data was key.
**Discussion:** We will provide recommendations for future trials that utilise data collected for purposes other than trial evaluations.

## P-45

### Different data source, different regulatory and management demands? The use of routinely collected data in a trials unit setting

Rebecca Cannings-John[1], Michael Robling[1], Julia Sanders[2], Fiona Lugg-Widger[1]
[1]Centre For Trials Research, Cardiff University, Cardiff, United Kingdom; [2]School of Healthcare Sciences, Cardiff University, Cardiff, United Kingdom
*Trials* 2019, **20(Suppl 1):**P-45

**Introduction:** The use of routinely collected data (RCD) in a trials unit is increasing rapidly. These data can be used to identify potential participants, answer primary and secondary outcomes as well as provide longer-term follow-up of participants. The response from a regulatory perspective is however, advancing at a less rapid speed and the understanding of how RCD can satisfy regulatory requirements remains unclear.
The aim of this presentation will be to compare traditional trials which are heavily curated, and audit trailed, to studies that use RCD which are not, in terms of workflows, skill set and balance and also how they may converge/diverge in terms of regulatory requirements.
**Methods:** Using an example trial that used RCD for two of the four primary outcomes, this will be compared with another study using only RCD as part of a large observational study. Building Blocks trial accessed data from Primary Care (individual GP practices), Hospital (via NHS Digital), Immunisations (PCTs) and Abortion (Department of Health and Social Care). The POOL study is accessing data directly from maternity records at hospital sites and from the National Neonatal Research Database.
**Results:** This presentation will compare and contrast how the use of RCD in these studies have diverged from the standard operating procedures of the trials unit, the additional processes required to ensure compliance with data provider, and regulatory requirements as well as how such projects are and should be resourced. It will also compare how the different studies might influence the approach to data management and analysis.
**Discussion:** While RCD reduces burdens such recruitment, retention and follow-up, the additional work required to ensure regulatory compliance does need to be considered and resourced appropriately.

## P-46

### The feasibility of using electronic medical record data in a trial testing approaches to increasing uptake of self-management education programmes for type 2 diabetes in a multi-ethnic primary care setting

Laura J Gray[1], Danielle Bodicoat[2], Agnieszka Glab[3], Kamlesh Khunti[2], Caroline Kristunas[1], Sally Schreder[3], Melanie J Davies[2]
[1]Department of Health Sciences, University of Leicester, United Kingdom; [2]Diabetes Research Centre, University of Leicester, United Kingdom; [3]Leicester Diabetes Centre, University Hospitals of Leicester, United Kingdom
*Trials* 2019, **20(Suppl 1):**P-46

**Introduction:** Self-management education (SME) for people with type 2 diabetes mellitus (T2DM) has been shown to be effective and cost-effective. Yet uptake is low. An "Embedding Package" addressing barriers and enablers to uptake at patient, healthcare professional and organisational levels is being developed and will be evaluated in a cluster RCT. The aim of this study was to test the feasibility of using electronic medical record data in the RCT.
**Methods:** Six general practices were recruited. Pseudonymised data were extracted from primary care medical records for all eligible patients. These data were supplemented by a questionnaire completed by patients. Recruitment to the questionnaire was either by post or opportunistically by the GP. Where consent was given the questionnaire and extracted data were linked. Descriptive statistics were used to assess completeness and accuracy. Where data were linked the two sources were compared.
**Results:** Data were extracted for 2877 patients. The primary outcome for the RCT, HbA1c, had less than 10% missing data. 63% of patients had no record of ever being referred to SME but using attendance records this dropped to 46%. 423 questionnaires were received, the sample was roughly similar to the total eligible population in terms of age and sex, but not for ethnicity. More participants were recruited via the postal invite (85%) than the GP (15%). 384 (90.8%) participants consented to have their questionnaire and extracted data linked. Self-reported and extracted data had poor agreement for HbA1c with moderate agreement for SME referral and attendance data.
**Discussion:** It is feasible to extract data from primary care with reasonable completeness and accuracy for the subsequent RCT. The questionnaire data provides additional outcomes not available in primary care, such as SME preferences. Measures will be taken to further improve the quantity and quality of the data collected for the RCT.

## P-47

### Obtaining electronic routine resource use data directly from hospitals; experience from the UPSTREAM randomised controlled trial

Caoimhe Rice[1,2], Amanda L Lewis[1,2], Sian Noble[1], Marcus Drake[3,4]
[1]Population Health Sciences, Bristol Medical School, University of Bristol, Bristol, UK; [2]Bristol Randomised Trials Collaboration, Bristol Medical School, University of Bristol, Bristol, UK; [3]Translational Health Science, Bristol Medical School, University of Bristol, Bristol, UK; [4]Bristol Urological Institute, North Bristol NHS Trust, Bristol, UK
*Trials* 2019, **20(Suppl 1):**P-47

**Introduction:** Using routine patient data is encouraged by funding bodies to improve research efficiency. Routine data (e.g. Hospital Episode Statistics) are available from national sources, however it can be a lengthy process to obtain the information, which may also be incomplete.
Instead of collecting data from Case Report Forms (CRFs), which research nurses manually complete via medical note review taking considerable time, the UPSTREAM trial (ISRCTN56164274) utilised a novel method directly obtaining resource use data from 26 NHS participating hospitals in England. We estimated the informatics resource cost to hospitals of this method.
**Methods:** We requested two data downloads directly from hospital informatics teams; an interim download 12-months from trial completion and a "top-up" download upon completion of trial follow-up. We invited Information analysts to complete feedback questionnaires, which included the time taken to complete the work, from coding to data verification, and the number of datasets queried.
**Results:** All 26 hospitals successfully returned diagnostic (ICD-10), procedure (OPCS-4) and currency codes (HRGs) for inpatient stays, outpatient appointments and A&E attendances for 802 trial patients for whom we had permission to access hospital records. Feedback requests were sent by email, with two follow-up reminders. 58% of analysts responded; for these sites, the average time taken for the initial data extraction was 187 minutes (95%CI: 121–241 minutes). Number of patients per site ranged from 7–114 and number of datasets queried 2–7.
As NHS Information analysts and nurses follow the same salary structure, any time reduction using this method versus CRF completion demonstrates cost saving.
**Discussion:** We demonstrated that it is possible to obtain, directly from hospitals, a complete dataset of inpatient, outpatient and A&E resource use. This method is likely to be cost saving over nurse completed CRFs and lower the potential for missing data.

## P-48

### Use of routinely collected data from electronic health records for outcomes in NIHR HTA trials

Andrew J. McKay[1], Andrew J. Farmer[2], Carrol Gamble[1], Ashley P. Jones[1], Paula R. Williamson[1]
[1]Clinical Trials Research Centre, University of Liverpool, Liverpool, United Kingdom; [2]Nuffield Department of Primary Care Health Sciences, University of Oxford, Oxford, United Kingdom
*Trials* 2019, **20(Suppl 1):**P-48

**Introduction:** The cost of later phase clinical trials have led to an increased focus on methodology that will support innovative and efficient delivery but still require the collection of consistent and reliable data.
Clinical trials usually require the development of bespoke data collection systems yet there are many Electronic Heath Records (EHRs) available that may collect the same data. EHRs could potentially be used more widely within the delivery of clinical trials.
National Institute for Health Research Health Technology Assessment (NIHR HTA) are a major source of funding for investigator-led clinical trials within the UK. The aim of this work is to review all protocols of ongoing clinical trials funded by the NIHR HTA Programme to identify the types of EHRs currently being used for outcome data collection to identify lessons learnt.
**Methods:** We identified ongoing randomised controlled trials (RCTs) by searching the NIHR journals library. Trials collecting at least one outcome from an EHR were included. For this pilot review a random sample were assessed for inclusion with data sources and outcome types extracted.
**Results:** For the pilot, from reviewing a sample of 49/282 studies in progress (01/05/2019), 35/49 are RCTs with 20/35 (57%) using routinely collected data for at least one clinical, health economic or safety outcome. The most common EHR sources used are Hospital Episode Statistics (n=6), Office for National Statistics (n=5), and disease registries (n=5).
**Discussion:** The pilot results show around half of ongoing RCTs used routinely collected data for at least one outcome from various EHRs. For the main review, we will extract data for all eligible studies and contact their lead investigators to obtain detail from gaps in reporting. Once completed, we hope to increase awareness of potential for using EHR data and explore factors that facilitate and offer barriers to its use.

## P-49

### Using routine electronic health record data in primary care to describe population demographics and healthcare resource use: lessons from the Candem and CATCH studies to inform future clinical trial design

Michelle Collinson[1], Ellen Mason[1], Amanda Farrin[1], Laura Ashley[2], Graham Brunt[3], Alys Griffiths[4], June Hennell[5], Rachael Kelley[4], Suzanne Richards[6], Claire Surr[4]
[1]Clinical Trials Research Unit, University Of Leeds, Leeds, United Kingdom; [2]School of Social Sciences, Leeds Beckett University, Leeds, United Kingdom; [3]Leeds Beckett University Service User and Carer Group, Leeds Beckett University, Leeds, United Kingdom; [4]Centre for Dementia Research, Leeds Beckett University, Leeds, United Kingdom; [5]Centre for Dementia Research Expert by Experience group, Leeds Beckett University, Leeds, United Kingdom; [6]Academic Unit of Primary Care, Leeds Institute of Health Sciences, University of Leeds, Leeds, United Kingdom
*Trials* 2019, **20(Suppl 1):**P-49

**Introduction:** Electronic health records (EHRs), used to record most UK primary care consultations, are a potential key resource for those conducting research. Data within EHRs are not collected for research purposes, posing issues to researchers interrogating these data.
We will present challenges encountered during analysis in two projects using EHRs and discuss solutions adopted.
**Methods:** ResearchOne is a database of de-identified clinical and administrative data drawn from the EHRs of UK GP practices using the TPP SystmOne clinical system.
Funded by NIHR RfPB and Macmillan Cancer Support we conducted two cross-sectional, observational studies, using ResearchOne data to describe the population size, characteristics and healthcare resource use of people with cancer and (1) dementia and (2) long-term health conditions.
Patients aged over 50 with a cancer/dementia diagnosis consistent with Quality and Outcomes Framework eligibility between 01/01/05 and 01/01/16 were included. The data covered 391 general practices (5.1% of all English practices) across 18 datasets totalling 37,095,534 records.
**Results:** Obtaining data was challenging. We encountered difficulties and delays in accessing ResearchOne data, affecting project timescales. We will present strategies to limit similar issues in future research.
Managing data was also challenging. Lack of data dictionaries or code-lists made data cleaning difficult and time-consuming. As GPs can add codes locally, many data items required recoding into useable categories i.e. cancer type had 1946 distinct values. Missing and inconsistent data also caused issues. We will share the methodologies employed in our analysis.
**Discussion:** Research utilising routine data is often perceived as more efficient. Analysis of EHR data is, however, challenging and time-consuming. Researchers should ensure they allocate sufficient time for receipt and analysis of data. We encourage researchers working with this data to share their methodologies and suggest data providers develop comprehensive and standardised data dictionaries to allow more efficient analysis of this data.

## P-50

### Use of electronic routine screening tools to identify clinical trial participants: case of the eFI

Catriona Parker[1], Suzanne Hartley[1], Michael Holland[1], Bonnie Cundill[1], Andrew Clegg[2], Amanda Farrin[1]
[1]Leeds Institute of Clinical Trials Research, University of Leeds, Leeds, United Kingdom; [2]Academic Unit of Elderly Care and Rehabilitation, University of Leeds, Leeds/Bradford, United Kingdom
*Trials* 2019, **20(Suppl 1):**P-50

**Background:** Use of routine data in healthcare research is increasingly prevalent, due to several advantages, including availability and relatively low costs. The limitations of routine data are well described, such as time-lag from collection to publication. We report on the experience of using a routine screening tool, the electronic frailty index (eFI) to identify the target population.
**Methods:** The eFI was developed to identify categories of frailty and facilitate planning and delivery of services for older people with frailty. The eFI is automatically populated from routinely recorded data contained within primary care electronic healthcare records (EHR) and is embedded into SystmOne, EMIS, Vision and Microtest EHR systems. PROSPER is a feasibility trial in older people with frailty utilising a specific eFI score as defined by economic modelling, as an inclusion criterion. Practices were asked to search EHRs to identify eligible participants based on their eFI score and four other factors.
**Results:** Economic modelling of eFI data successfully identified an appropriate eFI cut off of ≥0.21 as the target population for the clinical trial. Using the eFI score to identify eligible participants provided some challenges. The eFI is designed for use as a screening tool and is coded into frailty categories: fit, mild, moderate and severe. The eFI report is not integrated into the patient summary record, therefore not possible to search for patients with a certain eFI score and other criteria. Additional resources were required to enable identification of participants. These findings will inform the definitive trial.
**Discussion:** Use of routine data tools for purposes other than originally designed needs early consideration to ensure any limitations can be addressed in advance. However, can be hugely valuable, as shown by using the eFI to both define the target population and identify participants.

## P-51

### Preliminary analyses of anonymised UK hospital episode statistics data to inform a research protocol on surgical management of severe pressure ulcers

Barnaby Reeves[1], Rosie Harris[1], Maria Pufulete[1], Jess Harris[1], Jo Dumville[2]
[1]Bristol Medical School, University of Bristol, Bristol, United Kingdom; [2]Division of Nursing, University of Manchester, Manchester, United Kingdom
*Trials* 2019, **20(Suppl 1):**P-51

**Introduction:** In April 2018, the UK National Institute for Health Research sought to commission an “efficient cohort study” to identify patient groups in whom surgical interventions for severe pressure ulcers (SPU) may be indicated and to determine which interventions may be suitable for evaluation of clinical effectiveness. In the absence of population data on rates of surgical intervention for SPU, we analysed anonymised hospital episode statistics acute patient care data (HES-APC) to inform a research plan. Specifically, we wished to estimate numbers of hospital admissions with a SPU and describe the characteristics of admitted patients, managed surgically or not.
**Methods:** We requested anonymised HES-APC with an ICD-10 SPU diagnosis code (L89.2, L89.3, L89.9) from 01/10/2014 to 30/09/2016, linked to HES-APC episodes for six months before and after the index admission. Index patients were identified as continuous spells (admissions), with an SPU diagnosis code in any episode during the spell. Surgical intervention was defined as reconstructive surgery (Office for Population Censuses and Surveys, OPCS, codes S17-S27).
**Results:** Of 81,383 index admissions, 40%, 14% and 46% were coded as L89.2, L89.3 and L89.9. Only 165 patients (0.2%) had reconstructive surgery during the admission; 63 had reconstructive surgery in a subsequent admission, including six who had reconstructive surgery during the index admission. Reconstructive surgery (some multiple operations) occurred in 37, 55 and 92 index admissions coded as L89.2, L89.3 and L89.9. Surgical debridement only was more frequent (OPCS code S57.1, 2013 patients, 2.4%). Patients who had reconstructive surgery were about 20 years younger and had fewer comorbidities than those who did not; about half had paraplegia, tetraplegia, spinal injuries or sequelae of transport accidents.
**Conclusion:** Our ability to identify SPU and reconstructive surgery in HES-APC led us to propose a retrospective efficient cohort study based on linked HES-APC and mortality data (NHS Digital).

## P-52

### Efficacy, feasibility and acceptability of mHealth technology (SMS) for promoting glycaemic status and self-management among low-income earning adults in Eastern Cape, South Africa

Eyitayo Omolara Owolabi, Daniel Ter Goon
University Of Fort Hare, East London, South Africa
*Trials* 2019, **20(Suppl 1):**P-52

**Background:** Diabetes persists as a significant public health and socio-economic challenge in South Africa. There is a high level of sub-optimal control, low level of knowledge and adherence to therapy, which predisposes to poor health outcomes and complications development. Adherence to lifestyle regimen, particularly dietary recommendations and activity pattern poses significant challenge to achieving targeted health outcome. The study sought to assess the impact of text-messaging on diabetes self-management and glycaemic control among low-income earning diabetic patients in Eastern Cape Province of South Africa.
**Methods:** This was a multi-centre, two-arm, parallel, randomised-controlled trial designed to compare the impact of daily text-messaging to standard care to standard care on glycaemic control and self-management. Study was conducted among diabetic patients with uncontrolled glycaemic status in selected districts of the Eastern Cape Province of South Africa. Participants were randomly assigned into the intervention and control arm. Data was collected at baseline data and six months post-intervention. Fasting blood glucose measurement followed standard procedure. Self-management was measured using a pre-validated tool. A total of 216 participants were included in the study. Mixed-model effect analysis was used to assess the impact of the SMS on blood sugar. Linear regression was used to assess for its effect on diabetes self-management. P-value of <0.05 was considered statistically significant.
**Results:** Majority were females and had Type 2 diabetes. Self-management among the study participants was low. No significant difference in the mean change in glycaemic status and self-management following a six-month intervention, mean change was 0.26mmol/L (-0.81 to 1.32), p= 0.634 for blood glucose and -0.02 (-0.15 to 0.12), p=0.821 for self-management. Text-messaging was highly acceptable and feasible among the study participants.
**Conclusion:** Text-messaging was highly acceptable and feasible among low-income earning adults. However, its efficacy on improving glycaemic control and self-management remains doubtful.

## P-53

### Abstract omitted

## P-54

### The impact of alternative assumptions on QALY calculations in the presence of missing data

Catrin O Plumpton, Dyfrig A Hughes
Bangor University, Bangor, United Kingdom
*Trials* 2019, **20(Suppl 1):**P-54

**Introduction:** Quality-adjusted life-years (QALYs) are often derived from responses to questionnaires administered to trial participants. Questionnaire data, however, are prone to being incomplete, and while multiple imputation (MI) is established as a robust approach to dealing with values missing at random, a number of assumptions are necessary to implement MI. This study aims to explore the impact of alternative assumptions on the QALY, and consequently, the incremental cost-effectiveness ratio.
**Methods:** We will simulate a data set representative of economic data alongside an RCT. Missing values will be introduced for utilities, under an MAR assumption. Each simulated patient will have either complete, partial, or no utility data. Scenarios will simulate alternative proportions of partial and complete data. For each simulated data set, combinations of the identified alternative assumptions will be modelled. Three assumptions will be considered:
1)Defining complete QALY data as requiring a minimum of two data points, including baseline, or when all utility data is complete.
2)Imputing each utility or overall QALY.
3)How timing may be represented within the imputation model, given non-exact timing in questionnaire return, and increased rigour from using patient level data, over group means at time points.
Percent bias over 1000 replications for each scenario will be reported, compared with analysis of complete data. Estimates will be compared by Monte-Carlo 95% confidence intervals. We will draw conclusions as to the most appropriate assumptions based on proportions of missing data.
**Timing of potential results:** Results from the analysis will be available for the ICTMC conference in October.
**Potential relevance and impact:** These results could offer insight and guidance as to the most robust assumptions in the imputation of missing utility data in future economic evaluations alongside clinical trials.

## P-55

### Preventing mental health conditions in children and young adults: a review of the costs of complex interventions and their components

Joanna C Thorn, Deborah Caldwell, Sarah R Davies, Jennifer C Palmer, Paola Caro, Clare French, Nicky Welton
University of Bristol, United Kingdom
*Trials* 2019, **20(Suppl 1):**P-55

**Introduction:** Mental health conditions such as anxiety or depression are increasingly widespread amongst children and adolescents, and commonly continue into adulthood; prevention has the potential to realise long-term benefits to both patients and society. Many preventative interventions have been developed, tested and reviewed; however, less is known about the costs of such programmes. This study aims to assess current knowledge of the costs and cost-effectiveness of interventions for preventing mental ill health in children and young people and to apply it to component parts of the interventions.
**Methods:** A systematic review covering MEDLINE, Embase, CENTRAL and PsycINFO was conducted to identify randomised controlled trials of school-based interventions to prevent anxiety or depression in children and young people. Included interventions were categorised as targeted or universal, and broken down into relevant components (e.g. psychoeducational, physiological). The initial search strategy was extended to the NHS Economic Evaluation Database and all results were further restricted with terms to identify costing studies (e.g. "economic evaluation", cost). A narrative review describing cost-effectiveness analyses is in preparation. Based on detailed microcosting of 'representative' interventions for combinations of intervention components, we are developing a novel method for assigning costs to the individual components.
**Timing of potential results:** Preliminary results on intervention efficacy are already available; more detailed results on efficacy by intervention components are expected by June 2019, with economic analysis results expected by September 2019.
**Potential relevance & impact:** Preliminary examination of relevant studies indicates substantial heterogeneity in terms of interventions, outcomes and economic analyses. Breaking complex interventions down into components could potentially explain some of this heterogeneity to allow more meaningful conclusions to be drawn. The results could inform the design of interventions that draw on the most effective and potentially cost-effective combinations of components, and the work will inform costing methodology in future trials.

## P-56

### Implementation of an almost paperless trial in the Emergency Setting: The SCIENCE Study (Surgery or Casts for Injuries of the Epicondyle in Children's Elbows)

Duncan Appelbe, Louise Spoors, Juul Achten, Matt Costa, Dan Perry
NDORMS, University of Oxford, Oxford, United Kingdom
*Trials* 2019, **20(Suppl 1):**P-56

The SCIENCE Study is a multi-centre prospective randomised equivalence trial of a soft bandage and immediate discharge versus current treatment with rigid immobilisation for acute torus fractures of the distal radius in children. We plan on recruiting a minimum of 334 participants from over 70 sites. The aim of this trial is to evaluate the clinical and cost-effectiveness of soft bandage immobilisation and immediate discharge, compared to rigid splint immobilisation.
Patient information for the trial is entirely electronic using online multimedia encompassing animations, videos and web-content. Data collection for the study is electronic with patient reported outcomes, consent and baseline data entered directly into the data collection system. Follow-ups are completed by the participant at home, following an automated reminder sent via E-Mail or SMS.
Management of the study is facilitated by the studies electronic systems to collect delegated duties, training, prompt for sign-offs and alerts, thereby minimising the Trial Managers workload and allowing tasks to be more easily prioritised. Communication with large numbers of sites is facilitated by a bespoke communication system, which is able to personalise e-mails (including attachments).
We present the processes, and solutions that have been implemented to make the study as efficient as possible using a generalisable approach based on the REDCap data collection system and a small amount of bespoke programming.

## P-57

### EudraCT Safety Data Input Software Tool

Simon Bond[1,2]
[1]Cambridge Clinical Trials Unit, Cambridge, United Kingdom; [2]Medical Research Council Biostatistics Unit, Cambridge, United Kingdom
*Trials* 2019, **20(Suppl 1):**P-57

**Introduction:** The remit of the European Clinical Trials Data Base (EudraCT) is to provide open access to summaries of all registered clinical trial results; thus, aiming to prevent non-reporting of negative results and provide open-access to results to inform future research. The amount of information required and the format of the results, however, imposes a large extra workload at the end of studies on clinical trials units (CTU). In particular, the adverse-event-reporting component requires entering:
•each unique combination of treatment group and safety event
•for every such event above, a further 4 pieces of information (body system, number of occurrences, number of subjects, number exposed) for non-serious events, plus an extra three pieces of data for serious adverse events (numbers of causally related events, deaths, causally related deaths).

**Methods:** A project funded by the NIHR CTU funding call "Supporting efficient / innovative delivery of NIHR research" has developed tools using standard statistical software to:
•prepare the required statistics needed by EudraCT
•format them into the precise requirements to directly upload an XML file into the web portal, with no further data entry by hand.
**Timing of Potential Results:** The project will be completed by October 2019, and we will present the tools, explain how they may be accessed and provide routes to further training.
**Potential Relevance and Impact:** The tool should remove the workload on CTUs of manually entering a large amount of data points (e.g. over 1000 datum points for a recent oncology study) using the web-interface, which is expensive and error-prone. It should also prevent the alternative and lower quality practice of uploading pdf files with safety summaries, which are difficult to amalgamate with any other data sources, nor subject to any controls regarding content.

## P-58
### Development of a web-based investigational medicinal product management (IMP) system to track and account for an IMP in clinical trials: IMP-Track

Abby Willcox, Samir Bellani, Jonathan Evans, Lucy Culliford, Chris Rogers
Clinical Trials and Evaluation Unit, Bristol Trials Centre, University Of Bristol, Bristol, United Kingdom
*Trials* 2019, **20(Suppl 1):**P-58

**Introduction:** Efficient management of a clinical trial of an investigational medicinal product (CTIMP) requires robust systems to track the IMP throughout the trial. Not all investigators and clinical trials units (CTUs) have systems available, which can make management of the IMP difficult and time-consuming. The aims of this project were to scope CTUs' requirements for an IMP management system and develop a secure web-based application to meet the requirements.
**Methods:** A survey was designed to determine the number of CTIMPs run through CTUs, IMP management systems used, and the key features required. Survey responses and a focus group with representatives from four CTUs formed the basis of the design of a web-based application, IMP-Track, which can be used to centrally manage an IMP in CTIMPs. The application was validated and systematically tested for use in a range of trial designs and a user manual was prepared.
**Results:** Twenty-seven CTUs participated in the survey; 92% were delegated the task of managing the IMP by the sponsor. Current systems included paper or spreadsheet based; in-house IT solution; IT solution provided by a third-party. Eighteen (72%) respondents were interested in a CTU-developed system. Top requirements were the ability to track batch numbers, confirm receipt of the IMP, record the return of used/unused IMP, order the IMP from the manufacturer, record temperature monitoring data and inclusion of a central monitoring function. CTEU Bristol developed IMP-Track and accompanying user manual.
**Discussion:** IMP-Track supports parallel-group, factorial and cross-over designs, includes a randomisation facility and has a full audit trail. It can manage IMP across multiple sites, communicate with external databases, send email notifications, operate in masked and unmasked modes, and unmask participant allocations. All functions are restricted by user permission settings. IMP-Track is currently in use in three multi-centre CTIMPs and is available to all CTUs.

## P-59
### Ox-PRiME - Oxford Primary trial Management Environment

David Dyson, Emma Ogburn, Ly-Mee Yu
University of Oxford, Oxford, United Kingdom
*Trials* 2019, **20(Suppl 1):**P-59

**Introduction:** In recent years, clinical trials have become more automated and complicated in terms of data collection and investigation process. Although clinical database software is becoming increasingly feature-rich to keep up with these trends, the background trial management and site monitoring can often be overlooked. Commercial trial management software is often inaccessible to developing countries and academic organisations due to restrictive pricing models.
In academic settings, trial management is often performed using standard off-the-shelf software and data files stored on secure network drives. Although this method is easy to setup and use, it is often slow to update and susceptible to mistakes throughout the lifetime of the trial.
Using off-the-shelf software can mean that collating recruitment data, safety reports and data return rates from each of the participating GP recruitment sites can become a substantial drain on the management team's time. These situations are where a centralised database would enable the management team to work far more effectively.
**Methods:** We developed a reliable and accessible web-based app, the Ox-PRiME System (Oxford Primary trial Management Environment) to manage multiple multisite trials efficiently. The system has been developed combining key aspects of trial management while keeping an adaptable database structure that can meet the specifications of many trials.
Key features of Ox-PRiME include:
* Secure user management and permissions.
* Participant management including secure remote storage of identifiable information.
* Form progress tracking.
* Recruitment site management and approval.
* Management of user training and access logging.
* Custom page layouts and database tables for each trial (editable with no system downtime).
* Interfacing with REDCap to track completed forms.
* Custom regular email reporting on stored data.

**Results:** We will demonstrate the functions of Ox-PRiME using The HABIT (Health professional Administered Brief Insomnia Therapy) trial as a case study.

## P-60
### Development of an 'eSource Storage System' for capture and storage of electronic source data for large-scale, international, randomized clinical trials

Michelle Nunn, Alex Baxter, Michael Lay
Clinical Trial Service Unit (CTSU) and MRC Population Health Research Unit, Nuffield Department of Population Health, University of Oxford, United Kingdom
*Trials* 2019, **20(Suppl 1):**P-60

**Introduction:** It is increasingly common to use computerised systems to collect clinical trial data via an electronic Case Report Form (eCRF). However, there is a need for the development of a system that can be used to fulfil the FDA & EMA guidance on the electronic collection and storage of participant source data.
**Methods:** We have developed an 'eSource Storage System' (ESS) using a publicly available 'Platform as a Service' (PaaS) solution from a trusted third-party provider.
Trial data is entered via a Web Application and saved as source data on the ESS. A copy is created simultaneously onto the in-house Trial Data System database for subsequent analysis. This approach ensures that data entry and validation are done in real-time and enforces compliance with the protocol. Participant Identifiable Data is not saved to the ESS. Security measures are in place to protect data, including HTTPS connectivity for in-transit security.
**Discussion:** Advantages of the ESS are that data can't be overwritten or deleted although updated data can be appended to an existing form. All saved data is signed off by users and timestamped by the

ESS, so every form is verifiable. A full audit trail is maintained. Authorised users e.g. Investigators & Regulatory Authorities can access site appropriate data as required during the trial and for many years after completion.
Potential drawbacks include reliance on users having a good internet connection to be able to save data.
**Conclusions:** The eSource Storage System allows the current regulatory requirements for electronic collection and storage of clinical trial source data to be met. The initial set-up process is somewhat time-consuming and there are additional project costs and complexity. There may be more efficient ways to ensure that trial results are credible, particularly in an era of expanding technology and trial designs.

## P-61
### Enabling Blinded Independent Central Review of medical images for the RAMPART trial, with a bespoke, cloud-based, Picture Archiving and Communication System

Brendan Mauger, Dominic Hague, Benjamin Smith, Carlos Diaz-Montana, Angela Meade
MRC Clinical Trials Unit at UCL, UCL Institute of Clinical Trials and Methodology, London, United Kingdom
*Trials* 2019, **20(Suppl 1):**P-61

**Introduction:** Clinical trials may require blinded independent central review (BICR) of medical images to assess or confirm outcome measures obtained from such images, especially in licensing settings. BICR is costly and may not be needed until a positive result. However, in a multi-site trial, images would preferably be collected centrally on an ongoing basis rather than retrospectively. The RAMPART trial (NCT03288532) required a cost-effective system that would allow trial sites to remotely upload and anonymise medical images to a central repository, making the trial ready to undertake a BICR, if and when required.
**Methods:** We have developed a Picture Archiving and Communication System (PACS) for RAMPART, a cloud-based solution consisting on a web application, used to upload and manage images and generate reports, and cloud storage. The system aims to provide access to medical images at crucial time points from the beginning of a trial, to archiving and future analysis. It has been designed with the capacity for future trials to use, if required.
**Results**: The first phase of the system went live in October 2018, and it is currently accessible by the RAMPART trial team, who upload images centrally, allowing further testing and fine-tuning of the remote uploading functionality. The system will be available for sites' use in the second phase release, which is currently under development. Approximately 25 scans have been uploaded to date.
**Discussion:** We have implemented a cloud-based solution that leverages the full security, cost and accessibility that is coincident with a modern cloud-based system architecture. Current development is underway to add to the existing system functionality, as well as to make it easily configurable for other trials. This will provide the opportunity for large-scale storage of medical images at a low cost, facilitating the future proofing capabilities of clinical trials.

## P-62
### Identifying factors limiting accrual and facilitating improvements: experience of using an EDC system to capture screening data

Loretta Davies, Jonathan Cook, Heidi Fletcher, Beverly Shirkey, Akiko Greshon, ACL SNNAP study team, David Beard
University Of Oxford, Oxford, United Kingdom
*Trials* 2019, **20(Suppl 1):**P-62

**Introduction:** Collection of screening data on patients considered for trial participation has been indicated as a potential means of identifying problems and facilitating improvements to recruitment. Collecting screening data, however, can be challenging and detailed data on reasons for ineligibility and non-consent often difficult to obtain. This paper describes the development and use of a bespoke online electronic data capture (EDC) system, which was used to help monitor and identify potential factors limiting accrual during the 12-month pilot phase of an NIHR HTA funded trial: ACL SNNAP.
**Methods:** The bespoke online EDC system was developed in collaboration with Fr3dom Limited. A screening case report form captured data on patients considered for trial participation and enabled documentation of specific reasons for non-inclusion (i.e. reasons patients were ineligible or declined to participate). The form was completed electronically and automatically populated an electronic screening log, enabling immediate review of the collated screening data.
**Results:** Detailed screening data was available from 15/16 sites during the pilot phase of this trial. Feedback from sites was positive with main difficulties reported to be related to occasional online access issues. The system enabled prompt review of data and collection of consistent terminology for reasons for non-inclusion. Specific factors contributing to recruitment difficulties were identified, such as, patients' strong preference for particular treatment.
**Discussion:** Implementing an EDC system to capture screening data enabled a more immediate review of the collated screening data than potentially would occur with traditional paper recruitment logs. The data permitted early identification of important factors limiting accrual and enabled the implementation of specific changes to the protocol and site training. Consideration of the use of EDC systems to capture screening data should be made in future studies to support the timely identification of factors limiting accrual, and potentially inform more effective trial conduct.

## P-63
### Making performance metrics work: Developing a triggered monitoring management system

Carlos Diaz-Montana, Lindsey Masters, Sharon B Love, Sarah Lensen, Victoria Yorke-Edwards, Matthew R Sydes
MRC Clinical Trials Unit at UCL, UCL Institute of Clinical Trials and Methodology, London, United Kingdom
*Trials* 2019, **20(Suppl 1):**P-63

**Introduction:** Risk-based monitoring (RBM) strategies are encouraged by regulators; ICH GCP E6(R2) guidelines recommend that trialists should "develop a systematic, prioritised, risk-based approach to monitoring clinical trials". The use of technology and in particular computer systems are crucial in implementing efficient, systematic RBM approaches, such as triggered monitoring, where centrally monitored triggers observing performance metrics, drive monitoring activities.
We explore the possibility of using a bespoke computer system to help the implementation of a standard, systematic and adaptable triggered monitoring strategy in the MRC Clinical Trials Unit (CTU).
**Methods:** Building on the experience gained in developing the RBM system for the TEMPER study (Diaz-Montana et al 2019), we are developing a prototype of a triggered monitoring management system which will offer trial teams the ability to program bespoke triggers, set thresholds to highlight concerning behaviour, generate and visualize data to inform monitoring actions, and collect data on findings from monitoring visits. Triggers could evolve throughout the trial by adjusting thresholds and other properties, adding triggers with different performance metrics, while triggers that do not appear to discriminate could be dropped.
**Timing of Potential Results:** A system prototype will be released and trialling of the first triggers will start before October 2019.
**Potential Relevance & Impact :** We envision this type of system will play a key role in implementing a more standard and efficient RBM strategy across the CTU; we expect it will also allow us to collect trigger usage data, identify temporal trends and patterns across triggers, and observe correlations between triggers and visit findings; enabling the refinement and development of more discriminatory triggers.
Moreover, we are designing the system to facilitate the implementation of methodological projects to identify standard triggers and best practice in triggered monitoring, by providing a technological platform where different approaches can be tested.

## P-64

### Using study websites for more than just electronic data capture: gaining trial management efficiencies

Seonaidh Cotton, Karen Campbell, Karen Innes, Mark Forrest, Graham Devereux
University Of Aberdeen, United Kingdom
*Trials* 2019, **20(Suppl 1):**P-64

**Introduction:** There are many commercial data capture solutions available for both trials management and electronic data capture, but an advantage of building bespoke software is the inclusion of added functionality. In this abstract, we describe some administrative tools and functionality that can be included in our study websites.
**Site administration:** With this functionality we register a site on the study website as soon as they express interest. The regulatory and greenlight processes required prior to opening the site are then captured within this functionality, eg REC approval for the site, progress of the site agreement (to signature), SIV training and R&D approval.
**People administration:** With this functionality we register the people working at the recruitment site and can then link them to that recruitment site. We record names, contact details, dates working on the study, date of GCP training, date of CV etc.
**Greenlight checklist:** From the information entered into the site and people administration, the greenlight checklist for the site (documenting all the agreements are in place and documents available) can be generated automatically for confirmatory signature.
**Contact lists:** The email contact details entered into the people administration are used to generate email lists for study updates; these lists can be generated by site, by role or for everyone involved in the study.
**Reporting:** From the information captured, we generate progress reports on site opening and metrics describing time between, for example expression of interest to greenlight. We can also run regular reports in relation to GCP training that might need to be repeated.
**Discussion:** This website functionality has been implemented in a large multi-centre drug study. The efficiencies gained have helped the two part time trial managers greenlight 52 sites over a seven-month period and to start the regulatory and greenlight process for a further 100 sites.

## P-65

### Predicting serious adverse events in cardiac surgery patients using real-time vital sign monitoring

Yi-Da Chiu[1,2], Sofia S Villar[2], Joanna Clarke[1], Jonathan Mackay[1]
[1]Royal Papworth Hospital, Cambridge, United Kingdom; [2]MRC Biostatistics Unit, University of Cambridge, Cambridge, United Kingdom
*Trials* 2019, **20(Suppl 1):**P-65

An early warning score (EWS) is a worldwide guide for healthcare practitioners to expedite medical decisions that help prevent the occurrence of serious adverse events (SAEs). The national early warning score (NEWS) is the EWS currently recommended by the Royal College of Physicians and has been in use for the routine clinical assessment of adults in many UK hospitals since 2012. However, this guide has not been derived for particular patient populations. In addition, its additive feature based on categorised continuous physiological parameters, may undermine its ability to predict the onset of a SAE. This reasons lead us to investigate NEWS for cardiac patients and develop a potential early warning score system.
In our work we evaluated the predictive performance of a selection of statistical and machine learning methods (such as logistic regressions, tree-based methods, neural networks) as well as NEWS. The dataset used is confidential and composed of the vital sign observations of cardiac patients admitted to Royal Papworth Hospital in Cambridgeshire from 2014 to 2017. The composite SAE we used consists of ICU re-admission, Cardiac arrest or death. The primary outcome is whether an observation captures a SAE in 24 hours before the onset. We mainly used two validation protocols for evaluating predictive performance. In addition, we adopted area under receiver operator curve, sensitivity and specificity as performance measures.
We not only prove that NEWS may be inadequate to be utilised for cardiothoracic patients, but also provide evidence of the superiority of other predictive modelling methods. In particular, multi-level logistic regression showed the greatest improvement in predictive power when compared to NEWS due to the increased level of personalisation and information included in the model.
We conclude that future methods should take patient-level information into account when making predictions.

## P-66

### The MRC HTMR recruitment working group: Driving recruitment research through collaboration

Nicola L Harman[1], Daisy Elliott[2], Carol Gamble[1], Katie Gillies[3], Helen McAneney[4], Alba Realpe[2], Leila Rooshenas[2], Gillian W Shorter[5], members of the HTMR Recruitment Working Group[6]
[1]University of Liverpool, Liverpool, United Kingdom; [2]Population Health Sciences, University Of Bristol, Bristol, United Kingdom; [3]Health Services Research Unit, University of Aberdeen, Aberdeen, United Kingdom; [4]Centre for Public Health, School of Medicine, Dentistry and Biomedical Sciences, Queen's University , Belfast, United Kingdom; [5]Institute of Mental Health Sciences, Ulster University, Ulster, United Kingdom; [6]MRC HTMR Network
*Trials* 2019, **20(Suppl 1):**P-66

**Introduction:** The Hubs for Trials Methodology Research (HTMR) were set up by the UK Medical Research Council (MRC) in 2009 to create a UK-wide resource to improve the design, conduct, analysis, interpretation, and reporting of clinical trials. Collaborations and networking were fostered through working groups focusing on key priority areas for the UK trials community.
**Methods:** The remit of the Recruitment Working Group (RWG) is to develop, implement, and evaluate recruitment interventions in clinical trials and promote/cultivate new ideas and studies to improve the efficiency of recruitment research through collaboration.
**Potential relevance and impact**: Two landmark studies in recruitment research were reported in 2017, specifically the ORRCA online resource for recruitment research (a RWG collaborative project) and the PRioRiTy study that identified the top ten questions for recruitment research. These, along with systematic review evidence, were discussed by 20 RWG members, in a face-to-face meeting, and future RWG activities prioritised.
**Three key research areas were identified by the RWG:** the core information needs of clinical trial participants (PRioRiTy question 5), core training activities for health professionals working on a clinical trial (PRioRiTy question 6), and the inclusion of under-represented/minority groups within clinical trials (PRioRiTy question 8).
Sub-working groups for each of the three research questions have been established representing cross HTMR collaboration. Research proposals are in development that will address the research questions prioritised by the RWG and to develop SWAT protocols for future evaluation across multiple trials. The RWG has enabled a collaborative, innovative, cohesive, efficient network of researchers interested in trials methodology consolidating efforts to achieve more than the sum of the parts and built a solid foundation for future growth and expansion as the network develops.

## P-67

### Embedding a National Clinical Trial into the UK Organ Donation and Transplantation Community: the PITHIA trial

Emma Laing[1], Laura A Pankhurst[1], Alison J Deary[1], Helen L Thomas[1], Dominic M Summers[2], John OO Ayorinde[2], Karla Hemming[3], Sandrine Rendel[4], Margaret Stevens[5], Victoria Bardsley[6], Desley A H Neil[7], Gavin J Pettigrew[2]
[1]NHS Blood And Transplant Clinical Trials Unit, Cambridge and Bristol, United Kingdom; [2]Department of Surgery, University of Cambridge, Cambridge, United Kingdom; [3]Department of Public Health, University of Birmingham, Birmingham, United Kingdom; [4]Nuffield Department of Surgical Sciences, University of Oxford, Oxford, United Kingdom; [5]NHS Blood and Transplant, United Kingdom; [6]Cambridge University Hospitals NHS Foundation Trust, Cambridge, United Kingdom; [7]University Hospitals Birmingham NHS Foundation Trust, Birmingham, United Kingdom
*Trials* 2019, **20(Suppl 1):**P-67

**Introduction:** Around 5,000 people are waiting for a kidney transplant in the UK. Half of UK potential deceased donors are over 60 years old, but only 28% of kidneys are eventually transplanted from this pool.
The PITHIA (Pre-Implantation Trial of Histopathology in Renal Allografts) trial is now open and will assess whether introducing a national, 24-hour digital pathology (biopsy) service increases the number, and improves outcomes, of kidney transplants from donors aged over 60 in the UK. The stepped-wedge, registry-based design is a pragmatic and cost-effective approach, but logistically, this trial was complex to implement.
**Methods:** For the trial to work, it needed to be fully embedded into many teams within NHS Blood and Transplant. This included the Organ Donation and Transplantation Hub who coordinate donor organ allocation, the 200+ Specialist Nurses in Organ Donation and the ten abdominal retrieval teams. Biopsy processing is provided by six NHS Histopathology Laboratories, which use digital slide scanners to send biopsy microscopic images remotely to one of twelve on-call Specialist Histopathologists.
**Results:** Embedding the trial required extensive relationship building, training and the development of a range of Standard Operating Procedures and logistical tools. To illustrate the complexity: the first biopsy was requested for a donor in the Isle of Wight. The Cambridge Organ Retrieval team attended the donor and performed a biopsy, which travelled with the team for processing by the Cambridge laboratory. The on-call Histopathologist in London reviewed the biopsy, with the report then relayed to the Belfast transplant team who were implanting the kidney.
**Discussion:** Collaboration across this unique community, utilisation of existing networks and the development of supportive tools has enabled this trial to be implemented on a UK-wide scale. The PITHIA trial provides a template by which future introductions of large-scale services can be assessed effectively.

## P-68

### Recruitment rates vary when recruiting outside of the UK versus within the UK: Data from the international Tranexamic acid for hyperacute primary IntraCerebral Haemorrhage (TICH-2) study

Nikola Sprigg, Lisa Woodhouse, Diane Harvard, Lee Haywood, Philip M Bath, on behalf of the TICH-2 Investigators
Stroke Trials Unit, Division of Clinical Neuroscience, University Of Nottingham, United Kingdom
*Trials* 2019, **20(Suppl 1):**P-68

**Background:** TICH-2 was an international randomised controlled trial of tranexamic acid after intracerebral haemorrhage. Here we assess whether recruitment rates were affected by recruiting country.
**Methods:** TICH-2 was performed in 12 countries (Denmark, Georgia, Hungary, Ireland, Italy, Malaysia, Poland, Spain, Sweden, Switzerland, Turkey, and the UK). Ethics and regulatory approval was achieved in each country before recruitment to the study could commence. Recruitment rates were calculated as mean number of participants recruited per site or country per month. Rates were compared between UK and non-UK sites.
**Results:** In total 149 sites were set up for recruitment, 109 UK and 40 non-UK; 15/109 UK sites (14%) and 10/40 non-UK sites (25%) failed to recruit any patients.
2,325 participants were recruited over 54 months (11 March 2013 to 30 September 2017) from 124 sites in 12 countries. 1910 (82%) were recruited within the UK (first participant 11 March 2017) and 415 (18%) were recruited outside of the UK (first non-UK participant 11 Dec 2013).
Overall the mean recruitment rate was 42.5 participants per month, the maximum monthly recruitment was 43.3 participants. For the UK, the maximum monthly rate was 35.9 participants per month, recruited at a mean rate of 34.9 participants per month (range 0.01 -2.2 participants/site/month).
In a direct comparison, there were 415 non-UK participants, recruited at a mean rate of 7.6 participants per month. For non-UK countries, the maximum monthly rate was 9 participants per month (range 0.06 – 3.28 participants/country/month). 3 countries recruited their first participants more than 36 months after the opening of the trial.
**Conclusion:**
The majority of sites recruited participants, but recruitment rates varied widely. Regulatory approval took longer in Non-UK sites. Future studies should allow longer set up time for international sites. Measures to ensure all sites enrol participants should be explored.

## P-69

### Compensation for trial related injuries: what are we waiting for?

Ayat Ahmadi, Ehsan Shamsi
Tehran University of Medical Sciences, Tehran, Iran
*Trials* 2019, **20(Suppl 1):**P-69

**Introduction:** Trial Related Injuries (TRIs) are the occurrence of injuries for participants in trials that would not have happened if they had not participated in that trial. This study mapped evidence about compensation for TRIs and analyzed the situation of Iran, as a case of developing country with a high number of RCTs. A manual for TRIs compensation was developed as well.
**Methods:** a mixed method study including a comprehensive review of related literature and a qualitative study consisting of 3FDGs and 16interview, was conducted. Quantitative data was gathered from 50 academic members of ethic committees.
**Results:** Academic-Initiated vs. industry-initiated RCTs, casual relationship between the injury and the study, study costing, and increase in the induced demand for participation in RCTs were the most frequent challenges pointed out in literature. In qualitative section, drug companies stated that insurance companies are not interested to taking part in this topic. Insurance companies were worried about how to measure the study attributes risk for an injury and who is responsible for making the final decision. Regulatory bodies were interested in ways to determine high risk studies and trial protocol considerations. Researchers were agreed on considering TRIs compensation in industry initiated studies, but they though it increases the cost of academic studies. In the quantitative part, participants mentioned to the contribution of insurance companies is the best solution for industry initiated studies(42%) but not for academic studies. There were agreement on the ethic committees as the en-charge entity to approve protocols(63%).
**Discussion:** Consideration for TRIs compensation in trial protocols brings advantages for various stakeholders. It prevents civil causes and increase authenticity and confidence around the study results. It also prevents unjustified trials. In this study we provided a guideline in 4 seasons,45 articles and 4 appendix concerning definitions, injuries, risk assessment and compensation process.

## P-70

### Establishing the International Prediction of Pregnancy Complication (IPPIC) collaborative network – Harmonising an international database

John Allotey, Shakila Thangaratinam
Queen Mary University of London, United Kingdom
*Trials* 2019, **20(Suppl 1):**P-70

**Introduction:** Pre-eclampsia, stillbirth, fetal growth restriction, and spontaneous preterm birth are all adverse pregnancy outcomes suspected to follow similar pathophysiology. Over a hundred systematic reviews have been published to date, to identify factors for predicting these outcomes and even more risk prediction models developed. We set out to establish the IPPIC Collaborative Network of global researchers to access individual participant data (IPD) from existing studies and large databases, to reach a consensus towards well developed, and externally validated prognostic models.
**Methods:** We identified primary studies, and large birth or population-based cohorts studies with information to assess the accuracy of clinical characteristics alone or in combination with biochemical and ultrasound markers for the prediction of various adverse pregnancy outcomes, through systematic review of reviews and reviews of existing prediction models. Strict range and consistency checks ensure data quality and the PROBAST Tool was used to assess dataset quality. Missing data over a pre-specified limit or inconsistencies between pre-identified variables were queried and rectified with original author input.
**Results:** The network brings together more than one hundred researchers, clinicians, and epidemiologists from over 25 countries. The collaborative group agrees the minimum data to be requested, and authors of the primary studies/datasets are contacted to share their IPD. All requested data including those not reported in the published studies are obtained. The database is set-up as a living database, so can be added to as new studies or datasets become available.
**Discussion:** Standardizing and harmonizing IPD from various sources for analysis requires committed involvement and participation by the original study team. A pre-defined harmonization process is required, with clear definitions of predictors and outcomes to aid standardization. A living database such as the IPPIC repository with >3M pregnancies provides unique opportunities to answer research questions related to the prediction of various pregnancy outcomes.

## P-71

### Identifying and learning from non-commercial trials submitted in a pivotal role in a licensing submission to the European Medicines Agency

Andrew Embleton, Matthew Sydes, Rick Kaplan, Mahesh Parmar
MRC Clinical Trials Unit at UCL, London, United Kingdom
*Trials* 2019, **20(Suppl 1):**P-71

Isolated case studies of non-commercial trials being used in marketing authorisation applications are known to experienced researchers but are rarely published in the academic literature. As a consequence, little formal knowledge exists of the experience gained from these attempts to increase the number of approved medicines available for patients.
A literature search for published case studies uncovered extremely limited results, so other sources were sought. The European Medicines Agency (EMA) publish abbreviated assessment reports online for all applications. From 1417 submissions, we identified 109 with non-commercial trial involvement. Defining major involvement as a pivotal trial which would require extensive evaluation of the trial by EMA, 25 (23%) were major uses, 42 (39%) minor and 42 (39%) were micro.
Several products were assessed together by the EMA due to their similarity, leaving 22 unique appraisals. 17 of these 22 (77%) reviewed were given a positive opinion. Of those approved, 7 were initial-evaluation applications and 10 were extensions. 9 initial-evaluation applications were submitted in total, with the 78% observed approval rate similar to the all-encompassing 2018 85% initial-evaluation approval rate. Five applications were withdrawn/refused, with two being initial-evaluation applications and the rest extensions. Four of these had major objections due to the GCP conduct of the pivotal non-commercial trial. The volume of applications in rare diseases was much higher in our subset than observed generally, arguably an illustration of non-commercial trials being conducted in neglected populations.
The use of non-commercial trials in regulatory submission is more widespread than the academic literature would suggest. Reviewing EMA applications, we highlight that, while there are clear lessons to be learnt with respect to GCP conduct, the approval rate of non-commercial trials compares well with those utilising only company-led trials. Marketing approval on the basis of a non-commercial trial is an opportunity to expand patient choice.

## P-72

### First specialized center for promoting methodology and adopting regulatory affairs in clinical trials in Iran

Ayat Ahmadi[1], Hamed Hoseini[1], Ali Akhlaghi[1], Kazem Heidari[1], Leila Janani[2], Mansoor Shamsipour[1]
[1]Tehran University of Medical Sciences, Tehran, Iran; [2]Iran University of Medical Sciences, Tehran, Iran
*Trials* 2019, **20(Suppl 1):**P-72

**Background:** Iran has been encountering a sharp growth in the number of industry and academic initiated clinical trials during recent years. It brought intense demands to meet regulatory and methodological standards. Therefore, new requirements in term of regulatory demands and ethical considerations, that could not be accomplished in university departments, have come up. In 2012, Tehran University of Medical Sciences (TUMS) with active collaboration from Iran FDO established a clinical trial center (CTC) aiming to institutionalize Good Clinical Practice (GCP) as an ethical and scientific international standard in clinical trial in Iran.
**Activities:** Iran' FDO recognizes CTC as the academic entity to train and accredit researchers based on GCP principles over the country. In doing so, number of face-to-face or online courses has been holding by CTC. It includes RCT-GCP introductory courses(n=20) , GCP advanced courses(n=18); GCP monitoring courses(n=8), short courses on special topics such as project management, biostatistics, special designs, ethical consideration(n=8), customized courses based on requests and participant eligibilities and study specific courses in order to empower clinical trial staff of a specific study(n=12).
CTC participates in performing high quality trials through consultant meetings, developing protocols and SOPs, carrying out site visits and site monitoring, statistical advocacy and promoting studies in respect to ethical and regulatory considerations. Furthermore, CTC supervises high risk academic initiated trials in TUMS. It also provides special services to initiate and perform trials in the university.
CTC initiated the first private Knowledge-based Clinical Research Organization (CRO) in Iran that works closely with Iran' FDO to enhance standards of conducting industry-initiated clinical trials by providing functional services to the pharmaceutical, biotechnology and medical device industries.
Perspective: CTC is aiming to enhance international collaboration to preserve and promote its national capacity for high quality services and to develop capacity to stabilize international collaboration.

## P-73

### Development of a resource to guide set-up and conduct of international surgical clinical trials

Julie Croft[1], Helen Howard[1], Lucy Culliford[2], Laura Magill[3], Dmitri Nepogodiev[3], Deborah Stocken[1], Vicky Napp[1], Gill Booth[1], Julia Brown[1]
[1]Clinical Trials Research Unit, Leeds Institute of Clinical Trials Research, University of Leeds, United Kingdom; [2]Clinical Trials and Evaluation Unit Bristol, Bristol Trials Centre, University of Bristol; [3]University of Birmingham
*Trials* 2019, **20(Suppl 1):**P-73

Increasingly clinical trials research needs to adapt to the changing health environment as we move to personalised health approaches, a greater awareness of the need to inform global health and to address generalisability across cultures and health service structures. This leads to the requirement for international collaboration in clinical trial conduct. International trial conduct is however more complex which can cause significant time delays, hinder efficient delivery and hence delay the potential for patient benefit.
The Clinical Trials Research Unit (CTRU) at the University of Leeds, UK, has experience of running a number of international surgical trials, all of which faced varying challenges during set-up and implementation resulting in significant delays. With these issues in mind, the CTRU successfully bid for funding from a call issued by the UK's National Institute for Clinical Research (NIHR) which focussed on supporting efficient/innovative delivery of clinical trials. The funding enabled the development of an international trials toolkit for use by UK researchers, to guide the efficient set-up and conduct of international surgical trials and therefore improve the delivery of research.
This project was led by the Leeds CTRU and brought together expertise from other UK based clinical trials units with experience of running international surgical trials. The toolkit content is based on the obstacles and successes encountered by these CTUs in coordinating and delivering international trial collaborations from the UK.
The toolkit includes suggested collaborative models for trials running internationally, case studies, links to existing resources and key considerations. Areas for considerations covers sponsorship, finance, contracts, insurance, research governance, protocol, monitoring, trial supplies, data collection, sample collection, health economics/PROMS and data ownership/publication. Each section also covers different models of working along with key issues and practical advice on how to approach the difficulties that currently hinder the delivery of international surgical trials.

## P-74

### Impact of a Collaborative Clinical Trials Methodology Course on Trainees' Funding and Clinical Trials Activity

Shirley Yang, William Meurer
University of Michigan Medical School, Ann Arbor, United States
*Trials* 2019, **20(Suppl 1):**P-74

**Introduction:** Clinical trials are challenging, and many junior investigators lack the skills to move through phases in a coherent way. To address this, the National Institute of Neurological Disorders and Stroke Clinical Trials Methodology Course (CTMC) was created through cross university collaborations to provide junior investigators with relevant training to promote well-designed clinical trials. This study aims to explore the impact of CTMC by determining the trajectory of U.S. National Institutes of Health (NIH) funding and clinical trials activity in trainees after completion of the course.
**Methods:** The online databases NIH RePORTER and ClinicalTrials.gov were searched respectively to determine NIH funding and clinical trials activity of each CTMC trainee between 2014-2017. Data was collected on the Project Start Date of the first NIH grant and the Study Start Date of the first non-withdrawn clinical trial each trainee had after taking CTMC. Grants or trials prior to CTMC were noted. The date of a trainee's CTMC was designated as January 1st of the year following course completion. Time until grant or clinical trial was calculated as the difference between the Project/Study Start Date and the trainee's CTMC date.
**Results:** There were 131 CTMC trainees between 2014-2017. As of early 2019, 23.7% of these trainees received a NIH grant and 43.5% became involved in a clinical trial after the course. Excluding those with prior experience, 11.5% of all trainees received their first-ever NIH grant and 27.5% became involved in their first-ever clinical trial following completion of CTMC. For all trainees who received a NIH grant or became involved in a clinical trial after the course, the median times to these achievements were 1.1 years (IQR 0.5-1.9 years) and 1.2 years (IQR 0.6-2.2 years), respectively.
**Discussion:** These results suggest that completing CTMC may help promote successful funding and clinical trials by junior investigators.

## P-75

### Development of a composite outcome measure for frailty prevention trials – derivation and sample size comparison with other candidate measures

Miles Witham[1], James Wason[2], Richard Dodds[1], Avan Sayer[1]
[1]AGE Research Group, NIHR Newcastle Biomedical Research Centre, Institute of Neuroscience, Faculty of Medical Sciences, Newcastle University and Newcastle Upon Tyne Hospitals Trust, Newcastle, UK; [2]Institute of Health and Society, Faculty of Medical Sciences, Newcastle University, Newcastle, UK
*Trials* 2019, **20(Suppl 1):**P-75

**Introduction:** Trials to prevent or ameliorate frailty are in their infancy. A range of different outcome measures have been proposed, but current measures require either large sample sizes, long follow-up, or do not directly measure the construct of frailty.
**Methods:** We propose a composite outcome for frailty prevention trials, comprising progression to the frail state, death, or being too unwell to continue in a trial. To determine likely event rates, we used data from the English Longitudinal Study for Ageing, collected 4 years apart. We calculated transition rates between non-frail, prefrail, frail or loss to follow up due to death or illness. We used Markov state transition models to interpolate one- and two-year transition rates and performed sample size calculations for a range of differences in transition rates using simple and composite outcomes.
**Results:** The frailty category was calculable for 4650 individuals at baseline (2226 non-frail, 1907 prefrail, 517 frail); at follow up, 1282 were non-frail, 1108 were prefrail, 318 were frail and 1936 had dropped out or were unable to complete all tests for frailty. Transition probabilities for those prefrail at baseline, measured at wave 4 were 0.176, 0.286, 0.096 and 0.442 for non-frail, prefrail, frail and dropped out. Interpolated transition probabilities were 0.159, 0.494, 0.113 and 0.234 at two years, and 0.108, 0.688, 0.087 and 0.117 at one year. Required sample sizes for a two-year outcome were between 1000 and 7200 for transition from prefrailty to frailty alone, 250 to 1600 for transition to the composite measure, and 75 to 350 using the composite measure with an ordinal logistic regression approach.
**Conclusion:** Use of a composite outcome for frailty trials offers reduced sample sizes and could ameliorate the effect of high loss to follow up inherent in such trials due to death and illness.

## P-76

### Assessment of outcomes for inflammatory bowel disease in routine clinical practice: an ethnographic study

Violeta Razanskaite, Paula Williamson, Bridget Young, Keith Bodger
University of Liverpool, Liverpool, United Kingdom
*Trials* 2019, **20(Suppl 1):**P-76

**Introduction:** There is growing interest in utilising routinely collected outcome data to support efficient, pragmatic trials. In the area of inflammatory bowel disease (IBD), there are initiatives to standardise outcomes for trials (Core Outcome Sets) and routine health records (e.g. ICHOM) . Our aims were to explore variability in outcome assessment in clinical practice and the use of two common symptom-based indices promoted for routine use by the UK Biologics Audit (Harvey-Bradshaw Index [HBI] and Simple Clinical Colitis Index [SCCAI]).
**Methods:** We performed ethnographic observations of 76 IBD clinic consultations conducted by 19 IBD clinicians (9 consultants, 7 IBD nurses and 3 trainees) in five acute hospitals in the North West region of England. Consultations were audio-recorded, transcribed and analysed for pre-defined IBD outcomes elicited by clinicians and/or volunteered by patients during patient-physician encounters, including those items required for HBI and SCCAI.
**Results:** Most commonly elicited outcomes were general wellbeing (76 [100%]), abdominal pain (61 [80%]), stool frequency (56 [74%]), blood in stool (54 [71%]), and stool consistency (46 [61%]). HBI and SCCAI were collected in only 14 (18%) consultations. In the remaining 62 consultations, items of HBI and SCCAI were discussed in variable detail. Complete HBI coverage: 5/33 (15%) consultations. Symptom components of HBI (wellbeing, liquid stools, abdominal pain): 10/33 (30%) consultations. Complete SCCAI coverage: only 1 consultation. Partial coverage (5 out of 6 SCCAI items): 5/29 consultations (17%). Selected symptoms were elicited significantly more often by nurses compared to doctors.
**Discussion:** There is significant variability in the breadth, depth and quantification of outcomes during routine clinical assessments. Although most items of clinical disease activity indices were elicited, formal scoring and assessment over fixed time periods was rare. Interviews explored barriers and facilitators to capturing structured outcomes in routine records.

## P-77

### Measuring speech development in infants: methodological considerations based on experiences within the TOPS trial

Rachael Cooper[1], Elizabeth J Conroy[1], Carrol Gamble[1], Elisabeth Willadsen[2], Christina Persson[3], William Shaw[4], on behalf of the TOPS trial management group
[1]Clinical Trials Research Centre, University of Liverpool, a member of the Liverpool Health Partners, Liverpool, United Kingdom; [2]Department of Nordic Studies and Linguistics, University of Copenhagen, Copenhagen, Denmark; [3]Institute of Neuroscience and Physiology, Speech and Language Pathology Unit, Department of Rehabilitation and Health, Sahlgrenska academy at University of Gothenburg, Göteborg, Sweden; [4]University of Manchester, School of Medical Sciences, Division of Dentistry, Manchester, United Kingdom
*Trials* 2019, **20(Suppl 1):**P-77

**Introduction:** Infants born with a cleft palate can suffer from delayed speech development when compared to non-cleft infants. Whilst speech development during early years is paramount, assessing speech both accurately and efficiently to ensure the validity and reliability of results requires careful consideration.
**Methods:** TOPS is an international two-arm randomised controlled trial aiming to determine whether it is better to perform primary cleft surgery at age six or twelve months. Infants with cleft palate were recruited from 23 centres in the United Kingdom, Scandinavia and Brazil. Speech outcomes are assessed at age twelve months, three and five years by thirty international speech and language therapists. Spontaneous speech recordings of each infant were taken at each time point for independent assessments of trial outcomes. Prior to twelve-month speech outcome assessment, extensive calibration and training was undertaken to ensure reliability of the outcomes and a good level of agreement within and between assessors.
**Results:** The trial recruited 558 infants, of which 485 (87%) had speech recorded at twelve months. Pre-outcome assessment calibration and training indicated that outcomes assessed by three independent assessors were equivalent to four. Assessors independently classified the infants as the same ability in at least 85% of infants when assessing both binary and continuous speech outcomes. Speech recordings were considered representative of day-to-day ability, with parents' assessment of ability agreeing with the independent therapists in 87% of infants. A good level of inter- and intra-rater agreement was identified for common sounds produced by the infants, although agreement varied across languages.
**Discussion:** Accurate and efficient speech outcome assessment requires careful planning. Further considerations may be required for assessments across languages. We present the steps taken to ensure valid and reliable speech assessments within the TOPS trial.

## P-78

**Abstract omitted**

## P-79

**Abstract omitted**

## P-80

### A core outcome set for seamless, standardised evaluation and reporting of outcomes throughout the surgical device innovation lifecycle

Nicholas Wilson, Rhiannon Macefield, Kerry Avery, Shelley Potter
University Of Bristol, Bristol, United Kingdom
*Trials* 2019, **20(Suppl 1):**P-80

**Introduction:** Development of new surgical procedures and devices (e.g. pacemakers, implants) is not well regulated; rigorous evaluation is uncommon and reporting unstandardised. Detailed guidance on selecting and reporting outcomes at different developmental stages is lacking. Development of generic core outcome sets (COS) with a minimum set of outcome domains to be reported at each phase of device development is one strategy to promote safe and transparent evaluation.
**Methods:** A modular COS is being developed according to COMET (Core Outcomes Measures in Effectiveness Trials) and COS-STAD (Core Outcome Set - Standards for Development) guidelines. The study has three phases:
1.Generation of an outcome long-list identified from: (i) published studies of innovative procedures and devices, (ii) NHS new procedures committee policies, (iii) regulatory documentation, (iv) qualitative interviews with surgeon-innovators. A conceptual framework for outcome domain categorisation is being iteratively developed. Outcome domains identified will be formatted into questionnaire items for a Delphi consensus survey.
2.Key stakeholders, including 50 patients and 150 professionals (surgeon-innovators, device manufacturers, regulatory representatives, journal editors), will complete the survey to rate the importance of including outcomes in a COS.
3.Stakeholder consensus meeting(s) to discuss and agree the final COS(s) and accompanying guidance.
**Results:** A long-list was generated comprising 7,302 verbatim outcomes identified from 141 published studies (40 devices) and 17 Trust policies. Outcomes were categorised into domains and formatted into questionnaire items. Following internal piloting with surgeons and academics the survey consists of 47 and 27 items for reporting guidelines and COS inclusion respectively. The survey commences in May 2019.

Conclusion: Guidelines and generic modular COS(s) for seamless, standardised, outcome evaluation throughout the surgical device life-cycle are being developed. This will promote safe and transparent evaluation of innovative devices by reducing outcome reporting bias, improving the detection of emerging signals of promising or harmful innovations

## P-81

**Abstract withdrawn**

## P-82

### Ascertainment bias: accounting for differential STI screening frequency in a HIV prevention trial

Ellen White, on behalf of the PROUD study group
MRC Clinical Trials Unit at UCL, London, United Kingdom
*Trials* 2019, **20(Suppl 1):**P-82

**Background:** Ascertainment bias is a phenomenon whereby the more screening that is conducted, the larger the number of outcomes detected. PROUD, a pragmatic trial, investigated the effectiveness of pre-exposure prophylaxis (PrEP) in preventing HIV acquisition. A key secondary outcome was sexually transmitted infection (STI) diagnoses. However, clinic attendance, and therefore STI screening frequency, was higher amongst participants receiving PrEP. We describe the impact and relevance of adjusting for the number of screens in an analysis.
**Methods:** PROUD randomised participants to receive PrEP immediately (IMM) or after a twelve-month deferral period (DEF). The outcome was defined as the total number of STIs (chlamydia, gonorrhoea, or syphilis) detected during the randomised phase. Negative binomial models were used to allow for heterogeneity between participants. Unadjusted models and models adjusting for the number of STI screens (as a linear term) were compared.
**Results:** There was a significant difference in the number of screens between IMM and DEF (mean 4.1 vs. 3.6, P<0.001). STI incidence was higher amongst the IMM group (114.7 vs. 94.3/100PY). After adjustment, the incidence rate ratio (IRR) shifted towards the null (IRR=1.2 (95% CI:1.0-1.5), P=0.08 to aIRR=1.1 (95% CI:0.9-1.4), P=0.28).
**Conclusions:** Adjusting for the number of screens has been used in several PrEP studies to account for ascertainment bias. However, it can be seen as an external confounder (e.g. additional screening driven by clinic attendance for PrEP - which we want to adjust for) or a variable that lies on the causal pathway (e.g. clinic attendance driven by symptoms, which we do not want to adjust for). Simple statistical adjustment that does not discriminate between reasons for screening is likely to over-adjust for factors related to STI risk. Therefore unadjusted analyses provide a more clinically relevant insight into the PrEP effect on STIs.

## P-83

### Objective measurement of participants' physical activity: use of accelerometry in e-coachER - a randomised controlled trial of web-based support in exercise referral schemes

Wendy Ingram[1], Lisa Price[2], Brian Wainman[1], Rod Taylor[2], Adrian Taylor[1]
[1]Peninsula Clinical Trials Unit, Plymouth, United Kingdom; [2]University of Exeter, Exeter, United Kingdom
*Trials* 2019, **20(Suppl 1):**P-83

**Introduction:** Adult patients referred to an exercise referral scheme (ERS) were recruited from primary care to the e-coachER trial (ISCRTN15644451). Participants were randomised to usual ERS +/- web-based support. The primary outcome was physical activity (PA) level at 12 months, objectively measured using a GENEActiv™ Original accelerometer. This wrist-worn accelerometer is scientifically validated for clinical trials and designed for 24-hour wear.
**Methods:** Participants (N=450) were asked to wear an accelerometer continuously over 1 week at baseline, 4 and 12 months, returning the accelerometer to CTU by post on each occasion.
On return, participants received a £20 shopping e-voucher. The accelerometer was physically cleaned, re-charged, raw data extracted, and the accelerometer returned to the pool.
PA was analysed according to pre-defined thresholds for wear-time and activity, to derive parameters of interest (e.g. wear-time ≥16 hours per day, activity in ≥10 minute bouts).
**Results:** At 12 months, 329/356 (92%) participants returned the accelerometer. Once the thresholds were applied, data were available for primary analysis for 243/356 (68%) participants. 67% of the e-vouchers issued were claimed. 165 accelerometers were required (costing c£150 each) to deliver the trial; 66 were never returned to the CTU.
**Discussion:** Learning points on use of accelerometry:
•When pre-defining thresholds for accelerometer-derived PA outcomes, potential attrition of primary outcome data and the impact on assumptions for the trial's power calculation, should be assessed.
•Unlike commercially available wearable fitness trackers, activity level is not visible or accessible to the wearer of the GeneActive™ Original accelerometer; the accelerometer is not useful to a participant beyond use in the trial. Even so, accelerometers were not returned to CTU, at considerable cost.
•A bespoke database, built and maintained by the CTU, was critical to managing accelerometer stock versus demand across multiple sites, and enabled automatic issuing of e-vouchers to participants.

## P-84

### Sample size calculation based on win ratio approach

Sirui Zheng, Duolao Wang, Tao Chen, Luis Cuivas
Liverpool School of Tropical Medicine, Liverpool, United Kingdom
*Trials* 2019, **20(Suppl 1):**P-84

**Background:** The win ratio approach has been proposed and applied in analysis of composite endpoints (e.g. cardiovascular death and heart failure hospitalisation) and continuous non-normal outcomes in clinical trials. The win ratio method has the advantages of easy-to-use for reporting composite endpoints and giving appropriate priority to the more clinically important event. However, no literature is available on sample size estimation based on the win ratio approach, which has limited its application in the design of clinical trials.
**Methods:** This study will provide a sample size estimating procedure based on the win ratio method. The statistical power of win ratio method will be compared with other statistical methods such as Cox model, Anderson-Gill model and Poisson model for dealing with the composite endpoints using some real trial datasets as well as simulated datasets. By simulations, we will generate a series of sample size tables under various scenarios.
**Timing of Potential Results:** We have three trial datasets available. The literature review, statistical analysis and simulations are underway, and the results will be ready by the end of September 2019.
**Potential Relevance & Impact:** We expect that the sample size estimation procedure and the sample size tables can be used as guidelines for making informed decisions on the sample sizes required in designing a trial with a composite endpoint as its primary endpoint.

## P-85

### A discrete choice experiment to measure public preferences for genetic testing to prevent adverse drug reactions

Danielle Johnson[1], Andrea Jorgensen[1], Dyfrig Hughes[2], Sir Munir Pirmohamed[1]
[1]University Of Liverpool, Liverpool, United Kingdom; [2]Bangor University, Bangor, United Kingdom
*Trials* 2019, **20(Suppl 1):**P-85

**Introduction:** Personalised medicine is advancing at an ever-increasing pace, with the possibility of routine genetic testing to prevent adverse drug reactions looking more and more likely. It is essential that the views of the public are included in this advancement. We are designing an experiment to measure the general public's opinions about genetic testing to prevent adverse drug reactions. This will take the form of a discrete choice experiment (DCE), a survey design which allows us to quantify preferences.

**Methods:** A DCE requires participants to 'trade-off' different aspects of genetic testing. For example, we may find that people are willing to wait an extra month to receive results if the accuracy of the test was higher. Using this design allows us to provide quantitative answers to the question of public preferences.

To ensure the generalisability, relevance, and accuracy of results, qualitative work to inform DCE development is essential. We are planning to use online questionnaires with a patient group and with clinicians, followed up with focus groups with the general public. We are hoping to use a market research company to administer the survey, as they have access to a UK-representative panel and can return results within 48 hours.

**Timing of Potential Results:** At the time of abstract submission, we are awaiting ethical approval to begin the study. Once this is received, we anticipate spending 3 months on qualitative work, and 2 months for data analysis.

**Potential relevance and impact:** We are doing this to ensure that the views of the general public are heard and can inform future developments in personalised medicine. Quantifying using a DCE provides clear results to policy-makers and clinicians. This increases the likelihood of public acceptance of personalised medicine interventions.

## P-86

### Public involvement beyond clinical research studies

Laura Flight[1], Susan Baxter[1], Samaira Khan[1,2]
[1]School of Health and Related Research, University of Sheffield, United Kingdom; [2]NIHR Research Design Service, Yorkshire and the Humbe
*Trials* 2019, **20(Suppl 1):**P-86

**Background:** Public involvement as advisors in health research studies is becoming an established norm. However, scrutiny of the literature indicates that public involvement is predominantly described in clinical evaluation studies, such as trials of medical interventions. The value and role of public involvement in studies which are theoretical or methodological, such as the development of new statistical methods, are less well reported. We aimed to explore the value of having public advisors on these types of studies where, in contrast to clinical studies, their input is not drawing on patient experience.

**Methods:** A qualitative study, using focus groups with members of the public and with researchers, was used to explore the perceived role and value of public advisors in types of health research which are not clinical research studies. Focus groups were recorded, and transcribed, and qualitative data analysis software was used to systematically store and retrieve data during thematic analysis.

**Results:** Fifteen public and nine researcher participants took part in the study. Examination of the data suggested themes relating to potential benefits from public involvement; challenges to involvement; and opportunities provided. The data indicated potential for public involvement at different stages of the research cycle in all studies, including those which are more theoretical or methodological in nature, such as methods development in clinical trials.

**Discussion:** Involving the public as advisors in all forms of research adds value, and the study confirms that involvement should not be confined to research evaluating clinical interventions. The study provides information for health researchers in areas where public involvement may be less established, such as methods development in clinical trials. Involvement in these research areas has the potential to add diverse forms of knowledge, provide legitimacy, and aid impact.

## P-87

### Co-designing a virtual world with young service users to deliver social cognition therapy

Doctor Alba Realpe[1], Farah Elahi[2], Doctor Sandra Bucci[3,6], Max Birchwood[2], Ivo Vlaev[4], David Taylor[5], Doctor Andrew Thompson[2,7]
[1]Population Health Sciences, University Of Bristol, Bristol, United Kingdom; [2]Mental Health and Wellbeing Division, Warwick Medical School, University of Warwick, Coventry, United Kingdom; [3]Greater Manchester Mental Health NHS, Foundation Trust, Manchester, United Kingdom; [4]Behavioural Science Group, Warwick Business School, University of Warwick, Coventry, United Kingdom; [5]Department of Surgery & Cancer, Faculty of Medicine, Imperial College London, London, United Kingdom; [6]Division of Psychology and Mental Health, University of Manchester, Manchester, United Kingdom; [7]Centre for Youth Mental Health, University of Melbourne, Melbourne, Australia
*Trials* 2019, **20(Suppl 1):**P-87

**Introduction:** Involving service users in the design and conduct of research has been encouraged in government policy, but it is rarely achieved, especially at trial initial stages. Co-design implies genuine partnership in the generation of knowledge between service users and researchers. This paper shows a step-by-step co-design approach used to adapt an existing manualised social cognition intervention for people with a first episode of psychosis to a virtual world environment.

**Methods:** Clinical researchers, IT programmers and a group of young people who have used mental health services were invited to participate in the design of a virtual environment to deliver an accessible social cognition intervention to a hard to engage service user group. An iterative process between service users and the design team was set up and included developing initial ideas, creating a prototype and testing the virtual world.

**Results:** Twenty young service users of local mental healthcare services participated in the design and planning of intervention delivery. Young people felt the virtual environment should be familiar, urban spaces, akin to therapy rooms or classrooms they have used in real-life situations rather than non-traditional therapy spaces that were initially proposed. Findings reflected the demographic makeup of the sample.

**Discussion:** After the co-design process, the specific design, approach and protocol was tested in a proof-of-concept trial with young people who experienced a first episode of psychosis. Young service users were integral to an agile and iterative design. Technological innovations should be routinely co-designed and coproduced if they are to realise their potential to deliver acceptable and affordable mental health interventions.

## P-88

### The benefit of a Lived Experience Advisory Panel (LEAP) in the design and conduct of a clinical trial into depression

Yvette Walters[1], Niamh Quann[1], Eleanor Taylor[1], John Gledhill[2], Peter Bates[2], Rachel Hobson[1], Richard Morriss[2]
[1]University Of Leicester Clinical Trials Unit, Leicester, United Kingdom; [2]University of Nottingham, Nottingham, United Kingdom
*Trials* 2019, **20(Suppl 1):**P-88

**Introduction/Aims:** This trial is investigating Transcranial Magnetic Stimulation (TMS) as a treatment for patients with moderate to severe treatment resistant depression. From the outset the research team were keen to involve service users or carers with lived experience of managing depression, in order to optimise engagement with, and retention of participants.

**Methods:** Throughout the trial set up and current phase of recruitment we have imbedded LEAP in all activities by: -scheduling ongoing quarterly (LEAP) meetings; involving members who either have received TMS treatment, participated in previous TMS trials, or have experience of treatment resistance depression, all local to the research sites; including LEAP members in reviewing trial documents; completing a practice run-through of trial assessments; inviting LEAP members to sit on the interview panel for recruitment of trial staff.
**Findings:** LEAP feedback was invaluable in the development of this trial and enabled the research team to make key changes, e.g. avoiding medical jargon within participant facing trial documents, specifically the information sheet, consent form and advertising materials. LEAP members also advised on visit structure, suggested changes to outcome measures, and highlighted the importance of consistency in patient-staff relationships. Following this advice, the treatment period was extended to allow participants more flexibility for attendance. Additionally, LEAP members' inclusion in the interview process ensured that empathetic trial staff were appointed.
**Conclusion:** By involving patients with lived experience from the outset, several changes were made to the trial design to improve patient experience. The outcome has been promising thus far and we will continue these strategies, involving LEAP members in all dissemination activities. Using this approach throughout the trial has the potential to improve the experience of participants, and therefore has implications for participant retention in future clinical trials into depression.

## P-89

### Uptake of interventions to communicate results of a phase III randomised controlled trial to participants: early results from the Show RESPECT study (ISRCTN96189403)

Annabelle South[1], Cara Purvis[1], Ania Spurdens[1], Elizabeth James[1], Carlos Diaz-Montana[1], Matthew R. Sydes[1], William J. Cragg[1,2], Katie Gillies[3], Sierra Santana[1], Nalinie Joharatnam-Hogan[1], Archie MacNair[1], Andrew J. Copas[1]
[1]MRC Clinical Trials Unit at UCL, London, United Kingdom; [2]Clinical Trials Research Unit, Leeds Institute of Clinical Trials Research, University of Leeds, Leeds, United Kingdom; [3]Health Services Research Unit, University of Aberdeen, Aberdeen, United Kingdom
*Trials* 2019, **20(Suppl 1):**P-89

**Introduction:** Previous studies show that participants want to be informed of the overall results of trials they have taken part in, but that this often does not happen. Anecdotal reports suggest even when trials do attempt to communicate results to participants, it is often not done well. Show RESPECT tests three approaches, identified and developed through extensive PPI, to communicating trial results to UK participants of ICON8, a phase III ovarian cancer chemotherapy RCT.
**Methods:** Show RESPECT is a mixed methods cluster randomised factorial Study Within A Trial (SWAT), run within ICON8. Over 40 ICON8 UK centres (hospitals) are being randomised to communicate results to participants using combinations of:
-Link to a basic webpage (text only) vs enhanced webpage (including diagrams, video, links to further support and information, and the option to submit questions)
-Printed summary vs no printed summary
-Invitation to join an email list vs no invitation
The primary outcome measure (OM) of Show RESPECT is participant satisfaction with how the results are communicated. Data are collected from participants, site staff and CTU staff by questionnaires, and qualitative interviews are being carried out with site staff and participants.
Secondary OMs include uptake of the interventions, which is measured using analytic data from customised links (for webpages and email list) and data collected on logs by site staff (printed summaries).
**Timing of potential results:** The final sites will be randomised in May 2019. We will present data on the PPI process and intervention uptake (available from August). Results from the primary outcome will be presented at a later date.
**Potential relevance and impact:** These data have the potential to inform future approaches to better communicate overall results to similar participant populations in other trials, helping trialists meet their ethical obligations to offer results to patients in accessible ways.

## P-90

### MAMMO-50 - Working with the patients to enable multiple types of data collection within a clinical trial

Maria Ramirez[1], Andrea Marshall[1], John Lindley[1], Asha Bhandari[1], Annie Young[1], Maggie Wilcox[2], Sophie Gasson[1], Peter Donnelly[3], Andy Evans[4], Janet Dunn[1]
[1]Warwick Clinical Trials Unit, Coventry, United Kingdom; [2]ICPV, London, United Kingdom; [3]South Devon Healthcare NHS Foundation Trust, Torquay, United Kingdom; [4]Ninewells Medical School, Dundee, United Kingdom
*Trials* 2019, **20(Suppl 1):**P-90

**Introduction:** There is a lack of evidence/consensus amongst surgeons on optimum frequency or duration of follow-up including mammography for breast cancer patients aged 50 years and older at diagnosis. Mammo-50 RCT has the opportunity to gather patient reported outcomes and patients' perspectives on follow-up.
**Methods:** Mammo-50 trial has recruited over 5235 women in a randomised trial assessing duration of mammographic surveillance for women over 50 years old at diagnosis and 3 years post curative surgery. 91% of women agreed to participate in a quality of life substudy (QoL) and 75% of women consented to enter the Qualitative sub-study (QSS). In addition, a national patient-led survey on follow-up was developed by the Independent Cancer Patients' Voice (ICPV) to gather patients' experience of follow-up.
**Results:** Mammo-50 patient questionnaires indicated that 28% of patients had high levels of distress due to concerns about fatigue, sleep, worry/anxiety, memory/concentration, hot flushes and pain. Mammo-50 focus groups (6 at multiple sites) and individual interviews (32 telephone interviews) indicated that patients in general were satisfied with their care and happy to be in a trial. The interviews reached saturation quickly with patients being concerned about early discharge from hospital follow-up and the fear of recurrence. The ICPV survey indicated that over 2/3rds of respondents said they had some unmet needs during their follow up period; these were varied and included both physical and psychological needs.
**Discussion:** In summary, focus groups and individual interviews suggest that patients, when followed-up by the trial team, are happy with their care. When given the opportunity to report unmet needs through questionnaires/surveys, patients often report things which could be causing them distress, but which may go unnoticed in routine follow-up. Clinical trials have the opportunity to collect patient reported experiences alongside the standard QoL booklets, providing a rich source of additional data.

## P-91

### Abstract omitted

## P-92

### PIRRIST: Patient and public involvement (PPI) to enhance recruitment and retention in surgical trials

Joanna C. Crocker[1,2], Jennifer Bostock[3], Shaun Treweek[4], Nicola Farrar[5], Alan Chant[3], Jonathan A. Cook[6], Polly Kerr[2], Sian Rees[7], Louise Locock[1,4], Kerry Woolfall[8], Sophie Olszowski[9], Richard Bulbulia[10,11]
[1]Health Experiences Research Group, Nuffield Department of Primary Care Health Sciences, University of Oxford, Oxford, United Kingdom; [2]National Institute for Health Research (NIHR) Oxford Biomedical Research Centre, Oxford, United Kingdom; [3]Patient/Lay Partner, United Kingdom; [4]Health Services Research Unit, University of Aberdeen, Aberdeen, United Kingdom; [5]Population Health Sciences, University of Bristol, Bristol, United Kingdom; [6]Surgical Intervention Trials Unit, University of Oxford, Oxford, United Kingdom; [7]Oxford Academic Health Science Network, Oxford, United Kingdom; [8]Institute of Population Health and Society, University of Liverpool, Liverpool, United Kingdom; [9]SPZ Associates Ltd., United Kingdom; [10]Clinical Trial Service Unit, Nuffield Department of Population Health, University of Oxford, Oxford, United Kingdom; [11]Cheltenham General Hospital, Gloucestershire Hospitals NHS Foundation Trust, Cheltenham, United Kingdom
*Trials* 2019, **20(Suppl 1):**P-92

**Introduction:** Poor recruitment and retention are common challenges to the successful delivery of surgical trials. Patient and public involvement (PPI) has the potential to improve both but there have been few attempts to formally investigate this. We aimed to develop an evidence-based PPI Intervention to enhance Recruitment and Retention In Surgical Trials ('PIRRIST').
**Methods:** Four stages: (1) Online survey to identify current PPI practice in UK surgical trials; (2) Stakeholder focus groups and interviews to explore PPI needs and challenges, issues with participant recruitment and retention, and how PPI might address these; (3) Two online surveys to estimate the frequency and importance of the identified issues with recruitment, retention and PPI in surgical trials; (4) Stakeholder workshop to determine key features of the final PPI intervention.
**Results:** 393 individuals took part across four stages of data collection. Based on the findings, we made recommendations for PPI in surgical trial design including: use a two-tier model of PPI (ongoing partnership with individuals plus consultation with the wider patient population); involve patients/carers with personal experience of the target health condition; budget for staff time on PPI. We held eight events with surgical trial staff and patient/public contributors across the UK to review these recommendations and plan next steps. From this we developed succinct, practical guidance to aid Chief Investigators (CIs) in planning PPI in surgical trials. We involved a professional graphic designer, user-tested the guidance with CIs and consulted many stakeholders including PPI contributors and trial managers.
**Discussion:** Our evidence-based guidance will help CIs to plan PPI that should boost participant recruitment and retention (evaluation needed). Although based on evidence gathered from surgical trials, the guidance could be applied to other clinical trials within the UK. The guidance will be available online from June 2019 via www.phc.ox.ac.uk/pirrist

## P-93

### Patient and Public Involvement & Engagement in UKCRC Registered Clinical Trials Units – A Scoping Exercise

Heather Bagley[1], Steven Blackburn[2], Claire L Vale[3], Delia Muir[4], Cally Rick[5], John Cleland[6], Kym Thorne[7], Laura Mader[8], Daniel Beever[9], Adwoa Parker[10]
[1]Liverpool Trials Collaborative, University of Liverpool, Liverpool, United Kingdom; [2]Research Institute for Primary Care and Health Sciences, Keele University, Keele, United Kingdom; [3] MRC Clinical Trials Unit at Univerisity College London, London, United Kingdom; [4]Leeds Clinical Trials Research Unit, University of Leeds, Leeds, United Kingdom; [5]Nottingham Clinical Trials Unit, University of Nottingham, Nottingham, United Kingdom; [6]Glasgow Clinical Trials Unit, University of Glasgow, Glasgow, United Kingdom; [7]Swansea Clinical Trials Unit, Swansea University, Swansea, United Kingdom; [8]Patient Led Research Hub, University of Cambrdige, Cambridge, United Kingdom; [9]Sheffield Clinical Trials Research Unit, University of Sheffield, Sheffield, United Kingdom; [10]University of York, York, United Kingdom
*Trials* 2019, **20(Suppl 1):**P-93

**Introduction:** The UK National Institute for Health Research 'Breaking Boundaries' review of patient and public involvement (PPI) in research highlighted the need for a more strategic approach to PPI, recommending that PPI leads should have opportunities to network and share best practice. Furthermore, there is increasing emphasis on public engagement within clinical trials, including public dialogue about the need for and design of trials.
The UK Clinical Research Collaborative (UKCRC) Registered Clinical Trials Unit (CTU) Network established a Patient and Public Involvement and Engagement (PPI&E) Task and Finish Group to conduct a scoping exercise to:
•Identify different approaches for delivering PPI&E within CTUs
•Map existing PPI&E resources during the course of clinical trials
•Develop more effective collaborative PPI&E working across registered CTUs.
**Methods:** We conducted an e-survey with registered CTUs to investigate the current PPI&E landscape and challenges. Responses were discussed at a workshop with targeted activities to explore PPI&E practices. 4 public contributors also attended the workshop, sharing their perspectives on how CTUs should involve and engage patients and the public.
**Results:**
•46/51 registered CTUs completed the survey, 39 attended the workshop. Findings included:
•15/46 CTUs reported one person with overall PPI &E responsibility
•11 CTUs had PPI guidance for trial staff, 2 CTUs had PE guidance. 6 CTUs used PPI standard operating procedures
•No CTUs stated they would be unwilling to collaborate in PPI&E activity.
**Discussion:** This scoping exercise illustrated that PPI&E is evolving, with involvement currently more advanced than engagement. Resources to support PPI&E varied amongst CTUs. There is duplication of activities, suggesting a need for a formal sharing mechanism across the Network. The UKCRC PPI&E group will be developing a national communication strategy and central repository for resources and training for PPI&E to address the findings of this scoping exercise.

## P-94

### Research nurse and patients perspective on innovative early phase trial designs

Aimee Jackson, Ceri Armstrong, Faye Lowe, Christina Yap
Universtiy Of Birmingham, Birmingham, United Kingdom
*Trials* 2019, **20(Suppl 1):**P-94

**Introduction:** In recent years there has been a significant increase in the use of novel trial designs increasing statistical efficiency, particularly in the early phase setting. Little work has been done to assess how these designs impact patients and research nurses. We set out to develop an understanding of their views on novel early phase designs including Continual Reassessment Method, exploring if trial designs can be more patient-centred.
**Methods:** We conducted a joint interview with two research nurses working in early phase oncology trials (RN) and held a group discussion with nine representatives from a Patient and Public Involvement group (PPIgroup) to identify the aspects of trial design that mattered most. An interactive session was held at the NIHR Statistics - Early phase trials meeting, February 2019 (EPmeeting).
**Results:** 78% (7/9) in the PPIgroup and 48% (20/42) of EPmeeting felt that increased trial design complexity would have no impact on patient recruitment. Members of PPIgroup felt that an increase in logistical complexity would discourage trial recruitment but that trial design complexity would not. 39/44 delegates at the EPmeeting felt that patients would feel less secure joining a first –in-man trial however RN reported no concerns with recruiting to these trials; 1 member of the PPIgroup commented "We were clutching at straws and would have done anything". When asked about dose selection in a dose-finding trial, EPmeeting gave mixed responses across dose ranges however; the PPIgroup felt that patients would be reluctant to receive the highest dose due to potential toxicities.
**Discussion:** Trialists understanding of patients' needs may not necessary be in line with patients'. In order to ensure that innovative designs achieve benefits that matter most to patients, they need to be designed with patients as co-developers. Further work is required to identify how to encourage a more patient-centred approach.

## P-95

### Using Stakeholder Focus Groups in a Qualitative Study of the Acceptability, Feasibility and Design of a Randomised Trial – PARCS Feasibility Study

Cushla Cooper[1], Joanna Crocker[2], Naomi Merrit[1], Molly Glaze[1], Jonathan Cook[1]
[1]SITU, NDORMS, University of Oxford, Oxford, United Kingdom; [2]Dept of Primary Care, University of Oxford, Oxford, United Kingdom
*Trials* 2019, **20(Suppl 1):**P-95

Focus groups are widely used in health services research. They enable participants to speak freely about concerns and offer views on existing and proposed evaluation of new approaches to surgical treatment. A qualitative study was conducted using focus groups to elicit the views of stakeholders on the acceptability and feasibility of a randomised trial to evaluate the effectiveness of patches in augmenting rotator cuff repair. Stakeholders' opinions on various trial design options were also sought. This was part of a larger, multi-method project (the PARCS feasibility study) to assess the overall, acceptability and design of a trial of this surgical innovation.
Participants were recruited to separate focus groups reflecting each of the key stakeholder groups: patients/carers; industry; research regulatory representatives. The focus groups were facilitated by a trained member of the PARCS study team. Discussions were audio recorded and transcribed verbatim. Transcripts were analysed by two members of the PARCS team, alongside data collection and using thematic analysis. Themes from discussions were grouped according to whether they related to: patient population to include; the components of intervention and control arms; the outcomes to measure; and practical considerations in trial set-up.
A total of 24 people participated in the focus groups. There was widespread stakeholder support for patch use in rotator cuff surgery. There was discrepancy among some stakeholders about whether a trial was even needed. They had varied perspectives on timing of patch use and also what patches would be compared to in a trial setting (e.g. patch vs no patch, or patch A vs patch B).
The focus groups allowed us to elicit and understand views of a broad range of stakeholders. The findings were used to inform the subsequent stages of the PARCS study including a survey of surgeon trialists, a Delphi process and final consensus meeting.

## P-96

### What methods are used to involve patients and the public in numerical aspects of research? A scoping review

Beatriz Goulao, Marion Campbell, Katie Gillies, Craig Ramsay
Health Services Research Unit, University of Aberdeen, Aberdeen, United Kingdom
*Trials* 2019, **20(Suppl 1):**P-96

**Introduction:** Patient and public involvement (PPI) in clinical trials may improve research, but the best way to achieve its full potential is unclear. One challenge is the lack of methodology to involve patients and the public in numerical aspects of trials (ie design, data collection, analysis and interpretation). We aimed to review methods to involve the public or elicit their opinions about numerical aspects of research and draw lessons applicable to PPI in trials.
**Methods:** We conducted a scoping review using EMBASE (1946-April 2019) focused on stakeholder involvement and participatory research. We included primary research and case studies. The public included citizens or research end-users (ie farmers in veterinary medicine or patients in healthcare).
**Results:** 97 abstracts were identified, 38 full texts assessed, and 19 papers included. Studies were published between 2006 and 2018 and focused on environmental research (n=7), veterinary (n=6), policy (n=3), health research (n=2) and trials (n=1). Ten studies involved the public in the analysis; six in data collection; three in research design and one in the interpretation of findings. The main methods used were participatory modelling (n=9), participatory epidemiology (n=6), participatory mapping (n=1), questionnaire (n=1), focus group (n=1), discrete choice experiments (n=1). Nine studies involved multiple stakeholders (like researchers, politicians and the public). The studies discussed two main reasons to elicit the public's opinions on numerical aspects: to make research more relevant to stakeholders, increasing their sense of ownership; to gather information from stakeholders, when relevant data is unavailable.
**Discussion:** There are methods available to incorporate the public's opinions on numerical aspects of research. These methods have been successfully implemented in other fields and have the potential to improve current PPI practices in trials, but their acceptability and feasibility needs to be investigated.

## P-97

### Public Involvement in a study to improve patient empowerment in musculoskeletal care using an online health portal: The MSK-Tracker

Steven Blackburn[1], Katie Tempest[1], Kay Stevenson[1], Krysia Dziedzic[1], Stephanie Tooth[2], Jonathan Hill[1]
[1]Research Institute for Primary Care and Health Sciences, Keele University, Keele, United Kingdom; [2]Keele Clinical Trials Unit, Keele University, Keele, United Kingdom
*Trials* 2019, **20(Suppl 1):**P-97

**Introduction:** New musculoskeletal (MSK) care models are required that are patient-centred and better reflect patients' individual needs. The Arthritis Research UK Musculoskeletal Health Questionnaire (MSK-HQ) is a recently validated outcome measure to evaluate an individual's MSK health-status across the care pathway. The aim of this paper is to describe how people with MSK conditions have been involved in a study to test the feasibility and impact of implementing the MSK-HQ using an innovative IT platform, the MSK-Tracker.
**Methods:** As part of the study team and in advisory groups, people with MSK conditions contributed to the co-design and delivery of the study, including:
•a stakeholder workshop to determine the MSK-Tracker's requirements within a clinical setting
•study design, including recruitment methods and outcome measures
•co-design and informal testing of the MSK-Tracker platform
•development of participant information
•project management
•interpretation of results
**Results:** Public contributors actively shaped this project at every stage. For example, by identifying the MSK-Tracker's potential to give patients a "start-point for the consultation" by providing "an opportunity to discuss the things important to you". They also suggested having a secondary outcome to examine how the nature of the consultation conversation changed with the platform's introduction. Through co-design and informal testing, public contributors have been instrumental in improving the MSK-Tracker's usability and functions. Public contributors have actively supported the troubleshooting of difficult recruitment issues and are helping to explore the wider value of the MSK-Tracker to patients in the future.
Some challenges existed with public co-applicant involvement in project management meetings and sustaining regular feedback with other public contributors.
**Discussion:** People with MSK conditions made important contributions throughout this study. Future activities will focus on ways to share findings with patients and the wider public and how other studies can similarly embrace public involvement.

## P-98

### A framework for embedding meaningful Patient and Public Involvement in PROMS development

Steven Blackburn, Annette Bishop, Jonathan Hill, Krysia Dziedzic
Research Institute for Primary Care and Health Sciences, Keele University, Keele, United Kingdom
*Trials* 2019, **20(Suppl 1):**P-98

**Introduction:** Public Involvement (PI) is key to ensuring the relevance, acceptability, and quality of patient-reported outcome measures (PROMs) in clinical trials. Guidance exists on PROMs development which includes the use of qualitative research with patients to establish the content and face validity of PROMs. However, there is no specific guidance for PI roles throughout PROMS development. This paper proposes a new framework for embedding meaningful PI in this process. Working collaboratively with the research team, PI can have important roles alongside the research activities throughout all stages of PROMs development, as follows:
Scoping-
Research: i) Literature review to identify existing PROMS and relevant outcomes, ii) Expert opinion
PI: i) Review the quality and acceptability of existing PROMs; ii) Identify the need for a new PROM; iii) Advise on research plan (e.g. recruitment, interview topic guide)
Conceptual Framework & draft PROM-
Research: Qualitative interviews with patients to identify outcomes of importance
PI: i) Conduct interviews; ii) Analyse and interpret findings; iii) Develop conceptual framework; iv) Draft PROM content
Iterative development-
Research: Cognitive interviews to verify face and content validity of the draft PROM
PI: i) Analyse and interpret findings; ii) Finalise PROM wording and format; iii) Support translation and cultural adaptation for use in other countries
Assessment of psychometric properties-
Research: Observational or experimental study
PI: Contribute to the interpretation of the psychometric properties from and patient and public perspective (e.g. missing data issues, minimal clinical important difference)
Dissemination and implementation-
Research: Publish
PI: i) Support dissemination to the general public; ii) Encourage uptake and use of the PROM in clinical practice
**Conclusion:** A framework for embedding meaningful PI throughout the PROMS development process is proposed. Previous studies have implemented individual elements of this framework. Future work will test all elements of the framework together to assess added value and impact.

## P-99

### Raising the Standards of Public Involvement in Clinical Trials

Steven Blackburn[1], Carol Rhodes[1], Adele Higginbottom[1], Laura Campbell[1], Research User Group[1], Nadine Foster[2], Krysia Dziedzic[1]
[1]Research Institute for Primary Care and Health Sciences, Keele University, Keele, United Kingdom; [2]Keele Clinical Trials Unit, Keele University, Keele, United Kingdom
*Trials* 2019, **20(Suppl 1):**P-99

**Introduction:** The UK National Standards for Public Involvement in research have recently been launched to improve the quality of public involvement. Keele University is one of the ten test sites nationally developing ways to put the six National Standards into practice. This paper presents some of the approaches and resources developed to implement the National Standards for use with clinical trials.
**Methods:** In partnership with Keele Research User Group (RUG), we used the Standards to audit current trial processes and co-produce new resources to improve public involvement practices. Over 12 months, we adopted a Plan-Deliver-Review-Act approach to implement each Standard across the Research Institute and Clinical Trials Unit.
**Results:** The audit highlighted areas for improvement in how we deliver public involvement. We have developed new resources and practices for the six Standards, including:
Standard 1 (Inclusive Opportunities): A Diversity and Inclusion policy and new recruitment plan to ensure fair opportunities for public involvement
Standard 2 (Working Together): Clear role descriptions for all public involvement roles (Trial Steering Committees, Public Co-applicants, Advisory Groups)
Standard 3 (Support and Learning): Induction sessions for new public contributors and a 'RUG-Buddy' peer-mentoring scheme with more experienced members supporting new members
Standard 4 (Communications): a brief guide for researchers to encourage clear, two-way communication with public contributors, including improved feedback
Standard 5 (Impact): Tips on capturing and evaluating the impact of public involvement in trials
Standard 6 (Governance): A developing Public Involvement and Engagement Strategy, and Funding formula to ensure appropriate public involvement budgets.
**Discussion:** We have used the National Standards to reflect on current practices and develop new resources to improve public involvement in research. They are helping to drive a culture change towards doing better public involvement. More is to be done on encouraging wide-spread awareness and adoption of the Standards across studies.

## P-100

### Linking involvement and engagement in clinical trials research: a year in the life of a public involvement partnership

Katie Banister, Katie Gillies, Craig Ramsay, Members of the HSRU Public Involvement Partnership
Health Services Research Unit, University of Aberdeen, Aberdeen, United Kingdom
*Trials* 2019, **20(Suppl 1):**P-100

**Introduction:** We have focused on maximising public engagement, to drive better and more meaningful involvement in our research and worked with public partners to develop engagement activities about clinical trials. This presentation will highlight key learning points of setting up and running a public involvement partnership within a health services research unit and clinical trials unit (CTU) to link involvement and engagement.
**Methods:** We set up an independent Public Involvement Partnership, led by a dedicated PPIE coordinator (KB). Individuals within the group were recruited through a number of mechanisms, with involvement in projects varying across activities. Regular opportunities for feedback from the group contribute to a cycle of ongoing improvement, with opportunities for reflection at the end of the first year.
**Results:** A total of eight local public partners were recruited to the group. Over the year, the group's activities were based on improving the accessibility of information about trials, shaping trial questions and methods, and dissemination of trial results to the public.
The group valued face-to-face meetings and being part of a team. They enjoyed the opportunity to visit the CTU and see researchers in their day-to-day jobs. A bonus of the face-to-face format was the sharing of ideas and perspectives which was harder to facilitate by individual emails.
At the end of year review, the group were extremely positive about the group itself, the research they contributed to, and their enthusiasm for continuing. They are proud of what they have achieved.
**Discussion:** By linking involvement and engagement, we have promoted reciprocal sharing of ideas to improve the way we involve the public meaningfully in our research.

## P-101

### Improving the Induction of PPI Contributors on Trial Oversight Committees

Emily Pickering[1,2], Conor Tweed[3], Bec Hanley[3]
[1]University College London, London, United Kingdom; [2]Imperial College London, London, United Kingdom; [3]Medical Research Council Clinical Trials Unit at University College London, London, United Kingdom
*Trials* 2019, **20(Suppl 1):**P-101

**Introduction:** Clinical Trials Units are encouraged to integrate Patient and Public Involvement (PPI) into all aspects of trial design, running and oversight. This research explored the induction and training of PPI Contributors joining trial oversight committees and was used to update the Medical Research Council Clinical Trials Unit at University College London's (MRC CTU at UCL) induction pack for new PPI Contributors.
**Methods:** Published and unpublished materials on training for PPI Contributors on oversight committees were reviewed, with themes then triangulated to identify the most common topics covered in induction training. A face-to-face workshop with PPI Contributors from the MRC CTU at UCL reviewed a draft updated Induction Pack. Findings from these discussions were incorporated into a revised induction pack which was then re-reviewed by the workshop attendees.
**Results:** No published literature on this subject was found. However, several common themes were identified from unpublished materials. Workshop attendees agreed with most of the themes suggested in the initial draft pack based on the literature search and also provided a number of additional topics for discussion.
**Discussion:** There is very little consistency in the induction of PPI Contributors on oversight committees. Whilst most local guidance explains the general role of a PPI Contributor, more context and background of the particular trial needs to be provided to allow for adequate induction of new committee members. The Induction Pack created provides a framework upon which trial managers can build a full picture of their study.

## P-102

### Hard to reach or easy to ignore - the use of social media and community engagement in ROSHNI2

Farah Lunat[1], Nusrat Husain[2], Peter Bower[2], Nadeem Gire[1], Nafeesa Bhatti[1], Anharul Islam[1], Maariya Lorgat[1], Afsana Tutla[1], Wajid Malik[3]
[1]Lancashire Care NHS Foundation Trust, United Kingdom; [2]University of Manchester, United Kingdom; [3]Stafford NHS Trust, United Kingdom
*Trials* 2019, **20(Suppl 1):**P-102

**Background:** There is under-representation of ethnic minorities in clinical research. British South Asian women are considered 'hard to engage' due to multiple factors including language, culture and stigma. Social media and community engagement have the potential to play a pivotal role in bridging this gap in research participation.
**Design:** ROSHNI-2 means light in Urdu/Hindi language is a Randomised controlled trial (RCT) to compare culturally adapted Positive Health Programme (PHP) based on the principles of CBT with Treatment as Usual (TAU) in BSA women with postnatal depression. The study aims to recruit 720 mothers.
**Community engagement:**
•Social media: We have used well-known platforms such as Facebook, Twitter, Instagram to disseminate information. We have connected with ethnic channels and with popular social media influencers to raise awareness about maternal health.
•Chai with ROSHNI2: Inviting GPs & Health Visitors to meet the women and encourage conversation around mental health.
•There a many languages spoken throughout South Asia, the main ones being; Urdu, Hindi, Punjabi, Gujarati, Bengali and Tamil. Each researcher is able to speak English and one of the study languages fluently. Participants are able to communicate in the language that they feel most comfortable in and all study materials have been translated.
**Results:** In comparison to recruitment rates prior to the social media campaign, increased activity on our pages resulted in much higher rate of recruitment.
Feb-March 2019: 6400 post reached (575% increase from previous months) 2000 post enjoyments (168% up from previous months)
April-May 2019: 13,600 post reach (213% increase) 2063 engagements (7% increase)
**Conclusions:** The ethnic minorities are considered "hard to reach" though the feedback from the community tells us that they think they may be "easy to ignore". This may be because there is a lack of innovative and culturally sensitive recruitment strategies in place.

## P-103

### Considerations for statistical analysis when QALYs are a co-primary endpoint in a large phase II/III randomised controlled trial

Kara-Louise Royle[1], Adam Martin[2], David Meads[2], Walter Gregory[1], Lucy McParland[1]
[1]Clinical Trials Research Unit, Leeds Institute of Clinical Trials, University of Leeds, Leeds, United Kingdom; [2]Academic Unit of Health Economics, Leeds Institute of Health Sciences, University of Leeds, Leeds, United Kingdom
*Trials* 2019, **20(Suppl 1):**P-103

**Introduction:** STAR is a phase II/III, UK multicentre, randomised controlled, non-inferiority trial evaluating the use of treatment breaks compared with continuous treatment for patients with renal cancer. Quality adjusted life-years (QALYs) are a co-primary endpoint of STAR with overall survival. They are calculated using the utility index derived from the patient reported EQ-5D-3L questionnaire, which is collected during trial treatment and follow-up, encompassing both time-on and time-off trial treatment.
Missing EQ-5D-3L data occurs in STAR. We will describe and justify how this limitation has impacted on how QALYs will be derived and analysed.
**Methods:** Missing EQ-5D-3L data during the follow-up period will be imputed using multiple imputation by chained equations at the utility level. QALYs for each participant will be derived within each imputed dataset.
QALYs are hypothesized to display a bimodal distribution thus mixture models, with two normal components, will be fitted within each imputed dataset, regressing QALYs on randomisation allocation and the minimization factors of the trial. A marginal treatment effect will be obtained, and the results combined using Rubin's rules.
**Results:** The trial will conclude non-inferiority in terms of QALYs, if the lower bound of the 95% confidence interval around the combined marginal treatment effect corresponds to a ≤10% difference in the mean QALYs between the two arms.
**Discussion:** The statistical analysis planned for QALYs within STAR is the result collaboration between statisticians and health economists. This considered: the level of imputation (question or utility); whether the missing data pattern would be consistent over time; which periods to impute (on or off trial treatment, or both); the form and type of imputation model; how to account for withdrawal and death; how to model the assumed bimodal distribution of the QALYs and finally how to assess the decision related to the primary research question of non-inferiority.

## P-104

### Measuring patient reported outcomes in the POSNOC trial: strategies employed to promote high quality data return

Kathryn Monson[1], Dame Lesley Fallowfield[1], Valerie Jenkins[1], Wei Tan[2], Shirley May[1], Clare Brittain[2], Shabina Khan[2], Amit Goyal[3]
[1]SHORE-C, Brighton & Sussex Medical School, University of Sussex, Brighton, United Kingdom; [2]Nottingham Clinical Trials Unit, University of Nottingham, Nottingham, United Kingdom; [3]Department of Surgery, Royal Derby Hospital, Derby, United Kingdom
*Trials* 2019, **20(Suppl 1):**P-104

**Introduction:** POSNOC is a multi-centre RCT to determine whether axillary clearance/radiotherapy can be avoided safely in women with early breast cancer and one or two involved sentinel nodes. It is centrally coordinated at Nottingham CTU (NCTU) with patient reported outcomes (PROs) on quality of life and long-term side effects of axillary treatment managed by Sussex Health Outcomes Research & Education in Cancer (SHORE-C).
**Methods:** PRO measures are FACT B+4, EQ-5D-5L, STAI Y1/Y2, LBCQ, and QuickDash questionnaires. All are completed in clinic at baseline, then by post at 3, 6, 12, 24 & 36 months apart from LBCQ and QuickDash which are administered by site staff in clinic or by telephone. Site staff are trained in PRO procedures at trial initiation. Further training is provided at individual site level and via regular trial memos/newsletters. SHORE-C, NCTU and site staff liaise closely to ensure participant health status and contact details are kept up to date and as many PROs as possible are completed by patients. PRO data are monitored in real time to permit swift resolution of any problems.
**Results:** 1172 UK patients were randomised to 28/02/2019; seven did not commence PRO measures and a further 81 (7.5%) discontinued postal questionnaires. Key reasons by 12 months for stopping were patient decision and withdrawal from the trial. At 24 and 36 months questionnaires were discontinued if not returned at 3 previous timepoints. Thirteen patients had died. Postal (clinic) questionnaire returns, calculated as a percentage of the number expected at each timepoint were: 98% (97%), 92% (-), 93% (90%), 92% (90%), 87% (86%), and 90% (88%) respectively.
**Discussion:** A recent review on strategies to reduce missing PRO data concluded this can be minimised by implementing thoughtful design and methodology strategies. POSNOC has employed several of these strategies and achieved high PRO data return.

## P-105

### How often should outcomes be measured in eczema clinical trials?

Beth Stuart[1], Laura Howells[2], Joanne Chalmers[2], Kim Thomas[2]
[1]University of Southampton, United Kingdom; [2]University of Nottingham, United Kingdom
*Trials* 2019, **20(Suppl 1):**P-105

**Introduction:** Eczema has an episodic nature and in clinical trials, repeated measures are often used to give more precise estimates of between-group differences. The Harmonising Outcome Measures in Eczema (HOME) collaboration has recommended that outcomes be measured at least at baseline and follow-up but, where repeated measures planned for a trial, it is unclear how many measures would be optimal.
From a statistical standpoint, more measures are better because additional measures reduce intra-patient variation and can decrease the required sample size. But for researchers and trial participants, fewer measures minimises burden.
This study explores whether there is an optimal number of measurements beyond which there are few statistical gains.
**Methods:** The statistical efficiency can be calculated from the correlation within and between repeated measures. We used the correlation matrices from 4 published clinical trials to explore the marginal gains in statistical power from including additional repeated measures. All trials used the Patient Orientated Eczema Measure (POEM), the HOME recommended instrument for the symptoms domain, weekly over a 12-16 week period.
**Results:** Four or five measures seem to be optimal. On average, the sample size required with 4 follow-up measure was 64% of that required for a single follow-up measure, 61% for 5 measures and 60% for 6 measures. Few marginal gains in sample size were made beyond 5 measures.
There is no statistical requirement for the measurements to be evenly spaced but, if they are, this equates to approximately monthly measurements for a 12-week trial.
**Discussion:** For eczema trials, measuring outcomes around 4-5 times is likely to be most efficient, which is consistent with results for other conditions. This represents the statistical view and will be shared with the HOME collaboration.
However further discussion is needed to incorporate the views of patients, clinicians and researchers before recommendations can be made.

## P-106

### Abstract omitted

## P-107

### Do study participants complete electronic questionnaires?

Lucy A Culliford[1], Eleanor Gidman[1], Emma Hopkins[1], Rachel Maishman[1], Graziella Mazza[1], Terrie Walker-Smith[1], Jane M Blazeby[2], Chris A Rogers[1]
[1]CTEU Bristol, BTC, Translational Health Sciences, Bristol Medical School, United Kingdom; [2]Bristol Centre for Surgical Research, Population Health Sciences, Bristol Medical School, United Kingdom
*Trials* 2019, **20(Suppl 1):**P-107

**Introduction:**
Electronic capture of patient reported outcomes (PROs) is increasingly utilised in clinical research. Resources are required to develop and maintain electronic PRO tools, but this is offset with savings in printing and postage costs and data entry of paper questionnaires. Personal computers and smart phones have made the internet more accessible. However, if participants prefer paper questionnaires, or if data quality is compromised with electronic data capture, resources may not be optimally used. We present data from two large studies offering postal and online questionnaires to assess participant preferences and data completeness.
**Methods:** The studies: OMACS – Outcome Monitoring After Cardiac Surgery (ISRCTN90204321), a cohort study in cardiac surgery, collecting Quality of Life (QoL) data for 1y and By-Band-Sleeve (BBS; ISRCTN00786323) – a multicentre RCT of bariatric surgery collecting QoL data at multiple timepoints over 3y.
**Results:** OMACS: 642 participants, 12% of whom opted for online completion of the PRO. Compared to the "paper completion" group, the "online completion" group contained proportionally fewer females (26% vs 23%) and were on average 3 years younger (median age 69 vs 63y). Completion rates were lower in the "online completion" group (3 months 97% vs 93%; 12 months 86% vs 63%)
**BBS:** 1296 participants, 34% of whom opted for online completion of the PRO. Again, the "online completion" group contained fewer females (81% vs 69%) and were younger (median 52 vs 49y). Overall, 280 of ~5700 (~5%) questionnaires were completed online, with the remainder completed by post or in clinic.
**Discussion:** Uptake of the electronic PRO completion option is low and completion rates were lower than when paper was used. Many BBS participants who opted for online data capture have in practice completed questionnaires by post or in clinic. Reasons for these choices need investigation.
Both studies were funded by the NIHR.

## P-108

### Variation and poor reporting on the measurement of patient-reported outcomes can hinder the interpretation of study findings: a case study using the WOMAC measure

Bethan Copsey[1], Jacqueline Y Thompson[1,2], Karan Vadher[1,3], Usama Ali[1], Susan J Dutton[1], Ray Fitzpatrick[1], Sarah E Lamb[1], Jonathan A Cook[1]
[1]University of Oxford, United Kingdom; [2]University of Birmingham, United Kingdom; [3]Eli Lilly, United Kingdom
*Trials* 2019, **20(Suppl 1):**P-108

**Introduction:** A lack of consistency in how outcome measurement tools are used and reported in clinical research can hinder its interpretation and limit its usefulness. We explored this issue using a case study of the WOMAC, a patient-reported outcome commonly used in osteoarthritis research. The WOMAC is made up of multiple items across 3 subscales: pain, function and stiffness. A number of important variations in how the items can be measured and combined have been proposed. It is unclear what current practice is and whether these variations are used in practice. This study aims to review how the WOMAC tool is being used in a specific clinical area.
**Methods:** A cohort of randomised trials in hip or knee osteoarthritis that used the WOMAC measure were reviewed. Data were extracted on the version used, how it was implemented, use of subscales and total score, and how scores were analysed and reported.

**Results:** 62 randomised trials reported the WOMAC. The version used was unclear for one-third of studies (35%, n=22/62), although half used a Likert scale version (50%, n=31/62). The total (66%, n=41/62), pain or function subscales (63%, n=39/62) were reported in most trials. The stiffness subscale was reported less often (48%, n=30/62). Of trials that reported the WOMAC total score, only one-third reported the results for all three individual subscales (pain, function and stiffness) (37%, n=15/41). The range of the total score was unclear for 20% of trials (n=8/41) and, where reported, spanned from 0-10 to 0-2400.
**Discussion:** The measurement and calculation of the WOMAC score were highly variable. Furthermore, the reporting of the WOMAC methods and results was often poor. This hinders the interpretation of study findings. Clear, consistent and complete reporting of the WOMAC measurement methods is needed.

## P-109

### Considerations in implementing an electronic patient reported outcome (ePRO) system

Rebecca Lewis, Elizabeth Hill, Claire Snowdon, Emma Hall, Judith Bliss
The Institute of Cancer Research, London, United Kingdom
*Trials* 2019, **20(Suppl 1):**P-109

**Introduction:** Participants of trials managed by the Clinical Trials and Statistics Unit at The Institute of Cancer Research (ICR-CTSU) currently complete patient reported outcome (PRO) questionnaires on paper. We are now considering implementing electronic capture of PROs.
We summarise here our requirements for an ePRO system, together with plans for implementation and impact assessment.
**Methods:** System requirements:
•Secure access and data storage
•User friendly interface and password management
•Two step identity verification
•Scheduling and reminder facility
•Basic compliance reporting
•Statistical analysis data output facility
•Compliance with applicable regulations
**Implementation plans:** Following identification of a potential system, patient and public focus groups will assess user acceptability. Should this be successful, the system will be piloted within an appropriate trial. A study within a trial (SWAT) will be implemented with the aim of comparing paper and electronic questionnaire responses to investigate whether questionnaire modality impacts PRO data. Response rates to both modalities will also be compared. Participants will also complete user feedback surveys to capture qualitative data on their experience using the system. A statistical analysis plan will be developed prior to initiation of the pilot.
**Challenges:** ePRO system use has resulted in non-compliance findings during several MHRA inspections, related to lack of validation, poor audit trail and lack of source data. Regulators are currently developing further guidance on ePRO which will need to be taken into account if implementing ePRO in a trial which falls under the MHRA. Any system implementation requires comprehensive pre-implementation testing and user acceptability prior to rollout, necessitating dedicated resource investment.
**Discussion:** Anticipated benefits
We hope that the introduction of an ePRO system will improve participant experience over completing questionnaires on paper and may therefore improve response rates. The planned SWAT will aim to identify whether this is the case.

## P-110

### A hospital-integrated symptom monitoring system for patients after discharge following surgery: a pilot study in cancer-related surgery

Rhiannon Macefield[1], Hollie S. Richards[1], Kerry N. L. Avery[1], Amanda Portal[1], Ruth Harding[2], Trudy Reed[2], Katy A. Chalmers[1], Rob Carter[3], Kate Absolom[3], Rishi Singhal[4], Galina Velikova[3], Jane M. Blazeby[1]
[1]Bristol Centre for Surgical Research, Bristol Medical School: Population Health Studies, University of Bristol, Bristol, United Kingdom; [2]University Hospitals Bristol NHS Foundation Trust, Division of Surgery., Bristol, United Kingdom; [3]Patient Reported Outcomes Group, Leeds Institute of Cancer Studies and Pathology, University of Leeds, Leeds, United Kingdom; [4]Queen Elizabeth Hospital Birmingham, University Hospitals Birmingham NHS Foundation Trust, Birmingham, United Kingdom
*Trials* 2019, **20(Suppl 1):**P-110

**Background:** Cancer-related major abdominal surgery is associated with lengthy recovery and complications. Concerning symptoms can go undetected as clinical follow-up after hospital discharge is not standardised. Electronic patient-reported outcomes (ePRO) systems can enhance symptom monitoring and detection of complications and improve patient outcomes. Evidence for the use of ePRO systems in surgical oncology is lacking. This prospective pilot study evaluated the feasibility of a hospital-integrated electronic symptom monitoring system for patients after discharge following cancer-related major abdominal surgery.
**Methods:** The ePRO surgery system is an online questionnaire integrated into hospital electronic records that provides patients with tailored self-management advice or automated alerts to a clinician depending on symptom severity. Participants recruited from University Hospitals Bristol and University Hospitals Birmingham completed the ePRO questionnaire weekly post-discharge. Aims included examining barriers to using the ePRO system, questionnaire completeness, symptom severity-dependent actions generated by the system and technical performance. Interviews with participants and clinicians explored perceptions of the usefulness and acceptability of the ePRO system.
**Results:** Thirty-one participants were recruited to the study and questionnaire response rates typically exceeded 60% (range 50-100%). Of 197 ePRO completions analysed, most triggered self-management advice (39%) or advice to contact a clinician (37%), and 4% triggered email alerts to clinicians. Participants reported that they found the ePRO system reassuring and relevant to symptom management during recovery. Clinicians described the system's usefulness for understanding patients' experiences of recovery and monitoring symptoms in the context of their ongoing recovery.
**Discussion:** The use of the ePRO system to monitor patients during recovery after cancer-related surgery is feasible and acceptable to patients and clinicians. A future randomised controlled trial will evaluate the effectiveness of the ePRO system for improving the detection of symptoms, complications and patient outcomes after hospital discharge following major cancer-related surgery.

## P-111

### Chronic Headache Education and Self-management Study (CHESS) – Smartphone Application

Chloe Norman, Shilpa Patel, Martin Underwood
University Of Warwick, Coventry, United Kingdom
*Trials* 2019, **20(Suppl 1):**P-111

**Introduction:** The Chronic Headache Education and Self-management Study (CHESS) is a multi-centred randomised controlled trial comparing an education and self-management program with a relaxation control arm. We required frequent data collection for headache outcomes, and therefore aimed to develop and pilot a system for this using a smartphone app.
**Methods:** The app was developed by Clinvivo Ltd, a University of Warwick spin–out company, who worked closely with the CHESS team. Using the existing literature, the team's clinical expertise and input from our lay advisory group three questions were developed to capture headache frequency, duration and severity.
Once eligibility was confirmed, participants were asked to complete the smartphone app (or a paper alternative for those without a smartphone) and given detailed instructions on how to install and use. The app is completed weekly for six months then monthly for six months. For each question a calendar is displayed indicating the recall period of seven days, and participants receive app notifications when responses are due. Participants who never downloaded the app or haven't completed the app for at least three weeks are sent reminders via post or email. At 12 months, participants are provided with a summary of their responses should they wish to receive this.
**Results:** During feasibility, eight participants downloaded the app and completed for up to 11 weeks. Completion rates varied, participants did not report any difficulties downloading or using the app.
In our ongoing RCT the average completion rates for all app users is currently 62%, with 67% of participants completing at least 3 responses between randomisation and the 4 month questionnaire.
**Discussion:** The app data is still being completed by participants. We will have final results at the end of 2020.

## P-112

**Patient reported experiences collected in the PERSEPHONE Herceptin duration trial**

Janet A Dunn[1], Maggie Wilcox[2], Sophie Gasson[1], Louise Hiller[1], Claire Hulme[4], Peter Hall, Bethany Shinkins[4], Helena Earl[3]
[1]University Of Warwick, Coventry, United Kingdom; [2]Independent Cancer Patient Voice, London, United Kingdom; [3]University of Cambridge, Cambridge, United Kingdom; [4]University of Leeds, Leeds, United Kingdom; [5]University of Edinburgh, Edinburgh, United Kingdom
*Trials* 2019, **20(Suppl 1):**P-112

**Introduction:** PERSEPHONE is a Phase 3 randomised non-inferiority trial comparing 6 months of trastuzumab to the standard 12 months in patients with HER2 positive early breast cancer. Patients consented to a quality of life sub-study where information was collected about their experiences. Collecting 'quasi-qualitative' data via open-ended questions adds depth and complements quantitative quality of life data. It allows patients to report experiences that may otherwise remain unknown.
**Methods:** Alongside the toxicities reported on the trial case report forms (CRF) and patient booklets being collected, including quality of life (QoL) and Health Care Resource Usage, patients were invited to record any other comments they had about their treatment. Experiences were recorded prior to commencement of trastuzumab, then 3-monthly for a year, then every 6 months up to year 2. Within a mixed methods framework, both the trial researcher and patient representatives explored the information collected using thematic content analysis.
**Results:** Between October 2007 and July 2015, 4088 patients were randomised. In total, 5542 experiences were recorded from 2456 patients across the 6 time-points. Patients offered information on all aspects of the study, including their views on the treatment, their care, the QoL questionnaire and the research. Most often mentioned was the impact the treatment had on participants personally - physically, psychologically or socially. Most frequently cited were aches, pains and fatigue; for many, these did appear to be particularly distressing and intractable. In parallel, CRFs reported 20% of patients reporting a grade 3/4 toxicity during treatment (23% 12 month, 18% 6 month, $p=0.004$), with significantly higher rates of cough, pain, fatigue, chills and palpitations reported by patients having 12 months trastuzumab ($p<0.05$).
**Conclusions:** Patients' experiences during and beyond trastuzumab highlighted the long-term cumulative effects of their treatment and confirm that patients do suffer from burdensome toxicity, which affects their QoL.

## P-113

**Telephone interviews versus postal questionnaires in rehabilitation and clinical care clinical trials**

Ranjit Lall, Katie Booth
Warwick Clinical Trials Unit, Coventry, United Kingdom
*Trials* 2019, **20(Suppl 1):**P-113

**Introduction:** Several methods for collecting follow-up data from participants who take part in research studies have been cited in the literature and these include the use of the postal questionnaires, face-to-face interviews, telephone interviews, and use of the Internet. The postal questionnaire is the most frequently used and is considered to be the most cost effective but is often associated with the lowest response rate. Poor response to questionnaires is known to reduce the statistical power of the study as the effective sample size is reduced. It can also introduce bias if the nonresponders are systematically different on outcomes of interest to those who respond to the questionnaires. Lall et al (2012) reported that collecting data by telephone on patients who had not responded to questionnaires significantly increased the response rate and enhanced the treatment difference on one trial of back pain (Back Skills Training Trial).
**Methods:** We have taken a range of clinical trials within the rehabilitation and critical care fields (CHESS, I-WOTCH and BREATHE) and analysed the primary and secondary outcomes that were collected by postal questionnaire and if missing using this mechanism of data collection, were then collected by telephone. We have aimed to assess whether the additional information increases response rates, increases the patient representation and enhances the treatment effort across and within all the clinical trials.

## P-114

**Abstract omitted**

## P-115

**Priority safety data issues in late phase clinical trials: A qualitative approach from an academic clinical trials unit**

Genevieve Helen Wills, Alejandro Arenas-Pinto, David Dunn, Ruth Goodall
MRC Clinical Trials Unit at UCL, London, United Kingdom
*Trials* 2019, **20(Suppl 1):**P-115

**Introduction:** Collection, reporting and analysis of efficacy outcomes are the main focus for late phase clinical trials. These aspects have been relatively neglected in relation to safety data and this study aims to identify areas in need of improvement.
**Methods:** Interviewees were employees of the MRC CTU at UCL with experience working with safety data in clinical trials (analysis, collection, oversight of trial management). Potential interviewees were contacted via email, sent a protocol and information sheet, and invited to join. In-depth, semi-structured interviews were conducted on all participants, lasting between 30-60 minutes. Verbatim transcripts were produced, and content analysis was used to identify common themes.
**Results:** In total, 9/13 (69%) people responded favourably and were interviewed; 4 statisticians, 3 clinicians and 2 senior staff within trial/study management. Multiple areas were raised during interviews and flagged in analysis; four areas were identified in over half of the interviews.
Relatedness assessments:Collected to fulfil a regulatory requirement but not in themselves a useful measure. Subjectivity in assigning relatedness between and within assessor.

**Counting**: Information on the same safety event is collected in multiple places, particularly SAEs, recurrent events and those with associated symptoms. Over counting of events is a concern. Hard to define unique events.
**Collection:** Too much safety data is collected in general (seen as detrimental). Some trials could collect less safety data than others e.g. if using licensed drugs, phase III, in an academic trial setting.
Chronic/genetic/pre-existing conditions:Not well documented at randomisation and no consensus on how to handle these events in analysis
**Discussion:** Experienced trialists working on late phase clinical trials identified four key areas related to the collection, analysis and reporting of safety data which would benefit from further research. These findings reflect the general practice at one CTU and the experience of those interviewed but are likely to be applicable in a wider setting.

## P-116

**Abstract omitted**

## P-117

**Abstract omitted**

## P-118

### Successfully optimising trial recruitment via embedded qualitative studies: what's different about paediatric surgical trials in urgent care?

Bridget Young[1], Francis Sherratt[1], Lucy Beasant[2], Esther Crawley[2], Nigel Hall[3]
[1]University of Liverpool, Liverpool, United Kingdom; [2]University of Bristol, Bristol, United Kingdom; [3]University of Southampton, Southampton, United Kingdom
*Trials* 2019, **20(Suppl 1):**P-118

**Background:** We embedded a qualitative study within a feasibility trial of non-operative treatment versus appendicectomy for acute appendicitis in children (aged 4-15 years), with the aim of optimising trial recruitment. Previous optimisation work has focussed mostly on trials for adult patients in non-urgent care settings. Here we identify distinctive challenges and lessons for children's urgent care surgical trials.
**Methods:** Across three UK children's hospitals we audio-recorded 85 recruitment consultations from 58 families, and interviewed 35 health professionals, 34 parents and 14 children. Thematic data analysis informed training for recruiters and helped refine trial information for families.
**Results:** Following recruiter training, recruitment rates increased from 38% to 62%. Training focussed on presenting the trial arms in a balanced way, exploring family treatment preferences and refining communication about the trial's pathways. Recruiters' presentation of the trial became increasingly balanced following training, but training on preference exploration was only partially implemented. Few recruiters elicited the reasons underlying families' treatment preferences and when families volunteered reasons, recruiters rarely responded with tailored counterbalancing of preferences. Time constraints and concerns about coercion curtailed such counterbalancing. However, parents and recruiters were also concerned about children being exposed to conversations about treatment risks – which is often part of counterbalancing – and this further complicated treatment preference exploration. Lessons for further refining communication about children's trials in the urgent care settings include clarity about the timing of treatments, and sensitivity in the communication of treatment allocation, and in post-surgery discussions with families.
**Conclusions:** This qualitative study helped recruiters to present the trial in more balanced ways to families and led to increased recruitment, despite partial implementation of the training. We identify distinctive complexities in exploring treatment preferences in children's urgent care trials, but also distinctive opportunities to enhance communication and recruitment to similar trials in the future.

## P-119

### User-focused research to identify the benefits of digital tools for the recruitment and retention in trials: A qualitative study

Amanda Jane Blatch-Jones[1], Abby Bull[1], Jacqui Nuttall[2], Gareth Griffiths[2], Jeremy Wyatt[3]
[1]NIHR Evaluation Trials and Studies Coordinating Centre, Southampton, United Kingdom; [2]Southampton Clinical Trials Unit, University of Southampton, Southampton, United Kingdom; [3]Wessex Institute, Faculty of Medicine, University Of Southampton, Southampton, United Kingdom
*Trials* 2019, **20(Suppl 1):**P-119

**Introduction:** Digital tools are increasingly being used to identify, recruit and retain participants. While these tools are being used, there is a lack of quality evidence to determine their value in trial recruitment and retention. Given the lack of certainty around the evidence-base, there is a need to improve and sustain the evidence related to the use of digital tools.
**Methods:** The aim of the main study was to identify the benefits and characteristics of innovative digital recruitment and retention tools for more efficient conduct of Randomised Controlled Trials (RCTs). The qualitative study was conducted to identify the benefits of innovative digital recruitment and retention tools for more efficient conduct of RCTs from the perspective of five stakeholder groups (including research participants).
A purposive sampling strategy was used to identify 16 participants from the five stakeholder groups. A theoretical framework was developed from results of a UK Clinical Research Collaboration (UKCRC) registered Clinical Trials Units (CTUs) survey. Semi-structured interviews were conducted and analysed using an inductive approach. A content and thematic analysis was used to explore stakeholder's viewpoint and the value of digital tools.
**Results:** Content analysis revealed that 'barriers / challenges 'and 'awareness of evidence' were the most common areas across all stakeholders. The five themes were security and transparency, inclusivity and engagement, human interaction, obstacles and risks, and potential benefits. Three emergent themes were present across all groups: 'security and transparency', 'inclusivity', and 'human interaction'. Interesting similarities and differences were also noted between practitioner and participant groups.
**Discussion:** The qualitative study outcomes show that there is a common use of digital technology for the recruitment and retention of participants in trials. Developing the evidence base in this area will be important for future research and raises important questions around the potential value for participant involvement in trials.

## P-120

### Inside a trial: contextualising outcomes within participant experience of living with multiple sclerosis

Fiona Margaret Harris[1], Selina Doran[2], Kirsteen Goodman[2], Suzanne Hagen[2], Sean Treweek[3], Christine Norton[4], John Norrie[5], Peter Donnan[6], Sarkis Manoukian[7], Doreen McClurg[2]
[1]NMAHP Research Unit, University Of Stirling, Stirling, United Kingdom; [2]NMAHP Research Unit, Glasgow Caledonian University, Glasgow, United Kingdom; [3]Health Services Research Unit, University of Aberdeen, Aberdeen, United Kingdom; [4]Faculty of Nursing, Midwifery and Palliative Care, King's College London, London, United Kingdom; [5]Edinburgh Clinical Trials Unit, University of Edinburgh, Edinburgh, United Kingdom; [6]Tayside Clinical Trials Unit, Population Health and Genomics, University of Dundee, Dundee, United Kingdom; [7]Yunus Centre for Social Business and Health, Glasgow Caledonian University, Glasgow, United Kingdom
*Trials* 2019, **20(Suppl 1):**P-120

**Introduction:**
Traditional pre-specified clinical outcome measures cannot fully capture participant experience of complex interventions; thus some benefits or unintended consequences of an intervention may remain invisible. Qualitative process evaluation is one way of addressing this, although few process evaluations attempt to contextualise and explain outcomes. This paper illustrates the explanatory value of process evaluation within a RCT of abdominal massage for neurogenic bowel dysfunction (NBD) in people with multiple sclerosis (MS).

**Methods:**

This paper draws on longitudinal semi-structured interviews with 20 intervention participants, interviewed twice. Analysis explored participant contexts and the process of change, linking participants' bowel diaries to experiences of self-massage and reported changes in bowel habits over time.

**Results:**

Fifteen of the twenty participants reported improvements that they attributed to the intervention, and the intention to continue with the massage after the trial. Investigating the lack of improvement in five participants revealed important insights including the lack of sensitivity of the outcome measures to detect change, increasing severity of MS symptoms that either affected the ability to effectively conduct the self-massage, or led to new drugs that exacerbated constipation. NBD scores (the primary outcome measure) did not always reflect perceived impact of self-massage reported during interviews; some interviewees who reported no improvement nevertheless showed an improved NBD score; and the reverse was also true. Some reported benefits were not captured by primary or secondary measures.

**Discussion:**

Process evaluation findings contextualised trial results within participant experiences. The trial primary outcome finding favoured the intervention group, but the effect size was small and neither clinically nor statistically significant ($p = 0.0558$). However, we demonstrated positive benefits in the process evaluation subgroup that would not otherwise have been detected, suggesting that the impact of NBD is such that even small improvements not detected by outcome measurement may be important to patients.

## P-121

### Participant views of taking part in a tailored cancer awareness intervention: Preliminary participant interview findings from the Awareness and Beliefs About Cancer Trial (ABACus3)

Yvonne Moriarty[1], Harriet Quinn-Scoggins[2], Fiona Wood[2], Julia Townson[1], Kate Brain[2], Vasiliki Kolovou[1]
[1]Centre for Trials Research, Cardiff University, Cardiff, United Kingdom; [2]Division of Population Medicine, School of Medicine, Cardiff University, Cardiff, United Kingdom
*Trials* 2019, **20(Suppl 1):**P-121

**Introduction:** Cancer symptom awareness is lower in disadvantaged communities resulting in delayed help-seeking, late stage diagnosis, and poorer long-term outcomes. Evidence suggests that tailored community-based cancer awareness interventions can be successful at engaging low sociodemographic groups. We tested a tailored health-check intervention delivered by trained lay advisors in disadvantaged communities. The health-check assessed cancer symptoms, risk and screening behaviours and summarised results in a traffic light system with behaviour change advice delivered by the advisor.

This paper presents participants' views of taking part in the intervention.

**Methods:** Participants were purposefully sampled from the ABACus3 trial to take part in semi-structured telephone interviews at 4-6 weeks or 6 months post-randomisation. Sampling criteria included trial allocation, recruitment setting, gender, age and geographical location. Interviews were audio-recorded, transcribed and analysed using thematic analysis supported by NVivo11.

**Results:** Fifteen interviews were completed 4-6 weeks post-randomisation and to date thirteen interviews have been completed 6 months post-randomisation. 6-month interviews will be completed by August 2019.

Some participants reported being very knowledgeable about specific cancers/symptoms before taking part based on direct personal experience or caring for someone with cancer. Tailored behaviour change advice was taken on board by some who reported changing behaviour to reduce their cancer risk; however, many described being limited by factors outside of their control such as disability and accessing services. Participants showed some awareness of national campaigns, although were often unable to name them or specify what cancer/symptom they were raising awareness for.

**Discussion:** Preliminary results suggest that a tailored community-based intervention could be a successful method for raising awareness of cancer symptoms to people from low socioeconomic groups and in supporting them to identify how best to reduce their risk. Further work is needed to understand how any change implemented supports long-term awareness and risk reduction in this population.

## P-122

### When is enough enough? Patient, relative, and health care professionals experiences of research information provision in the intensive care unit

Katie Paddock[1], Lucy Frith[1], Carrol Gamble[2], Ingeborg Welters[3,4], Kerry Woolfall[1], Bridget Young[1]
[1]Department of Health Services Research, Institute of Population Health Sciences, University of Liverpool, Liverpool, United Kingdom; [2]Department of Biostatistics, Institute of Translational Medicine, University of Liverpool, Liverpool, United Kingdom; [3]Critical care, Royal Liverpool and Broadgreen University Hospitals NHS Trust, Liverpool, United Kingdom; [4]Department of Musculoskeletal Biology, Institute of Ageing and Chronic Disease, University Of Liverpool, Liverpool, United Kingdom
*Trials* 2019, **20(Suppl 1):**P-122

**Introduction:** Recruiting participants for research in the intensive care setting (ICU) is challenging, yet there is little evidence on stakeholder perspectives to inform consent practices. Our objectives were to investigate the experiences of stakeholders - patients, relatives, and health care professionals (HCPs) - with the overall aim of informing good practice guidance on consent to ICU studies.

**Methods:** Mixed methods study comprising surveys and in-depth qualitative interviews were conducted in 14 English ICUs.

**Results:** Surveys included in the analysis were from patients (n=115) and relatives (n=157) who had been approached about participating in a research study. HCPs with a research role (n=69) and HCPs with no research role (n=477). A total of 55 patients, relatives and HCPs were interviewed. Survey responses from patients and relatives indicated that most had enough time to consider participation (78%/80%), did not find it hard to take-in information about a study (55%/55%), or make a decision about participation (66%/58%). In contrast, most HCPs believed that patients/relatives found it hard to take-in information (63%) and to make a decision about participation (53%). Interviews indicated that these contrasting perspectives arose from differences in what patients/relatives and HCPs perceived was 'enough' time and information to understand what is being asked of them, and to make a decision. Some HCPs doubted that patients/relatives reached a sufficient understanding of a study, whereas patients/relatives prioritised acquiring enough information to discern what activities a study involved, and its impact on the patient.

**Discussion:** Patients and relatives held more favourable views of aspects of recruitment and consent to ICU research than HCPs anticipated. Interviews indicated that stakeholders also had different priorities regarding information provision, with patients/relatives emphasising the potential implications of participation for the patient. Centring information provision on the priorities of patients/relatives could minimise confusion and concern, and in turn, optimise recruitment.

## P-123

### Abstract omitted

## P-124

### Developing a measure of participant experience in randomised trials: qualitative study of trial participant and professional perspectives

Nicola Small[1], Claire Planner[1], Ailsa Donnelly[1], Caroline Sanders[1], Katie Gillies[2], Katrina Turner[3], Bridget Young[4], Peter Bower[1]
[1]University of Manchester, United Kingdom; [2]University of Aberdeen, United Kingdom; [3]University of Bristol, United Kingdom; [4]University of Liverpool, United Kingdom
*Trials* 2019, **20(Suppl 1):**P-124

**Introduction:** Involving members of the public in designing trials helps to improve recruitment and retention, but we also need a way of measuring participant experience. Most trials collect process and outcome measures, yet few routinely measure patient experience; measures that do exist have not been psychometrically tested.
**Aims:**
(1)To understand participant experience in trials;
(2)To understand advantages and challenges of using participant experience data to improve trial design and delivery.
**Methods:** Eleven professional stakeholders involved in trials were interviewed about their views on measuring participant experience, and what should be measured. Twelve trial participants were asked about their experience of participation. Six members of our Patient and Public Involvement Group (PPI) met to discuss what should go into a participant experience measure.
**Results:** Experience involved nine domains (Information provision; deciding to take part; trial logistics; treatment preferences; engaging with staff; knowing results; enabling future research; participant outcomes; and overall satisfaction) covering the participant 'journey'; PPI members gave insights of topics to cover in relation to each stage of the journey.
Trial participants described contributing to something worthwhile, yet they seek support throughout their journey to avoid feeling 'abandoned'. Trial logistics (e.g. flexibility of appointments), and trial processes (e.g. capturing expectations around participating), have a role in determining future participation.
Professional participants highlighted the importance of understanding how trial design impacts experience to inform future delivery; being able to compare data over time; and unravelling the experience of intervention and trial processes. Common concerns of using experience data included a potential workload burden among participants and professionals, and negatively impacting on relationships.
**Discussion:** Our work outlines the domains needing measurement, and the challenges of using experience measures in trials. We will discuss the implications for the development of our participant experience measure and share learning from our pilot study.

## P-125

### Recruitment observed and recruitment experienced – exploring the differences: qualitative study

Nicola Farrar[1], Jenny Donovan[1], Daisy Elliott[1], Marcus Jepson[1], Leila Rooshenas[1], Bridget Young[2]
[1]Population Health Sciences, University of Bristol, Bristol, United Kingdom; [2]Institute of Population Health Sciences, University of Liverpool, Liverpool, UK
*Trials* 2019, **20(Suppl 1):**P-125

**Background:** Recruiting patients to randomised controlled trials (RCTs) can be challenging. Recruitment research often involves either analysis of observations of doctors and nurses explaining the trial to patients during 'recruitment appointments', or elicitation of patients' experiences through qualitative interviews. However, little research has compared recruitment as observed with recruitment as experienced. This project aims to understand patients' decision-making about RCT participation by comparing what is communicated to them during recruitment appointments with patients' interpretations of this communication. This is achieved by linking audio-recorded appointments with follow-up patient interviews from three UK-based multicentre RCTs.
**Methods/Potential Results:** Data from 18 interviews with 17 patients (14 consented, 3 declined) and 10 audio-recorded appointments (for 7 patients) have been collected for RCT 1, which compares routine treatment (arm 1), with routine treatment plus additional intervention (arm 2) for a life-limiting condition. Data are being analysed thematically. Preliminary findings suggest that patients' motivations for participation were often linked to their hope to benefit from the additional intervention and reported altruism. Observations of appointments revealed that the uncertainty around the benefits of the intervention were not always clearly communicated by recruiters, although further analysis of these data is ongoing. As such, most patients had a preference for arm 2 and many misunderstood the study design. Data collection is ongoing for RCTs 2 and 3.
**Potential Impact:** This work will address the knowledge gap between the information that is provided to patients during an RCT consultation and how that relates to a patient's decision-making about trial participation. Findings will inform training and resources for recruiters to optimise trial recruitment and informed consent. By synthesising findings from audio-recordings and patient interviews collected across three RCTs, this work will identify considerations for patient recruitment that may be transferable across RCTs.

## P-126

### Negotiating uncertain findings; using process evaluation to support the interpretation of randomised controlled trials

Paul Leighton[1], Jo Chalmers[1], Garry Meakin[1], Rachel Haines[1], Kim Thomas[1], Jennifer White[1], Jonathan Batchelor[1], Miriam Santer[2]
[1]University Of Nottingham, Nottingham, United Kingdom; [2]University of Southampton, Southampton, United Kingdom
*Trials* 2019, **20(Suppl 1):**P-126

**Introduction:** Vitiligo is a condition resulting in white patches on the skin. It is a condition that is often poorly managed, partly due to lack of high-quality evidence to inform clinical care. People with vitiligo can suffer from low self-esteem, psychological disturbance and diminished quality of life.
The HI-Light Vitiligo Trial is a clinical trial involving 440 participants aged 5 years and over with vitiligo. The purpose of this study is to test the effectiveness of home-based light therapy using hand-held units and topical steroid for the treatment of vitiligo.
The HI-Light Vitiligo Trial includes a nested process evaluation.
**Methods:** The process evaluation includes: interviews with trial participants (incl. adults, young people (aged 12-17) and the parents of children included in the trial); interviews with NHS commissioners; an online survey of recruiting sites; and, discussion groups with clinicians and nurses involved in delivering treatments as part of the trial.
**Results:** Participants understood the treatments provided and had realistic expectations of improvements that might be achieved. Adherence to treatment was good, despite complicated regimens.
Healthcare professionals were positive about the potential treatment options and were supportive of recommendations for NHS delivery of home-based, hand-held light therapy for vitiligo. Healthcare professionals demonstrated some concerns about safety issues, about the complexity of treatment, and consequently suggested that any clinical service might have precise criteria for patient eligibility.
**Discussion:** Clinical data demonstrated a statistically significant difference in treatment options, with combination therapy (incorporating both light therapy and topical steroids) better than any individual treatment. However, wide confidence intervals suggest some uncertainty in the clinical significance and interpretation of these findings.
The process evaluation might offer a critical lense to re-interpret the clinical significance of these findings: that new treatments are badly needed for this population; that a well-managed service might have positive impact.

## P-127

### Informed consent in the context of surgical innovation: A qualitative study of stakeholders' views

Jez Zahra, Daisy Elliott, Sangeetha Paramasivan, Kerry Avery, Sian Cousins, Natalie Blencowe, Barry Main, Jane Blazeby
National Institute for Health Research Bristol Biomedical Research Centre, United Kingdom
*Trials* 2019, **20(Suppl 1):**P-127

**Introduction:** Informed consent forms an integral part of modern healthcare and is a critical step in supporting patient autonomy. Research exploring informed consent for surgery has generally centred on understanding consent in the context of established surgical procedures or randomised controlled trials. Consequently, very little is known regarding consent processes for innovative surgical procedures delivered in the context of early phase evaluation studies or as part of everyday routine clinical practice.
Aim: To explore stakeholders' views on information provision and informed consent in the context of surgical innovation.
**Methods:** In total, 43 one-to-one interviews with surgeons and governance representatives were conducted. Purposive and snowball sampling methods were used to ensure participants were from varying geographical locations and surgical specialities. Interviews were digitally audio recorded before being transcribed verbatim and transferred to data management software for thematic analysis.
**Results:** Personal accounts of informed consent discussions suggest innovative surgeries are commonly introduced as part of routine practice, outside of a formal research framework. Data indicates regulatory oversight of consent processes in this context is lacking and varies between trusts. While all interviewees felt information that extends beyond that provided during for standard surgery should be communicated, there are uncertainties surrounding the content and volume of information needed. Generally, interviewees felt that patients are receptive to innovative surgery, however, many perceive patients to have a poor understanding of information when providing consent.
**Discussion:** While participants valued the importance of consent, perspectives on what information is required during consultations for innovative surgery vary. More consistent and stringent regulation within trusts may help to improve patient information provision and consent processes. Collectively, findings suggest a need for standardised approaches to information provision and consent for innovative surgical treatments.

## P-128

### Recruitment methods for sarcopenia trials – lessons from the LACE randomised controlled trial

Margaret Band[1], Cheryl Hume[1], Kristina Pilvynte[1], Marcus Aitchison[1], Karen Smith[1], Alison Avenell[2], Paul Kemp[3], Peter Donnan[1], Adrian Hapca[1], Allan Struthers[4], Deepa Sumukadas[5], Jacob George[4], Miles Witham[1,6]
[1]Tayside Clinical Trials Unit, University of Dundee, Dundee, United Kingdom; [2]Health Services Research Unit, University of Aberdeen, Aberdeen, United Kingdom; [3]National Heart and Lung Institute, Imperial College, London, United Kingdom; [4]School of Medicine, University of Dundee, Dundee, United Kingdom; [5]Dept of Medicine for the Elderly, NHS Tayside, Dundee, United Kingdom; [6]AGE Research Group, NIHR Newcastle Biomedical Research Centre, Newcastle upon Tyne, United Kingdom
*Trials* 2019, **20(Suppl 1):**P-128

**Introduction:** Sarcopenia in older people, though common, is rarely diagnosed and recorded electronically, posing challenges to recruitment. We describe strategies to efficiently recruit participants to a factorial trial of perindopril and leucine for sarcopenia.
**Methods:** Primary care recruitment screened lists from collaborating primary care practices, and sent mailshots to patients aged 70 and over, not taking ACE inhibitors. Secondary care recruitment took place via inpatient and outpatient geriatric medicine services. Local research nurses screened clinic notes and approached potentially eligible patients. Telephone pre-screening, using the SARC-F questionnaire, was conducted either by research nurses at site or centrally by Tayside Clinical Trials Unit. De-identified recruitment information was held on an electronic tracking system run by Dundee Health Informatics Centre and analysed using SPSS.
**Results:** Thirteen UK sites undertook screening. From primary care, 13808 invitations were sent. 2955 (21.4%) responded positively and 557/2897 (19.2%) participants eligible at pre-screening were sent study information. 282 attended a screening visit, and 138 (1.0% of total contacted) were randomised. 633/2897 primary care respondents were pre-screened centrally with the mean number of calls per respondent 2.3. The conversion rate from pre-screening to randomisation was 18/633 (2.8%) for centralised calls, compared to 120/2264 (5.3%) for local pre-screening calls (p=0.01). A weak relationship was seen between higher (worse) SARC-F score at screening and lower likelihood of progression to randomisation (r=-0.07). In secondary care, 1202 sets of notes were screened at sites with 160 potential participants offered information. 24 agreed to attend screening visits, and 7 (0.6% of total notes screened) were randomised.
**Discussion:** Primary care recruitment led to higher response rates and overall numbers randomised than secondary care recruitment. Centralised pre-screening saved local research nurse time but may have been less effective. SARC-F was of limited help in targeting screening activity in this sarcopenia trial.

## P-129

### SWAT: Can social media advertising enhance trial recruitment?

Fay Chinnery[1], Nick Francis[2], Gareth Griffiths[1], Paul Little[3], Ingrid Muller[3], Jacqui Nuttall[1], Matt Ridd[4], Beth Stuart[3], Tracey Sach[5], Irene Soulsby[6], Karen Thomas[6], Kim Thomas[7], Alison Layton[8], Miriam Santer[3], Louise Stanton[1]
[1]Southampton Clinical Trials Unit, University of Southampton, UK; [2]Division of Population Medicine, Cardiff University, UK; [3]Primary Care and Population Sciences, University of Southampton, UK; [4]Population Health Sciences, University of Bristol, UK; [5]Health Economics Group, Norwich Medical School, University of East Anglia, UK; [6]PPI contributor, UK; [7]Centre of Evidence Based Dermatology, University of Nottingham, UK; [8]Hull York Medical School, University of York, UK
*Trials* 2019, **20(Suppl 1):**P-129

**Introduction:** The SAFA (Spironolactone for Adult Female Acne) RCT is aiming to determine the clinical effectiveness of spironolactone compared with placebo in addition to standard care in the treatment of moderate-severe persistent facial acne in adult women.
The trial recruits participants at 5 UK hospital sites. But potential participants are approached via a number of other routes including: local General Practices acting as Participant Identification Centres and community advertising. Social media advertising is being used to supplement these strategies.
**Methods:** The social media campaign consists of 2 approaches:
1.Targeted Facebook adverts. Facebook can target people by demographics and search history and geographical location. People on Facebook who have shown an interest in acne or relevant organisations linked with the condition and who fit the profile demographic are shown adverts for SAFA. If interested, they are directed to the trial website.
2.Google AdWords campaign. This approach targets people searching Google for acne drugs and treatments and directs people to the trial website. The trial website provides more information about the study and explains how to contact the local site study team.
**Timing of potential results:** SAFA opened in May 2019 and sites will recruit without using social media for the first 3 months. The Facebook adverts will be used first and their effect assessed after 3 months. If the Facebook adverts have not sufficiently boosted recruitment, or the trial team thinks the trial will benefit from broader advertising, the Google AdWords campaign will start. Impact will also be assessed after 3 months.
**Potential relevance and impact:** We will see if social media advertising can augment trial recruitment. Given that recruitment can be one of the most challenging aspects of a study, these findings will be of interest to anyone involved in clinical trials.

## P-130
**Abstract omitted**

## P-131
### Overcoming recruitment challenges in the ABACus3 cancer awareness intervention directed at the most socioeconomically disadvantaged groups in Yorkshire and South-Wales, UK

Vasiliki Kolovou, Louise Padgett, Sioned Owen, Yvonne Moriarty, Stephanie Gilbert, Harriet Quinn-Scoggins, Julia Tonwson, Kate Brain
Centre For Trials Research, Cardiff University, Cardiff, United Kingdom
*Trials* 2019, **20(Suppl 1):**P-131

**Introduction:** Recruitment for health intervention trials in socioeconomically deprived areas is often difficult due to issues like poor engagement, distrust, uncertainty and poor communication.
This paper reports on the recruitment strategies used in the Awareness and Beliefs about Cancer (ABACus3) trial, which tested the effectiveness of a tailored health check to raise awareness of cancer symptoms in areas of high deprivation of Yorkshire and South-Wales.
**Methods:** Participants (aged 40+) were recruited from healthcare (GP surgeries, pharmacies) or community (libraries, community-hubs, job-shop) settings using an opportunistic or appointment-based approach. Lay advisors identified and approached suitable venues. Recruitment methods varied by setting type and were regularly reviewed and adapted according to need. Site logs were kept to track the interest of venues and recruitment logs recorded numbers of individuals approached and recruited. Lay advisors were interviewed before and after recruitment to gain an understanding of the recruitment methods used.
**Results:** A total 113 venues were contacted to take part in the study via email, phone and personal visits, of which 42 venues became recruitment sites (n=13 Healthcare, n=29 Community). Recruitment strategies were adapted based on-site availability, study needs (i.e. achieving participant balance) and geographical spread. This allowed for a broad range of participants (n=234/448) to be recruited across all target areas. Recruitment maps depicted good reach within each area. Community setting recruitment was more successful than healthcare settings (n=174 community, n=60 healthcare). Lay advisor interviews indicated that this was due to high staff engagement and support.
**Discussion:** Building positive relationships between lay advisors and recruitment site staff were vital to ensure positive recruitment outcomes. Focusing on community settings and arranging planning meetings in advance was key to successful engagement. In turn, this facilitated high recruitment with members of the public who were more likely to engage with trusted site staff.

## P-132
### Managing and predicting recruitment for external pilot studies: could predictions have been better? A review of NWORTH data 2006-2018

Zoe Hoare
Bangor University, Bangor, United Kingdom
*Trials* 2019, **20(Suppl 1):**P-132

**Introduction:** Study recruitment can be affected by a number of issues with evidence to show that only around 55% of studies achieve their target sample size. Not being able to recruit to target can undermine the entire study but for external pilot/feasibility studies this reduces the precision of any further estimates that can be generated. Prediction of recruitment timeframes for external pilot/feasibility studies have the additional complexity of having limited prior knowledge and data.
Could prediction techniques in the literature have improved the estimates and expectations of external pilot/feasibility studies run in North Wales Organisation for Randomised Trials in Health (NWORTH) clinical trials unit (2006-2018)?
**Methods:** Data from all randomised external pilot/feasibility studies completed between 2006 and 2018 was extracted. The predictions made using the unconditional model and the actual recruitments achieved are compared. Subsequently whether the predictions could have been improved using a conditional model or homogenous and non-homogenous Poisson processes were all examined using measures of expected time to recruit and recruitment rates.
**Results:** Data was extracted for 17 studies. Only 24% managed to achieve their target sample size, increasing to 47% for those which achieved >80% of their target. The planned recruitment rate was overoptimistic in 88% of studies with only one study achieving recruitment in the time planned. Predictions for the planned recruitment period varied very little between the four prediction methods tested.
**Discussion:** Recruitment predictions for the NWORTH studies during 2006-2018 would not have been altered significantly using any of the proposed methods. Intuitively modelling using a non-linear recruitment process is more reasonable allowing for uncertainty and set-up. With external pilots being used to optimise recruitment strategies it would be sensible to consider a longer recruitment window to allow the testing of alternative strategies and assessment of impact rather than solely aiming for a target.

## P-133
### ELECtric Tibial nerve stimulation to Reduce Incontinence in Care homes. Findings from the ELECTRIC trial pilot

Joanne Booth, Catriona O'Dolan, Lisa Macaulay
Glasgow Caledonian University, Glasgow, United Kingdom
*Trials* 2019, **20(Suppl 1):**P-133

**Introduction:** Urinary incontinence (UI), is highly prevalent in residential and nursing care homes (CH), and profoundly impacts on residents' dignity and quality of life. UI affects physical and cognitive functioning, disturbs sleep, and increases risk of falls, fractures, and pressure ulcers. Currently, CHs predominantly use absorbent pads to contain UI rather than active treatment. Transcutaneous posterior tibial nerve stimulation (TPTNS) is a non-invasive, safe, low-cost intervention that has demonstrated effectiveness for reducing UI in adults. However, the effectiveness of CH staff delivering TPTNS to residents is yet to be established. ELECTRIC is a multicentre, placebo controlled, randomised parallel group trial that aims to compare effectiveness of TPTNS with placebo stimulation, to reduce UI in CH residents (protocol available at www.nihr.ac.uk).
**Methods:** A 6-month internal pilot aimed to test feasibility before proceeding to full trial using pre-determined progression criteria addressing: 1) recruitment rates; 2) adherence to stimulation programme; 3) completion of primary outcome measure; 4) fidelity to stimulation group. Data for each criterion
were assessed against targets using a traffic light system.
**Results and Discussion:** The ELECTRIC pilot achieved green (progress to full trial) for criteria 1-3 (recruitment = 97 residents randomised, target >90; adherence = 77% received >8/12 stimulations, target >70%; primary outcome = 88% completeness of 24 hour pad collection, target >70%). Criterion 4 was amber with >2 fidelity checks correct (for time, positioning, intensity) in 69% residents, target >70%. To improve fidelity, checks are now made by trial implementation support facilitators not CH PIs, with first checks made soon after stimulations start, so errors can be rectified early. Progression to full ELECTRIC trial was confirmed; final results due June 2020.
ELECTRIC is funded by the National Institute for Health Research.

## P-134

### Assessing the impact of site engagement on patient recruitment

Furrah Hussain[1], Marcus Achison[1], Clare Clarke[1], James Chalmers[2]
[1]Tayside Clinical Trials Unit, University of Dundee, Dundee, United Kingdom; [2]Department of Respiratory Medicine, University of Dundee, Dundee, United Kingdom
*Trials* 2019, **20(Suppl 1):**P-134

**Introduction:** Participant recruitment is crucial to the success of a study. One TCTU-led multi-centre trial currently has varying levels of recruitment across sites: 2 sites recruiting and randomising above their target and 2 sites unlikely to reach their randomisation target before the end of the recruitment period. The objectives of this Study within a Trial (SWAT) are two-fold. 1. To establish whether using real-time email updates of trial-wide recruitment acts as positive encouragement on recruitment across all study sites. The hypothesis being that keeping sites aware of each instance of recruitment may have a positive effect on recruitment, possibly by showing that recruitment is achievable or by introducing competitiveness across sites. 2. To determine if site engagement, i.e. attendance at monthly teleconferences, has an impact on recruitment.
**Methods:** 1. At the beginning of the trial, congratulatory emails were sent to individual sites each time they randomised a participant. As a result of the intervention, email notifications are now sent to all sites after each randomisation. The randomisation rate for 6 weeks prior to the intervention will be compared with the rate in the 6 weeks following the intervention 2. Teleconference attendance by site staff during the recruitment period will be monitored and recruitment numbers vs teleconference attendance at each site compared.
**Timing of Potential Results:** June 2019 (end of recruitment period).
**Potential Relevance & Impact:** Improving recruitment using email-based recruitment updates as motivational techniques for site staff and increasing participation of site staff in teleconferences may prove to be powerful tools for boosting recruitment at under-performing sites throughout the recruitment phase of a study. This in turn may change the way in which trial management responds to participant randomisation notifications and lead to improvements in the randomisation notification procedure for future trials.

## P-135

### Common problems recruiting to trials or specific challenges? A qualitative evaluation of factors affecting recruitment into a randomised controlled trial of foster care training

Gwenllian Moody, Mike Robling, Elinor Coulman, Lucy Brookes-Howell, Rebecca Cannings-John, Sue Channon, Mandy Lau, Alyson Rees, Jonathan Scourfield, Jeremy Segrott
Cardiff University, United Kingdom
*Trials* 2019, **20(Suppl 1):**P-135

**Introduction:** Randomised controlled trials (RCTs) are comparatively rare in UK social work but can offer distinct advantages. Confidence in Care (CiC) is an RCT with embedded process evaluation evaluating Fostering Changes (FC), a 12-week training programme for foster and kinship carers to increase skills and coping strategies. A challenge in most trials is participant recruitment. We designed an engagement strategy and tailored our approach to maximise carer recruitment, and target sample size was surpassed. To better understand setting-specific issues in recruitment we undertook a process evaluation incorporating key study stakeholder groups.
**Methods:** Focus groups were conducted, two with field-based recruiting staff (n=7), one with carers who attended the FC programme (n=8). Five interviews were conducted with facilitators who delivered FC. Transcribed audio-recorded data were inductively coded, double-coded by a second researcher, and thematically analysed.
**Results:** Six themes were identified. The first addressed aspects of the intervention affecting recruitment (e.g. committing to a 12-week training programme). A second focused on accuracy of communication between provider agencies and carers. A third concerned the ability of recruiting staff to contact carers, a particular challenge in group-based recruitment. A fourth addressed trial-related aspects (e.g. the relationship between trial team and recruiting staff). A fifth explored the lack of differentiation by carers between the roles of the various professionals (e.g. FC facilitators and provider agencies). The sixth addressed observations by stakeholders of differences between recruitment into social care and health studies.
**Discussion:** Recruitment challenges in this social care setting were similar to those in healthcare. Some (e.g. gatekeeping by professional staff) may be rooted in randomisation anxiety, or unfamiliarity with research methods. Researchers more familiar with healthcare recruitment were however encouraged about the experience of working in this care setting. The original recruitment strategy and adaptations form the basis of further recommendations for research practice.

## P-136

### Net Casting Recruitment in Personalised medicine for Asthma ConTrol

Pauline Armory[1], Roberta Littleford[1], Somnath Mukhopadhyay[2], Kristina Pilvinyte[1]
[1]Tayside Clinical Trials Unit, Dundee, United Kingdom; [2]Royal Alexandra Children's Hospital, Brighton & Sussex Medical School, Brighton, United Kingdom
*Trials* 2019, **20(Suppl 1):**P-136

**Introduction:** The 2015 Nuffield Bioethics report states ‘the time has come to protect Children and Young People(CYP) through research not from research’[1]. However low enrolment is common, notably this decline after age 14 [2] [3]. UK Children’s Research Networks, in collaboration with Primary Care and disease-specific Research Networks, have transformed CYP research access; evidenced by a two-fold increase in CYP enrolment between 2009-2019 [4]. Recruitment challenges are common across all age-groups; between 2002-2008, only 55% of non-commercial trials met recruitment target, furthermore 45% were extended with the majority continuing to fail [5]. Key CYP challenges relate to informed consent/assent [1].
The evaluation of prescribing asthma controller medication according to beta2 receptor gene status, to improve quality of life in 12-18 year olds with asthma required 240 participants. Predominantly recruited from asthma research databases, supplemented by primary and secondary care recruitment; within 23 months (including 8-month no-cost extension).
**Methods:** Commencing January 2016, PACT invitations were sent to 471 potentially eligible CYP identified from 3500 patients consenting to contact on BREATH and PAGES databases; 259 reminder mailings.
**Results:** After 8 months only 19/240 participants (8%) were recruited: 6 from the main recruitment method; 13 from primary/secondary care (4 sites). A novel wide-reaching primary-care recruitment strategy and 6-month funded extension was implemented. The rescue-plan, netted 16 CRNs and 441 GP practices. After implementation, recruitment increased by more than 100% in the preceding 8 months (60/240 – 25%) but remained behind projected target. Escalated enrollment (>300%) in the final 12 months added 187 participants. PACT exceeded target ahead of schedule: 241/240.
**Discussion:** Engagement with the Kent Surrey and Sussex NIHR Clinical Research Network [6] was fundamental to PACT achieving target. Despite regulatory challenges (new HRA approvals process, cross-border sponsorship and CYP consent processes) a UK-wide primary-care recruitment net was successfully implemented to recruit CYP.

## P-137

### Recruitment to the RAndomised IroN Deficiency anaemia management Pilot (RAINDroP)

Fiona McLaren-Neil[1], Pauline Armory[1], Phyo Kyaw Myint[2], Claire Jones[1], Amanda Cardy[3]
[1]University Of Dundee, Dundee, United Kingdom; [2]University of Aberdeen, Aberdeen, United Kingdom; [3]NHS Research Scotland Primary Care Network
*Trials* 2019, **20(Suppl 1):**P-137

**Introduction:**
The RAINDroP study is a pilot randomised clinical trial investigating the possible benefits of replacing oral iron therapy with intravenous iron in the treatment of iron deficiency anemia. One of the primary outcomes is recruitment rates, and the aim is to use multiple channels to maximise the pool of potential participants.
**Method:** Recruitment to the study is running in parallel in primary and secondary care utilising support from both primary care and ageing research networks to facilitate screening. Broad inclusion/exclusion criteria have been applied, to include co-morbidities that would be expected within the eligible population. Four sites have been selected for the study, two in Scotland and two in England. Study posters, a website and local press will also be used to boost recruitment
A secure Patient Management System (PMS) incorporating central mailing designed by the Health Informatics Centre at Dundee University is core to the administration of this multiple recruitment strategy. Where potential participants are identified their details will be securely uploadedto the PMS which can then be accessed by study staff at the relevant site.
**Timing of Potential Results:** The trial is due to end in January 2020, with a review of recruitment carried out in May 2019.
**Potential Relevance and Impact:** There is a significant population prescribed oral iron for iron deficiency anaemia but there is a lack of evidence to show the benefit of this intervention. The RAINDroP study aims to identify the most effective methods of recruitment and inform the design and sample size calculation for a future definitive trial.
The impact for patients with Iron deficiency anaemia could be a change to the treatment pathway which will include improved quality of life and physical outcomes along with reduced side effect profile should IV iron prove to be the best intervention.

## P-138

### A qualitative content analysis of education and training interventions for recruiters to trials

Hannah Delaney[1,2,4], Declan Devane[1,2], Andrew Hunter[2,3], Valerie Smith[4]
[1]Health Research Board-Trials Methodology Research Network (HRB-TMRN), National University of Ireland, Galway; [2]School of Nursing and Midwifery, National University of Ireland, Galway; [3]Qualitative Research inTrials Centre (QUESTS), National University of Ireland, Galway; [4]School of Nursing and Midwifery, University of Dublin, Trinity College Dublin, Ireland
*Trials* 2019, **20(Suppl 1):**P-138

**Introduction:** Participant recruitment is often challenging when conducting randomised trials. If recruitment targets are not met, research questions are left unanswered, wasting money and participants' time. Strategies such as training and education for trial recruiters are often implemented to improve recruitment processes. Conducted as part of a wider study (Training Recruiters-An educational Intervention (TRAIN)), this paper presents the findings of a qualitative content analysis of previous education and training interventions for trial recruiters.
**Methods:** We carried out a systematic search of published studies reporting on education and training interventions for recruiters to trials. Of the 31 full-text records assessed, 24 met the inclusion criteria for our content analysis. A directed qualitative content analysis approach was implemented using deductive categorisation, which involved four main stages: decontextualisation (data familiarisation and coding), recontextualisation (identifying un-coded text and assessing for inclusion), categorisation (grouping the codes), and compilation (presenting the results).
**Results:** We found that the training methods included mostly didactic teaching, individualised support and role play. Most of the interventions were delivered face-to-face (n=23), with few incorporating an online format (n=4). The content of the training was grouped into three main categories: contextual information (about the trial and/or the training approach), trial management (about managing the trial team and recruitment challenges/pathways/materials) and the recruitment consultation (such as informed consent and communicating randomisation).
**Discussion:** This content analysis offers a useful resource for the planning and development of education and training interventions for recruiters to trials, either in advance of a trial or in response to recruitment issues within an in-progress trial. It highlights a number of factors to consider including the format of intervention delivery, training components, content, timing and duration.

## P-139

**Abstract withdrawn**

## P-140

### Pragmatic design adaptation in response to recruitment challenges

Nuria Porta[1], Rebecca Lewis[1], Steven Penegar[1], Hugh Mostafid[2], Emma Hall[1]
[1]The Institute of Cancer Research, London, United Kingdom; [2]Royal Surrey County Hospital NHSFT, Guildford, United Kingdom
*Trials* 2019, **20(Suppl 1):**P-140

**Introduction:** CALIBER (NCT02070120) is a phase II study investigating intravesical chemoablation as an alternative to surgery for recurrent low risk non-muscle invasive bladder cancer. The primary endpoint is complete response (CR) following chemoablation. A control group was included to assess acceptance of randomisation. Participants were randomly allocated to chemoablation or standard surgical management (2:1 ratio).
**Original design:** CALIBER aimed to exclude a chemoablation CR rate <45%. Simon's 2-stage optimal design with $\alpha$=0.05, 90% power, p0=0.45, p1=0.60, required 51 chemoablation patients in the first stage. If <26 CRs were seen, recruitment would cease. If ≥26 CRs were seen, recruitment would continue to 110 chemoablation patients. If at least 58/110 chemoablation patients (60%) had CR then chemoablation would warrant phase III evaluation. With a control group and inflating for 5% non-compliance/drop-out, 174 participants were required: 116 chemoablation, 58 control.
**Recruitment:** CALIBER opened in February 2015. To March 2017, 48 chemoablation and 26 control participants had been recruited, with projected completion of recruitment (174 participants) in 2020. CALIBER's independent oversight committees therefore recommended sample size reduction to allow trial completion and suggested dropping the control group in stage 2. They stipulated that any redesign of stage 2 should be implemented prior to analysis of stage 1, to avoid outcomes influencing the design.
**Methods & results:** Adapted design
The design was amended such that:
1)The surgical control arm was dropped for stage 2
2)The power was reduced to 85% and type I error increased to 10%, with a success criterion of 31/60 chemoablation CRs. The revised target sample size (including stage 1 controls and 5% non-compliance) was 89 patients.
**Discussion:** Recruitment to trials is challenging, however collaboration with oversight committees and creative adaptations to trial design can enable trials to be completed rather than abandoned due to poor recruitment.

## P-141

### Monitoring changes in recruitment rate over time

Rosie Harris, Abby Willcox, Hana Tabusa, Jessica Harris, Barnaby Reeves
CTEU Bristol, BTC, University of Bristol, Bristol, United Kingdom
*Trials* 2019, **20(Suppl 1):**P-141

**Introduction:** Recruitment rates for grant applications are estimated before detailed information is available about relevant care pathways, times at which potential participants can be screened for eligibility, approached to provide study information, consented and randomised, and how these timings vary across participating sites. Also, in studies recruiting rare patients or over a long period, recruitment fatigue can occur. Consequently, it may be helpful to monitor changes in recruitment rate over time from site opening.
**Methods:** We analysed accruing data about dates of first contact with potential participants and consent/randomisation, and time since site opening, to generate graphs showing the average monthly recruitment rate per site for successive months since site opening, irrespective of the calendar months when sites opened. Hence, all open sites contribute to the estimate of recruitment rate for month one, but only sites open for longest contribute to the estimate for the month furthest from first recruitment into the study. We implemented these graphs as part of central monitoring in two NIHR-funded studies, one trial and one cohort study.
**Results:** In the trial, when seeking a recruitment extension, we sought reassurance that the rate of recruitment of rare patients was not declining; the graph provided the reassurance sought. In the cohort study, we wanted to understand why recruitment was slower than anticipated; the graph showed that the recruitment rate increased over the first six months open, by about 1.5 times, due to delays between first approach (time of consultant appointment) and consent (admission for surgery) and initial lack of familiarity with study processes.
**Conclusion:** In both instances, the graphs underpinned discussions with the funder. We recommend the approach as part of central monitoring, especially when constancy of recruitment rate over time since site opening is uncertain and is important for projecting future recruitment.

## P-142

### Participants declining participation in the TOPSY study: lessons learned for good clinical practice from a process evaluation

Carol Bugge[1], Melanie Dembinsky[1], Rohna Kearney[3,5], Aethele Khunda[4], Margaret Graham[1], Kirsteen Goodman[2], Lynn Melone[2], Suzanne Hagen[2]
[1]University Of Stirling, Stirling, United Kingdom; [2]Glasgow Caledonian University, Glasgow, United Kingdom; [3]Warrell Unit, St. Mary's Hospital, Manchester University Hospitals NHS Foundation Trust, Manchester Academic Health Science Centre, Manchester, United Kingdom; [4]South Tees Hospitals NHS Foundation Trust, Middlesbrough, United Kingdom; [5]University Institute of Human Development, Faculty of Medical Human Sciences, University of Manchester, Manchester, United Kingdom
*Trials* 2019, **20(Suppl 1):**P-142

**Introduction:** Recruitment is crucial for any study. Due to its importance a considerable amount of research has addressed barriers to successful recruitment. Less attention has been given to how recruiters manage participants who decline participation. As part of good clinical practice, it is important that potential participants feel reassured that they receive the best available care regardless of their decision to participate in research or not. This paper draws on evidence about recruitment that was gathered as part of a nested process evaluation within the TOPSY trial (a multi-centre randomised controlled trial that compares self-management to standard care in terms of quality of life outcomes for women using a vaginal pessary for prolapse).
**Methods:** This paper draws upon two data sources from the process evaluation: 1) audio-recordings of recruitment sessions between recruiters and potential participants (n=13); and 2) semi-structured interviews with recruiting pilot centre healthcare professionals (n=7 research nurses, specialist nurses or consultants). Data was transcribed verbatim and analysed using Framework Analysis.
**Results:** Data analysis resulted in identification of three themes: highlighting participation is voluntary; reassurance that decision does not affect care provision and emphasising patient care and wellbeing is paramount. All themes are representative of good clinical practice and how-to best support patients in their decision to decline trial participation and alleviating any concerns.
**Discussion:** As recruitment is a vital part of research it is important to address all aspects of it to ensure participant wellbeing and encourage informed decision about participation. We will contribute to the discussions surrounding recruitment by extending the focus to successfully dealing with patients declining trial participation in a sensitive manner. This allows potential research participants to feel secure in their choice to not participate while retaining a positive attitude for potential research participation in the future.

## P-143

### The effect of the method of invitation on recruitment of participants from GP practices to a trial of a smoking reduction intervention: a study within a trial

Helen Hancocks[1], Jennie King[1], Alex Gude[1], Lynn Callaghan[1], Shaun Treweek[2], Wendy Ingram[1], Tom Thompson[1], Adrian Taylor[1], Siobhan Creanor[1]
[1]Faculty of Health: Medicine, Dentistry and Human Sciences, University of Plymouth, Plymouth, United Kingdom; [2]Health Services Research Unit, University of Aberdeen, Aberdeen, United Kingdom
*Trials* 2019, **20(Suppl 1):**P-143

**Introduction:** TARS is a definitive, multi-centre RCT of tailored support as an aid to reducing smoking, recruiting participants from GP practices across four England cities (ISRCTN47776579). The preceding pilot RCT used invitation letters sent out by practices and reported a recruitment rate of 5.1%. However, six months into the TARS trial, recruitment was 1-2%. This study assessed the efficiency of three different GP practice invitation methods.
**Methods:** This was a randomised study within a trial (SWAT) and included six GP practices in one city. The TARS protocol described three different general practice invitation methods: (a) full invite pack (~£1/invite); (b) single-page invite (~£0.55/invite); (c) text message invite (£0 research cost). Postal invites were sent via DOCMAIL, a secure online mail management system used by GP practices. Practices searched electronic records to identify potentially eligible patients, who were then individually randomised (1:1:1). Patients allocated to receive text invite but who had no mobile number or had requested not to be contacted by text were excluded. The number of expressions of interest received, number recruited, and costs attributed to each method were captured.
**Results:** Of ~40,000 patients, 1377 were identified as potentially eligible for TARS. 459 were randomised to receive the full pack; 459 the brief invite; and of 459 randomised for text invite, 349 were eligible and sent the text invite. The full invitation pack resulted in the highest recruitment rate (2.8%), followed by the single-page invite (1.5%). One individual was recruited by text (0.03%). Initial invite costs per recruited participant were £35.31 and £36.06 for the full and single-page postal invite methods, respectively.
**Discussion:** Despite being the most expensive invitation method, the full invitation pack was the most efficient recruitment method. If cost is not an issue, the full invitation pack method is recommended for recruiting from GP practices.

## P-144

### A successful strategy for recruiting participants not in a care pathway to a CTIMP on time and to target

Anne Francis, Cathy Ball, Vicki Barber, Denise Condon, Heather Marie O'Connor, Susan J Dutton, Sarah Lamb, Jagdeep Nanchahal
University Of Oxford, Oxford, United Kingdom
*Trials* 2019, **20(Suppl 1):**P-144

**Introduction:** Successful and timely recruitment is known to be a challenge in conducting clinical trials. We sought to recruit 138 patients with early stage Dupuytren's disease, a benign hand condition, to determine the efficacy of a novel treatment in delaying disease progression which results in impairment of hand function.
There is no approved treatment for early stage Dupuytren's disease and patients are only referred to secondary care when they have impaired hand function and the finger joints are bent to 30 degrees or more.
Recruitment of people with early Dupuytren's disease to our clinical trial, the RIDD trial, via their existing care pathway was therefore not possible. Consequently, we used alternative strategies to ensure recruitment to target.
**Methods:** A number of strategies were used, including a website, social media and posters in GP practices. Data were collected on how people became aware of the trial to refine our ongoing recruitment strategy.
**Results:** Our most successful strategy was the provision of waiting room posters to GP practices, with accompanying information sheets for GPs. A notable number of enquiries were also received from people whose family/friends had seen our posters.
**Discussion:** We completed recruitment on schedule and our strategy could be applied to other studies where patients are not in a care pathway. In addition to recruiting such patients, this methodology could also be applied in other scenarios such as recruitment of patients at high risk of developing a disorder or to raise awareness of research projects prior to diagnosis of a condition.
Practical consideration including some of the challenges we encountered will also be presented.

## P-145

### A Concept Analysis of 'trial recruitment' using a modified Hybrid Model

Hannah Delaney[1,2], Declan Devane[1], Andrew Hunter[1], Shaun Treweek[3], Carrol Gamble[4], Nicola Mills[5], Valerie Smith[2]
[1]School of Nursing and Midwifery, National University of Ireland Galway, Ireland; [2]School of Nursing and Midwifery, University of Dublin, Trinity College Dublin, Ireland; [3]Health Services Research Unit, University of Aberdeen, Scotland; [4]Clinical Trials Research Centre, University of Liverpool, UK; [5]Population Health Sciences, Bristol Medical School, University of Bristol, UK
*Trials* 2019, **20(Suppl 1):**P-145

**Introduction:** Regulatory bodies, funders and the pubic widely accept the obligation of registration and timely reporting of clinical trials. In addition, as a condition of consideration for publication, the International Committee of Medical Journal Editors (ICMJE) requires registration of clinical trials in a public trials registry at or before the enrolment of the first trial participant. Ambiguity in anchoring participant recruitment/enrolment, and the trial start/end date, temporally to trial processes (e.g. invitation, consent, randomisation), however, has the potential for variation in how recruitment and enrolment are reported and understood in trial registries, protocols and reports. This research aims to theoretically define and operationalise 'trial recruitment' for purposes of reporting through a concept analysis.
**Methods:** A concept analysis using the Hybrid Model will be performed, involving three phases; i) the theoretical phase aims to gain an understanding of how 'trial recruitment' has been defined, used and measured in the literature; based on a systematic search of trial reports, their protocols and register details of trials published during 2018-2019 in the top five healthcare journals, and a review of these journal's policies/publication guidelines; ii) the fieldwork phase will refine the concept through focus group interviews with trial recruiters and individuals who have been previously invited to take part in a trial; iii) the analytical phase integrates the findings from the previous phases to provide theoretical and operational definitions, as well as enhanced understanding of 'trial recruitment'.
**Potential results:** We will present the final findings from the theoretical phase (complete: 08/2019) which will highlight how 'trial recruitment' is currently defined, used and measured.
**Potential impact:** Standardised reporting of completed trials is important to ensure unbiased assessments of evidence to inform health care decisions. This concept analysis will potentially enhance and standardise the reporting of trials by disambiguating the concept of 'trial recruitment'.

## P-146

### Innovative recruitment methods in surgical trials, a case study: Surgery Alone in Low Rectal cancer (SAILOR)

Kym Thorne[1], Amy Richards[1], Dean Harris[2]
[1]Swansea University Medical School, swansea, United Kingdom;
[2]Swansea Bay University Health Board, swansea, United Kingdom
*Trials* 2019, **20(Suppl 1):**P-146

**Introduction:** SAILOR was a multi-centre, feasibility trial to determine whether post-surgery outcomes at three months were better for patients with low rectal cancer who had early surgery compared with the standard method of treatment.
Participants were randomised on a 1:1 basis to receive either the intervention (early surgery alone) or standard care (pre-operative radiotherapy then surgery).
**Methods/recruitment issues:**
All eight sites reported significant recruitment issues relating to the narrow inclusion criteria, a lack of equipoise amongst consenting clinicians and, most importantly, patients' treatment preference. At one site, 9 out of 13 eligible patients cited treatment preference as the reason for declining the trial.
A modified Zelen design was introduced for the final 3 months of recruitment to determine whether it would increase the likelihood of patients consenting to the trial. The design involved two stages of consent; 1) participation and randomisation, then 2) acceptance of the randomisation result. If second stage consent was refused, the patient was offered whichever treatment they wanted and remained in the trial.
**Results:** We only consented one patient following the design change due to time constraints. This patient consented to both randomisation and the treatment they were allocated. The patient had a treatment preference at the point of consent but was randomised to that treatment anyway.
**Discussion:** Whilst no further insight could be gained into the value of the Zelen design, we believe that allowing patients the freedom to know that they can have the treatment they prefer and still be involved in research will lead to them consenting to trials.
We would suggest that other trials consider adopting the Zelen design where a treatment preference is likely and recruitment is likely to be poor due to strict eligibility or rare conditions should be considered.

## P-147

### Game of Stones: A feasibility randomised controlled trial of narrative short message system (SMS) and financial incentive interventions to support weight loss in men with obesity

Matthew McDonald[1], Stephan U Dombrowski[2], Marjon van der Pol[3], Mark Grindle[4], Alison Avenell[3], Paula Carroll[5], Eileen Calveley[1], Andrew Elders[6], Nicola Glennie[1], Cindy M Gray[7], Fiona M Harris[1], Adrian Hapca[8], Claire Jones[8], Frank Kee[9], Michelle McKinley[9], Martin Tod[10], Rebecca Skinner[1], Pat Hoddinott[1]
[1]University Of Stirling, United Kingdom; [2]University of New Brunswick, New Brunswick, Canada; [3]University of Aberdeen, Aberdeen, UK; [4]University of Highlands and Islands, Inverness, UK; [5]Men's Health Forum, Ireland; [6]Glasgow Caledonian University, Glasgow, UK; [7]University of Glasgow, Glasgow, UK; [8]University of Dundee, Dundee, UK; [9]Queen's University, Belfast, UK; [10]Men's Health Forum, England
*Trials* 2019, **20(Suppl 1):**P-147

**Introduction:** Obesity prevalence in UK men and women is comparable, yet fewer men engage in weight loss interventions and disadvantaged men are under-represented. The aim was to examine the acceptability and feasibility of a men-only weight management intervention consisting of text messages, with and without an endowment incentive, compared to waiting list control.
**Methods:** Men with obesity were recruited through community outreach and general practitioner obesity registers in two disadvantaged communities in Scotland. Participants were randomised to receive a) narrative text messages for 12 months (SMS), b) financial endowment incentives plus narrative texts messages for 12 months (SMS+I), or c) to a waiting list control. Acceptability and feasibility of recruitment, retention, engagement, and weight outcome verification were assessed by analysing quantitative and qualitative data at three, six and 12 months. A priori full trial progression criteria were set for recruitment and retention.
**Results:** 105 men from across the socioeconomic spectrum were recruited within four months, 60% from more disadvantaged areas. Fewer participants from the SMS+I group (64%) completed at 12 months compared to SMS only (79%) and control (83%). Compared to community recruits, men recruited via GP obesity registers were more likely to live in deprived areas (community=56% vs GP=64%), were older (community=48 years vs GP=57 years), more likely to report having a co-morbidity (44% community vs 87% GP) and had lower mean BMI (community=36.2 kg/m2 vs GP=35.0 kg/m2). Participants completing 12-month assessments differed slightly by recruitment route: community 43/60 (71%); GP 36/45 (80%). Retention was higher for participants from disadvantaged areas in the SMS+I and control groups, but not in the SMS group. Interventions were acceptable and all groups lost some weight.
**Conclusions:** Men from disadvantaged areas were engaged. Baseline characteristics and retention differed according to recruitment strategy, which has potential implications for preventative medicine trials and health inequalities.

## P-148

### Recruitment and retention of people with Dementia to a complex clinical trial: Lessons from the Journeying through Dementia trial

Ben Thomas[1], Emma Young[2]
[1]The University of Sheffield, Sheffield, United Kingdom; [2]The University of Sheffield, Sheffield, United Kingdom
*Trials* 2019, **20(Suppl 1):**P-148

**Introduction:** For results of trials to be valid and rigorous trial teams need to recruit and retain an adequate sample size to reach statistical power. Recruitment and retention of people with dementia in research is challenging due to increased frailty, morbidity, cognitive change, social isolation, the language of research, capacity issues and gatekeepers, such as family members, acting on behalf of the person with dementia. Research into complex interventions present an additional challenge for this population as there is often a requirement to commit to a structured programme.
Journeying through Dementia is a NIHR HTA funded randomised control trial investigating the clinical and cost effectiveness of a complex psychosocial intervention for individuals with early-stage dementia. The intervention aimed to improve quality of life by promoting self-management and independence. The trial recruited and randomised 480 participants from 13 sites.
**Aim:** To understand the factors impacting recruitment and retention of people with dementia in the Journeying through Dementia trial.
**Objectives:**
•To identify barriers and enablers to recruiting to, and retaining people with dementia in, a trial of a complex psychosocial intervention.
•To disseminate the lessons learnt from a large randomised control trial to improve recruitment and retention in the future.
**Methods:** A retrospective descriptive analysis of 480 participants' recruitment and retention rates along with bivariate analysis against demographic data. Additionally, semi-structured telephone interviews were conducted with a purposive sample of researchers, selected to include researchers who had experience of recruitment and retention in the trial. Interviews are analysed through thematic analysis.
**Timing of Potential Results:** The potential results will be available by the end of June 2019.
**Potential Relevance & Impact:** The results of this study aim to identify effective recruitment and retention strategies in trials of complex interventions involving people with dementia and other hard to reach groups.

## P-149

### Challenges of recruiting into a complex intervention trial (PD COMM)

Cally Rick[1], Christina Smith[2], Pui Au[3], Max Hughes[3], Sarah Tearne[3], Sylvia Dickson[4], Avril Nicholl[4], Marian Brady[4], Natalie Ives[3], Smitaa Patel[3], Rebecca Woolley[3], Patricia Masterson-Algar[5], Chris Burton[5], Carl Clarke[3,6], Cath Sackley[1,7]
[1]University Of Nottingham, Nottingham, UK; [2]University College London, London, UK; [3]University of Birmingham, Birmingham, UK; [4]Glasgow Caledonian University, Glasgow, UK; [5]University of Bangor, Bangor, UK; [6]Sandwell and West Birmingham Hospitals NHS Trust, Birmingham, UK; [7]King's College London, London, UK
*Trials* 2019, **20(Suppl 1):**P-149

**Introduction:** Recruitment into clinical trials is difficult to predict with just 56% recruiting to target (Walters et al., 2017). Barriers often increase for complex interventions and more so when they are dependent on the availability and expertise of small professional groups such as Speech and Language Therapists.
The PD COMM trial compares two types of speech and language therapy (SLT), for people with Parkinson's and communication problems, to a no intervention control in 42 sites across Britain. One intervention is an intensive therapy programme (16 one-hour sessions over 4 weeks). Sites start SLT within 4-7 weeks of randomisation. PD COMM is recruiting more slowly than predicted. Here we identify and review reasons for delays to aid future site selection.
**Methods:** A series of risks to recruitment were identified. These included: staffing levels; working patterns (full or part-time); and training /expertise of individuals in delivering Lee Sliverman Voice Treatment (LSVT). We reviewed delays in recruitment including initiating recruitment, slower recruitment than expected including planned temporary pauses in recruitment.
**Results:** Forty out of 42 open sites have recruited to the trial. 27 sites needed LSVT training for therapists, delaying the start; the average time to open was 5 months, and to first recruitment was 2 months; although three sites took 6 or more months. Of the combined 862 months open, there have been 32 pauses of at least 6 months, including 7 sites that have had 2 pauses. The most common reason reported by research nurses is lack of therapist availability due to staff changes and periods of leave. We will further explore the impact therapist numbers and working patterns have on these delays and the implications for complex interventions of this type.
**Discussion:** Recruitment has been affected by the lack of therapists' availability for delivering the intensive intervention.

## P-150

### Surgical fixation versus non-operative management for patients with stable thoracolumbar fractures: PRESTO - a feasibility study

Liz Cook[1], Elizabeth Coleman[1], Arabella Scantlebury[1], Alison Booth[1], Catherine Hewitt[1], Belen Corbacho[1], Amar Rangan[1], Joy Adamson[1], Arun Ranganathan[2], Almas Khan[3], Sashin Ahuja[4], David J Torgerson[1], Catriona McDaid[1]
[1]York Trials Unit, University of York, York, United Kingdom; [2]Bart's Health NHS Trust, London, United Kingdom; [3]Leeds Teaching Hospitals NHS Trust, Leeds, United Kingdom; [4]Cardiff & Vale University Health Board, Cardiff, United Kingdom
*Trials* 2019, **20(Suppl 1):**P-150

**Introduction:** Thoracolumbar fractures are the most common spinal fracture. Where the fracture is not obviously stable or unstable, and despite the seriousness, there is no current consensus on the best treatment. There are variations between surgeons, treating centres and within the evidence base as to whether surgical or non-surgical approaches should be used. A Cochrane review (Abudou et al., 2013) identified the need for a RCT investigating treatments for these fractures and prompted an NIHR commissioning brief.
The PRESTO study aims to determine the feasibility of undertaking a full-scale trial to evaluate cost and clinical effectiveness of surgical versus non-operative management for thoracolumbar fractures without neurological deficit.
**Methods:** The study consists of three elements: a two-arm randomised feasibility study which will explore the size of the population and completeness of follow-up; a qualitative study where both patients and staff will be interviewed to determine views and experiences of the intervention and trial processes and a national survey of surgeons to explore methods of establishing spinal stability, surgical fixation and non-operative management that are currently used in the UK. At least 60 patients will be recruited to the study via three secondary care centres, and followed-up using paper questionnaires, or data submitted online to the British Spine Registry.
**Timing of potential results:** Recruitment ended 31st March 2019, with final follow-up 3 months later. Results will be available in August 2019 and presented at the conference.
**Potential Relevance and Impact:** The study elements will be combined to give an overview of current practice in management of these fractures, the views of clinicians and patients on facilitators and barriers to a definitive trial, and feasibility of trial conduct. It will also provide information on using British Spine Registry data within a trial setting; of relevance for future trials in this population.

## P-151

### Support through Mobile Messaging and digital health Technology for Diabetes (SuMMiT-D) feasibility trial: strategies and data collection for monitoring of rapid recruitment in a primary care setting

Evgenia Riga[1], Ly, Mee Yu[1], David Judge[1], Carmelo Velardo[2], Yuan Chi[2], Andrew Farmer[1], The SuMMiT-D Collaborative Group[1]
[1]Nuffield Department of Primary Care Health Sciences, University of Oxford, Oxford, United Kingdom; [2]Department of Engineering Science, University of Oxford, Oxford, United Kingdom
*Trials* 2019, **20(Suppl 1):**P-151

**Introduction:** Feasibility studies play an important role in designing randomised controlled trials (RCT), especially in identifying and addressing challenges in primary care where rapid recruitment of large numbers of participants can be needed.
SuMMiT-D is a feasibility RCT testing the impact of SMS text-messages for behaviour change using a digital trial platform for recruitment, screening, e-consent, data collection, randomisation, intervention delivery and trial monitoring. It aims to recruit 200 patients with type 2 diabetes in three months.
With a narrow recruitment window and progression to a main trial dependent on recruiting to target, close monitoring by the trial team of set-up, and practice and patient recruitment, is needed.
**Methods:** The trial is being carried out in 16 practices in four clinical research networks.
Recruitment metrics, including set-up time, number of invitations sent, methods of identifying eligible patients and response rates were monitored weekly to assess the recruitment performance of each site. Resource use data for the study are also collected.
**Results:** A total of 8196 patients were identified across 16 practices and 7372 invitations to take part in the trial were sent out. The mean number of screened patients per practice was 20 (5% of those invited). Conversion from screening to randomisation was high (73%).
Data from the digital trial platform identified stages in the enrolment process where participants found difficulties, and these were modified in real-time during recruitment.
In total, we recruited 209 participants. The average number (range) of days from site setup to permission for recruitment was 66 (7 to 134) days and between site opening to first randomisation was 40 (12 to 80) days.
**Discussion/Conclusion:** Close monitoring and quick resolution of issues causing delays was key in quickly recruiting to target. The results collected will inform the design and recruitment plan of the subsequent main trial.

## P-152

### Recruitment and randomisation for group therapy interventions: challenges and experience from mental health trials at the Institute of Psychiatry, Psychology and Neuroscience (IoPPN)

Dominic Stringer, Laura Potts, Andrew Pickles, Kimberley Goldsmith, Sabine Landau, Ioannis Bakolis
Institute of Psychiatry, Psychology and Neuroscience, King's College London, United Kingdom
*Trials* 2019, **20(Suppl 1):**P-152

**Introduction:** A common challenge in mental health trials occurs during recruitment, where one or all the interventions involve group therapy. Typically, there is a therapeutic constraint on the minimum number of participants needed for the group therapy and it is necessary to recruit a group of participants before the intervention can start.
**Methods:** There are two approaches to randomising in this situation, by sequential randomisation, or by randomising once enough participants are recruited to ensure the minimum therapy group size ("block randomisation"). We will describe our experiences using both approaches.
**Results:** In either approach, many of the challenges arise from the need to delay the start of therapy. For sequential randomisation there is a potential delay between randomisation and start of the therapy and for block randomisation, a potential delay between baseline and randomisation. In either case, this can affect the validity of baseline measurements (which can become "outdated"), result in loss of participants during delays and lead to variability in time from baseline to commencement of therapy.
For one trial using "block randomisation" with a large block size, loss of participants prior to randomisation often resulted in a "block" never reaching the required number to be randomised. However, sequential randomisation possibly only delays this issue until after randomisation and losses post-randomisation result in missing data for the intention-to-treat analyses.
There are other design challenges. In another trial, including multiple strata within a "block" required randomising additional participants to ensure the minimum therapy group size was achieved.
**Discussion:** In our experience, "block randomisation" can be overly restrictive especially for large groups and can lead to under-recruitment. Sequential randomisation should be considered but may require flexibility in group sizes and/or relaxing the definition of the intervention (e.g. by having a "rolling" group where individuals start at different times).

## P-153

### Why do some research studies fall short of their predicted recruitment rate?

Rachel C Brierley, Maria Pufulete, Jessica Harris, Hana Tabusa, Abby Willcox, Holly Mckeon, Lucy Culliford
Clinical Trials and Evaluation Unit, Bristol Trials Centre, University of Bristol, Bristol, United Kingdom
*Trials* 2019, **20(Suppl 1):**P-153

**Introduction:** Many clinical trials fail to reach their planned sample size within the planned timeframe and budget. The predicted recruitment rate is based on both the sample size calculation and an estimated timeframe for recruitment. Various sources of information (e.g. recruitment data from previous research, clinician estimates, audit data) may be used to inform these estimates at the design stage, usually whilst applying for funding. It is unclear which sources are most reliable for estimating recruitment rates. We are conducting a survey of trial managers in the Bristol Trials Centre (BTC) to identify which information sources are used to calculate predicted recruitment rates, how often studies meet these targets and reasons why they may fall short.
**Methods:** We created a short online survey (SurveyMonkey) to collect information about (i) study design, budget and funder; (ii) sample size and planned timeframe (recruitment rate); (iii) information sources used to estimate the recruitment rate; (iv) actual recruitment rate; (v) details about why the study was/ was not on target. The survey will initially be circulated to the trial managers in the BTC (at least 60 studies) in the pilot phase. The survey respondents will be asked to answer questions about clinical research studies they manage that are in set-up, currently recruiting or have finished recruitment in the past three years.
**Timing of potential results:** We expect the results of the pilot phase of the survey in July 2019.
**Potential relevance and impact:** The results will provide evidence about information sources used for estimating recruitment rates and whether some sources provide more realistic targets than others. We plan to refine the survey, based on the pilot results, before circulating it as a national survey, via the UK Trial Managers' Network.

## P-154

### Establishing and augmenting acceptability of the Fever trial: a mixed methods feasibility study

Elizabeth Deja[1], Mark Peters[9], Imran Khan[2], Paul Mouncey[2], Rachel Agbeko[3], Blaise Fenn[4], Jason Watkins[4], Padmanabhan Ramnarayan[5], Shane Tibby[6], Kentigern Thorburn[7], Lyvonne Tume[8], Kathryn Rowan[2], Kerry Woolfall[1]
[1]North West Hub for Trials Methodology Research, University of Liverpool, Liverpool, United Kingdom; [2]Clinical Trials Unit, Intensive Care National Audit & Research Centre, London, United Kingdom; [3]Paediatric Intensive Care Unit, Great North Children's Hospital, Newcastle , United Kingdom; [4]Patient partner, United Kingdom; [5]Children's Acute Transport Service, Great Ormond Street Hospital, London, United Kingdom; [6]Guy's & St Thomas' NHS Foundation Trust, Evelina , London, United Kingdom; [7]Paediatric Intensive Care Unit, Alder Hey Children's Hospital, Liverpool, United Kingdom; [8]Faculty of Health and Applied Sciences, University of the West of England, Bristol, United Kingdom; [9]Respiratory, Critical Care and Anaesthesia Unit, UCL Great Ormond Street, London, United Kingdom
*Trials* 2019, **20(Suppl 1):**P-154

**Introduction:** Optimal fever management in critically ill children is unknown. We explored parent and practitioner views on the feasibility of a trial investigating temperature thresholds (37.5°C v 39.5°C) for the administration of paracetamol in children with fever and suspected infection.
**Methods:** 1) Pre-trial focus groups with practitioners and interviews with parents to inform the pilot trial design. 2) Embedded study within the pilot trial involving focus groups and surveys with practitioners and interviews and questionnaires with parents of randomised children. Data analysis drew on Sekhon et al's (2017) theoretical framework of acceptability.
**Results:** 1) Parents (n=25) were interviewed and practitioners (n=56) took part focus groups. Overall parents found the proposed trial acceptable. However, parents and practitioners raised concerns regarding proposed thresholds and not using paracetamol for pain or discomfort. Findings informed changes to the pilot trial protocol, participant information and site training. 2) Sixty parents of 57 randomised children took part in interviews and/or questionnaire; and practitioners (n=98) took part in either a focus group or survey. Both groups found the pilot RCT acceptable, with pre-trial research assisting practitioner 'buy-in'. However, concerns about children being in pain or discomfort when weaned from ventilation led to cases of withdrawal and protocol non-adherence. Nevertheless, n=87/100 parents provided consent and supported the trial. Practitioners had polarised views on the acceptability of the higher temperature threshold; those trained by the Fever team found it more acceptable than those trained by site colleagues.
**Discussion:** Challenges to delivering proposed trial included concerns about the acceptability of the protocol. Pre-trial research and experience of pilot trial conduct augmented views, providing insight into how challenges may be overcome; such as changing the inclusion criteria and delivery of site training. All seven constructs of the framework of acceptability would then be met. The Fever trial was deemed feasible.

## P-155

### Use of an active run-in period in a phase III cancer clinical trial: The Add-Aspirin trial experience

Fay H Cafferty, Komel Khabra, Nalinie Joharatnam, Tessa Dibble, Gemma Sancha, Alex Robbins, Ruth E Langley
MRC CTU at UCL, London, United Kingdom
*Trials* 2019, **20(Suppl 1):**P-155

**Introduction:** Run-in periods, whereby participants take trial drug(s) for a period prior to randomisation, are common in cardiovascular trials, but their use is often debated. By selecting participants for randomisation on the basis of compliance or tolerability, statistical power may be increased, but careful interpretation of results is needed. Run-ins generally have less relevance in oncology where duration of therapy may be short and/or compliance less of an issue. The Add-Aspirin trial is assessing aspirin for preventing recurrence following cancer diagnosis and treatment. Evidence suggests that aspirin must be taken for several years to see an effect and, since it is available over-the-counter, non-compliance is a concern. Additionally, tolerability is not well-documented in this setting. We evaluate use of a run-in period in the trial.
**Methods:** Add-Aspirin is a phase III, double blind, placebo-controlled, basket trial assessing daily aspirin (100mg or 300mg for 5 years) following treatment for an early stage cancer (breast, colorectal, gastro-oesophageal, prostate; total n=9920). An active run-in, whereby participants take 100mg aspirin daily for 8 weeks, was incorporated to select those most likely to tolerate and comply with the intervention.
Run-in data from a planned feasibility phase lasting 2 years will be used to evaluate use of the run-in period, considering: randomisation rates; reasons for not proceeding to randomisation; and differences (in demographics, disease, adherence and tolerability) between randomised and non-randomised participants. Feedback from recruiting teams on implementation of the run-in will also be incorporated.
**Timing of potential results:** The feasibility stage is complete (n= 3194, of which 2719 (85%) were randomised). Analyses will be finalised in Summer 2019.
**Potential Relevance & Impact:** Add-Aspirin demonstrates successful implementation of a run-in period in a late-phase cancer clinical trial. Evaluation of this design element, in terms of both statistical and practical aspects, will have relevance beyond the oncology setting.

## P-156

### Abstract withdrawn

## P-157

### Research in a Hostile Environment: Challenges of recruitment and follow-up in an Emergency Department trial

Sarah Campbell[1], Rebecca Chapman[1], Edward Carlton[2]
[1]Peninsula Clinical Trials Unit, Plymouth, United Kingdom; [2]North Bristol NHS Trust, Bristol, United Kingdom
*Trials* 2019, **20(Suppl 1):**P-157

**Introduction:** Emergency Departments (EDs) benefit from high patient numbers and a wide demographic to allow potential recruitment to clinical trials, however it is a challenging setting for research. EDs are crowded and patients may have prolonged waits to be seen. LoDED (Limit of Detection of Troponin and ECG Discharge Strategy) is a pragmatic randomised trial to assess clinical effectiveness of a novel discharge process for patients with chest pain. We describe several aspects considered in the trial to ensure recruitment to target and high-quality data.
**Methods:** We selected eight UK sites with a track-record in recruiting to ED trials and established research teams. We engaged clinical teams through bespoke teaching sessions to identify potentially eligible patients at the time of triage. To increase recruitment efficiency, pre-prepared participant packs were provided which contained necessary study documents.
Participants were given a two-page participant information sheet (PIS). Time taken for usual care laboratory processing of blood samples and time waiting to be assessed by a doctor allowed sufficient time to consider the PIS prior to being approached for consent.
Randomisation was undertaken using simple bespoke online software and efficient clinical data collection undertaken following participant discharge using electronic patient records. Follow-up of participants at 30 days was facilitated by using multiple methods of contact (texts, e-mail, post and phone).
**Timing of potential results:** In nine months 632 participants were recruited; 106.4% against a target of 594, with follow-up rates of approximately 95%. Results are anticipated in mid-2019.
**Potential Relevance and Impact:** This trial benefited from a high number of potentially eligible patients presenting with chest pain. Clinicians reported that participants were very engaged with the trial and appreciated the simple processes involved, and staff found the study easy to recruit to. This led to recruitment over target in a hostile clinical environment.

## P-158

### Does a video clip enhance recruitment into a parenting trial? Evidence from a study within a trial (SWAT)

Holly Mattock[1], Rachael Ryan[1], Christine O'Farrelly[1], Daphne Babalis[2], Paul Ramchandani[1]
[1]Centre for Psychiatry, Imperial College London, London, United Kingdom; [2]Imperial Clinical Trials Unit, London, United Kingdom
*Trials* 2019, **20(Suppl 1):**P-158

**Introduction:** Reaching recruitment targets in randomised controlled trials is a challenge. Media tools such as video clips are increasingly used to engage participants, yet there is a paucity of research into the use of video to optimise recruitment. We therefore tested whether adding a participant information clip to a standard participant information sheet following a screening phase improved recruitment into a parenting trial.
**Methods:** A study within a trial was embedded within a randomised controlled trial of a parenting intervention to improve child behaviour problems. Potential participants were randomised to receive either a standard participant information sheet (PIS) or a participant information clip (PIC) as part of an email contact following a screening phase; all participants went on to receive the PIS as part of the existing recruitment procedure.
**Results:** During the study within a trial period 107 eligible participants entered screening for the main RCT and were randomised to either the PIC condition (N=56) or to the PIS condition (N=51) on a weekly basis. The PIC condition did not increase the odds of recruitment into the trial (OR = 0.82, CI = 0.31 – 2.14, p = 0.68). Qualitative interviews indicated that participants perceived both the PIS and PIC to be useful, comprehensive and accessible, while researchers found the use of an initial email contact helpful in promoting participant interest in the study irrespective of the PIS/PIC condition.
**Discussion:** The introduction of a PIC into a parenting trial did not lead to an improvement in recruitment, however the small sample size precludes definitive inferences regarding group differences.

## P-159

### Successes and challenges of recruiting in nursing and residential care homes: Lessons learned from the ELECTRIC trial

Jo Booth, Catriona O'Dolan, Lisa Macaulay, Jacqueline Gray
Glasgow Caledonian University, Glasgow, United Kingdom
*Trials* 2019, **20(Suppl 1):**P-159

ELECTRIC is a multicentre, placebo controlled, randomised trial aiming to determine the effectiveness of non-invasive transcutaneous posterior tibial nerve stimulation (TPTNS) to treat urinary incontinence (UI) in care home residents. This paper discusses challenges and successes encountered in recruiting to the ELECTRIC trial and engaging nursing and residential care homes in research.
Two elements of recruitment are considered: i) Identifying and recruiting care homes as research sites ii) Identifying and recruiting care home residents to participate in ELECTRIC. The planned approach to recruitment of care home sites, how and why the actual strategies differed from the original plan will be described. Reflections on successes and challenges include involving the ENRICH (Enabling Research In Care Homes) network, fostering group partnerships, care home manager meetings, targeted recruitment and snowballing methods of introduction. The relative success of strategies specifically designed to foster enthusiasm and early uptake of the opportunity to engage with the trial will be discussed.
The crucial roles of the care home manager, delegated ELECTRIC lead and care home staff are highlighted for resident recruitment and the influence of organisational and individual care home culture explored. Lessons learned during the early stages of ELECTRIC trial recruitment and applied to later recruitment and engagement activities will be described. Details of resident recruitment and randomisation rates will be provided for i) Overall care home size and number of residents; ii) number of eligible residents iii) number of eligible residents for whom consent is available.
**Conclusion:** Recruitment to trials from nursing and residential care homes presents a number of context-specific challenges and opportunities for success that are not routinely encountered when recruiting through NHS services. Exploring the influencing factors, based on experiences of recruiting from forty care homes, may expedite the application of successful recruitment strategies in such research contexts in the future.

## P-160

### PROMoting THE USE of SWATs (PROMETHEUS): Routinely embedding recruitment and retention interventions within randomised trials

Adwoa Parker[1], Catherine Arundel[1], David Beard[2], Peter Bower[3], Paul Brocklehurst[5], Elizabeth Coleman[1], Cindy Cooper[4], Lucy Culliford[4], Declan Devane[7], Sandra Eldridge[8], Richard Emsley[9], Sandra Galvin[7], Catherine Hewitt[1], Alan Montgomery[10], Chris Sutton[3], Shaun Treweek[11], David Torgerson[1]
[1]The University Of York, United Kingdom; [2]University of Oxford, Oxford, United Kingdom; [3]University of Manchester, Manchester, United Kingdom; [4]The University of Sheffield, Sheffield, United Kingdom; [5]Bangor University, Bangor, United Kingdom; [6]University of Bristol, Bristol, United Kingdom; [7]National University of Ireland Galway, Galway, Republic of Ireland; [8]Queen Mary University of London, London, United Kingdom; [9]King's College London, London, United Kingdom; [10]The University of Nottingham, Nottingham, United Kingdom; [11]University of Aberdeen, Aberdeen, United Kingdom
*Trials* 2019, **20(Suppl 1):**P-160

**Introduction:** Recruitment and retention of participants are the biggest challenges to successful delivery of trials. Many interventions are used by trial teams to improve recruitment and retention; however, few have been rigorously evaluated. A Study Within A Trial (SWAT) is a robust method to evaluate the effectiveness of interventions for improving trial conduct. PROMoting THE USE of SWATs (PROMETHEUS) aims to make embedding SWATs standard practice across UK Clinical Trials Units (CTUs), by pump-priming and facilitating trial teams to start at least 25 SWATS of recruitment or retention.
**Methods:** We established a network of CTUs committed to starting at least two SWATs of recruitment and/or retention interventions. We identified promising recruitment and retention interventions from a variety of sources including Cochrane systematic reviews and existing prioritisation exercises. We created a priority list of 7 recruitment and 8 retention interventions, and developed template SWAT protocols for testing them. We are inviting trial teams to apply for funding of up to £5,000 to test one of our prioritised interventions or their own. Successful applicants are given funding, methodological and process support to embed and report the SWAT.
**Results:** 26 trial teams from 11 CTUs have been funded to undertake 30 SWATs of recruitment and retention strategies, exceeding our initial target of 25 SWATs ahead of schedule. Each recruitment and retention intervention is being evaluated in up to five host trials, and will be evaluated for its effectiveness in the context of individual trials, as well as across different trial populations and contexts.
**Discussion:** The RCT community has shown that with enough financial and methodological support, many are willing to engage with and implement SWATs to build rapidly the evidence base. This will help to deliver trials in a timely manner, patients to receive better treatments and funders to deliver on their objectives.

## P-161

### What use is an external pilot study?

Sandra Eldridge[1], Christine Bond[2], Mike Campbell[3], Claire Chan[1], Sally Hopewell[4], Gill Lancaster[5], Lehana Thabane[6]
[1]Queen Mary University of London, United Kingdom; [2]University of Aberdeen, Aberdeen, United Kingdom; [3]University of Sheffield, Sheffield, United Kingdom; [4]University of Oxford, Oxford, United Kingdom; [5]Keele University, Keele, United Kingdom; [6]McMaster University, Hamilton, Canada
*Trials* 2019, **20(Suppl 1):**P-161

The past 15 years have seen an exponential rise in published studies in health research described as pilot or feasibility studies. Many of these published studies are in preparation for larger randomised controlled trials evaluating efficacy or effectiveness. The vast majority of these studies are external pilot or feasibility studies conducted separately from the future larger randomised trial, andthe data they produce is used only to make decisions about whether and how to go on to a larger study. However, there has also been a rise in the number of effectiveness or efficacy randomised controlled trials in which the first part of the trial is a pilot phase used to test out the feasibility of trial processes such as recruitment and retention. These pilot phases are usually called internal pilot studies.
A pilot or feasibility phase for trials of complex interventions is widely recommended, for example by the UK MRC framework for the development and evaluation of complex interventions and is expected by funders such as the UK NIHR. However, researchers still face the question about whether and what sort of external pilot work is needed in relation to their own research area. In this talk, we will use some examples of external pilot and feasibility studies to reflect on when external pilot studies are particularly useful, and how to make judgements about their objectives, design and conduct. The examples cover a range of different health issues. We suggest that the usefulness of an external pilot study in advance of a larger randomised controlled trial may be best assessed on a case by case basis.

## P-162

### Recruiting women during pregnancy and childbirth to clinical trials – the barriers and enablers of trial recruiters: a qualitative evidence synthesis

Vivienne Hanrahan[1], Linda Biesty[2], Katie Gillies[3]
[1]National University of Ireland Galway, Galway, Ireland; [2]National University of Ireland Galway, Galway, Ireland; [3]University of Aberdeen, Aberdeen, Scotland
*Trials* 2019, **20(Suppl 1):**P-162

**Introduction:** The Prioritising Recruitment in Randomised Trials Priority Setting Partnership Study (PRioRiTy PSP), identified and prioritised unanswered questions around trial recruitment research. We utilised qualitative research methods to answer Question 5 'What are the barriers and enablers for trial recruiters?' within the maternity care setting.
The aim of this Qualitative Evidence Synthesis (QES) was to explore the evidence on the recruiter's experience and perceptions of recruiting women during pregnancy & childbirth to trials. We were specifically interested in exploring;
1)The recruiter's perception and awareness of how their own role (e.g. clinical or non-clinical) might influence recruitment.
2)The recruiter's perception and experience of how the 'type of trial' (i.e. pharmaceutical, non-pharmaceutical,) might influence recruitment.
3)Explore the setting and environment in which recruitment is undertaken.
**Methods:** Using SPIDER, a broad search of electronic databases (Pubmed, CINAHL, Embase, PsycINFO) & grey literature (Scopus, forward & backward citation searches) returned 13,401 citations. Abstracts were independently screened by two reviewers, of these, 29 citations progressed to full text screening, resulting in 8 eligible papers. We designed a data extraction tool and critically appraised using CASP checklist. A thematical approach to coding & synthesis was undertaken, applying CERQual for confidence in review findings.
**Timing of Potential Results:** We have preliminary results and expect the QES will be submitted for publication in December 2019.
**Potential Relevance & Impact:** The review will, for the first time, systematically synthesise existing research on factors associated with recruitment to RCTs in maternity care from the recruiters perspective. The findings will provide the basis and direction of an exploratory qualitative study seeking to develop a statement of recommendation (in collaboration with stakeholders) for successful recruitment of women during pregnancy & childbirth to RCTs.

## P-163

### Understanding and addressing recruitment challenges in a thoracic anaesthesia randomised controlled trial (RCT)

Caroline Wilson[1], Marcus Jepson[1], Fang Gao-Smith[2]
[1]Bristol Medical School, University of Bristol, Bristol, United Kingdom; [2]Institute of Inflammation and Ageing Centre of Translational Inflammation Research, University of Birmingham, Birmingham, United Kingdom
*Trials* 2019, **20(Suppl 1):**P-163

**Introduction;** The Topic 2 randomised controlled trial (RCT) (NIHR-HTA- 16/111/111) was set up to compare the effectiveness of thoracic epidural and paravertebral blockade in reducing chronic post-thoractomy pain. Recruitment was anticipated to be difficult and the QuinteT Recruitment Intervention (QRI) was integrated into the trial design to optimise recruitment.

**Methods:** In phase 1 of the QRI, generic tips and training were provided to assist recruitment from the outset. In-depth semi-structured interviews were conducted with healthcare professionals, TMG members and sites declining trial participation. Monthly screening logs were scruitinised and recruitment consultations and interviews analysed, using thematic and constant comparative methods. In phase 2, we provided confidential and supportive feedback to recruiters and collaboratively developed and implemented plans to optimise recruitment.
**Results**: (Recruitment and qualitative data collection is ongoing).
We conducted 12 interviews with surgeons, anaesthetists and research nurses, audio-recorded 9 recruitment consulations and carried out individual and group feedback sessions. Randomisation rates varied between 43 to 100 per cent (randomised/ eligible patients approached). Specific training was given to convey equipoise, to gently explore patient preferences and balance the description of the treatment arms. Despite centres exceeding recruitment targets, accrual may lag due to problems opening the target number of recruitment centres, owing to a sharp decline in the number of planned thoracotomies, a shift away from thoracic epidurals and limited capacity to deliver high-dependency unit nursing care.
**Potential relevance and impact**
This is the first anaesthesia RCT to embed qualitative methods to optimise recruitment. Findings may be of interest to trialists initiating RCTs when community equipoise challenges clinician preferences for routine local practice.

## P-164

### Recruitment strategies and screening yields in the Hypertension Approaches in the Elderly: a Lifestyle Study (The HAEL Study)

Daniel Umpierre, Lucas P. Santos, Cíntia Botton
Universidade Federal Do Rio Grande Do Sul, Porto Alegre, Brazil
*Trials* 2019, **20(Suppl 1):**P-164

**Introduction**: The identification and recruitment of research participants for clinical trials is a common challenge across studies, being considered a key determinant for adequate trial completion. Since several recruitment strategies may be implemented to enhance recruitment rates, we aimed to describe the recruitment strategies and preliminary results for the Hypertension Approaches in the Elderly: a Lifestyle Study (HAEL).
**Methods:** The HAEL Study is a 12-week randomised controlled trial (NCT03264443) that aims to assess blood pressure effects of a pragmatic combined training program (1:1 allocation ration) in comparison with a health education program. The sample size will be composed by 184 older adults, divided in two implementation centers. In the two sites, recruitment strategies include five main sources, as follows: press media, word-by-mouth, lists generated by electronic health records, professional referrals, and flyers. Descriptive statistics are used to monitor characteristics of study participants, distribution of sex across sites. Data are expressed as absolute and relative frequencies.
**Results:** From September/2017 to April/2019, four recruitment waves have been conducted in both, totalizing 289 and 200 monitored screening calls in coordination and field sites, respectively. The screening yields were consistent across site for word-by-mouth (62 [21.4%] of total screenings), flyers (8 [1.6%]), and professional referrals (7 [1.4%]). However, important discrepancy has been observed across sites for press media, which is the source of 154 (53.3%) screenings at the coordination site, and lists generated by electronic health records, which is the source of 104 (52.0%) screening at the field site.
**Discussion:** The observed discrepancies in recruitment strategies have yielded differences in distribution of women and men in different sites, suggesting that active monitoring of recruitment yields from different sources might be useful to avoid non-random distortions in screened and included subjects.

## P-165

### What proportion of ethically approved randomised clinical trials can be found in a trial registry?

Benjamin Speich[1,2], Dmitry Gryaznov[2], Viktoria GLoy[2], Kimberly A. Mc Cord[2], Arnav Agarwal[3], Benjamin Kasenda[2], Matthias Briel[2]
[1]Centre for Statistics in Medicine, Nuffield Department of Orthopaedics, Rheumatology and Musculoskeletal Sciences, University of Oxford, United Kingdom, Oxford, United Kingdom; [2]Basel Institute for Clinical Epidemiology and Biostatistics, Department of Clinical Research, University Hospital Basel, University Basel CH-4031 Basel, Switzerland, Basel, Switzerland; [3]Department of Health Research Methods, Evidence, and Impact, McMaster University, Hamilton, Ontario, Canada, Hamilton, Canada
*Trials* 2019, **20(Suppl 1):**P-165

**Background:** Randomised clinical trials (RCTs) provide the most trustworthy evidence when evaluating a medical intervention. However, it can be difficult to appraise the true effect of an intervention due to publication bias, i.e. the frequent non-publication of studies with unfavourable results. Clinical trial registries are supposed to give a comprehensive overview of all ongoing RCTs which helps to estimate and control publication bias and to avoid duplication of research. There is little evidence on what proportion of approved RCTs (published and unpublished) was actually registered. This study aimed to close this knowledge gap.
**Methods:** We had access to a total of 555 RCT protocols that were approved by a research ethics committee in 2012 or 2016 in Switzerland, Canada or Germany. For each RCT we systematically searched if it was registered in a clinical trial registry, and if it was registered before patient enrolment. We present results separately for 2012 and 2016 to assess if there was an improvement over time. In addition, we stratified the analysis by industry and non-industry sponsored RCTs.
**Results:** From the 555 RCTs, 491 (88%) were registered and 447 (81%) were prospectively registered. We did not find an increase in registrations over time (2012: 91% registered, 81% prospectively registered; n=262; 2016: 88% registered, 81% prospectively registered; n=293). Industry trials seemed to be more often registered (96%; 257 of 269) and prospectively registered (90%; 241 of 269) compared to non-industry RCTs (82% registered; 234 of 286; and 72% prospectively registered; 206 of 286). Data collection on publication status of RCTs approved in 2012 is ongoing and will be present at the conference.
**Conclusion:** Registration of RCTs is still incomplete, especially for non-industry RCTs. Our study will provide a first estimate of the proportion of unpublished RCTs that can be found in a registry.

## P-166

### Establishing minimum sample size requirements for stroke rehabilitation randomised controlled trials (RCTs) using the Barthel Index (BI) or modified Rankin Scale (mRS) as outcome measures

Kris McGill[1,2], Jon Godwin[3], Cath Sackley[1], David Gavaghan[4], Marian C Brady[2]
[1]King's College London, London, United Kingdom; [2]NMAHP Research Unit, Glasgow Caledonian University, Glasgow, Scotland; [3]Glasgow Caledonian University, Glasgow, Scotland; [4]University of Oxford, Oxford, United Kingdom
*Trials* 2019, **20(Suppl 1):**P-166

**Introduction:** Underpowered trials risk contributing to research waste through the production of inaccurate results. Stroke rehabilitation RCTs can experience recruitment challenges and limited sample sizes. Simulations have been used successfully in other fields to explore sample size adequacy and provide recommendations for future RCTs recruitment targets.
**Aim:** To examine the adequacy of stroke rehabilitation RCT sample sizes in the context of BI or mRS.

**Method:** We secured 2,350 anonymised individual participant data (IPD) on the BI or mRS from 18 stroke rehabilitation RCTs archived within VISTA-Rehab (www.virtualtrialsarchives.org/vista-rehab). Computer simulations were conducted using typical experimental event rates (EER) and control event rates (CER) for both outcome measures in order to determine appropriate sample size boundaries. Event rates were defined as clinically relevant improvements (BI 1.85; mRS 1). We examined numbers needed to treat (NNT), estimated how accurate these NNTs were for differing sample sizes, and estimated the RCT sample sizes required in order to achieve an NNT within clinically acceptable boundaries (+/- 1 of the true NNT value).
**Result:** For a 75% chance of a stroke rehabilitation RCT being able to accurately determine statistical advantage over control when using the BI (assuming a CER of 0.36 and EER of 0.43) 702 participants per group would be required and for accurate interpretation of effect sizes 1000s would be required. Simulations were not possible for the mRS as there was a higher CER (0.13) than EER (0.12).
**Discussion**: The BI when used as an outcome measure for stroke rehabilitation RCTs requires unfeasible sample sizes for effect size interpretation. The mRS appears to lack the sensitivity to detect change and is therefore unlikely to be a useful outcome measure for stroke rehabilitation. Therefore, the current use of these outcome measures for stroke rehabilitation RCTs is contributing to research waste.

## P-167

### Incorporating estimated correlation between baseline and follow-up measurements into sample size calculations in randomised trials: Efficient design or type 2 error risk?

Charlie Welch, Lydia Flett, David Torgerson
York Trials Unit, United Kingdom
*Trials* 2019, **20(Suppl 1):**P-167

In**troduction**: Adjusting for baseline measurements in the analysis of randomised trials increases efficiency, but this is often neglected from sample size calculations. We aim to explore the utility of incorporating estimated correlation between baseline and follow up measurements into sample size calculations.
**Methods:** Using data from trials coordinated by York Trials Unit (YTU), we obtained estimates of the correlation between baseline and follow up for three outcomes; body weight, Patient Health Questionnaire (PHQ-9) score and Hospital Anxiety and Depression Scale (HADS) score. We identified trials funded by the Health Technology Assessment (HTA) programme using one of these measures as a primary outcome and featuring a sample size calculation that did not account for repeated measurements. This yielded five trials using body weight, two using PHQ-9 and one using HADS.
**Timing of Results:** YTU data suggests correlation between baseline and follow up of around 0.9, 0.4 and 0.7 for body weight, PHQ-9 and HADS respectively. Using these values in a power calculation would reduce the sample size by 81% for body weight, 16% for PHQ-9 and 49% for HADS. For the five HTA trials using body weight, failure to account for correlation between repeated measurements in their sample size calculations may have led to unnecessarily large trials, increasing the chance of finding statistically significant differences, which are not clinically significant, while also being potentially inefficient. Of these five trials, two found statistically significant differences, although the point estimate was not clinically significant in one of these.
**Potential Impact:** Trials using outcomes with substantial correlation between repeated measurements, such as body weight, could be made more efficient if they incorporated this information into their sample size calculation. However, trials planning to use outcomes, such as the PHQ-9, with relatively poor correlation between repeated measurements, might consider using appropriate alternatives to improve efficiency.

## P-168

### Using data from a systematic review to establish whether a core outcome set is required for studies assessing the effectiveness of interventions to manage non-respiratory sleep disturbances in children with neurodisabilities

Catriona McDaid[1], Adwoa Parker[1], Arabella Scantlebury[1], Caroline Fairhurst[1], Vicky Dawson[3], Heather Elphick[4], Catherine Hewitt[1], Gemma Spiers[2], Megan Thomas[5], Bryony Beresford[1]
[1]University Of York, York, United Kingdom; [2]Newcastle University, Newcastle Upon-Tyne, United Kingdom; [3]The Children's Sleep Charity, Doncaster, United Kingdom; [4]Sheffield Children's NHS Foundation Trust, Sheffield, United Kingdom; [5]Blackpool Teaching Hospitals NHS Foundation Trust, Blackpool, United Kingdom
*Trials* 2019, **20(Suppl 1):**P-168

**Introduction:** One of the challenges we faced while undertaking a systematic review assessing the effectiveness of interventions to manage non-respiratory sleep disturbances in children with neurodisabilities was the diversity of outcomes assessed across included studies. We therefore undertook further analysis of the extracted data to assess whether a core outcome set is required for studies evaluating interventions for this population.
**Methods:**
We undertook a survey of outcome measures used in primary studies identified by the systematic review which searched seventeen electronic databases and other sources including ASSIA, CENTRAL, MEDLINE, and PsycINFO. Studies evaluating pharmacological or non-pharmacological interventions for children with a neurodisability and experiencing non-respiratory sleep disturbance were included. Outcomes were listed from each study and categorised into core outcome areas and domains.
**Results:** Five core outcome areas were identified across 39 studies: child sleep, other child outcomes, parent outcomes, adverse events and process measures. 54 different measures of child sleep were used (across the domains of global assessment of sleep; sleep initiation; maintenance; scheduling; and other). Total sleep time was most commonly used in pharmacological studies (92%) and parent-reported Child Sleep Habits Questionnaire (58%) in non-pharmacological studies. Fifteen non-pharmacological (58%) and four pharmacological studies (31%) reported child outcomes other than sleep across the domains of child behaviour, quality of life, ADHD symptoms, cognition, school-related, and other. 14 non-pharmacological (54%) studies reported parent outcomes (17 different measures) compared to one pharmacological study. Most melatonin studies (85%) recorded adverse events compared to one non-pharmacological study. Process measures, related to adherence, feasibility of delivery, acceptability of the intervention were used.
**Discussion:** There was a lack of consistency between studies in the outcome measures used. A minimum core outcome set, with international consensus, should be developed in consultation with parents, children and young people, and those involved in supporting families.

## P-169

**Abstract omitted**

## P-170

**Abstract omitted**

## P-171

### The impact of UK cancer trials: Lessons from the 2014 Research Excellence Framework Assessment (REF)

Catherine Hanna[1], Lauren Gatting[2], Rob Jones[1], Katie Robb[2]
[1]CRUK CTU, University of Glasgow, Glasgow, United Kingdom; [2]Institute of Health and Wellbeing, University of Glasgow, Glasgow, United Kingdom
*Trials* 2019, **20(Suppl 1):**P-171

**Introduction:** Millions of pounds are spent annually on cancer research [1] and many thousands of patients participate in this research. Clinical trials are a significant component of these investments making it important that cancer trials have real life impacts for patients and wider society. Our aim is to improve the impact of cancer trials in the UK by better understanding the impact of current trials, and identifying how future trials can have greater impact.
**Methods:** In 2014, higher education institutions submitted case studies outlining the wider impact of their research to the UK government in a funding allocation exercise (REF2014). After identifying case studies that focused on cancer trials, a content analysis was performed to understand the types of impact described and methods used to evidence them.
**Results:** In total, 46 case studies were identified, mentioning 106 individual trials. The majority were phase III (89%) and those investigating breast cancer were most common (35%). The most widely described benefits were on policy (93% of case studies), specifically national guidelines, and on the health sector (87%), for example, implementation of infrastructure, shorter waiting times and clinical practice change. Impact on individuals' health was less common (67%) and was usually predicted rather than evidenced through measured outcomes. Finally, half of the case studies mentioned economic impact.
Methods used to demonstrate impact included expert testimony, policy citation, surveys and interrogation of patient or population level data. There was minimal documentation of researchers making active attempts to improve impact.
**Discussion:** A content analysis of case studies submitted to REF2014 identified limitations in describing and evidencing of impact for cancer trials. We suggest that trialists consider the wider impact of their research early and make active efforts to evaluate and maximise a broad range of impacts, not only those that are easy to measure.

## P-172

### Are feasibility studies fairly funded? A review of studies conducted in a UKCRC registered Trials Unit

Cassandra Lucy Brookes[1], Nishal Bhupendra Jaicim[1], Ana Suazo di Paola[1], Rachel Hobson[1], Shaun Barber[1,2], Gavin Murphy[1,3]
[1]Leicester Clinical Trials Unit, University of Leicester, Leicester, United Kingdom; [2]NIHR Research Design Service for the East Midlands, Department of Health Sciences, University of Leicester, Leicester, UK; [3]Department of Cardiovascular Sciences, University of Leicester, Leicester, UK
*Trials* 2019, **20(Suppl 1):**P-172

**Introduction:** Feasibility studies are described by the National Institute for Health Research (NIHR) as a means of testing if a trial 'can be done' before investing in the full trial [1]. Feasibility studies typically aim to describe parameters useful to planning future studies such as estimating measures of variability and characteristics of outcome measures, availability and willingness of participants or clinicians and likelihood of success and completion of trial procedures and follow-up. Feasibility studies have gained popularity in recent years as they promise to minimise uncertainties surrounding the conduct of a full trial whilst minimising resources and duration thereby reducing risk to funders. However, it isn't clear if feasibility studies are able to successfully deliver within these constraints.
**Aim:** To assess the question 'Can feasibility studies successfully deliver the research questions within the constraints of the funding envelope awarded to conduct them'.
**Objectives:**
1.Identify and describe research studies conducted within LCTU as either feasibility or full (phase II or III);
2.Assess the relative impact of conducting studies in terms of design/preparation, conduct, and outcomes/deliverables to LCTU;
3.Assess the implications for the design of future feasibility trials.
**Setting:** Trials conducted via LCTU since 2013.
**Methods:** RCTs described as either feasibility or full studies with LCTU statistical support will undergo review of grant application, protocol and end of trial report. Parameters associated with demonstrating a trial's resource use will be extracted and findings summarised by trial type.
**Timing of results and potential impact:** The review of 28 trials is currently underway and the results will be presented. The review will explore if feasibility studies currently being undertaken by the LCTU are successfully delivering outcomes within the planned constraints of the awarded funding and any impact this has on the trials unit.

## P-173

### Methodological aspects of the ISDR RCT: Investigating individualised variable interval screening for diabetic retinopathy in a screening population

Christopher Paul Cheyne[1], Deborah M Broadbent[1,2], Tracy Moitt[1], Lolade Howard[1], Sue Howlin[1], Kate Silvera[1], Duncan Appelbe[3], Mehrdad M Rahni[1], Irene M Stratton[4], Marilyn James[5], Amu Wang[1], Paula R Williamson[1], Simon P Harding[1,2], Marta García-Fiñana[1]
[1]University Of Liverpool, Liverpool, United Kingdom; [2]Royal Liverpool University Hospital, Liverpool, United Kingdom; [3]University of Oxford, Oxford, United Kingdom; [4]Gloucestershire Retinal Research Group, Cheltenham, United Kingdom; [5]University of Nottingham, Nottingham, United Kingdom
*Trials* 2019, **20(Suppl 1):**P-173

**Introduction:** Diabetic retinopathy (DR) is one of the major causes of blindness worldwide. National systematic screening programmes for sight threatening DR (STDR) are established in several countries with many involving annual screening intervals. Extending the interval for people at low risk is expected to reduce the economic burden of annual screening on health systems without compromising efficacy. A randomised controlled trial was conducted to assess the safety, efficacy and cost-effectiveness of individualised variable interval risk-based screening for DR in Liverpool.
**Methods:** This RCT required data from multiple sources. Demographic and clinical (primary care) data were obtained from GPs across Liverpool (EMIS) and retinopathy data came from an externally held database (OptoMize). Randomisation was 1:1 to either annual or individualised screening (6, 12, 24 months). Screening interval allocations for the individualised arm were calculated using a risk engine. The primary analysis focuses on equivalence in attendance at 1st follow-up between the two arms. Development of STDR within 24 months (secondary outcome) was analysed across the arms using a non-inferiority approach.
**Timing of Potential Results:** The trial has now been completed. Results of the main analyses (attendance at 1st follow-up, STDR within 24 months) are expected to be available in June 2019. These will be presented along with a description of methodology and processes required throughout the data collection period.

**Potential Relevance & Impact:** This is the first RCT to study the effect of an individualised risk based approach to varying screening intervals for diabetic retinopathy. As a large trial with data obtained from multiple sources, our experience may be helpful for the design and conduct of similar trials in the future.

This abstract presents independent research funded by the NIHR UK (RP-PG-1210-12016). The views expressed are those of the authors, not those of the NHS, NIHR or Department of Health.

## P-174

### Under-representation in Clinical Trials: Participants with rare diseases, reporting and awareness

Helen McAneney
Centre for Public Health, Queen's University Belfast, Belfast, United Kingdom
*Trials* 2019, **20(Suppl 1):**P-174

**Introduction:** Trial populations need to reflect those in the community who may benefit from the treatment being tested if the results of the trial are to maximise patient health gains.

The European Union defines a rare disease as one affecting fewer than 5 in 10,000 of the general population. With over 8,000 known rare diseases, almost 6% of the population will be affected by a rare disease. This equates to approximately 3.5 million people in the UK and 30 million people across Europe.

Consequently, one would expect 6% of those recruited to clinical trials to have a rare disease; however this is at best unknown and likely to not be the case. This study will investigate the representation of rare disease participants within clinical trials and possible reasons for under-representation of these participants, including (i) lack of reporting that a participant has a rare disease, (ii) potential exclusion due to the recruitment criteria to the clinical trial and/or (iii) lack of awareness of rare diseases.

**Methods:** A sample of clinical trials for anti-hypertension medications, listed on ClinicalTrials.gov, will be investigated. Representation of participants will be considered by analysing the reporting of participants' rare disease and the inclusion/exclusion criteria from protocols, reports and publications of the clinical trials. Awareness of rare diseases will be explored through a network analysis of the citation of rare disease literature using CitNetExplorer, specifically citation of the European Union definition of a rare disease.

**Timing of Potential Results:** Expected August 2019.

**Potential relevance and impact:** The aim of the UK Strategy for Rare Disease is to 'ensure no one gets left behind just because they have a rare disease'. Clinical trials are pivotal to the improvement of patient health, and require representation of all patients, including and inclusion of rare disease patients in clinical trials.

## P-175

### Participant and site-level factors associated with missing data in palliative care trials: an individual participant level data analysis

Jamilla Akhter Hussain[1], David C Currow[2], Miriam J Johnson[1], Martin Bland[3], Prof Ian R White[4]
[1]Wolfson Palliative Care Research Centre, Hull, United Kingdom; [2]IMPACCT, Sydney, Australia; [3]University of York, York, United Kingdom; [4]MRC Clinical Trials Unit at UCL, London, United Kingdom
*Trials* 2019, **20(Suppl 1):**P-175

**Introduction:** Missing data (MD) can compromise the power, precision, generalisability and validity of trial findings. To minimise the impact of MD, it is essential that MD are reduced as much as possible. Despite this, very little evidence has been developed on how to reduce MD. To ensure effective interventions to reduce MD are developed and evaluated it is important that these are based on evidence and theory. Assessment of factors that are associated with missingness will help to inform the design of such interventions.

**Methods:** Individual participant-level data from 10 phase 3 palliative care trials were used to assess the association between participant and site-level factors and MD. Multi-level cross-classified models were developed. Missing values for the participant and site-level factors were handled according to the most plausible assumptions about the mechanism of covariate MD and compared in MD sensitivity analyses.

**Results:** Participants with MD at the previous time-point and poorer performance status were more likely to have MD for the primary outcome and quality of life (QoL) outcome, at both the primary follow-up point and end of follow-up. At the end of follow-up, sites who randomised more participants and those with two research personnel (compared to 1) were more likely to have MD. Sites with four research personnel were significantly less likely to have MD. Trial duration and the number of research personnel explained most of the variance at the trial and site level respectively.

**Discussion:** Participants with poorer performance status and those with previous MD are at high risk of MD in palliative care trials and should be identified early and provided with further support to enable the provision of complete data. Site-level factors should also be addressed to reduce MD at the end of follow-up.

## P-176

### Improving engagement with trial participant newsletters using theory-based enhancements: results of a randomised Study Within A Trial (SWAT)

Karen Bracken[1], Camille E Short[3], Andrew Vincent[3], Gary Wittert[2]
[1]NHMRC Clinical Trials Centre, University of Sydney, Sydney, Australia; [2]Freemasons Foundation Centre for Men's Health, Adelaide Medical School, University of Adelaide, Adelaide, Australia; [3]SAHMRI, University of Adelaide, Adelaide, Australia
*Trials* 2019, **20(Suppl 1):**P-176

**Introduction:** Participant newsletters are commonly used to maintain participant engagement and boost retention in clinical trials. However, there is little evidence to guide this trial activity, and an approach based on theories of human behaviour could improve newsletter engagement. We conducted a study within a trial (SWAT #91), nested within the T4DM diabetes prevention trial, to evaluate the impact of theory-based enhancements to an emailed participant newsletter on participants' engagement with the newsletter.

**Methods:** T4DM participants are Australian men aged 50-74 years. All participants (on-treatment and completed treatment) receive quarterly email newsletters from the central coordinating centre. In this evaluation, participants were randomised to receive a one-off email newsletter with one of eight possible variations using a 2x2x2 factorial design. Factors were subject line (standard wording versus enhanced competency-based wording), sender (trial name versus site nurse's name), and salutation line (no salutation versus personalised salutation). Enhancements were designed to influence competence and relatedness, based on self-determination theory. The primary outcome was email open rate. Analysis was by logistic mixed-effects regression modelling. Three-way factorial interaction was tested, and backward stepwise elimination performed to identify the optimal model.

**Results;** Of participants (n=931) randomised to an email option, 511 (55%) opened the email. After backwards variable elimination, the optimal model included sender and subject line only (OR=1.8, 95%CI=[1.1, 3.0], p=0.02), but not salutation. Participants who received an email with an enhanced, competency-based subject line sent under their site nurse's name were more likely to open the email compared to those who received the other combinations (approximately 10% higher absolute open rate).

**Discussion:** Using a theory-based approach, combining enhanced subject line and nurse's sender name, engagement with an emailed participant newsletter was modestly increased. Although requiring replication, we have demonstrated the potential of simple, theory-based adaptations to enhance participant engagement.

## P-177

### Development of participant-centred interventions to enhance retention in randomised controlled trials: a theory-informed study

Rumana Newlands[1], Shaun Treweek[1], Justin Presseau[2], Eilidh Duncan[1], Peter Bower[3], Jim Elliott[4], Margaret Ogden[1], Mary Wells[5], Miles Witham[6], Bridget Young[7], Graeme MacLennan[1], Jill Frances[8], Katie Gillies[1]
[1]Health Services Research Unit, University Of Aberdeen, Aberdeen, United Kingdom; [2]Clinical Epidemiology Program Ottawa Hospital Research Institute, Ottawa, Canada; [3]Division of Population Health, Health Services Research & Primary Care (L5), University of Manchester, Manchester, United Kingdom; [4]Health Research Authority, London, United Kingdom; [5]Imperial College HealthCare NHS Trust, London, United Kingdom; [6]Newcastle University , Newcastle, United Kingdom; [7]University of Liverpool, Liverpool, United Kingdom; [8]City University of London, London, United Kingdom
*Trials* 2019, **20(Suppl 1):**P-177

**Introduction:** Participant drop-out affects the credibility of findings from randomised controlled trial (RCT) and reduces their potential to influence clinical practice. Several aspects of trial retention can be thought of as a behaviour e.g. returning a questionnaire or attending a clinic. Most existing retention interventions have no theoretical basis, do not explicitly target behaviour change, and have little evidence to support acceptance by participants. Our research develops theoretically informed, participant-centred, behaviour change interventions to improve retention in RCTs. This presentation will introduce the potential benefits of using insights from behavioural science (specifically the Theoretical Domains Framework (TDF) and behaviour change techniques (BCTs)) to consider how to improve retention in RCTs.
**Methods:** Sixteen telephone interviews were conducted using a TDF-based topic guide with participants who had either failed to return a follow-up questionnaire and/or attend a follow up clinic. Participants were invited from a range of RCTs with poor retention identified from clinical trial unit portfolios, UK. Theory based content analysis was conducted. Domains were prioritised based on frequency and content and then mapped onto BCTs. Crucially, retention specific interventions were co-designed with trial participants using these BCTs.
**Timing of Potential Results and Potential Relevance and Impact:** We will know the form (i.e. content, mode, timing) of our interventions in September 2019. This is one of the first studies to apply a theoretical lens to the development of participant-centred interventions to improve trial retention. These findings provide a new methodology to develop interventions in clinical research trials to target participant retention using a behaviourally focussed approach that can be applied across trials in different contexts and ultimately lead to more reproducible, participant centred, interventions.

## P-178

### Abstract withdrawn

## P-179

### Moving to direct electronic capture of patient-reported data: lessons from the UKSTAR trial

Susan Wagland, Juul Achten, Matthew Costa
NDORMS, University of Oxford, Oxford, United Kingdom
*Trials* 2019, **20(Suppl 1):**P-179

**Introduction:** Aims: It is sometimes assumed that collecting data electronically is less staff resource-intensive than collection on paper, and that follow-up will be more complete. Evidence to support this is lacking. Our aims were to assess the impact of switching from data collection via postal paper questionnaires (paper-data) to electronic data collection (e-data) on follow-up rates.
Objectives: To report on the effectiveness of e-data compared to paper-data.
Setting: In the UKSTAR trial, we switched data collection from paper-data to e-data during follow-up. UKSTAR is an RCT comparing treatments for patients with Achilles tendon rupture.
**Methods:** 540 adult participants were invited to complete questionnaires 3, 6 and 9 months post-randomisation. Questionnaires were sent by post, during the first 15 months of follow-up. Data collection then switched to e-data for a further 13 months, with invitations sent via email and/or text. Relevant data collected for this sub-study included: patient demographics and follow-up rates.
**Results:** 1577 invitations were sent (732 postal, 845 electronic). Response rate after the initial invite was lower at all time-points with e-data than postal invitations (combined over time 59% vs 81% respectively). Both sexes had higher response rates to paper-data than to e-data (men 79% vs 56%; women 89% vs 70% respectively). Participants aged 55 and over responded better to a first invite by post than by edata (82% vs 60% respectively), as did younger participants (78% vs 59% respectively).
**Discussion:** In this study we showed that e-data collection has lower response rates than paper-data collection in an adult population that includes people of working age. Results of this study could have been limited by the study design; randomisation of patients to the data collection method could have provided more robust data.

## P-180

### Trial questionnaire response rates - Is bigger better?

Tracey Davidson, David Cooper
University Of Aberdeen, Aberdeen, United Kingdom
*Trials* 2019, **20(Suppl 1):**P-180

Retention in randomised controlled trials (RCT) can often seem like the poor cousin in comparison to the time and effort that is put into recruitment. Trial managers and data coordinators/administrators often spend inordinate amounts of time and effort collecting participant reported outcome (PRO) data using different strategies (e.g. postal questionnaires, reminders etc.). This can be a large burden upon both the trial budget and time for the trial team.
Within the NIHR HTA-funded SIMS Trial (a pragmatic multi-centred surgical RCT in female stress urinary incontinence) we evaluated whether sending A4 or A5 sized questionnaires with identical content, layout and pagination to participants affected questionnaire retention/response rates.
**Objectives:** The aim of the study was to determine if the physical size of the questionnaires affected response rates. We will also consider economic aspects (cost of questionnaires, postage etc.).
**Methods:** Participants were randomised to receive either A4 or A5 sized postal questionnaire at 15 months post randomisation.
**Results:** There was no statistically significant difference in response rates between the A4 and A5 sized questionnaires. 58.70% of participants receiving an A4 questionnaire responded compared to 52.10% of those receiving an A5 questionnaire. N = 562, P-value = 0.116
In addition to discussing this result, economic aspects, by questionnaire size, will be presented at the Conference.

## P-181

### Managing follow-up among parents of very pre-term infants: methods to improve questionnaire response rate

Madeleine Hurd[1], Ursula Bowler[1], Jon Dorling[2], Samantha Johnson[3], Ed Juszczak[1], Oliver Hewer[1]
[1]NPEU Clinical Trials Unit, Nuffield Department of Population Health, University Of Oxford, Oxford, United Kingdom; [2]IWK Health Centre, Halifax, Canada; [3]University of Leicester, Leicester, United Kingdom
*Trials* 2019, **20(Suppl 1):**P-181

**Introduction:** In randomised controlled trials it is important to maximise outcome ascertainment to minimise bias. SIFT (ISRCTN76463425)

was a multicentre randomised controlled trial run in neonatal units in the UK and Ireland, investigating two speeds of increasing milk feeding in 2804 infants with gestational age at birth <32 weeks and/or birth weight <1500g. Primary outcome was the proportion of infants surviving without moderate or severe neurodevelopmental disability at 24 months of age, assessed via parent-completed questionnaire.
**Methods:** Questionnaires were posted 17 days before participants reached 24 months of age corrected for prematurity. Lack of parental response triggered two postal reminders and one by phone, with two weeks between each.
Measures introduced to improve response included contacting parents prior to the questionnaire being posted; offering online questionnaire completion; a second reminder being accompanied by a phone call and text message; promotion by Bliss (third sector stakeholder); sending posters to sites for display in outpatient clinics; and ultimately an incentive voucher (described elsewhere). Outcome data were also sourced from routine clinical follow-up appointments at sites for infants whose parents did not complete the questionnaire.
**Results:** Response rate prior to all interventions was 51.0%; at data lock in April 2018 it was 76.5% (p<0.01). The largest increases (6%) following a single intervention were seen after introducing pre-questionnaire phone calls (p=0.07) and online completion (p=0.04).
Recruiting sites supplied additional data from 351 routine clinical follow-up appointments. Primary outcome could be determined for 88.5% of the cohort.
**Discussion:** Multiple methods of contact, especially phone contact prior to dispatch of questionnaire and availability of the questionnaire online, may improve response rate to postal questionnaires among parents of very preterm infants. Over time, promotion by sites and on social media may also play a role. Missing data can be supplemented by information from routine sources.

## P-182

### Conditional versus Non-Conditional Incentives to Maximise Return of Postal Questionnaires in Clinical Trials: A Randomised Study Within a Trial

Johanna Cook, Christopher Butler, Jonathan Cook, Emily Bongard, Carl Heneghan
University of Oxford, Oxford, United Kingdom
*Trials* 2019, **20(Suppl 1):**P-182

**Background:** High levels of retention in clinical trials is essential to gain robust evidence to guide care. Many approaches have been used to improve participant retention, but few have been evaluated. The addition of a monetary incentive has been shown to increase retention, but it is not known whether the point at which an incentive is given matters. We aimed to determine whether there was a difference in follow-up trial questionnaires returned when a monetary incentive given to trial participants at recruitment (non-conditional), and when patients were informed at recruitment that the incentive would be given only once a questionnaire had been returned (conditional).
**Method:** This was a sub-study within the Antivirals for influenza-Like Illness, An rCt of Clinical and Cost effectiveness in primary CarE Trial. Sites were matched according to previous recruitment or practice list size. Practice pairs were randomised to giving either a non-conditional or conditional incentive. Analyses were conducted according to randomised group irrespective of compliance. Statistical significance was assessed at the two-sided 5% level. The primary analysis was regression adjusted for practice pair with various sensitivity analyses.
**Results:** Only 28 out of the 42 sites recruited at least one participant (range 1 to 56) with 10 practice pairs recruiting one or more participants at both constituent sites. There was no evidence of a difference in the proportion of questionnaires returned, time taken to return questionnaires, nor proportion of pages completed, by intervention group (all p>0.05). Findings of the sensitivity analyses yielded similar findings. The conditional incentive cost approximately £23 less per diary returned.
**Discussion:** There was no evidence of a difference in questionnaire returns, nor the time to questionnaire return or completeness. There was low precision, given the small number of sites which recruited, and variability between sites in recruitment performance. The conditional approach cost less.

## P-183

### Pen and Social Incentive Letter Retention Study within a Trial (SWAT) - An embedded, factorial design randomised controlled trial to investigate whether the inclusion of a pen and/or social incentive text cover letter included with the 12-month postal questionnaire improved response rates

Sophie James, Adwoa Parker, David Torgerson
York Trials Unit, University of York, York, United Kingdom
*Trials* 2019, **20(Suppl 1):**P-183

**Introduction:** Poor return of questionnaires in randomised controlled trials (RCTs) affects retention rates. This can introduce bias and thus affect generalisability and validity, with an associated reduction in statistical power. The objective of this study within a trial (SWAT) was to assess whether a pen and/or social incentive text cover letter sent with the 12-month questionnaire increased postal questionnaire response rates for participants in an RCT. We aimed to compare the inclusion of a pen in questionnaires with no pen; and the use of a social incentive text cover letter compared with no cover letter.
**Methods:** A 2x2 factorial SWAT within the 'Occupational therapist home assessment and modification for prevention of falls trial (OTIS)' host trial. Participants due their 12-month follow-up questionnaire were randomised to be sent a pen; a social incentive text cover letter; both; or neither. Primary outcome was the proportion of participants in each group who completed and returned the questionnaire. Secondary outcomes were time to return and completeness of the questionnaire, number of reminder letters sent and the cost effectiveness. To date 624 participants have been randomised.
**Timing of Potential Results:** By the time of the conference we will present findings on questionnaire response rates, time to return and completeness of the questionnaire, number of reminders and cost effectiveness. Odds ratios will be calculated and reported, along with confidence intervals and p values. Adjusted hazard ratio results will be presented for time to return the questionnaire, and the need for a reminder.
**Potential Relevance and Impact:** Our SWAT will add to evidence for improving retention rates in RCTs. Findings of the pen SWAT will be combined with results of other SWATs in a meta-analysis to detect small but cost-effective differences. Evidence for the social incentive cover letter will need to be replicated in further SWATs.

## P-184

### Factors that affect attrition in RCTs for the treatment of depression

Saleema Selwiyn Rex[1], David White[2], Robin Chatters[2], Mike Bradburn[2]
[1]York Trials Unit, University of York, York, United Kingdom; [2]Clinical Trials Research Unit, University Of Sheffield, Sheffield, United Kingdom
*Trials* 2019, **20(Suppl 1):**P-184

**Introduction:** Attrition is a common feature in clinical trials; higher attrition rate can affect the statistical power of an RCT and can undermine the external validity of the study. Therefore, it is important to evaluate and understand the factors that could affect attrition so that informed decisions can be made when planning a trial. We undertook a systematic review and meta-analysis of randomised studies treating depression to determine attrition, and predictors of attrition in these RCTs.
**Methods:**A comprehensive search was undertaken to identify RCTs of interventions to treat depression. Firstly, Cochrane reviews with "depression" in the title, abstract or keywords were identified and were eligible if were published in or after 2005; their scope matched our review's eligibility criteria; and their inclusion criteria did not require another co-morbidity to be present (e.g. Bipolar disorder). We then screened both included and excluded trials from these reviews to identify eligible trials.
As well as estimating attrition rates overall, we hypothesised these may depend on year of the study, blinding, burden of the outcome measures, follow-up schedules, intervention burden, target sample size, recruitment setting, number of arms, type of intervention, type of control and participant characteristics.
**Results:** Many studies did not report their target sample size, number screened or drop out details. The average attrition rate was 22% but ranged from 0% to over 70%. Our results suggest that trials that had combinations of intervention types (i.e. behavioural and drugs) had lower attrition rate than trials with interventions that were purely drugs or behavioural therapy.
**Discussion:** Although a review cannot identify and quantify the true relationship between trial design and attrition, this review provides some insight into what levels of attrition may be expected in a future trial. It also highlights the lack of reporting of key factors.

## P-185

### Methods to improve follow up procedures in a sexual health study [safetxt]

Kimberley Potter, Lauren Jerome, Megan Knight, Christina Sparks, Zahra Jamal, Rosemary Knight, Ona McCarthy, Melissa Palmer, Caroline Free
London School Of Hygiene And Tropical Medicine, United Kingdom
*Trials* 2019, **20(Suppl 1):**P-185

**Introduction:** Almost half of trials fail to achieve their follow up target. Follow up in young people and on sensitive topics is particularly challenging, with less than 50% return rate of postal STI samples sent to young people in previous trials. Effective evidence-based strategies to increase follow up rates were used.
safetxt, a study of a sexual health intervention, recruited 6252 participants aged 16-24 from 52 UK sexual health sites. Participants were required to return two postal questionnaires and an STI test kit at 12 months. We used evidence-based strategies to increase follow up rates through contact by post, telephone, text message and email.
In May 2018, follow up return rates were 88.7% for the four week questionnaire, 74.7% for the one year questionnaire and 67.75% for the test kit. We aimed to further increase follow up rates.
**Methods:** Retaining participants that regularly changed address and phone number was challenging, but contact was achieved through various approaches. Sites were encouraged to emphasise the importance of follow up to participants at the time of consent and provided participants with pocket cards with a reminder of follow up dates. Mail outs were developed to improve communication with participants and simplify return methods.
Additional evidence based methods were implemented, including the use of postage stamps on return envelopes and a cash prize draw for returned test kits.
**Results:** By April 2019, follow up rates of the four week questionnaire were maintained. At one year, follow up rates were 76.8% for the questionnaire and 71.7% for the test kit.
Follow up rates for the one-year questionnaire and test kits returns have continued to increase since introducing strategies from May 2018.
**Discussion:** Even when evidence based methods are already being used, developing new approaches and employing additional evidence based strategies improved follow up rates.

## P-186

### Assessing attrition in Randomised Control Trials, the identification of attrition risk factors and the challenges of poor reporting: A comparison of reports from 2013 and 2018

Anna Kearney[1], Anna Rosala-Hallas[2], Naomi Rainford[2], Jane M Blazeby[3], Mike Clarke[4], Athene J Lane[3], Paula R Williamson[1], Carrol Gamble[1]
[1]North West Hub for Trials Methodology Research and Clinical Trials Research Centre, Department of Biostatistics, University of Liverpool, United Kingdom; [2]Clinical Trials Research Centre, Department of Biostatistics, University of Liverpool, United Kingdom; [3]ConDuCT-II Hub, School of Social and Community Medicine, University of Bristol, United Kingdom; [4]Centre for Public Health, Queen's University Belfast, United Kingdom
*Trials* 2019, **20(Suppl 1):**P-186

**Background:** Addressing attrition in clinical trial design is an important priority. Despite availability of statistical methods for missing data, prevention is preferred. The development of effective retention interventions needs to be based on improved understanding of attrition risk factors. We aimed to identify attrition risk factors.
**Methods:** Two-arm, parallel, RCTs reported in JAMA, NEJM, BMJ and The Lancet during 2013 and the first quarter of 2018 were identified using MEDLINE(Ovid). The number of randomised participants without observed primary outcome data were dual extracted. Associations with intervention type, primary outcome characteristics and trial setting were assessed using univariate analysis.
**Results:** 141/159 (89%) of 2013 trials had missing data equating to 5.4% [1.5, 10.7] of randomised participants per trial. This was lower in 2018 with 38/46 (83%) reporting a median 2.6% [0.3-15.4]. In 2013, increased attrition was associated with outpatient data collection, studies within chronic conditions, smaller trials (recruitment target and number randomised), shorter recruitment and longer follow up. Data collection by clinicians and recruitment in acute settings was associated with lower levels of attrition. The 2018 cohort generally supported these observations although in some areas the numbers were too small for comparison. Data extraction was challenging and the CONSORT often did not provide an effective trial summary: A fifth of all diagrams were in the supplementary material; 19% did not report the numbers analysed for the primary outcome and for a further 6% this did not match the results; Imputed data was not clearly reported in 27%.
**Discussion:** Levels of missing data were lower than anticipated, but this still equated to wasting an average of one month of participant recruitment. Poor reporting may underestimate the extent of missing data. Improvements to the CONSORT are recommended, in particular explicit reporting of imputed primary outcome data.

## P-187

### Generating collaborative relationships for a successful trial follow-up

Alpana Ghadge, Rebecca Brown, Karen Bracken
NHMRC Clinical Trials Centre, University of Sydney, Camperdown, Australia
*Trials* 2019, **20(Suppl 1):**P-187

**Introduction:** Collection of developmental follow-up outcomes is critical to demonstrate long-term effects of interventions in neonatal trials.
The Benefits of Oxygen Saturation Targeting (BOOSTII) trial enrolled 1135 infants born less than 28 weeks gestation, to investigate the effect of oxygen saturation levels during their hospitalisation. The primary outcome was death or major disability at 2 years of age. A high rate of 2-year follow-up was therefore crucial. Maintaining contact with families during follow-up presented numerous challenges, as families were no longer under the care of the hospital and frequently moved. We aim to describe various strategies implemented that achieved a high follow up rate for the BOOST II trial.

**Method**

1.Interactions with recruiting centres: The central coordinating team fostered positive and supportive relationships with the recruiting hospitals through regular (but not overwhelming) contact using multiple modes of communication, such as email, telephone contact, satellite meetings at annual conferences and celebration of trial milestones.

2.Interactions with families involved: Multiple tools were provided to recruiting hospitals to support their contact with families, such as personalised Christmas cards, trial newsletters, celebration of birthdays with nominal surprise baby gifts, and transport assistance for families suffering financial hardship.

Results

The 2-year follow up rate on the BOOSTII trial was over 96%.The strategies we introduced have been widely taken up by our study sites and implemented in other similar trials.

**Discussion:** One of the challenges with long term follow-up is maintaining effective communication. Establishing positive relationships promoted high follow-up rates through ongoing engagement with families. We suggest that small positive interactions with site research staff and families will alter perceptions of clinical trials and create a sense of shared ownership.

## P-188

### Maximising follow up rates of patient reported outcome measures: a study within a trial (SWAT) - results from the LoTS2Care Feasibility Trial

Lauren Moreau[1], Ivana Holloway[1], Seline Ozer[2], Anne Forster[2], Claire Hulme[3], Suzanne Hartley[1], Richard Brindle[1], Amanda J. Farrin[1]
[1]Clinical Trials Research Unit, Leeds Institute of Clinical Trials Research, University Of Leeds, Leeds, United Kingdom; [2]Academic Unit of Elderly Care and Rehabilitation, Bradford Institute for Health Research, Bradford Teaching Hospitals NHS Foundation Trust, Bradford, United Kingdom; [3]College of Medicine and Health, University of Exeter, Exeter, United Kingdom
*Trials* 2019, **20(Suppl 1):**P-188

**Background:**

Ensuring satisfactory follow-up rates in trials, where primary outcomes are patient-reported measures obtained via postal questionnaires, can be challenging. This challenge intensifies in populations with communication or cognition problems, as minimising measurement burden is key.

**Methods:**

LoTS2Care, a NIHR-funded Programme, included a feasibility cluster randomised trial, assessing a self-management intervention addressing longer-term health and social care needs for stroke survivors. Participants were followed-up at three, six and nine months using postal questionnaires, containing various self-reported outcome measures, alongside a resource use questionnaire.

A study within a trial (SWAT) was conducted to determine the most acceptable questionnaire format to maximise follow up rates for a future definitive trial. Stroke survivors (and available carers) were randomised (1:1) to receive one of two alternative questionnaire formats: a single comprehensive booklet containing all measures (19 pages) or two shorter booklets: one containing the outcome measures (12 pages); the other containing the resource use questionnaire (7 pages) at the six and nine month follow-up time points.

**Results:**

The study recruited 269 stroke survivors and 85 carers. At six months follow-up, 227 stroke survivors were randomised in the SWAT; with 212 still available for follow-up at nine months. Participants were more likely to complete all questionnaires at nine months when randomised to the single booklet group (97.2% vs. 91.3%, OR=3.32, 95% CI (0.87 – 12.61)). This trend was also observed at six months, but not for the carer completion rates.

**Relevance & Impact:**

Postal follow-up is common in many trials; strategies to maximise return rates are essential. This SWAT demonstrated that return rates are maximised when all study questions are contained within a single, but lengthier, postal booklet, contrary to prior expectations. This result is particularly pertinent to stroke and older populations, where communication and cognitive issues are common.

## P-189

### Maximising participant retention in a randomised prevention trial

Rachel H Haines[1], Joanne R Chalmers[2], Richard Swinden[1], Lucy E Bradshaw[1], Alan M Montgomery[1], Hywel C Williams[2]
[1]Nottingham Clinical Trials Unti, Nottingham, United Kingdom; [2]Centre of Evidence Based Dermatology, Nottingham, United Kingdom
*Trials* 2019, **20(Suppl 1):**P-189

**Introduction:** We explored strategies to enhance retention within a pragmatic, multicentre, randomised controlled eczema prevention trial (BEEP Study). The study randomised 1394 newborns from high risk families. The primary outcome of eczema was collected at 24 months.

We surmised that retention in a prevention trial of young families with healthy babies, having no face to face contact between consent and 24 months, would be challenging. Our sample size estimated 20% attrition and retention strategies were incorporated into the trial design.

**Methods:** Strategies for enhancing retention included: chasing questionnaires by post, text and phone (3, 6, 12, 18 months), sending birthday cards, small gifts (12 and 18 months), newsletters, appointment letters (21 months), and flexible visit scheduling at participant homes. An online scheduling system, monthly updates of upcoming appointments and retention league tables were provided to sites.

After 6 months of follow-up, retention was 76% and there were concerns this would drop further. Additional retention strategies initiated by the Trial Management Group (TMG) included: simplified appointment letters, handwritten notes to participants lost to follow-up and development of an alternative data collection process via post or through the participant's GP. Sites were offered additional support through teleconferences and an incentive scheme.

The TMG continued to monitor upcoming appointments and retention by site to allow for issues to be effectively and quickly managed.

**Results:** The final retention rate at 24 months was 1212/1394 (87%). Retention was balanced between groups but varied between the 14 sites (70% to 97%). Primary outcome collection was face to face for 1123 participants (81%) and remote for 89 (6%).

**Discussion:** Little evidence for targeted retention strategies exists; in this trial a combination of proactive and reactive retention strategies was required. Future trials with little direct participant contact may need to budget for similar multifaceted and resource-intensive retention strategies.

## P-190

**Abstract withdrawn**

## P-191

**Abstract omitted**

## P-192

### A novel application of the matched nested case-control design in the secondary analysis of clinical trial data

Christopher Partlett[1,2], Nigel Hall[3], Alison Leaf[2,4], Ed Juszczak[2], Louise Linsell[2]
[1]Nottingham Clinical Trials Unit, Nottingham, United Kingdom; [2]National Perinatal Epidemiology Unit, Oxford, United Kingdom; [3]University of Southampton, Southampton, United Kingdom; [4]University of Bristol, Bristol, United Kingdom
*Trials* 2019, **20(Suppl 1):**P-192

**Introduction:** A matched nested case-control study is an efficient design, conventionally used within a cohort study where there is a cost associated with measuring the exposure. We demonstrate how this study design can be embedded within a randomised trial and illustrate its potential for answering important research questions through the secondary analysis of prospectively collected trial data. We also highlight the advantages of this method over the conventional case-control design.
**Methods:** We applied this study design to a secondary analysis of the Abnormal Doppler Enteral Prescription Trial (ADEPT; ISRCTN:87351483). We investigated the role of milk feed type and changes in milk feed type (exposures) in the development of severe necrotising enterocolitis (NEC, outcome) in a group of 399 high risk growth-restricted preterm infants. We matched cases (infants with NEC) with up to four controls (infants without NEC) of the same sex and smallest Mahalanobis distance based on gestational age and birthweight. Conditional logistic regression was used to calculate an adjusted odds ratio between the exposure and outcome, adjusted for trial allocation.
**Results:** Using matching, we were able to generate a comparable sample of controls for the cases, and thereby reliably investigate the temporal relationship between feed type and NEC. Advantages of this method over the conventional case-control design were that controls were selected from the same population, exposure status was collected prospectively, and compared between cases and matched controls at the point at which an outcome event occurred.
**Conclusions:** A matched nested case-control study can be used to identify credible associations in a secondary analysis of clinical trial data where the exposure of interest was not randomised, and has several advantages over a standard case-control design. This method offers the potential to make reliable inferences in scenarios where it would be unethical or impractical to perform a randomised clinical trial.

## P-193

### Applying mixed models and quantile regression for a trial of breast care nurse delivered cognitive behavioural therapy (CBT) intervention to reduce the impact of hot flushes in women with breast cancer using partially nested data

Laura Day[1], Tom Maishman[1], Kayleigh Anne Hill[1], Debbie Fenlon[2], Myra Hunter[3], Jacqui Nuttall[1], Gareth Griffiths[1]
[1]Southampton Clinical Trials Unit, University of Southampton, Southampton, United Kingdom; [2]Department of Nursing, College of Human and Health Sciences, Swansea University, Swansea, United Kingdom; [3]Institute of Psychiatry, Psychology & Neuroscience, King's College London, London, United Kingdom
*Trials* 2019, **20(Suppl 1):**P-193

**Introduction:** Hot flushes and night sweats (HFNS) are problematic in women after treatment of breast cancer. Cognitive Behavioural Therapy (CBT) is known to be effective for the alleviation of hot flushes. However, it is not currently offered within the NHS for women with breast cancer, and it is not known whether it can be effectively delivered by breast care nurses (BCNs) in the NHS context.
This study's aim was to assess the impact of HFNS at 26-weeks post-randomisation to determine whether BCNs can be trained to effectively deliver CBT.
**Methods:** MENOS4(Breast Cancer Now:2015CR_004) is a multicentre phase III individually randomised trial of BCN delivered group CBT versus Usual Care and recruited 130 participants across six UK sites. Mixed models for partially clustered data were intended to be used for all primary and secondary outcomes to account for the therapist effect. However, for some secondary outcomes, the residuals were not normally-distributed and no sensible data transformations could be found, and quantile regression was therefore incorporated. All models adjusted for baseline score, and stratification factors of cohort and site.
**Results:** Mixed models were used to assess the primary outcome and all secondary outcomes in which the residuals were found to be normally-distributed, and quantile regression was used for all other secondary outcome measures. Both models found statistically significant improvements for CBT compared to Usual Care.
**Discussion:** Mixed models allowed us to adjust for the baseline questionnaire responses, site and cohort, and therapist effect with a partially clustered data design. Quantile regression was a valuable alternative model when no sensible transformations could be found as it continued to allow for the comparison of outcomes with an adjustment for baseline score, cohort and site. However, utilising this method resulted in no longer being able to adjust for the therapist effect in the nested design.

## P-194

### Analysis of duration of remission as an intention-to-treat analysis with application to the TURING trial

Wendi Qian[1], Andrea Machin[1], Megan Griffith[2], Lisa Willcocks[3]
[1]Doctor, Cambridge, United Kingdom; [2]Renal Unit, Hammersmith Hospital, Imperial College London, London, United Kingdom; [3]Cambridge University Hospital NHS Foundation Trust, Cambridge, United Kingdom
*Trials* 2019, **20(Suppl 1):**P-194

**Background:** In a randomised trial, direct comparison of duration of remission between treatments could be biased when it only includes patients who respond. The issue, however, has not been much of a concern, as remission rate rather than duration of remission is commonly used as the primary outcome measure, with comparison of remission rates an intention-to-treat (ITT) analysis.
**Methods:** Current treatments for nephrotic glomerulonephritis have serious limitations: although glucocorticoids are effective for the majority of patients, repeated courses are normally required, and the side effect of cumulative steroid exposure itself results in high morbidity and mortality in patients, just as in other autoimmune diseases where immunosuppression is required; steroid sparing regimens, that is, ultimately, a long remission duration treatment is needed. The TURING study is a randomised, two-arm, double blind, placebo controlled phase III trial to assess the efficacy of rituximab in treating nephrotic syndrome in patients with minimal change disease or focal segmental glomerulosclerosis. The primary outcome measure is the duration of remission. As an extension of the Kaplan-Meier survival curve method, the probability of a patient being in remission, will be used as a means of estimating the expected duration of remission across all randomised patients (ITT), given as the estimated remission rate times mean duration of remission for responding patients. This approach can be considered as a stochastic process in which a patient must start in state 0 (that is at start of treatment) and eventually progress to an absorbing state, 2 (progression, death in the absence of progression, treatment failure, relapse (or died from disease)), possibly passing through a transient state 1 (remission).
**Conclusions:** The TURING trial is ongoing. Simulation results with underlying time to response, duration of response and time to relapse from exponential, Weibull and log-normal distributions will be reported and discussed.

## P-195

**Controlled multiple imputation: an accessible flexible tool for estimating hypothetical estimands in clinical trials**

Suzie Cro[1], Susan Chan[2,3], Victoria Cornelius[1]
[1]Imperial College London, London, United Kingdom; [2]Guy's and St. Thomas' NHS Foundation Trust, London, United Kingdom; [3]King's College London School of Life Course Sciences & School of Immunology & Microbial Sciences, London, United Kingdom
*Trials* 2019, **20(Suppl 1):**P-195

**Introduction:** In clinical trials, intercurrent events such as use of rescue medication are often unavoidable. Typically for primary analysis the use of rescue medication will be ignored as a treatment-policy estimand, which estimates the effect of treatment assigned at baseline, will be of interest. A treatment policy estimand will be estimated by performing an Intention-to-treat analysis. But in trials where rescue medication is made available it can also be of value to conduct a supplementary analysis to establish the treatment effect that would have been obtained if rescue medication had not been taken. The recent publication of the ICH-E9 addendum has brought the estimation of such alternative 'hypothetical' estimands
into sharp focus. However, guidance is not provided on how these estimands may be statistically estimated.
**Methods:** We demonstrate the use of controlled multiple imputation procedures for estimating hypothetical estimands in the absence of rescue medications, using data from the Atopic Dermatitis Anti-IgE Paediatric Trial. This includes delta-based imputation and last mean carried forward imputation. Data collected post rescue medication initiation is set missing, then imputed under a contextually relevant assumption that reflects the hypothesised value of the outcome in the absence of rescue medication.
**Results:** The treatment policy estimand for the Children's Dermatology Life Quality Index was -3.6, 95% CI -6.5 to -0.6, p=0.018 (favouring Anti-IgE). When the rescued were assumed to have a mean outcome ranging from 0 to 7 Index points worse (greater) than the observed non-rescued, the average treatment effect ranged from -4.0 to -4.6. The treatment effect following last mean carried forward multiple imputation was -4.0, 95% CI -7.1 to -0.9, p=0.012.
**Discussion:** Supplementary analysis revealed Anti-IgE is an effective treatment for severe atopic dermatitis in the absence of rescue medication. Controlled multiple imputation provides a flexible accessible tool for estimating hypothetical estimands.

## P-196

**Abstract omitted**

## P-197

**Use of time-varying covariate in assessing disease remission in the early and late phases of treatment with application to RITAZAREM trial**

Marianna Nodale
Cambridge Clinical Trials Unit, Cambridge, United Kingdom
*Trials* 2019, **20(Suppl 1):**P-197

**Introduction:** In a randomised trial, we are interested in assessing whether overall improvement in relapse-free survival is sustained beyond a treatment period.
The RITAZAREM trial aim is to assess the efficacy of rituximab compared to the current standard of care (azathioprine or methotrexate and glucocorticoids) in the prevention of disease relapse in ANCA-associated vasculitis (AAV). Rituximab is an established induction agent in AAV, however the trial aims to demonstrate its efficacy as a maintenance agent given that a large majority of patients relapses within 2 years of a course of treatment.
**Methods:** Patients are recruited at the time of relapse and initiated on a 4 months induction regimen of rituximab. Those achieving disease control are then randomised to either maintenance treatment with rituximab lasting 20 months or standard therapy with azathioprine. Patients are followed up to between 36 and 48 months from enrollment. The primary outcome measure is time to disease relapse from randomisation. A secondary objective is to demonstrate disease remission beyond the 24 month treatment period. The primary intention-to-treat analysis will be based on a Cox proportional hazard model. Firstly we plan to test the null hypothesis for a hazard ratio of 1 at all time points. If rejected at a global level, we will employ time-varying covariates to investigate the two further sub-hypotheses of a hazard ratio of 1 pre- and post- 24 months. This will elucidate whether rituximab overall efficacy in maintaining disease remission is achieved during the active treatment phase of the trial and also sustained in the post-treatment phase.
**Potential Relevance & Impact:** The RITAZAREM trial is ongoing, with expected completion in November 2019. Simulations will investigate operational characteristics of our model under a variety of assumptions. Alternative methods of assessing efficacy at varying time points will also be considered.

## P-198

**Defining protocol deviations in a pragmatic non-inferiority trial of potassium control after Coronary Artery Bypass Grafting**

Elizabeth Allen[1], Diana Elbourne[1], Joanna Sturgess[1], Richard Evans[1], Ruth Canter[1], Matthew Dodd[1], Ben O'Brien[2]
[1]Lshtm, London, United Kingdom; [2]St Bartholomew's Hospital & Barts Heart Centre, London, United Kingdom
*Trials* 2019, **20(Suppl 1):**P-198

**Introduction:** Intention to treat (ITT) analysis is the analytic method of choice for superiority trials as it adheres to the randomisation process and is conservative. However, the ITT analysis may not be conservative for non-inferiority trials as including non-compliers may bias towards equivalence. Whilst a per-protocol (PP) analysis, is more likely to identify differences it can also bias results. Therefore, the recommended approach for non-inferiority trials is to carry out both an ITT and PP analysis. A clear definition of compliance with the intervention is therefore needed.
The Tight K trial is a randomised non-inferiority trial investigating the impact of maintaining serum potassium ≥3.6mEq/L vs ≥4.5mEq/L on the incidence of new onset atrial fibrillation after isolated elective Coronary Artery Bypass Grafting. A pilot trial was carried out.
Defining compliance with the potassium protocols in each arm is crucial for the analysis of the trial; however, is not straightforward. Whilst the protocols specify maintaining levels of potassium, supplementation generally occurs when a patient falls below the level required. Therefore, compliance cannot be based on all, or even most measurements being above the required thresholds. Additionally, patients' potassium levels vary differently by patient, with greater variability observed in some.
**Methods:** Here we use data from the pilot trial to explore possible definitions of compliance using statistical process control.
**Timing of Potential Results:** Initial Control Charts suggest that they could help define algorithms to identify patients who have not complied with their assigned protocol. Work is ongoing and results will be available by October 2109.
**Potential Relevance & Impact:** Many medical protocols are administered in the belief that they will have the desired effect with little evidence that they do. There is currently no accepted way of assessing protocol deviations in these situations such and the proposed approach shows promise.

## P-199

**Error rate control in perpetual platform trials**

David S Robertson[1], James M S Wason[1,2]
[1]MRC Biostatistics Unit, University of Cambridge, Cambridge, United Kingdom; [2]University of Newcastle, Newcastle, United Kingdom
*Trials* 2019, **20(Suppl 1):**P-199

**Introduction:** Platform trials allow the evaluation of multiple experimental treatments under a single master protocol and are designed to be perpetual in that new treatments can continue to be seamlessly added to the trial in the future. In a perpetual platform trial, controlling the family-wise error rate (FWER) leads to a low statistical power, as vanishingly small significance levels must be used for the later hypotheses being tested. An alternative error rate is the false-discovery rate (FDR), which controls the expected proportion of recommended treatments that are actually ineffective. In this work, we apply recent methodological research to show how to control the FDR in the perpetual platform trial setting.
**Methods:** We investigate various rules for controlling the FDR in perpetual platform trials, and propose a simple modification to the procedures for when there is an upper bound on the number of hypotheses to be tested. The number of ultimately evaluated treatment arms and proportion of treatments that are truly effective are varied.
**Results:** We show that the FDR can be controlled in perpetual platform trials, even under the dependence induced by a common control arm. Furthermore, controlling the FDR in perpetual platform trials results in a uniformly higher power compared with controlling the FWER.
**Discussion:** In perpetual platform trial settings, we recommend that sponsors and trialists consider controlling the FDR

## P-200

### Options and challenges of analysing data from recruitment intervention studies A lesson from MRC START Hi-Light data analysis

Wei Tan, Trish Hepburn
University of Nottingham, nottingham, United Kingdom
*Trials* 2019, **20(Suppl 1):**P-200

**Background:** Achieving high participation in RCTs has traditionally been difficult and there is a need to develop and test interventions to improve recruitment. Embedded randomised trials of recruitment interventions within in ongoing host RCTs have been growing recently.
MRC START Hi-Light is embedded within the H-Light study to test whether addition of multimedia resource (MMI) impacts on rate of recruitment, in comparison with standard host trial website.
**Methods:** The primary outcome was the proportion of patients randomised to the main trial, which was calculated and compared between the two arms. Various statistical methods were tested to find the most appropriate model to analyse the data and answer the research question.
**Results:** Linear model assumptions were violated for the original and the log-transformed data.
Negative binomial model was then tested to compare mean number of patients recruited, the assumptions of which were again not met. A zero inflated negative binomial was proposed however due to the single source of zero value in this study this model was deemed inappropriate.
As around 80% recruitment units recruited either 0 or 1 patient, we estimated the effect of MMI website using a binary outcome variable, where 0=recruited none and 1=recruited at least 1. Results from binary measure (risk difference = -1.6%, 95% CI -18.6% to 15.4%) indicate no evidence of effect of MMI. Results from other methods were not presented but in support of the estimates.
**Conclusion :** Recruitment intervention studies may result in data very similar to this study where the choice of primary analysis model must be carefully scrutinized. In this study various methods have been tested to best analyse the data which all support no evidence of intervention effect in improving recruitment. Better planning on the most appropriate analysis model is recommended to produce more reliable and robust results.

## P-201

### Effect-based traffic light progression criteria for pilot studies

Gareth Mccray, Martyn Lewis, Kieran Bromley, Gillian Lancaster
Keele University, United Kingdom
*Trials* 2019, **20(Suppl 1):**P-201

**Introduction:**
In the CONSORT guidelines for pilot and feasibility trials, formal hypothesis testing of effectiveness or efficacy is not recommended as it is likely that a study will have insufficient power to supply robust evidence (and this is the aim of the main trial). However, particularly for larger pilot studies and/or studies with a large assumed MCID/effect size, the results of a pilot may carry valuable information about the likely outcome of any follow-up trial. This presentation suggests a method that uses the pilot sample size and observed effect size to provide a traffic-light indication of the likely fruitfulness or futility of any follow-on trial.
**Methods:**
The traffic-light designation is based on the simulated sampling distributions of the effect size under both null and specified alternative hypotheses. The designation of "green", "amber", or "red" depends on the likelihood of the observed effect size given the sampling distributions. At smaller sample/effect sizes, an "amber" is most probable, but as sample/effect size increase, either "red" or "green" designations increase in probability, reflecting the additional information about the true effect from the data.
For "red" we recommend not to go to a full trial, for "amber" we recommend focusing more on the process outcomes to inform progression, while for "green" we recommend progression and there may be less need to focus on other process outcomes.
**Results:**
Look-up tables of critical values are presented for the cases of a difference between two independent means and two independent proportions (exact values). Worked examples from authentic studies illustrate the utility of the methodology.
**Discussion:**
While we strongly discourage potential efficacy/effectiveness being the primary feasibility outcome considered in a pilot study, we do however think that it warrants consideration in the 'proof of efficacy' debate and we present a reasoned method of doing so.

## P-202

### A simulation study to compare longitudinal methods for the analysis of randomised trials and the implications for sample size calculation

Bethan Copsey, Susan J Dutton, Ray Fitzpatrick, Sarah E Lamb, Jonathan A Cook
University of Oxford, United Kingdom
*Trials* 2019, **20(Suppl 1):**P-202

**Introduction**: The majority of randomised trials collect outcome data at multiple follow-up time points. Reviews have shown that analysis methods vary across different trials, such as mixed effects methods or repeated single time point analyses. The use of these different methods can have implications for the credibility of trial results, impacting on the type I error, level of power and bias of the treatment effect estimate.
This simulation study aims to compare the performance of different methods to analyse longitudinal data from a randomised trial.
**Methods:** Simulated datasets were generated for 240 different scenarios with different sample sizes (100, 200, 400, 600, 800), numbers of follow-up time points (2, 3, 4, 5), maximum level of treatment effect (0, 4, 8, 12) and different patterns of treatment effect. For each scenario, 1600 datasets were produced. Distributional parameters were based on the WOMAC measure and informed by a previous randomised trial of medications for knee osteoarthritis.

The statistical methods used to analyse each of the datasets were:
1.Repeated analysis of covariance (ANCOVA) at each follow-up time point.
2.Generalised estimating equations, and
3.Mixed effects.
The performance measures used to compare the methods were convergence, power, type I error, and coverage. Stata IC 14 software and the University of Oxford Advanced Research Computing (ARC) facility are being used to conduct this work.
**Timing of Potential Results:** It is anticipated that this study will be completed during the summer of 2019.
**Potential Relevance & Impact:** The results will provide evidence to inform the choice of longitudinal analysis methods in randomised trials. This could allow trialists to select the methods that will provide the highest statistical power for the anticipated pattern of treatment effect and could result in reducing the required sample size whilst maintaining adequate statistical power.

## P-203

### Allowance for learning and clustering effects in the design and analysis of multicentre randomised trials: current practice and experiences

Elizabeth J Conroy[1], Jane M Blazeby[2], Girvan Burnside[1], Jonathan A Cook[3], Carrol Gamble[1,4]
[1]Department of Biostatistics, University of Liverpool, a member of Liverpool Health Partners, Liverpool, United Kingdom; [2]Centre for Surgical Research, Population Health Sciences, University of Bristol, Bristol, United Kingdom; [3]Centre for Statistics in Medicine, University of Oxford, Oxford, United Kingdom; [4]North West Hub for Trials Methodology Research, University of Liverpool, Liverpool, United Kingdom
*Trials* 2019, **20(Suppl 1):**P-203

**Introduction:** Patient outcomes can depend on the treating centre, or health professional, delivering the intervention. Differences in treatment delivery can be more prominent in trials investigating a complex intervention, such as surgery. Considering any potential difference in delivery at trial outset will ensure that any adjustments, as appropriate, can be made. The objective of this work was to establish current practice for the allowance of learning and clustering effects in the design and analysis of randomised multicentre trials.
**Methods:** A ten question survey was developed by the study team comprising open and closed questions that drew upon quotes from existing guidelines, references to relevant publications, and example trial scenarios. All registered UK Clinical Research Collaborative registered Clinical Trials Units were invited to participate.
**Results:** Completed surveys were obtained from 44 of 50 registered Units. Adjusting for learning by design through defining a minimum level of expertise for treatment provider was common (89%), although one third of units also had experience of expertise based designs. Managing clustering by design through stratification by centre was universally most common across the various trial types presented, and by treatment provider less so. Analysis of learning was rarely performed for the main analysis (n=1), although many units reported approaches to complement such analyses, such as sensitivity analyses. The majority of responders had indicated experience in adjusting during analysis for clustering, by centre or treatment provider, although approaches to doing so varied. Responders provided insight behind the approaches used within their unit and reasons for, or against, alternative approaches.
**Discussion:** This survey identifies widespread awareness of the potential methodological challenges associated with the design and analysis of multicentre trials, although approaches used and opinions on these vary.

## P-204

### An Independent Patient Data Meta Analysis to compare adjuvant therapies in patients suffering with Pancreatic Cancer

Rebecca Griffin[1], Eftychia-Eirini Psarelli[1], Paula Ghaneh[1], John Neoptolemos[2], Richard Jackson[1]
[1]Liverpool Clinical Trials Unit, Liverpool University, Liverpool, United Kingdom; [2]University of Heidelberg, Heidelberg, Germany
*Trials* 2019, **20(Suppl 1):**P-204

**Introduction:** Pancreatic Cancer is a disease with particularly poor prognosis with approximately 5% of patients surviving 5 years or more. Only 20% of patients are eligible for surgery and adjuvant therapy, but for those who do, prognosis improves with reported 5 year survival rates of 30% or more. A number of trials have explored the use of radiotherapy and chemotherapy although the best approach is still a matter of much discussion.
**Methods:** An independent patient data meta analysis of randomised controlled trials was performed using 3553 patients from 10 randomised controlled trials. Data are analysed using a Hierarchical Bayesian Network Meta Analysis incorporating frailty terms for patient nationality, modelling the baseline hazard function using a Piecewise Exponential Model and adjusting for trials with more than two therapies evaluated. Sub groups analysis are performed on consistent prognostic factors.
**Results:** An established network allows for comparisons of radiotherapy vs Chemotherapy Vs Observation only using direct and indirect evidence. Furthermore, networks allow direct and indirect comparisons of various types of chemotherapy. Subgroup analysis shows that the effect of therapy does depend on reported prognostic factors.
**Discussion:** IPD meta analysis improves the knowledge base of the effect of adjuvant therapies for resectable pancreatic cancer and identifies consistent prognostic factors which can help direct treatment decisions.

## P-205

### Joint Modelling for longitudinal measures of marker CA19-9 and survival data in patients with pancreatic cancer

Silvia Cicconi[1], Paula Ghaneh[1], John Neoptolemos[2], Ruwanthi Kolamunnage-Dona[1], Richard Jackson[1]
[1]University Of Liverpool, Liverpool, United Kingdom; [2]University of Heidelberg, Heidelberg, Germany
*Trials* 2019, **20(Suppl 1):**P-205

**Introduction:** Diagnosis of pancreatic cancer is increasing worldwide but its prognosis remain extremely poor even after resection. The ESPAC-4 trial showed that the adjuvant combination of gemcitabine and capecitabine improves overall survival compared to gemcitabine alone after resection for pancreatic ductal adenocarcinoma. CA19-9 concentration is a post-operatively established prognostic marker but little is known of its role in providing predictions of survival probabilities over time.
**Methods:** The relationship between longitudinal and time-to-event data was explored on 431 patients from the ESPAC-4 trial by means of joint modelling technique. A Cox proportional hazard regression was used for modelling survival data while linear mixed model was applied for CA19-9 follow-up measurements, incorporating random intercept and slope components.
**Results:** Joint modelling describes the evolution of the CA19-9 marker in time and provides an estimate of its association with time to death. Results show that CA19-9 decrease after surgery, reaching a plateau for the following months. However, the marker concentration spikes again with proximity to death.
**Discussion:** Joint modelling analysis improves the understanding of the latent association between CA19-9 marker and survival outcome, which can lead to the development of dynamic prognostic models to allow updated survival prediction of patients who undergone pancreatic ductal adenocarcinoma at any time following surgery.

## P-206

**Abstract omitted**

## P-207

### Joint modelling of multiple primary outcomes in clinical trials with missing data

Victoria Vickerstaff[1,2], Rumana Z. Omar[3], Gareth Ambler[3]
[1]Marie Curie Palliative Care Research Department, University College London, United Kingdom; [2]Research Department of Primary Care and Population Health, University College London, United Kingdom; [3]Department of Statistical Science, University College London, United Kingdom
*Trials* 2019, **20(Suppl 1):**P-207

**Introduction:** Multiple primary outcomes are sometimes specified in clinical trials. The outcomes may be of different types, say a mixture of survival and continuous outcomes. For example, in a trial investigating the effect of a health intervention on cannabis users, the primary outcomes may be the time to psychiatric relapse and the level of cannabis in the urine. These outcomes are typically associated and it's likely that at least one of the outcomes has missing values.
Joint models can be used to link survival outcomes with continuous outcomes and may provide better estimates of the intervention effect. The survival and continuous outcomes may be analysed using a survival model and a longitudinal model, respectively, which are linked by shared parameters. Another approach to link the models is to use joint random effects. This study evaluates the performance of joint models in terms of bias and efficiency of the estimated treatment effects. The results are compared to the estimates obtained when analysing the outcomes separately.
**Methods:** Several scenarios were investigated using simulation by varying the size of the association parameter joining the models and the level of missing data. Joint models which share parameters or have joint random effects were implemented using the R packages: JoineR, jointModel and FrailtyPack.
**Results:** The results show that when the outcomes are analysed separately, parameter estimation for the survival outcome is typically biased. The bias is reduced when using joint models. The joint models had increased standard errors for the estimated treatment effect on the survival outcome compared to analysing the outcomes separately.
**Discussion:** The joint random effects models and the models that utilised shared parameters performed best. These models produced the smallest mean square error of the estimated intervention effects on the survival outcome.

## P-208

### Real Time Continuous Glucose Monitoring in Neonatal Intensive Care (REACT): statistical challenges from the REACT trial

Annabel Allison[1], Simon Bond[1], Kathryn Beardsall[2], Catherine Guy[2], Beatrice Pantaleo[1], Lynn Thomson[2]
[1]Cambridge Clinical Trials Unit, Cambridge, United Kingdom; [2]Department of Paediatrics, School of Clinical Medicine, University of Cambridge, Cambridge, United Kingdom
*Trials* 2019, **20(Suppl 1):**P-208

**Introduction**: Increasing numbers of infants are being born preterm, requiring intensive care and with a high risk of mortality and morbidity as well as longer term health problems. Treatable neonatal causes of long term health problems have been difficult to establish but it is thought that early postnatal glucose control may be an important risk factor for clinical outcomes. In utero, glucose levels are maintained between 4-6mmol/l, but preterm infants are at risk of both hyperglycaemia (>8mmol/l) and hypoglycaemia (<2.6mmol/l).
The REACT trial aims to evaluate the efficacy, safety, utility, and cost-effectiveness of real time continuous glucose monitoring (CGM) in Neonatal Intensive Care.
**Methods, Timing of Potential Results:** REACT is an open-label, multi-centre, parallel group, randomised controlled trial comparing CGM with paper based algorithm to standard clinical management (control). The control group are managed according to standard practice using point of care blood glucose monitoring. These patients also have a sensor inserted and glucose data collected continuously but the clinical team are blinded to this data.
Sensor glucose (SG) is recorded every five minutes for up to one week. The primary outcome is the percentage of time that SG is in the target range of 2.6-10mmol/l within the first six days of life. The primary analysis is a linear regression model adjusted for randomisation strata (gestation and site). Results of the primary analysis, and a range of pre-specified secondary analyses, will be presented.
Results will be available in August 2019.
**Potential Relevance & Impact:** The secondary analyses will explore some of the statistical issues that are present in the data: varying amounts of data between patients due to sensor insertion and calibration issues (leading to different observation periods), within-patient correlation due to the time series nature of the data, and potential measurement error of CGM compared to blood glucose monitoring.

## P-209

### Review of reporting of time to event analyses and the proportional hazards assumption in meta-analysis

Ashma Krishan[1], Nicky Welton[2], Catrin Tudur-Smith[1]
[1]Department of Biostatistics, University of Liverpool, United Kingdom; [2]Population Health Sciences, University of Bristol, United Kingdom
*Trials* 2019, **20(Suppl 1):**P-209

**Introduction:** The most commonly used approaches for the analysis of time-to-event (TTE) outcomes impose an assumption of proportional hazards (PH), such that the hazard ratio (HR) is assumed to be constant over time. It is currently unknown what impact non-PH can have on overall conclusions and clinical decisions in meta-analysis. The objectives were to (i) review the reporting of meta-analysis of TTE outcomes that have assumed PH; (ii) describe the reporting of the PH assumption in meta-analysis of TTE outcomes and (iii) assess how often PH was valid or invalid in a sample of studies.
**Methods:** Eligible studies included systematic reviews (SRs) that included meta-analyses of randomised controlled trials (RCTs) of TTE outcomes which were included as a primary and/or secondary outcome, where SRs included phase II/III studies and RCTs were analysed using a PH model and where SRs were published between 2005 and 2015 in order to capture the most recent methods and allow the review to be manageable.
**Results**: 123 SRs including 956 RCTs were eligible for inclusion in our review. 35 (28%) of the reviews included aggregate data, 81 (66%) included individual patient data (IPD) and 7 (6%) included both IPD and aggregate data. Although all of the included SRs used methods that assume PH, only 33 (27%) SRs examined whether this was a reasonable assumption to make.
**Discussion:** Findings of this review demonstrate the poor reporting of the investigation of the PH assumption in meta-analyses of TTE outcomes. 73% (90 out of 123) SRs failed to adequately describe methods and results of appropriate approaches to assess the validity of the PH assumption. The poor reporting confirms that further work is needed to assess what impact the violation of the PH assumption can have on the statistical conclusions and clinical decisions in meta-analysis.

## P-210

### Simulation study of the impact of including hypothesis tests for differential biomarker cohorts in a comparative clinical trial in oncology

Marion Procter
Frontier Science Scotland (Ltd), Kincraig, Kingussie, United Kingdom
*Trials* 2019, **20(Suppl 1):**P-210

**Introduction:** Suppose in the context of metastatic breast cancer, the initial sample size calculations indicated 380 events at the primary analysis would provide 80% power to detect a hazard ratio (HR) of 0.75 at the 0.05 significance level. There would be 600 patients recruited 1:1 to active and control. Suppose external evidence suggested that there is a differential biomarker effect in the active arm, and therefore there will be hypothesis testing in the biomarker cohorts. Simulation was used to investigate the impact of the differential biomarker effect on the hypothesis testing.
**Methods:** Consider the following scenario for the HRs:
Overall cohort 0.75
Biomarker present cohort (40% of patients) 0.65
The null hypothesis for the biomarker present cohort (H01) and the biomarker absent cohort (H02) are tested at the 0.01 significance level. Alpha recycling applies to the null hypothesis for the overall cohort (H03), and H03 is tested at the 0.03, 0.04 or 0.05 significance level.
Simulation of 1000 datasets was used to estimate the impact if the primary analysis:
i)was at 420 events and the sample size was 600 patients (case 1)
ii)was at 450 events and the sample size was 700 patients (case 2)
**Results:** Under case 1, there were 162 events expected in the biomarker present cohort and the estimated power to reject H01 was 54%. The estimated power to reject H03 at the 0.04 significance level was 83%. Under case 2, the corresponding figures were 172 events, an estimated powers of 61% and 85% respectively.
**Discussion:** When designing a trial where there may be a noticeable differential biomarker treatment effect, consider taking this into account in the alpha-spending function and sample size decisions. Simulation can be used to investigate the impact of the differential biomarker effect.

## P-211

### Outcome assessment by central adjudicators in randomised stroke trials: simulation of differential and non-differential misclassification

Peter J Godolphin[1], Philip M Bath[2], Alan A Montgomery[1], NA on behalf of the Adjudicating Outcomes in Stroke Trials Collaboration NA[3]
[1]Nottingham Clinical Trials Unit, University of Nottingham, Nottingham, United Kingdom; [2]Division of Clinical Neuroscience, University of Nottingham, Nottingham, United Kingdom; [3]NA, group authorship, NA, NA
*Trials* 2019, **20(Suppl 1):**P-211

**Background:** Central adjudication of the primary outcome in randomised trials is thought to control misclassification. We investigated the amount of differential and non-differential misclassification needed before central adjudication alters the primary trial results.
**Methods:** We included data from five randomised stroke trials. Differential misclassification was introduced for each study until the confidence interval for the ratio of treatment effects (RTE, adjudication vs no-adjudication) did not include one. This was simulated 1000 times. We calculated the between-simulation mean proportion of participants that needed to be differentially misclassified before the RTE differed from one. In addition, we simulated hypothetical trials with a binary outcome and varying sample size (1000-10000), overall event rate (10-50%), and treatment effect (0.67-0.90). Non-differential misclassification was introduced until the true treatment effect was missed for each scenario.
**Results:** For the five trials, unweighted kappa was reduced from 0.89-0.97 to 0.65-0.85 before the RTE differed from one. This corresponded to 2.1%-6% of participants misclassified differentially for trials with a binary outcome. For the hypothetical trials, those with a larger sample size, stronger treatment effect and overall event rate closer to 50% needed a higher proportion of events non-differentially misclassified before a real treatment effect was missed.
**Discussion:** Central adjudication is important to control for differential misclassification in a clinical trial. However, for large blinded studies, extensive random noise is required before adjudication changes conclusions.

## P-212

### A better method to analyse modified Rankin Scale in the out-of-hospital cardiac arrest patients

Yujin Lee, Chen Ji
Warwick Clinical Trials Unit, Coventry, United Kingdom
*Trials* 2019, **20(Suppl 1):**P-212

**Introduction:** Modified Rankin Scale (mRS) is a clinically preferred 7-level ordinal scale to measure neurological status post cardiac arrest. Multiple methods have been developed to analyse this ranked outcome. Ordinal logistic regression is commonly used to provide an interpretable estimate for clinical audience. However, the proportional odds assumption is not always satisfied, rendering the conclusions from this model to become somewhat limited. Partial proportional odds regression overcomes this issue but leads to difficulty in interpretation. Alternative methods include 1) shift analysis: it tests the ranking of the scores, 2) binary regression: it breaks the levels into 2 categories using a clinically meaningful cut-off point. 3) sliding dichotomy dichotomises the score by accounting for patients' baseline prognostic risk, 4) multinomial regression ignores the orders and treats the score as nominal data and 5) linear regression.
**Method:** The aim of this analysis is to demonstrate the application of these methods using data in the PARAMEDIC-2 trial. Paramedic-2 is a multicentre double-blinded controlled trial of adrenaline versus placebo in the treatment of out-of-hospital cardiac arrest patients. The statistical methods will be applied to the mRS data collected at hospital discharge, 3 and 6 months post randomisation. The model fit and clinical interpretability of these methods will be compared along with the discussion of other advantages and disadvantages.

## P-213

### A weighted Cox model for the identification of predictive markers in oncology

Richard Jackson, Trevor Cox
Liverpool Cancer Trials Unit, Liverpool, UK
*Trials* 2019, **20(Suppl 1):**P-213

**Background:** The current trend towards personalised medicine has led to greater emphasis being placed on the ability to identify predictive bio-markers which can inform treatment decisions and aid in trial design. Methods for identifying predictive markers are often carried out on small populations and either rely on direct interaction effects between biomarkers and treatment or use dichotomisation to categorise continuous markers into clinically meaningful groups. The results of either approach can be misleading and have the problem of either misrepresenting the underlying relationship between a biomarker and a treatment effect or not making efficient use of limited resources.
**Methods:** We developed a method based on weighted cox regression models. Here, for each individual marker value, a vector of weights are generated based on marker proximity and 'local' hazard ratios comparing treatment arms obtained. Repeating across all marker values gives a continuous profile giving a clear description of how treatment effects differ based on biomarker information. Further, as the approach is based on Cox modelling, estimates of predictive markers are obtained which adjust for other prognostic factors.
**Results:** Applied to a dataset of patients with advanced Pancreatic Cancer, the weighted Cox model was able to identify a single marker which could be considered predictive in showing that the effect of randomised treatment varied depending on the biomarker value. Further, it also identified markers as having little impact which had previously thought to be predictive. Simulations show this approach less likely to identify spurious markers as being predictive.

**Conclusion:** A Weighted Cox approach to identifying predictive markers has the following benefits, makes efficient use of all data, can incorporate other pertinent prognostic information, allows for a visualisation of the direct relationship between treatment effects and marker values and is less likely to identify spurious markers than other approaches.

## P-214
### Abstract withdrawn

## P-215
### Maximising detection of mediated effects: information incorporation via Bayesian and Longitudinal mediation models

Kimberley Goldsmith[1], Milica Miocevic[3], Trudie Chalder[1], Rona Moss-Morris[1], Hazel Everitt[2]
[1]King's College London, London, United Kingdom; [2]University of Southampton, Southampton, United Kingdom, [3]Utrecht University, Utrecht, The Netherlands
*Trials* 2019, **20(Suppl 1):**P-215

**Introduction:** Many trials include mediation and moderation analyses to inform refinement and targeting of treatments. One example is the ACTIB trial of cognitive behavioural therapy (CBT) for irritable bowel syndrome (IBS) (1), which collected potential mediators and moderators in a repeated measures design. An earlier pilot trial of CBT for IBS was designed similarly (2). These studies provide an ideal opportunity to apply rarely employed mediation methods: Bayesian and fully longitudinal mediation models.
**Methods:** Trial outcomes and mediators were measured at baseline and three post-randomisation time points both in the pilot CBT for IBS and ACTIB trials. We fitted Bayesian mediation models using blavaan in R calling the Just Another Gibbs Sampler (JAGS) software and longitudinal latent change mediation models using Mplus (3). Negative illness perception was explored as a mediator of the effect of CBT on the work and social adjustment outcome.
**Results:** Incorporation of prior/pilot information provided similar mediated effect estimates as compared to those using the ACTIB trial data alone (indirect/mediated effect estimates -2.1 vs -2.5), but these estimates were more precise (95% credible intervals -1.1 to -3.2 versus -1.4 to -3.6). Similarly, fully modelling longitudinal mediator and outcome processes, and assuming equal mediator – outcome estimates at all points, gave 2-fold and more than 3-fold more precise estimates as compared to modelling a single measure each of the mediator and outcome.
**Conclusions:** The availability of pre-main trial pilot data and repeated measures designs help provide robust answers to the question: does the treatment work? Such designs also allow us to flexibly and more precisely address important secondary mediation questions, providing good quality empirical treatment refinement guidance. We will explore whether combining Bayesian and longitudinal methods brings further gains.

#### References
1. Everitt et al, 2019; 10.1136/gutjnl-2018-317805.
2. Moss-Morris et al, 2010; 10.1017/S0033291709990195.
3. Goldsmith et al, 2019; 10.1037/met0000154.

## P-216
### Comparisons of different concentration–response models in the thorough QT studies

Huanyuan Luo[1], Jorg Taubel[2,3], Tao Chen[1], Duolao Wang[1]
[1]Liverpool School of Tropical Medicine, Liverpool, United Kingdom; [2]Richmond Pharmacology Limited, London, United Kingdom; [3]St George's University of London, London, United Kingdom
*Trials* 2019, **20(Suppl 1):**P-216

**Background:** The concentration–response modelling has been used a primary analysis tool in the thorough QT (TQT) studies to detect "threshold of regulatory concern", a drug-induced effect on the QTc (QT interval corrected by heart rate) beyond a limit of 10 msec. Different statistical methods have been used to model the concentration–response relationship. In this study, we aim to compare those models in terms of their statistical performances and parameter interpretations.
**Methods:** The commonly used concentration–response/effect models include linear mixed-effects models, Emax models, sigmoidal Emax models, log-linear models, and polynomial models (power function). This study will compare those models using real TQT studies as well as simulated datasets and assess the statistical performances in terms of model convergence, predictive accuracy, type I error and power, and sensitivity to model mis-specifications, as well as the interpretation of parameters.
**Timing of Potential Results:** We have the real TQT studies datasets so far. The literature review, statistical analysis and simulations are ongoing. The results will be ready by the end of September 2019.
**Potential Relevance & Impact:** Advantages and disadvantages of applications of different concentration–response models in TQT studies will be explored and relevant recommendations will be discussed regarding the design, analysis and reporting of TQT studies in the drug development.

## P-217
### Using expert opinions to inform subgroup analyses in clinical trials: example of a bayesian analysis of the VeRDiCT trial

Russell Thirard[1], Raimondo Ascione[2], Chris Rogers[1]
[1]Clinical Trials and Evaluation Unit, School of Clinical Sciences, University of Bristol, United Kingdom; [2]Bristol Heart Institute, University of Bristol, Bristol, United Kingdom
*Trials* 2019, **20(Suppl 1):**P-217

**Introduction:** Typically, subgroup analyses in clinical trials are conducted by comparing the treatment effect in each subgroup by means of an interaction test. However, trials are rarely, if ever, adequately powered for interaction tests, so clinically important interactions may go undetected. Within a Bayesian framework, expert opinion can be used to inform the characterisation of the interaction parameter. The motivating example for applying this methodology is the VeRDiCT trial investigating the effect of preoperative volume replacement therapy (VRT) in diabetic patients undergoing cardiac surgery. Two subgroup effects were of clinical interest: a) preoperative blood glucose control (oral medication and/or insulin) and b) preoperative renal function (high vs low risk of renal failure).
**Methods:** Clinical experts (cardiac surgeons and cardiac anaesthetists) were identified within one of the two trial centres. Questionnaires, designed to elicit opinions on the impact of VRT on early outcome (time until medically fit for hospital discharge post-surgery) in the different subgroups, were delivered face-to-face and as part of a routine research meeting. The main and interaction effects were elicited using unconditional and conditional questions respectively. A 'community of priors' as well as a robustness of results to various variance derivations are investigated in a Bayesian Cox proportional hazard model using the STAN software in R.
**Timing of Potential Results:** Analyses will be undertaken when the collection of clinical opinions ends in May 2019. The results should be available by July 2019.
**Potential Relevance & Impact:** The study results will quantify the effect of VRT for specific subgroups of patients, providing estimates of the probability of benefit or harm while accounting for expert views on the intervention (e.g. optimistic, sceptical, interaction priors) and the trial data. The methodology applied in this example could be applied in other trials with planned subgroup analyses.

## P-218

### A comparison of statistical methods to compensate for missing data in longitudinal cluster-randomised controlled trials

Courtney McDermott[1], Mary Codd[1], Ricardo Segurado[1], Barbara Dooley[2]
[1]University College Dublin, School of Public Health, Physiotherapy, and Sports Science, Ireland; [2]University College Dublin, School of Psychology, Ireland
*Trials* 2019, **20(Suppl 1):**P-218

**Introduction:** Clinical trials are the preferred method in the evaluation of medical interventions. However, missing data causes loss of power and may introduce biases, potentially leading to researchers over- or underestimating intervention effects. This is a particular issue in studies where data are correlated, such as in longitudinal cluster-randomised controlled trials (LCRCTs). Despite advances in statistical methods, many researchers choose to simply exclude cases with any missing values from the analysis (complete case analysis).
Multiple Imputation (MI) and Structural Equation Modelling (SEM) are two sophisticated statistical techniques that may provide unbiased corrections in compensating for missing data. The aim of this research was to compare the performance of MI and SEM in compensating for missing data in LCRCTs using computer-simulated datasets. The performance of these methods was also compared to a common method used to analyse longitudinal data, mixed linear modelling (MLM).
**Methods:** Simulated datasets were generated to imitate data from a real LCRCT. Data from the outcome measures were deleted at 5%, 10%, and 20% and then analysed using the three aforementioned methods, in addition to complete case analysis (CCA). Missingness was introduced by three mechanisms: missing completely at random (MCAR); missing dependent on covariate x (MAR); and missing dependent on outcome value (MNAR).
**Results:** Regardless of missingness mechanism, MI, SEM, and MLM provided similar, unbiased results. SEM and MLM produced the least biased results, though the SEM generated the smallest standard errors, therefore recovering more of the lost sample size. CCA produces the largest standard errors and most biased results.
**Conclusions:** From the results of these simulations, MLM and SEM are the preferred methods to compensate for missing data in LCRCTs. These two techniques are able to recover most of the lost sample size, and therefore researchers are less likely to miss important intervention effects.

## P-219

### Treatment effect adjusting for baseline covariates: a curious case of selection bias

Arijit Sinha
Roche Products Limited, Welwyn Garden City, United Kingdom
*Trials* 2019, **20(Suppl 1):**P-219

**Introduction:** In early phase often we have single arm trials with small patient population to generate evidence for treatment effect. A common approach in lack of randomized control arm is to compare outcome from treatment arm with potentially similar historical control arm adjusting for baseline covariates. Although we can minimize bias coming from the list of covariates at hand we may have unwanted factors which can potentially bias the treatment effect.
**Methods:** We will look into a case study from a Phase 2 trial. Treatment effect adjusting for baseline covariates using propensity scores and using adjusted Cox regression model will be demonstrated. Subsequent analysis with other factors in the model will be provided.
**Results;** Adjusted covariate analysis suggests significant treatment effect and potential planning for Phase 3 trial. However, subsequent analysis including other covariate in the model has raised questions about the magnitude of treatment benefit.
**Discussion:** Exploratory data analysis with statistically sound methodology can generate evidence of treatment effect from single arm trials. However, safeguarding against potential confounders is an issue. At the end we have to make decision based on risk benefit ratio. But questions should be raised and explored thoroughly to kill a drug before a fully planned Phase 3 trial is initiated.

## P-220

### Simple correction of admixed RNA samples using cancer purity information

Jules Hernández-sánchez
Roche Products Ltd, Welwyn Garden City, United Kingdom
*Trials* 2019, **20(Suppl 1):**P-220

**Introduction:** RNA expression data are very common in clinical research. In cancer, tissue samples, e.g. bone marrow aspirates, are usually a mixture of healthy and cancerous cells. The proportion of both types of cells varies across individuals. Downstream analyses are therefore affected by this source of error contributing to a reduction in power and increase in bias of parameter estimation. A simple tumor content correction is proposed to render RNA values more highly correlated with true levels of gene expression in cancer.
**Methods:** Assume the following simple admixture model:

$$E = pE\_c + (1-p)\ E\_n$$

,where E is the total RNA level for a given gene, e.g. log2(TPM*), Ec is the expression among cancer cells, and En is the expression among normal cells, and p is purity or cancer content in a 0-1 scale. A re-arrangement of the equation above leads to:

$$E = E\_n + (E\_c - E\_n\ )p = a + bp$$

, which is equivalent to a simple linear regression with intercept a (expected expression in normal cells), and slope b (expected differential expression –DE-); which after adding a model error e (residuals after regressing E onto p) would render E=a+bp+e. The proposed correction is:

$$E\_c \cong a + b + e$$

**Results:** Simulation work showed that power to detect DE is reduced when sample purity decreases, but that power remained high after adjusting for purity differences. The method corrects equally well regardless of the levels of DE. There is some prediction bias that can reduced by selecting only samples with higher purities.
**Discussion:** All downstream analyses using RNA data, e.g. DE, gene signatures, prognostic modelling etc, would experience an increase in power and reduction in bias after using this correction in admixed cancer samples.

## P-221

### Abstract withdrawn

## P-222

### A new instrument to assess the credibility of effect modification analyses (ICEMAN) in randomized controlled trials and meta-analyses

Stefan Schandelmaier[1,2], Matthias Briel[1,2], Ravi Varadhan[3], Christopher H Schmid[4], Niveditha Devasenapathy[5], Rodney A Hayward[6], Joel Gagnier[7], Michael Borenstein[8], Geert JMG van der Heijden[9], Issa Dahabreh[4], Xin Sun[10], Willi Sauerbrei[11], Michael Walsh[2], John PA Ioannidis[12], Lehana Thabane[2], Gordon H Guyatt[2]
[1]University Hospital Basel, Basel, Switzerland; [2]McMaster University, Hamilton, Canada; [3]Johns Hopkins University, Baltimore, USA; [4]Brown University, Providence, USA; [5]Indian Institute of Public Health - Delhi , New Delhi, India; [6]University of Michigan School of Medicine, Ann Arbor, USA; [7]School of Public Health, University of Michigan, Ann Arbor, USA; [8]Biostat Incorporation, Englewood, USA; [9]University of Amsterdam, Amsterdam, The Netherlands; [10]Chinese Evidence-Based Medicine Center, Sichuan University, Chengdu, China; [11]University of Freiburg, Freiburg, Germany; [12]Meta-Research Innovation Center, Stanford University, Stanford, USA
*Trials* 2019, **20(Suppl 1):**P-222

**Introduction:** Most randomized controlled trials (RCTs) and meta-analyses examine effect modification (also called subgroup effects or interaction) in which the effect of an intervention varies by another variable (the effect modifier). Assessing the credibility of an apparent effect modification presents challenges. Therefore, we aimed to develop an instrument for assessing the credibility of effect modification analyses (ICEMAN) in an RCT or meta-analysis of RCTs.
**Methods:** Following a stepwise process, we 1) developed a detailed concept; 2) identified candidate credibility considerations in a systematic survey of the literature; 3) together with leading experts, performed a consensus study to identify key considerations and develop them into instrument items; and 4) refined the instrument based on feedback from trial investigators, systematic review authors, and journal editors who applied drafts of ICEMAN to published claims of effect modification.
**Results:** The final instrument consists of a set of preliminary considerations, core questions (five for RCTs, eight for meta-analyses) with four response options, one optional item for additional considerations, and a credibility rating on a visual analogue scale ranging from very low to high credibility. An accompanying manual provides rationale, detailed instructions, and examples from the literature. Seventeen potential users tested the instrument. Implementing their suggestions improved the user-friendliness of the new instrument.
**Discussion:** ICEMAN is a rigorously developed instrument to assess the credibility of apparent effect modification. The instrument may aid trial investigators, systematic reviewers, journal editors and others who are interpreting or considering making a claim of effect modification.

## P-223

### On the need to adjust for multiplicity in confirmatory clinical trials with master protocols

Nigel Stallard[1], Susan Todd[2], Deepak Parashar[1,3,4], Peter K Kimani[1], Lindsay A Renfro[5]
[1]Warwick Medical School, University of Warwick, Coventry, United Kingdom; [2]Department of Mathematics and Statistics, University of Reading, Reading, United Kingdom; [3]The Alan Turing Institute, London, United Kingdom; [4]Warwick Cancer Research Centre, University of Warwick, Coventry, United Kingdom; [5]Division of Biostatistics, University of Southern California, Los Angeles, United States of America
*Trials* 2019, **20(Suppl 1):**P-223

**Introduction:** Recent advances in tumour biology and targeted therapies have led to clinical trials considering treatment effects in multiple subgroups of a patient population. These can lead to efficiency gains by testing several hypotheses in the same clinical trial. Proposed approaches include adaptive enrichment, umbrella and basket trial designs. Although much of the development of novel designs has been in exploratory phase II trials, there is growing interest in the application of such methods in confirmatory randomized controlled trials.
In a confirmatory trial setting, as the multiple hypothesis tests can lead to statistical error rate inflation, questions arise regarding the need for correction for multiplicity. The recent US FDA draft guidance on Master Protocols notes the risk of “potential overinterpretation of findings”, but offers no clear suggestions as to when statistical correction for multiplicity should be implemented.
**Methods:** We survey novel master protocol designs and explore the resulting multiplicity issues. A basic principle is that correction for multiplicity is needed in confirmatory trials if testing of multiple hypotheses increases the opportunity to claim of effectiveness for an experimental treatment.
**Results:** Based on consideration of when subgroups result in multiple opportunities for a claim of effectiveness for a treatment, we make a proposal for when multiplicity corrections are needed. We differentiate between different master protocol designs on the basis of the role of the subgroups included.
**Discussion:** Conventionally, correction is required when multiple hypothesis tests are conducted in a trial, but not when they are conducted in separate trials. This position has arisen mainly in simpler settings than those now being developed, and the use of innovative trial designs requires a reconsideration of multiplicity issues. There seems to be little current consensus on this question and this work provides a contribution to ongoing discussion.

## P-224

### Clinical utility of the EMPiRE prediction model using Decision Curve Analysis (DCA)

John Allotey[1], Borja Fernandez[2], Javier Zamora[1,2], Khalid Khan[1], Shakila Thangaratinam[1]
[1]Queen Mary University of London, United Kingdom; [2]Unidad De Bioestadística Clínica. Hospital Ramón Y Cajal, Spain
*Trials* 2019, **20(Suppl 1):**P-224

**Introduction:** Usual statistical methods for evaluating prediction models are based on assessing their performance by discrimination and calibration. These do not incorporate information on model utility, so a precise model is not always a clinically useful model. The EMPiRE model was developed to predict risk of seizures in pregnant women taking antiepileptic drugs at any time in pregnancy and until 6weeks postpartum. We also evaluated the clinical utility of the model using DCA.
**Methods:** We fitted the model using LASSO regression and used datasets of the prospective EMPiRE cohort study of 527 pregnant women with epilepsy to develop and validate the prediction model. We evaluated the net benefit of using the model with various probability thresholds to aid in clinical decision-making for individualized patient ‘treatment’ options, where the preferred strategy is the one with the highest net benefit at any given threshold.
**Results:** The EMPiRE model showed good performance in terms of discrimination 0.79 (95% CI 0.75,0.84) and calibration 0.93 (95% CI 0.44,1.41). The highest net benefit was observed between predicted probability thresholds of 12% and 99%. We did not recommend a specific decision threshold as this would likely vary with the user (healthcare professional or pregnant woman), intervention offered, potential adverse effect associated with the intervention and the intervention cost. Use of the model would reduce the number of women incorrectly offered the intervention by at least 2, and up to 54 of every 100 woman when the decision threshold is high.
**Discussion:** The EMPiRE model can be used to predict seizure risk in pregnant women with epilepsy on medication at any time during pregnancy and up to 6weeks postpartum. It has good predictive performance, and usefulness in clinical practice. Use of the model will facilitate decisions about the optimal management of these women customized to their risk profile.

## P-225

### Designing a study to evaluate the impact of the PREP models for predicting complications in early onset pre-eclampsia

John Allotey, Ratna Sohanpal, Chiamaka Esther Amaefule, Khalid Khan, Shakila Thangaratinam
Queen Mary University of London, United Kingdom
*Trials* 2019, **20(Suppl 1):**P-225

**Introduction:** The use of prediction models in healthcare aims to improve clinical decision-making by providing patient-specific risk of future outcome. The PREP study developed and externally validated prediction models for complications in early-onset pre-eclampsia. To improve health outcomes, model predictions need to affect the decision-making of clinicians. Draft NICE guidelines for Hypertension in pregnancy recommend the PREP model for use in clinical practice, however it is important to assess whether the model will likely be used by healthcare professionals and patients and if its use is likely to have a positive effect on clinical decision and patient outcome.
**Methods:** Our mixed method study interviewed healthcare professionals and women with previous pregnancies complicated by pre-eclampsia to understand how predicted risks were perceived and how these influenced their decision-making. This information was also used to arrive at thresholds for various treatment options based on predicted risk probabilities, and decide on the format of prediction model results and its interpretation when being assessed in a large-scale impact study.
**Results:** The predicted threshold for admission to hospital set by NICE in the draft guideline (≥30%) was conservative, compared to that identified by healthcare professionals (>40%) and women (>45%). The knowledge obtained on the perspective of the healthcare professionals and women, will influence the best way to present the model when incorporated in the design of an impact assessment study.
**Discussion:** A phased approach is needed to assess the impact of a prediction model (such as the PREP model) in clinical practice. Decision Curve Analysis should first be performed to identify the clinical utility of the model, before progression to a large-scale cluster randomized impact study. This will at least indicate the potential effectiveness of the model. Results from phases of this research process can help in optimizing the design of any future impact study.

## P-226
**Abstract omitted**

## P-227
### Bayesian Statistics in the design and analysis of cluster randomised controlled trials and their reporting quality: a methodological systematic review

Benjamin Gary Jones[1], Adam Streeter[1], Amy Baker[1], Rana Moyeed[2], Siobhan Creanor[1,3]
[1]Medical Statistics, Faculty of Health: Medicine, Dentistry and Human Sciences, University of Plymouth, Plymouth, United Kingdom; [2]School of Computing, Electronics and Mathematics, Faculty of Science and Engineering, University of Plymouth, Plymouth, United Kingdom; [3]Peninsula Clinical Trials Unit, Faculty of Health: Medicine, Dentistry and Human Sciences, University of Plymouth, Plymouth, United Kingdom
*Trials* 2019, **20(Suppl 1):**P-227

**Introduction:** In a Cluster Randomised Controlled Trial (CRCT), randomisation units are "clusters" such as schools or GP practices. This has implications for design and statistical analysis, since clustering often leads to correlation between observations which, if not accounted for, can lead to spurious conclusions of efficacy/effectiveness. Bayesian methodology offers a flexible, intuitive framework to deal with such issues. This review aimed to explore and quantify the use of Bayesian methodology in the design and analysis of CRCTs and appraise the quality of reporting against relevant published CONSORT guidelines.
**Methods:** The protocol for this systematic review was prospectively published (https://osf.io/2azrc/). We sought to identify all reported/published CRCTs incorporating Bayesian methodology, as well as papers reporting developments of new Bayesian methodology. We searched Medline, Embase and the Cochrane Library Central Register of RCTs. The initial sift of titles and abstracts, full text reviews and data extraction were undertaken twice, independently.
**Results:** Twenty-seven publications were included, six from an additional hand search. Eleven (40.7%) were reports of CRCT results: seven (25.9%) were primary results papers and four (14.8%) papers reported secondary results. Thirteen papers (48.1%) reported Bayesian methodological developments, the remaining three (11.1%) compared different methods. All eleven results papers used Bayesian methodology in analysis, none did so in design/sample size calculation. Of the seven primary results papers, none clearly accounted for clustering in sample size calculation, but six (85.7%) clearly did in the analysis.
**Discussion:** The popularity of the CRCT design has increased in the last twenty years and whilst there has been some effort to develop Bayesian methodology for the design and analysis of CRCTs, uptake of these modern methods remains low. There is an opportunity to further develop Bayesian methodology in the context of CRCTs in order to expand the accessibility, availability and, ultimately, use of this approach.

## P-228
### Statistical approaches to adjust for the use of rescue medication in randomised controlled trials

Anca Maria Chis Ster, Victoria Cornelius, Suzie Cro
Imperial College London, London, United Kingdom
*Trials* 2019, **20(Suppl 1):**P-228

**Introduction:** The use of rescue medication is often allowed in trials in chronic disease to aid recruitment and retention of participants in addition to ethical considerations. Use of rescue medication in addition to study treatment will impact the treatment effect estimate, especially if there is unequal use between arms. It can therefore be of value to obtain a treatment effect estimate adjusted for the use of rescue medication. There are no existing best-practice approaches recommended to calculate adjusted estimates. We aimed to identify what approaches are currently employed to describe and adjust for rescue medication use. We undertook a systematic review of randomised controlled trials (RCTs) in asthma and eczema to ascertain this.
**Methods:** We searched for original phase II-III monoclonal antibody drug RCTs in asthma and eczema. A standardised, pre-specified checklist was used to extract data on trial characteristics, reporting of rescue use, and methods of analysis to adjust for rescue medication on the primary outcome.
**Results:** We identified 59 trials and ascertained that 57 (97%) allowed for rescue medication in the trial but only 28 (47%) articles reported its use. Twenty six (44%) articles summarized rescue use by arm and 9 (15%) included an analysis on the primary outcome adjusting for rescue medication. Eight of these 9 trials used a sub-optimal approach of single imputation, such as last observation carried forward (LOCF) after setting post-rescue data to missing, 4 (44%).
**Discussion::** Rescue medication use is not commonly reported. There is evidence of sub-optimal statistical practice that could lead to bias being employed when undertaking rescue adjusted analyses. Guidance on accessible statistical approaches to adjust for post-randomisation variables, such as rescue use, would support better practice.

## P-229
### Designing early phase studies for individual participant data (IPD) meta-analysis (MA)

Rebecca Playle[1], Polyxeni Dimitropoulou[1], Rachel McNamara[1], David Linden[2], Vincent Poile[1], Gareth Watson[1], Kerry Hood[1]
[1]Cardiff University, Cardiff, Wales; [2]Maastricht University, Maastricht, The Netherlands
*Trials* 2019, **20(Suppl 1):**P-229

**Introduction:** BRAINTRAIN was a European consortium study designed to develop and test the feasibility and efficacy of neurofeedback for the treatment of mental and behavioural disorders.

**Methods:** The clinical studies of BRAINTRAIN were conducted as single-centre trials but under an overarching conceptual and design framework. A total of 7 small individual feasibility studies were completed in 5 countries. Four of the studies were randomised and three were single group studies for proof of principle. Since the trials were in 5 different clinical conditions (Alcohol dependence, PTSD, Autism, Anxiety and Obesity) and had differing clinical outcomes, standardised effects from each trial were compared. Common psychometric outcome measures to assess depression, mood and anxiety across disorders, and measures to predict neurofeedback success were agreed with study teams. A common data platform designed with input from the consortium members facilitated data sharing. Two stage, random effects IPD MA across trials was carried out for the randomised studies. The studies in Autism and Anxiety used a single arm design, therefore within group change effects calculated from each trial were used to carry out an aggregate data (AD) meta-analysis.
**Results:** Feasibility criteria were met by all studies for recruitment, retention and intervention uptake (>50% approached eligible; 50-80% of those eligible recruited; 50-80% retention at the primary endpoint; 60-90% uptake). There were positive effects of neurofeedback on PTSD symptom scores and for depression scores in the Alcohol Dependence trial. Change scores indicated promising effects in the Autism and Anxiety studies.
**Conclusions:** Meta-analysis is usually only carried out on completed definitive randomised trials, however it has been used here, by design, to combine and synthesise the results of small feasibility studies to give an overview of the potential effects of neurofeedback in various clinical con

## P-230

### Evaluation of the effectiveness of an incentive strategy on the questionnaire response rate in parents of premature babies. A randomised controlled Study Within A Trial (SWAT) nested within SIFT

Ed Juszczak[1], Christopher Partlett[2], Louise Linsell[1], Catherine Rounding[1], Jon Dorling[3], Madeleine Claire Hurd[1], Oliver Hewer[1]
[1]National Perinatal Epidemiology Unit, Nuffield Department of Population Health, University Of Oxford, Oxford, United Kingdom; [2]Nottingham Clinical Trials Unit, University of Nottingham, Nottingham, United Kingdom; [3]IWK Health Centre, Halifax, Canada
*Trials* 2019, **20(Suppl 1):**P-230

Introduction: Loss to follow-up compromises the validity of trial results by reducing statistical power, negatively affecting generalisability and undermining assumptions made at analysis, leading to potentially biased and misleading results. Evidence exists that incentives are effective at improving response rates, but there is little evidence as to the best approach, especially in the perinatal field. The NIHR-funded SIFT trial (ISRCTN76463425) follow-up of infants at two years of age provided an ideal opportunity to resolve this remaining uncertainty.
Methods: Participants: all infants from participating neonatal units in the UK and Ireland followed up for SIFT (multicentre RCT investigating two speeds of feeding in babies with gestational age at birth < 32 weeks and/or birthweight <1,500g). Interventions: parents were randomly allocated to receive incentives (£15 gift voucher) before or after questionnaire return. The objective was to establish whether offering an unconditional incentive in advance, or promising an incentive on completion of a questionnaire (conditional) improved the response rate in parents of premature babies. The primary outcome was questionnaire response rate. Permuted block randomisation was performed (variable size blocks), stratified by SIFT allocation (slower/faster feeds) and single/multiple birth. Multiple births were given the same incentives allocation. Parents were blinded to their incentives allocation, however this was not possible for the SIFT office staff.
Results: 923 infants were randomised: 459 'infants' allocated to receive incentive before, 464 'infants' allocated to receive incentive after; analysis was by ITT. An incentive before led to a significantly higher response rate, 83% (381/459) compared to the after group, 76.1% (353/464); adjusted absolute difference of 6.8% (95% CI 1.6% to 12.0%).
Conclusions: An unconditional incentive in advance led to a significantly higher response rate compared to the promise of an incentive on completion. Against a backdrop of falling response rates to questionnaires, incentives are an effective way to increase returns.

## P-231

### Accounting for treatment heterogeneity in systematic reviews of trials of complex interventions

Alexandra Wright-hughes[1], Rebecca Walwyn[1], Amanda Farrin[1], David Cotrell[2]
[1]Clinical Trials Research Unit, Leeds Institute of Clinical Trials Research, University of Leeds, Leeds, United Kingdom; [2]Leeds Institute of Health Sciences, University of Leeds, Leeds, United Kingdom
*Trials* 2019, **20(Suppl 1):**P-231

**Introduction:** Meta-analyses of RCTs conducted in the context of a systematic review aim to synthesise evidence across RCTs, similar in population, outcome and intervention, by quantifying the pooled treatment effect, accounting for differences between RCTs. Standard aggregate-data methods that account for treatment heterogeneity include random-effects meta-analysis (the average treatment effect is estimated, assuming a distribution of treatment effects across RCTs); subgroup analysis (separate treatment effects are estimated for each subgroup of RCTs); and meta-regression (sources of heterogeneity are included as study-level effect modifiers). Complex interventions introduce further sources of between and within-study heterogeneity as they comprise multiple, potentially interacting components, with complicated causal pathways, and meditators and moderators of treatment effect. Variability may exist at the patient-level due to intervention adherence or tailoring, and at the provider-level due to intervention delivery.
**Methods:** Using a planned individual-patient-data (IPD) meta-analysis of complex interventions to reduce self-harm in adolescents (RISA-IPD), we will discuss the benefits and challenges of using an IPD approach to handling treatment heterogeneity at the study, provider and patient levels in systematic reviews of complex interventions.
**Timing of Potential Results:** The review is in set-up and anticipated to include IPD for at least 19 RCTs; the analysis plan is expected to written by October 2019.
**Potential Relevance & Impact:** IPD meta-analyses are recognised as a way of explaining treatment heterogeneity at the patient-level (including patient-level, in addition to study-level, effect modifiers in a meta-regression). They provide greater scope and flexibility in analysis and the ability to appropriately handle treatment-related clustering associated with intervention delivery. However, there is little consideration or guidance in the current literature on how to allow for provider-level modifiers of treatment effects and limited experience of handling treatment-related clustering associated with providers. Methods for identifying study, provider and patient-level mediators within IPD meta-analyses are also unclear.

## P-232

### Sample size of four clusters-per-arm as a rule of thumb for pilot cluster-randomised controlled trials

Jen Lewis, Steven Julious
Scharr, University of Sheffield, Sheffield, United Kingdom
*Trials* 2019, **20(Suppl 1):**P-232

**Introduction:** There currently exists little guidance on sample size for pilot cluster-randomised controlled trials (cRCTs). Previous work shows that parameter estimates from such studies, particularly the ICC, are imprecise and problematic to use in subsequent sample size calculations. We performed a systematic review to describe trends in pilot cRCT sample size and illustrate the typical imprecision in ICC estimation. We also aimed to explore the changing precision of ICC estimates with increasing sample size, and establish the impact on main trial power when imprecise ICC estimates are used to design a main trial.
**Methods:** We searched PubMed and Web of Science for papers published between 2010-2017 with the terms 'pilot' or 'feasibility' in the title or topic, supplemented with terms to identify CRTs. We extracted sample sizes and other key information including estimation of the ICC. Swiger's formula was utilised to explore the precision and impact of ICC estimates generated from different pilot sample sizes.
**Results:** 574 studies were returned. 81 studies were included in the final review. These had a median of 4 planned clusters per-arm (IQR: 3,7) and 77 planned participants per-arm (IQR: 40, 240). The precision of ICC estimates was highly varied with 95% CIs ranging from less than 0.1 to more than 1. Analysis showed minimal gains in precision when using more than 8 total clusters. However, inaccurate ICC estimates can yield power of over 50% in a main trial when used in a sample size calculation.
**Discussion:** The trend of 4 clusters-per-arm is consistent with the minimum number of clusters suggested for pilot cRCTs in previous work. We have shown this sample size to be a reasonable minimum for such studies. Understanding the impact on power of utilising imprecise ICC estimates in sample size calculations will assist researchers in designing main trials.

## P-233

### Exploring challenges in trials with surgical versus non-surgical intervention comparators: a qualitative evidence synthesis

Loretta Davies[1], David Beard[1], Jonathan Cook[1], Andrew Price[1], Francine Toye[2]
[1]University Of Oxford, Oxford, United Kingdom; [2]Oxford University Hospitals NHS Foundation Trust, Oxford, United Kingdom
*Trials* 2019, **20(Suppl 1):**P-233

**Introduction:** Randomised controlled trials in surgery can be difficult to design and conduct, especially when including a non-surgical comparison. Only around half of initiated surgical trials reach their recruitment target, and failure to recruit is cited as the most frequent reason for premature closure of surgical RCTs.
The aim of this qualitative evidence synthesis was to identify, and synthesise findings from qualitative studies exploring the challenges in the design and conduct of trials directly comparing surgical and non-surgical interventions.
**Methods:** A qualitative evidence synthesis using meta-ethnography was conducted. Six electronic bibliographic databases were searched up to the end of February, 2018. Studies that explored patients' and health care professionals' experiences regarding participating in RCTs with a surgical and non-surgical comparison were included. The GRADE-CERQual framework was used to rate confidence in review findings.
**Results:** 3,697 abstracts and 49 full texts were screened and 26 published studies reporting experiences of patients and healthcare professionals were included. Five themes related to challenges to these types of trials were identified: 1. Radical choice between treatments; 2. Patients' discomfort with randomisation: best treatment for me as an individual; 3. Achieving balance: challenge of exploring patients' a priori preferences for treatment.4. Clinicians' conflict with equipoise: strong speciality convictions and 5. Imbalanced presentation of interventions and 'buy-in' of clinical specialities.
**Discussion:** The marked dichotomy between the surgical and non-surgical interventions was identified in this review as making the conduct of these types of trials particularly challenging. Consideration of these five specific challenges should be made in the planning and design of future studies of this type of comparison to optimise the delivery of these particular trials.

## P-234

### Systematic review of prospective studies comparing different monitoring strategies in clinical intervention studies

Katharina Klatte[1], Sharon B Love[2,3], Matt R Sydes[2], Hannah Ewald[4], Pascal Benkert[1], Nicole Bruni[1], Patricia Arnaiz[1], Christiane Pauli-Magnus[1], Matthias Briel[1]
[1]Department Of Clinical Research University Hospital Basel, Basel, Switzerland; [2]Institute of Clinical Trials & Methodology, University College London, London, United Kingdom; [3]Centre for Statistics in Medicine, Nuffield Department of Orthopaedics, Rheumatology and Musculoskeletal Sciences, University of Oxford, Oxford, United Kingdom; [4]University Medical Library, University of Basel, Basel, Switzerland
*Trials* 2019, **20(Suppl 1):**P-234

**Introduction:** Trial monitoring is requested by Good Clinical Practice (GCP) guidelines to ensure safety and rights of study participants as well as data quality. Recent developments at international bodies and regulatory agencies have supported the need for risk-based and centralised approaches to trial monitoring. Several studies have empirically assessed the effectiveness of such alternative monitoring approaches with discrepant findings. The objective of the present systematic review is to comprehensively summarise the benefits and disadvantages of different monitoring strategy for prospective intervention studies.
**Methods:** We submitted a protocol to the Cochrane Methodology Review Group and are currently systematically searching MEDLINE, EMBASE, and CENTRAL from their inception to May 3, 2019, for all prospective studies comparing different monitoring strategies in intervention studies. Two authors will independently assess the methodological quality of eligible studies using the Cochrane Risk of Bias tool and extract information on a number of key study characteristics using pre-piloted data collection forms. The primary outcome will be the number of critical and major monitoring findings as defined by the European Medical Association. Secondary outcomes will include patient recruitment and retention rates, and resource use. We will quantitatively pool results if appropriate.
**Timing of Potential Results:** Our electronic search yielded 3497 hits. Title and abstract screening is currently ongoing. There are at least five eligible studies evaluating different monitoring strategies (ADAMON, OPTIMON, TEMPER, START, MONITORING). At the time of the conference we will be able to present comprehensive results from this systematic review.
**Potential Relevance & Impact:** Given the large heterogeneity of monitoring practices among research institutions, a guideline for clinical studies describing and quantifying benefits and disadvantages of specific monitoring strategies is urgently needed. A new consensus might evolve on how on-site monitoring could be efficiently and judiciously used and tailored by central monitoring findings.

## P-235

### Bayesian Adaptive Design in Phase III Clinical Trials: A Gap between Theory and Practice

Sidong Li, Yang Wang, Yanyan Zhao, Wei Li
State Key Laboratory of Cardiovascular Disease, Fuwai Hospital, National Center for Cardiovascular Diseases, Peking Union Medical College & Chinese Academy of Medical Sciences, Beijing 100037, China., Beijing, China
*Trials* 2019, **20(Suppl 1):**P-235

**Introduction:** Bayesian methods for adaptive designs have been developed in these years due to its flexibility, but it was reported to be poorly used in practice especially in phase III clinical trials. Therefore, we aimed at describing the current development of Bayesian adaptive designs and the actual use in clinical settings.
**Methods:** We conducted a bibliometric study by using PubMed and Science Citation Index-Expanded databases to review the methodology development for Bayesian adaptive designs and actual use in Phase III clinical trials. And we also extracted the features of Bayesian methods in clinical practice including the characteristics of publications, trial characteristics and statistical attributions to demonstrate the most urgent need for methodologies.
**Timing of Potential Results:** 30 August 2019
**Potential Relevance & Impact:** Although there is an increase in using of Bayesian adaptive designs in this decade, most of these references are still from the biostatistical teams all over the world and less was transformed from theory into practice. Our results would indicate the current situation of Bayesian adaptive designs in phase III clinical trials, for example, most clinical trials are using non-informative priors and few earn the benefits of Bayesian method such as trial stopping early and sample size decreasing. It is still necessary for statisticians to conduct more efficient designs and demonstrate its real benefit in action.

## P-236

### Missing Tumor Measurement (TM) Data in the Search for Alternative TM-based Endpoints in Cancer Clinical Trials

Sumithra Mandrekar[1], Fang-Shu Ou[1], Jun Tang[3], Ming-Wen An[2]
[1]Mayo Clinic, Rochester, United States; [2]Vassar College, New York, United States; [3]University of Iowa, Iowa City, United States
*Trials* 2019, **20(Suppl 1):**P-236

**Introduction:** Missing data commonly occur in clinical trials and may hinder the search for alternative cancer clinical trial endpoints. We consider reasons for missing tumor measurement (TM) data in cancer clinicaltrials (CCT) and how missing TM data are typically handled. We explore the potential impact of missing TM data on the predictive ability of a set of tumor-measurement based endpoints.
**Methods:** Literature review identifies reasons for and approaches to handling missing TM data. Data from 3 actual clinical trials were used as an illustrative case study. A sensitivity analysis of the potential impact of missing TM data was performed by comparing the predictive ability of alternative endpoints to overall survival (OS) using observed data and imputed data.
**Results:** We documented several reasons for missing TM data in CCT. Although missing TM data impacted individual objective status (e.g. 12- week status changed for 53% of patients in one imputation set), it surprisingly only minimally impacted predictive ability of the endpoints on OS (e.g. pointwise c-indices ranged from 0.56-0.60 for N9741, 0.55- 0.63 for N9841, and 0.51-0.66 for N0026, across observed and imputed datasets).
**Discussion:** By understanding the reasons for missingness, we can better anticipate them and possibly minimize their occurrence. Our analysis suggests missing TM data may not impact predictive ability of endpoints but may impact classification of objective status. Given that response status is still a routine phase II endpoint in the development of new cancer therapies (including immunotherapy), we urge that complete TM data collection and following protocol disease evaluation as closely as possible in cancer clinical trials be a priority.

## P-237

### Evaluation of importance of international collaboration and data provision consistency: a case study (LI1 trial)

Ian Thomas[1], Mike Dennis[2], Robert Hills[3], Selman Mirza[1], Laura Upton[1]
[1]Cardiff University, Cardiff, United Kingdom; [2]The Christie NHS Trust, Manchester, United Kingdom; [3]University of Oxford, Oxford, United Kingdom
*Trials* 2019, **20(Suppl 1):**P-237

**Introduction:** The Less intensive-1 (LI1) trial is a study in 1000 elderly acute myeloid leukaemia (AML) patients not suitable for intensive chemotherapy, comparing novel agents against standard treatment using a Pick-a-Winner design. Designed and sponsored in the UK, LI1 includes strong international collaboration. The contribution of these international collaborations has not previously been evaluated.
**Methods:** Novel agents are added to the protocol via substantial amendment and collaborative groups must seek national approval before supporting a new protocol version. Technical, regulatory and practical detail relating to novel agents may result in differences in support provided by collaborative groups. Trial opened in UK December 2011; first patient randomised January 2012.
**Results:** On 30 Jan 2019, 952 patients randomised. 70 patients (7.4%) randomised from 6 sites in New Zealand, 52 (5.5%) from 4 sites in Denmark; collaborations with other national groups have provided an additional 11 (1.2%) patients.
5.7% of patients in the vosaroxin-only arm (3 Denmark, 0 New Zealand, 53 total), 7.5% of patients in the LDAC+vosaroxin arm (2 Denmark, 2 New Zealand, 53 total) ensured data was reported to Data Monitoring Committee 5-14 months early, dependent on randomisation arm.
Published data in the control group demonstrates equivalence of primary endpoint (complete remission, CR, rate across trial 12%, Denmark 13%, New Zealand 11%). Survival data, provided by sites, has been entered within median 55 days (inter-quartile range, IQR 42-90 days) – UK (55 days (IQR 43-98 days)), Denmark (47 days (IQR 35-61 days)) and New Zealand (48 days (IQR 35-74 days)).
**Discussion:** Supportive international collaborations have accelerated protocol accrual by 12 months over 8 years. Trial conduct and patient outcome appears equivalent across the collaborative groups suggesting no degradation in data quality/consistency. With future studies increasingly evaluating the diverse sub-populations of AML such international collaborations will remain important to facilitate timely delivery.

## P-238

### A proposed review of selected clinical trial protocols and publications to better understand the inadequate reporting of safety data

Genevieve Helen Wills, Alejandro Arenas-Pinto, David Dunn, Ruth Goodall
MRC Clinical Trials Unit at UCL, London, United Kingdom
*Trials* 2019, **20(Suppl 1):**P-238

**Introduction:** The CONSORT statement for harms was introduced to improve reporting of safety related data from randomised controlled trials (RCT). Based on trials publications, it has been found that adherence to the CONSORT guideline for harms was poor, statistical methods were inadequately reported and varied between trials, and safety data were not being fully utilised. Adequate reporting of safety information was found to occur in less than 30% of trials. It is important to understand whether these deficiencies are due to inadequate consideration of safety data at the design stage (reflected in the protocol and CRFs, for example) or at the analysis/publication stage.
**Methods:** A collection of recent prominent phase III HIV and TB treatment trials was identified using a clinical trials registry and key meta-analysis publications. All publications (primary and secondary) have been identified and trial protocols requested. A datapoint extraction checklist has been created, based on the CONSORT statement for harms along with additional items to gather more in-depth information. Available information from protocols and publications will be assessed against these criteria.
**Timing of Potential Results:** By September 2019 all analyses will be completed.
**Potential Relevance & Impact:** To our knowledge, this will be the first piece of research on safety data reporting that considers trial protocols and publications in conjunction. This should help to identify the point(s) in the trial process when improvements could be implemented.

## P-239

### The Use of Serious Adverse Event Data in Safety Analyses

Elizabeth Catherine James[1], Adrian Cook[1], Matthew Sydes[1], David Dunn[1], Andrew Clamp[2,3]
[1]MRC CTU at UCL, London, United Kingdom; [2]The Christie NHS Foundation Trust, Manchester, United Kingdom; [3]University of Manchester, Manchester, United Kingdom
*Trials* 2019, **20(Suppl 1):**P-239

**Introduction:** Clinical trial safety data are collected as both routinely-collected, time-specified adverse events (AEs) – usually gathered by populating a pre-defined list - and serious adverse events (SAEs), collected when sporadically occurring. However, these events are not necessarily mutually exclusive. This was the case in ICON8, an international phase III trial which explored the efficacy of three different chemotherapy regimens for ovarian cancer. The trial protocol stipulated that SAEs on the pre-defined AE list should also be reported as AEs. We investigated whether this was always followed.
**Methods:** Text searches of SAE data identified events from the pre-defined list which should have been included in AE-led safety analyses. Categorisable SAEs were then mapped to AEs according to pre-defined criteria: (a) SAE date matches AE assessment date +/-30 days; (b) AE assessment date between SAE onset and resolution dates; (c) Events occurred during same chemotherapy cycle. Safety analyses were repeated using the combined dataset.
**Results:** 150 patients from ICON8 were included (first 50 patients randomised to each arm). Pooling AEs and SAEs not additionally reported as AEs during the safety analysis period increased the number of toxicity events by 76 from 4583 to 4659. The proportion of patients with ≥1 G3+ AE increased in all arms; control: 10% (from 32% to 42%), experimental -1: 4% (from 56% to 60%), exeperimental-2: 10% (from 46% to 56%). Difference in proportion of patients with ≥1 G3+ AE vs control was unaffected by data pooling: experimental-1 from 24% (5%, 43%) to 18% (-1%, 37%); experimental-2 from 14% (-5%, 33%) to 14% (-5%, 33%), none breaching the pre-specified 15% lower bound threshold.
**Discussion:** Events that meet both AE and SAE reporting criteria should be accounted for when performing safety analyses. Mapping is feasible and important. Methods for accurately pooling the data should be further explored.

## P-240

### Lessons learned: evaluation of a centralised system of safety blood monitoring within a multicentre randomised placebo-controlled clinical trial

Holly Ennis, Catriona Keerie
Edinburgh Clinical Trials Unit, Usher Institute, University of Edinburgh, Edinburgh, United Kingdom
*Trials* 2019, **20(Suppl 1):**P-240

**Introduction:** Within placebo-controlled drug trial designs, concealing treatment allocation adequately while ensuring the robust collection and interpretation of blood safety data can present significant methodological challenges. This evaluation assesses the use of a centralised safety blood monitoring system employed within the TOPPIC trial, a randomised, placebo-controlled trial assessing whether mercaptopurine (MP) prevented or delayed recurrence of Crohn's disease following surgical resection.
**Methods:** Treatment with MP causes bone marrow suppression leading to leukopenia and thrombocytopenia and, less commonly, to anaemia. Consistent with clinical practice, all randomised participants in both MP and placebo arms underwent regular full blood count monitoring. A centralised blood monitoring system was implemented whereby blood samples were collected and processed at participating sites, with results transferred to the central trial office by independent staff members for entry into a web-based electronic data capture (EDC) system. Blood values were reviewed by a blinded team of clinical assessors, with queries and decisions fed back to site via the EDC system.
**Results:** 240 participants were recruited to the TOPPIC Trial and 4,994 (67.1%) of the total projected number of 7,440 safety blood tests were collected during the trial. Of the 4,994 collected bloods, 4,396 (88.0%) were collected within the target window with a median number of days either side of the target date of 0. Compliance within the target window for bloods taken at GP visits was higher at 3,089 (90.9%) than those taken at scheduled study visits (81.8%). A total of 5,088 (15.9%) sets of blood results resulted in abnormal value alerts with a median review time by clinician assessors of 1 day.
**Discussion:** The TOPPIC Trial demonstrated that a centralised masked system of safety blood monitoring can be both feasible and efficient within a multi-site trial. The anticipated volume of messages generated should be considered when developing future systems.

## P-241

### Provisioning, Support and Training in UK Academic Multicentre Trials

Matt Hammond[1], Claire West[1], Matt Nankivell[2]
[1]Norwich Clinical Trials Unit (UEA), Norwich, United Kingdom; [2]University College London, London, United Kingdom
*Trials* 2019, **20(Suppl 1):**P-241

**Background:** Despite substantial research in trial conduct there remains a lack of guidance for UK Clinical Trials Units (CTUs) in terms of support for clinical sites in multicentre trials. Through surveys and interviews this study assessed how different CTUs provide sites with materials, training, and support prior to and during trials, and how this is perceived by NHS staff to identify areas of unmet need.
**Methods:** CTUs were surveyed to obtain an understanding of current practice. A subsequent survey was distributed to research staff at 43

NHS Trusts to obtain perceptions of CTU practice. Finally semi-structured interviews were performed with NHS staff to discuss CTU support and areas for improvement.

**Results:** Conflicts between NHS staff preference and current practice were identified, particularly in training during the trial. In both the survey (82%) and interviews NHS staff perceived that there was an over-emphasis on recruitment. NHS staff registered a moderate or strong preference for on-site initiation training (91%), although only 50% of CTUs often or always offered this. Similarly, during the trial NHS staff preferred in-person (64%) training, although most CTUs did not offer this (67%).

In many areas CTU practice reflected the preferences of NHS research staff. Regular updates from the CTU trial team were identified as important in research staff interviews, and by 95% of NHS survey respondents. Most CTU staff (86%) sent regular updates, with the median being at least quarterly. Common themes identified in staff interviews included the importance of prompt data querying.

**Conclusions:** The study identified divergence between CTU practice and preferences of NHS staff, the clearest of which was between NHS staff wanting regular, onsite training and the general CTU drive to deliver training remotely. The study highlighted multiple themes that could be explored through future "study within a trial" projects to improve trial efficiency.

## P-242

### Trial data access: the trials and tribulations of implementing a new approach within a CTU

Victoria Emma Yorke-Edwards, Sharon B. Love, Sarah Lensen, Carlos Diaz-Montana, Mahesh K.B. Parmar, Matthew R. Sydes

MRC Clinical Trials Unit at UCL, University College London, United Kingdom

*Trials* 2019, **20(Suppl 1):**P-242

**Introduction:** The responsible sharing of data generated by clinical trials and research studies is an ethical obligation, one that a growing number of funders now mandate. However, data sharing raises various practical issues for Clinical Trials Units (CTUs) around the mechanisms for data sharing: demonstrating adequate participant consent; ensuring the visibility of metadata, and the clarity of request processes; the preparation of shareable datasets, and the timing of this; resourcing data preparation, and the review process; and maintaining adequate understanding of datasets over time. The MRC CTU at UCL has employed a unit-wide controlled access approach, with applications reviewed by individual Trial Steering Committees (TSCs) but is now exploring an alternative approach.

**Methods:** This poster provides a case study of the implementation of a new approach to data sharing within a CTU. It details the specific issues faced, including those presented by historical trial data. It then discusses how changes are being made to the data access process to resolve these issues.

**Timing of Potential Results:** An identified approach is under review based on a single unit-wide Data Access Committee, servicing data sharing post-publication into the medium term, with potential alternative approaches to accessing trial data much later in each trial's life. This approach will be finalised before October 2019.

**Potential Relevance & Impact:** Data sharing is becoming an increasingly important issue for CTUs. This case study provides a useful view of the issues of opening up access to clinical trial and research data and a potential blueprint for other CTUs to follow.

## P-243

### What kinds of things matter for judging how well a trial site is performing?

Zoë Skea[1], Shaun Treweek[1], Julie Turzanski[2]

[1]Health Services Research Unit, University of Aberdeen, United Kingdom; [2]University of Nottingham, United Kingdom

*Trials* 2019, **20(Suppl 1):**P-243

**Introduction:** Site performance is key to the successful delivery of large multicentre randomised trials. We report findings from an initial qualitative component of a wider study (Whitham et al 2018) which aimed to identify and agree a core set of key performance metrics and create a simple reporting tool for managing multicentre trials.

**Methods:** We undertook focus groups in three UK cities with stakeholders (32 participants) holding a range of roles within clinical trial contexts. Discussions focussed on views about the idea of monitoring trial site performance and the kinds of things that might matter in terms of judging how well a site is performing (and why and from whose perspective). Discussions were recorded, transcribed and analysed thematically.

**Results:** Participants were supportive in principle of the need for performance assessment. The process of measuring performance was regarded as being multi-dimensional in that performance could potentially vary across individuals within a site; across one site overall; and across different sites within the same trial. In terms of what might matter when gauging how well an individual trial site (or a trial more generally) is doing, there was consistency across the groups that three 'core' aspects were crucial: Recruitment; retention; and the quality of outcome data collected (although there was recognition that the importance of these might vary depending on specific trial context). Participants also discussed a range of issues that would likely impact on a site's ability to perform well across the three core aspects identified (e.g. quality of staff relationships etc).

**Discussion:** Participants identified three crucial aspects of trial performance and a range of indicators that are likely to feed into these and which could allow judgements to be made about how well a site is likely to perform. This informed the development of our site performance metric tool.

## P-244

### Digital Tools for More Efficient Conduct of RCTs: Trials Unit Survey

Jacqueline Nuttall[1], Athene Lane[2], Amanda Blatch-Jones[1], Gareth Griffiths[1], Jermey Wyatt[1]

[1]University of Southampton, Southampton, United Kingdom; [2]University of Bristol, Bristol, United Kingdom

*Trials* 2019, **20(Suppl 1):**P-244

**Introduction:** Recruitment of participants to, and their retention in, Randomised Controlled Trials (RCTs) is a key determinant of research efficiency, but is challenging. Digital tools and media are increasingly used to reduce costs, waste and delays in the conduct and delivery of research. The aim of this UK Clinical Trials Unit (CTU) survey was to identify which digital recruitment and retention tools are being used to support RCTs, their benefits and success characteristics.

**Methods:** A survey was sent to all UK Clinical Research Collaboration (UKCRC)-registered CTUs with a webinar to help increase completion. A logic model and definitions of a "digital tool" were developed by iterative refinement by project team members, the Advisory Board (NIHR Research Design service, NHS Trust, NIHR Clinical Research Networks and patient input) and CTUs.

**Results:** A total of 24/52 (46%) CTUs responded, 6 (25%) of which stated no prior use. Database screening tools (e.g. CPRD, EMIS) were the tool most widely used (10/22 45%) for recruitment and were considered very effective (7/10 70%). The most mentioned success criteria were saving GP time and reaching more patients. Social media was second (6/22 27%), but estimated effectiveness varied considerably, with only 17% stating very effective. Fewer retention tools were used, with SMS / email reminders reported most (10/15 67%), but certainty about effectiveness varied. A table of potential digital tools to support recruitment and retention tasks, with examples and a logic model showing relationships between the resources, activities, outputs and outcomes for digital tools were developed.

**Discussion:** Database screening tools are the most commonly used digital tool for recruitment, with clear success criteria and certainty about effectiveness. Our detailed definition of what constitutes a digital tool, with examples, will inform the NIHR research community about choices and help them identify potential tools to support recruitment and retention.

## P-245

### Lessons learnt from a multi-centre Type 3 surgical trial

Katie Biggs[1], Daniel Hind[1], Mike Bradburn[1], Lizzie Swaby[1], Steven Brown[2]
[1]University Of Sheffield, Sheffield, UK; [2]Sheffield Teaching Hospitals NHS Trust, Sheffield, UK
*Trials* 2019, **20(Suppl 1):**P-245

**Background:** Pragmatic randomised controlled trials are increasingly being used to evaluate surgical interventions, although they present particular difficulties in regard to recruitment and retention.
Methods
This paper details the procedures and processes related to implementation of a multi-centre pragmatic surgical randomised controlled trial.
**Results:** Forecasting consent rates based on previous similar trials ensured that the recruitment window was of adequate length. Adequate resource was available for study procedures at multiple clinics in each hospital due to micro-costing of study activities with research partners ensure. A video was produced targeting recruiting staff, which aimed to help recruiters explain the trial, randomisation and equipoise, based on methodological work and experiences from another study. Post-randomisation delays in delivering surgery to one study arm were investigated by assessing the outcomes at the time of randomisation and the day of surgery which provided confidence in the baseline measure. Real-time monitoring of participant drop-out due to delays in surgery meant we were able to extend the recruitment window in a timely fashion. Triangulation of data sources ensured adequate numbers of participants provided primary outcome data.
**Discussion:** This paper provides a range of evidence- and experience-based approaches which resulted in meeting our study's objectives and these lessons may be transferable.

## P-246

### Challenges in the design, planning and implementation of trials evaluating group interventions

Katie Biggs[1], Daniel Hind[1], Rebecca Gossage-Worrall[1], Kirsty Sprange[2], David White[1], Jessica Wright[1], Robin Chatters[1], Katherine Berry[3], Diana Papaioannou[1], Mike Bradburn[1], Stephen J Walters[1], Cindy L Cooper[1]
[1]ScHARR, University of Sheffield, Sheffield, UK; [2]NCTU, University of Nottingham, Nottingham, UK; [3]School of health Sciences, University of Manchester, Manchester, UK
*Trials* 2019, **20(Suppl 1):**P-246

**Background:** Evaluating group interventions in randomised controlled trials (RCTs) presents a set of practical problems which may not be immediately obvious and are not present in RCTs of one-to-one interventions.
Methods
Case-based approach summarising Sheffield clinical trial unit's experience in the design and implementation of group interventions across five randomised controlled trials.
**Results:** Median recruitment across the five trials was 5.8 (range 2.1-16.0) participants per site per month. Group intervention trials can involve a delay in the start of treatment whilst waiting for sufficient numbers to start a group. There was no evidence in our trials that the timing of consent, relative to randomisation, affected post-randomisation attrition, but attrition was a concern for all trial teams. Group facilitator attrition was common in studies where facilitators were employed by the health-system rather than the by the grant holder, and lead to the early closure of one trial. Solutions to this included training 'back-up' and new facilitators. Trials specified that participants had to attend a median of 62.5% (range 16.7% to 80%) of sessions, in order to receive a 'therapeutic dose'; a median of 75.0% (range 34.6% to 97.8%) received a therapeutic dose. Across the five trials, 66.4% of all sessions ran with fewer than the numbers pre-specified as ideal. A variety of methods were used to assess the fidelity of group interventions across the five trials.
**Discussion:** Investigators should expect delays / difficulties in recruiting groups of the optimal size, plan for both facilitator and participant attrition and consider how group attendance and group size affects treatment fidelity.

## P-247

### Delivering site set-up training to groups of sites versus individually: a randomised study within a trial

Eleanor Mitchell[1], Alan Montgomery[1], Garry Meakin[1], Rachel Haines[1], Reuben Ogollah[1], Chris Partlett[1], Kate Walker[1], Jon Dorling[2], Shalini Ojha[3], on behalf of the FEED1 Collaborative Group
[1]Nottingham Clinical Trials Unit, University of Nottingham, Nottingham, United Kingdom; [2]Dalhousie University, Halifax, Canada; [3]Division of Graduate Entry Medicine & Medical Sciences, University of Nottingham, Nottingham, United Kingdom
*Trials* 2019, **20(Suppl 1):**P-247

**Introduction:** Site initiation visits (SIVs) are conducted to deliver training to sites before opening them to recruitment, though this can be burdensome during the time-intensive trial set-up period. There is little evidence about the best way to deliver training for sites to perform well. Evaluating methods of training was the highest priority identified at a workshop exploring recruitment and retention of participants to trials. Two systematic reviews investigating training in clinical trials showed a variety of different training methods and more research is needed to determine what kind of training and support can improve recruitment. A small retrospective study showed that, whilst face-to-face training (either at SIV or group training session) was associated with better recruitment than remote training (i.e. telephone or DVD), no difference was seen between the two types of face-to-face training.
Our objective is to compare group-based training during the trial set-up period versus visiting the site to conduct a SIV to investigate the impact of the training method upon key site performance metrics. We will embed a SWAT into the FEED1 trial, funded by the NIHR HTA programme.
**Methods:** Once selected, sites will be randomised in batches to receiving their site-initiation training during a SIV or group-based training by attending a collaborators' meeting. To allow for non-availability of site staff, two meetings will be held. Outcomes will include recruitment and retention, data quality and protocol compliance (defined as core site performance metrics) and associated costs of each training method.
**Timing of potential results:** Clinical trial and SWAT results will be available in Q1 of 2023.
**Potential relevance and impact:** If the intervention is shown to be effective, there could be significant benefits to funders and trial teams, in particular reducing the length of time it takes to set-up a trial and open all sites.

## P-248

### Making a challenging trial possible: Lessons from the Emergency Department led EcLiPSE trial

Kerry Woolfall[1], Louise Roper[1], Mark. D Lyttle[4], Carrol Gamble[5], Amy Humphreys[5], Shrouk Messahel[3], Elizabeth Lee[3], Joanne Noblet[3], Helen Hickey[5], Naomi E.A Rainford[5], Anand Iyer[2], Richard Appleton[2]
[1]University of Liverpool, United Kingdom; [2]Neurology Department Alder Hey Children's NHS Foundation Trust, United Kingdom; [3]Emergency Department, Alder Hey Children's NHS Foundation Trust, United Kingdom; [4]Emergency Department, Bristol Royal Hospital for Children, United Kingdom; [5]University of Liverpool, Clinical Trials Research Centre (CTRC), United Kingdom
*Trials* 2019, **20(Suppl 1):**P-248

**Background:** Challenges to the successful conduct of 'The Emergency treatment with Levetiracetam or Phenytoin in Status Epilepticus in children (EcLiPSE)' trial were identified at the pre-trial stage. These included practitioner anxieties about conducting research without prior consent (RWPC), inexperience in conducting an Emergency Department (ED) led trial and use of a medication that was not usual ED practice. As part of an embedded study (the Consent Study) we explored parent and practitioner experiences of involvement in the EcLiPSE trial to inform the design and conduct of future ED led trials.
**Methods:** A mixed-method study involving questionnaires and interviews with parents of randomised children, interviews and focus groups with EcLiPSE practitioners and audio recorded trial discussions.
**Results:** A total of 143 parents (93 mothers, 39 fathers, 11 missing information) of randomised children completed a questionnaire and 30 (25 mothers, 5 fathers) were interviewed. We analysed 76 recorded trial discussions. Ten practitioners (4 medical, 6 nursing) were interviewed, 36 (16 medical, 20 nursing) participated in one of six focus groups. Practitioner anxieties around RWPC and changes to usual practice were addressed by: experience of trial recruitment, including positive responses from parents; a clinically important question; simple pragmatic trial design; and strong leadership. Lack of leadership at a few sites negatively affected staff engagement and recruitment. EcLiPSE completed on time, achieving its required sample size target.
**Conclusions:** Our study provides valuable insight into factors that helped to facilitate successful recruitment and conduct of a challenging emergency department led trial. Findings should be used to inform the design and conduct of future trials in this setting.

## P-249

### Considerations when implementing a new biological sample collection in an open clinical trial

Steven Penegar[1], Rebecca Lewis[1], Andrew Feber[4], Hugh Mostafid[2], Margaret A Knowles[3], Emma Hall[1]
[1]The Institute of Cancer Research, London, United Kingdom; [2]Royal Surrey County Hospital NHSFT, Guildford, United Kingdom; [3]University of Leeds, Leeds, United Kingdom; [4]University College London, London, United Kingdom
*Trials* 2019, **20(Suppl 1):**P-249

**Introduction:** CALIBER (NCT02070120) is a phase II study investigating intravesical chemoablation as an alternative to surgery for recurrent low risk non-muscle invasive bladder cancer. Eighty-two participants were recruited from 24 NHS sites between January 2015 and September 2017. A full sample collection could not be implemented from the outset due to budget restrictions. CALIBER-T was launched in October 2016, after securing additional funding to collect blood, tissue and urine samples for translational analysis.
**Methods:** Addition of CALIBER-T required:
•Additional regulatory approvals
•Amended site agreements
•Implementation of sample tracking system
•Streamlining of sample collection within existing patient pathways
•Reconsent of participants who joined the trial prior to implementation of CALIBER-T
•Additional central and local staff resources
Teleconferences were held with sites to discuss sample collection objectives, local patient pathway management and to promote engagement. A system for per-patient reconsent reminders to sites was setup. Systems were established with the collaborating laboratories to enable sample reconciliation, whilst maintaining patient confidentiality.
**Results:** 18/24 CALIBER sites, at which 58/82 CALIBER participants were randomised, agreed to participate in CALIBER-T. To May 2019, 24 blood samples had been received. This is lower than originally anticipated, despite reminding sites to reconsent, due to participant scheduling issues and limited site capacity for reconsenting.
**Discussion:** Recommendations
Sample collection should be embedded from the outset of a trial if possible. Participating centres should be encouraged to actively engage with the aims of the translational study to help facilitate collection of a robust sample set.
**Conclusion:** The value of implementing additional biological sample collection during a trial can outweigh the challenges encountered in its introduction, however it requires thorough evaluation, careful integration with existing systems and the support of key stakeholders to ensure that sample collection can be implemented effectively.

## P-250

### Case study of implementation of an urgent safety measure

Lee Priest[2], Hilary Critchley[3], Lee Middleton[2], Maxwell Feltham[2], Mary Ann Lumsden[4], Jane Daniels[1]
[1]University of Nottingham Clinical Trials Unit, Nottingham, United Kingdom; [2]University of Birmingham Clinical Trials Unit, Birmingham, United Kingdom; [3]University of Edinburgh, MRC Centre for Reproductive Health, Edinburgh, United Kingdom; [4]University of Glasgow, Reproductive and Maternal Medicine, Glasgow, United Kingdom
*Trials* 2019, **20(Suppl 1):**P-250

**Introduction:** UCON is an open-label randomised trial comparing ulipristal acetate (UPA) with the levonorgestrel-releasing intra-uterine system in women with heavy menstrual bleeding. In February 2018, the European Medicines Agency (EMA) issued a drug alert for UPA. An urgent safety measure (USM) was implemented, which included suspension of recruitment and UPA use.
We describe the impact of the USM on trial timelines.
**Methods:** Women take UPA for 12 weeks followed by 4 weeks off-treatment, for 3 courses. The participant-reported primary outcome is collected after the final off-treatment week. The USM allowed women to complete a course, if they wanted, but not start a subsequent course. The original sample size 220 women including 20% loss to follow-up: 198 participants had been randomised when recruitment was suspended. On resumption, the UPA group could complete 3 courses, subject to monthly blood tests.
**Timeline**
8/2/18 EMA issues drug alert with temporary recommendations that no new patients start UPA. Urgent teleconference amongst Trial Management Group.
12/2/18 Urgent teleconference with MHRA safety scientist. Implementation of USM. Notifications to MHRA and ethics committee.
13/2/18 Sites notified and template letter for participants provided.
26/2/18 Substantial amendment submitted.
18/5/18 EMA update allows restricted UPA resumption.
8/18 Clinician and patient surveys to determine commitment to recruitment and acceptability of monthly blood tests for UPA group.
7/8/18 MHRA allows UPA resumption. Restart amendment submitted.
21/08/18 Recovery plan submitted to funder. Approved 10/9/18.
18/10/18 Trial reopens to recruitment.
**Impact:** Sample size had to be increased by 84 to replace participants on study during USM. An extra 12 months of recruitment is required, at a cost of £199,505. Average recruitment in 5 months before USM was 8.4/month, now 2.4/month since resumption.
**Discussion:** Following a USM necessitating suspension of recruitment, prompt and coordinated action is required. Nevertheless, the impact on the trial can be substantial.

## P-251

### Automation of clinical trial statistical monitoring

Laura Collett, Eleanor Gidman, Chris Rogers
University Of Bristol, Bristol, United Kingdom
*Trials* 2019, **20(Suppl 1):**P-251

**Introduction:** Regular monitoring of clinical trial data is an essential component of trial conduct to ensure data are being collected to a high standard, intervention(s) are being administered according to the protocol and are safe. Automated reports of data compliance, protocol adherence and safety, tailored to the needs of the trial, are an efficient way of providing study teams with detailed information to monitor progress and guide decision-making.
**Methods:** Trial monitoring has been largely automated with the use of modular programming, control flow and configuration files, which allow database extracts to be validated and imported, appropriate datasets to be derived, and specific charts, tables and listings to be produced using the output delivery system in SAS. The input data and output summaries required (e.g. whether summaries are required for the study as a whole, by site or by intervention), are handled by optional initialisation parameters. Capabilities also include calling Stata and R from SAS, to best use the strengths of each software for specific and tailored monitoring; error handling; saving of log files; and automatic backup of data extracts.
**Results:** Use of a modular framework has improved code maintenance, extension, readability and speed. Code repositories are adapted to different trials, with development time reducing for each successive initiation. The methodology is being successfully applied to four large multicentre trials run through the clinical trials unit, with reports being produced regularly or ad hoc.
**Discussion:** The upfront investment in statistical programming time to design and set-up the automated monitoring system has been significant, but overall time burden has reduced and the benefit to trial teams has been substantial. Potential further developments include email notification of completed batch tasks, including alerts of errors encountered.

## P-252

### Central monitoring of clinical trials: a survey of current practice

Holly Emily Mckeon, Lucy Culliford, Rachel Brierley, Jessica Harris, Maria Pufulete, Hana Tabusa, Abby Willcox, Chris Rogers
University Of Bristol, Bristol, United Kingdom
*Trials* 2019, **20(Suppl 1):**P-252

**Introduction:** Monitoring site performance in multicentre studies is vital to inform the trial management group about trial conduct, arising issues, data quality, recruitment and retention. Onsite monitoring may be conducted periodically to verify study documentation and data collected for the study against source data. Remote central monitoring is conducted using manual and automated reviews of data submitted to online study databases. Increasingly, clinical trials units (CTUs) are using remote central monitoring of multicentre studies in preference to routine onsite monitoring, to improve efficiency. Remote central monitoring methods vary between studies. The aim of this study is to identify which data items are frequently monitored centrally, by what methods and the criteria for triggering onsite monitoring across a range of studies.
**Methods:** We designed an online survey using SurveyMonkey and are intending to send it to trial managers responsible for approximately 60 ongoing studies run through our clinical trials unit. The survey will take approximately 10 minutes to complete and will collect data on the following: central monitoring methods, data items monitored (e.g. percentage eligible patients consented, protocol compliance), effectiveness of central monitoring, how issues are identified and actions recorded (e.g. via email, minutes) and triggers for onsite monitoring (e.g. repeated serious breaches).
**Timing of Potential Results:** Results of this initial survey will be available in June 2019.
**Potential Relevance & Impact:** The results will be used to refine the survey before disseminating it to CTUs nationally. The data will be used to improve methods for remote central monitoring to ensure that it is effective in identifying data quality and conduct issues at sites.

## P-253

### Do consent procedures differ when recruiting outside of the UK versus within the UK: data from the international Tranexamic acid for hyperacute primary IntraCerebral Haemorrhage (TICH-2) study

Lisa Jane Woodhouse, Philip M Bath, Nikola Sprigg
University Of Nottingham, Nottingham, United Kingdom
*Trials* 2019, **20(Suppl 1):**P-253

**Background:** TICH-2 was an international randomised controlled trial of whether tranexamic acid halts haematoma expansion and improves outcome. Stroke treatments typically have greater efficacy if given early and so delays should be avoided. However, obtaining consent in the emergency situation is difficult since many stroke patients lack capacity. Here we assess whether consent procedures were affected by recruiting country.
**Methods:** Ethics approval was obtained to allow full informed consent or verbal assent (using a brief information sheet; BIS) followed by full written consent at a later date. The BIS was used when the therapeutic time window was short and the use of full written consent would inhibit recruitment into the trial. Where patients lacked capacity, approval for enrolment was obtained with permission from a relative, carer or friend acting as legal representative. Permission from legal representatives could also be given using the BIS. If no one was available then permission could be obtained if two clinicians (one unconnected with the trial) agreed to enrol the patient.
**Results:** Of 2325 enrolled participants, 1910 (82%) were recruited within the UK and 415 (18%) were recruited outside of the UK. Overall, full informed consent was obtained in 1552 (67%) participants, brief assent in 584 (25%) and independent physician assent 188 (8%). In a direct comparison, non-UK participants were more likely to have been recruited after full informed consent had been given than UK participants (83% versus 63%, $p<0.0001$). There was also a higher rate of non-UK participants who had the capacity to give full consent themselves (35% vs 21%, $p<0.0001$). Non-UK participants were also consented by an independent clinician slightly more often than UK participants (11% vs 8%, $p=0.038$).
**Discussion:** Non-UK participants were more likely to be enrolled after fully informed consent (given by the participant or a relative) than UK participants.

## P-254

### Sequence balance minimisation: new minimisation procedure for clinical trials using unequal treatment allocation ratios

Vichithranie Madurasinghe, Sandra Eldridge
Queen Mary University London, United Kingdom
*Trials* 2019, **20(Suppl 1):**P-254

**Introduction:** Minimisation is a widely used randomisation scheme that ensures excellent balance between treatment groups for several prognostic factors, even in small samples. However, extending it to trials using unequal allocation ratios is challenging. Sequence balance minimisation is a new procedure extending minimisation for trials using unequal allocation ratios. Here we show that sequence balance minimisation has good treatment and factor balancing properties, and is a valid alternative for stratified block randomisation especially in small trials using unequal allocation ratios.
**Methods:** Treatment and factor balancing properties of sequence balance minimisation were explored in a simulation study for a two arm trial with 1:2 allocation ratio. Number of prognostic factors on which to achieve balance ranged from 1 to 10 prognostic factors with 2 levels occurring in equal probabilities. Sample size was set to 30 and 120.
**Results :** Including additional prognostic factors (up to 10) in the sequence balance minimisation had little impact on overall treatment and factor balance; the mean and median number of allocations achieved remained as same as the expected number, that is 10 patients 5 of whom with each factor level in sample size 30, and 40 patients 20 of whom with each factor level in sample size 120 respectively.
Treatment and factor balance performance of stratified block randomisation deteriorated as the number of prognostic factors included in the scheme increased (up to 10) with variations in allocations achieved increasing and reaching similar levels to that of simple randomisation.
In all scenarios, sequence balance minimisation outperformed simple randomisation providing less variations in allocations achieved.
**Discussion:** Sequence balance minimisation has good treatment and factor balancing capabilities, and is a valid alternative to stratified block randomisation particularly in small trials, providing better treatment and factor balance across greater number of prognostic factors.

## P-255

### Bringing together the "pieces of the puzzle": a qualitative study of how trainee research networks and collaborators can work together to optimise trial conduct

Clare Clement[1], Karen Coulman[1], Thomas Pinkney[2], Jane Blazeby[1], Natalie Blencowe[1], Nick Heywood[3], Jonathan Cook[4], Richard Bulbulia[4], Tony Marson[5], Alejandro Arenas-Pinto[6], Athene Lane[1]
[1]University of Bristol, United Kingdom; [2]University of Birmingham, United Kingdom; [3]Manchester University National Health Service Foundation Trust, United Kingdom; [4]University of Oxford, United Kingdom; [5]Liverpool University, United Kingdom; [6]University College London, United Kingdom
*Trials* 2019, **20(Suppl 1):**P-255

**Introduction:** Trainee Research Collaboratives (TRCs) have been established across the UK which allow surgical trainees to join large collaborative research groups. These networks can generate new trials, improve recruitment to ongoing trials and develop a research-active future consultant workforce. How TRCs work with stakeholders and the roles they play have not been evaluated. Understanding them is key to maximising their potential to optimise TRCs and trial conduct.
**Methods:** Semi-structured interviews with 32 TRC members and linked personnel and observations of TRC meetings (n = 5) were undertaken. Interviewees were purposefully sampled to include a range of key stakeholders across different UK regions. Interviews and observations explored experiences of participating in TRC trials and understanding the roles of stakeholders. Interviews were audio-recorded, transcribed and analysed thematically, alongside observation field notes. A stakeholder meeting identified key strategies to enhance clinicians' engagement in trials.
**Results:** TRCs play an important role in conducting rigorous research, and the support of key players was needed to facilitate the success of TRC research. Consultant surgeons are needed to champion TRCs, provide mentorship to trainees and facilitate trainee involvement in trials. Research nurses are needed to help support trainees and coordinate trainee activity. Clinical Trials Unit (CTU) staff are needed for methodological input and training which compliments clinician expertise. However, there are challenges to collaborative working between the groups such as unclear contact points, lack of consensus on authorship policies and a lack of awareness of the benefits of collaborative working. To overcome these challenges, there needs to be an acknowledged mutually beneficial relationship between the groups, as well as improved communication and pathways for working.
**Discussion:** Multi-disciplinary teams are needed to ensure the success of TRC research. Enhancing relationships between key stakeholders and maximising the skills and knowledge within multi-disciplinary teams can optimise TRCs and thus trials.

## P-256

### Engaging surgical trainees to optimise clinical trials: a qualitative evaluation of how trainee research collaboratives achieve success

Clare Clement[1], Karen Coulman[1], Thomas Pinkney[2], Jane Blazeby[1], Natalie Blencowe[1], Nick Heywood[3], Jonathan Cook[4], Richard Bulbulia[4], Tony Marson[5], Alejandro Arenas-Pinto[6], Athene Lane[1]
[1]University of Brisol, United Kingdom; [2]University of Birmingham, United Kingdom; [3]Manchester University National Health Service Foundation Trust, United Kingdom; [4]University of Oxford, United Kingdom; [5]Liverpool University, United Kingdom; [6]University College London, United Kingdom
*Trials* 2019, **20(Suppl 1):**P-256

**Introduction:** Trials rarely recruit well or complete on time and one contributory factor is clinician engagement. This is often worse in surgical trials, where preferences for specific interventions are strong and research-active senior surgeons rare. Trainee Research Collaboratives (TRCs) have been established across the UK which allow surgical trainees to join large collaborative groups. TRCs have completed several surgical trials on time and target. We aimed to understand key elements of successful trainee engagement in trials and the role TRCs play in facilitating this engagement and conducting successful trials.
**Methods:** Semi-structured interviews (n=32) explored motivation for trainee engagement in trials, experiences of participating in TRCs trials, including barriers and facilitators. Observations of TRC meetings (n = 5), a survey (n=72) and a stakeholder meeting were also undertaken. TRCs and linked personnel were purposefully sampled to include a range of key stakeholders and specialties across different UK regions. Interviews were audio-recorded, transcribed and analysed thematically, alongside observation field notes.
**Results:** Trainees engage with trials to progress their careers, contribute to the surgical evidence base and improve patient care. Challenges to engagement included competing clinical priorities, "trainee fatigue", trainee confidence, integration into traditional hierarchies, recognition of trainee input and authorship issues. TRCs were perceived to play an important role in providing a supportive infrastructure to facilitate engagement with mentoring being a key feature. Challenges can be further overcome by engaging senior surgeon support, conducting achievable study designs, transparency of involvement and recognition, consideration of corporate authorship policies and providing pathways and activities for training including "on the job" training.
**Discussion:** Using multiple methods to understand trainee engagement in trials and the role of TRCs has helped elucidate how trial conduct and delivery can be improved. Findings could potentially translate to other specialties.

## P-257

### A systematic literature review of site staff training methods in clinical trials

Athanasia Gravani, Andrew Beswick, Chris Rogers, J Athene Lane
University Of Bristol, Bristol, United Kingdom
*Trials* 2019, **20(Suppl 1):**P-257

**Introduction:** Clinical trial staff must be able to demonstrate appropriate education and training relating to their roles to fulfil ICH Good Clinical Practice guidelines and facilitate trial conduct. We aimed to investigate training methods used in randomized controlled trials (RCT) of health-care interventions.
**Methods**: A systematic search was undertaken of multiple databases in November 2013 to identify articles reporting methods and practices of staff training within RCTs. Key trials journals and reference lists of included papers were hand searched for additional references. As data extracted was largely descriptive a meta-analysis was not possible, so thematic categories and sub-codes were developed and used to summarise and compare studies. An updated search revealed no new systematic reviews and identification of individual papers is ongoing (November 2018).
**Results:** 7,471 records were screened for eligibility (full texts of 177 articles were reviewed) and 89 studies identified (46 from database searches and 55 through manual searches). Host studies focused on a wide range of diseases: 23 (26%) circulatory system, 15 (17%) mental/behavioural disorders, 14 (16%) neoplasms and 13 (15%) endocrine diseases. Studies were mainly conducted in the USA (55%) or multi-nationally (27%). Multiple methods of training were used with different combinations of live (face-to-face and remote) and recorded (text-based and multimedia) methods. Training was often provided in groups (51%) by research team trainers (65%). Training costs were only provided in four (4.5%) studies.

**Conclusions:** The essential requirement for clinical trial staff training is discordant with the paucity of published details suggesting more standardised reporting is required. Wide variation existed in staff training process across RCTs of various intervention types and disease areas. Further research is needed to robustly evaluate staff training process within RCTs, including their impact on trial conduct and costs.
This was funded by the UK MRC ConDuCT-II Hub for Trial Methodology Research.

## P-258

### The challenges of conducting an online paediatric study (Teen Online Problem Solving for adolescents who have survived a brain injury in the UK: a feasibility study (TOPS-UK)

Alison Jeffery[1], Jonny Wilks[1], Siobhan Mitchell[2], Laura Cocking[1], Janet Smithson[2], Anna Adlam[2]
[1]Peninsula Clinical Trials Unit, Plymouth University, Plymouth, United Kingdom; [2]Department of Psychology, Exeter University, Exeter, United Kingdom
*Trials* 2019, **20(Suppl 1):**P-258

**Introduction:** Online studies have efficiency, convenience, geographical, time and cost saving advantages. In paediatric studies, participants may also feel more in control, with less social pressure to participate. However, risks include uncertainty around who has consented, limited opportunity for participants to ask questions, technology failure, requirement to be IT literate, and need for appropriate web access.
**Methods:** Our NIHR funded study tested an adolescent online psychological support programme intervention with Skype and telephone support but no face-to-face contact with any researcher. PenCTU delivered the online study management and data entry system. Participants were approached by post or email; informed consent, screening and study measures were managed online.
**Results:** Trial Manager perspective: Ethical approval was straightforward; consent forms were completed correctly and receipt at PenCTU was immediate. Participants dictated the rate of progress resulting in some long intervals between approach, consent, questionnaire and intervention completion. The lack of initial face-to-face contact may have contributed to poor recruitment to the study. However, once recruited, retention was good.
**Participant perspective:** Families accepted the approach and reported that the online format was convenient and intuitive to follow, but some technical difficulties increased participant burden. Some families required telephone support during questionnaire completion and would have welcomed initial face-to-face contact with the therapist to build rapport during the intervention.
**Discussion:** Online consent worked well in this feasibility study, however the risks and benefits would need to be carefully balanced for a higher risk or complicated study, and a Skype link would provide additional reassurance for both researchers and participants.
In future online paediatric studies, we would recommend an initial face-to-face meeting, offering contact by social media, improving compatibility with smartphones and incorporating interactive or video links into participant facing documents for further information.

## P-259

### Designing and implementing an individually-randomised trial in the presence of a substantial risk of contamination

Chris J Sutton[1], Denise Forshaw[2], Emma Neil[2], Lois H Thomas[2]
[1]The University of Manchester, Manchester, United Kingdom; [2]University of Central Lancashire, Preston, United Kingdom
*Trials* 2019, **20(Suppl 1):**P-259

**Introduction:** Cluster-randomised trials (CRTs) are often used to avoid the contamination risk inherent in individually-randomised trials (IRTs) of many complex interventions, although they can have other limitations including selection bias and large sample sizes. We aimed to design and implement an IRT of a systematic voiding programme (SVP) allowing for contamination whilst limiting its potential through careful design and close monitoring of usual care (UC) delivery.
**Methods:** Patients individually-randomised to receive either UC delivered by existing ward staff or SVP delivered by specifically-trained and allocated existing ward staff, supported by a study-specific champion.
Adjustment of effect size for contamination when calculating sample size; development of process measures to evaluate the extent of contamination; internal pilot to confirm an acceptable level of contamination. Data to evaluate contamination collected via case-note review of care received by UC participants.
**Results:** Even with a 25% reduction in effect size due to contamination, the sample size for the IRT was considerably less than that for a CRT without effect size contamination. Through discussions with the Trial Team and the Trial Steering Committee, we developed measures of contamination:
1.Percentage of UC participants with a:
a.strategy for minimising indwelling urinary catheterisation in the acute phase unless clinically justifiable; and
b.comprehensive continence assessment; and
c.tailored treatment plan (including behavioural approaches).
2.Percentage of UC participants receiving toileting assistance from staff trained to deliver the SVP intervention on 50% or more occasions.
These will be evaluated in an internal pilot: if each is <=25%, 'green light' to main trial.
**Discussion:** An IRT can be more efficient than a CRT, even in the presence of moderate contamination. However, it is important to limit contamination by careful design and implementation, to monitor and evaluate the extent of contamination, and to consider this when interpreting effectiveness and cost-effectiveness.

## P-260

### Conducting trials in public health settings: lessons from the Optimising Family Engagement in HENRY (OFTEN) cluster randomised controlled trial

Michelle Collinson[1], Wendy Burton[1], Julia Brown[1], Amanda Farrin[1], Robbie Foy[2], Suzanne Hartley[1], Jane Nixon[1], Kim Roberts[3], Harry Rutter[4], June Stevens[5], Sandy Tubeuf[6], Maria Bryant[1]
[1]Clinical Trials Research Unit, University of Leeds, Leeds, United Kingdom; [2]Leeds Institute of Health Sciences, University of Leeds, Leeds, United Kingdom; [3]HENRY Head Office, 8 Elm Place, Old Witney Road, Eynsham, United Kingdom; [4]Department of Social & Policy Sciences, University of Bath, Bath, United Kingdom; [5]Department of Nutrition, Gillings School of Public Health, University of North Carolina, USA; [6]Institute of Health and Society, Université Catholique de Louvain, Belgium
*Trials* 2019, **20(Suppl 1):**P-260

**Introduction:** Conducting trials within early years community settings, managed by public health teams is challenging; especially given the fluidity in structure and organisation, and the increasing political and economical demands.
Additionally, ensuring that routine data from research naïve environments is of high quality, sufficiently robust for research and is transferred in an appropriate manner can be time-consuming.
We will present the challenges encountered during the set-up, implementation and analysis of a public health trial, sharing the lessons learnt and detailing the strategies developed to overcome these issues.
**Methods:** OFTEN is a NIHR-funded, multi-centre, two-arm, cluster randomised controlled trial evaluating the effectiveness of an optimisation intervention aimed at promoting parent engagement to 'HENRY', a UK community-based obesity prevention intervention.
The trial was delivered in 126 children's centres across 20 local authorities; outcomes were evaluated using anonymised data, routinely collected from each centre.

**Results:** Set-up and implementation proved challenging as the setting underwent substantial restructuring due to austerity, resulting in centre closures or mergers, thereby reducing research capacity. Due to competing demands, the intervention was de-prioritised resulting in low intervention fidelity.
The trial faced many data issues: data required for randomisation was not consistently entered into databases; data specifications were not initially understood therefore some data were incorrectly entered; and differing methods were used to show missing data. Additionally, poor quality assurance processes led to transfer of incorrect data and the deletion of some data required for process evaluation.
**Discussion:** Researchers should ensure those involved in collecting and transferring data for research in public health settings receive training in data management and data protection. We also encourage the use of quality assurance checklists. Researchers can use our practical, easy to implement strategies to overcome some of the key challenges faced when conducting future research in public health settings.

## P-261

### The importance of continued follow-up in cancer trials: results from the TEAMM myeloma trial assessing the benefit of 12 weeks levofloxacin prophylaxis on febrile episodes or deaths

Gulnaz Iqbal[1], Mark Drayson[2], Stella Bowcock[3], Tim Planche[4], Janet Dunn[1]
[1]University Of Warwick, Coventry, United Kingdom; [2]University of Birmingham, Birmingham, United Kingdom; [3]Kings College Hospital NHS Trust, United Kingdom; [4]University of London, United Kingdom
*Trials* 2019, **20(Suppl 1):**P-261

**Introduction:** Myeloma causes profound immunodeficiency and recurrent, serious infections. There are approximately 5,500 new UK cases of myeloma per annum; a quarter will have a serious infection within 3 months of diagnosis. Newly diagnosed patients may benefit from antibiotic prophylaxis to prevent infection and early death but this may be associated with healthcare-associated infections
**Methods:** The Tackling Early Morbidity and Mortality in Myeloma trial (TEAMM) trial was a multicentre randomised, double-blind, placebo-controlled trial in newly-diagnosed myeloma patients randomised to receive Levofloxacin or placebo for 12 weeks at the start of anti-myeloma treatment. Follow-up was 4-weekly to 16 weeks and again at 1 year. The composite primary outcome was defined as time to first febrile episode or death in the first 12 weeks from start of trial treatment. Secondary outcomes included overall survival at 16 weeks up to 12 months.
**Results:** 977 patients were randomised (489 levofloxacin, 488 placebo). Primary events (febrile episodes, deaths, febrile episodes with death) in levofloxacin versus placebo arms were 19% vs 27% (87, 4, 4 vs 112, 15, 7), respectively; HR=0.66 (95% CI 0.51-0.86) p=0.002. There was a benefit in overall survival at 12 weeks (p=0.008) but not at 12 months (p=0.94).
**Conclusions:** The use of 12 weeks prophylactic antibiotics significantly reduced the number of febrile episodes or deaths within the first 16 weeks. However, this did not predict longer-term survival. Models to adjust for additional treatments indicated that the use of co-trimoxazole was independently associated with improved survival. TEAMM demonstrates the need for longer-term follow-up in cancer trials. This has influenced the design of TEAMM2 trial.

## P-262

### Challenges of running a cancer trial with multiple primary tumour sites – ICR-CTSU experience based on the CORE trial

Natasha Iles[1], Lucy Kilburn[1], Zaynah Gurreebun[1], Christy Toms[1], Anna Kirby[2], Merina Ahmed[2], Nicholas Van As[2], Vincent Khoo[2], Emma Hall[1]
[1]The Institute of Cancer Research Clinical Trials and Statistics Unit, Sutton, United Kingdom; [2]The Royal Marsden NHS Foundation Trust, London, United Kingdom
*Trials* 2019, **20(Suppl 1):**P-262

**Introduction:** CORE (ClinicalTrials.gov NCT02759783) is a multicentre phase II randomised controlled trial investigating the addition of stereotactic body radiotherapy (SBRT) to conventional care for extra-cranial oligometastases in patients with breast, prostate and non-small cell lung cancer. Including multiple cohorts each with a different primary tumour site, allows efficient evaluation of safety of SBRT and feasibility of conducting subsequent cohort specific phase III trials; however this also presents several challenges for both trial design and management.
**Methods and Results:** Trial eligibility criteria and patient pathways needed to be carefully considered to fit with different standard of care pathways for each cohort, whilst allowing for a combined analysis. Expertise for each cohort during protocol development and on oversight committees was essential. Similarly, at sites, liaising with three different clinical teams was common, necessitating a coordinated approach and more resources.
The trial design originally assumed even recruitment across cohorts, but practically, while recruitment to breast and lung cohorts was as predicted, recruitment to prostate exceeded expectations. With a single recruitment target over all cohorts, the larger number of prostate cancer patients recruited impacted on the statistical assumptions. Recruitment to the prostate cohort was temporarily suspended, while engagement with breast and lung communities was increased, amendments to the protocol were made to improve recruitment to these cohorts and discussions around the implications to the trial design were had with the Trial Steering Committee. The control group progression-free survival estimate was revised based on the recruitment ratio seen between the cohorts and therefore the sample size was increased.
**Discussion:** Recruitment was completed in February 2019. Frequent collaboration with the trial oversight committees and sites, and close monitoring of recruitment, enabled effective trial design and management to maximise the information gained from the trial, including the feasibility of subsequent phase III trials.

## P-263

### Challenges of using electronic real time measuring devices in randomised controlled trials

Seonaidh Cotton, Victoria Bell, Shaun Treweek, Steve Turner
University Of Aberdeen, Aberdeen, United Kingdom
*Trials* 2019, **20(Suppl 1):**P-263

**Introduction:** Electronic real time measuring devices are increasingly used in RCTs, eg to capture outcome data or as part of the intervention. They are not without challenges, as we discuss for two distinct devices in different trials.
**Challenges:** The first device is an adherence monitor used in RAACENO (ISRCTN67875351) to measure adherence to asthma medication as part of the intervention. We anticipated some challenges to using these monitors (eg children losing the monitor, uncharged batteries). There were also unanticipated challenges (eg NHS firewalls preventing download of adherence data; or some monitors not accurately capturing medication use). The study was designed so that monitor-reported or family-reported adherence could be used in treatment decision-making. Adherence monitors were returned at 430 follow-up appointments; in 357, adherence data could be downloaded from the monitor. Of these, 264 suggested adherence of <70% (ie non-adherent), but based on family-reported adherence, the research team classified 141/264 as being adherent to their asthma medication.
The second example is an activity monitor used in the EuroflT trial (ISRCTN81935608) to objectively measure primary outcome (total physical activity, total sedentary time). All participants also self-reported activity and sitting time. The activity monitors had challenges: skin reactions to the adherence tape; people not wearing them all day; and software problems. At follow-up, primary outcome data from the activity monitor was available from approximately 83% of participants. Some of those who did not have outcome data available from the activity monitor provided self-reported secondary outcome data on activity levels – however in this study there were substantial differences between objective measurement and self-report, casting substantial doubt on the value of self-report
**Discussion:** Trials using devices for trial outcome data need to understand both the technical challenges of implementation but also the differences between device data and other sources of notionally equivalent data.

## P-264
### What impact does form re-design and user testing have on consent form completion errors?

Peter Knapp[1], Peter Bower[2], Caroline Fairhurst[3], Jenny Roche[3], ISDR Study Team[4]
[1]University Of York & The Hull York Medical School, United Kingdom; [2]University of Manchester, UK; [3]University Of York, UK; [4]University of Liverpool, United Kingdom
*Trials* 2019, **20(Suppl 1):**P-264

**Introduction:** Consent to research requires participants to endorse and sign a consent form, usually a printed sheet. Completion errors may invalidate consent, requiring participants to be re-consented, incurring recruitment delays and researcher opportunity costs, particularly in postal recruitment trials. The aim was to compare a trial consent form with a revised version, developed through information design (making changes to layout, appearance and navigation) and user testing, for rates of completion errors and corrections.
**Method:** The two consent forms were compared in a SWAT with sequential groups design. The revised form was used for participants to the ISDR trial over the last 6 weeks of recruitment. Error and completion rates were compared with rates in a sample of original version forms, used in the first 18 months of ISDR trial recruitment.
**Results**: The revised form was completed by 307 participants at five eye clinics in one city in Northern England during April-May 2016. For the original version of the form we sampled the forms for 720 participants recruited at the same five centres over November 2014-March 2016. Rates of completion errors were low: 7/720 (0.97%) in original forms; 2/307 (0.65%) in revised forms (including 2/720 and 0/307 important errors that would invalidate consent). Form correction rates were: 82/720 (11.4%) and 18/307 (5.9%), with lower correction rates in revised forms (Chi-square=7.48; df=1; p=.006). Most corrections were minor, non-GCP corrections (61/720; 14/307) rather than more significant, GCP corrections (21/720; 4/307).
**Discussion:** The revised consent form did not reduce completion errors in the ISDR trial, although overall rates were low. The revised form halved the rate of form corrections, indicating that information design changes can impact on participants' ease of completion. The revised form needs evaluation in a SWAT using random allocation, ideally in a trial using postal or other remote recruitment method.

## P-265
### An assessment of feasibility and cost-effectiveness of remote monitoring on a multicentre observational study

Jennifer Murphy, Margarita Durkina, Puja Jadav, Gaia Kiru
Imperial Clinical Trials Unit, Imperial College London, United Kingdom
*Trials* 2019, **20(Suppl 1):**P-265

**Introduction:** ICH GCP E6(R2) encourages risk-adapted approaches to monitoring by streamlining operations in line with the risks inherent in the protocol. The aim of this analysis is to determine the feasibility and cost-effectiveness of a remote monitoring model for a European, multi-centre study.
**Methods:** 6,000 subjects in 130+ centres across 18 countries were recruited to a low-risk observational study, in which a combination of remote and on-site monitoring was performed.
The monitoring plan required verification of 100% informed consent forms (ICFs), source data verification (SDV) of data for 20% of subjects and an investigator site file (ISF) check.
1-2 on-site visits were performed in each country and monthly monitoring calls were conducted for all centres. Self-compliance checklists were employed to verify the ICFs, SDV and ISF.
Data collected as part of monitoring compliance to the monitoring plan in an 18 month period will be analysed to determine the rates of errors identified at on-site visits compared to self-compliance checklists. A cost-benefit analysis will also be undertaken.
**Timing of potential results:** The results will be available by October 2019.
**Potential relevance:** This analysis will determine if self-compliance checklists are a reliable and cost-effective tool for remotely monitoring compliance to the protocol, GCP and study procedures at investigator sites.

## P-266
### 50% of the participants from 20% of the recruitment sites?

Seonaidh Cotton, Anne Duncan, Karen Innes, Gordon Fernie, Tracey Davidson, Susanne Breeman
University Of Aberdeen, United Kingdom
*Trials* 2019, **20(Suppl 1):**P-266

**Background:** The phrase "50% of participants from 20% of recruitment sites; 80% of participants from 50% of recruitment sites" is often quoted as a metric about recruitment of participants into randomised controlled trials, but to our knowledge there is no empirical evidence to confirm this.
**Methods:** Using a sample of 10 trials from our CTU, we investigated the proportion of participants recruited from top recruiting sites. Our sample included completed and ongoing trials.
**Results:** The number of recruitment sites in the 10 trials ranged from 20 to 121. The proportion of sites that recruited 50% of participants ranged from 8% to 42% (median 22%). The proportion of sites that recruited 80% of participants ranged from 33% to 71% (median 48%).
**Discussion:** For seven of the studies, the proportions of sites recruiting 50% of participants ranged between 15% and 26%. There were three studies where the proportions were outwith this range, all of which included primary care sites. For eight of the studies, the proportion of sites recruiting 80% of participants ranged between 40% and 60%. The two studies where the proportions were outwith this range were studies including primary care sites.
Our initial results suggests that the metric "50% of participants from 20% of recruitment sites and 80% of participants from 50% of recruitment sites" may hold true for studies in secondary care, and we aim to confirm this in a larger sample. Identifying the sites likely to be the best recruiters remains a challenge; using our data we will investigate whether there are any common features of these sites, for example whether they are sites where the Chief Investigator or other grantholders are based.

## P-267

### Collaborative CTU Monitoring Survey

Karen Martin, Sara Yeats, Andrea Corkhill
Southampton Clinical Trials Unit, Southampton, United Kingdom
*Trials* 2019, **20(Suppl 1):**P-267

**Introduction:** This poster reports on a selection of results from a survey of UKCRC Registered Clinical Trials Units (CTUs) regarding their current monitoring structure and practices.
Academic CTUs undertake a range of trial types, often specialising in therapeutic areas. Trial monitoring is a key activity undertaken as part of trial management to ensure data integrity and subject safety.
It was evident from discussions between CTUs that there was a requirement to set up a collaborative forum to include all aspects of monitoring, lessons learned and to share experiences.
**Methods:** As a starting point, an online survey was created using Survey Monkey to collect details on the current monitoring set-up for each CTU, including novel approaches and areas of interest for future collaboration.
The survey comprises sixteen questions and five main categories. The categories include; Trial Portfolio and Trial Types; Sponsor; Monitoring Structure; Training and Monitoring Processes. Initially, the survey was distributed to UKCRC Registered Oncology CTUs that were known to have a specific interest in monitoring. To date, survey responses have been received from twenty-nine CTUs. We now plan to approach all the remaining UKCRC Registered CTUs to provide a much broader indication of monitoring set-up and practices across academic units.
**Timing of Potential Results:** The next wave of surveys will be distributed in May with results expected by the end of June. All results will then be analysed and presented, a selection of which will be included in the poster.
**Potential Relevance & Impact:** To our knowledge, there has not been a survey conducted previously to look specifically at monitoring and monitoring practices in academic CTUs and this will therefore be the first time this information has been collected and reported.

## P-268

### Challenges of conducting trials across multiple clinical specialities

Chris A Rogers, Sarah Baos, Lucy Culliford, Maria Pufulete, Ben Gibbison
University of Bristol, Bristol, United Kingdom
*Trials* 2019, **20(Suppl 1):**P-268

**Introduction:** Clinical trials are expensive and take several years to complete. Initiatives to improve efficiency and maximise generalisability of findings are actively encouraged. We describe the challenges of conducting an RCT across multiple specialties.
**Methods:** The NIHR-funded GAP study (ISRCTN63614165), to evaluate the effectiveness of gabapentin vs. placebo for pain management after surgery is being conducted in three surgical specialties (cardiac, thoracic and abdominal) in two hospitals.
**Results:** Surgical specialties are led by different clinical and research nurse teams based in different locations/departments within the hospitals. Despite this, regulations permit only one local PI for each hospital. This has presented logistical challenges (e.g. number of site files to have?) and has disincentivised clinicians in some specialties. PI delegation of responsibilities (e.g. assessing eligibility/safety) to colleagues in other specialties has had to be much higher than usual. Training of non-GCP-trained clinical staff has been a major undertaking, facilitated with targeted training materials covering key aspects of GCP.
Designing study materials (e.g. information leaflets, data collection forms) that include all necessary and appropriate information for different patient populations and recruitment pathways (e.g. cardiac surgery vs. cancer surgery) required careful consideration.
Adaptable study coordination and monitoring procedures are necessary; allowing for different ways of working across specialties (e.g. greenlighting the site or specialty) and enabling identification of trends at site and specialty level. The sponsor, whilst viewing each hospital as a single site, has monitored speciality separately. The system for capturing local research activity does not allow recruitment to be easily attributed to different specialties, resulting in discrepancies and frustration for local teams.
**Discussion:** The study design, involving three surgical specialties was chosen to maximise the value of the research for the NHS. This study highlights that while the design is methodologically attractive, the current regulatory structures and NHS systems make implementation sub-optimal.

## P-269

### Risk of bias and implication for sample size of an urgent safety measure requiring trial recruitment suspension

Lee Middleton[1], Hilary Critchley[2], Lee Priest[1], Peter Brocklehurst[1], Jane Daniels[3]
[1]University of Birmingham Clinical Trials Unit, Birmingham, United Kingdom; [2]University of Edinburgh, MRC Centre for Reproductive Health, Edinburgh, United Kingdom; [3]University of Nottingham Clinical Trials Unit, Nottingham, United Kingdom
*Trials* 2019, **20(Suppl 1):**P-269

**Introduction:** UCON is an open-label randomised trial comparing ulipristal acetate (UPA) with the levonorgestrel-releasing intra-uterine system (LNG-IUS) in women with heavy menstrual bleeding. In February 2018, the European Medicines Agency issued a drug alert for UPA. An urgent safety measure (USM) was implemented, prompting suspension of recruitment and UPA use.
We discuss the implications of the USM on the risk of bias and sample size.
**Methods:** Women take UPA for 12 weeks followed by 4 weeks off-treatment, for 3 courses. The participant-reported primary outcome is collected after the final off-treatment week. The USM allowed women to complete a course, if they wanted, but not start a subsequent course. The original sample size was 172 with primary outcome, 220 assuming ≈20% drop-out: 198 had been randomised when recruitment was suspended. On resumption, the UPA group could complete 3 courses, subject to monthly blood tests.
**Results:** Four populations are apparent, according to status at suspension.
A:Completed trial prior to USM, no risk of bias (44 UPA; 45 LNG-IUS).
B:Complete after USM, possible risk of bias, due to knowledge of USM (could impact either group) or decision to stop UPA (50 UPA, completed or discontinued 3rd course; 48 LNG-IUS)
C:In course 1 or 2 when USM implemented, high risk of bias, as UPA group could not complete 3 courses (6 UPA, 7 LNG-IUS).
D:Future participants. Theoretically no risk of bias, but UPA group will have monthly blood tests.
We will exclude populations B and C, both UPA and LNG-IUS, from the primary analysis, but include in a secondary analysis. 104 more randomisations are needed (population D).
**Discussion:** Following a USM, risk of bias should be considered and assumed to apply to both groups. Recruitment may need to be increased to replace impacted participants, enabling a clean primary analysis.

## P-270

### Recruiting to time and target: experiences from the ERIC-PPCI trial

Alexander Perkins[1], Richard Evans[1], Derek Yellon[2], Rajesh Kharbanda[3], Matt Dodd[1], Tim Clayton[1], Derek Hausenloy[4]
[1]London School of Hygiene and Tropical Medicine, London, United Kingdom; [2]The Hatter Institute, University College of London, London, United Kingdom; [3]John Radcliffe Hospital, University of Oxford, Oxford, United Kingdom; [4]Duke-NUS Medical School, Singapore, Singapore
*Trials* 2019, **20(Suppl 1):**P-270

**Introduction:** Recruiting sufficient patients to randomised trials is a familiar challenge. In ERIC-PPCI the ability of remote ischaemic conditioning to improve outcomes in heart attack patients was examined. The intervention and urgent clinical care had to be given simultaneously. Patients were conscious but had received morphine or were in pain, which reduced their capacity to consent. This presentation will discuss the measures taken to ensure the challenging research context did not affect the delivery of patient care or ability of the trial to recruit.

**Methods:** Trial treatment was automated to reduce the burden for the research staff and patients. A delayed consent with verbal assent model was used to expedite patient randomisation and treatment. Full consent was taken after the patient had received clinical care and the allocated trial treatment. Successful recruitment and consenting strategies were shared among participating sites throughout the trial.

**Results:** ERIC-PPCI completed recruitment of the initial target of 2000 patients 12 months ahead of schedule. This enabled the sample size to be increased to account for a slightly lower than expected event rate. The final recruitment total of 2800 patients was reached before the original recruitment end date.

**Discussion:** Integrating research pathways with care pathways makes recruitment easier for research staff and less disruptive for patients. Flexibility in consent models allows trials to meet the needs of the patients, clinical setting and research question. The ease with which patients can be randomised and the intervention can be given are key priorities in trial design.

Investment in well-designed trials could return savings as trials would be more able to respond to challenges and deliver definitive answers to the key research question without costly extensions or unnecessary repeated research.

## P-271

### Engaging surgical trainees in Orthopaedic, NIHR Portfolio, multicentre randomised controlled trials

Gemma Greenall[1], Cushla Cooper[1], Christopher, P. Bretherton[2], Alexander Martin[2], Jonathan Gower[3], Siôn Glyn-Jones[1,2]

[1]Nuffield Department of Orthopaedics, Rheumatology and Musculoskeletal Sciences (NDORMS), University Of Oxford, Headington, United Kingdom; [2]Oxford University Hospitals NHS Foundation Trust, Headington, United Kingdom; [3]NIHR Comprehensive Clinical Research Network Coordinating Centre, Leeds, United Kingdom

*Trials* 2019, **20(Suppl 1):**P-271

**Introduction:** Recruitment to surgical trials is challenging and clinician engagement plays a significant role in the success of a trial. Complexities such as rare patient population, multiple speciality involvement and the use of an investigational medicinal product in an environment where surgery is the area of interest, all raise challenges to engagement and recruitment.

The MANTIS trial (NIHR HTA 15/39/06), has adopted alternative methods to improve recruitment, due to these complexities. In recent years, regional 'Research Collaboratives' have formed to improve trainee engagement in surgical research, a resource utilised in MANTIS.

The aim of this project is to review the engagement/involvement of surgical trainees in NIHR Portfolio, multicentre, randomised controlled trials and how this can benefit both the research community and the professional development of surgical trainees.

**Methods:** A questionnaire will be circulated to trainee 'Research Collaboratives' across the UK and identified trainees already engaged at recruiting sites participating in the MANTIS trial. Domains will cover:

•Routes of engagement
•Level of involvement in ongoing/past trials
•Accreditation and incentives for participation
•Multi-disciplinary engagement
•Recruitment strategies

**Timing of potential results:** The deadline for responses to the questionnaire will be the end of August 2019. The results will be collated and analysed in September 2019.

Potential Relevance and Impact

Involving surgical trainees in clinical research has the potential to benefit the trial management team for multicentre randomised controlled trials. It could also aid the professional development of the trainees themselves and encourage the incorporation of research into routine clinical care and training.

If surgical trainees are engaged sufficiently early in trial development, their involvement could improve study set-up, recruitment and retention in trials. Understanding current trainee involvement (when and how they are involved) and how to improve engagement, has great potential to positively impact on trials going forward.

## P-272

### On-site monitoring of primary outcomes is important in primary care clinical trials: Benefits of Aldosterone Receptor Antagonism in Chronic Kidney Disease (BARACK-D) Trial a case study

Louise Jones, Emma Ogburn, Ly-Mee Yu, Nargis Begum, Aaron Long, F D Richard Hobbs

University Of Oxford, United Kingdom

*Trials* 2019, **20(Suppl 1):**P-272

**Background:** To assess whether remote or on-site monitoring is a more efficient way to verify outcomes in primary care clinical trials, we conducted a case study in the BARACK-D trial. BARACK-D is a PROBE (Prospective Randomised Open Blinded Endpoint) trial of the licensed drug, spironolactone in patients with moderately severe chronic kidney disease (CKD). Patients were randomised between spironolactone 25mg daily plus routine care, versus routine care. The primary outcome is change in rate of combined cardiovascular events. As with many trials, the observed primary event rate in the early phase of the trial was lower than expected. A risk-based approach to monitoring meant that all monitoring would be done remotely.

**Method:** Trial recruitment commenced in November 2013, from January – March 2018 on site monitoring visits were conducted to verify primary endpoints reported by the general practices, which generally have very busy competing clinical demands. To maximise data verification, practices were monitored where >5 participants were recruited, and participants had >1 years trial exposure from randomisation. 50 practices were visited and data relating to 598 participants verified.

**Results:** Prior to the source data verification 42 primary endpoints were reported from all 250 practices (3.8% of 1112 randomised), an incidence rate of 3.2 per 100 person-years (95% CI, 2.4 to 4.4) against the expected rate of 12. On-site monitoring identified an extra 73 primary endpoints, the primary outcome event rate for monitored practices rose to 10.01 per 100 person-years (95% CI, 8.10 to 12.38) which is in the region of expected rate.

**Conclusions:** Our data suggest that extensive on-site monitoring of primary outcomes is important to ascertain accurate reporting, for clinical trials in a primary care setting. This is in contrast to recent publications stating that triggered or risk-adaptive monitoring is an efficient way to prioritise and reduce on-site monitoring.

## P-273

### Developing and testing high-efficacy patient subgroups within a clinical trial using polygenic risk scores

Svetlana Cherlin[1,2], James M S Wason[2,3]

[1]Newcastle Clinical Trials Unit, Newcastle University, Newcastle Upon Tyne, United Kingdom; [2]Institute of Health and Society, Newcastle University, Newcastle Upon Tyne, United Kingdom; [3]MRC Biostatistics Unit, University of Cambridge, Cambridge, United Kingdom

*Trials* 2019, **20(Suppl 1):**P-273

**Introduction:** It is increasingly common in clinical trials to collect a lot of data about patients such as genomic, imaging, data from wearable technologies. There is the potential for this high-dimensional information to be informative for the efficacy of a new treatment in the situations where only a subset of patients benefits from the treatment. The adaptive signature design method allows a trial to develop and test efficacy of treatment in a high-efficacy patient group (the sensitive group) using genetic data. Patients are classified to be sensitive or not based on their genetic information. The method requires specification of a set of tuning parameters for identifying the sensitive group. Selection of the tuning parameters is implemented by a time-consuming nested cross-validation procedure.
**Methods:** We propose a variation to the adaptive signature design method that does not require selection of the tuning parameters. The method is based on polygenic risk scores that utilise weighed contribution of the gene expression levels. The sensitive group is found by applying a nonparametric clustering procedure to the polygenic risk scores. We have implemented the new method in an R package.
**Results:** The performance of the new method is assessed for various sample sizes and response rates. The new method has substantial reduction in computational time required. In many scenarios there is a substantial improvement in the ability to correctly identify the sensitive group and the overall power of the design.
**Discussion:** The new method for selecting a sensitive group of patients based on the polygenic risk scores shows a superior performance and drastically improves the computational time, in comparison to the existing one. Further research will focus on extending the method to incorporate different types of outcomes and a variety of types of biomarkers.

## P-274

**Sample size estimation for Randomised Controlled Trials with repeated assessment of patient reported outcomes: what correlation between baseline and follow up outcomes should we assume?**

Stephen Walters, Richard Jacques, Inês Henriques-Cadby, Jane Candlish, Nikki Totton
University Of Sheffield, Sheffield, United Kingdom
*Trials* 2019, **20(Suppl 1):**P-274

**Introduction:** Patient Reported Outcome Measures (PROMs) are now frequently used in randomised controlled trials (RCTs) as primary endpoints. RCTs are longitudinal and many have a baseline (PRE) assessment of the outcome and one or more post-randomisation assessments of outcome (POST). With such designs there are several ways of estimating the sample size and analysing the outcome data:
1)Analysis of post-randomisation treatment means (POST)
2)Analysis of mean changes from pre to post-randomisation (CHAN GE)
3)Analysis of covariance (ANCOVA)
Sample size estimation using the CHANGE and ANCOVA methods requires specification of the correlation between the baseline and follow-up measurements. Assuming a correlation of 0.70 between baseline and follow-up outcomes in the ANCOVA method would halve the sample size compared with using the POST method. Therefore, if the correlation is overestimated the study results may be under powered. So what correlation (between baseline and follow-up outcomes) should be assumed and used in the sample size calculation?
**Aims:** To estimate the correlations between baseline and follow-up PROMs in RCTs.
**Methods:** The Pearson correlation coefficients between the baseline and repeated PROMs (Barthel, EQ-5D, EORTC-QLC-C30, SPADI, SF-36, WOMAC etc) assessments from 20 RCTs (with 7,173 participants at baseline) were calculated and summarised.
**Results:** The 20 reviewed RCTs had baseline sample sizes ranging from 49 to 2,659. The time-points for the post-randomisation follow-up assessments ranged from 0.25 to 24 months. 464 correlations, between baseline and follow-up, were estimated; the mean correlation was 0.50 (SD 0.15; median 0.51, range -0.13 to 0.91).
**Conclusions:** There is a general consistency in the correlations between the repeated PROMs, the majority being in the range 0.41 to 0.60. A correlation of 0.5 implies that we can reduce the sample size in a RCT by 25% if we use an ANCOVA model for the design and analysis.

## P-275

**When is a Type B CTIMP not a Type B CTIMP?**

Fay Chinnery[1], Nick Francis[2], Gareth Griffiths[1], Paul Little[3], Ingrid Muller[3], Jacqui Nuttall[1], Matt Ridd[4], Beth Stuart[3], Tracey Sach[5], Irene Soulsby[6], Karen Thomas[6], Kim Thomas[7], Alison Layton[8], Miriam Santer[3], Louise Stanton[1]
[1]Southampton Clinical Trials Unit, University of Southampton, UK; [2]Division of Population Medicine, Cardiff University, UK; [3]Primary Care and Population Sciences, University of Southampton, UK; [4]Population Health Sciences, University of Bristol, UK; [5]Health Economics Group, Norwich Medical School, University of East Anglia, UK; [6]PPI contributor, UK; [7]Centre of Evidence Based Dermatology, University of Nottingham, UK; [8]Hull York Medical School, University of York, UK
*Trials* 2019, **20(Suppl 1):**P-275

**Introduction:** The SAFA (Spironolactone for Adult Female Acne) RCT compares spironolactone with placebo (in addition to standard topical care) for moderate-severe persistent facial acne in adult women.
Spironolactone is licensed in the UK for indications including hypertension and heart failure, but not acne. Consequently, the SAFA trial could be classed as a Type B CTIMP (testing authorised medicinal products according to treatment regimens outside the marketing authorisation; somewhat higher than the risk of standard medical care).
However, spironolactone has been used off-license for treating acne in women for over 30 years and the safety profile is well known. The MHRA actively promote a risk proportionate approach to CTIMPs, allowing methods to be adapted while still maintaining applicable standards.
**Methods:** Trial risks were assessed along with their mitigating strategies. Main considerations were:
-Trial dose (50-100mg spironolactone/day) is considered a low dose.
-Trial population is a much younger population than would be taking spironolactone within its licensed indication. Consequently, they are likely to have healthier cardiovascular and renal systems and fewer comorbidities. Blood serum potassium (to rule out hyperkalemia) and kidney function (eGFR) are tested at baseline. Evidence shows later testing is not needed.
-Concerns have been raised about teratogenicity of spironolactone, but risk of harm to the foetus is not thought to be high and is likely to be lower than for other oral treatments for acne. Women of child-bearing potential at risk of pregnancy must have a negative urine or serum pregnancy test at baseline.
**Results:** The MHRA accepted the risk assessment; the SAFA trial is classed as a Type A CTIMP (comparable to the risk of standard medical care).
**Discussion:** Assessing the IMP risk category and adapting the trial design accordingly maintains safety standards and reduces participants' burden of taking part in a clinical trial.

## P-276

**Continuity of researchers collecting outcome data within randomised controlled trials - any evidence of an impact on QoL measures?**

Rachel Evans, Zoe Hoare, Paul Brocklehurst
North Wales Organisation for Randomised Trials In Health (nworth), Bangor, United Kingdom
*Trials* 2019, **20(Suppl 1):**P-276

**Introduction:** Capturing changes in health and wellbeing within randomised controlled trials (RCT) can be complex. The precision and accuracy of outcome tools to measure change is crucial and consideration needs to be given to potential errors when collecting these outcomes.
Many RCTs use multiple researchers to collect data, accepting that this can introduce variation in measurement. This study aims to identify if there is a measurable effect of using different researchers to collect repeated assessments of Quality of Life (QoL) at different time-points.

**Methods:** A previously conducted study assessing the impact of reminiscence therapy on participants with dementia and carer (PwD-carer) dyads, 'REMCARE', was used. Data were categorised into those where the same researcher attended all assessments and those where different researchers undertook assessments. ANCOVA models used in the original REMCARE analysis with the addition of the 'researcher-consistency' variable were run on two QoL outcomes to assess the importance of this distinction.
**Results:** 330 PwD-carer dyads were included in the analysis. Statistically significant differences were found on researcher consistency at initial follow-up for the PwD QoL outcomes and at second follow-up for the proxy QoL outcome and the interaction between centre and researcher consistency for the PwD.
**Discussion:** These exploratory results indicate the possibility of an impact of researcher continuity on QoL outcomes within this dataset. Further research is required to establish causality definitively. If demonstrated this would have implications for planning future research studies and consideration of how outcome measures should be collected.

## P-277

### New methods for an update to a systematic review of core outcome sets for research

Elizabeth Gargon[1], Sarah Gorst[1], Christopher Norman[2,3], Mariska Leeflang[3], Aurelie Neveol[2], Prof Paula Williamson[1]
[1]University Of Liverpool, Liverpool, United Kingdom; [2]Limsi, Cnrs, Université Paris-Saclay , Paris, France; [3]Amsterdam Public Health, Amsterdam Umc, University of Amsterdam, Amsterdam, The Netherlands
*Trials* 2019, **20(Suppl 1):**P-277

**Introduction:** The Core Outcome Measures in Effectiveness Trials (COMET) Initiative maintains a public repository of core outcome sets (COS). Originally populated through a systematic review, annual updates keep it current. It is labour intensive and costly to keep this up-to-date. A balance is needed between managing this workload and identifying all eligible studies.
COS are now easily accessible and the Core Outcome Set-STAndards for Development (COS-STAD) recommendations were established to improve the methodological approach for developing COS, and help users assess whether a COS has been developed using a reasonable approach.
The fifth update of this systematic review aims to:
1.utilise automatic article ranking to assess suitability for assisting future updates to the annual systematic review of COS,
2.to apply COS-STAD to included COS and describe current COS standards of development.
**Methods:** Searches were carried out to identify COS (January-December 2018). A machine learning model, using logistic regression, was trained and evaluated, and subsequently used to rank records. Cut-off for screening was determined by the results of the model evaluation. Full texts of potentially relevant articles were assessed for inclusion. COS-STAD was applied to each included COS.
**Results timing:** We estimate that this model of automatic ranking can decrease the number of references that needs to be screened by 75% while identifying approximately 98% of all relevant references.
Results expected July.
**Potential Relevance & Impact:** We anticipate these results will demonstrate that automation can successfully assist the screening process in the annual update of the SR of COS, making it a more efficient process to keep the database current.
Research in the area of COS development is becoming more prevalent but is still quite new; we therefore expect few COS to meet all minimum-standards. This assessment is intended as a baseline against which future comparisons can be made.

## P-278

### Reducing bias due to measurement reactivity in studies of interventions to improve health

Diana Elbourne[1], Lisa Miles[2], David French[2], Andrew Farmer[3], Martin Gulliford[4], Louise Locock,[5], Jim McCambridge[6], Stephen Sutton[7]
[1]LSHTM, London, United Kingdom; [2]University of Manchester, Manchester, United Kingdom; [3]Nuffield Department of Primary Care Health Sciences, Oxford, United Kingdom; [4]School of Population Sciences and Health Services Research, Londin, United Kingdom; [5]Health Services Research Unit, Aberdeen, United Kingdom; [6]Department of Health Sciences, York, United Kingdom; [7]Department of Public Health and Primary Care, Cambridge, united kingdom
*Trials* 2019, **20(Suppl 1):**P-278

**Introduction:** There is clear systematic review evidence that measurement can affect people being measured. Changes in measured behaviour and other outcomes due to this reactivity may introduce bias in otherwise well-conducted randomised controlled trials. Researchers from Manchester, Aberdeen, Cambridge, Oxford, London and York developed the MEasurement Reactions In Trials (MERIT) study which was commissioned by MRC/NIHR to produce guidance on how to minimise bias due to measurement reactivity in studies of interventions to improve health.
**Methods:** Rapid systematic reviews have been conducted to establish the evidence-base for the guidance; an international Delphi procedure has been conducted to combine the views of experts on the required scope of the guidance, and a two-day expert workshop was then held to develop the guidance content.
**Results:** Systematic reviews showed that asking questions has a small but potentially important effect on measures of health-related behaviour. A rapid review of trials that investigated the effects of objective measurement on behaviour reported limited evidence, which focused mostly on physical activity.
40 experts participated in the Delphi procedure, and 23 in the expert workshop. These suggested that aspects of study design and appraisal, and selection of measurement tools and procedures, were important components to include in the guidance.
**Discussion:** The final MRC-NIHR guidance stresses the importance of considering risk of bias from measurement reactivity and theorising around potential measurement reactions during the early stages of trial design. It provides tools for appraising risk of bias from measurement reactivity, specific guidance on aspects of trial design that can be altered to minimise such biases, as well as guidance on trial conduct and analysis.

## P-279

### Statistical challenges in designing a randomised screening trial to identify efficacious components of a complex intervention: An example of a Multiphase Optimisation Strategy (MOST) design

Rebecca Walwyn, Alex Wright-Hughes, Amanda Farrin, Robbie Foy
University Of Leeds, Leeds, United Kingdom
*Trials* 2019, **20(Suppl 1):**P-279

**Introduction:** Complex interventions are often defined as interventions containing several potentially interacting components. While this definition recognises, they have multiple components, typically complex interventions are treated as black boxes, with randomised evidence built up on whether one package works relative to others. Updated MRC guidance on developing and evaluating complex interventions explicitly includes the possibility of empirical optimisation. Collins has argued that, in order to do this, factorial trial designs can be used to estimate the individual and combined effects of components of a complex intervention. Fisher claimed that one benefit of a factorial design is its wider inductive basis, enabling intervention components to be evaluated under a variety of contexts.
**Methods:** Using a trial with a fractional factorial design that aims to optimise the outputs of National Clinical Audits (ENACT), we will describe statistical challenges in designing a randomised screening trial under the Multiphase Optimisation Strategy (MOST) championed by Collins. We will outline the sample size calculation, the choice of components to evaluate, the choice of combinations of components to include in the design, the randomisation method and the statistical analysis plan.
**Timing of Potential Results:** The trial is expected to be ready for analysis in June 2019.
**Potential Relevance & Impact:** A potential barrier to the use of factorial designs in empirically optimising complex interventions is the recommendation that factorial designs are only used in clinical trials if it is safe to assume that there will be no interactions or if the trial is powered to detect realistic interactions. This view grew out of the proposal to use factorial designs as a way of providing multiple answers for the price of one. The impact has been that factorial trials are largely avoided if interactions are plausible, a situation expected where the factors are components of a complex intervention

## P-280

### Poor randomisation methods are associated with measures of bias: review of recent trials published in four general medical journals

Alex Mitchell[1], Thirimon Moe Byrne[2], Rachel Cunningham-Burley[1], Alexandra Dean[1], Aditi Rangan[3], Jenny Roche[1], David J Torgerson[1]
[1]York Trials Unit, University of York, United Kingdom; [2]Department of Health Sciences, University of York; [3]Newcastle Medical School, Newcastle University
*Trials* 2019, **20(Suppl 1):**P-280

**Introduction:** The correct use of random allocation in trials removes the risk of selection bias by ensuring researchers cannot allocate patients with certain characteristics to a particular treatment group. Randomisation will only remove the risk of selection bias if the allocation schedule is concealed from the recruiting researcher. However, some allocation concealment methods are better than others. We assessed whether there was evidence of a relationship between concealment quality and internal validity.
**Methods:** We identified 352 eligible papers. We hand searched four major medical journals for individually randomised controlled trials published in the years 2017 and 2018. For each article, we extracted summary statistics on age for each treatment group, and the p-value corresponding to the primary analysis. We categorised articles according to whether the allocation concealment was good (227; 64.5%), adequate (66; 18.8%), inadequate (48; 13.6%) or unclear (11; 3.1%). For each category we calculated the amount of heterogeneity in the standardised age difference between treatment groups using the $I^2$ statistic, and graphically displayed the distribution of p-values. We compared parametrically the likelihood of studies that used inadequate concealment reporting a statistically significant result to studies that used good or adequate concealment.
**Results:** We found that trials that used inadequate concealment were more likely to report statistically significant findings than trials that used good or adequate methods (OR 1.95; 95% CI: 0.95 to 4.04; p= 0.07). We found that for good, adequate and inadequate trials the value of $I^2$ was 0%, 1.0% and 31.1% respectively.
**Conclusion:** We have found a relationship between concealment quality and the p-value of the primary outcome. Trials that use inadequate concealment are more likely to have statistically significant p-values compared with trials using good or adequate methods, and there is evidence that the imbalance in age in inadequate trials is not due to chance alone.

## P-281

### Optimal randomisation strategies – simple, stratified or minimised: lessons from the WHIST trial

Ruth Knight[1,2], Nicholas Peckham[1,2], Matthew L Costa[3], Susan J Dutton[1,2]
[1]Oxford Clinical Trials Research Unit (OCTRU), Nuffield Department of Orthopaedics Rheumatology and Musculoskeletal Sciences (NDORMS), University of Oxford, Oxford, UK; [2]Centre for Statistics in Medicine (CSM), Nuffield Department of Orthopaedics Rheumatology and Musculoskeletal Sciences (NDORMS), University of Oxford, Oxford, UK; [3]Oxford Trauma, Nuffield Department of Orthopaedics Rheumatology and Musculoskeletal Sciences (NDORMS), University of Oxford, Oxford, UK
*Trials* 2019, **20(Suppl 1):**P-281

**Introduction:** Random allocation to treatment groups is a key feature of clinical trial design, used to achieve balance between treatment groups on baseline characteristics. Several different randomisation techniques, such as simple randomisation, stratified randomisation, and randomisation by minimisation have been developed and are used in a variety of trial scenarios; however, it is not always clear what the optimal randomisation strategy is for a given trial. The optimal randomisation strategy depends on the planned sample size, and the number of key characteristics (stratification factors) on which we wish to ensure suitable balance. The Wound Healing in Surgical Trauma (WHIST) trial (n = 1629) used randomisation by minimisation with three stratification factors (open versus closed wound at presentation, Injury severity score ≤15 versus ISS ≥ 16, and recruitment centre). The optimal randomisation strategy in this trial is explored.
**Methods:** Anticipated baseline imbalance in the WHIST trial under different randomisation scenarios (simple randomisation, stratified randomisation, and randomisation by minimisation) was investigated. Simulations were conducted to explore the optimal randomisation strategy under a variety of different scenarios including varying numbers of stratification factors and overall sample sizes.
**Results:** In the WHIST trial, randomisation by minimisation led to increased balance on minimisation factors compared with simple randomisation; however, the benefit of this increased balance was small compared to the added complexity of the randomisation system required. Stratified randomisation resulted in decreased balance compared to simple randomisation.
**Discussion:** In some instances, more complex randomisation schemes than needed are used. At other times inappropriate randomisation schemes may lead to unacceptable imbalances in baseline characteristics. Further guidance on this is needed.

## P-282

### Covariate constrained block randomisation for a cluster randomised trial

Kirsty James, Ewan Carr, Sabine Landau, Ben Carter
King's College London, United Kingdom
*Trials* 2019, **20(Suppl 1):**P-282

**Introduction:** Cluster randomised controlled trials require randomisation at the level of the cluster as opposed to the level of the participant. As there are fewer units being randomised than in an individually randomised trial the risk of baseline covariate imbalance is high. Standard methods of stratified randomisation can be employed but are limited to categorical covariates. In an ongoing trial we used stratified covariate constrained randomisation in order to accommodate continuous covariates.
**Methods:** Clusters were identified within catchment areas, 4-6 within each. We required balance in the trial arms for characteristics of the area's service user populations hence the randomisation was stratified by catchment area. In addition, we balanced trial arms for two continuous cluster level covariates; surgery quality and deprivation. The randomisation algorithm, adapted from the work of Carter and Hood, balanced trial arms within and across catchment areas for these two covariates.
**Results:** We randomised 28 clusters from 7 catchment areas (strata). All clusters within a stratum were supplied as a set over the course of the randomisation period. Every time the covariate information on clusters of a stratum became available the algorithm worked out all possible cluster assignments within the stratum and constructed a balancing index based on the clusters that have been randomised so far. An assignment is then chosen at random from the best performing allocations in terms of the balancing index to avoid the algorithm becoming deterministic.
**Discussion**: There were several added complexities in using this randomisation technique in terms of performing the allocations as it was a bespoke algorithm executed by the statisticians. This method does require all cluster information within a stratum to be provided at once which could be a limitation. Outside of this the algorithm allowed the flexibility that was required to balance on continuous covariates in a reliable way.

## P-283

### Key methodological issues for the design and delivery of placebo-controlled randomised trials of invasive procedures, including surgery: a systematic review

Sian Cousins[1], Carmen Tsang[1], Natalie Blencowe[1,2], Ava Lorenc[1], Katy Chalmers[1], Andrew Carr[3], Marion Campbell[4], Jonathan Cook[3,5], David Beard[3,5], Jane Blazeby[1,2]
[1]National Institute for Health Research (NIHR) Bristol Biomedical Research Centre Surgical Innovation Theme, Bristol Centre for Surgical Research, Department of Population Health Sciences, University of Bristol, 39 Whatley Road, Clifton, Bristol, BS8 2PS, UK, United Kingdom; [2]University Hospitals Bristol NHS Foundation Trust, Bristol, UK. BS2 8HW, United Kingdom; [3]Nuffield Department of Orthopaedics, Rheumatology and Musculoskeletal Sciences, National Institute of Health Research (NIHR) Biomedical Research Centre, University of Oxford, Headington, Oxford, UK, United Kingdom; [4]Health Services Research Unit, University of Aberdeen, Aberdeen, UK, United Kingdom; [5]Royal College of Surgeons (England) Surgical Interventional Trials Unit (SITU), Botnar Research Centre, University of Oxford, Headington, Oxford, UK, United Kingdom
*Trials* 2019, **20(Suppl 1):**P-283

**Introduction:** Placebo-controlled trials are rarely used to evaluate invasive procedures. As well as ethical issues to address, there are methodological challenges related to their conduct. Our systematic review examined key methodological considerations for using an invasive placebo intervention in a randomised controlled trial (RCT).
**Methods:** Any RCT comparing an invasive procedure with a placebo was eligible for inclusion. Articles published up to 31st December 2017 were retrieved from Ovid MEDLINE, Ovid EMBASE and CENTRAL electronic databases, hand searching references and expert knowledge.
Data were extracted on trial characteristics and key methodological areas - i) rationale for using invasive placebo interventions; ii) delivery of co-interventions in the placebo group; iii) intervention standardisation and fidelity, including offering the treatment intervention to patients randomised to placebo; v) information provision; vi) minimisation of risk.
**Results:** Identified were 113 articles reporting 96 RCTs. Most trials were conducted in gastrointestinal surgery (n=40, 42%) and evaluated minimally-invasive procedures (n=44, 46%). Over two thirds randomised fewer than 100 patients (n=65, 68%) and a third were single centre (n=21, 22%).
Approximately a third (n=33, 34%) did not report any rationale for using a placebo. Co-interventions were inconsistently reported, but 64 trials (67%) stated that anaesthesia was matched between groups. Attempts to standardise interventions and monitor their delivery were reported in few trials, (n=7, 7%) and (n=4, 4%) respectively. Treatment interventions were offered to patients randomised to placebo in 43 trials (45%). Provision of patient information regarding placebo use was infrequently reported (n=11, 11%). Most common strategies to minimise patient risk were operator skill (n=22, 23%) and independent data monitoring (n=28, 29%).
**Discussion:** Most placebo-controlled trials evaluated minimally-invasive procedures. Reporting of trial methodology was inconsistent. Standardised guidance is needed for the design, delivery and reporting of this type of trial to generate high quality evidence to inform clinical practice.

## P-284

### Methodological issues in trials of youth mental health: what have we learnt during the MILESTONE study?

Jane Warwick[1], George Bouliotis[1], Philip Wells[1], Yujin Lee[1], Jason Madan[1], Alastair Cannaway[1], Helena Tuomainen[2], Swaran Singh[2]
[1]Warwick Clinical Trials Unit, University of Warwick, Coventry, UK;
[2]Warwick Medical School, University of Warwick, Coventry, UK
*Trials* 2019, **20(Suppl 1):**P-284

**Introduction:** Discontinuity of care at the child and adult mental health service boundary is present in many European countries. Young people of transition age (typically 16-18) who need ongoing mental health care may be poorly served by the gap between child and adolescent (CAMHS) to adult mental health services (AMHS), left without adequate care or support, at a time when they are undergoing great stress and change (leaving home, for example). We therefore developed a complex intervention, "Managed Transition", to improve outcomes for young people at the child and adult mental health service (transition) boundary and undertook a cluster randomised controlled trial (cRCT) to assess the clinical and cost-effectiveness of "Managed Transition" compared to standard care.
**Methods:** Randomisation was 2:1 (control:intervention) and the cRCT was embedded within a longitudinal cohort study using a novel design. The primary outcome measure was the clinician reported Health of the Nation Outcome Scale for Children and Adolescents (HoN-OSCA) score at 15 months post-intervention. A range of secondary outcomes, including health and health economic measures, were also measured at 9 and 15 months post-intervention.
**Results:** A total of 793 young people from 40 CAMHS in 8 countries were recruited into the trial; 241 to the intervention and 552 to the control arm. Follow-up was completed in September 2018 and the findings are already being used to inform policy and service development in Europe.
**Discussion:** During MILESTONE we faced many challenges. Aside from the usual barriers, recruitment, retention, compliance and so on, we faced a multitude of methodological problems arising from the novel trial design and unique clinical and geographical settings.
In this presentation we share our experience of MILESTONE, using statistical and health-economic results, to illustrate what worked well and what did not, and to make recommendations for the design of future studies.

## P-285

### Challenges and considerations of patient blinding in a surgical trial: the UNBLOCS RCT of benign prostate surgery

Jo Worthington[1], J Athene Lane[1], Paul Abrams[2], Grace Young[1], Hilary Taylor[1], Sian M Noble[1], Sara T Brookes[1], Nikki Cotterill[2], Tobias Page[3], K. Satchi Swami[4], Hashim Hashim[2]
[1]University of Bristol, Bristol, United Kingdom; [2]North Bristol NHS Trust, Bristol, United Kingdom; [3]The Newcastle upon Tyne Hospitals NHS Foundation Trust, Newcastle upon Tyne, United Kingdom; [4]NHS Grampian, Aberdeen, United Kingdom
*Trials* 2019, **20(Suppl 1):**P-285

**Introduction:** The UNBLOCS randomised controlled trial (RCT) compared the current gold standard, transurethral resection (TURP), with a new laser technique, thulium vaporesection (ThuVARP), for benign prostate obstruction. The trial aimed to blind patients to their surgical allocation to avoid bias in patient-reported primary and secondary outcomes.
**Methods:** The RCT was conducted in seven hospitals. Patients were randomised 1:1 to TURP or ThuVARP at the point of surgery, whilst under anaesthetic, with patients blinded until completion of follow up. Two co-primary outcomes were measured; a patient reported symptom score and a clinical measure at 12-months post-surgery. Secondary outcomes included quality of life and patient satisfaction.
**Results:** Patient blinding was considered successful. Only 40% of patients believed they knew which operation they had received, with 58% of those predicting ThuVARP and 72% of those predicting TURP correct. However, of those who were correct, 76% guessed.
**Discussion:** Blinding was achieved primarily by randomising patients whilst under anaesthetic and blinding ward staff, with additional measures taken for patients under spinal anaesthetic. Successful blinding allowed the collection of robust and unbiased patient-reported outcomes. However, there were implications for trial conduct. All trial surgeons had to be able to conduct either procedure, and theatres needed to be equipped and staffed for both procedures, resulting in some delayed operations. The decision to list patients as daycase or inpatient had to be taken by sites regardless of surgical arm. This could be considered a limitation, as conversion to daycase was a potential key advantage of the laser, but most sites were reluctant to plan to carry out TURP as a daycase. However, in other respects it could be considered a strength, as sites could not apply pre-conceived ideas on the suitability of each procedure for daycase, and ultimately length of stay was then determined by the patient's recovery.

## P-286

### c-TRAK TN: A randomised trial utilising ctDNA mutation tracking to detect minimal residual disease and trigger intervention in patients with moderate and high risk early stage triple negative breast cancer: a novel design (CRUKE/16/024)

Katie Goddard[1], Lucy Kilburn[1], Ben Jenkins[1], James Morden[1], Sarah Kernaghan[1], Sophie Perry[1], Claire Snowdon[1], Nicholas Turner[2], Judith M Bliss[1], on behalf of the c-TRAK TN Trial Management Group
[1]Clinical Trials and Statistics Unit at The Institute of Cancer Research (ICR-CTSU), London, United Kingdom; [2]The Institute of Cancer Research and The Royal Marsden NHS Foundation Trust, London, United Kingdom
*Trials* 2019, **20(Suppl 1):**P-286

**Introduction:** Triple negative breast cancer (TNBC) has a high risk of recurrence. Previous work has demonstrated that circulating tumour DNA (ctDNA) detection in patients who have completed treatment for early BC is highly predictive of future relapse (Garcia-Murillas et al.Sci.Transl.Med.2015). c-TRAK-TN is the first study to assess whether ctDNA assays have clinical utility in guiding further therapy in TNBC patients.
**Methods:** c-TRAK-TN is a phase II, multi-centre, randomised trial in moderate-/high-risk early-stage TNBC patients, with no evidence of distant metastases, who have completed standard therapy. If tissue screening detects a trackable mutation, patients will undergo blinded 3-monthly ctDNA surveillance, with randomisation on a 2:1 ratio to pembrolizumab or observation triggered by detection of minimal residual disease indicated by a ctDNA positive (ctDNA+) result by 12months. Patients and their treating team will only be unblinded to the ctDNA+ result if randomised to pembrolizumab. As discussed and agreed with patient representatives, keeping the treating team and patient blind to ctDNA+ results aims to avoid unnecessary anxiety in otherwise asymptomatic patients and avoid pressures to restart treatment based on, as yet, an unproven test. Primary endpoints are ctDNA+ detection by 12months and absence of detectable ctDNA or recurrence at 12months after starting pembrolizumab (chosen as a surrogate of treatment efficacy, anticipated to correlate with long-term outcome).
**Timing of potential results:** Recruitment will end within the next year with results expected in 2021.
**Potential relevance and impact:** c-TRAK-TN adds to a growing portfolio of studies assessing ctDNA utility, and will assess whether ctDNA surveillance can predict recurrence and guide treatment in TNBC patients, with potential to change clinical practice. Details of the novel trial design and its rationale, including an unconventional use of blinding and novel endpoints, will be presented with illustrations of trial innovation and efficiencies. Clinical outcome data will not be presented.

## P-287

### When should Factorial Designs be used for Randomised Controlled Trials?

Alexander J Szubert, A Sarah Walker, Mahesh K B Parmar, Ian R White
MRC Clinical Trials Unit at University College London, United Kingdom
*Trials* 2019, **20(Suppl 1):**P-287

**Introduction:** In randomised controlled trials (RCTs), a factorial design compares two (or more) interventions (A, B; designed to affect the same or different mechanisms/endpoints) to control by randomising to control, A-only, B-only or both. Although this often requires fewer resources/participants than (e.g.) two 2-arm RCTs, investigators considering this design need to address several issues.
**Methods:** We surveyed journal articles published between 2000-2018 relating to designing factorial RCTs. Issues to consider were identified based on these and the authors' personal experience.
**Results:** Factorial RCTs are more desirable if: 1) Interventions can be easily co-administered. 2) Potential for interaction (effect of A differing when B administered) is low. 3) Eligibility criteria for A and B are similar. 4) Recruitment is feasible, e.g. no patient preference against B. 5) Each intervention and toxicities associated with it is unlikely to reduce either adherence to (and hence effectiveness of) the other intervention or overall follow-up. 6) Risk of safety issues from co-administration above individual risks of the separate interventions is low: i.e. an AE due to one intervention is unlikely to result in stopping the other intervention. Other considerations include: 7) Blinding: two placebos ("double-dummy") may be necessary; this could enable discontinuing only one intervention for a specific participant. 8) Methodological issues: scale of analysis and statistical model (e.g. for binary endpoints, logistic regression estimates odds ratios and absence of interaction is required on the logit scale), necessity or not of adjustment for multiplicity, sample size inflation if necessary. 9) Funding availability. 10) Regulatory requirements.
**Discussion:** These issues should be considered, especially for the primary endpoint but also for secondary endpoints when designing factorial RCTs. Factorial RCTs should also be considered as alternatives to multi (>2)-arm RCTs, and as extensions to standard 2-arm RCTs, particularly when there may be an opportunity to address additional management questions.

## P-288

### Molecularly Stratified Umbrella Study, Mesothelioma Stratified Therapy (MiST)

Dean Fennell[1], Seid Mohammed[2], Charlotte Poile[1], Aarti Gaba[1], Cathy Richards[1], Peter Well-Jordan[1], Gavin Murphy[2], Cassey Brookes[2], Shaun Barber[2], Amy Branson[1], Amy King[1], Anne Thomas[3]
[1]Leicester Mesothelioma Research Programme, University of Leicester, United Kingdom; [2]Leicester Clinical Trials Unit, University of Leicester, United Kingdom; [3]Leicester Cancer Research Centre, University of Leicester, United Kingdom
*Trials* 2019, **20(Suppl 1):**P-288

**Background:** Malignant Mesothelioma (MM) is a cancer caused by exposure to asbestos arising predominantly from the parietal pleura or peritoneal lining and is associated with predominantly contiguous growth. In the UK, 65,000 people are expected to die between 2001 and 2050, making MM one of the few predicted cancer epidemics. There have been no new approved treatments for mesothelioma for over 12 years. Currently no standard option for relapsed mesothelioma exists, despite several non-randomised phase II trials conducted over the last 3 decades.
**Aim:** To enable acceleration of novel, effective, personalised therapy for improving disease outcomes for patients with MM utilising a platform design.
**Methods:** MiST is a British Lung Foundation funded, multi-arm stratified therapy clinical trial for relapsed MM patients. MiST has three stages: Stage 1 (molecular pre-screeening) aims to enrol 120 patients, Stage 2 (treatment) aims to recruit 26 patients per arm and Stage 3 is genomic profiling. Patients are included in each arm based on their molecular screening as follows: Patients negative for BRCA1 and/or BAP1 and/or prior response to platinum therapy can be allocated to rucaparib (MiST-1); P16 negative patients can be allocated to abemaciclib (CDKN2A) (MiST-2); Patients with any histological subtype can be allocated to receive bemcentinib and pembrolizumab (MiST-3) and PDL1 positive or previously treated in arms 1-3 can be allocated to atezolizumab and bevacizumab (MiST-4).
The primary outcome is disease control rate (DCR) at 12 weeks with secondary outcomes including DCR and objective response rate (ORR) at 24 weeks as assessed by modified RECIST 1.1 (mRECIST1.1).
**Current progress:** MiST began recruitment in February 2019 with 32 screened to date and 17 entered into MiST 1. MiST 2-4 are due to open to recruitment in the next 3-6 months.

## P-289

### Design, Analysis and Reporting of Multi-Arm Trials and Strategies to Address Multiple Testing

Dmitry Gryaznov[1,5], Ayodele Odutayo[2,3,5], Bethan Copsey[3], Paul Monk[4], Benjamin Speich[1,3], Corran Roberts[3], Karan Vadher[3], Peter Dutton[3], Sally Hopewell[3], Matthias Briel[1], Douglas G. Altman[3]
[1]Basel Institute for Clinical Epidemiology and Biostatistics, Department of Clinical Research, University Hospital Basel, Basel, Switzerland; [2]Applied Health Research Centre, Li Ka Shing Knowledge Institute of St Michael's Hospital, Toronto, Canada; [3]Centre for Statistics in Medicine, Nuffield Department of Orthopaedics, Rheumatology and Musculoskeletal Sciences, University of Oxford, Oxford, United Kingdom; [4]Nuffield Department of Orthopaedics, Rheumatology and Musculoskeletal Sciences, University of Oxford, Oxford, United Kingdom; [5]*Contributed equally
*Trials* 2019, **20(Suppl 1):**P-289

**Introduction:** It is unclear how multiple treatment comparisons are managed in the analysis of multi-arm trials. We investigated strategies for managing multiple testing related to primary outcomes in multi-arm trials.
**Methods:** We investigated clinical trial protocols approved by ethics committees in the United Kingdom, Switzerland, Germany, and Canada in 2012 and their corresponding publications. We created a decision tool to determine the need for multiple testing procedures (MTPs) and compared the results of the decision tool to the analysis plan in the protocols. Pre-specified analysis plans in trial protocols were compared to those in corresponding publications.
**Results:** Sixty-four protocols for multi-arm trials were identified, of which 50 involved multiple testing. Nine of 50 trials (18%) used a single-step MTP and 17 (38%) used an ordered sequence of primary comparisons to control the overall type I error. In the 9 trial protocols that used a single-step MTP, 6 (67%) considered an adjustment in their sample size calculation to maintain statistical power and prevent type II error. Based on our decision tool, 45 of 50 protocols (90%) required use of a MTP but only 28 of the 45 (62%) accounted for multiplicity in their analysis or provided a rationale if no MTP was used. The remaining 5 of 50 (10%) protocols did not require MTPs based on our decision tool. There was little difference between industry and non-industry funded trials regarding the use of MTPs when required (Risk Ratio 1.12, 95% CI 0.57-2.22). We identified 30 protocol-publication pairs, of which 20 planned a MTP in the protocol. Four of these 20 trials (20%) did not perform the MTP in the publication and provided no rationale.
**Discussion:** Strategies to reduce type I and type II errors were inconsistently employed in multi-arm trials. Selective reporting of analyses occurred in publications of multi-arm trials.

## P-290

### Longitudinal evaluation of the reporting quality of clinical trial protocols – evidence for improvement?

Dmitry Gryaznov[1], Benjamin Kassenda[1], Erik von Elm[2], Belinda von Niederhäusern[3], Benjamin Speich[1,7], Lars G. Hemkens[1], Stefan Schandelmaier[1], Elena Ojeda Ruiz[2], Ramon Saccilotto[4], Yuki Tomonaga[5], Alain Amstutz[6], Matthias Briel[1]
[1]Basel Institute for Clinical Epidemiology and Biostatistics, Department of Clinical Research, University Hospital Basel, Basel, Switzerland; [2]Cochrane Switzerland, Bern, Switzerland; [3]Clinical Trial Unit, Department of Clinical Research, University Hospital Basel, Basel, Switzerland; [4]Department of Clinical Research, University Hospital Basel, Basel, Switzerland; [5]Epidemiology, Biostatistics and Prevention Institute (EBPI), University of Zurich, Zurich, Switzerland; [6]University of Basel, Basel, Switzerland; [7]Centre for Statistics in Medicine, Nuffield Department of Orthopaedics, Rheumatology and Musculoskeletal Sciences, University of Oxford, Oxford, United Kingdom
*Trials* 2019, **20(Suppl 1):**P-290

**Introduction:** We investigated the reporting quality of randomized clinical trial (RCT) protocols approved by Swiss research ethics committees (RECs), before the introduction of the Human Research Act in Switzerland in 2014 and the publication of the SPIRIT guidelines in 2013, and thereafter based on the SPIRIT checklist. We determined trial characteristics associated with non-adherence to SPIRIT items.
**Methods:** We included all RCT protocols approved by Swiss RECs in 2012 and 2016. For each protocol, we extracted information on general trial characteristics and evaluated for each of the SPIRIT checklist items whether the respective information was reported in the RCT protocol. We calculated the adherence to SPIRIT in terms of the proportion of reported SPIRIT items per protocol and the proportion of trial protocols reporting individual SPIRIT items.
**Results:** We included 183 RCT protocols approved in 2012 and 217 in 2016. No difference was found in the median proportion of reported SPIRIT items between protocols from 2012 (median 74%, interquartile range [IQR], 64%-80%) and 2016 (median 76%, IQR, 69%-82%). Significant improvement was found for non-industry-sponsored protocols (interaction p-value <0.01); the median proportion increased from 65% (IQR, 56%-74%) in 2012 to 76% (IQR, 66%-83%) in 2016, while for industry-sponsored protocols median adherence remained on a high level (79%, IQR 75%-82% in 2012, and 77%, IQR 72%-82% in 2016). Improvement in adherence of non-industry-sponsored protocols was due to an improvement in 23 individual SPIRIT items improving by 10% or more. The following RCT characteristics were significantly and independently associated with lower adherence to SPIRIT: single centre, no support from CTU or CRO, non-industry-sponsoring, and approval in 2012.
**Discussion:** Industry-sponsored RCT protocols were more complete according to SPIRIT than non-industry-sponsored protocols approved in Switzerland in 2012, but non-industry protocols showed moderate improvement when compared to 2016, while industry protocols remained on a high level.

## P-291

### Maximising participation via efficient electronic processes for data capture and consent: the TYPPEX feasibility study (NIHR Programme Grant for Applied Research RP-PG-0616-20003)

Polly-Anna Ashford[1], Maria Leathersich[1], Claire West[1], Martin Pond[1], Erika Sims[1], Clare Knight[2], Ann Marie Swart[1], Jesus Perez[2]
[1]Norwich Clinical Trials Unit, University of East Anglia, Norwich, United Kingdom; [2]Department of Psychiatry, University of Cambridge, Cambridge, United Kingdom
*Trials* 2019, **20(Suppl 1):**P-291

**Introduction:** Low burden, highly efficient trial designs are desirable in settings with limited research capacity, or where large sample size makes traditional consent processes unfeasible. As an example, we present ongoing work from the TYPPEX programme (Tailoring evidence-based psychological therapY for People with common mental disorder including Psychotic EXperiences) in the NHS Improving Access to Psychological Therapy (IAPT) service.

The TYPPEX feasibility study will test a trial design that incorporates electronic health record extraction/linkage, paperless consent and online follow-up to meet the challenge of obtaining outcome data for around 1200 participants across 12 IAPT teams in the planned cluster randomised controlled trial.

**Methods:** The intervention comprises a 3-day training package designed to upskill IAPT CBT therapists. The primary outcome of the definitive trial will compare reliable recovery between service users treated by therapists before and after training.

Participating Trusts will upload clinical data on all eligible service users to Norwich Clinical Trials Unit in a pseudonymised form (a linking identifier having been encrypted using a one-way secure hash algorithm), obviating the need for individual consent. In parallel, TYPPEX-trained therapists will recruit service users to complete questionnaires for health economic analysis, obtaining informed consent and baseline questionnaire responses on a tablet device which has been set-up for secure, unsupervised use (e.g. in a waiting room). Follow-up is via email questionnaires issued automatically at three and six months after baseline.

To maximise the utility of the final dataset, the secure hash algorithm will match questionnaire responses from consented participants with their corresponding clinical record.

**Timing of Potential Results:** Results from the feasibility study in December 2019 will inform the design of the definitive RCT opening in 2020.

**Potential Relevance & Impact:** The TYPPEX study demonstrates efficient electronic processes that could be adapted for future clinical trials.

## P-292

### Inclusive consent processes during a clinical trial of emergency care: The Paramedic Acute Stroke Treatment Assessment (PASTA) Trial

Lisa Shaw[1], Christopher I Price[1], Gary A Ford[2]

[1]Newcastle University, United Kingdom; [2]Oxford University, United Kingdom

*Trials* 2019, **20(Suppl 1):**P-292

**Introduction:** Involving all relevant patients in clinical trials of emergency care is challenging especially when trials commence pre-hospital and patients can lack capacity or early mortality is high. The Paramedic Acute Stroke Treatment Assessment (PASTA) trial was a multi-centre cluster randomised controlled trial which evaluated a pre-hospital initiated enhanced care pathway designed to facilitate access to emergency stroke treatment. The primary outcome was receipt of intravenous thrombolysis which must be administered within 4.5 hours of stroke onset at hospital. Novel approaches to consent were designed and used due to the trial setting, because many stroke patients lack capacity due to effects on neurological function, and inpatient mortality is 15%.

**Methods:** The study design comprised consent after hospital arrival and administration of urgent treatments. To locate eligible stroke patients conveyed by trial paramedics, a systematic identification process consisting of a series of questions answered for all stroke admissions using routine records, was developed. Questions included admission mode, whether the conveying paramedic was study active, stroke onset time. To offer enrolment to all eligible patients six consent procedures were designed, selected according to clinical assessment of neurological impairment affecting communication or capacity, early mortality or discharge.

**Results:** The trial enrolled 1214 patients using the six consent procedures: standard process for patients with capacity who were able to communicate (n=525); 'easy access' materials for patients with capacity but stroke related communication difficulties (n=24); use of a personal (n=327) or a professional (n=95) consultee for patients lacking capacity; an 'early mortality' principal investigator declaration for patients who died before consent approach (n=206); and postal consent added following an amendment for patients discharged before consent approach (n=37).

**Discussion:** Novel consent approaches enabled enrolment of a relevant study population for evaluation of an emergency pre-hospital intervention. This format may be useful for other pre-hospital emergency trials.

## P-293

### An example of the process of prior elicitation from multiple experts in a clinical trial

Nina Wilson[1], Cameron Williams[2], Kevin Wilson[2], John Matthews[1,2]

[1]Institute of Health & Society, Newcastle University, Newcastle Upon Tyne, United Kingdom; [2]School of Mathematics, Statistics & Physics, Newcastle University, Newcastle Upon Tyne, United Kingdom

*Trials* 2019, **20(Suppl 1):**P-293

**Introduction:** When performing a Bayesian sample size calculation (using assurance) or Bayesian analysis we can use a subjective prior distribution based on expert knowledge. If we have multiple experts, we may want to elicit individual priors and combine them into a single distribution using an aggregation technique, for example opinion pooling or Bayesian aggregation. Alternatively, we may want to use group elicitation to obtain a consensus between experts and therefore obtain a single group prior, for example via the Delphi Method or the Sheffield Elicitation Framework (SHELF).

**Methods:** We have completed the process of eliciting prior distributions from medical experts both individually and in a group setting to apply and compare mathematical and behavioural aggregation methods during the planning of a clinical trial looking the early diagnosis of Motor Neurone Disease.

**Results:** The behavioural aggregation technique resulted in consistently more overconfidence than the mathematical aggregation techniques. Under two different proper scoring rules (Brier Score and log likelihood), the rank ordering of the accuracy of the techniques reversed.

**Discussion:** When conducting a prior elicitation in order determine a sample size for a clinical trial the method used must be chosen carefully as it could have a large effect on the final sample size.

## P-294

### Finding the IDEAL approach to funding low frequency but highly invasive surgical interventions in cerebral palsy

Cushla Cooper[1], Tim Theologis[1], Julie Stebbins[2], Nicky Thompson[2], David Beard[1]

[1]SITU, NDORMS, University of Oxford, Oxford, United Kingdom; [2]Oxford University Hospitals NHS Foundation Trust, Oxford, United Kingdom

*Trials* 2019, **20(Suppl 1):**P-294

**Introduction:** Cerebral Palsy (CP) is the most common childhood physical disability, resulting in musculoskeletal deformity. Children frequently receive orthopaedic surgery to improve functional outcomes and minimise decline. Despite increasing use, there is weak evidence on the effectiveness of surgery in improving gait and function. The need for research is not reflected in the appetite to fund this important area. CP clinicians in the UK have tried for 10 years to develop a study to provide quality evidence for current surgical practices.

**Aim:** To explore previous funding applications to inform a strategy for future research on surgical interventions in CP patients.

**Method:** We reviewed feedback from previous funding applications; collated and explored the main points.

**Results:** Previous applications have included: surgical RCTs comparing new surgical techniques with standard practice; and a qualitative study exploring patient and parent perspectives on surgical treatment. Challenges identified include: the cost of a trial for a relatively small patient population; ethical concerns in introducing new treatments to this patient population; length of follow up, and defining the appropriate sample to include.
The review indicated a service evaluation was required: to collate the incidence of surgical interventions was required.
**Discussion:** Well-designed surgical trials are necessary in the field of CP so evidence is available to guide current and new practices. The challenges in doing so are varied. Attempting to provide high level evidence in the form of an RCT was not feasible. There was insufficient information about the standard of practices and patient pathways that exist in the NHS, and there was no clear primary outcome to include. Moving back along the IDEAL Framework, the initial study needed to be an information gathering exercise. From here, an appropriate, pragmatic trial design can emerge, that is attractive to funding bodies and study stakeholders.

## P-295

### Designing Scientifically Valid Infant Formula Trials Which Support Breastfeeding – Outcomes of a Delphi Consensus Project

Katharine Jarrold, Bartosz Helfer, Robert J Boyle
Imperial College London, London, United Kingdom
*Trials* 2019, **20(Suppl 1):**P-295

**Introduction:** Breastmilk substitutes are consumed by 90% of European infants, often at very high volumes per kilogram at a critical developmental stage. In BMS trials, regulators demand high-level BMS exposure from the first weeks of life to prove safety and it is common practice to provide free BMS to participants. This may conflict with supporting the initiation and maintenance of breastfeeding. For example, triallists may randomise during pregnancy or in the early postnatal days, which risks incentivising women to use BMS. We developed specific methodological guidance through a Delphi consensus project to guide the design, conduct, analysis and reporting of BMS trials.
**Methods:** We conducted three Delphi rounds and a consensus meeting from January to October 2018 with experts in clinical trials, breastfeeding support, infant feeding, critical appraisal and BMS regulation. To inform responses to the third round, we systematically reviewed a sample of BMS trials (PROSPERO CRD42018091928) and undertook a BMS industry consultation. Following the consensus meeting, we consulted BMS trial participants and a research ethics committee.
**Results:** An initial 73 criteria, derived from the literature, were sent to 23 experts affiliated with institutions across Europe, North America and Australasia. Key themes discussed at the consensus meeting were research integrity, study designs and their implications for supporting breastfeeding, and definitions of interventions and endpoints. The final guidance contains 57 criteria, including recommendations that randomisation does not occur until a participant expresses an intention to introduce BMS, and participants are offered skilled breastfeeding support from a trained breastfeeding counsellor at this stage. Guidance for reimbursement of BMS was developed.
**Discussion:** This consensus-derived guidance for the design, conduct, analysis and reporting of BMS trials aims to better protect BMS trial participants and better inform the infant nutrition community about the effectiveness and safety of BMS products.

## P-296

### Establishment of a virtual multidisciplinary team (vMDT) to provide central review of eligibility for a phase II/III radiotherapy trial assessing stereotactic body radiotherapy (SBRT) in patients with oligoprogressive non-small cell lung cancer (NSCLC)

Christy Toms[1], Priyanka Patel[3], Lucy Kilburn[1], Deborah Alawo[1], Simon Doran[2], Angela Riddell[3], Fiona McDonald[3], Judith Bliss[1]
[1]Clinical Trials and Statistics Unit at The Institute of Cancer Research (ICR-CTSU), London, United Kingdom; [2]Cancer Research UK Cancer Imaging Centre, The Institute of Cancer Research, London, United Kingdom; [3]The Royal Marsden NHS Foundation, London, United Kingdom
*Trials* 2019, **20(Suppl 1):**P-296

**Introduction:** HALT is a phase II/III trial assessing SBRT treatment of oligoprogressive disease (OPD) in patients with mutation+ advanced non-small cell lung cancer. OPD is a relatively newly described pattern of progression and RECIST criteria cannot be used alone to identify patients with OPD suitable for SBRT. Consistent classification of OPD and confirmation of lesion suitability for SBRT is central to the interpretation and success of HALT. To achieve this the team have developed a bespoke review process allowing remote, real-time, central eligibility assessment.
**Methods:** The HALT virtual multidisciplinary team (vMDT) consisting of clinicians, radiologists and trial team members, convenes remotely on a weekly basis. Treating clinicians may also attend. Via collaboration with the CRUK National Cancer Imaging Translational Accelerator (NCITA) at ICR, registered patients have anonymised radiology and clinical history uploaded centrally to a research PACS (XNAT image repository), which is made available remotely via a web portal. Case review by at least two oncologists and a radiologist located across UK, Europe and Australia occurs remotely utilising widely available commercial software. Entry criteria are scrutinised, and feedback provided to the referring centre.
**Results:** Between 05/01/2018 and 30/04/2019 30 patients have undergone vMDT review; 23 (76%) patients confirmed eligible, 7 (23%) confirmed ineligible. Reasons identified include >3 progressing lesions identified by vMDT, lesion suitability not confirmed, alternative treatment recommended. 22 patients have been randomised to date.
**Conclusion and Potential Impact:** Establishing the vMDT ensures members of the trial and participating site teams develop expertise in identifying OPD collectively and agree the technical SBRT approach per case. Such on-trial learning will be invaluable to the interpretation of results and subsequent development of the proposed international phase III trial. Details of the vMDT rationale, development and process will be presented and the potential impact on international guidelines and wider clinical practice discussed.

## P-297

### The EVIDENCE pilot study- using cluster randomisation of prescribing policy for comparative effectiveness research in primary care

Amy Rogers, Alexander S F Doney, Angela Flynn, Robert W V Flynn, Isla S Mackenzie, Thomas M MacDonald
University of Dundee, Dundee, United Kingdom
*Trials* 2019, **20(Suppl 1):**P-297

**Introduction:** Doctors and patients require relevant and reliable comparative effectiveness data to support shared decision making. The Evaluating Diuretics in Normal Care (EVIDENCE) study will compare medicines using cluster randomisation of prescribing policy and existing clinical prescribing mechanisms. Routinely collected NHS data will be used to link prescribing to mortality and hospitalisations. This pilot study explores the feasibility of applying this method in Scottish NHS General Practices.
**Methods:** GP practices were invited to take part in the study. Randomisation was applied at practice level to allocate a prescribing policy advising first-line choice when choosing a thiazide-type diuretic for hypertension. Existing long-term prescriptions were switched, where necessary, to comply with randomised policy by a study pharmacist. Patients were informed by letter of the study taking place and the potential for a prescription change with the option to discuss with the study team and/or opt-out of the switch.
**Results:** 26 practices have been randomised in 4 NHS board areas (mean list size 6297, range 1808-12778). 5920 patients with hypertension and taking either indapamide or bendroflumethiazide were identified (mean 228 per practice (53-556). 43% of identified patients were male, mean age 69.8 years. Bendroflumethiazide was taken by 79% of patients (63-94% per practice). 5130 patients were suitable for a drug switch. The study generated 139 telephone contacts with patients regarding potential medication switches; less than 3% of patients did not accept medication switching.
**Discussion:** There is a lack of comparative effectiveness evidence to guide prescribing in the NHS. The EVIDENCE methodology harnesses the potential of existing NHS prescribing and data infrastructure to compare commonly used medicines while addressing the limitations of observational research. This pilot study has demonstrated that the approach is feasible and cost-efficient with minimal disruption to existing practice workflows.

## P-298

### A web application for the design of multi-arm clinical trials

Michael Grayling[1], James Wason[1,2]
[1]Newcastle University, Newcastle upon Tyne, United Kingdom; [2]MRC Biostatistics Unit, University of Cambridge, Cambridge, United Kingdom
*Trials* 2019, **20(Suppl 1):**P-298

**Introduction:** Multi-arm clinical trial designs provide an effective means of evaluating several treatments. Given the large number of treatments now available for testing in many disease areas, it has been argued that their utilisation should increase. However, for any given clinical trial there are numerous possible multi-arm designs that could be used and choosing between them can be a difficult task. This task is complicated further by a lack of available easy-to-use software for designing multi-arm trials.
**Methods:** To aid the wider implementation of multi-arm clinical trial designs, we have developed a web application for sample size calculation when designing a multi-arm trial. It is built using the Shiny package in the R programming language.
**Results:** The application supports sample size calculation when using a wide selection of popular multiple comparison corrections and can control several varieties of power. In addition, optimised arm-wise allocation ratios can be determined. It is free to access on any device with an internet browser and requires no programming knowledge to use.
**Conclusions:** The application provides the core information required by statisticians and clinicians to review the operating characteristics of a chosen multi-arm clinical trial design. We hope that it will assist with the future utilisation of such designs in practice.

## P-299

### When routine data is not quite enough: a vertical audit within a massive cluster randomised trial

Jane Daniels[1], Kate Walker[1,2], Eleanor Mitchell[1], Rachel Haines[1], Lucy Bradshaw[1], Reuben Ogollah[1], Jim Thornton[1,2]
[1]University of Nottingham Clinical Trials Unit, United Kingdom; [2]University of Nottingham, Division of Child Health, Obstetrics and Gynaecology, Nottingham, United Kingdom
*Trials* 2019, **20(Suppl 1):**P-299

**Introduction:** The Department of Health commissioned a randomised controlled trial to determine whether routine testing of pregnant women for group B Streptococcus reduces the incidence of neonatal sepsis. A cluster-randomised trial was mandated. Use of routine data was encouraged, enabling a no-consent trial model. An objective was to identify key process factors that maximise the impact of testing. Two tests are available: antenatal microbiology or intrapartum molecular tests, both requiring a vaginal-rectal swab.
**Methods:** Population: all pregnant women intending vaginal childbirth
Intervention: Microbiology or molecular test, with maternal antibiotics given in labour to test positive women.
Comparator: Usual care, antibiotics given to women with clinical risk factors.
Sample size for neonatal sepsis outcome: 320,000 women, from 80 maternity units
Trial design: A prospective two-arm parallel cluster RCT, with a second-level randomisation of the testing units: 20 using microbiology test, 20 molecular test and 40 usual care. This will allow a direct comparison of tests. Both tests are significantly different processes, undertaken at different gestational ages; however, both are accurate and direct effective prophylaxis. The second-level comparison will be underpowered for the primary outcome but will be able to detect a realistic difference in the proportion of missed testing opportunities.
Data source: Routine datasets will be merged to determine the primary outcome and important maternal and neonatal outcomes. However, they do not describe whether tests were performed, antibiotics given in sufficient time to be effective nor whether a woman declines the swab. A vertical audit of 100 women per testing unit will collect this data.
**Discussion:** Trials requiring massive sample sizes can be resource intensive to collect prospective data whilst routine data sources can be more efficient, yet limited in the outcome data available. Supplementing routine data through a vertical audit in a subset of the sample can answer secondary objectives.

## P-300

### Methodology and consent issues in emergency medicine: the ARREST trial in out-of-hospital cardiac arrest

Alexander Perkins[1], Tiffany Patterson[2], Richard Evans[1], Tim Clayton[1], Rachael Fothergill[3], Mark Whitbread[3], Simon Redwood[2]
[1]London School of Hygiene and Tropical Medicine, London, United Kingdom; [2]St Thomas' Hospital, London, United Kingdom; [3]London Ambulance Service, London, United Kingdom
*Trials* 2019, **20(Suppl 1):**P-300

**Introduction:** Randomised trials in emergency medicine are challenging but vital for improving patient care. Obtaining informed consent in such an environment is a particular issue and can be controversial. The ARREST trial is assessing whether out-of-hospital cardiac arrest patients without an obvious cause should be taken to a specialist heart centre or the closest emergency department. This patient group presents specific difficulties: patients lack capacity to consent, presentation is unpredictable, care must not be delayed, and mortality rates can be >50%.
**Methods:** During the set-up of ARREST we accessed the following sources of information: 1) ARREST research team; 2) cardiovascular patient groups; 3) researchers running similar trials; 4) regulatory bodies; and, 5) published literature on research in emergency contexts.
The information that we collected guided the design of the trial with a focus on patient consent, documentation and follow-up.
**Results:** The ARREST trial uses deferred consent with remote online randomisation to enrol patients without delaying care. To minimise the risk of bias, baseline and primary endpoint data are collected on patients who die or are discharged prior to consent. Remote follow-up using health records reduces the burden on the patients and researchers.
Full ethical approval was received in January 2018 and the first patient was enrolled in February 2018. ARREST is recruiting to target and is on track to finish within the projected timelines.
**Discussion:** Deferred consent has been key to the success of ARREST and patients have been receptive. However, further qualitative research into the experience of patients in emergency medicine trials using deferred consent is needed to better understand when it is an appropriate model.
There is a shortfall in high-quality research in challenging environments from cardiac arrest care to humanitarian crisis response. Innovation in consent methods would facilitate research and benefit patient care.

## P-301

### Abstract omitted

## P-302

### Designing clinical trials of herbal products in the UK: lessons from two trials

Catherine Simpson[1], Merlin Willcox[2], Jeanne Trill[2], Jacqui Nuttall[1], Fran Webley[1], Michael Moore[2]
[1]Southampton Clinical Trials Unit, University of Southampton, Southampton, United Kingdom; [2]Primary Care and Population Sciences, University of Southampton, Southampton, United Kingdom
*Trials* 2019, **20(Suppl 1):**P-302

**Introduction:** There are several design issues specific to trials of herbal medicines: regulatory requirements, choice of preparation, dose and control. There is no guidance on conducting clinical trials involving herbal products in the UK's National Health Service, so the regulatory requirement for each trial needs to be determined individually.
**Methods :** Two randomised controlled trials looking at the use of herbal products to give symptom relief in acute infections to reduce antibiotic use were conducted in the UK primary care setting: ATAFUTI (a full-scale trial of Arctostaphylos uva-ursi for acute urine infections) and HATRIC (a feasibility trial of Pelargonium sidoides for acute bronchitis). The trial team met with Pharmaceutical Assessors from the Clinical Trials Unit at the MHRA (Medicines and Healthcare products Regulatory Agency) to determine whether the herbal product was considered a medicinal product and what documentation would be required. Dosage was determined from a literature review. The preparations were chosen according to availability on the market.
**Results:** The regulatory requirements and lessons from the trials will be discussed. In ATAFUTI the herbal product had a marketing authorisation in an EU member state, but was used in a different formulation, and was manufactured and packaged specifically for the trial within the UK. The placebo used was sugar beet fibre in capsules. The herbal product used in HATRIC had been granted a Traditional Herbal Registration certificate by the MHRA. The trial medication (active and placebo) was supplied by the company which manufactures and markets the herbal product in Germany. The feasibility study was very useful in informing choice of presentation of the herbal product for a main trial.
**Discussion:** Designing trials of herbal products is challenging. A decision algorithm is needed to inform the regulatory requirements for future herbal trials. Feasibility studies help to inform the design of larger trials.

## P-303

### Methodological review of evidence of risk of bias in cluster randomised trials

David Torgerson, Laura Clark, Sarah Cockayne, Claire Hirst, Laura Howe, Jenny Roche, Emma Turner, Laura Wiley
University Of York, York, United Kingdom
*Trials* 2019, **20(Suppl 1):**P-303

**Introduction**: Cluster randomised controlled trials are frequently used in health services research and they are generally more difficult to design and conduct than individually randomised studies. Puffer et al (2003) reviewed cluster trials published between 1997-2002 and found that 40% showed signs of bias: mainly through recruitment bias. In a more recent, smaller review, of trials in 2008 Brierley et al found a continuing problem of bias. Similarly, Bolzern and colleagues (2018) noted there was evidence of a continuing problem with selection bias in cluster trials. In this review we aim to replicate the study by Puffer and colleagues to see if more recently published cluster randomised trials have improved their methodology. Furthermore, we will examine statistical evidence for bias using baseline testing of age as well as other indicators of bias.
**Methods** – We have searched the BMJ, Lancet, Journal of the American Medical Association and The New England Journal of Medicine for cluster randomised controlled trials published between 01.01.2014 and 31.12.2018. At least two researchers have performed study selection and data extraction independently. We will undertake a qualitative and quantitative assessment of the likelihood of potential bias in the study samples. In preliminary results we have found continuing evidence of bias in cluster trials.
**Timing of Potential Results** – We will present the full results of our findings by September 2019.
**Potential Relevance & Impact** – We will compare our findings with previously conducted studies related to bias in cluster randomised trials, in order to identify how approaches to reducing the chance of bias have changed over time, if at all. Our aim is that this review will give an insightful update on current practices by summarising how cluster randomised controlled trials are conducted and reported in four major journals.

## P-304

### Pointless pilots? Evaluating lessons learned from the INDICATE study

Molly Glaze, Cushla Cooper, David Beard
University Of Oxford, United Kingdom
*Trials* 2019, **20(Suppl 1):**P-304

**Introduction:** Pilot and feasibility studies are necessary to answer whether a definitive trial can be performed in a clinical area. They are particularly beneficial in research naïve specialties, where there are more areas of uncertainty. These studies act as important decision points for whether or not a definitive trial is practicable. One example of this is the INDICATE study, which showed a definitive trial was not possible.
**Methods:** A pilot study was undertaken to assess whether a randomised trial comparing surgical decompression with a single steroid injection in patients with moderate Carpal Tunnel Syndrome (CTS) was possible in the NHS.
**Results:** The study gave evidence that it was possible to recruit and retain participants, but not at sufficient levels to achieve a successful definitive trial. Two of the five identified sites recruited participants and an extension of four months was needed to complete recruitment.
The clinical pathway exposed several significant barriers to recruitment. There was a high cost of recruiting through primary care, and Clinical Commissioning Groups were unwilling to fund one arm of the trial. The patient pathway was varied, making it difficult to identify the study population. There was also a lack of interest within the surgical community.
**Conclusion:** The results of INDICATE show patients, surgeons and funders need to be engaged to ensure appropriate treatment arms will be accepted, performed and paid for. INDICATE also demonstrated the need for NHS pathways to identify patients in a way that is conducive to research.
These lessons have impacted the design of subsequent pilot studies. Large structural changes are not as easily circumnavigated, and therefore unsuccessful pilots should be noted by those who aim to make clinical care amenable to research.

## P-305

### Achieving high recruitment and data completion rates for a large cluster randomised controlled trial evaluating a falls prevention intervention in care homes (NIHR HTA funded FinCH Trial: 13/115/29)

Erika J Sims[1], Jo Williams[1], Veronica Bion[1], Mes Cecile Guillard[1], Sue Stirling[1], Allan Clark[1], Lisa Irvine[1], Tracy Sach[1], Ann Marie Swart[1], Jane Horne[2], Katie Robinson[2], Kate Robertson[2], Pip Logan*[2], * On behalf of the FinCH Trial Team
[1]University of East Anglia, Norwich, UK; [2]University of Nottingham, Nottingham, UK
*Trials* 2019, **20(Suppl 1):**P-305

**Introduction:** The care home can be a challenging environment in which to undertake multi-centre research with respect to capacity, consultee availability, staff turnover, varying approaches to record keeping and archiving. We report strategies used to maximise recruitment and data collection in the Falls in Care Home (FINCH), a cluster randomised controlled trial evaluating a falls prevention intervention in older age care homes (CH).
**Methods:** Potential difficulties and risks to recruitment and data availability were evaluated with respect to eligibility, consent, outcome data availability within the CH and national datasets, and site resources. Learnings from the FiCH feasibility study*, TMG, PPI and CRN researcher feedback were considered when drafting the protocol, CH Manager, Resident and Consultee information sheets and consent forms, Case Record Form and database design.
**Results:** Pragmatic strategies implemented included: all care home residents were eligible except those at end of life, proxy (CH staff) reported EQ-5D-5L and DEMQOL-P-4D for all residents irrespective of capacity, 3-monthly data collection by RAs; not using personal-consultee reported data; using data from hospital episode statistics.
Ten sites were opened. 87 CH and 1698 residents were recruited. Per CH, an average of 20 (53%) residents (min 8 (9%) and max 65 (71%)) were recruited. For the primary outcome (falls), 100% data completeness was achieved for residents who were living in the CH at time of data collection. For the 633 residents who died or moved out of the CH since the last data collection, falls records were accessible for 379 (59%). Imputation will be used to model missing data. Rapid archival of resident notes was the primary reason for lack of data availability.
**Discussion:** Pragmatic recruitment and data collection strategies are necessary for care home studies. Rapid archiving of paper-based resident records should be considered when designing care home studies.
*Clin Rehabil. 2016 Oct;30(10):972-983

## P-306
### A Pragmatic Phase 4 Randomised Trial to evaluate the Effectiveness of Dapagliflozin compared to Standard of Care in patients with Type 2 Diabetes in Routine Primary Care (DECIDE: NCT02616666)

Rachael Williams[1], Susan Beatty[1], Paula Williamson[2], Peter Fenici[3], Susanna Dodd[2], Jesus Medina[4], John Wilding[2]
[1]Clinical Practice Research Datalink, Canary Wharf, United Kingdom; [2]University of Liverpool, Liverpool, United Kingdom; [3]AstraZeneca, Cambridge, United Kingdom; [4]AstraZeneca, Madrid, Spain
*Trials* 2019, **20(Suppl 1):**P-306

**Introduction:** Dapagliflozin is effective in managing blood glucose and reducing weight, without risk of hypoglycaemic events, in the treatment of type 2 diabetes. It is FDA/EMA approved and recommended by NICE as a combination therapy in standard clinical practice. Whilst dapagliflozin's efficacy and safety have been demonstrated, traditional trials may not fully reflect use in clinical practice or effectiveness in typical treated patient populations. DECIDE is a pragmatic trial investigating the real-world efficacy of dapagliflozin.
**Methods:** DECIDE utilises real-world research data and services provided by the Clinical Practice Research Datalink (CPRD), which routinely collects de-identified patient data from a network of 1,400 GP practices across the UK. Potentially eligible patients are located by searching the CPRD database using broad criteria. Pre-screened patient lists are sent to GP investigators for eligibility checks and subsequent recruitment. Following informed consent, patients are randomised to dapagliflozin or standard of care. Electronic health records are mapped directly into the trial database, enabling combination with investigator eCRFs and patient reported outcomes. DECIDE will enrol 872 patients and follow them for 2 years. The primary outcome is the proportion of patients achieving clinical success, measured by a composite endpoint at 52 weeks including (1) HbA1c reduction ≥0.5%, (2) weight loss ≥2kg, (3) no reported severe or documented hypoglycaemic events, and (4) no switching from, or adding to, the treatment to which the patient was randomised.
**Timing of Potential Results:** The estimated study completion date is 31/12/2021.
**Potential Relevance and Impact:** DECIDE will demonstrate the efficacy of dapagliflozin amongst real-world patients and clinical practice, potentially informing treatment guidelines. The first of its kind, DECIDE will also confirm the efficiencies of using CPRD data and services to inform protocol design, access a large pool of patients, target site selection, and reduce the burden and costs of producing real-world evidence.

## P-307
**Abstract omitted**

## PS1A
### - O1 Conducting Studies Within A Trial (SWAT) – Identifying the Challenges and Offering Solutions

Catherine Arundel, Adwoa Parker, David Torgerson
York Trials Unit - University of York, York, United Kingdom
*Trials* 2019, **20(Suppl 1):**PS1A

**Introduction:** Achieving high participation and retention of participants in RCTs has traditionally been difficult, and this can limit the internal and external validity of a study.
Testing of strategies to improve recruitment and retention of participants is therefore important. Rigorous testing can be completed by nesting or embedding recruitment and retention randomised studies within ongoing trials.
**Methods:** To enable testing of recruitment and retention strategies, the PROMoting THE Use of SWATS (PROMETHEUS) programme, has been funded by the Medical Research Council (MRC). The PROMETHEUS programme aims to support 25 or more SWATs over the lifetime of the study, to increase the routine embedding of SWATs, and so build the evidence base in relation to participant recruitment and retention in trials.
**Results:** In delivering the PROMETHEUS programme, difficulties and challenges specific to the approval, implementation and publication of SWATs have been identified. These include: applying for and obtaining relevant approvals and sponsorship; concerns about the content or format of the recruitment and retention strategies; potential for contamination, resentful demoralisation or impact on other planned trial activities; implementation of strategies; and reporting detail requested in publications. These concerns have arisen across the spectrum of research stakeholders including but not limited to host trials, Research Ethics Committees, sponsors, and journal editors. This presentation will outline the complexities related to the delivery of recruitment and retention SWATs, and will offer possible solutions to these problems.

## PS1A
### - O2 Same intervention, different opinions: some challenges of doing Study Within A Trial (SWAT) replication studies

Anne Duncan[1], Kirsteen Goodman[2], Suzanne Hagan[2], Shaun Treweek[1]
[1]Health Services Research Unit, University of Aberdeen, Aberdeen, United Kingdom; [2]NMAHP Research Unit, Glasgow Caledonian University, Glasgow, United Kingdom
*Trials* 2019, **20(Suppl 1):**PS1A

**Introduction:** Studies Within A Trial (SWATs) provide evidence to support trial process decisions. We will use the example of replicating the same SWAT intervention in three different trials to illustrate some challenges (expected and unexpected) in doing SWATs.
**Methods:** A theoretically informed cover letter (SWAT24) was found to improve response rates to postal questionnaires in the IQuaD dental trial by 6%. We aimed to see if the benefits of SWAT24 were replicated in other trials.
The INTERVAL (dental), AMBER (MS) and OPAL (urinary incontinence) trials replicated SWAT24, randomising participants to receive either the theoretically informed cover letter or standard cover letter with their follow-up questionnaires. Each trial was required to gain ethical approval, as well as buy-in from local PIs.
**Results:** Approval was given for all three trials. However, review of the same SWAT intervention led to different requirements from ethical committees to gain approval. For OPAL, the committee thought the language coercive and requested changes. There were similar concerns from the committee handling AMBER and a local PI thought the letter would undermine relationships with participants and did not take part. The committee handling approvals for INTERVAL thought the language aggressive. Minor changes were made to text for OPAL, AMBER and INTERVAL. To our knowledge, none of the 1432 participants receiving the SWAT24 letter in IQuaD, INTERVAL,

OPAL and AMBER raised concerns. Meta-analysis shows a pooled increase in response rate of 4% (95% CI=0% to 8%) in favour of the theory-informed letter.
**Discussion:** Replication is key to SWATs. For replication evaluations of a SWAT, it would be efficient for committees and others to have sight of previous ethical judgements, as well as information on response from previous participants. Trial Forge is working with the Health Research Authority on streamlining the approvals process for SWATs in the UK.

## PS1A
### - O3 Two-by-two factorial randomised trial to evaluate strategies to improve follow-up in a randomised prevention trial

Lucy Bradshaw[1], Joanne Chalmers[2], Rachel Haines[1], Hywel Williams[2], Alan Montgomery[1]
[1]Nottingham Clinical Trials Unit, University of Nottingham, Nottingham, United Kingdom; [2]Centre of Evidence-Based Dermatology, University of Nottingham, Nottingham, United Kingdom
*Trials* 2019, **20(Suppl 1):**PS1A

**Introduction**: Failure to collect outcome data in randomised trials is inefficient and can result in bias and loss of statistical power. Further evaluations of strategies to increase retention are required. We assessed the effectiveness of two strategies for retention in a randomised prevention trial using a two-by-two factorial randomised study within a trial (SWAT [SWAT Repository ID 25]).
**Methods:** Parents of babies included in the host trial were randomised to (1) SMS notification prior to sending questionnaires at 3, 6, 12 and 18 months versus no SMS notification and (2) £10 voucher sent with the invitation letter for the primary follow up visit at 24 months or given at/after the visit. The two co-primary outcomes were (1) collection of questionnaire data at interim follow-up times and (2) collection of host trial primary outcome at 24 months during a home/clinic visit with a research nurse.
**Results:** Between November 2014 and November 2016, 1394 participants were randomised: 350 to no SMS + voucher at/after visit, 345 to SMS + voucher at/after visit, 352 to no SMS + voucher before visit and 347 to SMS + voucher before visit. Overall 75% of questionnaires were completed in both the group allocated to the prior SMS notification and the group allocated to no SMS notification (odds ratio (OR) SMS v. none 1.02, 95% CI 0.83 to 1.25). Host trial primary outcome data was collected at a visit for 566 (81%) whose parents were allocated to receive the voucher at/after the visit and for 557 (80%) allocated to voucher before the visit (OR before v. after 0.89, 95% CI 0.69 to 1.17).
**Discussion:** There was no evidence for either strategy increasing retention. The Trial Forge PRIORITY 2 project is currently working to identify the top ten unanswered questions for trial retention to prioritise for future SWATs.

## PS1A
### - O4 Timing of text message reminders to increase trial participant response to postal questionnaires: an embedded randomized trial

Adwoa Parker[1], Stephen Brealey[1], Ada Keding[1], Lucksy Kottam[2], Alex Mitchell[1], Matthew Northgraves[3], Prasanna Partha Sarathy[1], Charlie Welch[1], Amar Rangan[1]
[1]University of York, York, United Kingdom; [2]South Tees Hospitals NHS Foundation Trust, Middlesbrough, United Kingdom; [3]University of Hull, Hull, United Kingdom
*Trials* 2019, **20(Suppl 1):**PS1A

**Introduction:** To evaluate whether text messages sent as pre-notification or post-notification reminders improve questionnaire response rates. This study was embedded in the UK FROST trial of treatments for frozen shoulder in a hospital setting.
**Methods:** Participants were randomised at the three-month follow-up to either text messages: pre-notification on the day of the questionnaire mail-out; or post-notification four days following questionnaire mail-out. The primary outcome was the proportion of participants who returned a valid questionnaire. The secondary outcomes were time to questionnaire return and proportion of participants requiring at least one return reminder. No formal power calculation was conducted. Electronic searches of multiple databases were undertaken to identify other similar embedded trials to perform a meta-analyses.
**Results:** Of 269 embedded trial participants, 122/135 (90.4%) returned a valid questionnaire at three months follow-up in the pre-notification arm and 119/134 (88.8%) in the post-notification arm (difference of -1.6%; 95% CI of difference: -8.9%, 5.7%). There was no statistically significant difference between groups in the rate of return (chi squared test: p=0.67; adjusted regression: OR 0.93; 95% CI: 0.41, 2.08; p=0.85), time to return (median of 13 days and 14 days for pre- and post-notification, log-rank p=0.93) or requirement of a reminder (47.4% and 41.0% for pre- and post-notification, p=0.29). From the electronic searches, one embedded trial of timing of text message reminders in patients with depression in primary care was eligible for pooling with our embedded trial. The unadjusted pooled odds ratio from a random effects model for response rate was 1.28 in favour of post-notification (95% CI: 0.69, 2.37; P=0.44).
**Discussion:** Text messaging is a simple and inexpensive strategy to improve response rates. There was, however, no evidence from this study of different timing of text message reminders being more effective. This is possibly due to the already high response rates.

## PS1A
### - O5 Identifying trial retention uncertainties using a James Lind Alliance Priority Setting Partnership – The PRioRiTy II (Prioritising Retention in Randomised Trials) Study

Katie Gillies[1], Dan Brunsdon[1], Linda Biesty[2], Peter Brocklehurst[3], Valerie Brueton[4], Declan Devane[2], Jim Elliott[1], Sandra Galvin[2], Carrol Gamble[5], Heidi Gardner[1], Patricia Healy[2], Kerry Hood[6], Joan Jordan[7], Doris Lanz[8], Beccy Maeso[9], Amanda Roberts[1], Imogen Skene[10], Derek Stewart[1], Irene Soulsby[1], David Torgerson[11], Shaun Treweek[1], Andrew Worrall[1], Caroline Whiting[9], Sharon Wren[1]
[1]Health Services Research Unit, University Of Aberdeen, Aberdeen, United Kingdom; [2]National University of Ireland, Galway, Ireland; [3]University of Birmingham, Birmingham, United Kingdom; [4]Kings College London, London, United Kingdom; [5]University of Liverpool, Liverpool, United Kingdom; [6]Cardiff University, Cardiff, United Kingdom; [7]EUPATI; [8]Queen Mary University London, Londond, United Kingdom; [9]James Lind Alliance, Southampton, United Kingdom; [10]Barts and St Thomas NHS Trust, London, United Kingdom; [11]University of York, York, United Kingdom
*Trials* 2019, **20(Suppl 1):**PS1A

**Introduction:** One of the top three research priorities for the UK clinical trial community is to address the gap in evidence-based approaches to improving participant retention to clinical trials. Despite this, there is little evidence supporting methods to improve retention. This presentation outlines the PRioRiTy II project, a Priority Setting Partnership (PSP) that identified unanswered questions and uncertainties around trial retention and then prioritised those in collaboration with key stakeholders.
**Methods:** There were three stages: (i) an online survey consisting of six open ended questions eliciting any questions or comments respondents had about retention to clinical trials. (ii) A second online survey where stakeholders were invited to select the questions from the longlist they felt needed to be answered. (iii) A face-to-face consensus workshop, where key stakeholder representatives finalised an ordered priority list of unanswered research questions for methods of retention to clinical trials.
The research project had a strong and effective patient & public involvement throughout the piece.
**Results:** 456 respondents yielded 2,431 answers to six open ended questions, from which 372 verbatim questions were identified. This allowed us, through our analysis and by merging questions in consultation with the steering group, to create a list of 27 questions. The top 21 questions from the second online survey were brought forward to a face-to-face consensus meeting, in which key stakeholder representatives prioritised the Top 10 list of research priorities for retention, which will be highlighted during this presentation.
**Conclusion:** The Top 10 priority list of unanswered questions about trial retention forms an agenda for trial methods research. It can be used by funders and researchers to ensure that focus is given to the areas of most importance in improving retention in trials.

## PS1B
### - O1 Nature and impact of time-to-treatment measurement error in clinical trials where early administration is essential

Raoul Mansukhani[1], Ian Roberts[1], Linda Sharples[2]
[1]Clinical Trials Unit, London School of Hygiene & Tropical Medicine, United Kingdom; [2]Department of Medical Statistics, London School of Hygiene & Tropical Medicine, United Kingdom
*Trials* 2019, **20(Suppl 1):**PS1B

**Background:** In trials of interventions for patients at very high risk of haemorrhaging, delays in treatment result in reduction in the effectiveness of the intervention. However, estimates of time to treatment often rely on clinician assessment, which is subject to mismeasurement and which may result in bias in the estimate of treatment effect (due to the interaction between delay and treatment effect). In such situations source data verification (SDV) of the time to treatment is undertaken for a sample of the trial participants. Motivated by a large international trial involving trauma patients (CRASH-3), this work aims to demonstrate the potential for bias due to mismeasured covariates; available approaches for addressing mismeasurement using SDV are discussed and the choices and assumptions necessary for different methods are clarified.
**Methods:** Through simulations and analysis of the CRASH-3 trial we describe existing statistical approaches to modelling the relationship between outcomes, clinician reported time to treatment and SDV time; these include regression calibration, multiple imputation for missing data and full Bayesian analysis. Specific issues to be addressed are sampling of patients for SDV, whether there is a gold standard measurement or not, whether the observed data is both biased and mismeasured and the (potentially) skewed distribution of observed times.
**Timing of potential results:** Patient recruitment is complete for the CRASH-3 trial. Our analytic work is ongoing and will be completed during summer 2019.
**Potential relevance and impact:** Source verification of data is expensive and time consuming, especially in large multi-centre trials. The impact of correcting for error in clinician reported time will provide more accurate estimates of treatment effectiveness to policymakers and clinicians and can be used to determine the amount of SDV required.

## PS1B
### - O2 Impact of the hazard rate on pre-specified methods of analysis in the presence of time-dependent treatment effects

Kim Jachno, Stephane Heritier, Rory Wolfe
Monash University, Melbourne, Australia
*Trials* 2019, **20(Suppl 1):**PS1B

**Introduction:** Long trial duration, composite endpoints, and complex interventions, are all reasons for clinical trials to anticipate time-varying effects of treatment. The exact nature of the time-dependence of a treatment effect may be hard to anticipate at the design stage of a trial, for example a lag to effect may be expected but the timing of that lag may be unclear. Various analysis approaches may be pre-specified in trial protocols in this instance such as persevering with a Cox proportional hazards model to estimate a single overall hazard ratio, employing an accelerated failure time model to estimate an acceleration factor, or undertaking a comparison of restricted mean survival time based on a parametric Royston-Parmar model.
**Methods:** We undertook simulation studies to quantify the extent to which standard approaches may provide misleading insights to the effect of treatment under different scenarios of time-dependency in the form of a simple piecewise lag to effect. We examined the sensitivity of results to the shape of the underlying hazard.
**Results:** We found that the timing of when events occur affects the degree to which the methods of analysis were misleading, i.e. that the shape of the underlying hazard function in the control group is influential. The longer the lag to effect, the more influential was the shape of the hazard in determining the relative merits of the different analytical approaches that were considered.
**Discussion:** These results highlight the complexity of designing trials and selecting analytical methods for pre-specification if time-dependent effects of treatment are to be anticipated but it is not clear exactly what form that dependency will take.

## PS1B
### - O3 An evaluation and application of statistical methods designed to analyse adverse event data in RCTs

Rachel Phillips[1], Victoria Cornelius[1], Odile Sauzet[2]
[1]Imperial College London, London, United Kingdom; [2]Epidemiologie & International Public Health, Faculty of Health Sciences, Universität Bielefeld, Bielefeld, Germany
*Trials* 2019, **20(Suppl 1):**PS1B

**Introduction:** Randomised controlled trials are considered the ‘gold standard’ for the evaluation of new and existing medicinal products. Consequently, the methods to analyse and report efficacy outcomes are well developed. This progress has not been matched for safety data. In recent work we found that trials typically rely on simple tabulations of frequencies and percentages. We aimed to identify whether there are statistical methods available that were specifically designed to analyse safety outcomes in trials.
**Methods:** We undertook a scoping review to identify such methods, systematically searching Medline and EMBASE via OVID and Web of Science and SCOPUS databases. Original methods or the original application of existing methods for the analysis of AEs were included if they incorporated a concurrent comparator group, were suitable for parallel group trials and had undergone peer-review. A taxonomy of methods was developed. Several of these methods were then applied and evaluated in simulated trial data.
**Results:** The search identified 10785 articles of which 44 were eligible. Methods were grouped into those for safety outcomes that were pre-specified in the protocol (n=9) or for safety outcomes that emerge during the study (n=35). Methods were then further categorised as: visual summary (n=8), hypothesis test (n=11), decision-making probability under a Bayesian framework (n=10), or estimation technique (n=15).
Application of two of the Bayesian methods, the Gamma-Poisson and Beta-Binomial models, in simulated datasets has shown adequate power to detect signals when sample sizes exceed 400 per group, regardless of the time of events when using non-informative priors.
**Discussion:** There are many subjective and quantitative signal detection approaches available to analyse pre-specified and emerging safety outcomes. Our review of trials published in journal articles has previously demonstrated that these methods are not used. The reasons for this are unclear and performance measures and barriers to implementation are being explored.

## PS1B
### - O4 Analysis of responder-based endpoints: improving power through utilising continuous components

James Wason[1,2], Martina McMenamin[2], Susanna Dodd[3]
[1]Newcastle University, Newcastle upon Tyne, United Kingdom; [2]MRC Biostatistics Unit, University of Cambridge, Cambridge, United Kingdom; [3]University of Liverpool, Liverpool, United Kingdom
*Trials* 2019, **20(Suppl 1):**PS1B

**Introduction**: Clinical trials often assess effectiveness of interventions through the use of responder-based endpoints. These classify patients based on whether they meet a number of criteria; some of these criteria are whether or not continuous variables take values above or below a threshold. Traditional analyses estimate the proportion of patients who are responders and test for differences between arms.
An alternative method called the augmented binary method utilises information contained within the continuous component(s) to increase the power considerably (equivalent to increasing the sample size by >30%). This method has been proposed in several methodological papers as being useful in solid-tumour oncology and rheumatoid arthritis. However, it could be potentially useful in a much wider variety of disorders.
In this talk we aim to summarise the method and provide results from a review identifying new clinical conditions where it could be used
**Methods:** We reviewed a database from the COMET initiative of physiological and mortality trial endpoints recommended for collection in clinical trials of different disorders. We identified responder-based endpoints where the augmented binary method would be useful for increasing power.
**Results:** We identified 68 new clinical areas where endpoints were used that would be more efficiently analysed using the augmented binary method.
**Discussion**: The augmented binary method can potentially provide large benefits in a vast array of clinical areas. Further methodological development is needed to account for some types of endpoint.

## PS1B

### - O5 Exploring the Hawthorne effect using a balanced incomplete block design in the aspire cluster randomised controlled trials

Michelle Collinson[1], Thomas Willis[2], Robbie Foy[2], Liz Glidewell[3], Suzanne Hartley[1], Paul Carder[4], Stella Johnson[4], Michael Holland[1], Amanda Farrin[1]
[1]Clinical Trials Research Unit, University of Leeds, Leeds, United Kingdom; [2]Leeds Institute of Health Sciences, University of Leeds, Leeds, United Kingdom; [3]Hull York Medical School, University of York, Leeds, United Kingdom; [4]NHS Bradford Districts Clinical Commissioning Group, Bradford, United Kingdom
*Trials* 2019, **20(Suppl 1):**PS1B

**Introduction:** The Hawthorne effect is a non-specific treatment effect: an alteration in behaviour resulting from observation /assessment, leading to an overestimate of intervention effectiveness. If this effect is unbalanced across trial arms, treatment estimates may be biased.
ASPIRE is a NIHR-funded programme evaluating interventions to promote adherence to quality indicators in general practice (GP). Implementation packages were evaluated using electronic health records in two parallel cluster-randomised controlled trials in West Yorkshire GPs.
**Methods:** Balanced incomplete block designs, were chosen to equalise Hawthorne effects whilst maximising power and efficiency. Trial 1 examined the effect of an intervention on adherence to diabetes control and risky prescribing whilst Trial 2 examined blood pressure control and anticoagulation in atrial fibrillation. Within trials, GPs randomised to the intervention for one indicator, acted as control practices for the other intervention and vice versa.
A non-intervention control group was included to allow exploration of Hawthorne effects: GPs randomised to this group received none of the adapted interventions.
If a Hawthorne effect is present, the non-random aspect of differences in intervention effects is attributed to the fact that GPs were aware of being observed and is not attributable to the intervention. We expect the intervention effect in the primary analysis will be smaller than in the secondary analysis utilising the non-intervention control practices.
**Results:** ASPIRE randomised 178 GPs using opt-out recruitment; trial 1=80; trial 2=64; non-intervention control=34. The intervention reduced risky prescribing (OR=0.82, 97.5% CI (0.67–0.99)) but had no statistically significant effect on other primary endpoints. Secondary analysis showed evidence of a Hawthorne effect; OR=0.76, 97.5% CI (0.63-0.92).
**Discussion:** Balanced incomplete block designs incorporating randomised non-intervention controls could inform the interpretation of RCTs, particularly those utilising routinely collected data in implementation research.

## PS1C

### - O1 MRC-NIHR Methodology Guideline Development on Utilising Benefit-Risk Assessments within Clinical Trials

Nikki Totton[1], Steven Julious[1], Dyfrig Hughes[2], Jonathan Cook[3]
[1]University Of Sheffield, Sheffield, United Kingdom; [2]Bangor University, Bangor, United Kingdom; [3]University of Oxford, Oxford, United Kingdom
*Trials* 2019, **20(Suppl 1):**PS1C

**Introduction:** The Medical Research Council (MRC) and the National Institute for Health Research (NIHR) fund randomised controlled trials to provide evidence to inform national policy decisions. Currently, these trials have a primary focus, which dictates the choice of primary outcome. However, there are commonly multiple outcomes of importance to evaluate. Benefit-risk methodology can be included in trials to simultaneously evaluate multiple outcomes by assessing the trade-off and allowing decisions on the most overall beneficial treatment.
Benefit-risk methodology is commonly used within the regulatory setting with much of the available information and guidance relating to regulatory drug trials conducted by innovator pharmaceutical companies. In the context of MRC/NIHR trials, the studies are of health technologies (not just drugs) and often of therapies that are already licensed. To utilise benefit-risk in the MRC/NIHR context requires consideration additionally of economic outcomes, the selection of core outcome measures and trial design features.
The MRC have funded this project as part of their Methodology State-of-the-Art Workshops series with an aim of developing guidance to include benefit-risk within MRC/NIHR funded trials. This aim will be achieved by completing the following objectives:
1.Review current practice of benefit-risk methodology
2.Review available benefit-risk methodologies
3.Achieve expert consensus on the recommended benefit-risk methodologies
**Methods:** The three objectives will be met using the following methods:
1.Web-based survey of current practice,
2.Rapid methodological review,
3.Two-day expert consensus workshop using nominal group technique.
**Timing of Potential Results:** Results from the survey and rapid review plus preliminary headline results from the workshop (held early September 2019) will be available for the ICTMC conference in October.
**Potential Relevance & Impact:** This research will provide guidance for researchers applying to MRC/NIHR funding streams to ensure research is appropriate to support NHS policy decisions.

## PS1C

### - O2 Essential items for a Health Economics Analysis Plan (HEAP): expert Delphi consensus survey

Joanna Thorn[1], Charlotte F Davies[1], Sara T Brookes[1], Melina Dritsaki[2], Ewan Gray[3], Dyfrig Hughes[4], Sian M Noble[1], Stavros Petrou[5], Colin Ridyard[4], Tracey Sach[6], Ed Wilson[6], Borislava Mihaylova[2], Sarah Wordsworth[2], William Hollingworth[1]
[1]University of Bristol, United Kingdom; [2]University of Oxford, United Kingdom; [3]University of Edinburgh, United Kingdom; [4]Bangor University, United Kingdom; [5]University of Warwick, United Kingdom; [6]University of East Anglia, United Kingdom
*Trials* 2019, **20(Suppl 1):**PS1C

**Introduction:** Health Economics Analysis Plans (HEAPs) setting out the proposed analysis in a randomised controlled trial (RCT) currently lack consistency, with uncertainty surrounding the appropriate content. We aimed to develop a list of essential items to include in HEAPs using Delphi methodology to gain consensus.
**Methods:** 72 potential items were extracted from existing HEAPs and an electronic Delphi survey was developed. Expert participants were recruited through a professional mailing list and other health economics contacts. Respondents were asked (round 1) to rate each item on a scale of 1–9 according to how strongly they felt the item should be included in a HEAP, to suggest additional items and to comment on the items. The survey results were scrutinised according to pre-set criteria for inclusion. Round 2 included a reminder of the participant's own scores and summary results from the whole panel; participants were asked to re-rate items. Consensus criteria for inclusion in the final list were predefined as >70% rating an item 7-9 and <15% rating it 1-3 after round 2. A final item-selection meeting was held to scrutinise the results and adjudicate on items for which consensus had not been reached.
**Results:** 62 participants completed round 1 of the Delphi survey. All 72 items were carried forward to round 2, but no new items were added; 48 respondents (77.4%) completed round 2. The expert panel at the final meeting (n=9) agreed that 58 items should be included in the HEAP essential list, moved 9 items to an optional list, and voted to drop 5 items.
**Conclusions:** The study generated 58 core items (e.g. measurement of resource-use data, key assumptions) that were considered essential for inclusion within a HEAP via expert consensus opinion. These essential items form a template HEAP that will facilitate trial-based economic evaluations in RCTs.

## PS1C
### - O3
### Abstract omitted

## PS1C
### - O4 A Bayesian Parametric Approach to Handle Missing Longitudinal Outcome Data in Trial-Based Health Economic Evaluations

Andrea Gabrio[1], Michael Joseph Daniels[2], Gianluca Baio[1]
[1]Department of Statistical Science, University College London, London, United Kingdom; [2]Department of Statistics, University of Florida, Gainesville, United States
*Trials* 2019, **20(Suppl 1):**PS1C

**Introduction**: Trial-based economic evaluations are typically performed on cross-sectional variables, e.g. QALYs and total costs, derived from the responses for only the completers in the study, using methods that ignore the complexities of utility and cost data (e.g. partially-observed data, skewness and spikes at the boundaries of the variables' range). This is an inefficient approach which may discard a substantial proportion of the sample and mislead the final assessment. We present an alternative and more efficient Bayesian parametric approach to handle missing longitudinal outcomes in economic evaluations, while accounting for the complexities of the data.
**Methods:** We specify a flexible Bayesian parametric model for the observed data and partially identify the distribution of the missing data with partial identifying restrictions and sensitivity parameters. We explore alternative nonignorable (i.e. missing not at random) scenarios through different priors for the sensitivity parameters, calibrated on the observed data. Our approach is motivated by, and applied to, data from a trial assessing the cost-effectiveness of a new treatment for intellectual disability and challenging behaviour.
**Results:** The results show the benefits of using our approach compared with a standard cross-sectional model and a considerable impact of alternative nonignorable assumptions on the final decision-making conclusions, suggesting a more cost-effective intervention compared with the results obtained under ignorability.
**Discussion:** Missingness represents a threat to economic evaluations as, when dealing with partially-observed data, any analysis makes assumptions about the missing values that cannot be verified from the data at hand. With respect to the current practice in economic evaluations, which typically assumes ignorability of the missing data mechanism, our approach represents a considerable step forward which allows to conduct sensitivity analysis to alternative nonignorable departures while jointly accounting for the typical complexities of the data.

## PS1C
### - O5 Calculating health utilities from PedsQL quality of life scores for patients with hyperammonaemic disorders

Elsa Marques[5], Fiona E Lithander[1], R Greenwood[2], Elinor Griffiths[2], Anthony J Killard[3,4], Stu Toms[1], Jo White[3], Julia Kan[5], Michael Champion[6], Suresh Vijay[7], Germaine Pierre[8], Anupam Chakrapani[9]
[1]NIHR Bristol Biomedical Research Centre (Nutrition Theme), Bristol, United Kingdom; [2]University Hospital Bristol NHS Foundation Trust, Bristol, United Kingdom; [3]University of the West of England, Bristol, United Kingdom; [4]BreathDX (UK) Ltd, Bristol, United Kingdom; [5]University of Bristol, Bristol, United Kingdom; [6]Guy's and St Thomas' NHS Foundation Trust, London, United Kingdom; [7]Birmingham Children's Hospital, Birmingham, United Kingdom; [8]Bristol Royal Hospital for Children, United Kingdom; [9]Great Ormond Street Hospital, London, United Kingdom
*Trials* 2019, **20(Suppl 1):**PS1C

**Introduction:** Hyperammonaemic (HA) disorders, including urea cycle disorders (UCD), are life-limiting inborn errors of metabolism (IEMs), with long-term consequences. The AMmonia in Breath Evaluation Research study estimated the health-related quality of life (HRQoL) in children with IEMs. This study mapped the children's HRQoL scores to utility scores and compared health utilities for children with and without HAs but other IEMs. Health utilities are used to derive quality adjusted life years (QALYs) that measure HRQoL over time on the same scale across patient groups and conditions, making them a useful policy tool.
**Methods:** Patients in four UK metabolic disease hospital clinics with HAs (cases) and without a HAs but other IEMs (controls). HRQoL was measured using the Paediatric Quality of Life Inventory (PedsQL), and scores were mapped to Euroqol's EQ-5D utility weights, using Khan et al's published mapping algorithms (models 5 and 6). Correlation between the PedsQL and utility scores was studied using Spearman's test. Differences in utilities between patients with HAs and without HAs but other IEMs were estimated using regression adjusting for age, sex, and hospital site.
**Results:** 45 cases (mean age 12.7 years [SD=5.4 years]; 24 females [53%]) and 46 controls (mean 12.9 years [SD 3.5]; 20 females [43%]) had complete PedsQL data. Both mapping algorithms produced very similar distributions of utility scores (average utility of 0.76 [SD=0.21]) and correlated highly (0.93 and 0.97) with PedsQL scores. Using Khan's model 5 algorithm, scores ranged from -0.04 to 0.97; mean utility was 0.70 (SD= 0.26) for cases compared with 0.82 (SD=0.14) in controls. Health utilities in cases were lower than those without HA (-0.146, 95%CI[-0.23, -0.06]).
**Discussion:** PedsQL scores can successfully be mapped into health utilities using Khan's mapping algorithms. Health utilities for patients with HAs is lower compared with patients without HAs but other IEMs.

## PS2A
### - O1 Data Dashboards – a novel approach of accurately tracking and monitoring electronic Case Report Form (eCRF) data return rates and missing data items for ongoing clinical trials, using a combination of data reporting and analysis tools capable of drilling down to data point level

Joshua James Northey, Michael Radford, Tom Maishman, Gareth Griffiths, Susannah Condie
Southampton Clinical Trials Unit, University of Southampton, Southampton, United Kingdom
*Trials* 2019, **20(Suppl 1):**PS2A

**Introduction:** Electronic Case Report Forms (eCRFs) are used to capture and store data collected during the course of a clinical trial. This allows for more accurate and timely data collection which can be easily accessed, checked and analysed. However, tracking eCRF return rates can be time-consuming and inefficient, especially on studies with large numbers of patients or clinical visits. Statisticians are often required to present the number of eCRFs received/expected to data monitoring committees.
The aim of this project was to develop a digital tool, which trial managers can use without statistician input, to help decrease the time required to monitor eCRF return rates and ensure they accurately reflect the status of a trial.
**Methods:** Using a combination of reporting tools available (Business Objects, SAS, and Excel), the structure of the RAVE database is extracted and used to build a hypothetical list of all possible forms that could be entered. A status for each form is derived using information already collected and trial-specific rules. Any eCRFs not expected are excluded whilst fields where data are expected but missing are extracted for reporting. This information is collated into a series of tables and fed into an interactive report, the Data Dashboard, which can be dynamically filtered and used for chasing sites and highlighting missing data trends.
**Results:** The Data Dashboard shows the number of eCRFs received/expected along with their level of completeness, broken down into categories. Performance information can be drilled down to site/subject level. The tool is easy to interpret and does not require statistical involvement.
**Discussion:** Data Dashboards provide accurate and efficient ways to monitor and track eCRFs, and highlight what expected data are missing at any time-point. Focused targeting in regards to querying and chasing of data, at site/patient level, is possible with less time and effort required.

## PS2A
**- O2**
**Abstract omitted**

## PS2A
### - O3 Improving data entry and study compliance efficiently using immediate audit and feedback tools

Katie Banister[1], Mark Forrest[1], Gillian Ferry[1], Craig Ramsay[1], Claire Cochran[1], Fernanda Dias da Silva[1], Pauline Garden[1], Usha Chakravarthy[2]
[1]Health Services Research Unit, University of Aberdeen, Aberdeen, United Kingdom; [2]Queen's University Belfast, Belfast, Northern Ireland
*Trials* 2019, **20(Suppl 1):**PS2A

**Introduction:** The EDNA diagnostic accuracy study is comparing five non-invasive tests for Wet Age-related Macular Degeneration. Unlike a trial with specified follow-up intervals, participants attended hospital monitoring visits (every 1-3 months) for up to 3 years. We collected routine clinical data over the monitoring period in simple case report forms (CRFs) uploaded to the study website. Due to the volume of data expected, we took a pragmatic, semi-automated approach to data monitoring. We report our experiences over the follow-up phase.
**Methods:** We used varied automated feedback to promote study compliant behaviour for data collection. We reassessed the feedback, including wording, timing and web-based tools, regularly after investigator meetings to maximise impact.
Firstly we provided sites with audit and feedback on data completeness throughout the study using a bespoke dashboard. The dashboard highlighted data collection forms in a visual grid using a traffic light system – green, amber and red representing complete, missing or absent data. Clicking the traffic light took the user straight to the relevant CRF. Sites with green dashboards were congratulated in newsletters and investigator meetings. Site payments were only issued once the dashboard display was green.
We developed further web-based monitoring tools, to confirm protocol adherence or track queries. Protocol compliance queries were issued weekly via automated emails, highlighting problems or congratulating the site on having no current queries.
**Results:** Prior to data cleaning, 7600 CRFs from 562 participants at 24 sites were logged on the study website. Less than 5% of CRFs had missing data. Overall protocol compliance in conducting study exit visits was 88%. Study teams found the dashboard system easy to use and a motivating tool.
**Discussion:** Automated feedback to sites is essential when conducting a large scale study. This should be reassessed regularly to optimise audit and feedback and involve and inform collaborators.

## PS2A
### - O4 The importance of communication and team work in achieving high quality data in clinical trials

Laura A Pankhurst[1], Alison J Deary[1], Helen L Thomas[1], Anna Sidders[1], Cara L Hudson[1], Katie Keen[1], Renate Hodge[1], Valerie Hopkins[1], Nick Smith[2], Helen Harizaj[3], Naomi Hayward[4], Beatriz Lopez Santamaria[5], Rachel J Johnson[1]
[1]Clinical Trials Unit NHS Blood and Transplant, Cambridge and Bristol, United Kingdom; [2]Corporate Continuous Improvement Team NHS Blood and Transplant, Leeds, United Kingdom; [3]Medway NHS Foundation Trust, Kent , United Kingdom; [4]St George's University Hospitals NHS Foundation Trust, London, United Kingdom; [5]Evelina London Children's Hospital Guy's and St. Thomas' NHS Foundation Trust, London, United Kingdom
*Trials* 2019, **20(Suppl 1):**PS2A

**Introduction:** During a clinical trial, data queries frequently arise and can be burdensome for the clinical trials unit as well as the research sites. We aimed to improve the data cleaning process in our Clinical Trials Unit (CTU).
**Methods:** A Continuous Improvement (CI) event was held focussing on data cleaning for Data Monitoring Committee (DMC) reports. CTU participants were data managers, statisticians and trial managers. Clinical research nurses from research sites were also invited. Data were gathered in advance on the number of queries raised, touch time and elapsed time for all processes for an existing trial. Documents were collated to map a detailed timeline of events. CI lean tools were used including '8 wastes' and 'the Kano model'. The process was captured using 'A3 thinking'.
**Results:** The process mapping highlighted the sheer volume of steps in the process. Multiple documents led to duplication, errors and wasted effort, with associated frustration. There was a communication gap with research sites, who were unaware of the DMC's scheduling and requirements. Several agreed actions were tested and adopted, and primarily focussed on improved communication across the whole team. These included an infographic for sites to illustrate the data journey throughout a clinical trial. The actions have led to a significant reduction in the number of queries raised, improved response rates from sites, and a reduction in workload for all. The multidisciplinary approach was invaluable in ensuring a successful outcome.
**Discussion:** High quality data are essential for reports to monitoring committees, and this should be communicated throughout each trial. The CTU team have worked collectively to record all data queries in a single source and clear communication with the site research teams has increased responses to data queries. Improved tools and reports are now being implemented for other trials.

## PS2A

### - O5 Current recommendations/practices for anonymising data from clinical trials in order to make it available for sharing: A scoping review

Aryelly Rodriguez[1], Christopher Tuck[1], Marshall F Dozier[2], Ines Mesa Eguiagaray[3], Sandra Eldridge[4], Steff C Lewis[1], Christopher Weir[1]
[1]Edinburgh Clinical Trials Unit (ECTU), Usher Institute of Population Health Sciences and Informatics, the University of Edinburgh (UoE), Edinburgh, United Kingdom; [2]Library & University Collections, Information Services, the University of Edinburgh (UoE), Edinburgh, United Kingdom; [3]Usher Institute of Population Health Sciences and Informatics, the University of Edinburgh (UoE), Edinburgh, United Kingdom; [4]Pragmatic Clinical Trials Unit (PCTU), Blizard Institute, Barts and the London School of Medicine and Dentistry, Queen Mary University of London (QMUL), London, United Kingdom
*Trials* 2019, **20(Suppl 1):**PS2A

**Introduction:** There are increasing pressures for anonymised datasets from clinical trials to be shared across the scientific community, and differing recommendations on how to perform anonymisation prior to sharing. We aimed to systematically identify, describe and synthesise existing recommendations for anonymising clinical trial datasets to prepare for data sharing. This review will inform (if applicable) the development of new recommendations.
**Methods:** We systematically searched MEDLINE®, EMBASE, and Web of Science from inception to 11 February 2019. Any publication reporting recommendations on anonymisation to enable data sharing from clinical trials was included. Two reviewers independently screened titles, abstracts and full text for eligibility. One reviewer extracted data from included papers, which then was sense-checked by a second reviewer. Results were summarised by narrative review.
**Results:** 18 articles were eligible for inclusion. Three distinct concepts are emerging: anonymisation; de-identification; pseudonymisation. In the past there was some ambiguity about these concepts and their differentiation. The most commonly used anonymisation techniques are: removal of direct patient identifiers (e.g. name or address); careful evaluation and modification of indirect identifiers to minimise the risk of identification (e.g. present age instead of date of birth); elimination of superfluous data (e.g. database audit data). Anonymised datasets joined with controlled access (e.g checking requesters are bona fide researchers with a valid research question) was the preferred method for data sharing.
**Conclusions:** There are not any standardised recommendations on how to anonymise clinical trial datasets for sharing, however, this systematic review shows a developing consensus on techniques used to achieve anonymisation. Researchers in clinical trials still consider that anonymisation techniques by themselves are insufficient to protect patient privacy and they need to be paired with controlled access.

Funding
AR by a UoE PhD scholarship supported by Asthma UK Centre for Applied Research (AUKCAR). CJW by NHS Lothian via ECTU

## PS2B

### - O1 Radiant-BC Platform Trial: Development of an efficient multi-arm multi-stage early phase trial of radiosurgery with immunotherapy and systemic therapies in breast cancer patients with brain metastases using a flexible Bayesian framework

Christina Yap[1, 10], Anthony Kong[1], Daniel Slade[1], Richard Jackson[2], Joshua Savage[1], Paul Sanghera[3], Sian Lax[1], Sarah Bowden[1], Richard Fox[1], Kristian Brock[1], Geoff Heyes[3], Susan Short[4], Vijay Sawlani[5], Sarah Jeffries[6], Colin Watts[1], William Greenhalf[2], Charlotte Rawcliffe[2], Valerie Jenkins[7], Kumar Das[8], Jacqueline Shaw[9], Carlo Palmieri[2]
[1]The University of Birmingham, Birmingham, United Kingdom; [2]University of Liverpool, United Kingdom; [3]University Hospital Birmingham, United Kingdom; [4]University of Leeds, United Kingdom; [5]The Queen Elizabeth Hospital, United Kingdom; [6]Cambridge University Hospitals NHS Trust, United Kingdom; [7]University of Sussex, United Kingdom; [8]The Walton Centre for Neurology and Neurosurgery NHS Trust, United Kingdom; [9]University of Leicester, United Kingdom; [10]The Institute of Cancer Research, United Kingdom
*Trials* 2019, **20(Suppl 1):**PS2B

**Introduction:** The conventional process of drug development of testing one experimental therapy in one trial is too slow and expensive. In Radiant-BC, the primary aims are to assess the safety and activity of the combination of radiosurgery and immunotherapy with several 'real world' systemic therapies that a patient's treating physician would utilise in a real-world setting. To do this efficiently, we implemented a flexible, adaptive Bayesian framework to test multiple arms simultaneously, allowing arms to be dropped (due to safety) or added, within a master protocol.
**Methods:** Radiant-BC is a non-randomised, phase Ib/expansion cohort trial with 10 arms currently; non-chemotherapy based (2 arms) and chemotherapy based (8 arms). Tolerability of each treatment arm in the non-chemotherapy based group with radiosurgery and immunotherapy will be assessed using a multi-stage Bayesian safety monitoring approach with up to 20 patients. Within the chemotherapy based group, dose-escalation decisions will be guided by the continual reassessment method (CRM) and time-to-event CRM to obtain the recommended phase II dose (RP2D) of the specific chemotherapy treatment, first with immunotherapy and then with the addition of radiosurgery. A Bayesian decision framework is utilised to assess if there is sufficient evidence of promising activity to recommend for further testing.
**Results:** Visualisation tools are used to define the tailored decision criterions used in the Bayesian design. Operating characteristics demonstrate the ability of the proposed methods to effectively assess safety, obtain the RP2D and recommend an active arm.
**Discussion:** The proposed early phase platform design is a practical, flexible, efficient approach and can be applied more widely to other disease settings. Particularly for dose-finding where accrual is often suspended at several stages to assess safety, having other arms where patients can benefit from other novel therapies will be attractive. This will also make recruitment more seamless and accelerate trial delivery.

**PS2B**

**- O2 Operational challenges of running platform trials – ICR-CTSU experience based on the plasmaMATCH trial**

Claire Snowdon, Sarah Kernaghan, Laura Moretti, Katie Wilkinson, Sue Martin, Grace Elwood, Sarah Fynn, Prof Judith M Bliss
Clinical Trials and Statistics Unit at The Institute of Cancer Research (ICR-CTSU), London, United Kingdom
*Trials* 2019, **20(Suppl 1):**PS2B

**Introduction:** The scientific efficiencies of platform trial designs are widely recognised, however operational challenges are often underestimated. plasmaMATCH, a multi-centre phase IIa platform trial consisting of ctDNA screening and therapeutic components, designed and conducted by the Institute of Cancer Clinical Trials and Statistics Unit (ICR-CTSU), opened within 15 months of grant activation, recruited ahead of target and will report the first 4 treatment cohorts within 3 years of first patient first visit. Many practical challenges were overcome to ensure the trial's success.
**Methods and results:** Ensuring appropriate funding arrangements for the original trial design and subsequent trial adaptations is vital. plasmaMATCH's screening platform was funded by Cancer Research UK with individual treatment cohorts funded by pharmaceutical partners, however this required complex costings and contract negotiations. Reciprocal confidentiality agreements and a bespoke template agreement with consistent terms and conditions for all pharmaceutical partners ensured efficient negotiations. The lack of regulatory framework for platform trials can cause delays in obtaining approvals. Issues encountered during set-up of plasmaMATCH were overcome through dialogue with the regulator. Earlier engagement via a regulatory advice meeting would have been beneficial.
Greater high level project management input is required in delivering platforms trials. Within plasmaMATCH, co-ordination of the screening platform and parallel treatment cohorts, complex amendments and increased on-going site support and training increased demands on trial management resource. Platforms trials require complex data management systems, the development of which led to challenges in meeting trial set up timeline expectations. Implementing amendments has significant ramifications across multiple systems, increasing demands on data management resource. Within plasmaMATCH a single protocol and consistent cohort assessment schedules reduced database complexity.
**Discussion:** The key lesson learnt at the ICR-CTSU is that the delivery of platform trials is resource intensive, however operational challenges can be overcome with sufficient resources and planning.

**PS2B**

**- O3 Designing and implementing a phase II targeted treatment platform study: a modular approach in metastatic Castration Resistant Prostate Cancer (mCRPC)**

Stephanie Burnett[1], Nuria Porta[1], Ajit Sarvadikar[3], Alexa Gillman[1], Penny Flohr[3], Ines Figueiredo[3], Adam Sharp[3], Pasquale Rescigno[2], Johann de Bono[2], Emma Hall[1]
[1]The Institute Of Cancer Research, Clinical Trials & Statistics Unit, London, United Kingdom; [2]The Institute of Cancer Research and The Royal Marsden Hospital NHS Foundation Trust, London, United Kingdom; [3]The Institute of Cancer Research, London, United Kingdom
*Trials* 2019, **20(Suppl 1):**PS2B

**Introduction:** Platform studies that answer more than one scientific question within a common clinical trials infrastructure provide a methodology for efficient clinical trial design. There is evidence for the molecular stratification of treatment for patients with mCRPC and a growing number of putative targeted therapies.
**Methods:** We designed a phase II platform study in mCRPC to deliver multiple targeted treatments via individual, linked protocols. MAESTRO, an overarching molecular stratification profiling protocol, will identify potentially eligible patients with actionable mutations, progressing on standard treatments. PERSEUS1, evaluating pembrolizumab, is the first of a number of planned phase II signal-finding studies.
**Results:** PERSEUS1 (NCT03506997) opened to recruitment in November 2018 as part of a pilot phase at the lead site, with patients molecularly characterised under a separate existing, single-centre, ethics-approved protocol. MAESTRO is a separately funded screening platform requiring ethics but not MHRA approval. To deliver the platform study nationally, MAESTRO and PERSEUS1 will open simultaneously at multiple centres in 2019. Additional phase II study cohorts are in development.
**Discussion:** We pragmatically separated the protocols due to the modular funding framework and to minimise multi-stakeholder negotiations, thus allowing for simpler contractual arrangements with multiple pharmaceutical companies/funders. In turn, this allowed natural separation of databases which facilitates simpler governance, regulatory and reporting arrangements.
Despite the administrative burden of multiple protocols requiring approval at sites, rather than implementing new cohorts as substantial amendments, this approach allows centres to select the downstream phase II trials they have capacity for.
Timing the implementation of future phase II studies relative to the rate of accrual in MAESTRO (recruitment capped by available funding) and the uptake of multiple planned downstream studies by participating centres is crucial for efficient recruitment.

**PS2B**

**- O4 Borrowing of information across similar subpopulations in Bayesian basket trials**

Haiyan Zheng, James Wason
Newcastle University, Newcastle upon Tyne, United Kingdom
*Trials* 2019, **20(Suppl 1):**PS2B

There has been a recent surge of interest in precision medicine, which goes beyond assessing the population-averaged effects of a new treatment in the conventional paradigms of drug development. A target patient population may be stratified into small subgroups using biomarkers. Basket trials provide a framework to evaluate an experimental therapy over the standard-of-care with respect to the subpopulations. However, considerable criticism has been levelled against designs of early basket trials for the low-powered standalone analysis strategies. Approaches such as standard hierarchical modelling may be limited, since the exchangeability assumption may be too restrictive.
We propose a novel methodology for phase II basket trials with several modules, where information from similar modules can inform analysis of a specific module. For each parameter that underpins the treatment effect in a module, a marginal predictive prior (MPP) is specified using information from the other modules; specifically, it combines one-to-one commensurate predictive priors (CPPs) that represent information from other modules. A spike-and-slab prior is considered for the precision parameter of each CPP. To characterise the pairwise commensurability, we compute the Hellinger distance between probability distributions of any two module-specific parameters and obtain a series of normalised weights for combining the CPPs. This leads to a robust MPP that only information from the most consistent modules will be borrowed. Following Bayes' Theorem, the MPP will be updated using the module data to a robust posterior. We illustrate our approach using the design of a trial of primary biliary cholangitis and Parkinson's disease as an example. Simulation results suggest our approach can identify consistent external modules to implement borrowing of information, and thus improves (i) precision of estimates and (ii) statistical power. In cases of modules being completely inconsistent, our approach gives similar results with that of a stand-alone analysis.

## PS2B

### - O5 Bayesian trial monitoring and power estimation in a complex Hepatitis C treatment trial (VIETNARMS)

Leanne McCabe[1], Ian R White[1], Graham S Cooke[2], A Sarah Walker[1]
[1]MRC Clinical Trials Unit at UCL, London, United Kingdom; [2]Imperial College London, London, United Kingdom
*Trials* 2019, **20(Suppl 1):**PS2B

**Background:** Multi-arm trials combined with Bayesian monitoring allow for many interventions to be tested simultaneously and for inferior interventions to be detected early and stopped, preserving both time and resources. However, testing multiple interventions with unknown effects may complicate interim monitoring and affect power. We explored methods for testing the operating characteristics of multi-arm trials with Bayesian monitoring, using a Hepatitis C trial exploring multiple treatment strategies as an exemplar.
**Methods:** VIETNARMS is a 14-arm trial that will factorially randomise patients to two drug regimens, three strategies for shortening treatment duration or control, with or without adjunctive ribavirin for shortened treatment. It will use Bayesian monitoring at interim analyses to detect, and stop recruitment into, unsuccessful arms, defined as >0.95 posterior probability of the true cure rate being <90%. We tested the operating characteristics of the stopping rule, planned the timing of the interim analyses and characterised power at the final analysis using theory and simulation.
**Results:** The probability of stopping an arm incorrectly is always maintained <0.05: arms with very low cure rates (<60%) are very likely (>90% chance) to stop after ~25% patients are recruited and arms with slightly lower than anticipated cure rates (80%) are likely to stop (70% chance) by the end of recruitment. The timings of interim analyses, based on the probability of stopping an arm with cure rate 60-90%, will take place 7, 10, 13, and 18 months after recruitment starts. Assuming an overall cure rate of 95% gives >90% power for almost all intervention cure rates and comparisons.
**Discussion:** The operating characteristics of the stopping rule are appropriate and interim analyses can be timed to detect failing arms at various stages. Bayesian monitoring can be effective in complex multi-arm trials.

## PS2C

### - O1 A hypothesis test of feasibility for external pilot trials assessing recruitment, follow-up and adherence rates

Duncan T. Wilson, Rebecca E. A. Walwyn, Julia Brown, Amanda J. Farrin
Leeds Institute of Clinical Trials Research, University of Leeds, Leeds, UK
*Trials* 2019, **20(Suppl 1):**PS2C

**Introduction:** The power of a large clinical trial can be adversely affected by low recruitment, follow-up and adherence rates. External pilot trials, conducted before a planned definitive trial but on a smaller scale, can be used to estimate these parameters and identify any issues. Pilot trials commonly specify decisions rules which use these estimates to determine if the definitive trial is feasible and should go ahead, but there is little methodological research underpinning how they, or the pilot sample size, should be chosen.
**Methods:** We argue that recruitment, follow-up and adherence rates are of interest primarily in how they affect the power of the definitive trial, and use this power as a quantitative measure of feasibility in a hypothesis test of pilot data. Considering a two-arm parallel group definitive trial with a single normally distributed primary endpoint, we show how appropriate hypotheses for this test can be defined. We suggest a test statistic and provide its sampling distribution, thus defining type I and II error rates, and show how these can be used to inform the choice of pilot trial sample size and stop/go decision rule.
**Results:** We use our method to re-design the TIGA-CUB trial, a pilot trial comparing a psychotherapy with treatment as usual for children with conduct disorders. Our results show that error rates around the conventional levels of 0.05 (type I) and 0.2 (type II) can be obtained using typical pilot sample sizes of between 30 and 50 participants per arm. In comparison to the proposed method, the standard approach of using several independent progression criteria leads to a substantial loss of efficiency.
**Discussion:** A formal hypothesis testing approach to the design and analysis of external pilot trials could lead to improved decision making without requiring any substantial increase in pilot sample size.

## PS2C

### - O2 Strategies to improve recruitment to a trial of less treatment: a mixed methods study of the OPTIMA prelim trial in early breast cancer

Carmel Conefrey[1], Jenny Donovan[1], Sangeetha Paramasivan[1], Robert Stein[2], John Bartlett[3], David Cameron[4], Amy Campbell[5], Janet Dunn[5], Helena Earl[6], Peter Hall[4], Victoria Harmer[7], Luke Hughes-Davies[6], Iain MacPherson[9], Christopher McCabe[13], Andreas Makris[8], Andrea Marshall, Adrienne Morgan[10], Sarah Pinder[14], Christopher Poole[11], Daniel Rae[12], Nigel Stallard[5], Leila Rooshenas[1]
[1]University Of Bristol, United Kingdom; [2]University College London Hospitals, United Kingdom; [3]Ontario Institute for Cancer Research, Canada; [4]Edinburgh Cancer Research Centre, University of Edinburgh, United Kingdon; [5]Warwick Medical School, University of Warwick, United Kingdom; [6]Oncology Centre, Addenbrooke's Hospital, United Kingdom; [7]Imperial College Healthcare NHS Trust , United Kingdom; [8]Mount Vernon Cancer Centre, Mount Vernon Hospital, United Kingdom; [9]Beatson West of Scotland Cancer Centre, United Kingdom; [10]Independent Cancer Patients' Voice, United Kingdom; [11]Arden Cancer Centre, University Hospitals Coventry and Warwickshire, United Kingdom; [12]School of Cancer Sciences, University of Birmingham, United Kingdom; [13]Department of Emergency Medicine, University of Alberta , Canada; [14]Division of Cancer Studies, Kings College London , United Kingdom
*Trials* 2019, **20(Suppl 1):**PS2C

**Introduction:** This study reports recruitment challenges in a demanding breast cancer trial (OPTIMA prelim) involving a 'less treatment arm' and describes strategies employed to successfully overcome them.
**Methods:** A mixed-methods recruitment intervention (the 'QuinteT Recruitment Intervention', QRI) was employed to investigate recruitment difficulties and feed-back iteratively findings to inform changes to recruitment processes during the trial. Quantitative site-level recruitment data, qualitative interviews (n=22) with healthcare professionals, audio-recorded recruitment discussions between oncologists and patients (n=36) and patient-facing documentation were analysed using descriptive, thematic, and conversation analyses. The findings were triangulated to inform a 'plan of action' to optimise recruitment.
**Results:** Despite belief and enthusiasm for OPTIMA prelim, some oncologists' routine practices and perceptions of equipoise appeared to unwittingly inhibit recruitment. A reluctance to deviate from the usual practice of recommending chemotherapy according to tumour clinicopathologic features meant that not all eligible patients were approached. Audio-recorded discussions with eligible patients also revealed how ingrained routine practices undermined recruitment in subtle ways, as some oncologists justified chemotherapy provision before explaining the RCT, and often encountered difficulties in conveying uncertainty/equipoise. To tackle these challenges, individual and group feedback was given to oncologists, using data from their appointments to illustrate communication issues and vignettes to air issues around approaching patients. More general 'tips' documents with suggestions on how to structure discussions, and convey equipoise were disseminated to all recruiters, alongside revisions to the Patient Information Sheet. The recruitment target was achieved, and a before/after analysis showed that the strategies improved recruitment.
**Conclusions:** This is the first study to illuminate the tension between routine practice and the requirements of recruiting to an RCT. Negotiating clinical and research roles is challenging, particularly in cancer trials involving less/no treatment. Iterative investigation and feedback on clinicians' practices can help to overcome these challenges as trials are underway.

## PS2C
### - O3 Development of a complex intervention to support informed decision-making by family members of adults who lack capacity to consent to trials

Victoria Shepherd[1,2], Kerry Hood[1], Mark Sheehan[3], Richard Griffith[4], Fiona Wood[2]
[1]Centre for Trials Research, Cardiff University, Cardiff, United Kingdom; [2]Division of Population Medicine, Cardiff University, Cardiff, United Kingdom; [3]Ethox Centre, University of Oxford, Oxford, United Kingdom; [4]College of Human and Health Sciences, Swansea University, Swansea, United Kingdom
*Trials* 2019, **20(Suppl 1):**PS2C

**Introduction:** Despite an ageing population and rising prevalence of conditions associated with cognitive impairments, adults who lack capacity to consent are under-represented in research. Trials involving adults who lack capacity raise a number of ethical and practical challenges. Participants who are unable to consent require a family member to act as a proxy decision-maker, however, families can experience an emotional and decisional burden as a result. Despite numerous innovations to improve informed consent processes, there are no interventions for proxy decision-makers. We have developed a decision support tool which aims to support families making decisions about research participation on behalf of an adult who lacks capacity to consent.
**Methods:** The intervention was developed using the MRC guidance for the development of complex interventions, which recommends a phased approach using available evidence and theoretical principles. The intervention was informed by a systematic review, analysis of existing information provision, qualitative interviews with families who had acted as proxies, and the development of a theoretical framework. The intervention was iteratively developed in conjunction with lay advisors and relevant stakeholders.
**Results:** Utilising our previous research findings, and applying decision-support development frameworks, we identified the complex intervention components. We developed a decision-support tool which includes information about the proxy's role and the basis for their decision, and uses values clarification and decision-support methods. This is supported by a brief training intervention for the researcher/clinician seeking consent. We conducted acceptability testing with a group of stakeholders which found high levels of acceptability.
**Discussion:** Ensuring the inclusion of under-represented or vulnerable groups in randomised trials is a priority area. A novel intervention has been developed to support families making proxy decisions about research. The decision-support tool is acceptable to users but requires feasibility testing and establishment of outcome measures prior to any future evaluation of its effectiveness.

## PS2C
### - O4 Why is the early intervention development phase for complex health care interventions important? An overview of new guidance

Alicia O'Cathain[2], Elizabeth Croot[2], Edward Duncan[1], Nikki Rousseau[1], Katie Sworn[2], Katrina Turner[3], Lucy Yardley[3], Pat Hoddinott[1]
[1]University Of Stirling, Stirling, United Kingdom; [2]University of Sheffield, Sheffield, UK; [3]University of Bristol, Bristol, UK
*Trials* 2019, **20(Suppl 1):**PS2C

**Introduction:** Good research ideas often do not produce the anticipated results.[1] It is unknown which intervention development processes lead to real world impact on health outcomes as they are seldom published. Is this a missed opportunity for learning? Could there be avoidable waste? The UK Medical Research Council and National Institute of Health Research funded INDEX study aimed to produce guidance for researchers on how to develop and report complex interventions to improve health or health care outcomes.
**Methods:** Evidence was triangulated from: two systematic reviews, qualitative interviews and e-Delphi studies, guided by two international stakeholder workshops. Systematic reviews of i) published methodological approaches to intervention development ii) international primary research studies reporting intervention development, published in 2015-16, to identify and categorise practices. In parallel, qualitative interviews with a diverse sample of developers (clinicians, academics, social scientists) and wider stakeholders (public representatives, funders, journal editors) were analysed iteratively, inductively and thematically. Data triangulation generated 85 items for two e-Delphis with i) experts in intervention development, ii) wider stakeholders, to measure consensus and explore reasons for divergence. All data fed into a logic model and final guidance on intervention development and reporting.
**Results:** An overview of the guidance will be presented. Key principles include: iterative cycles of development with stakeholder input at each cycle; integrate creativity with scientific methods; be open to failure, change, and consider unintended consequences; look ahead to future evaluation and real-world implementation. Novel qualitative insights include: ways to meld the art and the science of design; the meanings and drivers of "success" and understanding divergence of opinion.
**Conclusions:** The guidance provides a comprehensive tool for consideration when undertaking intervention development. Reporting intervention development processes will promote transparency so that in future researchers can link early design decisions to trial outcomes.

#### Reference
1. Chalmers et al, Lancet, 2014: https://doi.org/10.1016/S0140-6736(13)62229-1

## PS2C
### - O5 ORRCA and ORRCA2: A large-scale, international, collaboration to map recruitment and retention literature

Anna Kearney[1], Anna Rosala-Hallas[2], William J Cragg[3], Declan Devane[4], Katie Gillies[5], Nicola L Harman[1], Athene J Lane[6], Paula R Williamson[1], Carrol Gamble[1]
[1]North West Hub for Trials Methodology Research and Clinical Trials Research Centre, Department of Biostatistics, University of Liverpool, United Kingdom; [2]Clinical Trials Research Centre, Department of Biostatistics, University of Liverpool, United Kingdom; [3]Clinical Trials Research Unit, Leeds Institute of Clinical Trials Research, University of Leeds, United Kingdom; [4]HRB-Trials Methodology Research Network and NUI Galway, Ireland; [5]Health Services Research Unit, University of Aberdeen, United Kingdom; [6]ConDuCT-II Hub for Trials Methodology Research, School of Social and Community Medicine, University of Bristol, United Kingdom
*Trials* 2019, **20(Suppl 1):**PS2C

**Introduction:** Addressing recruitment and retention challenges in trials are important priorities for methodological research, but navigating this growing literature is difficult and time consuming. In 2016, ORRCA (www.orrca.org.uk) launched a free, online, searchable, database of recruitment research that is currently being updated with recent publications and extended to include retention research (ORRCA2). We report the latest results including a mapping exercise of trial recruitment and retention literature, assessment of the database impact and lessons learnt from conducting an international, collaborative, methodology project.
**Methods:** Search strategies from relevant Cochrane reviews were tailored to the trial recruitment and retention objectives and to the databases: MEDLINE(Ovid), WoS, Scopus, CINAHL, PyscINFO, and the Cochrane Library. An international team of reviewers were trained and quality assurance approaches introduced. Following abstract screening, full texts were retrieved for potentially eligible articles. Studies evaluating or reporting recruitment or retention strategies and case reports were included. Eligible articles are being mapped against an agreed framework of recruitment or retention domains and categorised by evidence type (e.g. randomised or non-randomised evaluations, studies without evaluation).
**Results:** 68,900 abstracts and 6,028 full texts have previously been reviewed for ORRCA, identifying 3,555 eligible articles. Screening of an additional 14,465 abstracts for ORRCA and 69,740 abstracts for ORRCA2 by 31 reviewers from six countries is nearly complete. Predicted number of articles for full text review are 860 and 3,600

respectively. A cohort of 2010-2017 publications from ORRCA and ORRCA2 will be prioritised and results presented.
**Relevance and Impact:** The database allows identification of effective recruitment and retention solutions tailored to specific trial designs and patient populations. It has supported several publications including two systematic reviews. The recruitment and retention frameworks allow neglected areas to be highlighted for future targeting of research resources. Lessons learnt will help development of other large-scale methodological projects.

## PS2D
### - O1 Agreeing outcomes that matter to patients – co-production of an animation to explain core outcome sets

Heather Bagley[1], Sarah Gorst[1], Rosemary Humphreys[2], Rebecca Craven[2], Christine Vial[2], Paula R Williamson[1], Bridget Young[1], Nicola Harman[1], Elizabeth Gargon[1]
[1]University of Liverpool, Liverpool, United Kingdom; [2]Public contributor, UK
*Trials* 2019, **20(Suppl 1):**PS2D

**Introduction:** The development of standardised core outcome sets (COS), for all trials of effectiveness in a particular condition, can facilitate the comparison of trials and increase the relevance of research. COMET brings together people interested in the development and application of COS and seeks to facilitate the optimal input of patients, members of the public as key stakeholders in COS development. However, engaging patients and other stakeholders in COS development can be challenging. To help stakeholders to understand what COS are, COMET developed a plain language whiteboard animation.
**Methods:** Three public contributors and 5 researchers co-designed the animation, including the narrative approach, scripting, graphics and the evaluation question.
**Results:** The 3-minute animation, which will be screened as part of this presentation, explains from a patient perspective what COS are, why they are needed and how they are developed. The public contributors emphasised the need for the animation to be presented through a patient voice and the group decided to focus on asthma as an exemplar commonly recognised condition.
The animation (http://www.comet-initiative.org/resources/PlainLanguageSummary) has had over 1000 views. Of the 49 people currently responding to our evaluation question: "How well did this video explain core outcome sets to you?" all responded positively. The animation is available with subtitles and has so far been translated into French, Portuguese and Dutch.
**Discussion**: Involving patients in co-designing our animation helped ensure COS were explained in an accessible way. A dissemination strategy for the animation aims to ensure it is used by COS developers and patient organisations to promote meaningful participation in COS.

## PS2D
### - O2 Patient and public involvement (PPI) in trial oversight: an ethnographic study of eight clinical trials

Karen Coulman[1], Alex Nicholson[1], Alison Shaw[1], Anne Daykin[1], Helen Cramer[1], Carrol Gamble[2], Rhiannon Macefield[1], Malcolm E Pick[3], Lucy Selman[1], Gillian W Shorter[4], Matt R Sydes[5,6], Gordon Taylor[3], J Athene Lane[1,3]
[1]MRC ConDuCT-II Hub for Trials Methodology Research, Population Health Sciences, Bristol Medical School, University Of Bristol, Bristol, United Kingdom; [2]MRC North West Hub for Trials Methodology Research, Institute of Translational Medicine, University of Liverpool, Liverpool, UK; [3]Bristol Randomised Trials Collaboration, Population Health Sciences, Bristol Medical School, University of Bristol, Bristol, UK; [4]Institute of Mental Health Science, School of Psychology, Ulster University, Coleraine, UK; [5]MRC Clinical Trials Unit at UCL, Institute of Clinical Trials and Methodology, University College London, London, UK; [6]MRC London Hub for Trial Methodology Research, London, UK
*Trials* 2019, **20(Suppl 1):**PS2D

**Introduction:** Trial oversight is important for trial governance and conduct. Patients and/or lay members of the public are increasingly included in oversight committees, influenced by international patient and public involvement (PPI) initiatives to improve research quality and relevance. However, guidance on undertaking PPI in trial oversight is lacking. We explore how PPI functions in oversight committees and provide recommendations to optimise PPI in future trials as part of a larger study investigating the role and function of oversight committees in trials facing challenges.
**Methods:** Using an ethnographic study design, we observed oversight meetings of eight UK trials and conducted semi-structured interviews with members of their trial steering committees (TSCs) and trial management groups (TMGs) including PPI contributors, trial sponsors and funders. Thematic analysis of data was undertaken, with findings integrated to provide a multi-perspective account of how PPI functions in trial oversight.
**Results:** Eight TSC and six TMG meetings from eight trials were observed. 52 purposively sampled oversight group members, including three PPI contributors, were interviewed. PPI was reported as beneficial in trial oversight, with PPI members contributing a patient voice and advocacy role. However, PPI contributors were not always active at meetings and were sometimes felt to have a tokenistic role, with trialists reporting a lack of understanding of how to undertake PPI. Interviewees highlighted the importance of planning effective strategies to recruit PPI contributors, considering the level of oversight and stage(s) of trial to include PPI, and regular support for PPI contributors by the trial team.
**Discussion:** Consideration should be given at trial design stage on how to recruit and involve PPI contributors within trial oversight, and support and mentorship for both PPI contributors and trialists (in how to undertake PPI effectively). This study further strengthens the evidence base on facilitating meaningful PPI within clinical trials.

## PS2D
### - O3 Patient and Public Involvement in the Delivery of Platform Trials – ICR-CTSU experience

Sarah Kernaghan[1], Claire Snowdon[1], Laura Moretti[1], Katrina Randle[2], Mairead MacKenzie[2], Judith M Bliss[1]
[1]Clinical Trials and Statistics Unit at The Institute of Cancer Research (ICR-CTSU), London, United Kingdom; [2]Independent Cancer Patients' Voice, United Kingdom
*Trials* 2019, **20(Suppl 1):**PS2D

**Introduction:** The delivery of platform trials presents many challenges, particularly where designs are complex, challenge patient pathways, require multiple biopsies or lack direct patient benefit. The Institute of Cancer Research Clinical Trials and Statistics Unit (ICR-CTSU) has found the involvement of patient advocates essential in overcoming some of these challenges, including patient acceptability of trial design and explanation of the complex trial in plain English as exemplified by plasmaMATCH and PHOENIX, complex platform trials in breast cancer.
**Methods and results:** Patient and public involvement (PPI) in trial design was sought at an early stage, with an extensive consultation process undertaken across multiple patient advocate groups and involving targeted forums and NCRI Dragons Den events. This provided invaluable feedback, ensuring alignment of the trial design with the patient pathway and providing reassurance of patient acceptability of the complex designs, collection of multiple tissue and blood samples (a key component of trial design in both plasmaMATCH and PHOENIX) and around issues of patient benefit and recruitment. PPI in patient information sheets and the consent process has been vital in ensuring the complex designs are effectively described in simple language.
Consumer representatives as named collaborators on grant applications has ensured continued engagement throughout trial development, and consumer representation at regulatory advisory meetings and research ethics committees meetings has proved to be a valuable tool in assisting communications about the proposed research, highlighting to regulators and ethics committees the acceptability and importance of the novel trial design to patients. The inclusion of consumer members on Trial Management Groups ensures continued PPI as the trials evolve, which is crucial in platform trials given the dynamic nature of these trials.

**Discussion:** PPI input from an early stage and throughout the lifetime of the trial is vital in designing and delivering complex and innovative platform trials.

## PS2D

### - O4 The 'Schools Teaching Awareness of Randomised Trials (START)' Initiative

Linda Biesty[1,3], Tom Conway[2], Sandra Galvin[2], Patricia Healy[3], Elaine Finucane[5], Declan Devane[1,2,3,4]
[1]School of Nursing and Midwifery, NUI Galway, Ireland; [2]Health Research Board-Trials Methodology Research Network (HRB-TMRN), NUI Galway; [3]Evidence Synthesis Ireland, NUI Galway; [4]Cochrane Ireland , NUI Galway; [5]Centre for Health Evaluation, Methodology Research and Evidence Synthesis, NUI Galway
*Trials* 2019, **20(Suppl 1):**PS2D

**Introduction:** The Health Research Board – Trials Methodology Research Network (HRB-TMRN) in Ireland celebrates International Clinical Trials Day with the help of the younger members of our community through the network's START programme www.startcompetition.com
START is an outreach initiative that incorporates both a competition for primary schools and also a fun activity day https://www.youtube.com/watch?v=zNdhQhWh5Jk
Launched in 2016, START asks children (8-12 years old) to simulate and report their 'very own' randomised trial.
**Aim:** To explore the START initiative from the perspective and experiences of participating children and their teachers.
**Methods:** Using a qualitative descriptive study we conducted eight interviews with teachers and eight focus groups with 63 children. Interviews were recorded, transcribed and analysed using template analysis.
**Results:** Data were grouped into six broad themes; i) START – it caught our attention; ii) it was doing science not just reading about it; iii) we created our trial; iv) we know about trials now; v) START – it's relevant across the primary school curriculum; vi) improving START. The children shared their understanding of trial processes, the "scientific knowledge" they had gained. They spoke of communicating their learning to other children and with their families. START was identified as having the potential to contribute substantially to key aspects of the broader school curriculum. Working in a team, the children were able to see each other's strengths in delivering the trial; a trial they held, with pride, as their own.
**Discussion:** It is accepted across the trial community that taking part in trials can be beneficial for healthcare, but when there is a lack of public understanding around trials, this means recruitment and overall acceptance of trials is limited. By educating young children about the importance of trials, the outreach initiative of the HRB-TMRN (START), contributes to efforts to address this problem.

## PS2D

### - O5 Complexities of informed consent in an emergency, perinatal, cluster-randomised pilot study: The experience of developing the ACROBAT study (Administering Cryoprecipitate in Obstetric Bleeding at an Earlier Time)

Doris Lanz[1], Jahnavi Daru[1], Laura Green[1,2,3]
[1]Queen Mary University of London, London, United Kingdom; [2]Barts Health NHS Trust, London, United Kingdom; [3]NHS Blood and Transplant, London, United Kingdom
*Trials* 2019, **20(Suppl 1):**PS2D

**Introduction:** The ACROBAT study (ISRCTN12146519) is a pilot cluster-randomised trial of early cryoprecipitate administration in major obstetric haemorrhage requiring blood transfusion. ACROBAT introduces a number of complexities around consent. The relative rarity of the condition and urgency of treatment questions the practicality of advance consent. The cluster design, introduced to ensure uniformity of treatment in a highly multidisciplinary setting, limits women's options to opt out. Development of the consent model required in-depth patient and public involvement (PPI).
**Methods:** We developed and discussed the consent model in collaboration with 'Katie's Team', a women's health PPI group, across four meetings between February and December 2018. Discussions centred on:
-Timing of consent including the level of information deemed appropriate for antenatal discussion
-Content of study materials including antenatal information leaflets, posters and participant information sheets (PIS)
-Opinions on handling data for participants losing capacity
-Content and setting for qualitative research interviews
**Results:** Most members accepted full meaningful consent is only feasible post-intervention. However, some suggested that a very generic advance consent might be appropriate; with a few preferring not to be given any information at all, especially in an emergency setting.
Members gave valuable input on wording and presentation of information, including a graphical representation of the intervention and helping to organise the PIS in a more patient-centred way. PPI members were strongly against delegation of consent to a partner where capacity is lost.
Recruitment to the ACROBAT study began in March 2019 and is ongoing. Learning points from the recruitment process will be presented.
**Discussion:** Research in emergency clinical situations can highlight a wide range of opinions among PPI members on key issues, which develop over time and can be highly emotive. This requires thoughtful, iterative engagement and feedback from the trial team to develop practical, acceptable solutions.

## PS3A

### - O1 QuinteT Recruitment Intervention in the By-Band-Sleeve study: trials, tribulations and lessons learnt

Sangeetha Paramasivan[1], Alba Realpe[1], Caroline Wilson[1], Paul Whybrow[2], Graziella Mazza[1], Chris Rogers[1], Jane Blazeby[1], Jenny Donovan[1], on behalf of the NIHR BBS study TMG and investigators
[1]University Of Bristol, Bristol, United Kingdom; [2]Hull York Medical School, Hull, United Kingdom
*Trials* 2019, **20(Suppl 1):**PS3A

**Introduction:** The By-Band-Sleeve (BBS) randomised controlled trial (RCT) (NIHR-HTA-09/127/53) was set up to compare gastric band, gastric bypass and sleeve gastrectomy operations for complex obesity. Recruitment was anticipated to be difficult and the QuinteT Recruitment Intervention (QRI) was aimed at optimising recruitment.
**Methods:** In phase 1 of the QRI, we conducted in-depth semi-structured interviews with healthcare professionals; audio-recorded and observed recruitment consultations; scrutinised screening logs; and analysed data primarily using qualitative research methods. In phase 2, we provided confidential and supportive recruitment feedback/training to recruiters, aimed at collaboratively developing and implementing a plan of improvement.
**Results:** We conducted 35 interviews with healthcare professionals, received audio-recordings of ~2500 patients' consultations, observed 19 consultations, provided 110 feedback/training sessions (29 site-specific, 77 recruiter-specific, four research nurse sessions) and disseminated recruitment tips documents. The feedback/training, alongside tremendous commitment from surgeons and nurses in the centres and the Trials Unit, enabled recruitment in all 12 centres. Randomisation rates varied from 20%-60% of eligible patients. Lower levels of recruitment occurred in centres that encountered insurmountable organisational barriers and when recruiters struggled to implement QRI training, for example to address patients' preferences. Recruitment is now close to completion, but a 15-month costed extension (Jul 2018-Sep 2019) was required to meet the full target of 1341. Many lessons were learnt to improve future QRIs: spreading good practice from leading recruiters to others, better targeting of recruitment training in higher-volume centres, focusing support on particular recruiters rather than on all, and the need for close integration with the Trials Units.
**Discussion:** BBS has recruited the largest number of participants for a bariatric RCT. Notwithstanding the measures used to optimise recruitment, an extension was required to complete recruitment. The QRI continues to evolve and lessons from BBS have added to the knowledge base on recruitment and will enhance future QRIs.

## PS3A

### - O2 TRCPAD: Accelerating Participant Recruitment in AD Clinical Trials

Oliver Langford[1], Michael C. Donohue[1], Gustavo Jimenez-Maggiora[1], Reisa A Sperling[3,4,5], Jeffrey L. Cummings[2], Paul S Aisen[1], Rema Raman[1]
[1]University Of Southern California, San Diego, United States; [2]Cleveland Clinic, Cleveland, United States; [3]Brigham and Women's Hospital, Boston, United States; [4]Massachusetts General Hospital, Boston, United States; [5]Harvard Medical School, Boston, United States
*Trials* 2019, **20(Suppl 1):**PS3A

**Introduction:** Trial Ready Cohort for Preclinical/Prodromal Alzheimer's Dementia (TRCPAD) aims to develop a large, well-characterized, biomarker-confirmed, trial-ready cohort to facilitate rapid enrollment into AD prevention trials. In current trials, screening evaluation, which often includes amyloid PET imaging and disclosure of results, is an expensive and time-consuming process. Preclinical Alzheimer's studies have had more than a 2/3rd amyloid screen fail rate, resulting in prolonged and expensive recruitment.
To achieve our aim we have set 3 objectives:
1)Build an efficient and sustainable recruitment system in order to enroll an initial TRCPAD Cohort.
2)Optimize an innovative, adaptive algorithm to identify the appropriate trial participants.
3)Develop and validate web-based cognitive and functional outcome measures for future clinical trials.
**Methods:** To reduce recruitment time and costs we have developed a web-based registry https://www.aptwebstudy.org/. Individuals complete online cognitive assessments in an unsupervised context to assist in evaluating eligibility for in-person screening and establishment of a trial-ready cohort (TRC). ApoE genotype may be determined from blood or salivary samples collected from the participants. Eligible participants with increased risk for developing AD are referred to affiliated clinical sites for amyloid PET and other assessment and, depending on results, will have the opportunity to participate in AD prevention clinical trials. Enrolment and preliminary longitudinal information from this cohort can be used as run-in data for new trials and will minimize the screen fail rates and the enrollment timeline for new trials.
**Results:** APT webstudy was launched on December 22nd, 2017. Our recruitment rate stands at ~1,000 consented participants per month, with participation throughout the US.
**Discussion:** Participant identification and enrollment into TRC in-clinic assessments are on target to begin before end of 2019. In this presentation, we will describe the approach used to establish this cohort and provide some data on the current status.

## PS3A

### - O3
**Abstract omitted**

## PS3A

### - O4 Can nurse peer support improve recruitment to complex clinical trials? – Experience from the ISCOMAT trial

Suzanne Hartley[1], Jessica Pool[1], Lauren Moreau[1], Emma McNaught[1], Chris P Gale[1], Alison Blenkinsopp[2], Peter Gardner[1], Jonathan Silcock[2], Beth Fylan[2], Yvonne McGill[1], Amanda Farrin[1]
[1]University Of Leeds, Leeds, United Kingdom; [2]University of Bradford, Bradford, United Kindgom
*Trials* 2019, **20(Suppl 1):**PS3A

**Introduction:** Delivery of large, multicentre trials is complex and despite significant resources, many require extensions to recruit to target. Potential recruitment barriers are often not fully understood at trial inception. Reasons for poor recruitment are multifaceted and it is challenging for central trial teams to unpick underlying causes. We describe our experience of using centralised nurse peer support as a strategy to maximise recruitment in a multi-centre cluster randomised controlled trial (cRCT).
**Methods:** ISCOMAT, a NIHR research programme, includes a cRCT to evaluate an intervention to optimise medicines use for heart failure patients following hospital discharge, with target sample size of 2,100 patients from 42 NHS Trusts. Trial recruitment commenced June 2018, with variable recruitment rates observed across sites. Variation appeared unrelated to size of ward; throughput of patients; or differences in patient pathways and staffing structures. In February 2019, we appointed an experienced cardiology research nurse, based with the central team, to provide peer support to sites. Support is provided by phone, email and site visits where required. Details of contact is documented.
**Results:** Engagement with the peer support has been high and well received by research nurses at sites. Immediate benefits of providing peer support included:
•Greater engagement with the recruiting teams;
•Recruitment barriers identified;
•Reduced time to recruit first participant at each site;
•Increased monthly recruitment rates for sites open to recruitment;
•Improvements in other aspects of trial performance resulting from higher engagement.
**Discussion:** Embedding an experienced nurse within a central trial team has been a positive experience, improving engagement between the central trial team and sites. It allows for identification of how local issues impact on trial performance. It should be considered as a cost effective strategy in large multi-centre trials where patient recruitment is complex and variable.

## PS3A

### - O5 Achieving high-volume, low-cost participant screening and enrolment through automation and centralisation: experiences from the T4DM diabetes prevention trial

Karen Bracken[1], Anthony Keech[1], Wendy Hague[1], Gary Wittert[2]
[1]NHMRC Clinical Trials Centre, University of Sydney, Sydney, Australia; [2]Freemasons Foundation Center for Men's Health, Adelaide Medical School, University of Adelaide, Adelaide, Australia
*Trials* 2019, **20(Suppl 1):**PS3A

**Introduction:** Recruitment to diabetes prevention trials is challenging and expensive, involving screening large numbers with low rates of screening success. The T4DM study is a recently completed Australian trial of testosterone to prevent Type II diabetes in high-risk men aged 50 – 74 years. We had to screen a large number of volunteers at few sites and with a low screening budget per participant. We therefore adopted and evaluated a semi-automated approach to screening and enrolment.

**Methods:** A sequential multi-step screening process was implemented: i) web-based pre-screening, ii) laboratory screening through a network of third-party pathology centres, and iii) final on-site screening. Online data collection, computer-driven eligibility checking, and automated, email-based communication with prospective participants were used. Participant screening status, and associated costs and resource use were centrally tracked.
**Results:** Of 19,022 screened participants, 1,007 were randomised. Nearly all (95%) participants chose online over phone-based pre-screening. At peak, 1,403 participants were pre-screened in a single day. On average, 11 staff hours were required for each participant randomised. Screening costs, including both direct and staffing costs, were AUD$1,420,909 (£782,228) in total (AUD$75 (£41) per subject screened and AUD$1,411 (£777) per participant randomised).
**Discussion:** A screening process incorporating centralised, online and automated elements achieved high-volume, low-cost participant screening. Compared to similar, previous trials, screening savings are conservatively estimated at AUD$5,899,126 (£3,247,543); equivalent to 76% of the entire T4DM study budget. Our approach could be adapted for use in other trials seeking to screen large numbers of participants.

## PS3B
**- O1**
**Abstract omitted**

## PS3B
### - O2 Optimising the design and delivery of placebo surgical interventions in randomised controlled trials: The DITTO framework

Sian Cousins[1], Carmen Tsang[1], Natalie Blencowe[1,2], Katy Chalmers[1], Aryan Mardanpour[1], Andrew Carr[3], Marion Campbell[4], Jonathan Cook[3,5], David Beard[3,5], Jane Blazeby[1,2]
[1]National Institute for Health Research (NIHR) Bristol Biomedical Research Centre Surgical Innovation Theme, Bristol Centre for Surgical Research, Department of Population Health Sciences, University of Bristol, 39 Whatley Road, Clifton, Bristol, BS8 2PS, UK., United Kingdom; [2]University Hospitals Bristol NHS Foundation Trust, Bristol, UK. BS2 8HW, United Kingdom; [3]Nuffield Department of Orthopaedics, Rheumatology and Musculoskeletal Sciences, National Institute of Health Research (NIHR) Biomedical Research Centre, University of Oxford, Headington, Oxford, UK, United Kingdom; [4]Health Services Research Unit, University of Aberdeen, Aberdeen, UK, United Kingdom; [5]Royal College of Surgeons (England) Surgical Interventional Trials Unit (SITU), Botnar Research Centre, University of Oxford, Headington, Oxford, UK, United Kingdom
*Trials* 2019, **20(Suppl 1):**PS3B

**Introduction:** Designing and delivering placebo surgical interventions for use in randomised controlled trials (RCTs) is complex. An in-depth understanding of the constituent components of the treatment intervention (to ascertain what should, and should not, be delivered as part of the placebo intervention) is needed. Furthermore, assessment of potential risk to patients and utilisation of strategies to ensure the placebo effectively mimics the treatment are required. To date no guidance exists for the design of placebo surgical interventions.
This study aimed to develop a framework to optimise the design and delivery of placebo interventions in surgical RCTs.
**Methods:** A preliminary framework was developed by expanding the scope of an existing typology that facilitates the deconstruction of surgical interventions into their constituent components. Then, strategies to optimise placebo interventions were identified from published RCTs. Finally, the framework was refined after consultation with key stakeholders in surgical trial methodology, medical ethics and consensus methods.
**Results:** The resultant DITTO framework consists of five stages: Stage 1 - Deconstruct treatment intervention to produce a comprehensive list of components and co-interventions; Stage 2 - Identify critical element believed to provide therapeutic benefit; Stage 3 - Take out critical element; Stage 4 - Think risk, feasibility, and role of placebo in trial when considering inclusion of remaining intervention components; and Stage 5 - Optimise placebo and ensure effective blinding of trial persons, e.g. by use of auditory masking.
**Discussion:** The DITTO framework provides a structure for the design of placebo surgical interventions. It facilitates in-depth analysis of the treatment intervention. It also outlines key considerations regarding the composition of the placebo intervention, including potential risk to patients and the use of placebo optimisation strategies.

## PS3B
### - O3 Clinical trial simulation and value of information to optimise design of clinical trials from a pharmaceutical industry perspective

Daniel Hill-McManus, Dyfrig Hughes
Bangor University, Bangor, United Kingdom
*Trials* 2019, **20(Suppl 1):**PS3B

**Introduction:** The optimal design for a clinical trial using value of information (VoI) methods is the point at which it becomes more costly to collect additional data than the value gained from that data. This requires prior distributions for trial outcomes, either based on past trials or expert opinion. However, a proposed trial cannot typically be expected to produce data consistent with earlier studies. The aim of this study was to demonstrate the utility of using pharmacometric clinical trial simulation (CTS) to address key limitations of current VoI approaches to phase III clinical trial design using gout treatments as a case study.
**Methods:**The methods consist of four principal stages: a CTS to predict the distribution of treatment response rates for a given sample size; a payer model that links a rate of treatment response to an estimate of the maximum reimbursement price a payer would be willing to pay to access the drug; a model of the pharmaceutical company return on investment linking drug prices to sales revenue; and an analysis of the sensitivity of the optimal decision to the uncertainty in specific model parameters using expected value of partial perfect information (EVPPI).
**Results:**The optimal sample size for a single trial comparing febuxostat 80 mg and allopurinol 300 mg once daily was estimated to 500 patients per arm, given assumptions on the incidence of patients and a minimum launch price. EVPPI for each uncertain model parameters indicated that uncertainty in parameters for drug adherence, rather than drug pharmacology, dominated the uncertainty regarding the optimal sample size decision.
**Discussion:** Using clinical trial simulation to generate distributions of trial outcomes removes a key limitation of value of information approaches to trial design, the requirement for prior distributions on outcomes, and EVPPI may focus efforts to reduce uncertainty to specific areas.

## PS3B
### - O4 Two-stage adaptive enrichment designs with time to event data: Point and interval estimation

Peter Kimani[1], Susan Todd[2], Lindsay Renfro[3], Ekkehard Glimm[4], Josephine Khan[5], John Kairalla[6], Nigel Stallard[1]
[1]University of Warwick, Coventry, United Kingdom; [2]University of Reading, Reading, United Kingdom; [3]University of Southern California, California, USA; [4]Novartis Pharma AG, Basel, Switzerland; [5]University of Cambridge, Cambridge, United Kingdom; [6]University of Florida, Florida, USA
*Trials* 2019, **20(Suppl 1):**PS3B

**Introduction:** Two-stage adaptive enrichment designs are efficient for trials in stratified medicine. In stage 1 patients are recruited from the full population while in stage 2, recruitment is restricted to a biomarker-driven subgroup that is selected based on the interim analysis of stage 1 data. Confirmatory analysis in the selected subgroup consists of stages 1 and 2 patients. The aim of this work was to develop appropriate point and interval estimators for survival outcomes which are common in oncology, where subgroup analysis is common. Such estimators do not exist.

**Methods:** One challenge in the work was to determine the asymptotic distribution for the log hazard ratio. We have proposed the asymptotic normal distribution of the score statistic as it aligns with the log-rank test. Another challenge was how to include stage 2 data from patients with censored observations in stage 1 in estimation without inducing correlation between stages 1 and 2. To achieve approximate independent increment structure, we have proposed fixing the number of events from each of the following, patients used in the interim analysis, patients with censored observations at the interim analysis and patients recruited in stage 2. This enabled borrowing the group sequential ideas that assume independent increment structure. After addressing the two challenges, we extended methods for normally distributed outcomes to derive an approximate asymptotic uniformly minimum variance conditional unbiased estimator (UMVCUE) and a new confidence interval for the log hazard ratio.
**Results:** Using simulations, the approximate UMVCUE has negligible bias and does not have substantially bigger root mean squared error than the naive estimator. Further, unlike the naïve confidence interval, the new confidence interval has the desired coverage probability.
**Discussion:** Naive estimators following two-stage adaptive enrichment clinical trials have undesirable properties. Therefore, we recommend the approximate UMVCUE and the new confidence interval.

## PS3B
### - O5 Trial design and management challenges for clinical trials of novel cell therapies: results from a mixed methods study

Ruchi Higham[1], Andrew Webster[2]
[1]Leeds Institute of Clinical Trials Research, University of Leeds, Leeds, United Kingdom; [2]Science and Technology Studies Unit, University of York, York, United Kingdom
*Trials* 2019, **20(Suppl 1):**PS3B

**Introduction:** Despite significant progress in scientific stem cell research there are still very few novel cell therapies licensed for clinical use. Clinical trials have been identified as one of the main translational challenges for cell therapies, and issues such as length of time to complete, under-funding and manufacturing challenges have been the subject of much discussion amongst policy-makers, regulators, clinicians, methodologists and commercial developers. Although there has to date been some engagement with the trials community this has tended to focus on specific clinical areas (e.g. oncology). This paper examines some of the challenges experienced in designing and conducting cell therapy trials in the UK with a view to identifying opportunities to help overcome these challenges.
**Methods:** Mixed methods study including a quantitative analysis of all current UK trials of novel cell therapies (n=44), 17 semi-structured interviews with clinicians, cell manufacturers and scientific researchers involved in current trials and an ethnographic case study of a musculoskeletal cell therapy trial.
**Results:** Three meta-themes were identified which appear to be particularly challenging for trials:
1)Uncertainty: difficult to design trial protocols and interpret results because basic science, mechanism of action and ideal treatment protocol often poorly understood.
2)Complexity: treatments involve a number of components (e.g. surgical procedures, manufacturing processes) which are highly context specific.
3)Variability: both between trials (e.g. clinical area, cell type) and within trials (e.g. site expertise and resources, heterogeneity in cell batches, effective dose, patient response).
**Discussion:** These findings suggest that cell therapies should be treated as complex interventions. The MRC Complex Interventions Evaluation Framework and the IDEAL framework for surgical trials offer useful guidance that could be adapted to address the particular challenges of cell therapies. Methodological opportunities to improve the efficiency of trials include adaptive and factorial designs, randomised registry trials and embedded process evaluations.

## PS3C
### - O1 Optimal curtailed designs for single arm phase II clinical trials

Martin Law[1], Michael Grayling[1,2], Adrian P Mander[1]
[1]Hubs for Trials Methodology Research, Medical Research Council Biostatistics Unit, University of Cambridge, Cambridge, United Kingdom; [2]Institute of Health and Society, Newcastle University, Newcastle, United Kingdom
*Trials* 2019, **20(Suppl 1):**PS3C

**Introduction:** Given the high cost and failure rate of single-arm phase II oncology trials, it is of interest to reduce the expected sample size, allowing efficacious treatments to be found more quickly and minimising the number of patients receiving an inefficacious treatment. Simon's design uses a single interim analysis to reduce expected sample size. However, further reductions can be achieved using curtailment. Curtailment is stopping when the final go or no-go decision is certain, so-called non-stochastic curtailment, or likely, known as stochastic curtailment.
We reduce the expected sample size by proposing designs that incorporate stochastic curtailment for either a go or no-go decision.
**Methods:** We obtain optimal stopping boundaries by searching over a range of potential conditional powers, beyond which the trial will stop for a go or no-go decision. This search is novel: firstly, the search is undertaken over a range of values unique to each possible design realisation. Secondly, these values are evaluated taking into account the possibility of early stopping. Finally, each design realisation's operating characteristics are obtained exactly.
The proposed designs are compared to existing designs in a real data example. They are also compared under three scenarios, both with respect to four single optimality criteria and using a loss function.
**Results:** Using the real data, the proposed designs approximately halve the sample size. Comparing designs across the three scenarios, the proposed designs are superior in almost all cases. Optimising for the expected sample size under either the null or alternative hypothesis, the saving compared to the popular Simon's design ranges from 22% to 55%.
**Discussion:** We have proposed designs for phase II, single-arm, binary outcome clinical trials, and augmented them with a number of approaches for finding better design realisations. These designs, combined with the proposed approaches, are shown to be superior to existing designs.

## PS3C
### - O2 Two-stage single-arm oncology trials: More adjusted analyses needed

Michael Grayling[1], Adrian Mander[2,3]
[1]Newcastle University, Newcastle upon Tyne, United Kingdom; [2]Cardiff University, Cardiff, United Kingdom; [3]MRC Biostatistics Unit, University of Cambridge, Cambridge, United Kingdom
*Trials* 2019, **20(Suppl 1):**PS3C

**Introduction:** Two-stage single-arm designs remain the most common design in phase II oncology. Consequently, much research has been conducted on how to analyse such trials. However, it is not clear that recommended methods are regularly utilised, and therefore many trials may report results that are biased or have inflated type-I error-rates. We present a review that quantifies the degree of this problem.
**Methods:** PubMed was used to identify phase II oncology trials conducted between 2013-2017. Each extracted record was assessed to identify trials that utilised a two-stage single-arm design with a binary primary outcome, for subsequent inclusion in the review. Included trials were evaluated against a variety of quality of reporting criteria. Wherever possible, the design parameters were matched against reported operating characteristics for each trial. Those trials that proceeded to stage two were also assessed on whether they (a)

performed an analysis that accounted for the interim analysis, and (b) accounted for any design deviation. For trials that did not conform to (a) or (b), the final analysis was replicated using recommended inferential procedures to assess quantities such as the bias in the final point estimate.
**Results:** Over 5000 records were extracted from PubMed, leading to more than 500 included trials. Key design parameters and resultant operating characteristics were well reported. However, few trials that proceeded to stage two adjusted their inference to account for the interim analysis. Similarly, despite design deviation being common, almost no studies accounted for this in their reported analyses.
**Conclusions:** Two-stage single-arm designs can efficiently weed-out inactive anti-cancer agents, but their value is reduced when inference is not performed in the recommended manner. Currently, few trials conform to established recommendations. Future two-stage single-arm trials should utilise adjusted analysis procedures, so that they conform to their desired type-I error-rate, and do not over-estimate response rates.

## PS3C
### - O3 BOP2: Bayesian Optimal Design for Phase II Clinical Trials with Binary, Co-primary and Other Complex Endpoints

Ying Yuan[1], Heng Zhou[2], Jack Lee[1]
[1]University Of Texas MD Anderson Cancer Center, Houston, United States; [2]Merck, United States
*Trials* 2019, **20(Suppl 1):**PS3C

The endpoints for immunotherapy and targeted therapy are often complicated, making conventional phase II trial designs or commonly used basket designs inefficient and disfunctional. We propose a flexible Bayesian optimal phase II (BOP2) design that is capable of handling simple (e.g., binary) and complicated (e.g., ordinal, nested and co-primary) endpoints under a unified framework. We use a Dirichlet-multinomial model to accommodate different types of endpoints. At each interim, the go/no-go decision is made by evaluating a set of posterior probabilities of the events of interest, which is optimized to maximize power or minimize the number of patients under the null hypothesis. Unlike most existing Bayesian designs, the BOP2 design explicitly controls the type I error rate, thereby bridging the gap between Bayesian designs and frequentist designs. In addition, the stopping boundary of the BOP2 design can be enumerated prior to the onset of the trial. These features make the BOP2 design accessible to a wide range of users and regulatory agencies, and particularly easy to implement in practice. Simulation studies show that the BOP2 design has favorable operating characteristics with higher power and lower risk of incorrectly terminating the trial than some existing Bayesian phase II designs. The software to implement the BOP2 design is freely available at www.trialdesign.org

## PS3C
### - O4 How to use a margin of practical equivalence to include considerations other than efficacy in randomised selection trials

Hakim-Moulay Dehbi[1], Allan Hackshaw[2]
[1]Comprehensive Clinical Trials Unit at UCL, London, United Kingdom; [2]CRUK & UCL Cancer Trials Centre, London, United Kingdom
*Trials* 2019, **20(Suppl 1):**PS3C

**Introduction:** In rare cancers or subtypes of common cancer a comparison of multiple promising treatments may be required. The selected treatment can then be assessed against the standard of care (if it exists) or used as backbone for combinations with new, possibly targeted, agents. There could be different experimental therapies or different doses of the same therapy, and either done in combination with standard treatments.
In practice a “pick-the-winner” design is often used, focusing only on efficacy to select the most promising treatment. Recent selection trials have modified the pick-the-winner rule in an ad-hoc manner to make decisions in case of equal/similar observed responses.
There is a need to formalise how a treatment with a slightly lower efficacy compared to another treatment may actually be preferred if it has a better toxicity profile, is easier to administer, or cheaper.
**Methods:** We define at the design stage a margin of practical equivalence, which corresponds to the extent by which a superior treatment needs to be to be selected based on efficacy only.
Considering binary outcomes, we used exact probabilities to calculate the required sample size for two- and three-arm selection trials incorporating a margin for a large variety of design inputs. The sample size is obtained by making sure that the probability of selecting the superior treatment, assuming it exists among the options considered, is above a threshold, often 80% or more.
**Results:** We developed a free user-friendly web application for sample size calculation. The application also derives the probability of selecting the superior treatment for a given sample size.
**Discussion:** There is a need to improve the design of selection trials. The application will facilitate the adoption of the margin of practical equivalence, which formalises the flexibility required compared to the strict pick-the-winner rule.

## PS3C
### - O5 The critical and recommended characteristics for the reporting of treatment-as-usual in behaviour change trials

Neza Javornik[1], Daniel Powell[1], Marion Campbell[1], Marijn De Bruin[1,2]
[1]University Of Aberdeen, Aberdeen, United Kingdom; [2]Radboud University Medical Center, Nijmegen, Netherlands
*Trials* 2019, **20(Suppl 1):**PS3C

**Introduction:** Treatment-as-usual (TAU) is often used as a comparator in behaviour change trials. Its characteristics are poorly described in published manuscripts but are highly heterogeneous and can influence trial outcomes. Guidelines for reporting of behavioural trials offer little guidance on which elements of TAU should be reported. This study identifies which TAU characteristics are critical in behaviour change trials and should be routinely reported.
**Method:** A three-round modified Delphi study was conducted with an international panel (N = 25) of health psychology practitioners and research experts in trials methodology, primary care, TAU and behavioural interventions. Participants rated the importance of 60 possible items, organised under eight categories (the reporting of health behaviours, active content, tailoring of active content, setting, provider, treatment time, fidelity of delivery and therapeutic alliance) in Rounds 1 and 3 on a 9-point scale (1-3: not important; 4-6: recommended; 7-9: critical). Participants also rated how detailed the reporting should be. Round 2 was a 2-week online discussion forum amongst participants to discuss items. Consensus in Round 3 was established using pre-determined thresholds.
**Results:** Experts agreed that 19 TAU characteristics (organised under health behaviours, active content and tailoring, provider’s profession, treatment time, setting and fidelity of delivery) are critical to report at a general level. All these characteristics were also recommended to be reported in more detail.
**Discussion:** This study adds to the existing reporting guidelines by specifying which individual TAU characteristics are critical when reporting behaviour change trials with TAU comparators, and how detailed their reporting should be. Further research is needed to assess how feasible it would be to collect these data.

## PS3D
### - O1 Framework for approaches to address statistical multiplicity in pragmatic RCTs through systematic review and surveys of statistical practice

Katie Pike, Barnaby C Reeves, Chris A Rogers
Clinical Trials and Evaluation Unit, Bristol Trials Centre, University of Bristol, Bristol, United Kingdom
*Trials* 2019, **20(Suppl 1):**PS3D

**Introduction:** We are seeking to determine: a) optimal approaches to address statistical multiplicity in pragmatic RCTs, and b) whether approaches should vary according to the taxonomy of the situation and context of research questions. The first step is to collate information from three sources:
a)Systematic literature review around opinions, guidelines and methods used
b)Survey of practice within UK based Clinical Trial Units (CTUs)
c)Survey of the methods used in pragmatic RCTs recently published in high impact medical journals
**Methods:** A key objective is the creation of a conceptual framework to synthesise the information obtained from the three sources. We are extracting data from relevant papers into nVivo software in order to generate themes and visualise findings graphically, e.g. comparison diagrams and concept maps. These findings will also be used to inform the design of both surveys, which will include factual questions about specific approaches and open-ended questions to capture alternative methods.
**Provisional results:** Provisional systematic review findings suggest varying opinions/guidelines on the approach to multiplicity, and that methods used range from simple Bonferroni adjustment through to more complicated procedures, e.g. hierarchical/gatekeeping procedures. Once results from all three sources are available the synthesised findings will be triangulated to inform the rest of the research; the first step of which is planned to be empirical studies comparing probabilities of Type I and II errors when applying different methods to different multiplicity situations.
**Potential relevance and impact:** This research will provide guidance on recommended approaches to address multiplicity for methodologists designing pragmatic RCTs and health professionals interpreting RCT findings. The overall results have potential to be applied to most RCTs commissioned by the NIHR, hence influencing evidence-based changes in practice in the UK NHS across many disease areas.
This research is funded by the NIHR and the NIHR disclaimer applies.

## PS3D
### - O2 Assessing the impact of early stopping on systematic reviews: Recommendations for interpreting guidelines

Ian Marschner[1,2], Lisa Askie[1,2], Manjula Schou[3]
[1]NHMRC Clinical Trials Centre, Sydney, Australia; [2]University of Sydney, Sydney, Australia; [3]Macquarie University, Sydney, Australia
*Trials* 2019, **20(Suppl 1):**PS3D

**Introduction:** The CONSORT Statement says that early stopping of a clinical trial weakens the conclusions from the trial. The GRADE guidelines go further, saying that early stopping is a study limitation carrying the risk of bias, and recommending systematic reviewers conduct sensitivity analyses omitting such studies. Our aim is to assess methodologies for conducting these sensitivity analyses and to make recommendations about interpretation of the guidelines.
**Methods:** We began by reviewing and summarising the range of possible impacts of early stopping on treatment effect estimation in single studies and meta-analyses. We then used simulation studies to evaluate the performance of various approaches to meta-analysis when early stopping is present. Our primary focus was early stopping for benefit and we investigated the performance of meta-analyses where treatment effect estimates were adjusted for the interim monitoring using the statistical method of conditional maximum likelihood estimation.
**Results:** Early stopping at the first interim analysis leads to overestimation of treatment effects but at subsequent interim analyses the effect is unpredictable, and may lead to overestimation, underestimation or even unbiased estimation. Interim monitoring that does not stop the study early leads to underestimation. Importantly, these effects do not translate into biased meta-analyses, because the underestimation and overestimation balance each other in multiple studies. In contrast, exclusion of studies stopped early always leads to underestimation in meta-analyses. However, if treatment effect estimates are adjusted for the interim monitoring, prior to conducting the meta-analysis, the underestimation is rectified.
**Discussion:** We recommend against sensitivity analyses that simply exclude studies that stopped early from meta-analyses. If a sensitivity analysis is conducted in accordance with GRADE, we recommend that treatment effect estimates are first adjusted for the interim monitoring. To facilitate this, we recommend specific information that should be reported in adhering to the CONSORT reporting standards on early stopping.

## PS3D
### - O3 Reporting of methodological aspects of randomised trials: 1996-2016; has it changed over time?

Shona Fielding, Ruiyi Long, Neil Scott
University Of Aberdeen, Aberdeen, United Kingdom
*Trials* 2019, **20(Suppl 1):**PS3D

**Introduction:** The Consolidated Standards of Reporting Trials (CONSORT) statement was introduced in 1996 and revised in 2010. It provides a checklist for reporting details of the study design/results of randomised controlled trials (RCTs). We aimed to survey RCTs published in 1996, 2006 and 2016 to determine whether reporting standards of key methodological details had changed over time.
**Method:** We used MEDLINE to obtain a random sample of published RCT articles from each year. There was no restriction on journal. Titles and abstracts were screened, and full text of potentially eligible studies assessed for inclusion. We collected information on the journal, number of treatment arms, whether a CONSORT flow diagram and power calculation were included and how randomisation methods and baseline data were reported.
**Result:** 252/603 studies were considered eligible for inclusion. Just over a third provided a CONSORT flow diagram. A table of baseline characteristics was provided in 83% of studies, but over half of these also provided p-values for baseline data. Details of a power calculation were included for 47% of studies. The method used to generate the random sequence and method of allocation concealment was unclear/not reported in 70% and 65% of studies respectively. Over time, the proportions including CONSORT diagrams, power calculations and baseline tables increased. Reporting of randomisation methods also improved; permuted blocks and sealed envelopes remained the most common methods for sequence generation and concealment.
**Discussion:** Our survey was not restricted to high impact journals and showed that reporting standards have improved between 1996 and 2016, but despite the existence of CONSORT, there is still poor reporting of key methodological aspects of RCTs. Notably, over half of articles reported significance tests for baseline characteristics, contrary to current recommendations, and the method of randomisation was either unclear or not reported in over half of the studies.

## PS3D
### - O4 How well are binary outcomes analysed and the findings reported? – A systematic review of randomised trials

Ines Rombach[1,2,3], Nicholas Peckham[1,2,3], Ruth Knight[1,2,3], Jamie Stokes[1,2,3], Jonathan A Cook[1,2,3]
[1]Oxford Clinical Trials Unit, Oxford, UK; [2]Centre for Statistics in Medicine, Oxford, UK; [3]Nuffield Department of Orthopaedics, Rheumatology and Musculoskeletal Sciences, Oxford, UK
*Trials* 2019, **20(Suppl 1):**PS3D

**Introduction:** Randomised clinical trials (RCTs) need to be reported in a way that enables robust and reliable interpretation of their results. Here we review how well current publications of RCTs report binary primary outcomes and appropriate sensitivity analysis for missing data.
Numerous different statistical approaches exist for the analysis of binary data, some of which have been shown to be suboptimal. Guidelines, including the Consolidated Standards of Reporting Trials (CONSORT), stipulate that analyses of binary outcomes should present estimates for both absolute and relative effects, together with confidence intervals. Where some primary outcome data are missing, sensitivity analysis should investigate the effect of changes to assumed missing data mechanism on the trial conclusions. It is uncertain what is typically done.
**Methods:** We searched MEDLINE for primary publications of RCTs published in January 2019, and identified those that reported a binary primary endpoint. Data to be extracted include the study size, loss to follow-up by trial arm, statistical analysis methods (principal and secondary) for the primary endpoint, together with whether or not absolute as well as relative effects and their confidence intervals were reported. Details of sensitivity analyses performed will be extracted, and reporting of the results will be considered
**Timing of Potential Results:** The search and study assessment process is in progress and data extraction and synthesis will take place over the summer. Around 200 trials are anticipated to be included.
**Potential Relevance & Impact:** Current practice on how RCTs with binary primary endpoints are analysed, and how well the results are presented will be summarised. Presentation of both relative and absolute effects, as well as the use of appropriate sensitivity analysis are assessed. This will be useful to both authors and reviewers of RCT reports and contribute to improving reporting standards, as well as identifying potential areas for improvement.

## PS3D
### - O5 Overestimation of Event Rate and Target Difference among Randomized Clinical in sample size calculations Trials: a cross-sectional survey review

Tao Chen[1], Chao Li[2], Yang Wang[3], James Dodd[1], Victoria Cornelius[4], Duolao Wang[1]
[1]Liverpool School Of Tropical Medicine, Liverpool, United Kingdom; [2]Xi'an Jiaotong University Health Science Centre, Xi'an, China; [3]Peking Union Medical College and Chinese Academy of Medical Sciences, Beijing , China; [4]Imperial College London, LONDON, United Kingdom
*Trials* 2019, **20(Suppl 1):**PS3D

**Background:** When designing a randomized clinical trial (RCT), unlike power and alpha which can be set at conventionally accepted values (usually 90% or 5%, respectively), assumptions about the event rate of the primary endpoint and the targeted difference (or minimal clinically important difference) for the sample size calculation are often based on prior knowledge from empirical studies or expert opinion. Overestimation of event rates and target difference can have a detrimental impact on the power of an RCT. In this study, we aim to systematically investigate the prevalence and extent of overestimation of event rates and targeted difference. We will then examine their impact on trial conclusions.
**Methods:** We are planning to perform a cross-sectional survey review of phase II-III RCTs published in seven high impact medical journals from January 2015 to January 2019.
**Timing of Potential Results:** This study is part of an ongoing clinical trials survey. It will include approximately 200 two-arm, parallel and superiority trials with a single binary outcome. In a pilot review of the first 68 RCTs, we have found that 34 (50.0%) had an overestimated primary event rate (i.e. smaller than the lower limit of the 95% CI of observed event rate) and 31 (45.6%) had a larger than targeted difference (i.e. outside the 95% CI of the actual treatment difference). However, among those 41 studies either having overestimation of event rate or target difference, 10 of them drew a positive conclusion.
**Potential Relevance & Impact:** We think that many trials may be underpowered due to higher event rates or aiming to detect unrealistic treatment effects. If this is the case the results from these studies should be interpreted with caution and not in isolation. This is particularly important for trials with positive conclusion.

## PS4A
### - O1 Internal pilots in clinical trials: Current practice in design and assessment

Anna Rosala-Hallas[1], Carrol Gamble[1], Jane Blazeby[2], Paula R Williamson[3]
[1]Clinical Trials Research Centre, University of Liverpool, a member of the Liverpool Health Partners, Liverpool, United Kingdom; [2]Medical Research Council ConDuCT II Hub for Trials Methodology Research, NIHR Bristol Biomedical Research Centres, Population Health Sciences, Bristol Medical School, University of Bristol, Bristol, United Kingdom; [3]North West Hub for Trials Methodology Research/Clinical Trials Research Centre, University of Liverpool, a member of the Liverpool Health Partners, Liverpool, United Kingdom
*Trials* 2019, **20(Suppl 1):**PS4A

**Introduction:** Running an internal pilot can help to optimise methods for a trial. The literature provides recommendations for the design of internal pilots, however, information is lacking regarding the designs used and the process of review against progression criteria. The aim of this research is to provide an overview of current practice.
**Methods:** A cohort of clinical trials, extracted in 2017, comprised those with an internal pilot having undergone the progression review stage, funded by the National Institute for Health Research Health Technology Assessment programme. Data about the design and assessment of the internal pilots were abstracted from: project descriptions; summary of changes from the first stage application; funder/reviewer feedback about the full application; funder monitoring notes; progress report history and trial protocols.
**Results:** Fifty-seven studies were reviewed. The majority of internal pilots were first proposed in the initial stages. The Trial Steering Committee was mostly commonly involved in the review process, alongside the funder. Progression criteria included: target number for recruitment, rate of randomisation, retention/primary outcome ascertainment rate, rate of treatment adherence and consent rate. All studies but one continued to the main trial, however a quarter did not strictly meet their progression criteria. Actions taken for studies which did not meet their progression criteria included a second review, recovery plan and close monitoring. Changes were made to the design of the main trial for 25% of the studies; these were primarily to do with conduct of recruitment.
**Discussion:** We provide insight into the process of designing and assessing internal pilot trials. Progression criteria are sometimes not met; however, committees involved in the reviewing process will generally support continuation to the main trial, usually accompanied by a second review or close monitoring. We make recommendations to optimise the decision-making process.

## PS4A
### - O2 External Pilot and Feasibility Studies: Past, Present and Future Challenges

Lehana THABANE[1], Sandra Eldridge[2], Christine Bond[3], Claire Chan[2], Gillian Lancaster[4], Michael Campbell[5], Sally Hopewell[6]
[1]McMaster University, Hamilton, Canada; [2]Queen Mary University of London, London, UK; [3]University of Aberdeen, Aberdeen, UK; [4]Keele University, Keele, UK; [5]University of Sheffield, Sheffield, UK; [6]University of Oxford, Oxford, UK
*Trials* 2019, **20(Suppl 1):**PS4A

External pilot and feasibility (PAF) trials are an essential part of trial preparation, particularly for the planning of complex interventions. However, they rarely published, and the ones that are published tend show a lack of clarity in the objectives and methodological focus. There are also misunderstandings about the purpose of PAF trials including confusion about the definitions of the terms "pilot trial" and "feasibility trial". In this presentation, we discuss: 1) some key challenges with past practice in the conduct and reporting of external PAF trials; 2) progress on addressing some of the challenges that include providing the framework for defining PAF studies; and the CONSORT extension to PAF trials focusing on those conducted in preparation for a randomised controlled trial (RCT); and 3) future plans to address important, unresolved uncertainties in design, conduct, analysis and reporting of external pilot and feasibility studies. These will include developing guidelines for sample size estimation, writing study protocols for external PAF trials, and guidance for reporting of external PAF studies which are not randomized controlled trials.

## PS4A

### - O3 Assessing differences in start-up between a pilot and main RCT in the ICU: The CYCLE international multicentre rehabilitation study

Michelle E Kho[1,2], Ian M Ball[3], Sue Berney[6], Karen EA Burns[4], Deborah J Cook[1,2], Frederick D'Aragon[5], Erick Duan[7], Shane English[8], Alison Fox-Robichaud[1], Margaret S Herridge[9], Tim Karachi[1], Sangeeta Mehta[9], Alexander Molloy[2], Amy Pastva[10], Laurel Patterson[2], Stacey Priest[1], Brenda Reeve[11], Julie C Reid[1], Bram Rochwerg[1], Andrew JE Seely[8], Jennifer Tsang[7], Avelino Verceles[12]
[1]McMaster University, Hamilton, Canada; [2]St. Joseph's Healthcare, Hamilton, Canada; [3]Western University, London , Canada; [4]St. Michael's Hospital, Toronto, Canada; [5]Universite de Sherbrooke, Sherbrooke, Canada; [6]University of Melbourne, Melbourne, Australia; [7]Niagara Health, St. Catharines, Canada; [8]University of Ottawa, Ottawa, Canada; [9]University of Toronto, Toronto, Canada; [10]Duke University, Durham, USA; [11]Brantford General Hospital, Brantford, Canada; [12]University of Maryland, Baltimore, USA
*Trials* 2019, **20(Suppl 1):**PS4A

**Introduction:** Pilot randomized clinical trials (RCTs) inform the design, planning, and conduct of full-scale RCTs. We reviewed the start-up activities of an ongoing international rehabilitation RCT (NCT03471247) examining early in-bed cycling in critically ill patients comparing the pilot with the main RCT.
**Methods:** We included all sites that participated in the pilot (CYCLE Pilot RCT, CYCLE Vanguard) and those in the main RCT which initiated ethics, contracts, or personnel training as of April 30, 2019. We extracted time to ethics and contracts approval, number of training sessions and personnel trained on the protocol, and time from last activity (approvals or training) to the date of first patient enrolment.
We hypothesized: 1.Shorter time to ethics approval for the full RCT. 2.No difference in time to contract approval. 3.No difference in time to first patient enrolment. We calculated mean and standard deviation (SD) or median and interquartile ranges [Q1, Q3] for data that were not normally distributed. We conducted an unpaired t-test or Mann Whitney-U Test, as appropriate. We used $p<0.05$ for statistical significance.
**Results:** We included 10 pilot (8 Canada, 1 US, 1 Australia) and 14 full RCT sites (11 Canada, 2 US, 1 Australia). Of these, 9 sites (7 Canada, 1 US, 1 Australia) participated in both the pilot and full RCT. There was a shorter time to ethics approval in the full RCT (18 vs 68 days,$p=0.007$), but no differences in time to contracts approval (64 vs 81 days,$p=0.805$) or first patient enrolment (39 vs 26 days,$p=0.389$) (Table 1).
**Discussion:** Experiences and knowledge gained from the CYCLE pilot RCT were instrumental in informing and planning start-up activities of our main RCT that led to faster ethics approval. Future studies examining the generalizability of our results to other rehabilitation or critical care trials are needed.

## PS4A

### - O4 Distinctive ethical aspects of consent in pilot and feasibility studies

Julius Sim[1,2]
[1]School of Primary, Community and Social Care, Keele University, United Kingdom; [2]Keele Clinical Trials Unit, Keele University, United Kingdom
*Trials* 2019, **20(Suppl 1):**PS4A

**Introduction:** Although ethical issues related to consent in an external pilot study (PS) or a feasibility study (FS) resemble those in a definitive randomized controlled trial (RCT), there are important differences in emphasis.
**Analysis:** Consent is given, or withheld, based on an understanding of the study's nature and purpose, and an appraisal of its potential benefit; participants may altruistically shoulder the burden of participation for the sake of generating knowledge. Whilst the direct benefit of a full RCT relates to clinical practice, the direct benefit of a PS or FS relates to whether or not to conduct a full RCT; clinical benefit is indirect, and uncertain, given that a full RCT may not ultimately occur. As a randomized PS may outwardly resemble a main RCT, participants may mistake its true purpose. Thus, the intended and the perceived value of the study may differ, creating a different variety of what is known as 'therapeutic misconception' from that arising in a definitive RCT. The quality of consent will diminish accordingly.
Additionally, consent may be undermined by participants' tendency to overestimate the benefits, and underestimate the risks, of taking part (referred to as 'therapeutic misestimation'). This tendency may raise specific concerns in a PS or FS: i) the nature and/or intensity of an intervention being refined in a FS will not yet be fully optimized; ii) participants in a randomized PF may not benefit from the superior treatment until the completion of the main RCT very much later; iii) if a subsequent main RCT is not in fact undertaken, no such clinical benefit will accrue.
**Conclusion:** Ethical issues associated with consent in a PS or FS are similar to those in a main RCT but require a different focus. This should be reflected in the consent process and in the associated consent documentation.

## PS4A

### - O5 Determining sample size for progression criteria using hypothesis testing in pragmatic pilot RCTs

Martyn Lewis[1], Kieran Bromley[1], Gareth McCray[1], Christopher Sutton[2], Gillian Lancaster[1]
[1]School of Primary, Community & Social Care, Keele University, United Kingdom; [2]Health Services Research & Primary Care, University of Manchester, United Kingdom
*Trials* 2019, **20(Suppl 1):**PS4A

**Introduction:** The current CONSORT guidelines for reporting of pilot trials do not recommend hypothesis testing of clinical outcomes on the basis of a pilot trial being under-powered to detect such differences and this being the aim of the main trial. It states that primary evaluation should focus on descriptive analysis of feasibility or process outcomes (e.g. recruitment, adherence, treatment fidelity). Whilst the argument for not testing clinical outcomes is justifiable, the same does not necessarily apply to feasibility/process outcomes, where differences may be large and detectable with small sample sizes. Moreover, there remains much ambiguity around sample size for pilot trials.

**Methods:** Many pilot trials adopt a 'traffic light' system for evaluating progression to the main trial determined by a set of criteria set up a priori. We can set up a hypothesis-testing approach focused around this system that tests against being in the RED zone (unacceptable outcome) based on an expectation of being in the GREEN zone (acceptable outcome).
Results. For example, in relation to treatment fidelity, if we assume the upper boundary of the RED zone is 40% and the lower bound of the GREEN zone is 70% (designating unacceptable/acceptable treatment fidelity, respectively), the sample size required for analysis given 90% power and one-sided 5% alpha would be n=22 (intervention group alone). A larger sample size would increase the power to reject the RED signal in favour of potential progression.
**Discussion:** In general, more than one key process outcomes are assessed for progression to a main trial; a composite approach would be to appraise the rules of progression across all these key outcomes. This methodology begins to provide a formal framework for hypothesis-testing and sample size indication around process outcome evaluation for pilot RCTs.

## PS4B
### - O1 Considerations concerning the use of health economics in the design and analysis of adaptive clinical trials – a qualitative study

Laura Flight[1], Daniel Hind[1], Steven Julious[1], Alan Brennan[1], Susan Todd[2]
[1]University of Sheffield, United Kingdom; [2]University of Reading, United Kingdom
*Trials* 2019, **20(Suppl 1):**PS4B

**Introduction:** Both adaptive designs and health economics offer innovative approaches to efficient research and decision making. Adaptive designs allow data to be examined as a trial progresses to inform changes to that trial. The methods of health economics aim to maximise the health gained for the money spent. Methods for evaluating cost-effectiveness could be incorporated into the design and analysis of adaptive clinical trials, to give innovative and efficient trials.
**Methods:** A qualitative study explored the attitudes of key stakeholders – including researchers, decision makers and members of the public - towards the use of health economics in the design, monitoring and analysis of adaptive clinical trials. Data were collected using interviews and focus groups. Framework analysis was used to analyse the data and identify themes.
**Results:** Twenty-nine participants took part in the study. Participants considered that answering the clinical research question should be the priority in a clinical trial, despite their awareness that cost-effectiveness was important for decision making. Concerns raised by participants included: handling the volatile nature of cost data at interim cost-effectiveness analyses; implementing this approach in global trials; resourcing the development of adaptive trial designs that use health economics in their design and analysis and training stakeholders in these methods so that they can be implemented by researchers and appropriately interpreted by decision makers.
**Conclusion:** The use of health economics in the design and analysis of adaptive clinical trials has the potential to increase the efficiency of health technology assessments worldwide. However, careful consideration is needed to ensure the statistical methods reflect the importance of clinical effectiveness and adequate training and resources are provided to facilitate the implementation of this approach.

## PS4B
### - O2 Stopping a clinical trial early based on the probability that cost-effectiveness is unlikely: An extension of conditional power computations to economic evaluation iftekhar Khan
University of Oxford, Oxford, United Kingdom
*Trials* 2019, **20(Suppl 1):**PS4B

**Introduction:** Conditional power (CP) is a well-established method for conducting futility analyses in clinical trials to stop a trial early for lack of efficacy using observed data at some interim point. However, despite futility criteria not being met, a trial may continue with modest efficacy gains that are unlikely to be cost-effective. Treatment effects from such trials may not offer value for money to tax payers because the cost per quality adjusted life year (QALY) can be much higher than acceptable thresholds. Stopping a trial based on the joint criteria of efficacy and costs have not been examined in detail.
**Objectives:** We extend the conditional power computations and examine the probability of cost-effectiveness conditional on the data observed at some interim point when applied in practice.
**Methods:** Expressions for the conditional power based on efficacy for the two sample case (1:1 allocation) are extended to cost-effectiveness. The incremental net benefit (INB), willingness to pay (WTP) threshold, interim and final sample sizes, variabilities and correlations between costs and effects are examined. Data from several clinical trials are used to examine the operating characteristics of the conditional power of cost-effectiveness (CPCE).
**Results (TBC):** Both CP and CPCE agree in general, about 70% of the time for a CP threshold of 80%. CPCE conclusions will differ and can be much lower 30% of the time, depending on the WTP. Hence, trials may continue based on clinical efficacy, despite interventions being unlikely to be cost-effective. Value of information methods (VOI) and Bayesian predictive power results provide consistent conclusions with CPCE.
Conclusions: CP based on cost-effectiveness is feasible and a useful tool during IDMC meetings (where health economists are increasingly involved) for decision making, especially where clinical effects are modest or small. CPCE uses more information than standard CP methods and is more informative.

## PS4B
### - O3 Cost-Effective Clinical Trial Design: Application of a Bayesian Sequential Stopping Rule to the ProfHER Pragmatic Trial

Martin Forster[1], Stephen Brealey[1], Stephen Chick[2], Ada Keding[1], Belen Corbacho[1], Andres Alban[2], Amar Rangan[1,3,4]
[1]University Of York, York, United Kingdom; [2]INSEAD, Fontainebleau, France; [3]University of Oxford, Oxford, United Kingdom; [4]James Cook University Hospital, Middlesbrough, United Kingdon
*Trials* 2019, **20(Suppl 1):**PS4B

**Introduction:** We investigate value-based, sequential, clinical trial design by applying a Bayesian decision-theoretic model of a sequential experiment to data from the ProfHER pragmatic trial. The work represents the first applied analysis of this value-based, sequential, design to retrospective data from a completed clinical trial.
**Methods:** We take information on the research cost profile from the trial and incorporate information on the accumulation of evidence on cost-effectiveness to obtain a stopping boundary. We compare this with the accumulation of data in the trial itself and use a bootstrap analysis to study the stopping rule's operating characteristics.
**Results:** We show that the model's stopping policy would have stopped the trial early, saving about 5% of the research budget

(approximately £73,000). The bootstrap analysis suggests that the trial's expected sample size could have been reduced by approximately 40%, saving an expected 15% of the budget, with 93% of resampled paths making a decision consistent with the result of the trial itself. Hence we show how substantial benefits to trial cost stewardship may be achieved by accounting for research costs in defining the trial's stopping policy and active monitoring of trial data as it accumulates.

## PS4B
### - O4 Dealing with unavoidably high loss to follow-up in care home trials - The DCM-EPIC trial

Rebecca Walwyn[1], Amanda Farrin[1], Claire Surr[2]
[1]University Of Leeds, Leeds, United Kingdom; [2]Leeds Beckett University, Leeds, United Kingdom
*Trials* 2019, **20(Suppl 1):**PS4B

**Introduction:** Conducting cluster-randomised controlled trials (cRCTs) in care homes is challenging and resource intensive. Significant but unavoidable loss to follow-up is to be expected due to the frailty and age of the population. High loss to follow-up can lead to an underpowered trial, biased results and uncertain conclusions due to missing data.
**Methods:** DCM-EPIC was a cRCT evaluating the effectiveness and cost-effectiveness of staff-implemented Dementia Care Mapping (an observational tool to support the implementation of person-centred care) for people with dementia living in care homes in England. The primary outcome was the level of agitation in residents 16-months following care home randomisation.
**Results:** In DCM-EPIC, we faced ~50% at 16-months in clusters whose size is fixed by the care home size/beds.
**Discussion:** Typically there are two trial design options: a closed-cohort or a cross-sectional cRCT. DCM-EPIC was originally designed as a closed-cohort cRCT but the loss to follow-up meant a design change was required to maintain statistical power. A theoretically attractive alternative that addresses the high loss to follow-up, termed an 'open-cohort' or 'dynamic-cohort' cRCT design, can be considered a hybrid between closed-cohort and cross-sectional designs, but is presently not a widely recognised choice for cRCTs. Using DCM-EPIC as an example, we will describe the rationale for using an open-cohort design in this setting, the possible implications for the scientific elements of the research (sample size, bias and analysis) as well as study implementation elements (additional researcher resource to recruit further residents, data collection and management considerations), when compared to traditional alternatives.

## PS4B
### - O5 To fund or not to fund a paediatric severe asthma trial: that is the question

Daphne Babalis[1], Sejal Saglani[2], Victoria Cornelius[1]
[1]Imperial Clinical Trials Unit, Imperial College London, London, United Kingdom; [2]National Heart and Lung Institute, Imperial College London, London, United Kingdom
*Trials* 2019, **20(Suppl 1):**PS4B

**Introduction:** Clinical Trial Unit teams (CTUs) work closely with investigators to develop funding applications for trials. The last 5 years have seen an increase in the detail and reassurances funders request prior to confirming funding success. When novel or adaptive designs are proposed these iterations and reassurances can be even harder to navigate. We share our experience and learning on a three year journey from first submission to award of a small population paediatric trial.
**Methods:** In 2016 the NIHR released a commissioned call for "Difficult to Treat Severe Asthma" encouraging novel approaches to make a step-change in treatment. A national collaboration of investigators identified priority research questions for the subset of children with severe treatment resistant asthma.
Feasibility work confirmed a maximum achievable sample size of 150 over 3-years and PPI work established a placebo arm would only be acceptable for a short period. In order to answer three research questions and achieve the efficiency required, a non-standard design of two interlinking trials was selected using a continuous primary outcome, within-participant variability, and inclusion of placebo arm to allow efficacy assessment and bio-marker validation.
The design presented challenges in terms of achieving blinding due to complexity of the treatments and it was considered too complex by the funder.
**Results:** With funder engagement the applicants re-examined the research questions and undertook a radical re-design leading to an open-label non-inferiority comparison of two treatments, using a Bayesian approach with clinician elicited prior. Operational complexity was reduced by the removal of blinding but Industry were less keen to support and cost increased due to longer duration.
**Discussion:** Effective collaboration between Investigators, CTU staff, PPI experts and funder is essential to ensure the right questions are addressed and trial design is acceptable in small population settings.

## PS4C
### - O1
**Abstract omitted**

## PS4C
### - O2 The challenges of delivering a time-critical intervention in emergency care

Helen Thomas[1], Claire Foley[1], Joanne Lucas[1], Amy Evans[1], Ross Davenport[2,3], Nicola Curry[4,5], Simon Stanworth[5,6,7,8], Karim Brohi[2,3], on behalf of the CRYOSTAT-2 investigators
[1]NHS Blood and Transplant Clinical Trials Unit, Cambridge and Bristol, United Kingdom; [2]Centre for Trauma Sciences, Blizard Institute, London, United Kingdom; [3]Queen Mary, University of London, London, United Kingdom; [4]Oxford Haemophilia and Thrombosis Centre, Oxford University Hospitals NHS Trust, Oxford, United Kingdom; [5]Oxford NIHR BRC Haematology Theme, Oxford University, Oxford, United Kingdom; [6]Transfusion Medicine, NHS Blood and Transplant, Oxford, United Kingdom; [7]Department of Haematology, Oxford University Hospitals NHS Trust, Oxford, United Kingdom; [8]Radcliffe Department of Medicine, University of Oxford, Oxford, United Kingdom
*Trials* 2019, **20(Suppl 1):**PS4C

**Introduction:** CRYOSTAT-2 is an on-going Phase III trial recruiting 1568 trauma patients with major haemorrhage from 23 major trauma centres in the UK and planned recruitment at level 1 US trauma units. The intervention is early infusion of cryoprecipitate (a blood component), alongside the standard major haemorrhage protocol, which must be started within 90 minutes of arrival at the Emergency Department (ED).
**Methods:** Learning from two feasibility trials it was clear that the trial needed to be pragmatic to maximise recruitment in a challenging and time critical environment. There were several challenges to overcome including participant consent, randomisation method, timely preparation and delivery of cryoprecipitate from blood banks to ED, and comprehensive follow-up, as patients may abscond from hospital, be returned to custody or suffer ongoing emotional trauma. Recruitment out of hours was also considered crucial to recruit representative patients and meet recruitment targets.
**Results:** A waiver of consent process enables rapid recruitment when patients are often incapacitated, followed by personal and/or professional consultees and participant informed consent for continuation in the study. Randomisation uses simple physical envelopes, and is conducted by blood bank at some sites to reduce the burden on the ED team. The trial is pragmatic to enable recruitment out of hours and 29% of participants have been recruited between 8pm and 8am so far. In those who receive the intervention, 83% receive it within 90 minutes, up from 79% in the first six months of the study. Section 251 approval is being sought from the Confidentiality Advisory Group to enable complete follow-up.

**Table 1 (abstract PS4A - O3).** See text for description.

| | Pilot RCT N=10 sites | Main RCT N=14 sites | p-value |
|---|---|---|---|
| Time to ethics approval, median [Q1, Q3] (days) | 68 [30, 106] | 18 [14, 36][a] | 0.007 |
| Ontario sites participating in pilot and main RCT (n=7)* | 69 [28, 113] | 16 [13, 18] | 0.018 |
| Time to contracts approval, median [Q1, Q3] (days) | 81 [42, 119] | 64 [38, 141][b] | 0.805 |
| Time to first enrollment, mean ± SD (days) | 26±31 | 39±25[c] | 0.389 |
| Training sessions across all sites, n | | | |
| Cycling | 14 | 15[d] | - |
| Outcome measures | 12 | 13[e] | - |
| Research coordinator | 12 | 13[f] | - |
| Personnel trained | | | |
| Cycling, n | 52 | 44[d] | - |
| Cycling, median [Q1, Q3] (people per site) | 6 [3, 6] | 3 [2,4][d] | - |
| Outcome measures, n | 83 | 74[e] | - |
| Outcome measures, median [Q1, Q3] (people per site) | 10 [6, 10] | 6 [4, 7][e] | - |
| Research coordinator, n | 32 | 28[f] | - |
| Research coordinator, median [Q1, Q3] (people per site) | 2 [2, 3] | 2 [1,3][f] | - |

Abbreviations: RCT: Randomized clinical trial; Q=quartile; SD = standard deviation. *In the Main RCT, Ontario instituted a central REB. Sample sizes for Full RCT sites varied, based on completed activities as of April 30, 2019: [a]Time to REB approval: n=12; [b]Contracts approval: n=11; [c]First enrollment n=8; [d]Cycling: n=13 sites; [e]Outcome measures: n=12 sites; [f]Research coordinators: n=12 sites

**Discussion:** Trials with time-critical interventions are challenging, particularly in the context of a trauma call, but can be successfully delivered through pragmatic study design, a high quality multidisciplinary team approach, and ongoing review and support from the lead investigators and dedicated Clinical Trials Unit.

## PS4C
### - O3 Optimising surgical trials through clinician engagement: Strategies for enhancing trainee engagement in trials

Athene Lane[1], Clare Clement[1], Karen Coulman[1], Jane Blazeby[1], Nick Heywood[2], Natalie Blencowe[1], Jonathan Cook[3], Angelos Kolias[4], Graeme MacLennan[5], Claire Snowdon[6], Zoe Hilton[7], Pauline Boyle[8], Victoria Hardy[8], Laura Magil[9], James Glaseby[10], Dion Morton[10], Dmitri Nepogodiev[10], Thomas Pinkney[10]
[1]University Of Bristol, United Kingdom; [2]Manchester University National Health Service Foundation Trust, United Kingdom; [3]University of Oxford, United Kingdom; [4]University of Cambridge, United Kingdom; [5]University of Aberdeen, United Kingdom; [6]London Schoolof Hygiene and Tropical Medicine, United Kingdom; [7]Cardiff and Vale University Health Board, United Kingdom; [8]NIHR Clinical Research Network West Midlands, United Kingdom; [9]University of Birmingham Clinical Trials Unit, United Kingdom; [10]University of Birmingham, United Kingdom
*Trials* 2019, **20(Suppl 1):**PS4C

**Introduction:** Over 34 surgical Trainee Research Collaboratives (TRCs) have been established across the UK which allow surgical trainees to join large collaborative research groups focused either geographically or by clinical speciality. These networks can generate new trials, improve recruitment to ongoing trials and develop a research-active consultant workforce. TRCs have completed several surgical trials, including the ROSSINI trial in 21 UK centres led by the West Midlands' collaborative. The reasons for TRC successes have not been evaluated but understanding them is key to potential wider translation to other clinical specialities.
**Methods:** Semi-structured interviews on experiences of participating in surgical trials involving TRCs, including barriers and facilitators, and observation of trial and TRC meetings (n = 5) were undertaken. TRCs and linked personnel were purposefully sampled to include a range of key stakeholders and surgical specialties across different geographical locations in the UK. 32 interviews were audio-recorded, transcribed and analysed thematically, alongside observation field notes. Findings were synthesised and presented at an external stakeholder meeting in 2018 with 13 experienced trialists, nurses and surgeons. The meeting identified key strategies to enhance clinicians' engagement in trials across specialities and inform post-graduate training.
**Results:** Analysis revealed four main themes: trainee motivations and barriers to engaging with trials, perceived benefits, and challenges to TRCs and overcoming these challenges. The most important strategies identified were: identifying a Consultant champion to support TRCs, conducting a "flagship" study over a relatively short duration to promote the TRC and motivate trainees, transparency about what is involved for trainees, facilitating trainees to generate study ideas and becoming Co-CIs or Co-PIs, increasing research training and working more closely with trial methodologists.
**Discussion:** If implemented these strategies could overcome the challenges identified and enhance clinician engagement and trial conduct within surgery and other specialties. Further work is exploring this impact.

## PS4C
### - O4 Design of Vaccine Efficacy Trials for Priority Emerging and Epidemic Diseases

Conall Watson[1,2], Neil Ferguson[3], Christl Donnelly[1,3], Matt Keeling[4], Mike Tildesley[4], Peter Horby[1], John Edmunds[2]
[1]University Of Oxford, United Kingdom; [2]London School of Hygiene & Tropical Medicine, United Kingdom; [3]Imperial College, London, United Kingdom; [4]University of Warwick, United Kingdom
*Trials* 2019, **20(Suppl 1):**PS4C

**Introduction:** Vaccine efficacy (VE) trials use clinical trial frameworks yet are not "clinical" trials: where disease natural history in patients drives clinical trial design, understanding infectious disease transmission in populations underpins VE trials. Design of VE trials can be further complicated by the sporadic nature and uncertain duration of epidemics, alongside occurrence in resource-poor settings, and public health imperatives to rapidly end transmission.
The NIHR-funded Vaccine Efficacy Evaluation for Priority Emerging Diseases (VEEPED) project builds on the investigators' experience in responding to Ebola in West Africa, including in the design of the WHO's novel ring vaccination cluster randomised trial which demonstrated efficacy for the rVSV-ZEBOV vaccine in Guinea.
VEEPED aims to determine appropriate VE trial designs for priority emerging infectious diseases and to make recommendations to triallists and policymakers on factors determining the feasibility or otherwise of VE trials under different trial designs and epidemic circumstances.
**Methods:** VEEPED applies epidemiological evidence review and transmission modelling to high-pathogenicity infectious diseases which have been prioritised by the UK Vaccine Network and the WHO R&D Blueprint. Through consideration of epidemic dynamics and application of practical findings from studies such as the Ebola ring vaccination trial, VEEPED evaluates VE trials designs to assess those which may most feasibly demonstrate efficacy for novel vaccine candidates and appropriateness of different control strategies. The project considers both specific priority diseases as well as broader design principles based on epidemiological characteristics.
**Timing of Potential Results:** Preliminary findings of the VEEPED project will be available in October 2019. Full findings will be available in late 2020.
**Potential Relevance & Impact:** Inappropriate VE trial designs divert resources and delay determination of efficacy for candidate vaccines. Addressing VE trial design in the inter-epidemic periods strengthens the prospect of rapid VE evaluation and deployment of effective vaccines.

## PS4C
**- O5**
**Abstract withdrawn**

## PS5A
### - O1 When to do an external or internal pilot study: Findings from an interview study with research funders

Katherine Fairhurst[1], Kerry Avery[1], Alicia O'Cathain[2], Pat Hoddinott[3], Jane Blazeby[1]
[1]Centre of Surgical Research & Medical Research Council (MRC) ConDuCT-II (Collaboration and innovation for Difficult and Complex randomised controlled Trials In Invasive procedures) Hub for Trials Methodology Research, Bristol Medical School, Department of Population Health Sciences, University of Bristol, United Kingdom; [2]School of Health Related Research (ScHARR), University of Sheffield, United Kingdom; [3]NMAHP-RU (Nursing, Midwifery and Allied Health Professions Research Unit), University of Stirling & Medical Research Council (MRC) ConDuCT II Hub for Trials Methodology Research, United Kingdom
*Trials* 2019, **20(Suppl 1):**PS5A

**Introduction :** A standalone external pilot explores the feasibility of performing a definitive RCT, with outcome data not routinely combined with data from the subsequent RCT. An internal pilot is designed and conducted as the first phase of an RCT, with outcome data included in the main analysis. When to perform an internal or external pilot is poorly understood. Qualitative work is needed to explore the views and perceptions of funders regarding how, when and why to choose an external or internal pilot study design.
**Methods:** Purposive sampling identified participants from UK funding panels including NIHR (HTA/RfPB/EME/PGfAR) CRUK, CSO and ARUK. Maximum variation sampling ensured inclusion of multiple characteristics, including chair/deputy chair/member positions on different funding panels and various methodological roles. Semi-structured interviews performed face-to-face or by telephone using a topic guide explored participants' views and practices of funding pilot work. Data analyses were conducted according to principles of thematic analysis, in an iterative and cyclical process as further interviews were conducted and until no new themes emerged or evolved.
**Results:** Of 27 participants contacted, 19(70%) consented and were interviewed in three iterative phases (mean duration 59minutes, range 30-88). Most participants agreed an external pilot design should be chosen when substantial uncertainty exists about one or more design parameters. Of these parameters, a stable, deliverable and acceptable intervention was perceived by most as essential for proceeding to a main trial. Some discussed how staged funding for external pilot studies progressing to a feasible main trial could improve efficiency and limit waste, through avoiding conduct of studies with little hope of main trial funding. Others felt an open ended funding strategy presented significant logistical difficulties, despite it's appeal.
**Conclusion:** Future work will focus on developing recommendations for when to do an external pilot, and establishing whether a flexible design model is possible.

## PS5A
### - O2 Exploring patient treatment preferences enhances trial recruitment, so why do trial recruiters often avoid doing it?

Frances C. Sherratt[1], Lucy Beasant[2], Esther Crawley[2,3], Nigel J. Hall[4], Priya Francis[5], Helen Hickey[5], Carrol Gamble[5], Michael D. Jenkinson[6,7], Bridget Young[1]
[1]Institute of Population Health Sciences, University Of Liverpool, Liverpool, UK; [2]Centre for Child and Adolescent Health, School of Social and Community Medicine, University of Bristol, Bristol, UK; [3]Centre for Surgical Research, School of Social and Community Medicine, University of Bristol, Bristol, UK; [4]University Surgery Unit, Faculty of Medicine, University of Southampton, Southampton, UK; [5]Clinical Trials Research Centre, University of Liverpool, Liverpool, UK; [6]The Walton Centre NHS Foundation Trust, Liverpool, UK; [7]Institute of Translational Medicine, University of Liverpool, Liverpool, UK
*Trials* 2019, **20(Suppl 1):**PS5A

**Introduction:** Patient treatment preferences are one of the most common preventable reasons for poor trial recruitment. Exploring treatment preferences during trial consultations entails eliciting and acknowledging the reasons for a patient's preference and providing information to balance treatment views. Doing so can improve informed consent, trial recruitment and retention. We examined how trial recruiters respond to treatment preferences during consultations and recruiters' views about exploring treatment preferences.
**Methods:** Transcribed audio-recordings of 128 trial consultations from 97 patients and semi-structured interviews with 53 trial recruiters (surgeons, oncologists, and nurses) from two multicentre trials (CONTRACT ISRCTN15830435; ROAM/EORTC-1308 ISRCTN71502099). Data analysis was thematic.
**Results:** Initially, few recruiters elicited treatment preferences but following training they increasingly did so. However, contrary to the training, recruiters' exploration and balancing of preferences tended to be asymmetrical - they particularly avoided exploring and balancing preferences when the patient's preference aligned with the recruiter's own preference. In one of the trials, this often resulted in the patient declining to participate. Recruiters spoke of being reluctant to explore and balance preferences and some attributed this to concerns about unduly influencing patients to participate. Some thought preference exploration would take too much time or would conflict with their clinical responsibilities to advise patients about treatments.
**Discussion:** Despite trial communication training, recruiters were hesitant to explore patient treatment preferences. Consequently, patients will often be relying on suboptimal information about treatments to inform their decisions about trials. Emphasising that preference exploration, regardless of the recruiter's own preference, is consistent with a supported and informed approach to decision-making could help to overcome recruiters' concerns. Evidence on the perspectives of patients on treatment preference exploration would inform recruiter training and practice. Trialists also need to consider the potential impact of recruiter biases on trial communication when designing future trials that compare markedly different treatments.

## PS5A
### - O3 Review of use of the Trials within Cohorts (TwiCs) design approach

Clare Relton[1], Beverley Nickolls[1], Merrick Zwarenstein[4], Lars G Hemken[5], Rudolf Uher[3], Ole Frobert[6], Philippa Fibert[7], Mahukh Imran[8], Linda Kwakkenbos[9], Brett D Thombs[2]
[1]Queen Mary University Of London, London, United Kingdom; [2]McGill University and Jewish General Hospital, Montreal, Canada; [3]Dalhousie University, Department of Psychiatry, Halifax, Canada; [4]Department of Family Medicine, Epidemiology & Biostatistics, University of Western Ontario, London, Canada; [5]Basel Institute for Clinical Epidemiology and Biostatistics , Basel, Switzerland; [6]School of Medical Sciences, University of Orebro, Orebro, Sweden; [7]ScHARR, University of Sheffield, Sheffield, UK; [8]Lady Davis Institute for Medical Research, Jewish General Hospital, Montréal, Canada; [9]Behavioural Science Institute, Clinical Psychology, Radboud University, Nijmegen, Netherlands
*Trials* 2019, **20(Suppl 1):**PS5A

**Introduction:** Trials within Cohorts (TwiCs) is an innovative approach to the design and conduct of multiple randomised controlled trials (RCTs) (Relton et al, 2010). This approach utilises an observational cohort to recruit trial populations and obtain short and longer term outcomes. We describe what is currently known about the use of this design approach.
**Methods:** An extension of the 2010 Consolidated Standards of Reporting Trials (CONSORT) Statements for RCTs using cohorts and/or routinely collected health data is in development, supported by a scoping review that includes publications of methods or reports of protocols or results from RCTs using cohorts, registries, electronic health records and administrative databases. Data sources for this scoping review included Medline and Cochrane Methodology Register and were limited to English language.

This review of use of the TwiCs approach uses publications of methods or reports of protocols or results from RCTs that use cohorts to recruit identified in the scoping review. This is supplemented with information from topic experts.
We report: (i) types of cohorts (setting, population, condition/ disease area), (ii) how the cohorts are utilised (identifying potential trial participants, recruitment, randomisation, process and outcome data collection including bespoke and/or routine health record data, types of trials conducted/ planned), (iii) approaches to informed consent, e.g. staged approach (Young-Afat et al, 2016), and (iv) any purported and/or real study design (in)efficiencies.
**Timing of Potential Results:** Early results indicate 75+ eligible full text articles, including 23 trial protocols and 23 articles reporting the results of trials using cohorts. Full results will be available in August 2019 and presented at the conference.
**Potential Relevance and Impact:** Standard approaches to trial design are often costly and frequently fail to recruit sufficiently large or representative samples. This review will help provide information on the use and potential (in)efficiency of the TwiCs approach.

## PS5A

### - O4 Using a Discrete Choice Experiment to Examine the Factors Influencing Clinical Trial Participation

Michelle Queally[1,2], Declan Devane[3], Matthew Griffin[2], Paddy Gillespie[1,2]
[1]J.E. Cairnes School of Business & Economics, NUI Galway, Galway, Ireland; [2]CÚRAM Centre for Research in Medical Devices, NUI Galway, Galway, Galway, Ireland; [3]HRB-Trials Methodology Research Network, Department of Midwifery, NUI Galway, Galway, Ireland
*Trials* 2019, **20(Suppl 1):**PS5A

**Introduction:** Understanding the key factors within individuals' decision making process with respect to clinical trial participation has the potential to improve both the efficiency of recruitment for clinical trials and their management. Currently little known about the relative importance of the different factors influencing individuals' decisions regarding clinical trial participation. The objectives of this study were to use a discrete choice experiment (DCE) to: (1) identify key factors important to individuals regarding participation in clinical trials, (2) better understand how individuals' value and trade-off between these factors; and (3) estimate willingness-to-pay for these factors.
**Methods:** A literature review, four focus groups, two consultations with a public and patient involvement (PPI) group and three consultations with expert groups of clinicians and researchers were conducted to select attributes and levels for the DCE. The Nominal Group Technique (NGT) which is a structured, multistep, facilitated group meeting technique was used to elicit and prioritize responses to identify the most important attributes in clinical trial participation.
**Timing Potential Results:** Six attributes ranked most important regarding clinical trial participation were identified: (1) Communication, (2) Knowledge, (3) Risk, (4) Benefit, (5) Incentive; and (6) Follow up time of the trial. A Bayesian efficient DCE design to include twelve choice sets was piloted (n=45). The main survey will be administered (May 2019) to a nationally representative sample in Ireland (n=500).
**Potential Relevance and Impact :** To the best of our knowledge, this is the first study to utilise a DCE to measure and quantify preferences of individuals regarding factors influencing clinical trial participation. The incorporation of choice task questions will enable trial researchers to elicit how individuals weigh up their choices and consider trade-offs between the attributes. This study will also be the first to include a PPI panel throughout the DCE design and development.

## PS5A

### - O5 Physical Rehabilitation Core Outcomes in Critical Illness (PRACTICE): a secondary modified thematic analysis characterising reasons for change in rating importance of outcomes for physical rehabilitation trials

Bronwen Connolly[1,2,3], Matthew Barclay[1], Evelyn Corner[4], Chantal Davies[5], Nicholas Hart[1,2], Natalie Pattison[6], Gordon Sturmey[5], Linda Denehy[3], Dale Needham[7], Paula Williamson[8], Bronagh Blackwood[9]
[1]Guy's and St.Thomas' NHS Foundation Trust, United Kingdom; [2]King's College London, United Kingdom; [3]The University of Melbourne, Melbourne, Australia; [4]Brunel University, Uxbridge, United Kingdom; [5]Independent ICU Representative, United Kingdom; [6]University of Hertfordshire and East & North Hertfordshire NHS Trust, Hatfield, United Kingdom; [7]Johns Hopkins University, Baltimore, United States of America; [8]University of Liverpool, United Kingdom; [9]Queen's University Belfast, United Kingdom
*Trials* 2019, **20(Suppl 1):**PS5A

**Introduction:** Trials of physical rehabilitation in critical illness across the continuum of recovery are challenged by outcome heterogeneity. PRACTICE is an international, mixed-methods study designed to develop a core outcome set (COS) for such trials. Following completion of stage one of PRACTICE, where outcomes for inclusion in the core set were agreed, we sought to explore participant reasons for changing scoring of outcome importance during the consensus process.
**Methods:** A preliminary secondary analysis of a two-round modified Delphi consensus process involving multiprofessional researcher and clinician, and patient/caregiver stakeholder groups, in which participants rated the importance of 30 outcomes for inclusion in the COS using a 9-point scale (1-3, not important; 4-6, important but not critical; 7-9, critical). Feedback from scoring in the first round was provided in the second round, presented for overall participants and per stakeholder group. Where a participant changed their rating of an outcome resulting in subsequent change in importance category, they were prompted to provide a reason for that change. Changes were classified as 'positive' or 'negative' indicating either an increase or decrease in outcome importance, respectively. A modified thematic analysis was conducted to characterise reasons for change.
**Results:** 301 participants completed both rounds of the consensus process. In total, 832 reasons for change were provided (n=627 positive, n=205 negative). Fourteen themes characterising reasons for change were identified, the most common of which were i) 'Considering the impact on other outcomes' (n=179, 21.5%), ii) 'Being influenced by the patients' responses' (n=174, 20.9%), and iii) 'Reconsideration' (n=107, 12.9%).
**Discussion:** Multiple factors influenced rating of outcome importance for inclusion in the PRACTICE COS. That patient opinion featured heavily in decision-making highlights the value of their contribution to the COS development process, and may also infer the importance of patient involvement in primary study design.

## PS5B

### - O1 Open-cohort designs in institutional settings: findings from a literature review of cluster-randomised trials and epidemiological studies

Laura Marsden[1], Rebecca Walwyn[1], Andrew Copas[2], Claire Surr[3], Amanda Farrin[1]
[1]University of Leeds, Leeds, United Kingdom; [2]University College London, London, United Kingdom; [3]Leeds Beckett University, Leeds, United Kingdom
*Trials* 2019, **20(Suppl 1):**PS5B

**Introduction:** Cluster-randomised trials (CRTs) are often implemented in institutions such as care homes, schools, hospitals, prisons, and whole communities. These institutions can be defined as 'open cohorts,' as individuals join and leave the cluster over the study period. Two accepted designs currently exist for CRTs. Closed-cohort designs follow the same individuals over time, allowing us to assess intervention effects on individuals. As care home CRTs suffer unavoidably high drop-out rates, mainly due to death, use of a closed-cohort design can result in substantial missing data, introducing bias and leading to questionable conclusions. Alternatively, cross-sectional designs collect data from different groups of individuals at the start and end of a trial. Whilst unaffected by drop-outs, this design only permits analysis of change at the population-level, not on individuals.
We propose an 'open-cohort design' which accounts for migration and uses data from all individuals present in the cluster between baseline and follow-up, allowing us to generalize at both individual- and cluster-level and assess long-term effects. Whilst open-cohort designs are not currently widely recognised as an alternative for clinical trials, open-cohort studies are conducted in epidemiology.
**Methods:** A review of epidemiological studies and CRTs in care homes, schools, hospitals and other settings was conducted. We explore the extent to which open-cohort designs have, or could have, been used across these settings. Practical challenges arising in implementation and analysis, generally or specific to setting, are investigated.
**Timing of potential results:** The review is underway and expected to be completed by June 2019.
**Potential relevance and impact:** Open-cohort designs have obvious advantages; fewer clusters would need to be recruited, reducing costs, and research questions targeting all those exposed to the intervention during the study period could be addressed. However, research is needed to determine how this design complicates the conduct, analysis and interpretation of CRTs.

## PS5B
### - O2 Power calculations for Cluster Randomised Trials (CRTs) with truncated Poisson-distributed outcomes: A motivating example from a malaria vector control trial

Lazaro M. Mwandigha[1], Keith J. Fraser[1], Amy Racine[2,3], Samer Mouksassi[3,4], Azra C. Ghani[1]
[1]MRC Centre for Global Infectious Disease Analysis, Imperial College London, London, United Kingdom; [2]Novartis Pharma AG, Basel, Switzerland; [3]Bill & Melinda Gates Foundation, Seattle, United States of America; [4]Certara, Montreal, Canada
*Trials* 2019, **20(Suppl 1):**PS5B

**Background:** Cluster-randomised trials are increasingly used to study the efficacy of interventions targeted at the population level. Whilst formulae exist to calculate sample sizes taking into account the intra-cluster correlation, they assume that the outcome under consideration covers the full range of allowable values. This assumption is frequently violated in epidemiological trials in which counts of infection episodes are truncated due to practical constraints on the number of times a person can be tested.
**Methods:** Motivated by a malaria vector control trial with right-truncated Poisson-distributed outcomes, we investigated the effect of truncation on power using Monte Carlo simulations.
**Results:** The results demonstrate that the adverse impact of truncation is directly proportional to the magnitude of the event rate λ with estimates of power being overestimated in instances where the truncation was not accounted for. The severity of the adverse impact of truncation on power increased with magnitude of the intra-cluster correlation (ICC) but decreased the further the truncation point was from zero.
**Discussion:** Potential truncation should therefore be accounted for in the estimation of sample size requirements and power at the study design stage.

## PS5B
### - O3 Comparison of different randomisation methods in a cluster randomised vaccine effectiveness trial: a simulation study using real-world data

Xinxue Liu[1], Merryn Voysey[1], K Zaman[2], Farhana Khanam[2], Firdausi Qadri[2], Andrew Pollard[1], John Clemens[2]
[1]Oxford Vaccine Group, Department of Paediatrics, University of Oxford, Oxford, United Kingdom; [2]International Centre for Diarrhoeal Disease Research – Bangladesh (icddr,b), Dhaka, Bangladesh
*Trials* 2019, **20(Suppl 1):**PS5B

**Background:** Cluster randomised controlled trials (CRCTs) of vaccine effectiveness randomly allocate all individuals in a geographically-defined cluster to receive either the test vaccine or a control vaccine according to their cluster of residence. Randomisation by cluster rather than by individual can result in the baseline characteristics of individuals being imbalanced between the treatment groups. The lack of balance at baseline will increase the variance of the estimated effect and thus reduce the efficiency of the study.
**Methods:** We conducted a simulation study using census data from a CRCT (Typhoid Vaccine Acceleration Consortium-Bangladesh, TyVAC-Bangladesh) to compare three randomisation methods (simple block randomisation, stratified block randomisation, and restricted randomisation) in three different scenarios: all 150 clusters in TyVAC-Bangladesh, or a subset of 50 or 20 randomly selected clusters. For each randomisation method, we generated 1000 randomisation lists allocating each cluster to either typhoid vaccine or control. For each baseline characteristic, imbalance was defined as ≥10% difference between the two arms. We assessed the performance of each randomisation method by comparing the proportion of simulated randomisation lists with that were imbalanced for each selected baseline characteristics.
**Results:** For individual-level continuous normally distributed variables, such as age, all randomisation methods achieved perfect balance. For continuous cluster-level variables (such as the number of male participants in the cluster), the performance of the randomisation method depended on the variable's distribution. For highly skewed variables, most methods had imbalance proportions >70%. However, when restricted randomisation was used with the skewed variable as a design variable, the imbalance proportion dropped to 20%. Scenarios with large numbers of clusters were less likely to be imbalanced.
**Conclusion:** Choosing the right design variables for cluster randomisation is important to achieve good baseline balance in CRCTs. Outcome predictors with highly skewed distribution at cluster-level should to be incorporated as a design variable.

## PS5B
### - O4 Common concerns about the feasibility of stepped-wedge cluster randomised trials and issues encountered during trials of this design: findings of an online questionnaire

Caroline Kristunas[1], Karla Hemming[2], Helen Eborall[1], Laura Gray[1]
[1]Health Sciences, University of Leicester, Leicester, United Kingdom; [2]Institute of Applied Health Research, University of Birmingham, Edgbaston, United Kingdom
*Trials* 2019, **20(Suppl 1):**PS5B

**Introduction:** Stepped-wedge cluster randomised trials (SW-CRTs) are a complex design with features that may increase the likelihood of the trial encountering issues that may impact its feasibility. The aim of this work was to determine the common issues affecting the feasibility of SW-CRTs.
**Methods:** An online questionnaire, consisting of closed questions and free-text responses, asked respondents about their involvement in SW-CRTs, concerns about the feasibility of the trial and any problems encountered. Participants were also asked about any concerns they had about SW-CRTs in general. Potential participants were identified from the authors of published SW-CRTs, list of delegates at conferences about SW-CRTs, panel member pages of funders' websites, and the websites of clinical trials units and the Research Design Service. Recipients were encouraged to forward their email invitation onto anyone that might be interested in participating. Open invitations were posted on Twitter. A descriptive analysis was conducted.
**Results:** Email invitations were sent to 403 individuals, 154(38%) responded, mostly trialists (66%) and funding panel members (16%). Most participants had been involved with a SW-CRT (82%) and had concerns about some aspect of the design (94%). Some of the most common concerns related to the staggered implementation: not having all clusters ready when the trial starts; clusters not willing or able to start the intervention when randomised to; when to inform clusters of when they'll start the intervention; and retention of the last clusters to start the intervention.
**Discussion:** There are features of the SW-CRT design which have the potential to impact the feasibility of the trial. In particular, the staggered implementation of the intervention, although often one of the main reasons for choosing this design, can cause problems. The additional challenges of staggering the implementation of the intervention need to be weighed against the potential benefits.

## PS5B

### - O5 Optimal incomplete stepped wedge trials with continuous recruitment

Richard Hooper[1], Andrew Forbes[2], Jessica Kasza[2]
[1]Queen Mary University of London, LONDON, United Kingdom; [2]Monash University, Melbourne, Australia
*Trials* 2019, **20(Suppl 1):**PS5B

**Introduction:** We consider the problem of optimal design for a stepped wedge trial with continuous recruitment. Suppose we recruit from a fixed number of clusters where eligible participants present continuously over a fixed duration, and suppose we have a fine degree of control over the timing of cross-over in each cluster. Suppose also that we want to minimise the number of participants, leading us to consider "incomplete" designs (which do not recruit for the entire duration at every cluster). How should we schedule recruitment and cross-over at different clusters to achieve given precision with the fewest participants?
**Methods:** The optimality of incomplete designs is poorly understood. Time here is continuous, and the time effect modelled as a polynomial, but to simplify we assume that eligible participants present at regular intervals. Without exploring every possible design there is no guarantee of finding a global optimum, but an iterative algorithm which makes slow, steady modifications may come close. We demonstrate one such approach. At each iteration (starting from a complete design) a single participant – the participant making the least impact on precision – is removed, and then small changes preserving the total sample size are made repeatedly to improve precision until no further improvement is possible. This continues until further iterations would compromise the desired precision. To triangulate the solution the algorithm restarts with a very sparse design, adding a participant at each iteration until the desired precision is achieved.
**Results:** Unusual and striking shapes emerge. Solutions tend to focus recruitment and cross-over on the leading diagonal of the cluster-by-time diagram, but in some scenarios clusters become organised into distinct steps or phases.
**Discussion:** There is evidently much to be learned about optimal trial design in this setting. Algorithmic searches could offer a practical approach to trial design in complex settings.

## PS5C

### - O1 Health System Trials

Clare Relton[1], Merrick Zwarenstein[2], Lars G Hemkens[3], Helena M Verkooijen[4], Ole Frobert[5]
[1]Pragmatic Clinical Trials Unit, Queen Mary University Of London, London, United Kingdom; [2]Western University, Toronto, Canada; [3]Basel Institute for Clinical Epidemiology and Biostatistics, Department of Clinical Research, University Hospital Basel, University of Basel, Basel, Switzerland; [4]University Medical Centre Utrecht, Utrecht, The Netherlands; [5]School of Medical Sciences, Orebro University, Orebro, Sweden
*Trials* 2019, **20(Suppl 1):**PS5C

Pragmatic randomised trials aim to provide evidence to support decisions by stakeholders in healthcare systems (patients, clinicians, funders, policy makers). The typical pragmatic trial recruits participants who provide data for the trial using purpose built data collection systems. At the end of the trial – all is disbanded. This approach is costly and frequently fails to recruit sufficiently large or representative samples.
Since the advent of electronic data, pragmatic trials are increasingly using routine health data collected from administrative, clinical and patient sources. A new group of trial designs have emerged which we describe as 'Health System Trials'. These include Registry-based Randomised Controlled Trials (RRCTs), Electronic Health Record (EHR) Trials, Administrative Data (AD) Trials and Trials within Cohorts (TwiCs). These four designs purposefully utilise existing and/or newly created health system data structures for one or more trial activities: identifying potential trial participants, recruitment, randomisation, process and outcome data collection, etc. The process of informed consent is often spread out (staged) as occurs in routine healthcare especially with TwiCs designs.
By utilising populations within health systems and the data that derives from their healthcare encounters, these trials efficiently recruit large representative populations and obtain both short and longer term outcomes. These designs reduce the effort and cost of trials whilst improving the applicability of the trial results for decision makers in health systems.
We discuss the opportunities for these types of trial designs to be integrated within health systems, enabling the continuous generation of knowledge that is an essential feature of learning health systems.
CONSORT Reporting guidelines for Trials Using Cohorts and Routine Health Data are currently being developed. Drawing on development work for these guidelines we describe real world examples of 'Health System Trials', including examples of both nascent vertical (disease focused) and horizontal (e.g. practice based) learning health systems.

## PS5C

### - O2 Dealing with real world data in clinical trials

Catriona Keerie[1], Christopher Tuck[1], Ronald Harkess[1], Fiona Strachan[2]
[1]Edinburgh Clinical Trials Unit, University Of Edinburgh, United Kingdom; [2]British Heart Foundation Centre for Cardiovascular Science, University of Edinburgh, United Kingdom
*Trials* 2019, **20(Suppl 1):**PS5C

**Introduction:** The High-STEACS trial (ClinicalTrials.gov Identifier: NCT01852123) was a randomised controlled trial, enrolling 48,282 patients between 2013 and 2016. The trial's objective was to determine if implementation of a new high-sensitivity troponin assay would improve outcomes in patients presenting with suspected acute coronary syndrome to hospital emergency departments across Scotland.
The trial was unusual in making use of routine electronic healthcare data in unconsented patients. This presented a number of data management and governance challenges.
**Methods:** The trial accessed routine electronic healthcare data sources from ten hospitals in two NHS health boards in Scotland. Participant data were linked across twelve distinct data sources using the participant CHI (Community Health Index) number as a unique identifier.
Data extraction was supported by the NHS Safe Haven of each health board and eligible patients were assigned a unique study ID prior to removing identifiable participant data. De-identified data were transferred to a secure analysis platform and combined into a single database for statistical analyses, accessible only to approved individuals.
The study had ethical and local management approval for record linkage.
**Results:** Reporting of the trial presented challenges not encountered in conventional randomised controlled trials. Data linkage was complex – collection of the correct data during the correct timeframe from multiple data sources was challenging. Creation of a 'meta database' whilst ensuring data accuracy was made possible by drawing on a number of securely linked raw NHS data sources.
**Discussion:** The High-STEACS trial successfully reported on a large number of patients via routinely collected healthcare data. Using data from electronic health records without individual patient consent required adherence to rigorous data governance processes. This approach enabled all patients to be identified, rather than limiting findings to a selected, possibly unrepresentative, group which would have been the case in a traditional clinical trial setting.

## PS5C

### - O3 Using routine practice-aggregated data in primary care implementation laboratory trials: benefits and challenges

Sarah Alderson[1], Tracey Farragher[2], Paul Carder[3], Thomas Willis[1], Amanda Farrin[1], Robbie Foy[1]
[1]University Of Leeds, Leeds, United Kingdom; [2]University of Manchester, Manchester, United Kingdom; [3]West Yorkshire Research and Development, Bradford, United Kingdom
*Trials* 2019, **20(Suppl 1):**PS5C

**Background:** Trials within implementation laboratories involve collaborations between healthcare systems and research teams to address health systems' priorities and produce generalisable knowledge about factors—context, intervention design, and delivery—that could influence effectiveness of feedback interventions. Sequential head-to-head trials can compare various consecutive refinements of feedback interventions with embedded process evaluations to examine mechanism of action and effect modifiers. Such incremental improvements could lead to faster changes in policy, address healthcare system priorities, and advance improvement science.
Primary care data sources for feedback interventions include large-scale databases (General Practice Research Database), high-level nationally gathered databases (OpenPrescribing.com) or data extracted directly from electronic health records (EHR). Different sources of data have implications for implementation laboratory trial design.
**Methods:** The Campaign to Reduce Opioid Prescribing is a primary care feedback intervention that has led to a deeper understanding of the utility and challenges of primary care data sources.
316 practices in West Yorkshire were provided with bimonthly feedback on opioid prescribing for non-cancer pain. Effectiveness of this enhanced feedback intervention was assessed using an interrupted time-series design.
**Results:** High-level, nationally gathered data showed opioid prescription rates fell significantly in intervention practices during the post-intervention period compared to control practices (0.05 prescriptions per 1,000 patients (95%CI -0.10, -0.01)). Locally extracted EHR data gives a more in-depth analysis by reducing 'noise' (exclusion of opioids prescribed for palliative care), potential unintended consequences (increased referrals) and demonstrates changes in clinically relevant sub-groups, however data extraction was complex and identified challenges in collection.
**Discussion:** UK primary care data sources are heterogeneous, with different purposes, structures and collection methods. The utility of data sources have implications for conducting pragmatic trials that will have consequences for methodological advances in the development of 'implementation laboratories' and will be integral to the design of future trials in this field.

## PS5C

### - O4 COS and the healthcare research ecosystem

Susanna Dodd, Paula R Williamson
University Of Liverpool, Liverpool, United Kingdom
*Trials* 2019, **20(Suppl 1):**PS5C

**Introduction:** There is growing interest in identifying how core outcome sets (COS) might fit into the different stages of the healthcare research and delivery ecosystem, and how this might be facilitated. It has been suggested that COS may be used to inform clinical guidelines, audit, quality standards (QS) and quality indicators (QI) (1). If so, the potential of electronic health records (EHR) to facilitate comparative effectiveness research by providing a readily available source of data would be greatly enhanced as the outcome data being collected in routine practice and the outcomes chosen for this type of research would be the same.
**Methods:** We will compare COS for research with COS for routine care, matched by condition. We will present a 'proof of concept' case study in type 2 diabetes (T2D), mapping outcomes across COS, QS, QI, and EHR.
**Results:** To date, 275 COS studies (333 COS) for research and an additional 32 COS studies (35 COS) for both research and routine practice have been published (2). Of 257 ongoing COS registered in the COMET database, 131 are for research and 10 for routine care, with the remaining 116 being developed for both research and routine care. Sixteen (89%) of the 18 core outcomes included in the SCORE-IT T2D COS (3) were represented within the ICHOM diabetes set for routine care (4), all were recorded within CPRD, and 13 (72%) and 14 (78%) featured in the NICE QS (5) and QI set (6) for T2D respectively.
**Discussion:** This talk will focus on the importance of and opportunity for choosing outcomes to measure that matter to patients and decision-makers, and the barriers and facilitators for core outcomes to be incorporated into routine data collection.

1. https://doi.org/10.1177/0046958018761495
2. https://doi.org/10.1371/journal.pone.0209869

3. https://doi.org/10.1186/s13063-018-2805-2
4. https://www.ichom.org/portfolio/diabetes/
5. https://www.nice.org.uk/guidance/qs6
6. https://www.nice.org.uk/Media/Default/Standards-and-indicators/indicator-menu-update.pdf

## PS5C

### - O5 MOUSE – Mapping OUtcomes measured in pre-clinical Studies against randomised phase 3/4 Effectiveness trials. Do core outcome sets developed for phase3/4 effectiveness trials translate to pre-clinical research?

Nicola L Harman[1], Adrián Sanz-Moreno[2], Kamar E Ameen-Ali[3], Stamatia Papoutsopoulou[1], Katie Lloyd[1], Marta Garcia-Finana[1], Malcolm MacLeod[4], Paula R Williamson[1], on behalf of the SCORE-IT study team
[1]University Of Liverpool, United Kingdom; [2]Helmholtz Zentrum München, Germany; [3]Newcastle University, United Kingdom; [4]University of Edinburgh, United Kingdom
*Trials* 2019, **20(Suppl 1):**PS5C

**Introduction:** Translational failure from pre-clinical animal studies to clinical trials has been noted in a number of disease areas. Whilst multiple contributory factors including poor study conduct and reporting have been acknowledged, little attention has been given to whether outcomes measured in pre-clinical studies are relevant to those considered important in clinical trials. Core Outcome Sets (COS) aim to reduce waste in research by defining a minimum set of outcomes to be used in all trials of a particular condition. However, these have been developed for phase 3/4 effectiveness trials and their utility in pre-clinical research is unknown.
**Methods:** To better understand the translatability of outcomes a systematic review of outcomes used in preclinical pharmacological interventions for type 2 diabetes in mouse models will be completed. We will extract exact descriptions of outcomes and categorise these according to the COMET taxonomy. The list of outcomes will then be compared to outcomes identified in a systematic review of phase 3/4 trials of glucose lowering interventions. Preclinical outcomes will also be reviewed against the outcomes considered most important, and recently included in a COS, by people with type 2 diabetes, healthcare professionals, researchers, and policymakers.
**Timing of Potential Results:** Results of the pre-clinical systematic review and extracted outcomes will be presented. The core outcome set for glucose lowering interventions for type 2 diabetes and systematic review of phase 3/4 studies have been completed.
**Potential Relevance & Impact:** This review of pre-clinical studies will enable better understanding of the outcomes measured at different phases of research and the translatability of COS. The use of established COS in pre-clinical studies may also provide a way for patients to influence pre-clinical research to make it more relevant to their needs and contribute to the refinement of animal studies and overall reduction of animals in research.

## PS5D

### - O1 Using data from routine sources in the development of an objective measure of early outcome after surgery

Rachel Maishman[1], Ben Gibbison[2], Barney Reeves[1], Chris Rogers[1]
[1]Clinical Trials and Evaluation Unit, Bristol Trials Centre, University of Bristol, Bristol, UK; [2]Department of Cardiac Anaesthesia and Intensive Care, Bristol Heart Institute/University Hospitals Bristol NHS FT, Bristol, UK
*Trials* 2019, **20(Suppl 1):**PS5D

**Introduction:** In trials in surgery, post-operative length of hospital stay (LoS) is often used as a proxy for early outcome. However, blinding of surgeons is typically not possible, which leads to potential for bias, as the surgeon decides when a patient is ready for discharge. This has led us to investigate how routinely collected clinical measures during the first 24 hours and complications that occur throughout the post-operative stay could be used to develop a more objective measure of early outcome.
**Methods:** Measurements routinely collected during the first 24 hours after surgery have been extracted from hospital electronic records and combined with trial data for a cohort of patients who have previously participated in a cardiac surgery RCT. Relationships between six measurements (summarised over the first 24 hours) and LoS have been investigated to identify early indicators of poor outcome. The six measures were chosen in discussion with clinician colleagues.
**Provisional results:** A total of 808 patients who had participated in one of 7 trials were included. Initial univariable and multivariable analyses suggest higher arterial mean blood pressure and higher minimum haemoglobin were significantly associated with shorter LoS, while higher maximum lactate and maximum potassium were associated with longer LoS. Heart rate and temperature were not associated with LoS. Effect estimates were attenuated for all measurements in the multivariate analysis. We will also describe the association between these measures and complications and how these are being combined, alongside patient and clinician “ratings” of complication severity, to develop of an objective measure of early outcome.
**Potential relevance and impact:** Supplementing clinical trial data with routinely collected measurements provides more detailed information on early outcome at minimal additional cost. The methodology to develop this measure is applicable in other clinical areas.

## PS5D

### - O2 Exploring the barriers and facilitators to core outcome set (COS) uptake: assessing the impact of a funder’s recommendation to use COS followed by qualitative interviews with clinical trialists

Karen L Hughes[1], Bridget Young[1], Mike Clarke[2], Jamie Kirkham[1], Paula R Williamson[1]
[1]University Of Liverpool, United Kingdom; [2]Queen's University Belfast, United Kingdom
*Trials* 2019, **20(Suppl 1):**PS5D

**Introduction:** Core outcome sets (COS) improve the consistency and relevance of outcomes in trials but these benefits will only arise if trialists use them. Our first aim was to assess the extent to which trialists followed a funder’s recommendation to search for a COS. Our second aim is to explore trialist’s views on COS.
**Methods:** In January 2012, the NIHR HTA programme updated their guidance to recommend that applicants search for a COS when preparing a funding application for a trial. We examined 95 researcher-led applications submitted to the NIHR HTA from then to December 2015 for evidence of such a search and other rationale for outcome choice. We also surveyed applicants to explore their use of COS and choice of outcomes. Our next step is to conduct semi-structured interviews with researchers working on NIHR HTA clinical trials. Purposive sampling from the NIHR portfolio of research projects will identify participants whose experiences can inform our understanding of outcomes in trials. We will seek data saturation and our analysis will draw on thematic approaches.
**Results:** Results of the qualitative interviews will be available for presentation at the conference but our document-based work showed that 18 of the 95 NIHR HTA applications (19%) stated that a search for a COS had been done. Of the 77 (81%) applications that did not mention COS, our survey of applicants found that 18 had searched for a COS. Some applicants who did not search for COS gave reasons for their choice of outcomes.
**Discussion:** A funder can have an impact on COS uptake by encouraging a search for a COS, but more actions are needed to increase this impact. Our interviews with researchers will provide further information on the barriers and facilitators to COS uptake, which will inform such strategies to improve uptake of COS.

## PS5D
### - O3 Participating in core outcome set development via Delphi surveys: Qualitative interviews from the EPITOME study provide pointers to inform guidance

Alice Mary Biggane[1,2], Paula R Williamson[1], Bridget Young[3]
[1]Department of Biostatistics, University of Liverpool, Liverpool, United Kingdom; [2]INSERM, U1153 Epidemiology and Biostatistics Sorbonne Paris Cité Research Center (CRESS), Methods of therapeutic evaluation of chronic diseases Team (METHODS), Paris Descartes University, Sorbonne Paris Cité, Paris, France; [3]Department of Health Services Research, University of Liverpool, Liverpool, United Kingdom
*Trials* 2019, **20(Suppl 1):**PS5D

**Introduction:** Core outcome sets (COS) represent the minimum outcomes that should be measured and reported in all clinical trials in a specific condition. Input from patients in COS development, and subsequent uptake of COS, will ensure that future studies provide users of research with relevant knowledge regarding interventions. A 2017 survey found that Delphi surveys are being utilised in 89% of ongoing COS with patient participants. It is unclear how patients experience Delphi surveys as part of COS development and whether these methods are suitable for facilitating patient participation. The objective of this study was to explore participants views of the Delphi survey used for COS development.
**Methods:** Patients and health professionals who participated in a Delphi survey as part of a COS study took part in semi-structured qualitative interviews which explored participants' understanding of COS and their experiences of the Delphi survey. Analysis was interpretative and thematic.
**Results:** Twenty-four participants from 7 COS studies were interviewed. They varied in how accurately and fully they understood the purpose of COS and the Delphi survey, which influenced their participatory experience. They also differed in how easily they interpreted and subsequently used the written guidance provided to COS participants. Some participants wanted guidance regarding whose perspective to take into account when scoring outcomes and on how to apply the scoring system. Participants' motivation for taking part included the international and expert consensus aspects of the Delphi survey. A small number of participants raised the positive and negative emotional impact of participation when reviewing outcomes and stakeholder feedback.
**Discussion:** The findings identify ways of improving information for COS Delphi participants to enhance their experience of participation and make the process more meaningful for them.

## PS5D
### - O4 The impact of patient-reported outcome (PRO) data from clinical trials: a systematic review and critical analysis

Samantha Cruz Rivera[1], Derek Kyte[1,2], Olalekan Lee Aiyegbusi[1], Anita Slade[1,2], Christel McMullan[1], Melanie Calvert[1,2]
[1]Centre for Patient Reported Outcomes Research, Institute of Applied Health Research, College of Medical and Dental Sciences, University of Birmingham, UK; [2]NIHR Birmingham Biomedical Research Centre, and NIHR Surgical Reconstruction and Microbiology Research Centre University Hospitals Birmingham NHS Foundation Trust and University of Birmingham, UK
*Trials* 2019, **20(Suppl 1):**PS5D

**Background:** Patient-reported outcomes (PROs) are commonly collected in clinical trials and should provide impactful evidence on the effect of interventions on patient symptoms and quality of life. However, the different types of research impact associated with PRO trial results, appropriate impact metrics and barriers and facilitators are not well defined. Objectives: i) to determine the range of potential impacts from PRO trial data, identify potential PRO impact metrics and identify barriers/facilitators to maximising PRO impact; and ii) to examine real-world evidence of PRO impact based on Research Excellence Framework (REF) 2014 impact case studies.
**Methods:** Two independent investigators searched MEDLINE, EMBASE, CINAHL+, HMIC databases from inception until December 2018. Articles were eligible if they discussed research impact in the context of PRO trial data. In addition, the REF 2014 database was systematically searched for case studies incorporating a trial in which PRO data were collected.
**Results:** Nine types of PRO trial impact were identified; the most frequent of which centred on PRO data informing clinical decision-making. The included publications identified several barriers and facilitators centred around PRO trial design, conduct, analysis and reporting. Twelve (17%) REF case studies outlined demonstrable PRO trial impact; including changes to international and national guidelines, influencing cost-effectiveness analysis and contributing to drug approvals.
**Conclusions:** PRO trial data may potentially lead to a range of impacts and benefits for patients and society, which can be measured through impact metrics. However, in practice, there is relatively limited evidence demonstrating directly attributable real world PRO-related research impact. In part, this is due to the wider challenges of measuring the impact of research and PRO-specific issues around design, conduct, analysis and reporting. Adherence to existing international guidelines is essential to maximise the use of PRO trial data, facilitate impact and minimise research waste.

## PS5D
### - O5 An exploratory study of the limitations of outcome measures used in a randomised controlled trial of a complex intervention in dementia

Benjamin Thompson[1], Gail Mountain[2], Ben Thomas[1], Ellen Lee[1], Bethany Crawford[3]
[1]ScHARR, The University Of Sheffield, Sheffield, United Kingdom; [2]Centre for Dementia Studies, University of Bradford, Bradford, United Kingdom; [3]University of Nottingham, Nottingham, United Kingdom
*Trials* 2019, **20(Suppl 1):**PS5D

**Introduction:** Dementia research uses multiple measures due to the complexity of the condition. Limited dementia-specific scales exist and generic measures are used in their absence. Problems such as the acceptability of responses and respondent fatigue, as well as the use of retrospective recall in a population with recall difficulties are challenges to effective outcome assessment in dementia research.
**Aims:** To explore the limitations of the outcome measures used in a randomised controlled trial of a complex intervention for persons with early stages of dementia.
**Objectives**
-Use retrospective analysis of study data and outcome assessor comments recorded during data collection to identify potentially problematic items or scales and explore participant difficulties in completing the outcome measures.
-To report on the effectiveness of the measures used and make recommendations for future dementia measure development.
**Setting:** 'Journeying through Dementia' is a randomised controlled trial of a community-based self-management intervention for people with early stages of dementia and their carers. 480 people with dementia took part in the trial, and outcome measures were collected face-to-face at baseline, 8-, and 12-month intervals. We selected dementia specific outcome measures based upon recommendations for research across Europe and used general measures where dementia-specific scales were not available.
**Methods:** A retrospective secondary analysis of 8-month follow up data from the trial. Quantitative analysis of missed item responses, missed scales and drop-out points identified potentially problematic items and measures. A narrative review of comments made by outcome assessors explored why participants had trouble in responding to outcome measures.
**Timing of Potential Results:** Potential results will be available in July 2019.
**Potential Relevance and Impact:** We report on the problems experienced using outcome measures in a large scale RCT for dementia. Learning from the Journeying through Dementia trial may guide future trial conduct and outcome measure development.

## PS6A

### - O1 Making trials less lossy: is there anything worth knowing from non-randomised evaluations of trial retention strategies?

Adel El Feky
University Of Aberdeen, Aberdeen, United Kingdom
*Trials* 2019, **20(Suppl 1):**PS6A

**Background:** Poor retention is common. It reduces statistical power and can bias the estimates of intervention effect, especially with differential loss-to-follow across trial arms. Given the sparsity of evidence from randomised evaluations of effective retention strategies, we performed a systematic review to synthesise evidence from non-randomised evaluations to supplement existing evidence.
**Methods:** We searched MEDLINE, Embase, and Cochrane CENTRAL from 2007 to 2017 for studies that compared two or more strategies to increase retention in randomised trials, but did not use randomisation for allocation. The retention trials had to be nested in real 'host' trials.
Abstract and text screening was done in duplicate. Two investigators independently rated the risk of bias of included studies using the ROBINS-I tool and determined the certainty of evidence using GRADE.
**Results:** We identified 7609 abstracts and included 14 studies in the review. Most retention strategies were targeted at increasing questionnaire response rate rather than face-to-face appointments. Six strategies suggested a promising increase in questionnaire response rates: telephone follow-up compared to postal questionnaire completion; electronically-transferred monetary incentives compared to cash incentives; cash versus no incentive; reminders to non-responders; shortened versus longer questionnaires; online questionnaire follow-up compared to postal questionnaire [absolute increase in retention ranged from 10-40%]. However, each retention strategy was evaluated in a single observational study, which together with risk of bias concerns meant that GRADE certainty was low or very low for all included studies.
**Conclusions:** This systematic review provides low or very low certainty evidence on the effectiveness of retention strategies evaluated in non-randomised studies. Despite the uncertainty, some of the reported effect sizes were substantial and would remain large even if greatly reduced. Further evaluation in randomised studies (particularly telephone follow-up) would be helpful to provide a more certainty around the actual effect size.
**Keywords:** Clinical trials; Drop-outs; Non-randomised evaluations; Retention

## PS6A

### - O2 Exploring retention in clinical trials: A meta-ethnographic synthesis of studies reporting participant reasons for drop out

Katie Gillies, Zoe Skea, Rumana Newlands
Health Services Research Unit, University of Aberdeen, United Kingdom
*Trials* 2019, **20(Suppl 1):**PS6A

**Background:** Issues around retention, especially those issues reported by trial participants, have not received the same scrutiny in the literature as trial recruitment. This is a mistake. Poor retention is just as important for trial validity and is quite capable of fatally undermining a trial. Our aim was to undertake a meta-ethnographic synthesis of findings from primary qualitative studies that have explored factors influencing trial participant drop-out.
**Methods:** A systematic search of Embase, Ovid MEDLINE, PsycINFO, Cochrane CENTRAL, SSCI, CINAHL and ASSIA covering papers published from 1946 to August 2018 was conducted. Meta-ethnography was utilised to synthesise findings from eligible papers that contained qualitative data from trial non-retainers.
**Results:** We identified 11 studies reporting qualitative data from 13 trials. The studies were undertaken between 2008 and 2018. Each study included between 3 and 40 people who had dropped out from a trial, with findings from 168 people in total reported across the papers. Emergent from our synthesis was the significance of trial non-retainers' perceptions around the personal 'fit' of key aspects of the trial with their personal beliefs, preferences, capabilities or life circumstances. These related to their own health state; preferences for receiving trial 'care'; individual capabilities; beliefs about or experiences of trial medication; and considerations whether trial participation could be accommodated into their broader lives. All these factors raise important issues around the extent to which initial decisions to participate were fully informed.
**Conclusions:** To improve retention in clinical trials, researchers should work to reduce the burden on trial participants both through the design of the intervention itself as well as through simplified data collection processes. Providing more detail on the nature of the trial interventions and what can be expected by 'participation' at the consenting stage may prove helpful in order to manage expectations.

## PS6A

### - O3 Assessing non-adherence in non-inferiority trials: implications from a simulation study

Yin Mo[1,2,3], Cherry Lim[1,2], James Watson[1,2], Ben Cooper[1,2]
[1]Mahidol-Oxford Research Unit (MORU), Thailand; [2]Nuffield Department of Medicine, University of Oxford, Oxford, United Kingdom; [3]National University Hospital, Singapore
*Trials* 2019, **20(Suppl 1):**PS6A

Non-adherence in non-inferiority trials increases the risk of falsely claiming non-inferiority, enabling consecutively worse treatments to be accepted into clinical practice. We performed a simulation study to i) explore the impact of various patterns of non-adherence and analysis methods on trial estimates; ii) quantify the risk of falsely claiming non-inferiority (type I error) under reasonable assumptions in a typical non-inferiority trial, iii) propose alternative analysis methods, and vi) provide a tool for trial investigators to design non-inferiority trials using intuitive parameters. We simulated a hypothetical two-arm non-inferiority randomized controlled trial with a binary outcome and incorporated patient characteristics as confounders which may influence both the likelihood of taking up the allocated intervention and the primary outcome. Different scenarios of non-adherence, both random and driven by confounders, were considered. Using the intention-to-treat analysis, the trial estimates (given by treatment effect of the experimental treatment – treatment effect of the control treatment) drift towards 0 with increasing degree of non-adherence. The bias in the trial estimate derived by the per-protocol analysis increases with the strength of correlation between confounders and non-adherence behaviour. The risk of committing type I error can be as high as 10% even with relatively high levels of adherence (90%). Modified per-protocol analysis with inverse probability weighting was able to provide unbiased estimates provided that confounders are conditioned on. Instrumental variable estimation overcomes this limitation and gives unbiased estimates in all scenarios of non-adherence but requires a large sample size. We propose that trial investigators should consider the pattern and degree of expected non-adherence, causal relationships between the confounders and the outcomes, and the primary analysis method in power calculations during the planning stage of a non-inferiority trial. Modified per-protocol analysis with inverse probability weighting can be considered as a primary analysis method in non-inferiority trials with non-adherence.

## PS6A

### - O4 Statistical transparency in clinical trials: an evaluation of unexplained discrepancies between planned and conducted analyses

Brennan Kahan[1], Gordon Forbes[3], Tahania Ahmad[1], Nicholas Johnson[2], Suzie Cro[2]
[1]Pragmatic Clinical Trials Unit, Queen Mary University of London, United Kingdom; [2]Imperial Clinical Trials Unit, Imperial College London, United Kingdom; [3]Department of Biostatistics and health informatics, Institute of Psychiatry, Psychology & Neuroscience, Kings College London, United Kingdom
*Trials* 2019, **20(Suppl 1):**PS6A

**Introduction:** Results of clinical trials depend upon the statistical methods used for analysis. Modifications to the planned analysis approach can introduce bias if based upon trial data. Transparent and accurate reporting of planned and conducted analyses is necessary to allow for appropriate evaluation of methods.
**Methods:** We conducted two separate reviews of published randomised trials in order to evaluate transparency around the statistical methods. The first review included 101 trials published in six high-impact medical journals. The second included 100 trials published in all journals indexed in PubMed. We evaluated the number of trials with a publicly available pre-specified analysis approach for the primary outcome, how often changes to the pre-specified approach were made, whether reasons for changes were given, and information around the blinding status of trial statisticians in relation to data access.
**Results:** Across the two reviews we found that pre-specified analysis methods were often not publicly available, and when available were often dated after recruitment to the trial began. Most trials with an available pre-specified approach had discrepancies between the planned and conducted analysis, which were often not explained or justified. In many cases, incomplete reporting of statistical methods made it impossible to evaluate whether discrepancies occurred. Very few trials reported the blinding status of statisticians in relation to data access, prohibiting evaluation of whether changes to the statistical methods were made based on unblinded trial data.
**Discussion:** Investigators frequently made changes to their pre-specified analysis approach during the course of a trial. However, evaluation of whether such changes may have introduced bias was hampered by the limited availability of pre-specified analysis approaches, lack of explanation around the reasons for changes, and incomplete reporting of the statistical methods used.

## PS6A

### - O5 'Better healthcare through more inclusive research' – an NIHR workstream to improve trial delivery for underserved groups

Miles Witham[1], Lynn Rochester[1], Gary Nestor[1], Paul Dark[2], Kim Down[1], Alistair Hall[3], Helen Hancock[4], Joanna Knee[5], Rebecca Maier[4], Gail Mountain[6], Amanda Tortice[7], James Wason[8]
[1]NIHR Clinical Research Network Cluster E Specialty Team, Newcastle University, Newcastle, UK; [2]University of Manchester, Manchester, UK; [3]University of Leeds, Leeds, UK; [4]Newcastle Clinical Trials Unit, University of Newcastle, Newcastle, UK; [5]NIHR Clinical Research Network Coordinating Centre, University of Leeds, Leeds, UK; [6]Bradford University, Bradford, UK; [7]NIHR Clinical Research Network Yorkshire and Humber, Sheffield, UK; [8]Newcastle University, Newcastle, UK
*Trials* 2019, **20(Suppl 1):**PS6A

**Introduction:** Participants in clinical trials often do not reflect the populations in which treatments are needed or will be used. Enhancing representation of underserved groups in clinical research is important to ensure that research findings are widely applicable.
**Methods:** The project used a multicomponent workstream led by the UK NIHR Clinical Research Network Cluster E Specialty Team. We undertook: a) literature review by NIHR Innovation Observatory to identify previous work, underserved groups, and barriers to inclusion; b) surveys of professional stakeholders and participant representative groups involved in research delivery to refine these initial findings and identify example of innovation and good practice; and c) a series of workshops bringing together key stakeholders from funding, design, delivery and participant groups to reach consensus on definitions, barriers and a roadmap for future work.
**Results:** 'Underserved groups' was identified as the preferred term. Three-quarters of stakeholders felt that a clear definition did not currently exist; definition was challenging and context-specific but 17 exemplar groups were identified as underserved. Barriers to successful inclusion of underserved groups grouped into communication between research teams and participant groups; how trials are designed and organised; differing agendas of research teams and participant groups; and lack of trust in the research process. Examples of good practice included long-term engagement with participant groups, the use of co-design, research champions within underserved communities, and removal of unnecessary trial exclusion criteria and processes. The consensus for future work was to develop toolkits to address these barriers, including generic advice and advice targeted for those working with specific groups, and engaging researchers, funders, research users and others working with underserved groups.
**Conclusions:** The work of the group over the next 12 months will build on these findings by generating resources customised for different underserved groups to improve the representativeness of trial populations

## PS6B

### - O1 Covariate adjustment in individually randomised trials

Elizabeth Williamson[1,2], Clemence Leyrat[1], Karla Diaz-Ordaz[1]
[1]London School of Hygiene & Tropical Medicine, London, United Kingdom; [2]Health Data Research UK London, London, UK
*Trials* 2019, **20(Suppl 1):**PS6B

**Introduction:** While an unadjusted analysis of a randomised trial is unbiased, adjusting for measured pre-randomisation characteristics can increase statistical power. Guidelines regarding best practice for covariate adjustment in trials typically recommend adjusting for a small number of covariates, not including interactions of covariates with randomised arm, and the pre-specification of both the adjustment variables and the model.
These guidelines are often at odds with theoretical literature. Bias introduced by misspecifying an adjustment model in a trial can often be avoided by including interactions between covariates and trial arm. Promising new statistical approaches may not allow the pre-specification of the model or adjustment variables.
Whether and how to perform covariate adjustments in trials remains contentious.
**Methods:** Using simulations, we explore the statistical properties of a number of covariate adjustment methods in a range of settings based on real clinical trials. These include settings with few highly prognostic pre-specified adjustment variables, and those with large numbers of measured covariates that may be desirable to adjust for. We compare traditional covariate adjustment, inverse-probability-of-treatment-weighting, doubly robust methods, and targeted maximum likelihood estimation. We investigate continuous, count, binary, and time-to-event outcomes.
We illustrate key findings using the ViDiAs trial, a randomised controlled trial of vitamin D3 supplementation for the prevention of asthma exacerbation and URI. 250 adults were randomised to a course of vitamin D3 or placebo over a year. For URI, the primary analysis found no effect (Hazard Ratio 0.87, 95% confidence interval 0.62, 1.16, p=0.34), after adjusting for stratification factors. However, a number of other pre-randomisation variables showed imbalance between arms.
**Timing of Potential Results:** Simulation results will be finalised this summer.
**Potential Relevance & Impact:** The results of these simulations, and application to key exemplar trials, will provide practical guidance for trialists regarding how to plan and perform covariate adjustment in trials.

**PS6B**

**- O2 Practical choice of a method to account for baseline covariates in randomised trials**

Tim Morris, A Sarah Walker, Ian R White
MRC Clinical Trials Unit at UCL, London, United Kingdom
*Trials* 2019, **20(Suppl 1):**PS6B

**Introduction:** It is advisable to account for baseline covariates in the analysis of randomised trials for two reasons: 1) To increase power and 2) To obtain valid estimates of error when covariates have been balanced in the randomisation procedure. There are several different methods of accounting for covariates beyond simple covariate adjustment, and the choice of method is not straightforward outside standard linear regression models.
**Methods:** We work through how we choose a method at the point of writing a statistical analysis plan. Considerations include the outcome type (e.g. quantitative or binary), the possibility of non-convergence of adjusted models, and the estimand of interest (e.g. marginal risk ratio or conditional odds ratio, where conditional applies to specific covariate values and marginal is averaged across these). We illustrate the choices for each using the Vietnarms trial, considering three broad methods: regression adjustment, marginal standardisation and inverse-probability-of-treatment weighting (IPTW).
**Results:** When the estimand is a risk ratio or risk difference, regression adjustment carries a high risk of non-convergence and should not be used. Regression adjustment is also unsuitable for non-collapsible quantities when the estimand of interest is marginal. Marginal standardisation cannot currently be used to estimate a marginal hazard ratio, though methods are in development.
**Discussion:** Regression adjustment is suitable for estimation of conditional mean differences, conditional odds ratios and conditional hazard ratios. Surprisingly, IPTW seems to be the unifying method suitable for any marginal estimand, and is simple to implement. The disadvantage is that closed-form variance formulas are not available for hazard ratios, forcing a reliance on the bootstrap. Further work will include exploring further estimands, such as the difference in restricted mean survival time; considering more efficient methods; and developing the missing closed-form variance formulas.

**PS6B**

**- O3 Exploring mechanisms of action in clinical trials of complex interventions using mediation**

Linda Sharples[1], Saleema Rex[2], Olympia Papachristofi[3]
[1]London School of Hygiene and Tropical Medicine, London, United Kingdom; [2]University of York, York, United Kingdom; [3]Novartis A. G., Basel, Switzerland
*Trials* 2019, **20(Suppl 1):**PS6B

**Introduction:** Randomised trials are increasingly applied to complex interventions such as surgery and behavioural therapies. These interventions are characterised by two main features, multiple components and clustering of outcomes due to healthcare provider or group settings. Moreover, they are usually evaluated in open-label, pragmatic trials, allowing flexibility in delivery of the intervention, as well as differential use of co-interventions. Heterogeneity in the treatments delivered complicates evaluation but provides an opportunity to explore mechanisms of action.
Our aim is to demonstrate the use of mediation in this context, using the AMAZE trial as an illustration.
**Methods:** AMAZE was a trial of atrial fibrillation (AF)-surgery as an addition to planned cardiac surgery to treat irregular heart rhythm. The primary outcome (binary) was return to normal heart rhythm at 12 months. A binary co-intervention (removal of the left atrial appendage) was undertaken at the surgeon's discretion. Using logistic models with surgeon as a random effect, the contribution of this co-intervention to the total treatment effect was explored.
**Results:** Of the 280 patients with outcomes at 12 months post-surgery, 67/143 (47%) controls and 84/137 (61%) AF-surgery patients returned to normal heart rhythm. Left atrial appendage removal was more likely in AF-surgery patients than controls (55% versus 30%). Adjusting for baseline confounders, the difference in probability of return to normal rhythm (total effect) was 17% (8%, 27%), with average mediated effect of 13% (3%, 24%) and indirect effect via left atrial appendage removal of 4% (0.4%, 8%). Sensitivity to presence of unknown confounders was further assessed.
**Discussion:** Although heterogeneity of delivery complicates interpretation of complex interventions, it provides an opportunity to explore potential mechanisms of action in a quantitative (rather than qualitative) way. Interpretation must be cautious since methods require strong assumptions.

**PS6B**

**- O4 Quantifying bias of naive per-protocol (PP) versus intention-to-treat (ITT) analysis in randomised controlled trials: A meta-epidemiological study**

Mohammod Mostazir[1], Rod Taylor[2,3], Edward Watkins[1]
[1]College of Life and Environmental Sciences (CLES), University of Exeter, Exeter, England, United Kingdom; [2]University of Glasgow, Glasgow, Scotland, United Kingdom; [3]College of Medicine and Health, University of Exeter, Exeter, England, United Kingdom
*Trials* 2019, **20(Suppl 1):**PS6B

**Objective:** Intention-to-treat (ITT) is the recommended statistical method for analysing randomized control trials (RCTs). However, given that trial participants often do not fully adhere to the treatment protocol, research teams often also report a per-protocol (PP) analysis. Naive PP (i.e. comparing participants who achieved a minimum level of treatment with control participants) is known to break random allocation and may therefore result in a biased treatment effect estimate. Nevertheless, the level of bias associated with the naïve PP method is not well understood. The aim of this meta-epidemiological study is to quantify this bias.
**Methods:** We will identify RCTs (published between April, 2017 and March, 2019) across 5 major journals (Lancet, NEJM, BMJ, JAMA, Annals of Internal Medicine) that report both ITT and PP estimates for the primary outcome. Our pilot of 25 trials shows that we require 79 RCTs to detect ≥6% difference between ITT and PP at 90% power. Given that we are pooling RCTs across populations and treatments, we will use random effects meta-analysis to compare the ITT and PP treatment estimates reported for the primary outcome. Treatment effect across studies will be converted to the common metric of log of odds ratio (OR).
**Timing of potential results:** Our pilot study of 25 trials indicate that PP estimates were an average 4% higher than ITT estimates (OR: 1.04, 95% CI: 0.94 to 1.06, p = 0.14). The full study is ongoing and results will be ready before September, 2019.
**Potential relevance and impact:** Treatment non-adherence is a problem that besets virtually all RCTs and, as result, the reporting of treatment estimate using ITT alone may not be completely informative. This study seeks to quantify the level of bias associated with naïve PP method and whether the PP reporting is a useful supplement alongside the traditional ITT estimate.

**PS6B**

**- O5 Misinterpretation of factorial design trials and inappropriate meta-analysis: misleading the reader**

Tim Clayton[1], Kristin Veighey[2], Jennifer Nicholas[1]
[1]London School of Hygiene & Tropical Medicine, London, United Kingdom; [2]Wessex Kidney Centre, Portsmouth, United Kingdom
*Trials* 2019, **20(Suppl 1):**PS6B

Factorial designs are an under-used but potentially important design in the evaluation of more than one medical intervention within a single trial. However, the design of such trials is often misunderstood and the analysis, reporting and interpretation inappropriate.
Systematic reviews and meta-analyses, at their best, can be a vital tool to bring together the worldwide evidence from randomised trials assessing the impact of particular treatments or interventions. However, the data are often poorly understood and results misrepresented and misinterpreted, for example, through the handling of missing data and the misuse of random effects meta-analysis. This can lead to delays in effective treatments for patients and can be a barrier to further research opportunities.
These issues will be illustrated through the example of the factorial design REPAIR trial and subsequently published meta-analysis. The REPAIR trial evaluated the impact of remote ischaemic pre-conditioning in patients undergoing living donor kidney transplantation indicating improved short and long-term effects on estimated glomerular filtration rate. The meta-analysis used data that were extracted and reported incorrectly and despite subsequent changes the published report remains inadequate. This is due mainly to the misunderstanding of the factorial design and the subsequent misrepresentation of the results through the use of random effects meta-analysis. This has led to a potentially effective intervention being denied to patients, delays in publication of long-term REPAIR results, and prevented further research in kidney transplantation. The trial design and published results from REPAIR will be presented together with the reported results from the meta-analysis to allow informed conclusions to be made. In addition the 5 year outcomes from REPAIR will be presented.
We encourage measures to improve the acceptance and understanding of factorial trials as well as caution in over-interpreting the results from random effects meta-analyses from randomised trials.

## PS6C
### - O1 Paper versus electronic completion of patient reported outcomes: What do we know?

Kirsteen Goodman[1], Suzanne Hagen[1], Lynn Melone[1], Melanie Dembinsky[2], Rohna Kearney[3], Andrew Elders[1], Carol Bugge[2]
[1]NMAHP Research Unit, Govan Mbeki building, Level 6, Glasgow Caledonian University, Cowcaddens Road, Glasgow, G4 0BA, Glasgow, United Kingdom; [2]Faculty of Health Sciences and Sport, University of Stirling, FK9 4LA, Stirling, United Kingdom; [3]The Warrell Unit, St. Mary's Hospital, Manchester University Hospitals NHS Foundation Trust, Manchester Academic Health Science Centre, Manchester, M13 9WL, United Kingdom; University Institute of Human Development, Faculty of Medical Human Sciences, University of Manchester, United Kingdom., Manchester, United Kingdom
*Trials* 2019, **20(Suppl 1):**PS6C

**Introduction:** Patient Reported Outcome Measures (PROMs) are a widely used method of collecting both primary and secondary outcome data. Completion of PROMs can be via the traditional paper questionnaire or online via a link which directs the participant to an electronic version of the questionnaire that they can complete via their smart phone /computer.
"What are the most effective ways of collecting information from participants to increase retention?" has just been published as item 7 in the top 10 priorities for research into trial retention. We explored this by giving participants the option to choose between paper and electronic methods in two trials.
**Methods:** In two large multicentre trials (TOPSY and OPAL), participants (women with prolapse and urinary incontinence respectively) were given the option of completing their follow-up questionnaires via paper or online. We measured;
1.What proportion of participants prefer each method?
2.Is the method of completion influenced by socio-demographic characteristics?
3.Is there a difference in a) the return and b) the completion rate for the two methods?
**Timing of potential Results:** The OPAL trial is complete and the TOPSY trial is ongoing (complete mid 2021). We will present data on participant preference for mode of completion; differences for participants with different socio-demographic characteristics for the different modes of completion; percent of paper and email return rates and extent of completion at different time points overall and for different sociodemographic characteristics.
**Potential relevance and Impact:** The results have the potential to influence future trial design by informing researchers about likely benefits, and potential drawbacks, of different methods of questionnaire administration for different participants. There is also the potential to set up one or more SWATs to collaborate with other trialists to prospectively gather evidence to answer item 7 in the top 10 priorities for retention research.

## PS6C
### - O2 Paper diary capture vs. electronic data capture for patient reported outcomes in Primary Care: an investigation into completion rates

Jenna Grabey, Johanna Cook, Rajendra Raghuraman
University Of Oxford, United Kingdom
*Trials* 2019, **20(Suppl 1):**PS6C

**Introduction:** Patient reported outcomes (PROs) are used to evaluate the effectiveness of an intervention by the patient measuring their own health and quality of life. It is not uncommon for PROs to be used for primary outcome data in primary care clinical trials or studies. From previous research it is not clear whether paper data capture (pDC) or electronic data capture (eDC) is most efficient when it comes to completeness of data. Some say adherence to pDC is higher (Blondin et al., 2010) and others that adherence to eDC is higher (Hufford et al., 2002). Previous studies have focused on time efficiency, error rate and cost. This study will investigate the comparative completion rates of PROs from pDC and eDC (forms completed via computer/mobile by patient).
**Methods:** Completed studies are included in this project if they meet the following inclusion criteria: (1)randomised clinical trial or observational study; (2)Primary Care Clinical Trials Unit (PC-CTU), University of Oxford involved in study management; (3)data management carried out by PC-CTU; (4)both pDC and eDC options were available for PROs.
**Analysis:** The first analysis will compare the number of forms per trial that were completed via pDC or eDC. The second analysis will sum the number of required missing fields, these will then be compared within the trial (pDC vs. eDC) and across the selected trials using tests of significance (t-test, ANOVA). We will also perform secondary analyses looking at (1)age range (2)number of times participant asked to complete a PRO and (3)whether or not reminders were sent to participants.
**Timing of potential results:** August 2019.
**Potential relevance and impact:** The outcome of this study will help to inform which method of data capture will provide the highest completion rates for collecting PROs.

## PS6C
### - O3 A machine learning algorithm and tools for automatic detection of spin (distorted presentation of results) in articles reporting randomized controlled trials

Anna Koroleva[1,2], Patrick Paroubek[1]
[1]LIMSI, CNRS, Université Paris-Saclay, Orsay, France; [2]Academic Medical Center, University of Amsterdam, Amsterdam, Netherlands
*Trials* 2019, **20(Suppl 1):**PS6C

**Introduction:** Spin (distorted reporting of research results) consists in presenting research results as being more positive or significant than proved by experiments. In randomized controlled trials (RCTs), spin consists in exaggerating the beneficial effects (efficacy, safety) of the studied intervention. Spin results in overestimation of the intervention by clinicians and induces unjustified positive presentation of the intervention in press releases and health news.
Recent studies (2016-2019) showed that spin is prevalent in articles on RCTs with non-significant primary outcome in various domains, e.g. surgery (40%), cancer (47%), obesity (46.7%), otolaryngology (70%), anaesthesiology (32,2%) and wound care (71%). Spin often remains unnoticed by peer reviewers and can be unintentional (arising from benevolent desire to present the most important results). Thus, our aim is to provide spin detection assistance to authors and reviewers.
**Methods:** We propose a machine learning algorithms and tools for spin detection developed in collaboration with experts in clinical trials. It uses Natural Language Processing (NLP) and machine learning (BERT neural embeddings) to extract spin-related items (primary and reported outcomes, patient population, significance levels) and relations between them to detect potential spin. The system flags the phenomena of interest in the text and generates a report. It was evaluated on manually annotated corpora. The tool set includes a spin detector in Python and a graphic interface (TkInter library) to annotate new training data.
**Results:** Our algorithms achieved operational performance for detecting relevant phenomena (F-measure from 76,2 to 97.8%). The most difficult task is extracting reported outcomes, where our system achieves an F-Measure of 76,2%, outperforming existing algorithms.
**Discussion:** The proposed tool can be used by both authors and reviewers to detect potential spin, helping to improve the quality of research results reporting. The tool and the annotated dataset will be freely available.

## PS6C
### - O4 The use of regular text messaging over one year to collect primary outcome data in a randomised controlled trial

Kieran James Bromley[1,2], Reuben Ogollah[3], Kika Konstantinou[1,4], Nadine Foster[1,2], Martyn Lewis[1,2]
[1]School of Primary, Community & Social Care, Keele University, United Kingdom; [2]Keele Clincal Trials Unit, Keele University, United Kingdom; [3]Nottingham Clinical Trials Unit, University of Nottingham, United Kingdom; [4]Haywood Hospital, Midlands Partnership NHS Foundation Trust, Stoke-on-Trent, United Kingdom
*Trials* 2019, **20(Suppl 1):**PS6C

**Introduction:** Frequent follow-up data collection may be desirable in RCTs as it allows greater longitudinal assessment, and increased power and accuracy by reducing attrition bias, which undermines the intention-to-treat (ITT) principle. Short message service (SMS) using mobile telephones offers an alternative way to enhance outcome data collection to postal, online, or telephone-call follow-up processes.
**Methods:** Data from an RCT (SCOPiC trial ISRCTN75449581) evaluating stratified care in patients with sciatica in primary care were used to examine the rate of responses for primary outcome data collected largely through SMS over 12 months.
**Results:** Data were received and evaluated from 476 trial participants. The primary outcome was time to resolution of sciatica symptoms from baseline, assessed on a 6-point ordinal scale collected using SMS (with reminder SMS processes and an alternative of brief phone calls). Data were collected weekly for weeks 1-16, then either monthly for weeks 17-48 or until two consecutive responses of 'completely recovered'/'much better' were received. In total, 426/476 (89.5%) of participants opted for SMS follow-up. Overall response was 89.3% (9467 responses from 10,601 attempts); 90.2% via SMS and 81.9% via phone-call. Response rate was higher over the earlier 'weekly' period at 93.9% than for the later 'monthly' period at 84.7%. SMS choice (versus phone-call) was significantly associated with lower age (mean, 50.5 years v 65.2) and currently working (73% v 35%). Participants with incomplete follow-up lived in significantly more deprived neighbourhoods. There were no strong associations with baseline and follow-up health status variables.
**Discussion:** Within this low back pain population in primary care (and likely more broadly), SMS follow-up of key outcome data can be successful, particularly when targeting younger and/or working populations. It offers an accessible and robust way of enhancing follow-up, improving the quality of statistical analyses and reducing data collection costs.

## PS6C
### - O5 Feasibility of collecting digital images of surgical wounds taken by patients themselves after leaving hospital: a method for remote and blinded outcome assessment (The Selfi wound study)

Rhiannon Claire Macefield[1], Kerry NL Avery[1], Jonathan RE Rees[1,2], Anne Pullyblank[3], Barnaby Reeves[4], Anni Skilton[1], Jane M Blazeby[1]
[1]Population Health Sciences, Bristol Medical School, University of Bristol, Bristol, United Kingdom; [2]The Division of Surgery, Head & Neck, University Hospitals Bristol NHS Foundation Trust, Bristol, United Kingdom; [3]General Surgery, North Bristol NHS Trust, Bristol, United Kingdom; [4]Clinical Trials and Evaluation Unit, Bristol Trials Centre, Bristol Medical School, University of Bristol, Bristol, United Kingdom
*Trials* 2019, **20(Suppl 1):**PS6C

**Introduction:** Advances in technology mean the collection of digital images of wounds taken by patients themselves after leaving hospital is increasingly more accessible. Images may supplement patient-reported data and facilitate research and routine follow-up (e.g. remote, blinded assessment of problems/healing).
The Selfi wound study (self-taken images of surgical wounds) aimed to develop and pilot a method for collecting images of wounds after patients leave hospital following surgery, and explore its potential use for remote outcome assessment of wounds.
**Methods:** Existing guidelines for wound photography (e.g. medical illustration documents, study protocols/manuals) informed instructions for patients to take images using their own mobile devices. Cognitive interviews (n=16 patients) were conducted to pre-test and refine the instructions and test a system for transmitting images to a study database. A further group (n=61 patients) undergoing abdominal or vascular surgery tested the method remotely, selectively sampled for a range of ages, surgery types and wound locations. Data were collected on experience with technology, time taken to photograph wounds and whether help was needed. Response rates, participant burden, and image quality were examined.
**Results:** Key considerations for photographing wounds (e.g. lighting, camera angle) informed provisional instructions for patients. Cognitive interviews demonstrated acceptability and capability for taking and uploading images, with some modifications to the instructions required. Images were received from 34/61 (56%) patients testing the method remotely with 14 (41%) needing a reminder. Median time to respond was four days (range 0 to 24). Photographing wounds took <5 minutes for the majority (84%) of responders. Images were predominantly clear and of suitable quality for assessing the wound.
**Conclusions:** Remote collection of digital images of surgical wounds from patients using their own mobile devices after leaving hospital is feasible, practical and acceptable to patients. Further evaluation of the method for facilitating outcome assessment in trials is planned.

## PS7A
### - O1
**Abstract omitted**

## PS7A
### - O2 Using systematic data categorisation to quantify the types of data collected in clinical trials

Evelyn Crowley[2], Gordon Fernie[1], Katie Banister[3], Suzanne Breeman[1], Anne Duncan[1], Lynda Constable[1], Adel El Feky[3], Heidi Gardner[3], Kirsteen Goodman[4], Doris Lanz[5], Alison Mcdonald[1], Emma Ogburn[6], Natasha Stevens[7], Marie Valente[8], Shaun Treweek[3]
[1]Centre for Healthcare Randomised Trials, Health Services Research Unit, University of Aberdeen, Aberdeen, United Kingdom; [2]HRB Clinical Research Facility, University College Cork, Cork, Republic of Ireland; [3]Health Services Research Unit, University of Aberdeen, Aberdeen, United Kingdom; [4]NMAHP Research Unit, Glasgow Caledonian University, Glasgow, United Kingdom; [5]Centre for Primary Care and Public Health, Barts and the London School of Medicine and Dentistry, Queen Mary University Of London, London, United Kingdom; [6]Primary Care Clinical Trials Unit, University of Oxford, Oxford, United Kingdom; [7]Queen Mary University Of London, London, United Kingdom; [8]Birmingham Clinical Trials Unit, University of Birmingham, Birmingham, United Kingdom
*Trials* 2019, **20(Suppl 1):**PS7A

**Introduction:** Data collection consumes a large proportion of trial resources. Each data item requires time and effort for collection, processing and quality control procedures. Generally speaking, more data equals a heavier burden for trial staff and participants. It also increases the cost of the trial. Data is generally collected for 3 broad reasons:
•To answer the main research question (a primary outcome is specified and drives sample size calculations).
•Secondary outcomes to supplement the primary outcome.
•Additional data to monitor safety, maintain quality and for regulatory and data management needs.
Here we report the results of a collaborative Trial Forge project which measured the proportion of data fitting these three broad categories, across 18 trials run from 5 institutions in Ireland and the UK.
**Methods:** We developed a standard operating procedure to categorise data. We categorised all variables collected on trial data collection forms from 18, mainly publically-funded Randomised Controlled Trials, including clinical trials of an investigational medicinal product and surgical trials. Categorisation was done independently in pairs: one person having in-depth knowledge of the trial, the other independent of the trial. Disagreement was resolved through reference to the trial protocol and discussion, with the project team being consulted if necessary.
**Results:** Primary outcome data accounted for 11.2% (mean) and 5% (median) of all data items collected. Secondary outcomes constituted a mean of 42.5% (median: 39.9%) of data items. Non-outcome data represented a mean of 36.5% (median: 32.4%) of data items collected.
**Discussion:** Our study highlights the proportion of data collected to answer the main research question is minimal in comparison with other data collected, and that much of this is non-outcome data. We discuss implications including whether such data collection is excessive or has detrimental effects on a trial.

## PS7A
### - O3 Do RCTs reflect patient populations and does it matter? Considerations and a case study

Mike Bradburn[1], Ellen Lee[1], David White[1], Daniel Hind[1], Simon Heller[2]
[1]Clinical Trials Research Unit, University Of Sheffield, Sheffield, United Kingdom; [2]Department of Oncology and Metabolism, University of Sheffield, Sheffield, United Kingdom
*Trials* 2019, **20(Suppl 1):**PS7A

**Introduction:** RCTs have been criticised for lacking external validity. A sizeable body of meta-epidemiological evidence has shown RCT participants can often differ from wider patient populations, either through entry criteria restrictions or through selective uptake ("volunteer bias"). RCTs may struggle to convince a clinical community of their merit if they do not represent the patients they themselves see. We assessed whether a trial in type I diabetes mellitus (T1DM) mirrored the wider patient population, and applied sample-weighting methods to derive a treatment effect projected onto a more representative T1DM population. We describe how to apply these methods, their limitations, and their impact on our trial's findings.
**Methods:** The REPOSE (Relative Effectiveness of insulin Pump Over MDI and Structured Education) trial was nested within a large UK-based cohort of patients with T1DM. The database captured detailed demographic, clinical and QoL data for T1DM patients undergoing structured diabetes-specific education. We firstly assessed whether our RCT participants were comparable to this cohort using propensity score modelling. Following this we re-weighted the trial population to better match the wider cohort, and re-estimated the treatment effect from this.
**Results:** Our trial patients differed from those of the cohort in regards to sex, weight, HbA1c and also QoL and satisfaction with current treatment. Nevertheless, the treatment effects derived from alternative model weightings were similar to that of the original RCT.
**Discussion:** We found our RCT recruited a non-random set of participants but that the main results were unaffected by re-weighting. We advocate researchers to take steps to address criticisms of generalisability, including these analyses. Doing so is nevertheless problematic: external data is difficult to obtain and may contain information is too limited to make informative adjustments. Analyses can be susceptible to model misspecification, especially in smaller trials.

## PS7A
### - O4
### Abstract omitted

## PS7A
### - O5 Rewards and challenges of undertaking health-related research within the UK Police setting

Alison Booth[1], Catriona McDaid[1], Arabella Scantlebury[1], Adwoa Parker[1], Caroline Fairhurst[1], Julie Parkes[2], Sara Morgan[2], Benjamin Taylor[3], Caroline Chapman[3], Catherine Hewitt[1], David Torgerson[1]
[1]University of York, York, United Kingdom; [2]University of Southampton, Southampton, United Kingdom; [3]Hampshire Constabulary, Southampton, United Kingdom
*Trials* 2019, **20(Suppl 1):**PS7A

**Introduction:** York Trials Unit (YTU) has gained valuable experience through working with the Police. We present here key learning outcomes from a co-production project in mental health, attempts to set up a trial related to speeding offences and a trial of a youth offending intervention.
**Methods:** YTU's experience comes from:
Co-production with North Yorkshire Police (NYP) of a series of systematic reviews to improve the evidence base related to mental health, and an RCT of training for police officers to improve their handling of situations where members of the public they are in contact with are experiencing mental ill health.
Developing a proposal for a trial with the Traffic division of NYP to improve the response rate to conditional offers for speeding offences.
An on-going NIHR funded project with the University of Southampton and Hampshire Constabulary to undertake an RCT, with economic and qualitative evaluation of Gateway, an out-of-court, community-based intervention aimed at improving life chances for 18-24 year old offenders and reducing reoffending.
**Results:** Four systematic reviews were completed and the cluster RCT involving 12 police stations and 249 officers receiving the bespoke training, showed it may have a positive effect on recording of incidents.

The trial related to speeding offences was not feasible as there was no way of linking data between two NPY information systems.
Recruitment to the Gateway trial has been delayed for various reasons.
**Discussion:** Police Forces are enthusiastic collaborators and keen to underpin their working practices with robust evidence. Conducting pragmatic, health related trials within the police setting is feasible and acceptable. However, even simple evaluations need to be designed with care to fit in with existing work practices, in particular Police IT systems, the Police legal framework, and the rapidly evolving political and social environment. Close collaboration between police and academia is key.

## PS7B
### - O1 Using Bayesian adaptive designs to improve phase III randomised controlled trials

Elizabeth Gabrielle Ryan, Simon Gates
Cancer Research UK Clinical Trials Unit, University of Birmingham, United Kingdom
*Trials* 2019, **20(Suppl 1):**PS7B

**Introduction:** Bayesian adaptive designs can improve the efficiency of trials, and can produce high quality evidence more quickly, with fewer patients and lower costs than traditional methods. The aim of this work was to determine how Bayesian adaptive designs can be constructed for multi-arm phase III clinical trials, and to assess the influence that Bayesian designs would have on trial efficiency and study results.
**Methods:** We re-designed the Collaborative Ankle Support Trial (CAST) using Bayesian adaptive design methods, to allow for the possibility of response adaptive randomisation (RAR), arm dropping, and early stopping for efficacy or futility. We constructed several alternative Bayesian designs and studied their operating characteristics via simulation. We then virtually re-executed the trial by implementing the Bayesian adaptive designs using the CAST data to demonstrate the practical applicability of the designs.
**Results:** We constructed five alternative Bayesian adaptive designs, each of which had high power and recruited fewer patients on average than the original design. The virtual executions showed the Bayesian adaptive designs with RAR and/or arm dropping allocated more patients to better performing arms, but did not stop the trial early for efficacy or futility.
**Discussion:** Researchers and funders have recognised the need for trials to become more efficient, yet the overwhelming majority of trials continue to use traditional methods, particularly with fixed designs. Whilst Bayesian adaptive designs have proved to be popular for early phase studies, their use in phase III trials remains limited. Using the CAST trial as an example, this case study found that Bayesian adaptive designs can be constructed for phase III multi-arm trials using clinically relevant decision criteria. These designs demonstrated that they can potentially generate earlier results and allocate more patients to better-performing arms. We recommend the wider use of Bayesian adaptive approaches in phase III clinical trials.

## PS7B
### - O2 Designing trials for small populations

Victoria Cornelius, Suzie Cro
Imperial Clinical Trials Unit, Imperial College, United Kingdom
*Trials* 2019, **20(Suppl 1):**PS7B

**Introduction:** A key design challenge for trials in rare diseases and subgroups of special interest is a restricted sample size. Standard frequentist-based approaches to power trials based on large sample theory are not always suitable. To obtain robust high-quality evidence alternative approaches are required. This challenge has recently come to the forefront and has motivated new activity on how best to design and evaluate treatments when sample size is restricted. A bold framework adopting a pragmatic approach to designing trials for small populations was proposed by Parma et.al. 2016. More recently recommendations have been produced by the International Rare Diseases Research Consortium Small Population Clinical Trials Task Force, and the FDA with guidance for adaptive designs. Methods for specific acceptable approaches are lacking.
**Methods:** We present the development and design of two NIHR funded small population trials. The evaluation of a new biological drug for pustular psoriasis, a rare and debilitating condition in adults, and a new treatment option for paediatric severe treatment resistant asthma (STRA).
**Results:** No validated measure of pustular psoriasis exists and there was minimal safety data for the drug in this population. A novel two-stage adaptive placebo-controlled trial was designed to evaluate two potential disease measures for the primary outcome, check proof of concept and safety in stage one. Efficacy assessment, requiring a sample size of 64 (90% power, two-sided 5% significance level), occurs in stage two.
In STRA, placebo was not allowed for ethical reasons. With a fixed sample size of 150 a Bayesian approach with an informative prior distribution will be used to assess non-inferiority of treatment. The predicted probability of non-inferiority demonstrates the value of undertaking this study.
**Discussion:** Novel and bold approaches can be used to design trials that will provide robust randomised evidence which would not be possible using standard approaches.

## PS7B
### - O3 Multi-arm multi-stage designs with fixed stage-wise sample sizes

Michael Grayling[1], James Wason[1,2]
[1]Newcastle University, Newcastle upon Tyne, United Kingdom; [2]MRC Biostatistics Unit, University of Cambridge, Cambridge, United Kingdom
*Trials* 2019, **20(Suppl 1):**PS7B

**Introduction:** Several sequential multi-arm trial designs have now been presented. This includes multi-arm multi-stage (MAMS) designs that allow futile treatments to be dropped, or efficacious treatments to be identified, at each interim analysis. Whilst this approach can be highly efficient, the actual sample size required by a MAMS design is difficult to predict. Consequently, a multi-stage drop-the-losers design, with a fixed total required sample size, was recently described. In many scenarios though, this design will be less efficient than its MAMS analogue. Here, we present a possible compromise between these approaches; a class of MAMS designs with fixed stage-wise sample sizes.
**Methods:** We examine MAMS designs in which the sample size allocated to each arm, in each stage, is dependent on the number of active arms. In particular, the operating characteristics of our new designs are contrasted to a variety of possible sequential multi-arm designs, using design parameters motivated by oncology, HIV, and orthostatic hypotension MAMS trials. To facilitate the use of such designs in practice, we also present an online web application for identifying sequential multi-arm designs.
**Results:** In many instances, at a small cost to the expected sample size, the variation in the sample size required by our novel MAMS designs may be easier to handle in practice. A loss function can be utilised to choose between the available approaches. The presented web application is able to contrast each of the designs in an efficient manner.
**Conclusions:** The design used by a sequential multi-arm clinical trial should be chosen carefully. In particular, key expected sample sizes and possible variations in the required sample size should be taken in to consideration at the design stage. Fixing the stage-wise sample size may offer a compromise between conventional MAMS and drop-the-losers designs.

## PS7B

### - O4 Investigating the application of a multi-arm, multi-stage (MAMS) design to compare optimal treatment duration of Herceptin in a non-inferior setting in treating early breast cancer patients

Pankaj Mistry, Janet A Dunn, Louise Hiller, Andrea Marshall
University Of Warwick, Coventry, United Kingdom
*Trials* 2019, **20(Suppl 1):**PS7B

**Introduction:** Implementing multi-arm, multi-stage (MAMS) designs have proven to be efficient and effective for assessing new treatments with survival outcomes. MAMS designs are yet to be applied in studies assessing different durations of treatment. This simulation study aimed to evaluate the efficiency and effectiveness of a MAMS design for testing the optimal duration of Herceptin in treating early breast cancer.
**Methods:** Non-inferiority (NI) properties were explored in six Herceptin duration trials. Simulations were used to assess performance of implementing a four arm three stage MAMS NI trial with 12 months of Herceptin as the control arm (standard care) versus experimental arms of six months, three months and nine weeks of Herceptin. Intermediate and primary outcomes, ranges of NI margins and survival outcome rates based on trials were investigated.
**Results:** Simulations showed that implementation of a four arm three stage MAMS design is more efficient than performing each trial separately as the required sample size and duration of the study was reduced. The most efficient and effective MAMS design proved to be when five year disease-free survival of 81% was used as the intermediate and primary outcome with a 3% NI margin. Emphasis was placed on ensuring that the family-wise error rate (FWER) remained below 5%.
**Conclusion:** A MAMS design should be considered when implementing a new therapy into a disease area as it allows different treatment duration to be explored as well as the efficacy of the treatment. Simulations showed that a MAMS design for the Herceptin duration question in early breast cancer would have identified the optimal treatment duration quicker.
Implementation of MAMS designs in practice requires input from clinical stakeholders to determine the appropriate timing for randomisation and the minimum amount of follow-up required once patients have completed the course of treatment before performing an interim analysis.

## PS7B

### - O5 Experiences of setting up Trials within Cohort Studies: Overcoming challenges and maximising efficiency – a case study

Ines Rombach[1,2,3], Marion Watson[1,2,3], Yvonne Sinomati[1,2,3], Laura Coates[3]
[1]Oxford Clinical Trials Unit, Oxford, UK; [2]Centre For Statistics In Medicine, Oxford, UK; [3]Nuffield Department of Orthopaedics, Rheumatology and Musculoskeletal Science, Oxford, UK
*Trials* 2019, **20(Suppl 1):**PS7B

**Background:** The Trials within Cohorts (TWiCs) recruit trial participants from an existing cohort. This pragmatic design allows robust generalizability to routine healthcare, avoids disappointment bias, aids recruitment and assessment of longer-term outcomes.
Despite their theoretical advantages, implementation and conduct can be demanding. Here, we share our experiences of setting up our first TWICs.
**Methods:** MONITOR is a multicentre TWiCs cohort assessing the effectiveness of a treat-to-target approach in psoriatic arthritis. Currently, two RCTs are set up within our cohort study.
**Results:** Detailed explanations of methodology were essential for approvals by funders, sponsors and regulators unfamiliar with this novel design. Separate protocols were required for the cohort and each RCT. While this resulted in duplication, increased administration and additional approval processes, it allows for set-up and amendment of individual trials while the cohort continues.
The use of routinely collected data ensures the efficient collection of high-quality data. However, it necessitated the use of separate databases, resulting in additional programming to collate data, and more complex data checks.
Our TWICS design required a two-stage eligibility check for randomisation, and additional safety reviews for the interventional arms only. Close collaboration with our programming team allowed us to adapt existing systems and minimise patient burden.
Safety reports are identical for all included RCTs, and can therefore automated easily and will be presented to a joint committee, reducing workload and the number of independent oversight committee members required.
Statistical considerations include the potential for differential take-up of the intervention between the trial arms, and the potential for differential missing data rates.
**Discussion:** TWICS can be challenging to set-up, and require careful planning involving all trial team members and relevant external agencies. When implemented successfully, they are a very efficient design to facilitate a multitude of trials in a specific patient population.

**Funding statement**

Dr Laura Coates is funded by a National Institute for Health Research (NIHR) Clinician Scientist Award for this research project. This presentation presents independent research funded by the National Institute for Health Research (NIHR). The views expressed are those of the authors and not necessarily those of the NHS, the NIHR or the Department of Health and Social Care.

## PS7C

### - O1 Outcome assessment by central adjudicators versus site investigators in randomised stroke trials: A systematic review and meta-analysis

Peter J Godolphin[1], Philip M Bath[2], Alan A Montgomery[1], NA on behalf of the Adjudicating Outcomes in Stroke Trials Collaboration NA[3]
[1]Nottingham Clinical Trials Unit, University of Nottingham, Nottingham, United Kingdom; [2]Division of Clinical Neuroscience, University of Nottingham, Nottingham, United Kingdom; [3]No affiliation, group authorship, NA, NA
*Trials* 2019, **20(Suppl 1):**PS7C

**Background:** In randomised stroke trials, central adjudication of a trial's primary outcome is regularly implemented. However, recent evidence questions the importance of central adjudication in randomised trials. The aim of this review was to compare outcomes assessed by central adjudicators with outcomes assessed by site investigators.
**Methods:** We included randomised stroke trials where the primary outcome had undergone assessment by site investigators and central adjudicators. We searched MEDLINE, EMBASE, the Cochrane Central Register of Controlled Trials, Web of Science, PsycINFO and Google Scholar for eligible studies. We extracted information about the adjudication process as well as the treatment effect for the primary outcome, assessed both by central adjudicators and by site investigators. We calculated the ratio of these treatment effects (RTE) so that an RTE > 1 indicated that central adjudication resulted in a more beneficial treatment effect than assessment by site investigator. A random-effects meta-analysis model was fitted to estimate a pooled effect.
**Results:** Fifteen trials including 69,560 participants were included. The primary outcomes included were stroke (8/15, 53%), a composite event including stroke (6/15, 40%) and functional outcome after stroke measured on the modified Rankin Scale (1/15, 7%). The majority of site investigators were blind to treatment allocation (9/15, 60%). On average, there was no difference in treatment effect estimates based on data from central adjudicators and site investigators (pooled RTE=1.02, 95% C.I: [0.95, 1.09]).
**Discussion:** We found no evidence that central adjudication of the primary outcome in stroke trials had any impact on trial conclusions. This suggests that potential advantages of central adjudication may not outweigh cost and time disadvantages in stroke studies if the primary purpose of adjudication is to ensure validity of trial findings.

## PS7C

**- O2**

**Abstract omitted**

## PS7C

**- O3 Increasing the trial process evidence base without increasing research waste**

Shaun Treweek, on behalf of the Trial Forge initiative
University Of Aberdeen, United Kingdom
*Trials* 2019, **20(Suppl 1):**PS7C

**Introduction:** The evidence available to inform trial process decisions is thin. This leads to research waste. One way of improving the evidence base is to evaluate trial process alternatives in a Study Within A Trial (SWAT). SWATs are gaining traction, especially in the UK and Ireland, with SWAT funding streams from the National Institute for Health Research (NIHR) and the Health Research Board (HRB) in Ireland.
**The problem ahead:** NIHR, HRB and others see a problem ahead: how do we avoid contributing to research waste ourselves by funding or doing SWATs that have, in fact, already answered their questions?
**Developing criteria to make sensible judgements:** Trial Forge (https://www.trialforge.org) brought together a group of 28 trialists, methodologists, clinicians, patients, research funders and research governance staff from the UK, Ireland and Switzerland to develop a set of criteria to make decisions about when doing another evaluation of a SWAT is needed.
I will present our five criteria. The criteria consider the evidence coming from the cumulative meta-analysis of all evaluations of the SWAT, the certainty we have in that evidence judged using GRADE, the contexts in which the SWAT has been evaluated, and the balance of benefit and disadvantages for a) participants and b) the host clinical trial. I will present worked examples of how the criteria apply to two SWATs, one in recruitment, one in retention. I will also discuss how these criteria can be used to not only make decisions about whether a SWAT needs further evaluation but to guide the selection of the types of trials in which to embed future SWAT evaluation.
**Conclusion:** To avoid research waste, decisions about which SWATs to evaluate, how and where needs a coordinated and structured approach. The criteria we have developed are the start of such an approach.

## PS8A

**- O1 Staff training to improve participant recruitment into surgical randomised controlled trials: a feasibility study embedded within four randomised controlled trials**

Adwoa Parker[1], Nicola Mills[2], Leila Rooshenas[2], Marcus Jepson[2], Jenny L. Donovan[2], Catherine Arundel[1], Puvanendran Tharmanathan[1], Elizabeth Coleman[1], Catherine Hewitt[1], Prasanna Partha Sarathy[6], David Beard[7], Peter Bower[8], Paul Brocklehurst[9], Cindy Cooper[10], Lucy Culliford[2], Joseph Dias[11], Declan Devane[12], Sandra Eldridge[13], Richard Emsley[14], Sandra Galvin[12], Alan Montgomery[15], Chris Sutton[8], Shaun Treweek[16], David Jayne[4], Julie Croft[4], Amar Rangan[1], Andrew Metcalfe[5], Elke Gemperle-Mannion[5], David Torgerson[1]
[1]The University of York, York, United Kingdom; [2]University of Bristol, Bristol, United Kingdom; [3]University Hospitals of Leicester, Leicester, United Kingdom; [4]University of Leeds, Leeds, United Kingdom; [5]University of Warwick, Coventry, United Kingdom; [6]York Teaching Hospital NHS Foundation Trust, United Kingdom; [7]University of Oxford, Oxford, United Kingdom; [8]University of Manchester, Manchester, United Kingdom; [9]Bangor University, Bangor, United Kingdom; [10]The University of Sheffield, Sheffield, United Kingdom; [11]University of Leicester, Leicester, United Kingdom; [12]National University of Ireland Galway, Galway, Republic of Ireland; [13]Queen Mary University of London, London, United Kingdom; [14]King's College London, London, United Kingdom; [15]The University of Nottingham, Nottingham, United Kingdom; [16] University of Aberdeen, Aberdeen, United Kingdom
*Trials* 2019, **20(Suppl 1):**PS8A

**Introduction:** Fewer than 50% of RCTs recruit to target. RCTs evaluating surgical interventions are especially challenging. Training trial recruiters is the top priority topic for recruitment research for UK Clinical Trials Unit Directors. There is currently no evidence-based training for staff recruiting patients into surgical RCTs.
The University of Bristol's Qualitative research integrated within Trials (QuinteT) team have developed a one-day ConDuCT-II training course for staff recruiting into surgical RCTs. This training looks promising for increasing confidence with recruitment, raising awareness of hidden challenges, and impacting positively on recruitment practice.
**Aim:** To evaluate the feasibility of undertaking a Studies Within A Trial (SWAT) of the recruiter training intervention on participant recruitment into surgical trials.
**Methods:** A cluster randomised SWAT design. Surgical trials recruiting participants were invited to be host trials. Staff recruiting participants to the host trials in UK hospitals were asked about their interest in attending a training workshop. Interested sites were randomised 1:1 to be offered the training (intervention group) or no training (usual recruitment practice; control group). Outcomes include: percentage of staff randomised, numbers attending training, collection of recruitment data and recruitment rate.
**Timing of potential results:** To date four surgical randomised controlled trials have been recruited: DISC (ISRCTN18254597); PROFHER 2 (ISRCTN76296703); IntAct (ISRCTN13334746); and START:REACTS (ISRCTN17825590). 27 recruiting sites have been randomised, involving 57 recruiting staff. The training for the intervention group will take place on 10th May 2019. By the time of the conference we will present other outcome data such as recruitment rates.
**Potential relevance & impact:** This SWAT demonstrates that it is feasible to test the same recruitment intervention across multiple trials simultaneously, speeding up evidence generation using SWATs. If successful, training staff will help teams recruit more quickly, and improve staff confidence in doing so.

## PS8A

**- O2 Good Statistical Practice: GCP for Statisticians**

Helen Mossop[1], Emma Armstrong[2], Steff Lewis[3], Susan Dutton[4], Clare Peckitt[5], Lucy McParland[2], Carrol Gamble[6], Deborah Stocken[2]
[1]Institute of Health & Society, Newcastle University, United Kingdom; [2]Institute of Clinical Trials Research, Faculty of Medicine and Health, University of Leeds, United Kingdom; [3]Edinburgh Clinical Trials Unit, Usher Institute of Population Health Sciences and Informatics, University of Edinburgh, United Kingdom; [4]Centre for Statistics in Medicine, Oxford Clinical Trials Research Unit, University of Oxford, United Kingdom; [5]The Royal Marsden NHS Foundation Trust, United Kingdom; [6]Clinical Trials Research Centre, Department of Biostatistics, University of Liverpool, United Kingdom
*Trials* 2019, **20(Suppl 1):**PS8A

**Introduction:** Statisticians are fundamental in ensuring clinical trials are conducted with quality, transparency and integrity. Conduct of clinical trials according to Good Clinical Practice (GCP), an internationally recognised, ethical and quality standard, is a regulatory requirement. Statisticians are required to undertake training on GCP but existing training is generic and, crucially, does not cover statistical activities. This results in statisticians undertaking training mostly unrelated to their role and variation in awareness and implementation of relevant regulatory requirements with regards to statistical conduct. The need for role-relevant training is recognised by the HRA, MHRA and MRC as well as the UKCRC Registered CTU and NIHR Statistics Groups. Here we discuss an NIHR funded project to develop and deliver a role-specific GCP training tailored to statisticians.
**Methods:** A scoping survey of the UKCRC Registered CTU Statistics Group identified an obvious need for relevant GCP training. User preference was for a stand-alone, face-to-face course with online training also available for interim completion. Training materials have been developed based on MHRA GCP and cover legislation and guidance for best practice across all clinical trial processes with statistical involvement, incorporating existing UKCRC guidance on analysis plans, validation of statistical programming and data sharing. The course contains exercises and real-life scenarios to bridge the gap between theory and practice. Comprehensive feedback from initial pilot work has been incorporated.

**Timing of Potential Results:** Further pilot work with UKCRC CTU and NIHR Statisticians is underway. Refinement and input from NIHR and MHRA will be completed prior to release, expected October 2019.

**Potential Relevance & Impact:** This project will result in accessible, comprehensive, piloted training with relevance to all statisticians working in the clinical trials arena for national and international adoption. This training will encourage best practice, leading to transparent and reproducible statistical activity as required by regulatory authorities.

## PS8A

### - O3 Career development for Trial Managers: a survey of UK-based trial management professionals

Eleanor Mitchell[1], Natalie Wakefield[1], Suzanne Hartley[2], Alison McDonald[4], Shelley Rhodes[5], Jodi Taylor[6], Kirsteen Goodman[7], Helen Meadows[8], Helen Hickey[9], Barbara Farrell[3], on behalf of the UK Trial Managers' Network
[1]Nottingham Clinical Trials Unit, University Of Nottingham, Nottingham, United Kingdom; [2]Clinical Trials Research Centre, University of Leeds, Leeds, United Kingdom; [3]University of Oxford, Oxford, United Kingdom; [4]The Centre for Healthcare Randomised Trials, University of Aberdeen, Aberdeen, United Kingdom; [5]Exeter Clinical Trials Unit, University of Exeter, Exeter, United Kingdom; [6]Bristol Randomised Trials Collaboration, University of Bristol, Bristol, United Kingdom; [7]NMHAP Research Unit, Glasgow Caledonian University, Glasgow, United Kingdom; [8]Institute of Clinical Trials and Methodology, University College London, London, United Kingdom; [9]Clinical Trials Research Centre, University of Liverpool, Liverpool, United Kingdom
*Trials* 2019, **20(Suppl 1):**PS8A

**Introduction:** Effective trial management is essential to the successful delivery of high quality trials and appointing a dedicated trial manager has been shown to be a factor in trials that recruited more successfully. It's also acknowledged that once trial funding has been awarded, "the most important members of the team are not the Profs and investigators but the trial managers". Trial Managers often come from a diverse range of backgrounds with no recognised career pathway and often learn "on the job". Groups such as the UK Trial Managers' Network (UKTMN) aim to support the career development of trial managers, however there is structural inequality within the field of clinical trials and trial managers deserve a recognised career structure. The aim of this study was to survey UKTMN members to understand what is important to them with respect to their own career development.

**Methods:** We sent an online survey link to all UKTMN members, who are actively working in trial management roles within the UK-setting. A mixture of quantitative and qualitative questions were included. Responders were asked to describe their experience, opportunities and barriers to progression, and what factors are considered important for career development. Quantitative data will be analysed and presented descriptively and free-text responses will be reviewed for themes.

**Timing of potential results:** Results from this survey will be presented at the conference.

**Potential relevance and impact:** Trial Managers play a vital role in the delivery of clinical trials and a key priority for the UKTMN is to support trial managers, providing opportunities to enhance career development. Survey results will demonstrate what is important to trial managers themselves and will be disseminated to significant stakeholders (e.g. funders, CTUs, universities) with the aim of developing a more standardised career structure.

## PS8A

### - O4 What information should be fed back to trial participants? – Findings from a Q-methodology study with trial stakeholders

Hanne Bruhn[1], Marion K. Campbell[1], Vikki Ann Entwistle[3], Rosemary Humphreys[1], Sandra Jayacodi[1], Peter Knapp[2], Katie Gillies[1]
[1]University Of Aberdeen, Aberdeen, United Kingdom; [2]University of York, York, United Kingdom; [3]National University of Singapore, Singapore, Republic of Singapore
*Trials* 2019, **20(Suppl 1):**PS8A

**Introduction:** While recommended as good practice, timely reporting of trial results to participants is rare, perhaps in part because of uncertainties about "what" should be shared, "how", "by whom" and "when". This study aimed to identify what content different trial stakeholder groups consider important and why. The work is part of the REporting Clinical trial results Appropriately to Participants (RECAP) project, which aims to develop guidance for researchers to enable feeding back trial results.

**Methods:** Q-methodology was used to explore the relative importance to stakeholders of informational items for inclusion in feedback to trial participants. Candidate items (n=28) were identified from current guidance on trial feedback.

Participants were sampled purposefully from six trial stakeholder groups: Members of the public with trial experience; NHS Research Ethics Committee Members; Sponsor Representatives; Regulator Representatives; Funder Representatives; Trialists.

Participants were asked to arrange the 28 items in order of importance on a Q-sort grid (whilst undertaking "think aloud"). Quantitative ratings will be analysed using principal components analysis (PCA) with varimax rotation to identify relationships between rankings and different viewpoints. Thematic analysis of the 'think aloud' data will aid interpretation of the PCA findings.

**Timing of Potential Results:** All data have been collected and interviews transcribed. PCA analysis, and qualitative data describing and explaining the viewpoints will be ready in September. Full findings will be presented at the conference.

**Potential Relevance and Impact:** Researchers and trialists sometimes assume they know what information participants want to know about trial results. However, trial participants can have different perspectives and expectations, and the nature and context of the trial findings might also affect what is considered important content. Well-informed guidance on how to feedback trial results to participants should help trial teams align practice with the ethical imperative to disseminate findings to those who contributed.

## PS8A

### - O5 Transparency in Clinical Research: An Audit of Feedback Provision to Participants in Phase III Pragmatic Clinical Trials

Mohammad Zulfiqar Raza, Hanne Bruhn, Dr Katie Gillies
Health Services Research Unit, University of Aberdeen, Aberdeen, United Kingdom
*Trials* 2019, **20(Suppl 1):**PS8A

**Introduction:** Clinical research is increasing in the UK. In 2018, over 700,000 patients were recruited across 99% of the NHS trusts in the UK. With this growing trend in clinical research, there is a need for better public engagement and trust. A key factor in achieving this is promoting transparency through the dissemination of trial results to participants. In 2015, The Health Research Authority (HRA) published guidelines, recommending that all researchers communicate results to their study participants. However, this is not legally binding and the act of communicating results to participants varies greatly. Therefore, we conducted an audit of what research teams said they would do versus what they actually did in practice with regard to feeding back trial results to participants.

**Methods:** The Integrated Research Application System (IRAS) will be used to investigate how researchers report their intention and means of informing patients of Phase III trial results through questions in the submitted ethics application form. Post-study confirmation of dissemination of results is reported in the end of study ethics report which is accessed through the

HRA Assessment Review Portal (HARP). The match between what researchers said they would do on IRAS will be compared to what was actually reported as recorded through HARP and presented as frequencies. We will also present data on the reported involvement of patients in trials as recorded in the IRAS form.
**Timing of Results:** This audit is a part of the RECAP study. Undertaken as an MSc project, it is scheduled to be completed in July 2019.
**Potential Relevance and Impact:** There Is currently a lack of data regarding the compliance to the 2015 HRA guidelines in the UK. The results of this audit will hopefully give a baseline to measure the impact of any potential future measures designed to increase compliance.

## PS8B
### - O1 Using the learning curve and Bayesian analysis to decide when surgeons are ready to randomise

Fei Shan[1,2], Ziyu Li[2], Allison Hirst[1], Peter McCulloch[1]
[1]Nuffield Department of Surgical Sciences, University of Oxford, Oxford, United Kingdom; [2]Gastrointestinal Cancer Center, Peking University Cancer Hospital & Institute, Beijing, China
*Trials* 2019, **20(Suppl 1):**PS8B

**Objectives:** Surgeons' learning curves may bias trials for new procedures, but methods to estimate learning curves lack precision. We investigated whether repeated Bayesian analysis is a useful alternative.
**Methods:** We applied repeated Bayesian inference to a dataset (415 cases) from an experienced surgeon learning laparoscopic gastrectomy. We developed methods to predict the point probability of learning phase completion, and to estimate power for this prediction.
Through literature review, we identified learning curve effects for operation time, number of retrieved lymph nodes and postoperative complications, and derived prior probabilities for each indicator. We calculated the probability density function assuming a "normal" probability distribution, and used repeated Bayesian inference for each indicator and for combinations to identify when 80%, 95% and 99% predicted the probability of learning curve completion was achieved.
**Results:** The method successfully modelled the probability of completing the learning curve in real operative data, achieving a degree of precision suitable for practical use. Curve completion varied widely depending on the single indicator used: 95% probability was achieved at case 14 (operative time) 277 (complications) and 415 (Lymph node yield). Combining all three indicators, 95% probability was achieved at case 38 (80% at case 35 and 99% at case 44).
**Discussion:** Evaluating real surgical learning curves is challenging because surgeons modify their technique and indications as they learn, producing a multi-peak curve. Single estimators give very different estimates of curve completion, ranging from 14 to over 400 in this series, and are therefore poor guides training or clinical research decisions. The Bayesian analysis allowed precise estimation of learning curve completion using a combination of estimators, giving a more clinically meaningful figure for learning curve completion than single estimators. It also permits real-time updating of probability estimation and confidence. Great care is required in selecting appropriate priors, to ensure credible conclusions.

## PS8B
### - O2 Statistical considerations in a non-inferiority trial: results from the PERSEPHONE early breast cancer herceptin duration trial

Janet A Dunn[1], Louise Hiller[1], Donna Howe[1], Anne-Laure Vallier[2], Kerry Raynes[1], Helen Higgins[1], David A Cameron[3], David Miles[4], Andrew M Wardley[5], Helena Earl[2]
[1]University Of Warwick, Coventry, United Kingdom; [2]University of Cambridge, Cambridge, United Kingdom; [3]University of Edinburgh, Edinburgh, United Kingdom; [4]St Guys & St Thomas NHS Trust, London, United Kingdom; [5]Christie Hospital NHS Trust, Manchester, United Kingdom
*Trials* 2019, **20(Suppl 1):**PS8B

**Introduction:** Non-inferiority (NI) trials set out to show that a new treatment is not inferior to the standard treatment at a certain predefined margin, for a pre-defined set of assumptions. NI trials are particularly appropriate when a new treatment is thought to offer greater safety or convenience, or less expense, while providing similar efficacy to a standard. However these trials need careful monitoring and interpretation.
**Methods:** PERSEPHONE is a 4000-patient prospective, multicentre, phase 3 randomised trial that tested the hypothesis that 6 months of trastuzumab therapy is non-inferior to the 12 month standard in patients with HER2 positive early breast cancer. Prior to the set-up of the trial, there was consensus from the trial management group and the patient and public involvement group, that an absolute reduction up to 3% in 4-year disease-free-survival (DFS) for the 6-month treatment group was acceptable. Assumptions for the 4-year DFS rate expected in PERSEPHONE were based on results from previous studies.
**Results:** The control arm 4-year DFS at the time of analysis was 89.8% which was higher than the original estimate of 80% which had an effect on the NI margins. Following the procedures set out in the SAP and agreed by the DMC, the HR limit for NI was set at 1.32 and findings were that 6 months trastuzumab was NI to 12 months trastuzumab (HR=1.07 (90%CI 0.93-1.24), p for NI=0.01).
**Discussion:** In a comparator trial there is a certain level of flexibility around the timing of analyses and the relaxing of assumptions. However, in a NI trial the assumptions and pre-specified criteria on which to base the interpretation of NI are crucial and have to be monitored with care. Our experience with the interpretation of the PERSEPHONE trial has highlighted a requirement for consolidated reporting instructions for NI trials.

## PS8B
### - O3
### Abstract omitted

## PS8B
### - O4 The ADAPTT Study: Using routinely-collected data to emulate a randomised trial

Jessica Harris[1], Jonathan AC Sterne[2], Barnaby C Reeves[1], Thomas W Johnson[3], Umberto Benedetto[4], Daniel Lasserson[5], Yoon Loke[6], Andrew Mumford[4], Chris A Rogers[1], Maria Pufulete[1]
[1]Clinical Trials and Evaluation Unit, Bristol Trials Centre, University of Bristol, Bristol, United Kingdom; [2]NIHR Biomedical Research Centre, Department of Population Health Sciences, University of Bristol, Bristol, United Kingdom; [3]Bristol Heart Institute, Bristol, United Kingdom; [4]Bristol Medical School, University of Bristol, Bristol, United Kingdom; [5]Institute of Applied Health Research, University of Birmingham, Birmingham, United Kingdom; [6]Norwich Medical School, University of East Anglia, Norwich, United Kingdom
*Trials* 2019, **20(Suppl 1):**PS8B

**Introduction:** "Target trial" emulation means applying the design principles from randomised controlled trials (RCTs) to the analysis of observational data. The ADAPTT study (ISRCTN ISRCTN76607611) is using routinely-collected health data to emulate four pragmatic "target RCTs" to quantify associations between different regimens of dual antiplatelet therapy (DAPT) and bleeding events in four populations: i) acute coronary syndrome (ACS) undergoing percutaneous coronary intervention (PCI); ii) stable angina undergoing PCI; iii) coronary artery bypass grafting (CABG); and iv) ACS treated conservatively (medication only).
**Methods:** We defined eligibility criteria, treatment strategies, assignment to interventions and follow up using clinical input and review of the DAPT literature. We applied the eligibility criteria and identified our populations from fully anonymised, linked Hospital Episode Statistics (HES) and Clinical Practice Research Datalink (CPRD) data. To emulate random assignment to different DAPT regimens at baseline, we identified potential confounding factors (systematically, using literature review, interviews with clinicians and short surveys with additional clinicians) to allow description of comparability of groups at baseline and adjustment for these factors. Bleeding events were identified from GP consultations (CPRD) and inpatient stays (HES) in the year after "assignment" to DAPT interventions.
**Results:** Preliminary analyses show variation in bleeding risk consistent with expectation based on the characteristics of the DAPT regimens. For example, in the ACS PCI population, bleeding occurred in 10% of patients receiving more potent DAPT versus 8% in patients receiving less potent DAPT (adjusted hazard ratio 1.38, 95% confidence interval 1.16 to 1.64). We are unable to control for some confounding factors that were identified.
**Discussion:** Detailed consideration of eligibility criteria, treatment assignment, start and end of follow-up period and potential confounding factors, along with an intention-to-treat analysis strategy, will hopefully avoid the common pitfalls of observational studies.

## PS8B

### - O5 The use of visual analytics for clinical trial safety outcomes: a methodological review

Rachel Phillips[1], Victoria Cornelius[1], Suzie Cro[1], Odile Sauzet[2]
[1]School of Public Health, Imperial College London, London, United Kingdom; [2]Epidemiologie & International Public Health, Faculty of Health Sciences, Universität Bielefeld, Bielefeld, Germany
*Trials* 2019, **20(Suppl 1):**PS8B

**Introduction:** Visual analytics covers the use of graphical displays to communicate data effectively. In the clinical trial setting, graphics can provide an efficient means to convey and interpret large amounts of emerging adverse event (AE) data. This view is supported by guidelines, such as the CONSORT extension on harms and recommendations from journal editors and the pharmaceutical industry. We aimed to identify, appraise and demonstrate the use of graphical displays proposed specifically to analyse safety outcomes in clinical trials.
**Methods:** A scoping review was undertaken to identify articles that proposed graphical displays for pre-specified and non-specific emerging events. We systematically searched Medline and EMBASE databases via OVID and Web of Science and SCOPUS databases. We also searched the reference lists of eligible articles to identify any articles the database search may have missed. Information on methods were extracted and applied to trial data.
**Results:** The review identified eight papers published between 2001-18 proposing 20 unique plots to display safety data. Examples of these plots will be provided and appraised for binary AEs, continuous laboratory (e.g. bloods) and vital signs (e.g. pulse-rate) data. We will contrast the visual summary from two of these plots, the volcano and dot plot, with AE data presented in published articles, including data from Whone et al. 2019's Parkinson's study. Both the volcano and dot plot provide visual summaries of the incidence of emerging AEs.
**Discussion:** Graphical approaches offer an efficient means to summarise large amounts of emerging AE data from RCTs and many have been proposed. Statistical software eases the implementation of graphical displays, however; previous work identified only a small proportion of journal reports incorporating graphics into the AE analysis. We have demonstrated the benefits of graphical displays and share newly developed Stata code for implementation to promote the wider uptake of such methods.

## PS9A

### - O1 Undertaking trials methodology research using data from clinical trial registries: an exemplar related to core outcome set uptake

Jamie J Kirkham[1, 3], Paula Williamson[3], Mike Clarke[2]
[1]University of Liverpool, Liverpool, United Kingdom; [2]Queen's University Belfast, Belfast, United Kingdom; [3]University of Manchester, Manchester, United Kingdom
*Trials* 2019, **20(Suppl 1):**PS9A

**Introduction:** Clinical trial registries are being increasingly used as data providers in trials methodology research. The objective of this study is to demonstrate how we can use the information from multiple data providers registered with the World Health Organisation-International Clinical Trials Registry Platform (WHO-ICTRP) to evaluate the uptake of a core outcome set (COS), and to discuss the advantages and challenges of this approach. As a motivating example we examine how to assess the uptake of the well-known rheumatoid arthritis COS.
**Methods:** An observational review was carried out on clinical trials of rheumatoid arthritis that were indexed on the WHO-ICTRP from inception to 9th November 2018. Four measures of uptake were calculated in order to assess the reliability of results when considering data from both trial registries which includes ongoing trials, trial publications from completed trials and a combination of both sources. As part of the assessment we evaluate the efficiency, reliability and geographic diversity of using clinical trial registries in trials methodology research.
**Results and Discussion:** A total of 341 unique trials were eligible for the evaluation of COS uptake which were taken from 13 out of the 17 data providers listed on the WHO-ICTRP search portal. The four uptake statistics that estimated the proportion of trialists that intended to measure and/or report the full COS ranged from 70-79%. The highest level of uptake came from trials with published results while the lowest uptake rate was based on planned trial outcomes that came from trial registry data. Use of the WHO-ICTRP identified eligible trials from all major continents and in this application provided a reliable and efficient estimate of COS uptake without the need to find, obtain and read trial publications. However, the quality of trial registry data may be lower due to reporting deficiencies.

## PS9A

### - O2
**Abstract omitted**

## PS9A

### - O3 To add or not to add a new treatment arm to an on-going trial

Kim May Lee[1], James Wason[1,2], Nigel Stallard[3]
[1]University Of Cambridge; [2]Newcastle University; [3]University of Warwick
*Trials* 2019, **20(Suppl 1):**PS9A

**Context**: Clinical trials are expensive investments that aim to evaluate the efficacy of new treatments on patients. When there are several new treatments available for testing at different times, a platform trial approach can be considered. This trial approach allows for adding new arms to an existing trial that has similar objectives and settings. This feature of adding arms is appealing to practitioners because of the efficiencies from running one trial instead of several.

Nevertheless, the decision of always opening a new arm in an existing trial may reduce the resources for testing the initial treatments, and prolong the overall duration to establish the treatment benefits. On the contrary, the decision to not add may reduce the chances of patients getting better treatments earlier.
**Methods:** To support the decision of adding or not adding a new treatment arm to an existing trial, this work proposes a decision-theoretic framework. Two ways of defining utility are considered in the framework: one for trials that aim to maximize the number of rejected hypotheses; the other for trials that would declare a success when at least one hypothesis is rejected from the study. Within a two-stage trial setting, the framework provides an optimal decision based on the observed data from an initial stage.
**Results:** The marginal benefits of adding arms vary according to the utility considered in the framework and the design of the trials. The decision to add a new treatment is optimal under two scenarios: when the initial treatments are not more efficacious than the control treatment, and when the initial treatments are considerably more efficacious than the control treatment.
**Discussion:** Adding a new treatment arm to an on-going trial when it becomes available is not always optimal. The progress of the initial treatment arms shall not be ignored.

## PS9A
### - O4 Introducing the CONsolidated Standards of Reporting Trials (CONSORT) statement for randomised controlled trials (RCTs) using cohorts and routinely collected health data

Edmund Juszczak[1], Linda Kwakkenbos[3], Stephen McCall[1], Mahrukh Imran[4], Lars G Hemkens[5], Merrick Zwarenstein[6], Ole Fröbert[16], Clare Relton[17], Margaret Sampson[18], Chair Lehana Thabane[7], Eric I Benchimol[8], Marion K Campbell[9], David J Torgerson[10], David Erlinge[11], Danielle B Rice[12], Sinéad Langan[13], Kimberly A Mc Cord[5], Tjeerd P van Staa[14], David Moher[15], Helena M Verkooijen[19], Rudolf Uher[20], Maureen B Worron-Sauve[21], Isabelle Boutron[22], Philippe Ravaud[23], Brett D Thombs[4], Chris Gale[2]
[1]National Perinatal Epidemiology Unit, Nuffield Department of Population Health, University Of Oxford, Oxford, United Kingdom; [2]Imperial College London, London, United Kingdom; [3]Behavioural Science Institute, Clinical Psychology, Radboud University , Nijmegen , The Netherlands; [4]Lady Davis Institue for Medical Research, Jewish General Hospital, Montreal, Canada; [5]Basel Institute for Clinical Epidemiology and Biostatistics, Department of Clinical Research, University Hospital Basel, University of Basel, Basel, Switzerland; [6]Depts of Family Medicine and Epidemiology/Biostatistics, Western University , London , Canada; [7]Biostatistics Unit (St Joseph's Healthcare)/FSORC, Hamilton, Canada; [8]Department of Pediatrics and School of Epidemiology and Public Health, University of Ottawa, Ottawa, Canada; [9]University of Aberdeen, Aberdeen, United Kingdom; [10]University of York, York, United Kingdom; [11]Department of Cardiology, Lund University, Skane University Hospital, Lund, Sweden; [12]McGill University, Montreal, Canada; [13]London School of Hygiene and Tropical Medicine, London , United Kingdom; [14]University of Manchester, Manchester, United Kingdom; [15]Centre for Journalology, Clinical Epidemiology Program, Ottawa Hospital Research Institute, Ottawa, Canada; [16]Örebro University Hospital, Örebro, Sweden; [17]Pragmatic Clinical Trials Unit, Queen Mary University of London, London, United Kingdom; [18]CHEO, Ottawa, Canada; [19]University Medical Center Utrecht, Utrecht, the Netherlands; [20]Dalhousie Unversity, Department of Psychiatry, Halifax, Canada; [21]Scleroderma Canada, Hamilton, Canada; [22]Université de Paris, Paris, France; [23]Centre de Recherche Épidémiologie et Statistique Sorbonne Paris Cité (CRESS-UMR1153), Paris, France
*Trials* 2019, **20(Suppl 1):**PS9A

**Background:** Randomised controlled trials (RCTs) are increasingly being conducted using existing sources of data, such as cohorts, administrative databases, disease registries and electronic health records. RCTs conducted using existing data sources require additional information to be reported. This reporting guideline is an extension of the 2010 version of the Consolidated Standards of Reporting Trials (CONSORT) Statement for RCTs using cohorts and routinely collected health data.
**Methods:** A long-list of potential items for the checklist was identified through two methods: firstly, modifications to the current CONSORT checklist were generated using existing reporting guidelines, including the Reporting of Observational Studies in Epidemiology (STROBE) and REporting of studies Conducted using Observational Routinely-collected health Data (RECORD) statements. Secondly, a scoping review of RCTs conducted in the last decade using cohorts and routinely collected health data facilitated the modification and identification of other potential items. Using the long-list, a three-stage Delphi exercise was conducted to assess the importance of each item for inclusion in the final extension checklist, which was finalised at a face-to-face meeting of experts.
**Results:** A long-list of 27 items was created and 125 experts registered for the three-round Delphi exercise (92, 77 and 62 experts participated in each round respectively). Consensus was reached on 21 out of 27 items. The results of the Delphi exercise informed a face-to-face consensus meeting in May 2019; core items to be included in the extension checklist were finalised at this meeting. Corresponding explanations of extensions and new items with examples of good reporting were developed subsequently.
**Conclusion:** The guideline checklist can facilitate transparent reporting of RCTs using cohorts and routinely collected health data, to assist evaluations of rigour and reproducibility, enhance understanding of the methodology, and make the results more useful for clinicians, journal editors, reviewers, guideline authors, and funders.

## PS9A
### - O5
**Abstract omitted**

## PS9B
### - O1 Trial recruitment decision-making: crucial but not evidence-based

Shaun Treweek[1], Marie Pitkethly[2], Jonathan Cook[3], Cynthia Fraser[1], Elizabeth Mitchell[4], Frank Sullivan[5], Catherine Jackson[5], Tyna Taskila[6], Heidi Gardner[1]
[1]University Of Aberdeen, UK; [2]University of Dundee, UK; [3]University of Oxford, UK; [4]University of Hull, UK; [5]University of St Andrews, UK; [6]The Work Foundation, UK
*Trials* 2019, **20(Suppl 1):**PS9B

**Introduction:** Evidence-based trial recruitment strategies would benefit patients, trialists and health research. This talk summarises the 2018 update to the Cochrane review of strategies to improve recruitment to randomised trials.
**Methods:** Randomised evaluations of recruitment interventions embedded within a host randomised trial were eligible. Six databases, including MEDLINE were searched. Abstract screening, full text assessment, data extraction, risk of bias and GRADE assessments were conducted independently by two reviewers. Risk difference and 95% confidence interval (CI) were calculated. Meta-analysis was done where appropriate. Protocols for evaluation of interventions considered priorities for replication studies were developed.

**Results:** 24,432 abstracts were screened and 68 studies included, involving over 74,000 people. We found 72 interventions; only three were GRADE High certainty:
1.Open trials rather than blinded, placebo trials. Risk difference: 10% (95% CI 7% to 13%).
2.Telephone reminders to people who do not respond to a postal invitation. Risk difference: 6% (95% CI 3% to 9%).
3.Using a bespoke, user-tested participant information leaflet. Risk difference: 1% (95% CI -1% to 3%).
Eight other interventions had GRADE Moderate certainty with certainty generally being reduced because of only having a single evaluation. Only seven interventions had been evaluated more than once and the uncertainty around most interventions in the review has not changed in a decade. Further evaluations of telephone reminders, text messages and financial incentives were considered priorities because of remaining uncertainty combined with their potential for widespread use and evaluation protocols provided as part of the review.
**Discussion:** The evidence base to support trial recruitment is poor. Where evaluations have been done a combination of design flaws, solitary evaluations and poor precision mean we can conclude little from the bulk of them. Prioritised and coordinated approaches to generating recruitment evidence are needed to avoid another decade without progress.

## PS9B

### - O2 Enrolling patients without capacity to trauma trials; successes and challenges

Stephanie Wallis, Robin Lerner, Elizabeth Tutton, Juul Achten, Xavier Griffin, Matthew Costa
University Of Oxford, Oxford, United Kingdom
*Trials* 2019, **20(Suppl 1):**PS9B

**Introduction:** Patients lacking capacity are frequently excluded from research populations. The results of research carried out without these patients, may not be generalisable and patients lacking capacity could miss out on access to evidence-based treatment. The reasons for excluding patients based on capacity vary, but it has been suggested that recruiting and collecting outcomes is difficult, and that researchers are unsure of the ethical implications.
Lack of capacity has been reported in 30- 40% in hip fracture patients. The World Hip Trauma Evaluation (WHiTE) trials, investigating this patient group, are amongst the largest studies to include patients without capacity.
**Aim:** This paper describes the process used by WHiTE to recruit patients lacking capacity, the retention of patients and success of follow-up.
**Method:** Patients lacking capacity are usually enrolled by a professional consultee (PrC) before surgery. After surgery, if appropriate, patients will either be asked to consent to continuing participation or a personal consultee (PeC) will be approached. We recorded data on pre- and post-enrollment consent types, withdrawals, and 120-day retention rate.
**Results:** 2,146 patients enrolled between June 2016 and April 2019 - 1,461 (68%) were enrolled using consultee agreement. After surgery, of the 1,384 patients enrolled by PrC, 25% continued under patient consent, 36% under PeC agreement and 18% under continued PrC agreement. Only 7% of patients/PeC declined to continue with the study. 71% of patients initially enrolled by Consultees provided 120-day follow-up, compared to 89% of those enrolled under patient consent.
**Conclusion:** Recruiting and collecting data from a frail patient group lacking capacity is feasible. The vast majority of participants who regained capacity agreed to continue participating.
Whilst retention rates for patients who initially lacked capacity were slightly lower than those for patients who provided consent, inclusion of this data increased the overall generalisability of the data.

## PS9B

### - O3 Prediction and monitoring of patient recruitment in clinical trials: gaps between current practice and available methodology

Efstathia Gkioni[1,2], Susanna Dodd[1], Roser Rius[3], Carrol Gamble[1]
[1]Department of Biostatistics, University of Liverpool, Liverpool, United Kingdom; [2]Paris Descartes University, Sorbonne Paris Cité, Paris, France; [3]Department of Statistics and Operations Research, School of Mathematics and Statistics, BarcelonaTech (UPC), Barcelona, Spain
*Trials* 2019, **20(Suppl 1):**PS9B

**Introduction:** Predicting the recruitment of patients in clinical trials is challenging. Participation rates may be lower than expected due to several factors, which may include unrealistic recruitment rates.
However, approaches used to predict and monitor recruitment remain frequently unreported. Various deterministic and stochastic statistical models have been developed and published to model the process.
We aimed to determine how recruitment is predicted and monitored and whether the methods developed are being utilised in practice.
**Methods:** A survey was developed to identify current practice and knowledge of available methodology. The survey was piloted and then circulated across the UKCRC registered CTU Statistics Group and the European Clinical Research Infrastructure Network (ECRIN). We aimed to seek their opinions and experiences in relation to prediction and monitoring of recruitment.
**Results:** Responses were received from 50/51 across the UK CRC network and from 19/53 across ECRIN. 81% (56/69) believed that a statistician should be involved in prediction and monitoring of recruitment to provide a more realistic estimate of recruitment targets and accrual periods.
However, the majority of responders 90% (62/69) did not recognise recruitment as a stochastic process in the approaches used. 42% (29/69) stated a preference for using simple approaches, with 36% (25/69) being unconvinced of the value of implementing the models identified.
Simple approaches were also used for monitoring accrual, where investigators use tables (83%, 57/69) and graphs (87%, 60/69) showing the expected and actual recruitment rates. When participants were asked whether they would use a web application implementing the models mentioned, 55% (38/69) responded that they would use it for both prediction and monitoring.
**Discussion:** Recruitment is a stochastic process, however, statisticians are not utilising statistical distributions within the approaches used to predict or monitor recruitment. The value of implementing these complex models needs to be demonstrated prior to adoption.

## PS9B

### - O4 Traumatic Decisions; Research Recruitment and Randomisation in an Acute Emergency Setting

Claire Cochran[1], Marion K Cambell[1], Jan O Jansen[2], Chukwuemeka Emele[1], Mark Forrest[1], Alison McDonald[1]
[1]University Of Aberdeen, Aberdeen, United Kingdom; [2]University of Alabama at Birmingham, Birmingham, USA
*Trials* 2019, **20(Suppl 1):**PS9B

**Introduction:** Emergency research within the clinical context of an acute trauma situation poses a unique set of challenges in terms of recruitment and consent. Patients who are incapable of giving consent are an established exception to the general rule of informed consent in clinical trials and in such situations it is neither practicable nor ethical to embark on lengthy recruitment screening and randomisation exercise.

Uncontrolled haemorrhage (bleeding) is responsible for approximately one-third of trauma deaths. Temporary aortic occlusion can limit haemorrhage and maintain blood flow to the heart and brain thereby improving patient survival odds. REBOA (Resuscitative Endovascular Balloon Occlusion of the Aorta) is a novel technique whereby a percutaneously inserted balloon is deployed in the aorta, providing a relatively quick means of temporarily controlling haemorrhage (via aortic occlusion) until definitive control can be attained.

The National Institute for Health Research (NIHR) Health Technology Assessment (HTA) funded UK-REBOA trial aims to establish the clinical and cost-effectiveness of REBOA, as compared with standard treatment alone, for the management of uncontrolled torso haemorrhage caused by injury.

**Methods and Discussion:** In this paper we describe the decision making process behind establishing the most effective and efficient randomisation and consent pathways for the UK- REBOA trial. We will discuss the pros and cons of different approaches to randomisation considered as well as describe the bespoke randomisation service which was eventually developed. Additionally, we will also describe the 'consent' models considered and explain the reasoning for the approach finally chosen.

**Results:** All decisions we had to make were influenced not only by similar trials practice, but also heavily by legal requirements, ethics and the practical capacity of the trial staff involved. It is likely that lessons from the UK REBOA Study experience will be both valuable and applicable to other similar emergency care studies.

## PS9B

### - O5 Enhancing practitioner explanations and parental understandings of recruitment and consent- an adapted model for paediatric emergency medicine trials

Louise Roper[1], Kerry Woolfall[1], Mark D. Lyttle[2], Carrol Gamble[3,4,7], Amy Humphreys[4,7], Helen Hickey[4,7], Shrouk Messahel[5], Elizabeth Lee[5], Joanne Noblet[5], Naomi E.A. Rainford[4,7], Anand Iyer[6], Richard E. Appleton[6]
[1]Institute of Population Health, University of Liverpool, Liverpool, United Kingdom; [2]Emergency Department, Bristol Royal Hospital for Children, Bristol, United Kingdom; [3]Department of Biostatistics, University of Liverpool, Liverpool , United Kingdom; [4]Clinical Trials Research Centre, University of Liverpool, Liverpool, United Kingdom; [5]Emergency Department, Alder Hey Children's NHS Foundation Trust , Liverpool , United Kingdom; [6]Department of Neurology, Alder Hey Children's NHS Foundation Trust, Liverpool , United Kingdom; [7]Institute of Child Health, Alder Hey Children's NHS Foundation Trust , Liverpool , United Kingdom
*Trials* 2019, **20(Suppl 1):**PS9B

**Introduction:** Alternatives to prospective informed consent have enabled the conduct of paediatric emergency medicine (PEM) trials. Research without prior consent (RWPC) involves practitioners approaching parents after the trial intervention has been given and seeking consent for their child to continue in the trial. As part of an embedded study in 'The Emergency treatment with Levetiracetam or Phenytoin in Status Epilepticus in children' (EcLiPSE) trial (across 30 hospital sites in the UK) we aimed to explore how the trial and RWPC was described by practitioners during recruitment discussions, and how well this information was understood by parents.

**Methods:** Qualitative analysis of audio recorded (i) trial discussions and (ii) telephone interviews with parents within two months of hospital discharge. Thematic analysis drew upon the Realpe et al (2016) model for successful trial recruitment.

**Results:** We obtained 76 recorded trial discussions between parents and EcLiPSE trial recruiters, and conducted 30 parent telephone interviews. For 19 parents we had both recorded trial discussion and interview data, which were matched for analysis. Parental understanding of the EcLiPSE trial was enhanced when practitioners: provided a comprehensive description of trial aims; explained RWPC; discussed uncertainty about which intervention was best; gave a balanced description of each intervention; provided a clear explanation about randomisation; and provided an opportunity for questions. We present an adapted six step model to inform recruitment and RWPC discussions in future PEM trials.

**Discussion:** This study provides a framework to help enhance recruitment and parent understanding of PEM trials involving RWPC. Further testing of this model in PEM trials is required.

## PS9C

### - O1 A comparison of Phase I dose-escalation designs in clinical trials with monotonicity assumption violation

Pavel Mozgunov[1], Caroline Rossoni[2], Thomas Jaki[1], Xavier Paoletti[2], Rachid Abbas[2]
[1]Lancaster University, Lancaster, United Kingdom; [2]Institute Gustave Roussy, Villejuif, France
*Trials* 2019, **20(Suppl 1):**PS9C

**Introduction**: In oncology, new combined treatments make it difficult to order dose levels with increasing toxicity to satisfy the monotonicity assumption. New dose-finding designs that take into account uncertainty in dose levels ordering were compared to classical designs through simulations with monotonicity assumption violation. We give recommendations for the choice of dose-finding design.

**Methods:** Motivated by a combination-schedule clinical trial for patients with high-risk neuroblastoma in which several combinations could not be ordered prior to the trial, we considered both designs that require a monotonicity assumption, the Bayesian continual reassessment method (CRM), the modified toxicity probability interval (mTPI), and designs that allow to adapt dose level ordering during the trial the Bayesian partial ordering CRM (PO-CRM) and the no monotonicity assumption (NMA). We considered 8 scenarios including monotonic and non-monotonic dose-toxicity relationships among 6 dose levels.

**Results:** For monotonic scenarios, the CRM resulted in the highest proportion of correct selections (PCS). However, the CRM failed to identify the target dose level if the monotonicity assumption is violated and tends to recommend either underdosing or highly toxic levels. While the designs relaxing monotonicity assumption, NMA, and PO-CRM, were able to identify the target dose level in monotonic scenarios, they also resulted in the highest PCS in non-monotonic scenarios, nearly 20% higher than alternatives designs.

**Discussion:** The violation of the monotonicity assumption has dramatic effects on the performance of dose-finding designs, we recommend the consideration of designs that can relax this assumption like the NMA or the PO-CRM. The choice of the design must be guided by comprehensive simulations.

## PS9C

### - O2 A meta-analysis of toxicity and efficacy outcomes by dose in recent phase I trials in oncology

Kristian Brock[1], Victoria Homer[1], Gurjinder Soul[1], Claire Potter[1], Codruta Chiuzan[2], Shing Lee[2]
[1]University Of Birmingham, Birmingham, United Kingdom; [2]Columbia University, New York, USA
*Trials* 2019, **20(Suppl 1):**PS9C

**Introduction:** Dose-finding trials commonly seek to identify a maximum tolerable dose. Common experimental designs include the rule-based 3+3, and the model-based continual reassessment method (CRM, O'Quigley, 1990). Each assumes that the probabilities of toxicity and efficacy increase monotonically as dose is increased. If this assumption is violated, these methods may recommend inappropriate doses.
**Methods:** We collated a dataset to investigate the appropriateness of these assumptions. Chiuzan et al. (2017) conducted a systematic methodology review of oncology dose-finding trials between 2008 and 2014. We extracted dose-level response and dose-limiting toxicity (DLT) outcomes from 122 manuscripts in their sample. We then analysed the series individually using simple logistic regression models, and collectively using hierarchical Bayesian models.
**Results:** DLT outcomes were collected for 131 series of fully-ordered doses. Several manuscripts yielded more than one series. DLT outcomes convincingly had a positive gradient with respect to dose for many treatment classes, including chemotherapy, inhibitors, and radiotherapy. Monotonicity was less certain for monoclonal antibodies, although sample size was small.
Response outcomes were collected for 77 series. Evidence was weak that the probability of response increased with dose in all treatment types. Generally, the odds of toxicity increased faster than odds of response.
**Conclusions:** Rationale dictates that subtherapeutic doses exist. In recent dose-finding trials, these data suggest that escalations have tended to coerce greater toxicity risk without commensurate increases in efficacy. This asymmetry suggests that trialists should avoid relying unduly on establishing a maximum tolerable dose and instead focus on identifying a dose that offers an attractive trade-off between toxicity and response. Use of so-called seamless phase I/II designs could help achieve this goal, including EffTox (Thall & Cook, 2004) and Wages & Tait (2015).
**Availability of materials:** full dataset is available at: http://edata.bham.ac.uk/337/1/Database.xlsx
This article and accompanying analyses are available at: https://github.com/brockk/dosefindingdata/tree/master/docs/ICTMC2019

## PS9C

### - O3 Dose-Transition Pathways for Time-to-event Continual Reassessment Method: To wait or not to wait?

Christina Yap[1,2], Shing Lee[3], Ken Cheung[3]
[1]University Of Birmingham, United Kingdom; [2]The Institute of Cancer Research, United Kingdom; [3]Columbia University, United States
*Trials* 2019, **20(Suppl 1):**PS9C

**Introduction:** Most dose-finding designs have multiple enrollment suspensions as they require each cohort of patients to have complete evaluation of occurrence of dose-limiting toxicity (DLT) before new patients can be recruited. The time-to-event Continual Reassessment Method (TITE-CRM), which uses a weighted measure based on DLT follow-up period, has the attractive feature of not requiring enrollment suspension while patients are still being observed for DLT (Cheung & Chappell 2000). However, questions that puzzle trialists during its implementation include: At any given time, how much information is required before the TITE-CRM will recommend a dose escalation? Our aim is to create an implementation tool to help trialists understand how the TITE-CRM works for the immediate next patient(s) and to decide when one should impose a waiting window to allow for better informed decision.
**Methods:** Extending previous work on Dose-Transition Pathways (DTP) by Yap et al 2017 (based on complete information only), we provide a simple tool, TITE-DTP, to aid trialists to decide whether one should enrol the next available patient(s) per the TITE-CRM's current recommendation, or one should wait for additional information from patients still being followed for DLT. Data visualization tool will be used to help the trialists understand how additional information will affect the recommendation.
**Results:** Without any imposed waiting, one might dose too many patients at the current dose due to insufficient information for the model to recommend a change. We illustrate the usefulness of TITE-DTP as a tool to guide the conduct of TITE-CRM in two ongoing cancer trials.
**Conclusions:** Though it is attractive to have continuous enrolment using TITE-CRM to expedite trial delivery, it might sometimes be worthwhile to wait to accumulate more safety information. This would allow more patients to be treated at higher doses if safe, which is particularly useful when patient numbers are limited.

## PS9C

### - O4 Practicalities in running early-phase trials using the Time-to-Event Continual Reassessment Method for interventions with long toxicity periods

Elena Frangou[1], Sharon Love[1], Samantha Hinsley[2], Jane Holmes[3], Sarah Brown[4], Erik van Werkhoven[5]
[1]MRC MRC Clinical Trials Unit at UCL, London, United Kingdom; [2]Cancer Research UK Clinical Trials Unit, Glasgow, United Kingdom; [3]Centre for Statistics in Medicine, Nuffield Department of Orthopaedics, Rheumatology and Musculoskeletal Sciences, Oxford, United Kingdom; [4]Clinical Trials Research Unit, University of Leeds, Leeds, United Kingdom; [5]Netherlands Cancer Institute, Amsterdam, The Netherlands
*Trials* 2019, **20(Suppl 1):**PS9C

**Introduction:** Model-based designs for dose-finding studies such as the Continual Reassessment Method (CRM) are now becoming more commonplace amongst clinicians, statisticians and trial management staff. Complexities and challenges in developing and delivering trials using these methods have slowed down their implementation, albeit suitable guidance is now emerging to make the process of trial design and set-up easier and more efficient [1].
In radiotherapy trials, toxicities can occur a long time after treatment has finished. Consequently, the lifetime of such trials may become extremely long with most standard phase I designs. The Time-to-Event CRM (TiTE-CRM), a modification to the original CRM, accounts for the time to event of late-onset toxicities and results in shorter trial duration.
**Methods:** The TiTE-CRM encapsulates the dose-toxicity relationship with a statistical model, taking into account the observed toxicities and a weight for the proportion of completed follow-up of patients without toxicity. This model uses all available data to determine the next patient's dose and subsequently declare the maximum tolerated dose.
**Results:** We present some practicalities in designing, setting-up and running TiTE-CRM trials. In particular, we describe the properties that need to be defined at the design stage, especially time-related parameters such as the toxicity observation window, discuss issues and propose solutions with regard to the grant application and suggest ways of conducting the trial efficiently, without additional burden e.g. timely collection of data when a dose allocation needs to take place.
**Discussion:** Model-based designs can be complex but we found they are both feasible and worthwhile. Sharing experience and knowledge is key and by doing so, we aim to demystify the conduct of dose finding trials using the TiTE-CRM.

[1]: Wheeler, G. M., et al. (2019). "How to design a dose-finding study using the continual reassessment method." BMC Medical Research Methodology 19(1): 18.

## PS9C

### - O5 Setting up a stopping boundary for safety in a phase II trial: the Poppi trial

Jennifer L Bell[1], Pollyanna Hardy[2], Nigel Stallard[3], Rebeccah Slater[4], Caroline Hartley[4], Eleri Adams[5], Gabrielle Green[4], Fiona Moultrie[4]
[1]National Perinatal Epidemiology Unit, Nuffield Department of Population Health, University of Oxford, Oxford, United Kingdom; [2]Birmingham Clinical Trials Unit, University of Birmingham, Birmingham, United Kingdom; [3]Warwick Medical School, University of Warwick, Coventry, United Kingdom; [4]Department of Paediatrics, University of Oxford, Oxford, United Kingdom; [5]Newborn Care Unit, John Radcliffe Hospital, Oxford University Hospitals NHS Foundation Trust, Oxford, United Kingdom
*Trials* 2019, **20(Suppl 1):**PS9C

**Introduction:** Premature infants in neonatal intensive care receive many painful procedures a day. Although morphine is often used for sedation, its analgesic efficacy is unclear. Poppi was a blinded randomised placebo-controlled trial investigating the efficacy of morphine analgesia for procedural pain in premature infants. A stopping boundary was required to monitor safety with respect to incidences of apnoea requiring intervention, for review by an independent data monitoring committee (DMC) after every 25 infants. Specifying this boundary was a challenge due to a lack of existing methodology for one-sided stopping boundaries for safety.
**Methods:** A pragmatic approach was taken to defining the most appropriate stopping boundary. A range of hypothetical trial scenarios, whereby the frequencies of safety events were varied across the trial arms, were discussed with the trial investigators. Their decisions were plotted, and three equivalent possible stopping boundaries identified. These were based on an assumed average event rate of 7% in the placebo group and an average tolerated difference of 12% between the groups. The DMC were consulted to select a single boundary.
**Results:** A gamma spending function, with a type I error rate of 0.2 and 81% power was chosen. This would allow changes to be made part-way through the trial, for instance, to the frequency of analyses. The trial was stopped after the stopping boundary was crossed, with 3/15 events in the morphine group and 0/15 in the placebo group.
**Discussion:** We took a pragmatic approach to defining this safety stopping boundary, using the clinical expertise of the investigators. This was essential to ensure that the boundary would alert the DMC to potential harm at the right time. It was a useful tool that enabled an informed decision to be made about stopping the trial, in consideration with other trial data.

## PS9D

### - O1 Optimising the efficiency of identifying and addressing trial recruitment issues through pre-trial and 'real-time' qualitative investigation

Leila Rooshenas, Samantha Husbands, Alba Realpe, Jenny Donovan, Fergus Caskey, On behalf of the Prepare for Kidney Care study team
University of Bristol, United Kingdom
*Trials* 2019, **20(Suppl 1):**PS9D

**Introduction:** Qualitative research is increasingly used to enhance the conduct of randomised controlled trials (RCTs) - particularly recruitment. The QuinteT Recruitment Intervention (QRI) investigates recruitment issues in 'real-time' to inform tailored solutions as the trial proceeds. Qualitative research may also be conducted at the pre-trial stage to understand anticipated recruitment issues. We combined pre-trial and 'real-time' (QRI) investigation of recruitment issues in the Prepare for Kidney Care RCT: a challenging trial comparing the (cost)-effectiveness of 'preparing for dialysis' or 'conservative care'. This methodological sub-study aimed to compare the insights/actions generated from each stage of qualitative work, to examine if/how findings could be used to enhance the efficiency of addressing recruitment challenges.
**Methods:** Pre-trial work: observations of introductory site-visits and interviews with clinical professionals (n=16) from forthcoming recruiting sites. 'Real-time' investigation included audio-recordings of recruitment discussions between recruiters/patients (n=38), interviews with recruiters (n=16), and descriptive analyses of screening-log data.
**Findings:** The pre-trial investigation highlighted professionals' concerns around patients holding treatment-preferences, as many sites advocated 'future-treatment-planning' earlier in the disease trajectory relative to the point at which patients became eligible. Professionals also anticipated that patients/relatives may be concerned about forgoing dialysis. This informed refinements to the presentation of the trial arms, whilst issues less amenable to change (e.g. eligibility criteria) became focal points for the 'real-time' investigation. The 'real-time' investigation confirmed that recruiters were reluctant to approach patients with 'future-treatment-plans', which was addressed through specific training/guidance disseminated early on. Unanticipated issues also arose: patients generally held preferences for conservative care, although audio-recorded recruitment discussions indicated this could be shaped by recruiters' information provision. Communication-related feedback/training and guidance around approaching was delivered iteratively, coinciding with improved recruitment per site/month over time.
**Conclusion:** Combining pre-trial and 'real-time' qualitative research can provide an effective means of optimising, through a blend of pre-emptive and responsive actions.

## PS9D

### - O2 Are participant-researcher relationships during complex intervention trials an intervention component, engagement tool or trial retention strategy?

Pat Hoddinott[1], Matthew McDonald[1], Fiona M Harris[1], Rebecca Skinner[1], Stephan U Dombrowski[2], GOS Team[3]
[1]University Of Stirling, Stirling, United Kingdom; [2]University of New Brunswick, New Brunswick, Canada; [3]Game of Stones Team, UK and Ireland
*Trials* 2019, **20(Suppl 1):**PS9D

**Introduction:** Trials of complex interventions typically include participant and researcher contacts for process and outcome measurement. Relationships develop but their interaction with trial interventions, procedures and outcomes has received little attention. Our aim was to examine how researcher-participant relationships contributed to the overall findings of the Game of Stones randomised feasibility trial for weight loss in men.
**Methods:** 105 men with obesity were randomised at two Scottish sites to three groups: text messages with financial incentives (SMS+I), text messages (SMS only), and waiting list control. Weight, behaviours and acceptability outcomes were assessed by a site researcher at 3, 6 and 12-month face-to-face appointments (intervention groups) and 12 months only (control). The same researchers conducted qualitative interviews at 3 months (n=51/55; intervention groups only) and at 12 months (n=33/78; all groups). Qualitative, retention and survey acceptability data were analysed thematically with matrices constructed by trial group, guided by the Framework approach.

**Results:** Themes that appeared to interact with both the intervention and trial outcomes were: valuing continuity of relationships with researchers; a trusting non-judgemental rapport, feeling listened to and supported. Some expressed disappointment with brief assessments at 6 months (no qualitative interview) and with researcher discontinuity when assessor blinding was assessed. More men in the control group (83%) attended at 12 months: SMS only (79%); SMS+I group (64%). Retention related themes were: not wanting to disappoint the good lad/nice lass researchers, the potential for weight-gain-shame, research altruism and understanding science.
**Conclusion:** Participant-researcher relationships can have positive and negative consequences for a trial and supportive accountability was evident. Consideration of relationships in trial design and evaluation is important because there may be unintentional interactions between the intervention, outcomes, qualitative interviews, engagement or retention strategies. This has within and beyond trial implications, including how interventions are translated, implemented and sustained in practice.

## PS9D
### - O3 What worked for us in which circumstances, and what didn't; reflections upon incorporating a realist evaluation within a clinical trial of a complex intervention

Paul Leighton, Janet Darby, Francis Allen, Rachel Evley, Pip Logan
University Of Nottingham, United Kingdom
*Trials* 2019, **20(Suppl 1):**PS9D

**Introduction:** MRC guidelines for the development and evaluation of complex interventions identify the importance of process evaluation in this cycle. Moreover, recent addition has stressed the relevance of theoretically informed evaluation; realist evaluation is one such theoretically informed approach which is popular in applied health research.
FinCH is a multi-centre cluster randomised controlled trial which evaluates the "Guide to Action Care Home" (GtACH) fall prevention programme in care homes. FinCH has recruited more than 1500 participants across 50+ sites, a realist evaluation in 6 settings has considered the delivery of GtACH.
**Methods:** The process evaluation has incorporated observation of training, observation of implementation, stakeholder interviews (managers, staff, residents), and staff focus groups. Outcome data relating to falls and injury has also been reviewed.
Results:
Context-mechanism-outcome (CMO) configurations have been generated to illustrate those circumstances and individual/organisational responses which enable or inhibit GtACH. CMOs which are repeated across multiple sites are identified.
**Discussion:** Despite philosophical foundations which might suggest otherwise, authors have debated the potential for "realist randomised controlled trials" and have advocated the use of realist methods in the development/evaluation cycle.
The FinCH experience has exposed a number of areas where realist methods and randomised controlled trials might sit uneasily.
The iterative nature of sampling in realist evaluation might be compromised by randomisation – the realist wants to carefully select sites so as to explore certain contexts, randomisation might not serve this. Realists also want to complete data collection and analysis prior to commencing a new site, trial recruitment opportunities might not facilitate this.
Realists might value early release of outcome data to support CMO generation, trial protocols might not allow this.
Realist methods have lots to offer in understanding complex interventions, their delivery alongside randomised controlled trials is possible but requires significant planning and preparation.

## PS9D
### - O4 Experiences of providing and receiving sham treatment – the LiTEFORM trial (A Randomised Controlled Trial of the Clinical and Cost Effectiveness of Low Level Laser in the Management of Oral Mucositis in Head and Neck Cancer Irradiation)

Nikki Rousseau[1], Lyndsay Lindley[1], Tim Rapley[2], Holly Fisher[1], Linda Sharp[1], Jenn Walker[1], Janet Wilson[1], Jo Patterson[3], Mike Nugent[3]
[1]Newcastle University, Newcastle upon Tyne, United Kingdom; [2]Northumbria University, Newcastle upon Tyne, United Kingdom; [3]City Hospitals Sunderland NHS Trust, Sunderland, United Kingdom
*Trials* 2019, **20(Suppl 1):**PS9D

**Introduction:** Delivery of sham treatment of non-pharmacological medical technologies may be invasive and arduous for patients. Additionally, procedures to maintain blinding of those delivering the intervention can be complex and demanding. The LiTEFORM trial was a double blind randomised controlled trial of Low Level Laser therapy (LLLT) in the management of oral mucositis in patients undergoing radiotherapy for head and neck cancer. An embedded qualitative study aimed to explore patient and health professional perspectives and experiences of trial participation, including of sham treatment.
**Methods:** Participants attended for LLLT treatment three times a week, timed to coincide with radiotherapy treatment. LLLT took about 30 minutes, with the experience being comparable to a dental examination. Blinding was achieved with the use of randomly allocated sham/active settings on the device, and special glasses for both patients and those administering the LLLT. Qualitative interviews were conducted with staff and with patients shortly after recruitment and at follow up, and a sample of recruitment conversations were recorded.
**Timing of potential results:** Recruitment to the trial is complete, data collection is now in the follow up phase and analysis will be complete by October. Preliminary results suggest that the sham treatment arm did not limit willingness of patients to participate, and that patients felt that even a placebo effect was worth having. However sham treatment was associated with challenges for patients and staff and some patients cited the possibility that they might be receiving sham treatment as a factor in their decision to discontinue LLLT.
**Potential relevance and impact:** Ethical and practical concerns limit the use of sham treatment arms in non-pharmacological studies. Relatively little is known about experiences of sham treatments. Findings from this study will help other researchers to anticipate and overcome challenges and may lead to more robust evaluations of non-pharmacological health technologies.

## PS9D
### - O5 Unique challenges and proposed solutions for designing and conducting pilot and feasibility work to optimise surgical trials

Katherine Fairhurst[1], Shelley Potter[1], Jane Blazeby[1], Carrol Gamble[2], Kerry Avery[1]
[1]Centre for Surgical Research & Medical Research Council (MRC) ConDuCT-II (Collaboration and innovation for Difficult and Complex randomised controlled Trials In Invasive procedures) Hub for Trials Methodology Research, Bristol Medical School, Department of Population Health Sciences, University of Bristol, United Kingdom; [2]Department of Biostatistics & Medical Research Council (MRC) NWHTMR (North West Hub for Trials Methodology Research), University of Liverpool, United Kingdom
*Trials* 2019, **20(Suppl 1):**PS9D

**Introduction:** The value of robust pilot and feasibility studies (PFS) to inform RCTs in surgery is increasingly recognised by funders. Surgical PFS, however are relatively rare, sub-optimal in design and remain poorly reported and disseminated. Qualitative work is needed to explore the challenges and barriers to performing surgical PFS and consider solutions to improve research practice.

**Methods:** Purposive sampling engaged key professional stakeholders involved in designing, funding, conducting and publishing PFS for surgical trials in the UK. Semi-structured interviews performed face-to-face or by telephone using a topic guide, explored experiences and perceptions of surgeons, methodologists, funders and journal editors, around challenges and barriers to undertaking PFS in surgery. A thematic approach to data analysis was performed in an iterative and cyclical process as interviews were completed, until no new themes emerged and/or established themes ceased to evolve.
**Results:** Of 33 contacted, 27 (81%) participants consented and were interviewed in 3 iterative phases: 11 (40%) surgeons, 16 (60%) experienced trial methodologists (mean duration 58minutes, range 27-101). Three quarters (20, 74%) also had current/recent experience on funding body panels. Both surgeons and methodologists acknowledged PFS as vital to address the multiple challenges unique to surgical trials. In contrast to methodologists, surgeons valued PFS more in pragmatic terms (e.g. for gaining main trial funding) and methodological conceptualisations of the types/purpose of PFS were rarely acknowledged. Both groups identified current methodology (e.g. access to CTUs)/funding infrastructure as an obstacle to efficient and timely completion of PFS. Proposed improvements include accessible, funder-endorsed guidance, wider dissemination of PFS findings through efficient funder-driven reporting systems, and methods for enhancing surgeon/methodologist collaborations.
**Discussion:** Differences in understanding exist regarding the purpose and value of PFS for optimising surgical trials. Educating surgeons about PFS and developing guidance to address the unique challenges of PFS to optimise surgical trials are needed.

## PS10A
### - O1 "I was meaning to read that, but…" – An international qualitative study of how time-poor trialists choose their recruitment strategies

Heidi Gardner, Shaun Treweek, Katie Gillies
University Of Aberdeen, Aberdeen, United Kingdom
*Trials* 2019, **20(Suppl 1):**PS10A

**Introduction:** Participant recruitment to trials is challenging. To date research has focussed on recruitment once the trial has started, rather than planning strategies to support it e.g. developing information leaflets with user feedback. We aimed to explore if people involved with participant recruitment have explicit strategies, and if so, how these are developed, and if not, what barriers prevent effective planning.
**Methods:** Design: One-to-one qualitative semi-structured interviews. Data were analysed using a framework approach, themes were linked through comparison of data within and across stakeholder groups.
Participants: 23 trialists; 11 self-identifying as 'Designers'; those responsible for designing recruitment methods, and 12 self-identifying as 'Recruiters'; those who recruit participants. Interviewees' experience with interventions, clinical areas, and recruitment expertise were diverse.
Setting: UK NHS primary, secondary and tertiary-care sites involved in trials, academic institutions (UK, the Netherlands, Canada) and contract research organisations supporting pharmaceutical companies (UK, South Africa, Italy).
**Results:** To varying degrees, respondents had prospective strategies for recruitment. These were never explicitly based on evidence of recruitment benefit.
Two main themes encapsulated barriers to strategy development:
1)Timing of grant applications; time-pressures due to tight deadlines often result in rushed planning followed by an 'amendment cascade' after recruitment commences.
2)Research governance; trialists are often over-burdened with administrative tasks resulting from this amendment cascade.
Themes relating to the types of environments needed to facilitate successful recruitment also emerged; communication and relationship-building within and across teams involved in multi-centre trials; recruitment support, and trialists' workload.
**Discussion:** Respondents from all countries considered limited preparation times and disproportionate approvals processes as major structural challenges to recruitment planning. Poor planning is a mistake that trial teams live with throughout the trial. Effective recruitment requires strategies to increase the time available for trial planning, as well as access to evidence-based strategies that can be straightforwardly implemented.

## PS10A
### - O2 Do investigator meetings improve recruitment into clinical trials? – A retrospective review of data from nine trials

Eleanor Mitchell, Garry Meakin, Kirsty Sprange, Peter Godolphin
Nottingham Clinical Trials Unit, University of Nottingham, Nottingham, United Kingdom
*Trials* 2019, **20(Suppl 1):**PS10A

**Introduction:** Clinical trials often fail to recruit to time and target. A study reviewing strategies to enhance recruitment into clinical trials reported that holding meetings with collaborators is common, offering opportunities for sites to network, receive trial updates, re-train, and share good practice with other sites. Our aim was to investigate the impact of an investigator meeting upon recruitment.
**Methods:** We compared recruitment and investigator meeting data from nine clinical trials managed by Nottingham Clinical Trials Unit, recruiting between 2014-2018. Sites were eligible if UK-based and open to recruitment at least 8 weeks before and after the meeting. Data was collected using a data collection form and from trial databases.
Mixed-effects models were fitted with 16 weeks of recruitment data per site to estimate the change in recruitment after an investigator meeting. Models were fitted for all eligible sites, and only for sites that attended their investigator meeting.
**Results:** Most trials recruited adults only (6/9, 67%) with a median sample size of 269 (1st quartile: 180, 3rd quartile: 517). Meetings occurred throughout the year with at least one in each quarter. The nine trials included 121 sites, 81 were eligible of which fifty-nine (73%) attended their investigator meeting.
There was insufficient evidence to suggest that holding an investigator meeting increased recruitment (weekly recruitment increase per site: 0.07, -0.13 to 0.28). For the 59 sites that attended the investigator meeting, the increase in recruitment was marginally higher (weekly recruitment increase per site: 0.10, -0.17 to 0.37).
**Discussion:** Investigator meetings may improve recruitment in the eight-week period thereafter, with sites that attended seeing one additional participant randomised per 10 sites p/week. However, variability around this estimate may suggest the result is due to chance. A larger study including more trials as well as looking at costs would be useful to investigate further.

## PS10A
### - O3 SWATs at scale: meta-analysis of the results of the first co-ordinated programme of SWATs exploring improvements to patient information in trials

Vichithranie Madurasinghe[1], Peter Knapp[3], Sandra Eldrige[1], Peter Bower[2], on behalf of the START Group
[1]Queen Mary University London, London, United Kingdom; [2]The University of Manchester, Manchester, United Kingdom; [3]University of York, York, United Kingdom
*Trials* 2019, **20(Suppl 1):**PS10A

**Introduction:** There is increasing acceptance that the evidence base for recruitment to trials is poor. Studies-within-a-trial (SWATs) represent a rigorous method of testing recruitment strategies and improving the evidence-base. The recent NIHR HTA SWAT funding mechanism was a welcome stimulus. However, rapidly and rigorously testing recruitment interventions requires a co-ordinated effort to deliver SWATs across multiple studies. Here, we report the completion of the first co-ordinated SWAT programme (START), funded by the MRC.
**Methods:** We identified a recruitment intervention of high priority for testing - patient information sheets optimised by a process of re-design and testing with patients. We recruited 8 trials to test the recruitment intervention using SWAT methodology. We meta-analysed the data to assess the overall impact on recruitment rates, and to explore the degree to which effectiveness varied over different trials
**Results:** 8 trials were recruited from 2014, but only 7 actually delivered data, with one trial reporting two separate comparisons. These were a mix of trials in adults, including both screening and treatment, and physical and mental health. The final data set was not delivered until 2019. Meta-analysis of 28,476 patients across 7 comparisons showed that optimised patient information sheets were associated with an effect of odds ratio = 1.03 (95% CI 0.90 to 1.19). Other outcome measures (responding positively to an invitation, retention) will be presented at the conference.
**Discussion:** Optimised patient information sheets were not associated with improved recruitment. The MRC START programme showed that co-ordinated programmes of SWATs are feasible, although there are major challenges to recruiting trials quickly and ensuring that data are delivered and analysed in a timely fashion to inform decision-making. We discuss the implications of the START programme for the next phase of SWAT development and delivery.

## PS10A
### - O4 Evaluation of the validity and reliability of the DevPIC tool for measuring quality of informed consent discussions during trial recruitment

Julia Wade[1], Elka Humphrys[2], Alba Realpe[1], Jenni Burt[2], Miss Daisy Gaunt[1] , OPTiMISE Study group[2,3], QuinteT research group[1]
[1]University of Bristol, Bristol, United Kingdom; [2]University of Cambridge, Cambridge, United Kingdom; [3]University of Oxford, Oxford, United Kingdom
*Trials* 2019, **20(Suppl 1):**PS10A

**Introduction**: Evidence suggests that discussion is essential for effective information provision for informed consent during trial recruitment. Measures to evaluate information provision assess recruiter information provision or patient information recall of information but not the quality of the interaction or evidence of patient understanding emerging during discussion. The DevPIC tool assesses the quality of information provision as evidenced in recruitment consultations and includes evaluation of patient contributions during the interaction. Initial evaluation of DevPic shows promising validity and reliability applied to an initial set of consultations from secondary care trials.
We are proposing to evaluate the validity and reliability of the DevPIC applied to audio recordings of trial recruitment consultations in the OPTiMISE trial within primary care in collaboration with colleagues not involved in the original tool development. It is part of a programme of work to determine whether it is a practical method to evaluate informed consent in recruitment conversations.
**Methods:** This is a mixed methods study. 18 primary care consultations from OPTIMISE will be analysed using both the published version of the PIC and a parallel thematic analysis. Findings from the two analyses will be used to establish a coding manual to guide application of the measure. This coding manual will be applied in evaluation of a further set of consultations sampled from a pool of 60. Findings will report the feasibility, validity, reliability and stability of the tool.
**Timing of potential results and potential relevance:** Findings reported in October 2019 will report on validity and reliability and how the measure can be used to evaluate the quality of informed consent discussions during recruitment to trials and potential future application to evaluate recruiter training.

## PS10A
### - O5 Why do patients take part in research? An overview of systematic reviews, and mapping to theory and trial recruitment research

Peter Knapp[1], Rebecca Sheridan[2], Petr Bower[3], Adwoa Parker[2], Jackie Martin-Kerry[2], Joanna Hudson[4]
[1]University Of York & The Hull York Medical School, United Kingdom; [2]University of York, United Kingdom; [3]University of Manchester, United Kingdom; [4]King's College London, United Kingdom
*Trials* 2019, **20(Suppl 1):**PS10A

**Introduction:** Understanding why people take part in health research is critical to improve trial efficiency and generalisability. The aim of this overview of systematic reviews was to identify psychosocial determinants of participation and map them to psychological theory and empirical trial recruitment research, to identify effective strategies to increase participation.
**Methods:** Qualitative and quantitative systematic reviews were identified systematically. Methodological quality was rated using AMSTAR and poor quality reviews were excluded. Psychosocial barriers and facilitators were coded to psychological theory (Theoretical Domains Framework) and empirical trial recruitment research (interventions subjected to SWAT evaluation and included in Treweek et al, 2018, Cochrane review).
**Results:** We included 22 systematic reviews (345 primary studies), covering a wide range of populations and settings. We identified five groups of facilitators, of which three were dominant (potential for personal benefit; altruism; trust) and relevant across research setting and design. We identified nine groups of barriers, which were more dependent on the particular study (context, population and design). Two determinants (participant information; social influences) were found to be both barriers and facilitators. Barriers and facilitators could be coded to the Motivation and Opportunity components of the Theoretical Domains Framework; only one was coded to a Capability component. There was limited overlap between psychosocial determinants and empirical trial recruitment research, and some barriers and facilitators had not been tested at all.
**Discussion:** Our synthesis of the main psychosocial determinants of research participation is useful knowledge for clinicians, researchers and research ethics organisations. Identifying barriers and facilitators should make it possible for adjustments to be made to trial design to anticipate them. Mapping determinants to theory offers the potential for greater understanding of decision-making and opportunities for intervention design. The current lack of overlap between some psychosocial determinants and empirical research also offers the potential to guide intervention development.

## PS10B
### - O1 Health informatics (HI) innovations in randomised trials and clinical cohorts - Identification, screening, stratified care and data collection during primary care consultations

Simon Wathall[1,2], Nadine Foster[1], Jonathan Hill[2], Kika Konstantinou[2,3], Sarah Lawton[2], Sara Muller[2]
[1]Keele Clinical Trials Unit, Keele University, UK; [2]Primary Care Centre Versus Arthritis, Research Institute for Primary Care & Health Sciences, UK; [3]Midlands Partnership NHS Foundation Trust, Staffordshire, UK
*Trials* 2019, **20(Suppl 1):**PS10B

**Introduction:** Recruitment and retention of participants remain the main challenges for clinical research, particularly for primary care studies undertaken at point-of-care where consultation time is pressured.

The Health Informatics (HI) team at Keele Clinical Trials Unit (CTU) has developed tools which are embedded into GP clinical IT systems, to facilitate the conduct of trials and cohort studies in real-time GP consultations.

**Methods:** HI clinical system protocols were developed to efficiently utilise patients' electronic primary care medical record to automate processes such as patient identification and eligibility screening whilst remaining as unobtrusive as possible for the clinician end-user.

Protocols and templates are developed to embed stratified care tools into the consultation to guide treatment decision-making, and automate coding of patient eligibility, consent and outcome/trial data into patients' primary care records.

**Results:** Automated coding facilitates efficient and accurate data recording for a number of clinical research studies. Regular audits allow the level of GP engagement and efficiency with recruitment and the study intervention to be assessed. Three exemplar studies will be presented, all of which had bespoke point-of-care HI templates that have facilitated research delivery in busy GP consultations.

Arthritis Research UK funded PMR Cohort Study (Polymyalgia Rheumatica – a low incidence condition):

386 GP practices, suitable for invitation n=739

NIHR HTA funded SCOPiC Trial (SCiatica Outcomes in Primary Care):

42 GP practices, HI protocol fires n=19,375, eligible n=3,963, suitable for invitation n=2,677

NIHR PGfAR funded STarT MSK Pilot trial:

8 GP practices, HI protocol fires n=3,063, eligible n=1,653, suitable for invitation n=1255.

**Discussion:** Utilising GP clinical IT systems to embed HI research templates has resulted in efficient recruitment to randomised trials and cohort studies. Consideration needs to be given to clinical coding, training of clinical end-users, consultation styles and auditing system usage behaviour to ensure these HI solutions are successful.

## PS10B

### - O2 Utility of routine electronic health records used as outcome measures in UK randomised trials: a systematic review

Sharon Love[1], Sarah Lensen[1], Archie Macnair[1], Graham Powell[3], Victoria Yorke-Edwards[1], Elizabeth Williamson[2], Matthew R Sydes[1], James Carpenter[1,2]

[1]MRC Clinical Trials Unit at UCL, London, United Kingdom; [2]The London School of Hygiene and Tropical Medicine, London, United Kingdom; [3]University of Liverpool, Liverpool, United Kingdom

*Trials* 2019, **20(Suppl 1):**PS10B

**Introduction:** There is a wave of interest in, and resources for, unleashing the potential for routine electronic health records (EHR) to support medical research. In the context of randomised controlled trials, EHR data can be used to supplement or replace established data collection procedures. We undertook a systematic review of trials accessing EHR data in the UK, to characterise these trials and the ways they are using (or planning to use) these data.

**Methods:** Potential sources of EHR were defined as health databases/sets held by national organisations, registries or audits that are not involved in direct patient care; such as CPRD and NHS Digital. A list of all trials accessing these EHR between 2013-2018 was developed through screening public release registers, database websites, and direct contact with database staff. Trials were eligible if the released data was pertaining to individuals randomised to a trial; for example, data access solely to identify eligible patients for recruitment was excluded. Information was sought on trial characteristics (e.g. sample size, disease area), the EHR data received (registry source, datasets accessed) and how the data was used, from all available sources (e.g. publication, trial websites). Data were extracted onto a piloted form and entered into a Macro database. All screening and data extraction was undertaken in duplicate, independently with analyses done in Stata. PROSPERO: CRD42019123088.

**Timing of Potential Results:** Data extraction ongoing Mar-2019 to Jun-2019. Results available from Aug 2019.

**Potential Relevance and Impact:** There are many efforts and resources directed towards increasing the accessibility and quality of "big data" in healthcare, however the extent to which UK trials are using this data has not been described. This review will characterise the current use of EHR in UK trials to supplement or replace trial outcome data, and explore the scope for use in future trials.

## PS10B

### - O3 Routinely-collected hospital datasets can be used to identify endpoints predictive of overall survival outcomes in randomised controlled trials (RCT): a prostate cancer study within the STAMPEDE protocol (NCT00268476)

Harriet P Mintz[1,7], Helen M Parsons[1], Prashant Patel[2,7], Claire Amos[3], Rachael Brannan[4], Christopher Brawley[3], Joanna Calvert[3], Melissa R Gannon[5], Luke Hounsome[4], Fiona Ingleby[3], Sean McPhail[4], Mahesh KB Parmar[3], Mary Rauchenberger[3], Haiyan Wu[6], Matthew R Sydes[3], Nicholas D James[2,7]

[1]Warwick Medical School, University of Warwick, Coventry, United Kingdom; [2]Institute of Cancer and Genomic Sciences, University of Birmingham, Birmingham, United Kingdom; [3]MRC Clinical Trials Unit at UCL, Institute of Clinical Trials & Methodology, UCL, London, United Kingdom; [4]Public Health England, London, United Kingdom; [5]Department of Health Services Research and Policy, London School of Hygiene and Tropical Medicine, London, United Kingdom; [6]HDR UK, UCL Institute of Health Informatics, London, United Kingdom; [7]University Hospitals Birmingham NHS Foundation Trust, Birmingham, United Kingdom

*Trials* 2019, **20(Suppl 1):**PS10B

**Introduction:** Validated endpoints that appear earlier, and are more information rich than overall-survival (OS), are desirable for oncology RCTs e.g. progression, metastases, failure-free survival and skeletal-related events (PFS, MFS, FFS, SRE). We present work, embedded in a large multi-arm multi-stage trial, STAMPEDE, using routine data to test alternative RCT analyses.

**Methods:** An algorithm using routine data (Hospital Episode Statistics: HES) was developed using iterative training and validation, creating a HES-derived endpoint predicting OS benefits. Intense note review was undertaken during training, (N=47) and the HES-derived endpoint was compared to the STAMPEDE-endpoints, which was also undertaken during validation (N=46). Standard survival analysis methods compared interventions (+/-docetaxel) (N=93). Multi-site validation is near completion (N=2,949).

**Results:** The algorithm was based on HES-derived cancer-related event activity, over 8-week time periods. We could not reliably identify individual endpoint events but identified a composite activity-based endpoint. During validation (N=46), 36/46 patients experienced a confirmed MFS, FFS or PFS event, reported in the trial. In 30/36, the HES algorithm corresponded to at least one trial endpoint, including 14/33 FFS, 23/29 PFS and 22/28 MFS events. Docetaxel effects were similar to the traditional STAMPEDE endpoints (N=93) with the HES-activity endpoint: HES-HR=0.59 (95%CI: 0.33-1.03); STAMPEDE-FFS-HR=0.51 (95%CI: 0.29-0.89); based upon 66/93 HES and 67/93 STAMPEDE-FFS events occuring. The final multi-site validation on 2,949 patients will be presented at the meeting.

**Discussion:** Although traditional endpoints could not be identified, we developed a novel HES-activity-based endpoint: "hospital activity-free survival". This identified trial events and some unreported events and enabled estimation of treatment effects. If validated, routine data is feasible for analysis, reducing costs, resources, and patient/clinician follow-up burden, increasing efficiency. Our novel activitybased efficacy indicator may be desirable to patients (e.g. identifying burdensome healthcare interactions) and could correlate with health-related quality-of-life; further work is investigating this.

## PS10B

### - O4 Getting animated about routine data: Using animations to inform and engage future trial participants about linkage to routinely collected data to aid recruitment

Fiona Lugg-widger, Lianna Angel, Peter Gee, Jeremy Segrott, Mike Robling
Cardiff University, United Kingdom
*Trials* 2019, **20(Suppl 1):**PS10B

**Introduction:** The use of routinely held health, social care and other public health records has become an important feature of the research landscape and offers significant potential to advance knowledge across a wide range of clinical and public health domains. The involvement and engagement of this topic input with the public is key if we are to be successful in recruiting to studies using routine data/data linkage.
The aim of this work is to co-produce with members of the public an 'animation package' that researchers can use to increase public (including future trial participants) understanding of and engagement in research using routine data including aiding recruitment.
**Methods**: We have developed an animation by working with a group of young mothers and a group of 14-25 year olds in the South Wales area. The next step is to test and improve the animation through wider engagement with stakeholders including users of and receivers of the animation package. A number of stakeholder groups have been formed: 1) those who recruit participants; 2) Trial managers; 3) study lay representatives and 4) members of the public. They will provide input on their understanding of the key concepts described in the animation, its utility as a trial recruitment tool to enable informed consent when using routine data and the best format/media for its use over the long-term.
**Timing of Results:** The animation package will be finalised in Autumn 2019 and the feedback, utility and final product will be presented.
**Relevance & Impact:** The outputs from this work will be available and accessible for researchers to use as part of the information provided at study registration to facilitate recruitment when using routinely collected data and data linkage.

## PS10B

### - O5 Does regulation of routine data sharing pose a risk for Individual Patient Data (IPD) meta-analysis? A review of some key challenges in a UK context

Michael Robling, Fiona Lugg-Widger, Fergus Macbeth, Jo Smith, Richard Adams
Cardiff University, Cardiff, United Kingdom
*Trials* 2019, **20(Suppl 1):**PS10B

**Introduction:** Maximising primary research outputs through greater sharing of trial data is a key element of the Open Research agenda. Similarly, UK policy interest in routine data includes greater use of routine data in trials. Meanwhile key providers of routine data (eg NHS Digital) have modified their approaches to data sharing driven by regulatory requirements. But, how well do these developments sit alongside each other to promote access? We focus on use of shared routine data for individual participant data (IPD) meta-analyses. We aim to identify current challenges and where these may be addressable through organisational change.
**Methods:** Using a case study approach we sampled studies from our Centre that sourced routine data and plan to share individual level data further. This includes both secondary analysis and IPD meta-analysis. For each we clarify how legal requirements and organisational governance serves to support or hinder further sharing of routine data. We identify what options exist for researchers intending to contribute trial data and apparent pros and cons of these approaches. We talk with data providers and investigators to elicit their understanding of the issues and potential solutions.
**Timing of potential results:** Completed case studies will be ready for presentation in autumn 2019.
**Potential relevance and Impact:** Mapping organisational and regulatory challenges to IPD for trials using routine data will identity weaknesses in the evolved architecture for data sharing. This will help researchers better plan for data sharing (eg drafting participant information sheets, primary data sharing agreements) including alternative sources of primary data (eg hospital sites rather than data centres). Current experiences of data sharing for IPD involved researcher uncertainty, high access costs and significant delays in access. For IPD still to be an effective and deliverable tool under current regulations requires dialogue with providers and regulators, acknowledging barriers and developing solutions.

## PS10C

### - O1 Estimating treatment effects in the presence of informative missingness

Ruwanthi Kolamunnage-Dona
University of Liverpool, United Kingdom
*Trials* 2019, **20(Suppl 1):**PS10C

**Introduction:** In many clinical trials with longitudinal outcome data, a common situation is where some patients withdraw or dropout from the trial before completing the measurement schedule but the dropout may be non-ignorable or informative. However, the standard methods for analysing longitudinal outcome data ignore the reasons for dropout, which could result in a biased comparison between the treatment groups.
**Methods:** Joint modelling of longitudinal and event-time outcome processes has gained its popularity in last decade as they yield more accurate and precise estimates. However, adopting this framework in clinical trials has been limited. We explore the impact of informative dropout on the evaluation of treatment effect in the MAGNETIC trial; the largest randomised placebo-controlled study to date comparing the addition of nebulised magnesium sulphate to standard treatment in acute severe asthma in children.
**Results:** The primary outcome of the MAGNETIC trial was Yung Asthma Severity Score (ASS). It was measured at baseline and 20, 40, 60, 120, 180 and 240 minutes following randomisation, and the amount of missingness was over 5%. The reasons for missingness were sometimes clearly related with study withdrawal due to good or poor prognosis of the child, but in many instances these reasons were unclear. The results from simple approaches such as complete case analysis and the proposed joint modelling are compared in the context of evaluating the treatment effect.
**Discussion:** Ignoring the informative nature of the missing outcome data could result in inaccurate estimation of treatment effect in clinical trials. We emphasise the importance of undertaking an appropriate statistical analysis accounting for such missing outcome data. The joint modelling is proposed as a general approach for evaluating the sensitivity of conclusions to assumptions regarding missing data in clinical trials with longitudinal outcomes.

## PS10C

### - O2 Reference-based multiple imputation for data missing not-at-random in cost-effectiveness analysis

Baptiste Leurent[1], Manuel Gomes[2], Suzie Cro[3], Nicola Wiles[4], James Carpenter[1,2]
[1]London School of Hygiene and Tropical Medicine, London, UK; [2]University College London, London, UK; [3]Imperial College London, London, UK; [4]University of Bristol, Bristol, UK
*Trials* 2019, **20(Suppl 1):**PS10C

**Introduction:** Missing data are ubiquitous in cost-effectiveness analyses (CEA) of randomised trials, and are often addressed under the 'Missing-at-random' (MAR) assumption. However, this assumption is untestable and sensitivity analyses are required to assess the implications of departures from MAR. Reference-based multiple-imputation (MI) is an attractive approach which formulates the departures from MAR by imputing from a specific reference group. For example, a plausible not-at-random mechanism in a placebo-controlled trial would be to assume that participants in the experimental arm who drop out and stop taking their treatment, have similar outcomes to those in the placebo arm.
**Methods:** We extended the reference-based MI approach to jointly accommodate missing cost and effectiveness data in a multivariate MI framework, implemented in Stata. The approach allows for different missing data assumptions for cost versus effectiveness endpoints. We illustrated the approach using data from the CoBalT trial, which evaluated the cost-effectiveness of cognitive behavioural therapy (CBT) in addition to usual care for primary care patients with treatment-resistant depression.
**Results:** The trial enrolled 469 participants and 101 (22%) had missing cost or effectiveness (QALY). Under MAR, the difference in QALY between the CBT and usual care arm was 0.088 (95%CI: 0.035 to 0.142), and difference in cost £996 (£802 to £1,190). When we assumed that patients in the active arm behaved similarly to those in the control arm after dropping-out, the differences were reduced to 0.079 (0.025 to 0.134) and £813 (£630 to £996) respectively, but CBT remained more cost-effective than usual care (at £20,000/QALY). This was confirmed under a range of other missing data assumptions, including 'baseline-mean-carried-forward' which assumed participants jumped back to the baseline mean level, after drop-out.
**Discussion:** This study extends reference-based MI to trial-based CEA, and illustrates its flexibility and accessibility for conducting sensitivity analysis to departures from MAR in this context.

## PS10C
### - O3 A framework for extending trial design to facilitate missing data sensitivity analyses

Alexina Jane Mason[1], Richard D Grieve[1], Alvin Richards-Belle[2], Paul R Mouncey[2], David A Harrison[2], James R Carpenter[1,3]
[1]London School of Hygiene & Tropical Medicine, London, United Kingdom; [2]Intensive Care National Audit & Research Centre (ICNARC), London, United Kingdom; [3]MRC Clinical Trials Unit at UCL, London, United Kingdom
*Trials* 2019, **20(Suppl 1):**PS10C

**Introduction:** Missing data are an inevitable challenge in Randomised Controlled Trials (RCTs). Methodological guidance recommends that studies should undertake sensitivity analyses which recognise that data may be 'missing not at random' (MNAR), and these can be informed by elicited expert opinion. However, few published trials plan and undertake the required elicitation exercises. The aim of this research is to provide a framework that anticipates and allows for MNAR data in the design and analysis of clinical trials.
**Methods:** The framework was developed and critically examined within the POPPI trial, which investigated whether a preventive, complex psychological intervention, commenced early in ICU, would reduce the development of patient-reported post-traumatic stress disorder symptom severity, and improve health-related quality of life. In particular, we performed and used expert elicitation to frame sensitivity analyses for the missing outcome data. This required addressing key practical challenges that arise when adopting this approach in trials: the criteria for identifying relevant experts, the outcome scale for presenting data to experts, the appropriate representation of expert opinion, and the evaluation of the elicitation results.
**Results:** The framework includes the following steps: defining the scope of the elicitation exercise, developing the elicitation tool, eliciting expert opinion about the missing outcomes, evaluating the elicitation results, and analysing the trial data. For the POPPI trial, 113 experts were asked to participate in the elicitation exercise, providing 59 usable responses. The sensitivity analyses found that the results from the primary analysis were robust to alternative MNAR mechanisms, based on a range of pooled and individual expert opinion.
**Discussion:** Future studies can adopt this framework to embed expert elicitation within the design of clinical trials. This will provide the information required for MNAR sensitivity analyses that examine the robustness of the trial conclusions to alternative, but realistic assumptions about the missing data.

## PS10C
### - O4 Methods to deal with missing data in area under the curve outcomes in randomised controlled trials: the OPEN trial case study

Beatriz Goulao[1], Graeme MacLennan[2]
[1]Health Services Research Unit, University of Aberdeen, Aberdeen, United Kingdom; [2]Director of the Centre for Health Care Randomised Trials, University of Aberdeen, Aberdeen, United Kingdom
*Trials* 2019, **20(Suppl 1):**PS10C

**Introduction:** The area under the curve (AUC) is used to summarise longitudinal outcomes in RCTs. When follow-up time points are (nominally) anchored to time zero by randomisation, within-person missing data is easily handled. In the OPEN trial, comparing two surgical options for men with ureteral stricture, additional outcome data was collected at time points anchored to index surgery and/or reintervention that varied from participant to participant. Missing data at these "floating" time points is more challenging to deal with.
**Methods:** In OPEN the primary outcome was the AUC of ICIQ collected at fixed (baseline, 18, 24-months post-randomisation) and floating time points (pre-surgery, 1-week post catheter removal, 3, 6, 9, 12, and 24-months post-surgery). The analysis strategies were: 1) any available data (at least two ICIQ); 2) minimal data (one early and one late ICIQ measure, extrapolating between observed time points); 3) multiple imputation (MI) at the participant level; 4) MI at time-point using different auxiliary models for fixed and floating measures. We used linear regression in Stata 15. Simulation work will assess the statistical properties of each strategy.
**Results:** Participants were randomised to urethroplasty (108) or urethrotomy (112); 104 vs 93 participants had at least two ICIQ and 90 vs 69 had minimal data. The complete case estimate of difference in AUC was -0.51 95% CI (-1.89, 0.87). For minimal data it was -0.36 95% CI (-1.74, 1.02); MI at the participant level -0.33 95% CI (-1.74, 1.09); MI at time-point level -0.69 95% CI (-2.11, 0.72).
Discussion: A range of analyses to deal with missing data in the OPEN trial produced similar results with comparable precision. Our simulation work is ongoing to explore how these approaches performs in a range of scenarios.

Download to read this full article text.

Publisher’s Note

Springer Nature remains neutral with regard to jurisdictional claims in published maps and institutional affiliations.

